# Transcriptome Profiling of Citrus Fruit Response to Huanglongbing Disease

**DOI:** 10.1371/journal.pone.0038039

**Published:** 2012-05-31

**Authors:** Federico Martinelli, Sandra L. Uratsu, Ute Albrecht, Russell L. Reagan, My L. Phu, Monica Britton, Vincent Buffalo, Joseph Fass, Elizabeth Leicht, Weixiang Zhao, Dawei Lin, Raissa D'Souza, Cristina E. Davis, Kim D. Bowman, Abhaya M. Dandekar

**Affiliations:** 1 Plant Sciences Department, University of California Davis, Davis, California, United States of America; 2 U.S. Horticultural Research Laboratory, United States Department of Agriculture, Agricultural Research Service, Fort Pierce, Florida, United States of America; 3 Mechanical and Aerospace Engineering Department, University of California Davis, Davis, California, United States of America; 4 Center for Computational Science and Engineering, University of California Davis, Davis, California, United States of America; 5 Bioinformatics Core, Genome Center, University of California Davis, Davis, California, United States of America; 6 Dipartimento di Sistemi Agro-Ambientali, Università degli Studi di Palermo, Viale delle Scienze, Palermo, Italy; Naval Research Laboratory, United States of America

## Abstract

Huanglongbing (HLB) or “citrus greening” is the most destructive citrus disease worldwide. In this work, we studied host responses of citrus to infection with *Candidatus* Liberibacter *asiaticus* (CaLas) using next-generation sequencing technologies. A deep mRNA profile was obtained from peel of healthy and HLB-affected fruit. It was followed by pathway and protein-protein network analysis and quantitative real time PCR analysis of highly regulated genes. We identified differentially regulated pathways and constructed networks that provide a deep insight into the metabolism of affected fruit. Data mining revealed that HLB enhanced transcription of genes involved in the light reactions of photosynthesis and in ATP synthesis. Activation of protein degradation and misfolding processes were observed at the transcriptomic level. Transcripts for heat shock proteins were down-regulated at all disease stages, resulting in further protein misfolding. HLB strongly affected pathways involved in source-sink communication, including sucrose and starch metabolism and hormone synthesis and signaling. Transcription of several genes involved in the synthesis and signal transduction of cytokinins and gibberellins was repressed while that of genes involved in ethylene pathways was induced. CaLas infection triggered a response via both the salicylic acid and jasmonic acid pathways and increased the transcript abundance of several members of the WRKY family of transcription factors. Findings focused on the fruit provide valuable insight to understanding the mechanisms of the HLB-induced fruit disorder and eventually developing methods based on small molecule applications to mitigate its devastating effects on fruit production.

## Introduction

Huanglongbing (HLB) or “citrus greening” is the most destructive citrus disease today [1], [2]. HLB is caused by phloem-limited bacteria of the genus *Candidatus* Liberibacter. Three species of this pathogen are currently associated with the disease in citrus. The Asian form, *Candidatus* Liberibacter *asiaticus* (CaLas), is found in all HLB-affected countries outside Africa. The African form, *Ca*. L. *africanus* (CaLaf), and the American form, *Ca*. L. *americanus* (CaLam), are currently found only in Africa and Brazil, respectively [1]. CaLas, present in Asia, Brazil, Florida, and Louisiana, has recently been cultured [3]. The genome sequence of CaLas was obtained using a metagenomic approach from vascular tissues [4] and an infected psyllid [5]. CaLas is transmitted by *Diaphorina citri* Kuwayama, the Asian citrus psyllid (ACP). Typical symptoms of citrus greening disease include blotchy, mottled, and variegated leaf chlorosis, followed by tree decline. Infected leaves become upright, followed by leaf drop and twig dieback at later stages. Early flowering is also observed in CaLas-infected sweet orange. Initial symptoms are very similar to those caused by mineral nutrient stresses such as zinc, magnesium, or iron deficiency, hampering accurate diagnosis.

Understanding host responses to pathogen infection is essential to clarify the mechanisms of plant-microbe interactions and to develop novel strategies for therapy. Studies have been conducted to identify key genes and proteins induced by HLB in leaf tissues [6], [7], [8]. These studies showed that key pathways and processes such as cell defense, transport, photosynthesis, carbohydrate metabolism, and hormone metabolism are affected by the disease [6]. Clarifying host responses and disease development in fruit peel and leaves is critical for disease detection at the earliest stages. CaLas is typically found in leaves, but can be present in bark, root, flower (petal and pistil), and fruit (peduncle, columella, and seed coat) of infected trees [9]. Although pathogen detection is typically conducted on leaf tissues, fruit peel can also be employed for the analysis of host responses. The analysis of host responses in both leaves and fruits is important to understand the mechanisms of the fruit disorder and tree decline induced by the disease.

CaLas-infected peel tissues often show a characteristic color inversion as the fruit changes from green to yellow/orange [1]. HLB also results in fruits that are small, asymmetric, and lopsided, with a bent fruit axis, small or aborted seeds, and a strong yellow to brown stain in vascular bundles within the axis at the peduncular end [2].

Microarray technology has been used in numerous studies of host response to infection by pathogens including bacteria, viruses, and fungi [10], [11], [12]. However, this technology can reveal the expression of only those genes represented on the array. Possible misleading interpretations of microarray results can occur due to non-specific hybridization. Next-generation DNA sequencing technology can reveal very rare and unknown transcripts, offering a more precise and accurate picture of the transcriptome. These tools, already applied to plants [12], [13], [14], [15], assume extensive prior knowledge of the organism under investigation. For plant species that lack whole-genome sequence information, an extensive EST database is required. Misleading results due to multiple mapping locations for the same sequence might occur. Indeed, data obtained are usually confirmed with qRT-PCR analysis or integrated with proteomic and metabolomic analyses. In addition, analysis of the deep transcriptome profile using biological network theory can help define gene regulatory networks. Protein networks are increasingly used to describe the molecular basis of disease-related subnetworks [16] and to define protein-protein interaction networks (PPI) that regulate disease resistance in plants and plant-pathogen interactions [17]. Bioinformatic tools are now available for visualizing and characterizing statistical properties of these networks (e.g. the BNArray, GeneReg and igraph packages of R, and other software such as Centibin, Graphviz, Pajek, and Cytoscape).

At present, no therapeutic treatments are available for HLB, and removal of infected trees and insect control are the main management strategies to limit or prevent its spread. Traditional diagnostic approaches rely on symptom recognition in the field, confirmed by PCR based on primers developed for individual *Candidatus* Liberibacter species. [18], [19], [20], [21]. These practices were very useful to speed up pathogen detection and accelerate management procedures, although the pathogen may elude detection at asymptomatic stages [21], [22], [23]. This is probably due to the fact that the pathogen is phloem-limited and not uniformly distributed within the tissues of infected trees [9].

This study examines global changes in host gene expression due to CaLas infection in fruit peel. It aims to elucidate metabolic changes induced by the disease in the fruit. Using next generation sequencing technology, mRNA transcripts from fruit peel sample types representing various stages of disease were compared. Fruits displaying symptoms were compared to asymptomatic fruits from the same tree and to apparently healthy fruits taken from trees free of HLB symptoms in the same orchard. Fruits were categorized into one of the three stages based on qRT-PCR in addition to commonly observed symptoms.

## Results

### Transcriptome profiling using RNA-Seq

Between 24 and 41 million 85-nt paired-end reads were obtained from each of four cDNA libraries derived from mature fruit peel at different disease stages: fruit peel from uninfected trees in an orchard with no HLB present; fruit peel from apparently healthy trees (PCR-positive at the time of sample collection, healthy without any symptoms) in an orchard with HLB; symptomatic and asymptomatic fruit peel from the same trees infected with CaLas (PCR-positive with HLB symptoms). These reads were aligned to the NCBI citrus unigene set, with 42 to 46% of reads per sample mapping to a unigene. Six pairwise comparisons were made between symptomatic, asymptomatic, apparently healthy, and healthy control fruit peel to calculate changes in expression (log fold ratio) of individual genes (Tables S1, S2, S3, S4, S5, S6). The comparison between asymptomatic and symptomatic stages of the disease identifies genes related to the appearance of symptoms. Interestingly, the overall expression profiles of apparently healthy and asymptomatic fruit peel were very similar, indicated by fewer differentially expressed transcripts in this comparison.

The four fruit types were also tested for the presence of Citrus Tristeza Virus (CTV) using CTV CP reference sequence T36 (M76485) to show cross-responses to multiple pathogens. CTV was detected in all four sample types. No significant differences were observed among the three fruit categories from the infected orchard (Figure S1).

### Functional analysis of RNA-Seq data

was performed to determine how the four fruit types can be separated based on their overall transcriptome profile (Figure S2). Linear combination of transcriptomic data generated vectors or groups to best explain overall variance in the data set without prior assumptions about whether and how clusters might form. It was clearly evident that apparently healthy and asymptomatic fruits showed close similarities and were separated from the other two fruit types. Healthy fruits and symptomatic fruits were clearly distinguished from each other. The 21 target genes most specific to each category are listed in Table S7. Three complementary methods were used for functional analysis of the transcriptomic data: Fisher's Exact Test and Gene Ontology (GO) descriptions, PageMan gene set enrichment analysis, and pathway enrichment analysis using Pathexpress.

**Figure 1:**
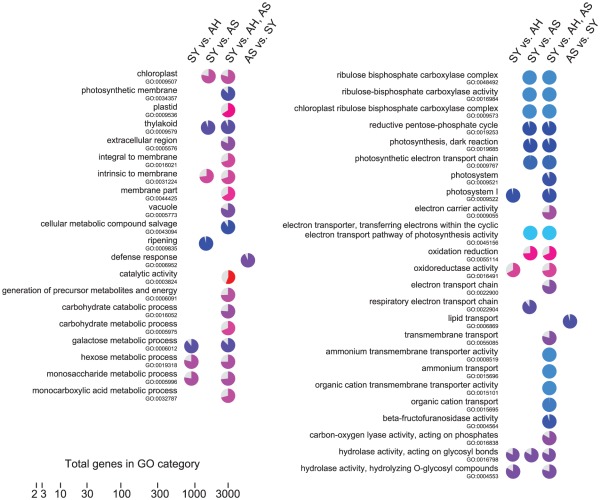
Fisher's Exact Test analysis. Over-represented GO terms in four pairwise comparisons: symptomatic (SY) vs. apparently healthy (AH), symptomatic vs. asymptomatic (AS), symptomatic vs. apparently healthy and asymptomatic, asymptomatic vs. symptomatic. The size of the colored sector of each pie chart corresponds to the proportion of that GO term in the test set compared to all the genes to which that GO term has been assigned, where 100% colored means all of the genes in the reference set are in the test set. Colors correspond to the number of genes in the GO category on a log scale, as indicated by the scale bar.

The Fischer Exact Test as provided in Blast2GO [24] is useful to determine the specific GO terms affected by the disease. Some GO terms were significantly over-represented among differentially expressed genes obtained from pairwise comparisons (Table S8). Among these, several GO terms associated with cell wall biogenesis, modification, and organization and related metabolic processes were over-represented among more abundant transcripts in healthy control fruit than in apparently healthy, asymptomatic, or symptomatic fruit. Interestingly, GO terms related to photosynthetic reactions (Photosystem I and II, rubisco activity, thylakoid localization) were over-represented in fruits showing the typical symptoms of HLB infection. Over-represented GO terms in four pairwise comparisons (symptomatic vs. apparently healthy, symptomatic vs. asymptomatic, symptomatic vs. apparently healthy and asymptomatic, and asymptomatic vs. symptomatic) were analyzed to determine which GO terms correlated significantly with HLB disease (Figure 1). It is interesting to note that when symptoms are clearly evident, GO terms of small carbohydrate metabolism (monosaccharide, sucrose, and galactose catabolic and metabolic processes) and other important pathways of primary metabolism such as the pentose-phosphate cycle are over-represented. As expected from visual analysis of fruit symptoms, many gene functions related to photosynthesis were over-represented at the symptomatic stage (e.g., electron carrier activity, chloroplast, photosystems, ribulose bisphosphate carboxylase complex and activity, thylakoid, photosynthetic dark reaction) compared with all other conditions. GO terms for ion transport were also over-represented at the symptomatic stage. The high number of GO terms for oxidoreductase activity is consistent with the hypothesis that the disease induces oxidative stress. Several categories of GO terms involved in vesicle transport and cell wall biogenesis, metabolism, and organization were over-represented among genes expressed at the symptomatic stage. Interesting defense-related and lipid transport GO terms were over-represented at the asymptomatic stage.

PageMan software [25] was used to visualize functional classes that were significantly affected by HLB disease (Figure S3). This method pinpoints which subcategory of genes were upregulated and downregulated in each main gene category based not only on metabolic pathways but also on cell functions. Increased expression in diseased samples is seen in photosynthesis, N-metabolism, amino acid synthesis, isoprenoids, jasmonate and salicylic acid, and several transcription factors. Functional classes with decreased expression include sucrose and starch biosynthesis, glycolysis, gibberellins, DNA and protein synthesis, and flavonoids metabolism. Additional changes in expression can be seen in the comparison between samples from the disease-free location and apparently healthy fruits (asymptomatic but from the infected orchard). These changes, eventually affected by environmental variability, probably were induced in early stages of HLB disease. Differential expression of transcripts comparing different stages of HLB infection and their functions were visualized using MapMan software [26]. This provided more specific information on pathways and functions identified by Fisher's Exact Test and PageMan. For MapMan data analyses, a mapping file composed of NCBI *Citrus sinensis* unigenes was used.

**Figure 2:**
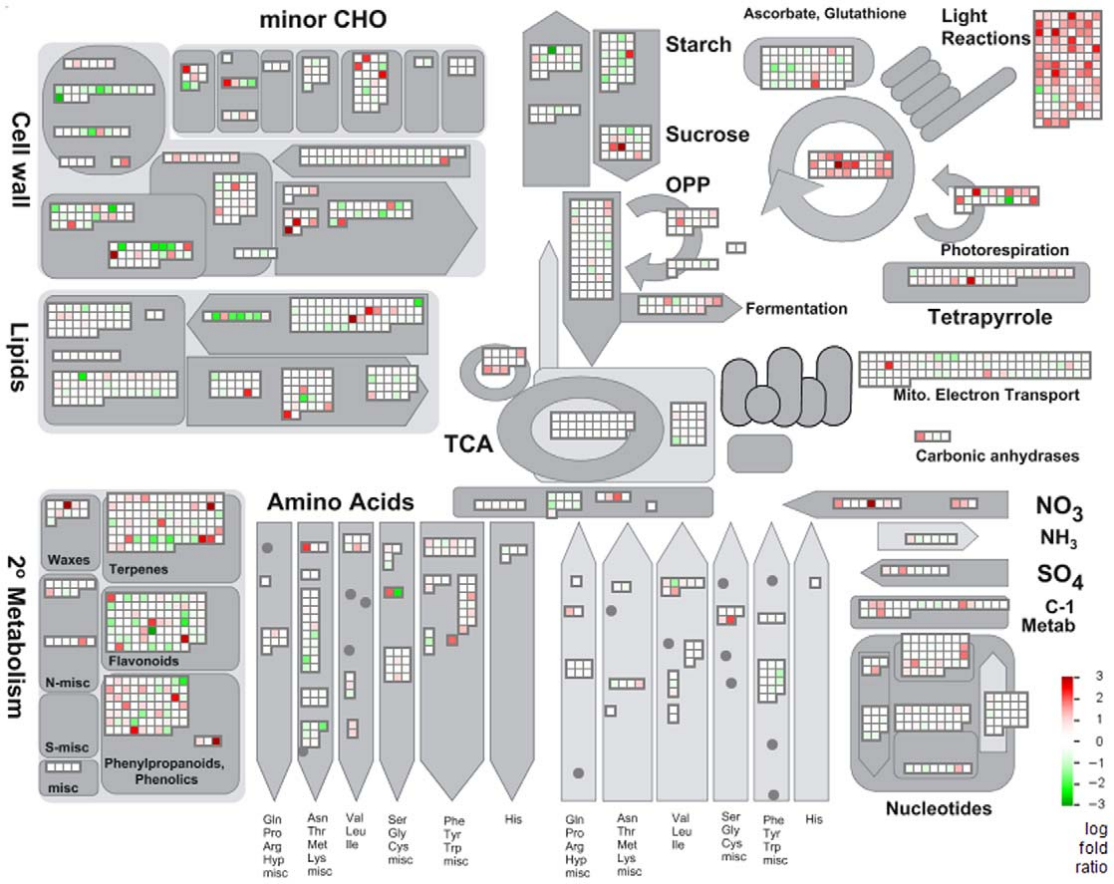
Functional categorization of differentially regulated genes in symptomatic fruits. Metabolism overview in MapMan depicting differential gene expression in symptomatic and apparently healthy fruits from the infected orchard. Log fold ratios are indicated as a gradient between red (up-regulated) and green (down-regulated).

The third enrichment method was conducted using the Pathexpress web-tool [27] to determine which metabolic pathways were significantly affected by the disease by comparing symptomatic and asymptomatic fruits (Table 1). The MapMan graphical metabolic overview identifies transcripts that are differentially expressed in symptomatic and apparently healthy fruit, with each colored square representing a single annotated gene in a particular pathway (Figure 2). Several genes involved in light reactions of photosynthesis, mitochondrial electron transport, sucrose metabolism, glycolysis, and fermentation were up-regulated. In contrast, gene transcripts for cell wall modification and degradation, pectin esterase activity, and cellulose synthesis were mostly down-regulated. We also identified several differentially expressed genes involved in secondary metabolic pathways, including terpenes, flavonoids, and phenylpropanoids.

**Table 1:** Differentially regulated pathways (up- and down-regulated) involved in HLB response in the symptomatic fruit as determined using Pathexpress.

Regulated Pathways	Between Trees	Within Tree
Starch and sucrose	5*10^−4^	0.03
Carbon fixation	9*10^−4^	0.02
Ascorbate and aldarate	0.03	n.s.
Phenylpropanoid	0.03	0.02
Alpha-linolenic acid	0.04	2*10^−3^
Pentose and glucoronate	0.06	n.s.
Pentose phosphate	0.07	n.s.
Fructose and mannose	0.08	n.s.
Cyanoamino acid	n.s.	0.06
Glycerophospholipids	n.s.	0.06
Flavonoids	n.s.	0.06

Analysis was performed on the list of genes differentially expressed at a significance level of p<0.1 comparing symptomatic to asymptomatic fruits within tree and between different trees “N.s.” means not significant.

### Photosynthesis and carbohydrate metabolism

**Figure 3:**
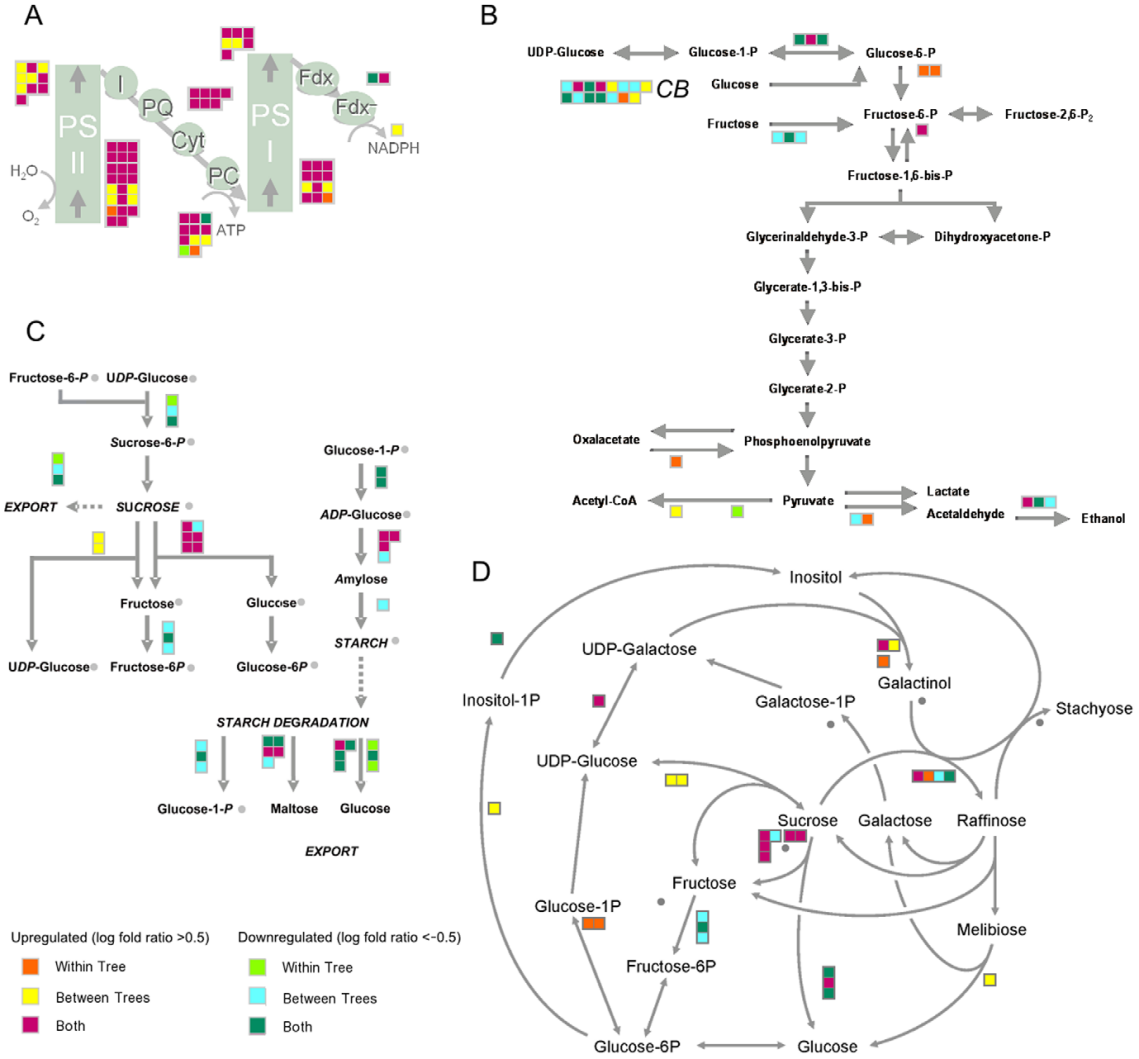
MapMan views of differentially regulated primary metabolism genes. Symptomatic fruit was compared to asymptomatic fruit within the same tree (AS), different tree (AH) and both trees. A) Light reactions of photosynthesis. B) Glycolysis. “CB” block shows gene expression in cytosolic branch genes of small carbohydrate metabolism. C) Sucrose and starch metabolism D) Raffinose metabolism. Colored data points indicate up or down expression with log fold ratios >0.5 and <−0.5, respectively.

The expression of several genes involved in photosynthesis and carbohydrate metabolism increased significantly in symptomatic fruits, when compared to asymptomatic fruit from the same tree or different trees. Transcripts for oxygen-evolving enhancer 3 (PsbQ), photosystem II subunit Q-2, photosystem II reaction center protein J, and other genes encoding different subunits of photosystem II were highly abundant in symptomatic fruit. In addition, genes encoding subunits of cytochrome b6/f (complex subunit 8, subunit IV) and genes encoding ATP synthase subunits (ATPase subunit III, alpha, beta, F, and complex CF0) were induced (Figure 3A). Several transcripts encoding subunits of photosystem I increased, including chlorophyll A apoprotein subunit G, photosystem I reaction center subunit (PSI-N), and D1 subunit.

Several genes for enzymes involved in the first steps of glycolysis were differentially expressed. Up-regulation was observed for genes encoding protein serine/threonine kinase and fructose-2,6-biphosphatase (Figure 3B). There were significant changes in transcripts related to carbohydrate metabolism in symptomatic and asymptomatic fruit. The citrus orthologs of two *Arabidopsis* genes encoding different isoforms of invertase (AB276108 and EY662586) were induced in symptomatic fruit. In starch metabolism, transcription of glucose-1-phosphate adenylyltransferase was diminished, while several genes involved in starch degradation were significantly differentially expressed (Figure 3C). Expression of genes involved in raffinose synthesis were differentially regulated, while transcripts involved in galactinol metabolism were abundant in symptomatic fruits (Figure 3D).

### Hormone-related pathways

**Figure 4:**
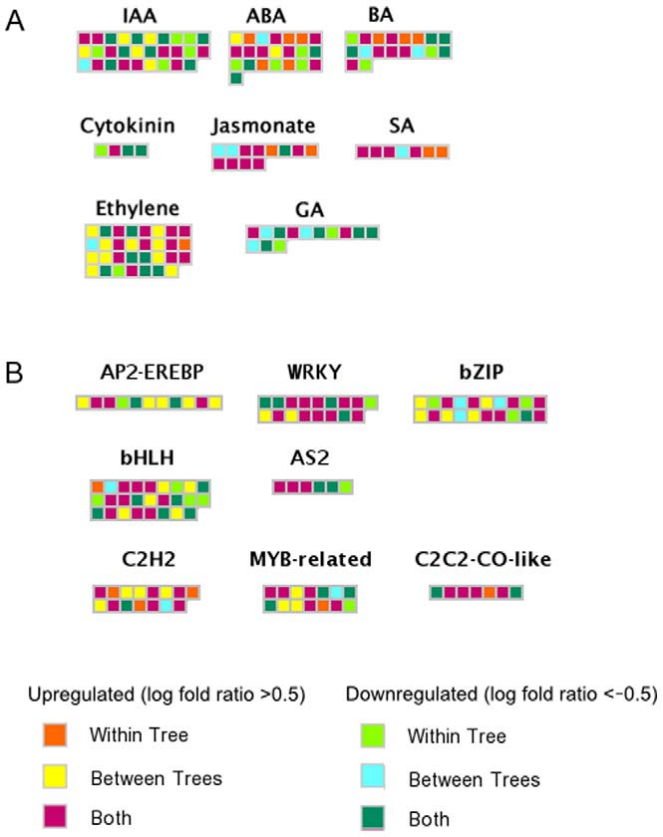
HLB-altered expression of hormone and transcription factors. Differential expression of (A) hormone-related transcripts and (B) transcription factors. Color scale indicates the comparisons between symptomatic asymptomatic and apparently healthy fruit, same as used in Figure 3.

Significant transcriptional changes in response to CaLas infection were observed for a group of genes involved in hormone biosynthesis, mobilization, and signal transduction. Log fold ratios for differentially expressed genes in symptomatic fruits compared to apparently healthy and asymptomatic fruits are shown (Figure 4). Transcripts related to GH3-like proteins involved in auxin synthesis (IAA-amino acid conjugate hydrolase and GH3.1 and GH3.4) were more abundant in symptomatic fruit. Transcripts for GRAM-domain containing protein, involved in the abscisic acid pathway, were more abundant. Several genes involved in ethylene biosynthesis and signal transduction were upregulated in HLB-affected fruit including ACO4, ethylene receptor 1 (ETR1), ethylene response element binding factors (ERF1 and ERF2), ethylene forming enzyme (EFE), while ACO1 and an ethylene-responsive protein were downregulated. Interestingly, genes for ATHK1 and Snakin-1, involved in the cytokinin and gibberellin pathways, were repressed in infected fruit (Figure 4A).

### Transcription factors

Several transcription factors were differentially expressed in HLB-infected fruit, including those belonging to the AP2-EREBP, helix-loop-helix, bZIP, C2H2 zinc finger, C2C2-CO-like, MYB-related, WRKY, and AS2 gene families (Figure 4B). Among genes for bZIP, haloacid dehalogenase-like hydrolase, GBox binding factor 3 and 6 and GBF1 were upregulated at asymptomatic and symptomatic stage, while elongated hyocotyl 5 was downregulated. Some, but not all, bHLH proteins were upregulated in HLB disease. Two AS2 transcription factors (lateral organ boundaries gene family), LBD4 and LBD40, were upregulated while LBD41, LBD21, and LOB were downregulated. Interstingly most of the C2H2 transcription factors were upregulated in response to HLB as were C2C2-CO-like proteins (B-Box types). Several WRKY genes, known to be involved in biotic stress responses, showed increased expression as the disease progressed, including WRKY54, WRKY65, WRKY70, WRKY53, WRKY54, WRKY18, and WRKY50. Fewer WRKY transcripts were less in abundant: WRKY32, WRKY45, and WRKY47.

### Volatiles and defense response

**Figure 5:**
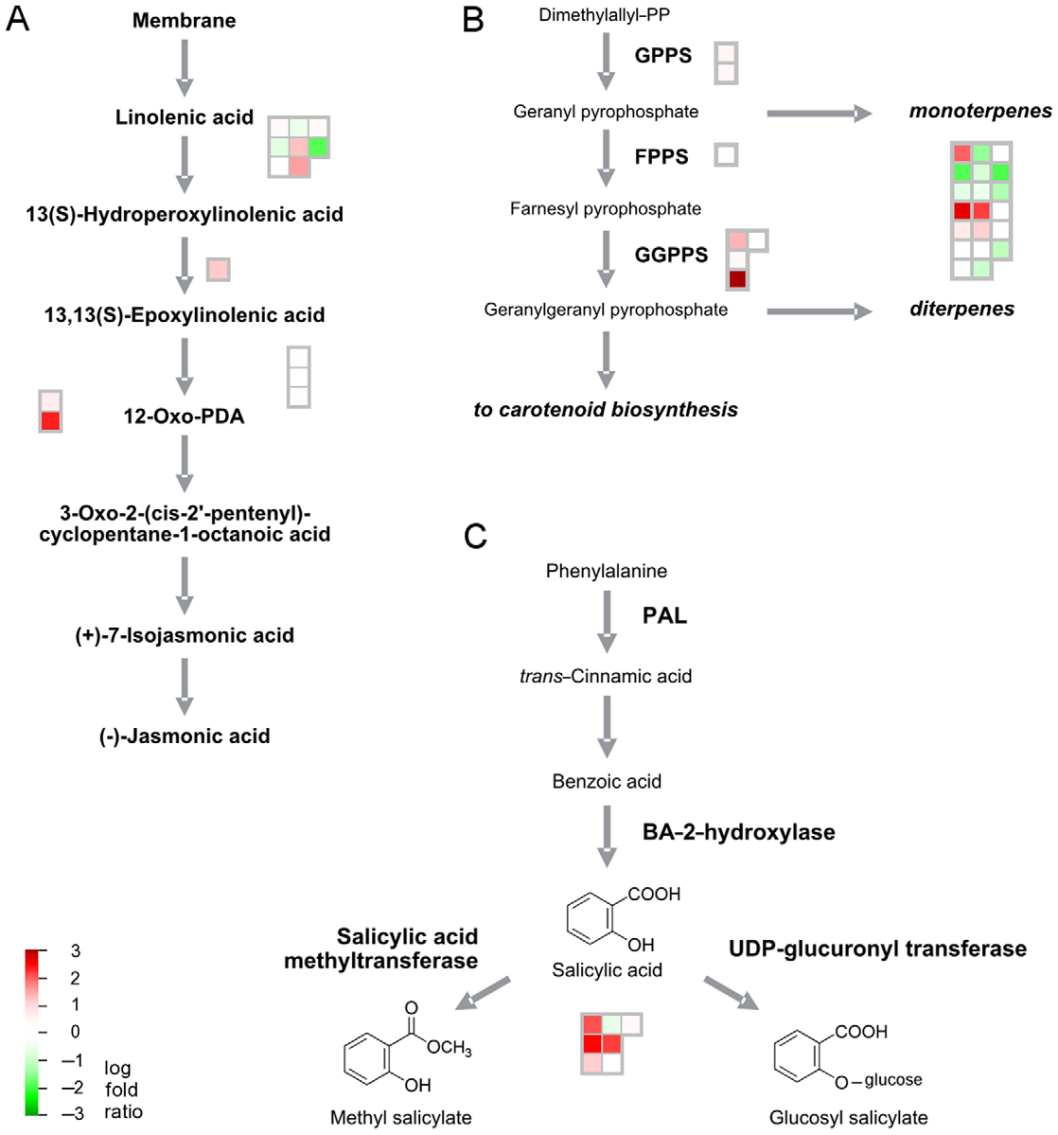
HLB-altered expression in symptomatic fruit in volatile and defense response pathways. Log fold ratios are indicated by a gradient from red (up-regulated) to green (down-regulated). Comparison was made between symptomatic and apparently healthy fruits from the infected orchard. A) Jasmonate biosynthesis, B) Terpenoid and carotenoid biosynthesis, C) Salicylic acid pathway.

Up-regulation of genes involved in jasmonic acid (JA) biosynthesis such as allene oxide synthase, 12-oxophytodienoate reductase, and jasmonic acid-carboxylmethyltransferase was observed in infected fruit, showing that defense responses were induced (Figure 5A). It is interesting to note that while Lipoxygenase2 (EY727780) expression was down, expression increased for Lipoxygenase1/3 (EY663264). Transcriptional changes were also observed in the non-mevalonate or 1-deoxy-D-xylulose/2-C-methyl-D-erythritol-4-phosphate (DOXP/MEP) pathway for isoprenoid biosynthesis located in the plastids. Transcript abundance of 4-hydroxy-3-methylbut-2-en-1-yl diphosphate reductase (ISPH) and the geranylgeranylpyrophosphate synthase 1 genes involved in carotenoid synthesis was elevated in CaLas-infected fruit. Additionally, genes encoding several types of terpene synthases involved in mono- and diterpene biosynthesis, which affect aroma composition and nutritional properties of citrus fruit, were differentially expressed in HLB-infected fruit (Figure 5B). The salicylic acid-mediated response was also activated as shown by the higher abundance of transcripts for genes encoding salicylic acid methyltransferases (Figure 5C).

### Protein degradation

**Figure 6:**
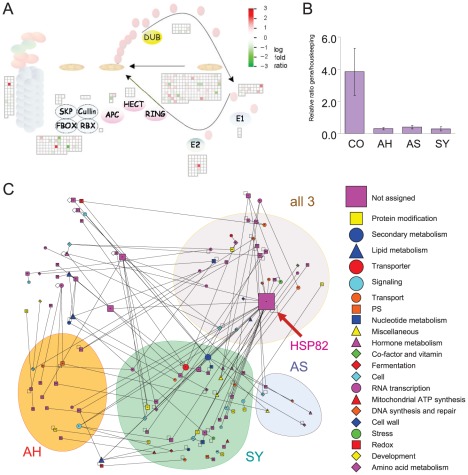
HLB-altered expression related to protein degradation and misfolding. A) Differentially expressed genes involved in ubiquitin-dependent degradation, showing comparison between symptomatic and apparently healthy fruits. B) Pattern of expression of heat shock protein 82 (HSP82) in HLB-free location (CO), apparently healthy (AH), asymptomatic (AS), and symptomatic (SY) fruits. C) Protein-protein interaction network predicted in *Citrus* based on the *Arabidopsis* knowledgebase. Networks between proteins encoded by HLB-regulated genes were divided into four different clusters. Node legend indicates functional classes of transcripts.

Differentially expressed genes involved in protein degradation were identified at the symptomatic stage (Figure 6A). Upregulated genes included the ubiquitin-ring family of proteins: zinc finger C3HC4-type ring finger, zinc ion binding ring-H2 protein, F-box family protein, UBQ10, and UBQ3, while transcripts for other C3HC4 ring finger proteins were downregulated. At the same time, transcripts for heat shock protein 82 were downregulated at both asymptomatic and symptomatic stages (Figure 6B). This gene is a major hub in the protein-protein interaction network deduced from the *Arabidopsis* protein-protein knowledgebase (Figure 6C; Table S1).

### Real time PCR validation

**Figure 7:**
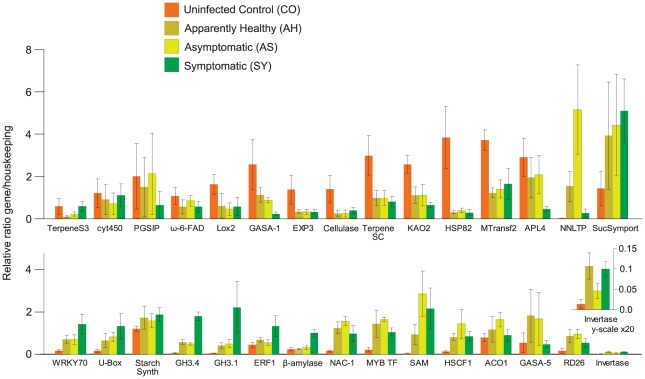
qRT-PCR analysis. Pattern of expression of genes belonging to different pathways in the four categories of fruits: symptomatic (SY), asymptomatic (AS), apparently healthy (AH) and healthy from HLB-free location (CO). Gene names correspond to those listed in Table 2. Bar heights for expression of Invertase also shown at 20-fold magnification (lower-right inset).

Real-time PCR (RT-PCR) analyses were conducted using all four fruit types to validate the expression patterns of a subset of differentially expressed genes identified by next generation sequencing (Figure 7; Table 2). Among hormone-related transcripts, indole-3-acetic acid amido synthetase (GH3.4) was expressed more at all disease stages. Ethylene responsive factor 1 (ERF-1) expression was higher at the symptomatic stage. Transcripts for ent-kaurenoic acid hydroxylase 2 (KAO2) and gibberellin-responsive protein GASA1 were less abundant in infected fruits. The jasmonic acid and salicylic acid pathways were affected by HLB disease. Transcript abundance of lipoxigenase2 (Lox2), which is involved in jasmonate biosynthesis, was lower in fruit from infected trees at asymptomatic and symptomatic stages than in healthy trees from the disease-free location. Salicylic acid methyltransferase (SAM) was strongly up-regulated in infected fruits. Transcription factors are key players in transducing signals generated in response to pathogen infection. Among them, the WRKY family was highly involved in HLB response. WRKY70 was highly up-regulated in symptomatic fruits and its transcript abundance was also significantly higher in asymptomatic, apparently healthy fruit. Transcripts of RD26, NAC-1, and Myb-related transcription factors (MYB TF) were more abundant at asymptomatic stages. Heat shock protein 82 (HSP82), the hub with the highest degree in the protein-protein interaction network, was down-regulated in all infected fruits, with likely consequences for overall fruit metabolism.

**Table 2:** List of genes analyzed by quantitative RT-PCR, including P-values for each of three pairwise comparisons from ANOVA of the expression data.

*Accession*	*Name*	*P-value*		
		*AH vs. CO*	*AS vs. CO*	*SY vs. CO*
EY685091	Acidic cellulase	0.038	0.039	0.056
AF321533	ACC oxidase (ACO-1)	0.352	0.02	0.615
EU861194	APL4	0.276	0.323	0.009
EY710632	β-Amylase	0.856	0.276	0.003
EY710254	Cytochrome P450 (Cyt450)	0.632	0.368	0.841
EY660393	ERF-1	0.06	0.419	0.037
EY747590	Expansin 3 (Exp3)	0.059	0.055	0.054
CK935746	GASA-1	0.110	0.068	0.026
EY721422	GASA-5	0.171	0.219	0.795
EY693577	GH3.1	0.009	0.017	0.037
CF837667	GH3.4	0.001	0.0003	0.0001
EY745561	Heat Stress TF C-1 (HSCF1)	0.002	0.031	0.005
DY306049	HSP82	0.014	0.015	0.014
AB276107	Invertase	0.009	0.062	0.003
CV714426	Ent-kaurenoic acid oxidase (KAO2)	0.012	0.019	0.002
EY677115	Lipoxgenase2 (Lox2)	0.081	0.023	0.048
EY719680	Methyltransferase2 (MTransf2)	0.001	0.003	0.015
EY719020	Myb related TF (MYB TF)	0.031	0.00007	0.003
EY670400	NAC-1	0.002	0.0004	0.019
EY746283	NNLTP	0.021	0.014	0.087
EY683840	Omega-6-FAD	0.157	0.454	0.136
CX078423	Glycogenin-2/PGSIP	0.691	0.936	0.231
DY306001	RD26	0.024	0.006	0.054
EY747349	SA-methyltransferase (SAM)	0.029	0.01	0.02
EY710657	Starch synthase	0.178	0.137	0.03
AY098894	Sucrose symporter (SucSymport)	0.178	0.11	0.021
EY709847	Terpene synthase 3 (TerpeneS3)	0.089	0.171	0.997
CV886175	Terpene synth. cyclase (TerpeneSC)	0.028	0.027	0.019
EY655547	U-Box	0.067	0.008	0.029
EY675876	WRKY70	0.008	0.019	0.009

Names of genes (or abbreviated gene names in parentheses) correspond to bar clusters in Figure 7. Symptomatic (SY), asymptomatic (AS), apparently healthy (AH), healthy control (CO). P-values<0.05 are considered significant.

Glucose-1-phosphate adenylyltransferase (APL4) was highly down-regulated in symptomatic fruits while invertase (β-fructofuranosidase) was clearly up-regulated in the peel of apparently healthy and symptomatic fruits from the infected orchard. Sucrose symporter (SucSymport) was up-regulated in infected, symptomatic fruits.

The terpenoid pathway was affected by HLB disease, as shown by the down-regulation of terpene synthase cyclase (TerpeneSC). Induction of lipid transfer protein (NNLTP) was observed in the absence of symptoms. The expression of other genes such as acidic cellulase and methyltransferase2 (MTransf2) was diminished in infected fruits.

## Discussion

Huanglongbing, a highly destructive disease of citrus, threatens citrus-producing areas worldwide [1]. Previous studies monitored transcriptional changes in leaves on a large scale using microarrays to investigate host responses and reveal the mechanisms underlying disease development [6], [7]. The present work focuses on transcriptional regulation in fruit peel to analyze the response to CaLas infection at different disease stages. Next-generation sequencing technology (NGS), as used here, can find differential expression of an increased number of transcripts, many of which may not be present in EST databases or represented in microarrays. NGS data can be used for specific transcriptome assemblies that become resource datasets for annotation of genomes and annotation of differentially regulated genes and proteins analyzed using any “omic” technique. However, the absence of a completed genome sequence limited the advantages of RNA-Seq technologies.

The aim of this work was to provide data using NGS technology for a comprehensive analysis of metabolic changes in fruit induced by HLB disease. These findings will uncover the fruit disorder mechanisms and facilitate development of short-term therapeutic strategies for already-infected trees. Toward this end, the experimental design included four types of fruit: healthy control fruit from an HLB-free orchard, apparently healthy fruit from non-symptomatic trees in an orchard affected by HLB, and asymptomatic and symptomatic fruit from infected symptomatic trees in the affected orchard. This design made it possible to identify differentially expressed genes at different disease stages. A comparison between apparently healthy and asymptomatic fruit revealed genes induced early in disease development. Comparing asymptomatic and symptomatic fruit identified genes involved in the host response during disease progression. Comparing symptomatic and apparently healthy fruit revealed host response genes related to the presence of HLB symptoms. Healthy fruit from the HLB-free location were also compared to the three sample types from the infected location. Differences in gene expression in these comparisons are likely to result from environmental and agronomic variability due to the difference in location, in addition to the effects of HLB.

A range of 1154 to 1762 differentially regulated genes (log fold ratio <−1.5 and >1.5) were found using RNA-Seq comparing the three categories of fruits from the infected orchard with those taken from a location free of HLB (Tables S1, S2, S3). In qRT-PCR analysis, 31 of 33 differentially regulated genes confirmed the pattern of expression found by RNA-Seq. Comparisons among the three fruit categories in the infected orchard resulted in fewer differentially regulated genes (301–780; Tables S4, S5, S6). This was expected, since samples within the same orchard grew under similar environmental and agronomic conditions.

**Figure 8:**
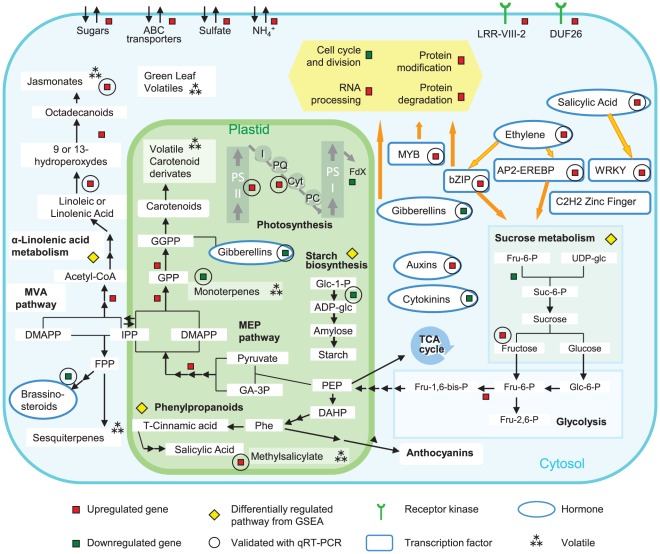
Fruit metabolism and regulatory pathways in CaLas-infected fruits. Genes, pathways, and cell functions that were differentially expressed are indicated with a square (red for up-regulated, green for down-regulated). Significantly differentially regulated pathways in gene set enrichment analysis are indicated in yellow.

A similar contrast between samples from the infected and uninfected orchards is seen in principal component analysis. The transcripts that most strongly characterize the asymptomatic and apparently healthy samples (MF-AS, MF-AH) are common between the two samples. The symptomatic (MF-SY) and uninfected orchard samples (MF-CO) are both distinct groups and widely separated in the biplot (Figure S2). A visual summary outlines the most important transcriptional changes in the networks among genes, pathways, and cell functions in the fruit peel (Figure 8). Gene set enrichment analysis identified several pathways significantly affected by HLB as symptoms appear, considering both within-tree and between-tree comparisons, such as those for phenylpropanoids, starch and sucrose metabolism, carbon fixation, ascorbate, and alpha-linolenic acid (Figure 1, Figure S3).

Other pathways were also affected by HLB in the two comparisons within the same orchard. Transcripts encoding different subunits of the photosystem II reaction center and cytochrome b6-f complex subunit were more abundant in symptomatic HLB-infected fruit than in healthy fruit. Photosynthesis is central to all aspects of plant biology, since it provides energy for growth and reproduction, but its regulation by biotic and abiotic stresses is still unclear. The induction of photosynthetic light reactions in the fruit is consistent with the observation that symptomatic HLB-infected fruit often remains green. The retention of green color and increase of photosynthesis reactions is probably linked to the lower amount of ethylene detected in symptomatic fruits [28]. Photosynthesis is usually downregulated by pathogen attacks. The upregulation of photosynthetic reactions in the fruit does not contradict this common consideration because CaLas infections typically occur in young leaves. The transcriptomic changes observed in the fruit are probably linked to the source-sink disruption caused by leaf infections.

Protein degradation and modification pathways were significantly changed by CaLas infection, as shown by the up-regulation of genes such as C3HC4-type ring finger proteins involved in ubiquitin-mediated degradation. Interestingly, heat shock proteins HSP82 and HSP70, highly interactive proteins in the PPI network inferred in citrus, were down-regulated at different stages of the disease. Heat shock proteins are highly conserved proteins induced in cells subjected to elevated temperatures or other environmental stresses [29], [30]. These proteins act as molecular chaperones to stabilize, reduce misfolding, or facilitate refolding of proteins that have been denatured during stress events. In plant cells, HSP70 and HSP90 are involved in signal transduction leading to plant defense responses. Both proteins interact with a salicylic acid-induced protein kinase and their silencing affected the hypersensitive response in *Nicotiana benthamiana* while inducing non-host resistance to *Phytophthora infestans* [31]. HSP90 also modulates the innate immune responses involving gene-for-gene specific interactions, acting as a scaffold protein in a complex that mediates signal transduction [32]. Based on these findings, we speculate that down-regulation of heat shock proteins observed at different stages of HLB disease might increase protein misfolding in the fruit peel.

Genes encoding ATP synthase gamma and delta chains were also induced in symptomatic fruit, supporting the idea that CaLas may act as an energy parasite by scavenging ATP from its host with a pathogen-specific ATP/ADP translocase. Indeed, the recently sequenced genome of CaLas revealed the presence of an ATP/ADP translocase in addition to ATP synthase [33]. ATP scavenging may be a possible mechanism of pathogenicity, affecting the fruit peduncle, columella, and seed coat.

Photosynthesis regulation in infected fruit peel may also reduce transport of sugars and ions such as nitrate, sulfate, and potassium. In this study, several differentially expressed genes were associated with transport of ions, including ammonium, sulfate, and phosphate (Table S1). Inorganic ions can modify sugar metabolism and photosynthesis [34], [35]. Polarized vesicle trafficking, transport, and secretion of plant materials are associated primarily with non-specific resistance during host-pathogen interactions. ABC transporters play important roles in this process, since they are also involved in virulence, host range, and symptom elicitation [36].

Citrus proteins related to sugar and PDR/ABC transporters implicated in secretion of antimicrobial terpenoids [36] were specifically induced by HLB at the symptomatic stage (Table S1). Interestingly, among the genes identified in the CaLas genome were those for phosphate and zinc uptake into the cell [33]. This implies that mineral uptake by the pathogen may be enhanced due to induction of endogenous genes as well as host genes. However, phloem necrosis induced by CaLas contributes to the impaired nutritional transport functions and source-sink communication observed in this study.

Starch accumulation in leaf chloroplasts of sweet orange trees infected with CaLas has previously been demonstrated and is characteristic for HLB [1]. Genes encoding the large subunit of ADP-glucose pyrophosphorylase, the key enzyme catalyzing the first and limiting step in starch biosynthesis, were up-regulated in infected leaves [6], [37]. Interestingly, a gene encoding the large subunit of glucose-1-phosphate adenylyltransferase was down-regulated in infected and symptomatic fruit in the present study.

**Figure 9:**
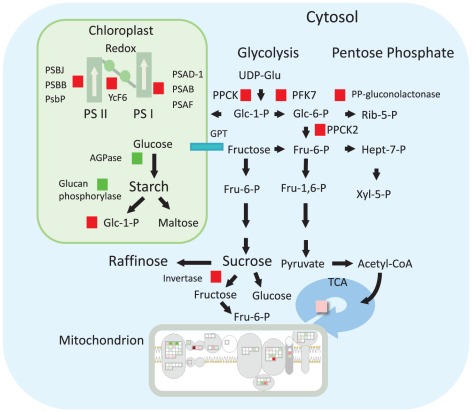
HLB-regulation of primary metabolism in symptomatic fruits. Differentially regulated genes and pathways involved in primary metabolism are indicated with a square (red for up-regulated, green for down-regulated).

Also of interest are contrasting patterns of expression for different isoforms of starch synthase and starch cleavage genes, leaving the dynamics of starch accumulation in the fruit unclear (Figure 9). This is in contrast to experimental observation of starch accumulation in infected leaf tissues. In leaves, this accumulation was linked to up-regulation of genes involved in starch biosynthesis and down-regulation of its conversion to maltose (DPE2 and MEX1) [37]. It has been hypothesized that starch accumulation is an effect of phloem plugging/necrosis during HLB infection, although it usually occurs before these symptoms are visible [38]. Differences in starch accumulation are probably linked to the different types of organs: mature leaves are “source” and fruit are “sink”. However, further analysis of fruit starch accumulation must be performed to validate this hypothesis.

An increased abundance of transcripts for genes involved in the first steps of glycolysis and sucrose metabolism was observed in fruits from infected trees. In the cytosol, an invertase gene was up-regulated in asymptomatic and symptomatic fruit, affecting the sugar balance and communication between sink and source tissues. We speculate that this leads to increased glucose and fructose and decreased sucrose in fruit cells, which should be further validated with carbohydrate analysis. It is important to note that elevated glucose and fructose has been demonstrated in citrus leaves infected with CaLas [39]. Interestingly, different types of invertases were up-regulated in leaves (cell wall) [37] and fruits (vacuolar). Over-expression of yeast invertase in the cell wall of transgenic tobacco (*Nicotiana tabacum*) disrupted sucrose export, allowing soluble sugars and starch to accumulate, which consequently inhibited photosynthesis and resulted in stunted growth and bleached or necrotic leaf areas [40].

As previously suggested for leaves [38], it is possible that the differential expression of key genes involved in sucrose and starch metabolism, as observed in CaLas-infected citrus fruit, might affect the osmotic potential and induce plasmolysis, thus altering the ripening process and producing typical HLB symptoms. However, further analysis of sugar concentrations will be necessary to clarify the causes and effects of disease symptoms in the fruit. Other studies on CaLas-infected leaves have shown increased sucrose and glucose, but not fructose [37]. In fruit, it is possible that altered fructose and glucose concentrations might be responsible for physiological disorders and affect source-sink relationships with leaves.

The gene set enrichment analysis confirmed that sucrose and starch metabolism were highly affected by the disease. Integrated analysis of leaf and fruit data indicates that sugar and starch metabolism play a key role in the metabolic dysfunction induced by HLB disease. However, few studies have been conducted to address the effects of altering sugar metabolism on resistance to pathogen infections. Invertase plays a key role in the activation of stress responses and may function as an extracellular indicator for pathogen infection [41], [42]. Indeed, transgenic plants overexpressing sugar metabolism enzymes such as a heterologous invertase from yeast have helped clarify source-sink relationships [4]. The expression of different viral movement proteins in transgenic plants and the resulting effects on photosynthesis, carbohydrate accumulation, and partitioning emphasize the importance of sugars in activating defense responses against biotic attacks [43], [44], [45].

Sink metabolism may be essential to satisfy the energy requirements of activating the cascade of defense responses. Interestingly, sucrose synthase was also more abundant in symptomatic fruit while sucrose transporter genes were downregulated by HLB. The Genevestigator database [46], indicated that these proteins may be down-regulated by hormones such as ethylene, methyl-jasmonates, and indol-3-acetic acid. The concept of sucrose metabolism regulators as a potential target for HLB therapeutics is intriguing.

Increasing evidence indicates an extensive cross-talk between sugar, hormone, and light signal transduction networks in plants [47]. Hormone pathways were significantly altered in fruit peel in response to CaLas infection. Two genes involved in auxin synthesis, GH3.1 and GH3.4, were induced in affected fruit. Gibberellin and cytokinin-related genes were mostly down-regulated in symptomatic fruits. Gibberellin regulation has been observed in other fruit disorders such as albedo breakdown disorder (unpublished data) and applications of GA_3_ before fruit color break can reduce the occurrence of some fruit disorders. It is possible that sugar metabolism changes observed in the fruit might be linked with the down-regulation of gibberellins that regulate energy and carbohydrate metabolism. Previous studies have demonstrated that regulation of gibberellic acid-induced gene expression is affected by sugar and hormone signaling [48]. That cytokinins play a role in sugar regulation has been demonstrated [49]. Therapeutic approaches using small-molecule hormones such as cytokinins and gibberellins may allow modification of fruit metabolism to mitigate the negative impact of HLB on fruit quality and productivity.

Ethylene regulates a variety of developmental processes and stress responses in plants, including seed germination, cell elongation, senescence, fruit ripening, and defense. Nonetheless, ethylene can promote either disease resistance or susceptibility, depending on the host–pathogen interaction [50], [51]. In our study, considerable changes were observed in the transcriptional profiles of genes related to ethylene biosynthesis and signal transduction. ACC synthase and ACC oxidase play pivotal roles in ethylene biosynthesis and their expression is often affected by pathogen attack [50]. It was unclear how ethylene concentration changes in fruit in response to HLB. Different isoforms of ACC synthase and ACC oxidase were up- or down-regulated. In addition, several transcripts involved in ethylene signaling and response were more abundant in symptomatic fruits, suggesting that HLB may have a stronger effect on ethylene signaling than ethylene biosynthesis. The general up-regulation of ethylene-related genes does not agree with the lower ethylene concentration previously reported in HLB symptomatic fruits [28]. This may be due to differences in fruit developmental stages and health status. Furthermore, gene networks responsible for ethylene biosynthesis and perception are still not fully elucidated. A large number of genes annotated as ethylene-related occur as parologs playing tissue-specific roles, and many play additional roles in biotic stress response. Their expression may be drastically affected by the complex gene regulatory network involved in immune responses without directly affecting ethylene levels. The increased number of ERF- and AP2/EREBP-related genes modulated by HLB supports this notion. These factors control expression of many PR proteins and defense response effectors [50]. In addition, the induction of ethylene biosynthesis and signal transduction could profoundly modify fruit metabolism by accelerating senescence linked to the typical fruit malformations caused by HLB.

Salicylic acid and jasmonates are hormones involved in activating defense responses to pests and pathogens. Pathways for both hormones were activated in CaLas-infected citrus. It is known that different hormone-regulated defense pathways are activated depending on whether the pathogen is a necrotroph or biotroph [4]. The mechanisms of CaLas pathogenesis are poorly understood. The putative bacterial pathogen is closely related to bacterial families with symbiotic properties (i.e., *Rhizobiaceae*) [1]. However, the gene expression in challenged fruit showed an up-regulation of jasmonate-induced defense responses, typical of a host response to localized necrotroph invasion. This pathway is also stimulated by long distance signaling involving volatile compounds. Salicylic acid methyl-transferase was up-regulated in early, asymptomatic disease stages, potentially leading to production of volatile methylsalicylate. Nonexpressor of pathogenesis-related genes1 (NPR1) was up-regulated in symptomatic fruits (Table S1). In *Arabidopsis,* NPR1 is required for SA-mediated suppression of JA-dependent defenses [52]. Also, ethylene modulates the NPR1 dependency of SA-JA antagonism, compensating for enhanced allocation of NPR1 to functions in SA-dependent activation of PR genes [53]. In plants, IRE1 (inositol-requiring 1) gene is considered to be involved in unfolded protein response (UPR) mediated by SAR with the engagement of BiP [54]. HLB upregulation was observed for IRE1a (EY679744), a gene closely involved in the UPR in plants [55]. This gene expression change suggests that UPR may be activated in the infected fruit.

Plants employ a network of intertwined mechanisms to counter infection by pathogens and parasites. One line of defense is based on dominant disease resistance (R) genes encoding nucleotide-binding, leucine-rich repeat (NB-LRR) proteins that mediate resistance to pathogens possessing corresponding avirulence (Avr) genes [56]. Several leucine-rich repeat (LRR) receptor kinases were up-regulated in symptomatic fruit, implying that they may be receptors triggering a futile defense response against CaLas.

Innate immunity can be regulated by transcription factors. Several genes belonging to the WRKY family of transcription factors such as WRKY70, WRKY13, WRKY30, WRKY40, WRKY65, and WRKY31 were up-regulated at both asymptomatic and symptomatic stages of HLB. WRKY70 acts as a convergence point, determining the balance between SA- and JA-dependent defense pathways in addition to being required for R gene-mediated resistance. Its role in JA and SA signaling, however, has recently been questioned [57]. Similarly, AtWRKY53 positively modulates SAR. Members of the WRKY family were implicated in regulating the transcriptional reprogramming associated with plant immune responses and they may act as positive and negative regulators of disease resistance [58]. Other genes encoding bZIP and C2H2 zinc finger transcription factors were up-regulated in CaLas-infected fruits. The regulation of bZIPs in HLB-affected citrus may be associated with reported modifications observed in energy metabolism [59].

In the cytosol, up-regulation of 3-hydroxy-3-methyl-glutaryl CoA reductase (HMG1), 12-oxophytodienoate reductase 2, and allene oxide synthase may induce an increase in jasmonate-derived volatiles. Several genes involved in terpenoid metabolism, such as terpene synthase 3 and terpene synthase cyclase, were down-regulated in symptomatic fruits. These enzymes are involved in the synthesis and transport of a variety of terpenes, gibberellins, brassinosteroids, alkaloids, and plant volatiles that play diverse roles in plant development and defense [60]. The regulation of genes involved in these pathways has important implications for volatile emissions and is associated with a variety of responses. Further studies on the volatile emission profiles of CaLas-infected fruits will clarify the role of these enzymes.

Next generation sequencing enabled us to identify genes differentially expressed in citrus fruit peel in response to CaLas infection, leading to a better understanding of the processes involved in HLB disease development. This study identified several genes that were differentially expressed at the asymptomatic stage that may aid disease detection at primary stages of infection, before the pathogen can be detected by PCR. However, their usefulness as HLB-specific induced genes cannot be determined until a similar expression analysis is conducted on the same tissues infected by other citrus pathogens such as Citrus tristeza virus, *Xylella fastidiosa*, and *Xanthomonas axonopodis*. In the fruit peel, HLB induced transcriptional changes in important pathways such as sucrose and starch metabolism, hormone signaling, and isoprenoid synthesis. WRKY transcription factors appear to help regulate defense responses to CaLas in the fruit. The induction of genes involved in the light reactions of photosynthesis might increase the occurrence of reactive oxygen species, leading to protein degradation and misfolding. The application of small-molecule hormones is another promising short-term strategy to mitigate the devastating negative physiological effects of HLB.

## Materials and Methods

### Plant material and experimental design

**Figure 10:**
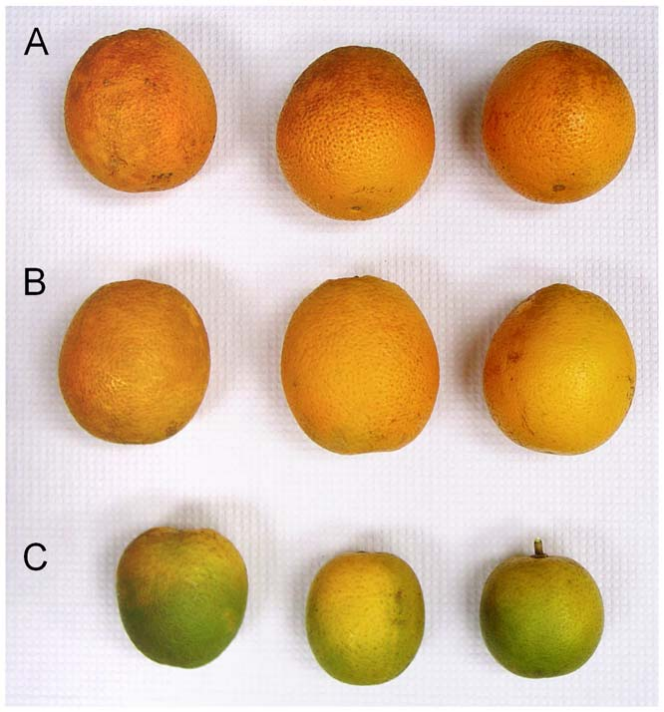
Stages of infection in the *Citrus* fruits studied. (A) Fruits of apparently healthy plants; (B) Asymtomatic fruits of infected plants; (C) Symtomatic fruits of infected plants.

Four types of mature fruit peel were analyzed based on origin, phenotype, and presence of the pathogen (Figure 10). Three categories of fruit were collected from “Valencia” sweet orange (C. sinensis L. Osb.) trees located at the USHRL-USDA Farm in Fort Pierce (St. Lucie County, FL). Trees were analyzed by PCR for the presence of CaLas using leaf petioles from four to six leaves collected from different areas in the canopy. All trees at this location were found to be PCR-positive at the time of collection. The first two categories of fruit peel were collected from trees with typical HLB disease symptoms on leaves (blotchy mottle and chlorosis) and fruit (small, green, and irregular in shape). Fruit peel was collected from asymptomatic and symptomatic fruit of the same tree. The third category was fruit peel from apparently healthy trees at the same location. These trees were HLB-positive using leaf petioles at the time of sampling. The fourth category was healthy fruit from ‘Valencia’ PCR-negative trees at a disease-free location, the Citrus Research and Education Center (Lake Alfred, FL). Fruit peduncles, stored at −20°C, from all collected fruits were analyzed by PCR for the presence of CaLas. Peduncles from apparently healthy trees were PCR-negative while those from all other fruits in the infected orchard were PCR-positive, except for one asymptomatic sample. Peduncles of healthy fruits from disease-free location were PCR-negative. Five to ten fruit were collected from each of five different trees per treatment group, representing five biological replicates. Fruit peel segments were cut and mixed, immediately frozen in liquid nitrogen, and stored at −80°C. Juice sacs were removed before extraction.

### PCR detection of *Ca*. L. asiaticus

Petioles and peduncles were ground in liquid nitrogen with a mortar and pestle and 100 mg ground tissue was used for DNA extraction. DNA was extracted using the Plant DNeasy® Mini Kit (Qiagen, Valencia, CA) according to manufacturer's instructions, yielding 20 to 30 ng DNA per extraction. Real-time PCR assays were performed using primers HLBas (5′- TCGAGCGCGTATGCAATACG -3′) and HLBr (5′- GCGTTATCCCGTAGAAAAAGGTAG -3′) and probe HLBp (5′- AGACGGGTGAGTAACGCG -3′) [61]. Amplifications were performed using an ABI 7500 real-time PCR system (Applied Biosystems, Foster City, CA) and the QuantiTect Probe PCR Kit (Qiagen) according to manufacturer's instructions. All reactions were carried out in duplicate in a 20 µL reaction volume using 5 µL DNA per reaction. Plants or fruits were considered PCR-positive when C_T_ (cycle threshold) values were below 32.

### RNA extraction

One gram of peel tissue was ground in liquid nitrogen with a mortar and pestle and resuspended in 10 mL guanidinium isothiocyanate buffer [62]. Total RNA was extracted according to Strommer et al. [63] with slight modifications. Phenol/chloroform/isoamylalcohol (25∶24∶1) extraction was followed by two extractions with chloroform/isoamylalcohol and precipitation of RNA with isopropanol at −20°C overnight. RNA was pelleted by centrifugation at 10,000 *g* and 4°C for 1 h, resuspended in 5 mL water and precipitated overnight at 0°C with an equal volume of 8 M LiCl. After centrifugation at 10,000 *g* and 4°C for 1 h, RNA was washed twice with 70% ethanol, air-dried and dissolved in 500 µL of water. RNA was further purified using the RNeasy® MinElute Cleanup kit (Qiagen, Valencia, CA, USA) according to the manufacturer's instructions. RNA quality and purity were assessed using an Agilent Bionalyzer (Folsom, CA).

### cDNA library construction and high throughput sequencing

RNA from the five biological replicates was equally pooled to 10 µg and then used to construct a cDNA library for each of the four fruit types. The cDNA libraries were constructed following the Illumina mRNA-sequencing sample preparation protocol (Illumina Inc., San Diego, CA). Final elution was performed with 16 µL RNase-free water. The quality of each library was determined using a BioRad Experion (BioRad, Hercules, CA). Each library was run as an independent lane on a Genome Analyzer II (Illumina, San Diego, CA) to obtain read lengths of up to 85 bp per paired end.

### Sequence data processing and analysis

The raw Illumina reads were trimmed to remove low quality reads using custom scripts. Individual reads from each sample were aligned to the *Citrus sinensis* unigene set (15,808 sequences; NCBI Unigene Build #11, 4/20/09) with Burrows-Wheeler Transform (BWA) [64]. Read counts were generated with SAMtools [65] and custom scripts.

Six pairwise comparisons were made between the read counts for the four samples. For each pairwise comparison, the raw count data was normalized to control for different sequencing depths across samples with the DESeq Bioconductor package [66]. DESeq was developed for the statistical analysis of experiments with few or no replicates, accommodating this by treating samples from differing treatments or phenotypes as replicates in the estimation process. The results of this pooling are more conservative than in an experiment with more replicates.

### Principal component analysis

Data analysis was performed to alleviate possible bias caused by the amount of collected material for each class or other confounding factors. A within-sample normalization process was applied to each sample to calculate the ratio of each target gene. This way, the sum of the ratios of all target genes was 1 while each target gene had its own count ratio within (0 to 1). For each sample, all target genes were first sorted in order from high to low. Then the top target genes with their accumulated ratio counting for 25% of the total were retained for further analysis, to make the differentially regulated gene selection more robust and focused on a relatively small number of strong target genes. By integrating all selected target genes for the four classes, we generated a complete list of potential target genes. In this list, some genes are shared among all four classes, while some are only observed in one class. Principal component analysis was then applied to the ratio matrix of this gene list to examine the contribution of each target gene to the separation of the classes. A biplot was constructed based on the first two principal components. The length of the loading vector for each target gene indicates the contribution of that gene to the separation based on the first two principal components. To examine which genes contribute most to each class, two criteria for screening were employed. First, the mean value of the loading vector lengths (strengths) of all potential target genes was calculated and 80% of that value was set as the threshold for strength screening. Target genes with loading vector lengths larger than this threshold were considered strong. Secondly, the directional similarity was calculated between each target gene and each class and then 0.98 (i.e, the cosine of 10°) was set as the threshold value for similarity screening. Larger similarities indicate bigger contributions of a gene to a class.

### Functional categorization and protein-protein network analysis

The *Citrus sinensis* unigene set was annotated using Blast2GO [67] to assign Gene Ontology (GO) terms to each gene. Lists of transcripts that were differentially expressed at a significant level (p<0.01, absolute value of Log Fold Change >1.5) in the pairwise comparisons were used as input for one-tailed Fisher's Exact Test in Blast2GO to identify GO terms that were significantly over-represented.

In addition, the differentially expressed genes were also functionally analyzed using the MapMan knowledgebase blasting to the TAIR database [25]. Gene set analysis was also performed using Pathexpress [26] for the highest *Arabidopsis* hit per *Citrus sinensis* unigene set (considering as a cutoff Log Fold Ratio >1/−1). A protein-protein interaction network (PPI) was deduced for *Citrus* based on PPIs in *Arabidopsis* [68] using blast analysis. Networks were identified and visualized using Graphviz software.

### Real time TaqMan PCR system

Real Time Taqman PCR analysis was conducted to validate RNA-seq data. Three biological replicates (a pool of 5 to 10 fruits from different plants) were used for each of the four types of fruit peel (healthy, apparently healthy, asymptomatic, and symptomatic). For each target gene, PCR primers and a TaqMan® probe were purchased as an assay mix from Applied Biosystems (Foster City, CA). DNase treatment and cDNA synthesis were performed in a combined protocol following instructions of the Quantitect Reverse Transcription Kit (Qiagen). A standard curve to determine the linearity of amplicon quantity vs. initial cDNA quantity was generated for each gene. Amplifications used 25 ng cDNA in a 20 µL final volume with TaqMan Universal PCR Master Mix and Taqman Assay ABI mixes (Applied Biosystems). Amplifications were performed on a StepOne Real Time PCR system (Applied Biosystems) using standard amplification conditions: 1 cycle of 2 min at 50°C, 10 min at 95°C, 40 cycles of 15 s at 95°C, and 60 s at 60°C. All PCR reactions were performed in duplicate. Fluorescent signals were collected during the annealing temperature and C_T_ values extracted with a threshold of 0.04 and baseline values of 3 to 10. *Citrus sinensis* elongation factor 1 alpha (EF-1α, accession AY498567) was used as an endogenous reference and ΔΔ*C*_T_ was calculated by subtracting the average EF-1α *C*_T_ from the average *C*_T_ of the gene of interest.

Real Time Taqman PCR analysis was also conducted for CTV (Citrus Tristeza Virus) detection using the same RNA to determine the presence of the virus in the samples analyzed for HLB response. Primers were designed based on CTV CP reference sequence T36 (M76485) and the same protocol was followed as previously described.

ANOVA was performed considering the three biological replicates for each of the four fruit categories. P-values were determined for each of the six pairwise comparisons and values lower than 0.05 were considered significant.

## Supporting Information

Table S1 **Differentially expressed genes in symptomatic fruit in comparison to control (healthy in disease-free location), annotations and number of protein-protein interactions deduced from Arabidopsis knowledgebase.**
(HTM)

Table S2 **Differentially expressed genes in asymptomatic fruit in comparison to control (healthy in disease-free location), annotations and number of protein-protein interactions deduced from Arabidopsis knowledgebase.**
(HTM)

Table S3 **Differentially expressed genes in apparently healthy fruit in comparison to control (healthy in disease-free location), annotations and number of protein-protein interactions deduced from Arabidopsis knowledgebase.**
(HTM)

Table S4 **Differentially expressed genes in apparently healthy fruits in comparison to asymptomatic, annotations and number of protein-protein interactions deduced from Arabidopsis knowledgebase.**
(HTM)

Table S5 **Differentially expressed genes in symptomatic fruits in comparison to asymptomatic, annotations and number of protein-protein interactions deduced from Arabidopsis knowledgebase.**
(HTM)

Table S6 **Differentially expressed genes in symptomatic fruit in comparison to apparently healthy, annotations and number of protein-protein interactions deduced from Arabidopsis knowledgebase.**
(HTM)

Table S7 **21 genes making the strongest contribution to each of four classes.** Five genes with count and log2foldchange values also appear in two or more of Tables S1, S2, S3 and S6 as indicated.
(HTM)

Table S8 **GO categories of transcripts found in significantly higher levels in pairwise comparisons (indicated as “up” and “down” groups), using Fisher's Exact Test.**
(HTM)

Figure S1 **CTV detection in Citrus using qRT-PCR.** Analysis were conducted for the four categories of fruits: symptomatic (SY), asymptomatic (AS), apparently healthy (AH) and healthy from HLB-free location (CO).
(EPS)

Figure S2 **Principal component analysis of differential gene expression in four HLB sample types.** The length of the loading vector of each target gene indicates that gene's contribution to the separation of four categories of mature fruit peel by the first two principal components (C1, C2). MF-SY, symptomatic; MF-AS, asymptomatic; MF-AH, apparently healthy; MF-CO, HLB-free orchard.
(EPS)

Figure S3 **Gene set enrichment analysis of Citrus fruits.** PageMan analysis using Wilcoxon Rank Sum test, without multiple testing correction and cutoff = 1.0 Comparisons are shown between AS (asymptomatic) and SY (symptomatic), AH (apparently healthy) and SY (symptomatic), CO (HLB-free orchard) and AH (apparently healthy).
(EPS)
