# Supplementary material for: Transcriptome Profiling of Citrus Fruit Response to Huanglongbing Disease
Source: PLoS One. 2012 May 31;7(5):e38039. doi: 10.1371/journal.pone.0038039 (PMC3364978; doi:10.1371/journal.pone.0038039)
Supplement: Table S1 — Differentially expressed genes in symptomatic fruit in comparison to control (healthy in disease-free location), annotations and number of protein-protein interactions deduced from Arabidopsis knowledgebase. (HTM) [file pone.0038039.s001.htm]

Table�S1


# Table�S1

| Table S1. Differentially expressed genes in symptomatic fruit in comparison to control (healthy in disease-free location), annotations and number of protein-protein interactions deduced from Arabidopsis knowledgebase. Those that also appear in Table S7 are hightlighted in color. | | | | | | | | |
|  |  |  |  |  |  |  |  |  |
| GB id | id2 | count CO | count SY | norm CO | norm SY | log2foldchange | PPI | annotation |
| EY693454 | S44237646 | 4826 | 163 | 4128.155 | 190.5544 | -4.437222637 | 444 | heat shock protein 82 |
| CX047422 | S22593365 | 42 | 88 | 35.92675 | 102.876 | 1.517775605 | 69 | septum-promoting gtp-binding protein 1 ame: full=gtpase spg1 ame: full=sid3 protein |
| EY696492 | S44297884 | 42 | 6 | 35.92675 | 7.014271 | -2.356693513 | 69 | microtubule-associated protein rp eb family member 3 ame: full=end-binding protein 3� |
| EY696074 | S44239496 | 108 | 228 | 92.38308 | 266.5423 | 1.528663921 | 64 | asc1-like protein 1 ame: full=alternaria stem canker resistance-like protein 1 |
| CN184953 | S22553179 | 8549 | 1201 | 7312.805 | 1404.023 | -2.380858114 | 61 | heat shock 70 kda protein |
| EY705807 | S44247549 | 986 | 3432 | 843.4233 | 4012.163 | 2.25005141 | 58 | sugar transport protein 13 ame: full=hexose transporter 13 ame: full=multicopy suppressor of snf4 deficiency protein 1 |
| CN191703 | S22550924 | 23 | 111 | 19.67418 | 129.764 | 2.721515319 | 57 | branched-chain-amino-acid aminotransferase chloroplastic� |
| EY694175 | S44297233 | 43 | 11 | 36.78215 | 12.8595 | -1.516171727 | 54 | g2 mitotic-specific cyclin-1 |
| EY747286 | S44276977 | 226 | 34 | 193.3202 | 39.74754 | -2.282054712 | 53 | probable mannitol dehydrogenase ame: full=nad-dependent mannitol dehydrogenase |
| EY718661 | S44256224 | 207 | 806 | 177.0676 | 942.2504 | 2.41181048 | 50 | coatomer subunit beta -1 ame: full=beta -coat protein 1� |
| DC887942 | S47735884 | 104 | 12 | 88.96149 | 14.02854 | -2.664815808 | 49 | fatty acid 2-hydroxylase ame: full=fatty acid alpha-hydroxylase |
| DN620775 | S24240822 | 3 | 18 | 2.566197 | 21.04281 | 3.03562391 | 48 | ucrcs11\_05m20\_f parent washington navel orange scale-infested rind cdna library ucrcs11 citrus sinensis cdna clone mrna |
| DR908750 | S26279627 | 189 | 18 | 161.6704 | 21.04281 | -2.941656014 | 48 | nuclear transport factor 2� |
| CX303205 | S24636242 | 7 | 14 | 5.987792 | 16.36663 | 1.450661409 | 44 | heat shock 70 kda protein |
| CX675417 | S23017596 | 11 | 0 | 9.409388 | 0 | -Inf | 43 | triacylglycerol lipase 2 flags: precursor |
| EY727672 | S44305930 | 62 | 11 | 53.03473 | 12.8595 | -2.044103283 | 41 | chromatin modification-related protein yng2 ame: full=ing1 homolog 2 |
| EY656304 | S44211814 | 101 | 379 | 86.39529 | 443.0681 | 2.358503964 | 34 | hevamine-a includes: ame: full=chitinase includes: ame: full=lysozyme flags: precursor |
| EY664669 | S44288455 | 55 | 15 | 47.04694 | 17.53568 | -1.423807709 | 32 | homocysteine s-methyltransferase 1 ame: full=s-methylmethionine:homocysteine methyltransferase 1� |
| EY649695 | S44206857 | 358 | 66 | 306.2328 | 77.15698 | -1.988760249 | 31 | tubulin beta-7 chain ame: full=beta-7-tubulin |
| EY684585 | S44231499 | 1019 | 2766 | 871.6515 | 3233.579 | 1.891308514 | 31 | threonine dehydratase chloroplastic ame: full=threonine deaminase� |
| EY662694 | S44287810 | 12942 | 316 | 11070.57 | 369.4183 | -4.905330805 | 31 | �kda proline-rich protein |
| CF833058 | S22568663 | 42 | 5 | 35.92675 | 5.845226 | -2.619727919 | 30 | csl zinc finger domain-containing protein |
| FC921949 | S49955777 | 26 | 88 | 22.24037 | 102.876 | 2.20965331 | 30 | s-adenosylmethionine-dependent methyltransferase |
| CK933773 | S22537734 | 25 | 3 | 21.38497 | 3.507136 | -2.60823228 | 29 | chromatin assembly factor subunit |
| EY754184 | S44281777 | 4 | 12 | 3.421596 | 14.02854 | 2.03562391 | 29 | enhancer of polycomb-like protein |
| CK934395 | S22538579 | 6 | 17 | 5.132394 | 19.87377 | 1.95316175 | 27 | dna-directed rna polymerase subunit beta ame: full=pep ame: full=plastid-encoded rna polymerase subunit beta� |
| EY719103 | S44256666 | 291 | 893 | 248.9211 | 1043.957 | 2.068302431 | 26 | sugar transport protein 14 ame: full=hexose transporter 14 |
| EY707908 | S44299542 | 38 | 271 | 32.50516 | 316.8112 | 3.284882937 | 26 | thioredoxin-like 4 |
| EY650454 | S44207504 | 11 | 45 | 9.409388 | 52.60703 | 2.483082887 | 25 | ccr4-not transcription complex subunit 3 ame: full=ccr4-associated factor 3 ame: full=leukocyte receptor cluster member 2 |
| DY305789 | S34125351 | 819 | 1581 | 700.5717 | 1848.26 | 1.39956342 | 24 | guanylate kinase ame: full=gmp kinase |
| EY757748 | S44283689 | 254 | 1568 | 217.2713 | 1833.063 | 3.076686566 | 23 | ctp synthase 2 ame: full=utp--ammonia ligase 2 ame: full=ctp synthetase 2 |
| CX046244 | S22589334 | 681 | 2614 | 582.5267 | 3055.884 | 2.391193847 | 22 | ribose-phosphate pyrophosphokinase 2 ame: full=phosphoribosyl pyrophosphate synthetase 2 ame: full=prs ii |
| EY692586 | S44237338 | 12 | 1 | 10.26479 | 1.169045 | -3.134301092 | 22 | dna replication licensing factor mcm5 ame: full=cdc46 homolog ame: full=p1-cdc46 |
| CX046469 | S22587621 | 324 | 57 | 277.1493 | 66.63558 | -2.05629858 | 21 | PREDICTED: hypothetical protein [Vitis vinifera] |
| EY664033 | S44216939 | 20 | 52 | 17.10798 | 60.79035 | 1.829173032 | 21 | protein |
| DC900352 | S47736649 | 1021 | 52 | 873.3623 | 60.79035 | -3.844666024 | 20 | chloroplast-targeted copper |
| CV886848 | S22584136 | 853 | 1770 | 729.6553 | 2069.21 | 1.503793123 | 19 | aldehyde dehydrogenase family 3 member h1� |
| EY718305 | S44255972 | 9130 | 1220 | 7809.792 | 1426.235 | -2.453072303 | 19 | heat shock protein 101 |
| EY657469 | S44212615 | 318 | 1667 | 272.0169 | 1948.798 | 2.840816843 | 17 | probable inositol transporter 2 |
| EY701465 | S44243991 | 1518 | 299 | 1298.496 | 349.5445 | -1.893292992 | 16 | tubulin-specific chaperone a ame: full=tubulin-folding cofactor a� |
| EY750416 | S44312759 | 652 | 148 | 557.7201 | 173.0187 | -1.68861338 | 15 | 3-ketoacyl- synthase 6� |
| CB290235 | S22555041 | 2131 | 4752 | 1822.855 | 5555.303 | 1.607665652 | 14 | 3-hydroxy-3-methylglutaryl-coenzyme a reductase 1� |
| DN619030 | S24242071 | 29 | 172 | 24.80657 | 201.0758 | 3.018945169 | 14 | protein |
| EY674364 | S44223840 | 94 | 194 | 80.4075 | 226.7948 | 1.4959854 | 14 | ribonucleoside-diphosphate reductase small chain ame: full=ribonucleotide reductase small subunit ame: full=ribonucleoside-diphosphate reductase r2 subunit |
| EY756199 | S44314760 | 24 | 3 | 20.52957 | 3.507136 | -2.549338591 | 14 | arabidopsis thaliana pola2 (dna polymerase alpha 2) dna binding dna-directed dna polymerase complete cds |
| CN190487 | S22548612 | 750 | 1842 | 641.5492 | 2153.381 | 1.74697197 | 13 | �nuclease harbi1 ame: full=harbinger transposase-derived nuclease |
| EY745239 | S44275817 | 404 | 1127 | 345.5812 | 1317.514 | 1.930721726 | 13 | cbl-interacting serine threonine-protein kinase 14 ame: full=sos2-like protein kinase pks24 ame: full=snf1-related kinase ame: full=serine threonine-protein kinase sr1� |
| EY666662 | S44288880 | 326 | 833 | 278.86 | 973.8146 | 1.80410594 | 13 | two-component response regulator-like aprr1 ame: full=pseudo-response regulator 1 ame: full=timing of cab expression 1 ame: full=abi3-interacting protein 1 |
| EY654993 | S44210909 | 131 | 443 | 112.0573 | 517.887 | 2.208401296 | 12 | probable pre-mrna-splicing factor atp-dependent rna helicase |
| EY728087 | S44263101 | 352 | 51 | 301.1004 | 59.6213 | -2.336344868 | 12 | peptide transporter ptr2 ame: full=histidine-transporting protein |
| DC900116 | S47736414 | 26566 | 3166 | 22724.53 | 3701.197 | -2.61818645 | 11 | phosphoenolpyruvate carboxykinase� |
| CX074546 | S22599307 | 32 | 1 | 27.37277 | 1.169045 | -4.549338591 | 10 | interactor of constitutive active rops 4 |
| CX675973 | S23017894 | 29 | 3 | 24.80657 | 3.507136 | -2.822357085 | 10 | probable histone ame: full=hta7 |
| DY257364 | S34124642 | 75 | 659 | 64.15492 | 770.4008 | 3.585977374 | 10 | ammonium transporter 2� |
| DY257490 | S34124768 | 1376 | 2689 | 1177.029 | 3143.563 | 1.417250694 | 10 | calcium-dependent protein kinase 32 |
| EY657623 | S44212657 | 491 | 86 | 420.0009 | 100.5379 | -2.062653051 | 10 | xylem serine proteinase 1� |
| EY755084 | S44313981 | 85 | 15 | 72.70891 | 17.53568 | -2.051838932 | 10 | autophagy-related protein 18 ame: full=cytoplasm to vacuole targeting protein 18 ame: full=swollen vacuole phenotype protein 1 ame: full=needed for premeiotic replication protein 1 |
| CF836343 | S22571875 | 84 | 20 | 71.85351 | 23.3809 | -1.619727919 | 9 | probable 26s proteasome complex subunit sem1-2 |
| CV719482 | S22579072 | 31 | 0 | 26.51737 | 0 | -Inf | 9 | syntaxin-related protein knolle ame: full=syntaxin-111� |
| EY748702 | S44277833 | 20 | 95 | 17.10798 | 111.0593 | 2.698588922 | 9 | 30s ribosomal protein chloroplastic |
| EY693723 | S44297131 | 1312 | 280 | 1122.283 | 327.3327 | -1.777607579 | 9 | sugar carrier protein a |
| EY751712 | S44313067 | 5517 | 11104 | 4719.236 | 12981.08 | 1.459784996 | 9 | glutathione s-transferase erd13 ame: full=gst class-phi |
| FC871499 | S49955153 | 16 | 4 | 13.68638 | 4.676181 | -1.549338591 | 9 | calcineurin b-like protein 7 |
| CX050627 | S22591393 | 25 | 67 | 21.38497 | 78.32603 | 1.87289441 | 8 | histidine-containing phosphotransfer protein 5 |
| DY305483 | S34125045 | 2674 | 10415 | 2287.337 | 12175.61 | 2.412252878 | 8 | ferritin- chloroplastic ame: full=s -3 flags: precursor |
| EY747765 | S44311578 | 59166 | 12718 | 50610.53 | 14867.92 | -1.76723514 | 8 | �kda class ii heat shock protein |
| CF838294 | S22572832 | 7 | 31 | 5.987792 | 36.2404 | 2.597502797 | 7 | ocs element-binding factor 1� |
| CX049208 | S22594324 | 372 | 734 | 318.2084 | 858.0792 | 1.431138851 | 7 | 30s ribosomal protein chloroplastic |
| CX050469 | S22595000 | 53 | 10 | 45.33614 | 11.69045 | -1.955330951 | 7 | agamous-like mads-box protein agl11 |
| DR909276 | S26280153 | 265 | 563 | 226.6807 | 658.1724 | 1.537803972 | 7 | chloride channel protein clc-a� |
| DY305900 | S34125462 | 1542 | 3194 | 1319.025 | 3733.93 | 1.501222957 | 7 | glucan endo- -beta-glucosidase 13 ame: full=(1- |
| DY305942 | S34125504 | 1289 | 2711 | 1102.609 | 3169.282 | 1.523234258 | 7 | glucan endo- -beta-glucosidase 14 ame: full=(1- |
| EY688187 | S44234143 | 57 | 8 | 48.75774 | 9.352362 | -2.382228605 | 7 | hva22-like protein c� |
| EY692055 | S44236919 | 1460 | 3982 | 1248.882 | 4655.138 | 1.898186261 | 7 | inorganic phosphate transporter 1-6� |
| EY707504 | S44249246 | 129 | 30 | 110.3465 | 35.07136 | -1.653675251 | 7 | unknown protein [Arabidopsis thaliana] |
| EY724518 | S44260871 | 2327 | 510 | 1990.513 | 596.213 | -1.73924065 | 7 | oligopeptide transporter 3� |
| CX044490 | S22588416 | 107 | 948 | 91.52768 | 1108.255 | 3.597937672 | 6 | sulfate transporter ame: full=ast12 ame: full= 1 |
| DY305635 | S34125197 | 161 | 605 | 137.7192 | 707.2723 | 2.360535863 | 6 | squalene monooxygenase ame: full=squalene epoxidase� |
| EY683854 | S44231104 | 256 | 578 | 218.9821 | 675.7081 | 1.625587092 | 6 | fructose-bisphosphate cytoplasmic isozyme 1 |
| EY690487 | S44235673 | 12 | 2 | 10.26479 | 2.33809 | -2.134301092 | 6 | nitrate transporter, putative [Ricinus communis] |
| EY702355 | S44244881 | 22 | 3 | 18.81878 | 3.507136 | -2.423807709 | 6 | ubiquitin carrier protein e2 20 |
| EY748686 | S44277817 | 1860 | 3954 | 1591.042 | 4622.405 | 1.538671659 | 6 | cbl-interacting serine threonine-protein kinase 6 ame: full=sos2-like protein kinase pks4 ame: full=snf1-related kinase ame: full=sos3-interacting protein 3 |
| EY757042 | S44283319 | 383 | 952 | 327.6178 | 1112.931 | 1.76427859 | 6 | probable ribose-5-phosphate isomerase ame: full=phosphoriboisomerase |
| EY653615 | S44285171 | 384 | 968 | 328.4732 | 1131.636 | 1.784562146 | 6 | auxin response factor 5 |
| FC871823 | S49955477 | 14 | 36 | 11.97558 | 42.08563 | 1.813231488 | 6 | predicted protein [Populus trichocarpa] |
| EY687316 | S44233580 | 20 | 4 | 17.10798 | 4.676181 | -1.871266686 | 5 | gamma-glutamyltranspeptidase 1 ame: full=gamma-glutamyltransferase 1 contains: ame: full=gamma-glutamyltranspeptidase 1 heavy chain contains: ame: full=gamma-glutamyltranspeptidase 1 light chain flags: precursor |
| EY746955 | S44276870 | 243 | 15 | 207.8619 | 17.53568 | -3.567260499 | 5 | aquaporin |
| CF836050 | S22571733 | 11 | 2 | 9.409388 | 2.33809 | -2.00877021 | 4 | set domain-containing protein |
| CV720168 | S22579462 | 4 | 11 | 3.421596 | 12.8595 | 1.910093028 | 4 | stevia rebaudiana ipp dmapp synthase complete cds |
| CX044129 | S22586518 | 494 | 3166 | 422.5671 | 3701.197 | 3.130739718 | 4 | probable wrky transcription factor 53 ame: full=wrky dna-binding protein 53 |
| CX070827 | S22597584 | 152 | 446 | 130.0206 | 521.3942 | 2.003633795 | 4 | phosphorylated carbohydrates phosphatase tm\_1254 |
| EY653808 | S44209808 | 148 | 24 | 126.599 | 28.05708 | -2.173829456 | 4 | cell division control protein 2 homolog d |
| EY679753 | S44228347 | 1425 | 3744 | 1218.943 | 4376.905 | 1.844279925 | 4 | protein time for coffee |
| EY679947 | S44228429 | 62 | 269 | 53.03473 | 314.4732 | 2.567927461 | 4 | nitrate chlorate transporter |
| EY692066 | S44236930 | 14 | 4 | 11.97558 | 4.676181 | -1.356693513 | 4 | homeotic protein knotted-1� |
| EY701434 | S44243960 | 25 | 2 | 21.38497 | 2.33809 | -3.193194781 | 4 | anaphase-promoting complex subunit cdc20 |
| EY701974 | S44244500 | 515 | 94 | 440.5304 | 109.8902 | -2.003178361 | 4 | vacuolar cation proton exchanger 1 ame: full=ca(2+) h(+) exchanger 1 ame: full=ca(2+) h(+) antiporter cax1 ame: full=protein rare cold inducible 4 |
| EY704060 | S44246138 | 3930 | 3332 | 3361.718 | 3895.259 | 0.212520497 | 4 | pleiotropic drug resistance protein 1 ame: full= 1 |
| EY709191 | S44250400 | 389 | 410 | 332.7502 | 479.3085 | 0.526515164 | 4 | serine carboxypeptidase-like 19 ame: full=sinapoylglucose--choline o-sinapoyltransferase� |
| EY710708 | S44251581 | 305 | 660 | 260.8967 | 771.5698 | 1.564318191 | 4 | probable gibberellin receptor gid1l2 ame: full=gid1-like protein 2 |
| EY752385 | S44280398 | 99 | 18 | 84.68449 | 21.04281 | -2.00877021 | 4 | cyclin-u3-1� |
| EY674034 | S44291660 | 281 | 555 | 240.3671 | 648.8201 | 1.43257905 | 4 | calreticulin-3 flags: precursor |
| EY710755 | S44300374 | 38 | 119 | 32.50516 | 139.1164 | 2.097551659 | 4 | uncharacterized aarf domain-containing protein kinase chloroplastic flags: precursor |
| CV718146 | S22582026 | 207 | 399 | 177.0676 | 466.449 | 1.397419388 | 3 | conserved hypothetical protein [Ricinus communis] |
| CV886686 | S22585644 | 752 | 1412 | 643.26 | 1650.692 | 1.359596931 | 3 | glucan endo- -beta-glucosidase ame: full=(1- |
| CX051384 | S22591744 | 298 | 667 | 254.9089 | 779.7531 | 1.61303584 | 3 | atp synthase subunit chloroplastic ame: full=f-atpase subunit alpha ame: full=atp synthase f1 sector subunit alpha |
| CX048165 | S22593763 | 7 | 18 | 5.987792 | 21.04281 | 1.813231488 | 3 | serine threonine-protein kinase ctr1 |
| CX053639 | S22596692 | 11 | 24 | 9.409388 | 28.05708 | 1.576192291 | 3 | random slug protein 5 ame: full=cral-trio domain-containing protein 5 |
| CX069358 | S22596906 | 69 | 134 | 59.02253 | 156.6521 | 1.408226143 | 3 | atp synthase subunit mitochondrial ame: full=lipid-binding protein |
| CX302518 | S24635505 | 191 | 20 | 163.3812 | 23.3809 | -2.804839324 | 3 | ubiquitin |
| DR908226 | S26279103 | 1357 | 74 | 1160.776 | 86.50934 | -3.746090231 | 3 | thioredoxin h-type 2� |
| DY305494 | S34125056 | 421 | 5957 | 360.1229 | 6964.002 | 4.27335523 | 3 | probable calcium-binding protein cml45 ame: full=calmodulin-like protein 45 |
| DY305968 | S34125530 | 40 | 84 | 34.21596 | 98.1998 | 1.521050737 | 3 | protein |
| DY306092 | S34125654 | 764 | 2464 | 653.5248 | 2880.527 | 2.140019122 | 3 | riboflavin biosynthesis protein chloroplastic includes: ame: full= -dihydroxy-2-butanone 4-phosphate synthase� |
| EY651251 | S44207965 | 214 | 44 | 183.0554 | 51.43799 | -1.831373959 | 3 | vesicle transport protein sft2b ame: full=sft2 domain-containing protein 2 |
| EY652436 | S44208828 | 465 | 990 | 397.7605 | 1157.355 | 1.540859218 | 3 | uncharacterized protein |
| EY652956 | S44209348 | 446 | 102 | 381.5079 | 119.2426 | -1.677813149 | 3 | lys-63-specific deubiquitinase brcc36 ame: full=brca1-a complex subunit brcc36 ame: full=brisc complex subunit brcc36 ame: full=brca1 brca2-containing complex subunit 3 ame: full=brca1 brca2-containing complex subunit 36 |
| EY657959 | S44212993 | 113 | 238 | 96.66008 | 278.2328 | 1.52530021 | 3 | protein |
| EY670058 | S44221312 | 366 | 942 | 313.076 | 1101.241 | 1.81454482 | 3 | disease resistance protein |
| EY679459 | S44228053 | 24 | 0 | 20.52957 | 0 | -Inf | 3 | protein hothead ame: full=protein adhesion of calyx edges flags: precursor |
| EY681315 | S44229573 | 84 | 194 | 71.85351 | 226.7948 | 1.658256828 | 3 | mitochondrial carrier |
| EY691000 | S44236186 | 4068 | 8182 | 3479.763 | 9565.128 | 1.458795267 | 3 | membrane protein |
| EY692100 | S44236964 | 24 | 5 | 20.52957 | 5.845226 | -1.812372997 | 3 | cholesterol transport protein |
| EY706290 | S44248032 | 190 | 2256 | 162.5258 | 2637.366 | 4.020357153 | 3 | probable lrr receptor-like serine threonine-protein kinase at1g56130 flags: precursor |
| EY711989 | S44252316 | 27 | 2 | 23.09577 | 2.33809 | -3.304226093 | 3 | lrr receptor-like serine threonine-protein kinase gso1 ame: full=protein gassho 1 flags: precursor |
| EY714990 | S44254085 | 2101 | 4143 | 1797.193 | 4843.354 | 1.430261067 | 3 | syntaxin-121� |
| EY745245 | S44275823 | 107 | 279 | 91.52768 | 326.1636 | 1.833315734 | 3 | probable udp-glucose:glycoprotein glucosyltransferase a ame: full=developmental gene 1109 protein flags: precursor |
| EY751253 | S44279496 | 83 | 262 | 70.99811 | 306.2898 | 2.109044979 | 3 | polyphosphoinositide phosphatase ame: full=phosphatidylinositol -bisphosphate 5-phosphatase ame: full=factor-induced gene 4 protein |
| EY681747 | S44293241 | 408 | 1015 | 349.0028 | 1186.581 | 1.765500079 | 3 | monoglyceride lipase� |
| EY692393 | S44296361 | 3314 | 515 | 2834.792 | 602.0583 | -2.235267856 | 3 | two-component response regulator-like prr73 ame: full=pseudo-response regulator 73� |
| EY692767 | S44296511 | 2 | 11 | 1.710798 | 12.8595 | 2.910093028 | 3 | beta-hexosaminidase subunit b2 ame: full=n-acetyl-beta-glucosaminidase subunit b2 ame: full=beta-n-acetylhexosaminidase subunit b2 flags: precursor |
| EY694875 | S44297373 | 602 | 1336 | 514.9502 | 1561.844 | 1.600746025 | 3 | dual specificity protein |
| CN191461 | S22549113 | 64 | 124 | 54.74553 | 144.9616 | 1.404857719 | 2 | upf0706 protein at5g01750 |
| CV714332 | S22576139 | 2 | 115 | 1.710798 | 134.4402 | 6.29615146 | 2 | dctp pyrophosphatase 1 ame: full=deoxycytidine-triphosphatase 1� |
| CV886565 | S22584007 | 679 | 132 | 580.8159 | 154.314 | -1.912212236 | 2 | acyl-protein thioesterase 2� |
| CV887095 | S22584249 | 0 | 1 | 0 | 1.169045 | Inf | 2 | bifunctional dihydroflavonol 4-reductase flavanone 4-reductase ame: full=dihydroflavonol 4-reductase� |
| CX043308 | S22586138 | 13 | 2 | 11.12019 | 2.33809 | -2.249778309 | 2 | probable polyamine oxidase 5� |
| CX071523 | S22602384 | 129 | 4 | 110.3465 | 4.676181 | -4.560565846 | 2 | probable auxin efflux carrier component 1c ame: full= 1c |
| CX072825 | S22603084 | 123 | 279 | 105.2141 | 326.1636 | 1.632268215 | 2 | 60s ribosomal protein mitochondrial |
| CX676405 | S23018114 | 318 | 850 | 272.0169 | 993.6884 | 1.869097485 | 2 | upf0717 protein at5g11950 |
| CX671540 | S23020242 | 17 | 75 | 14.54178 | 87.67839 | 2.592017258 | 2 | primary amine oxidase ame: full=amine oxidase flags: precursor |
| DN620167 | S24240472 | 52 | 489 | 44.48074 | 571.6631 | 3.683912346 | 2 | arogenate dehydrogenase chloroplastic ame: full= 2 flags: precursor |
| CX300811 | S24634749 | 0 | 1 | 0 | 1.169045 | Inf | 2 | uncharacterized oxidoreductase yhdf |
| DY305576 | S34125138 | 1539 | 5244 | 1316.459 | 6130.473 | 2.219335863 | 2 | 12-oxophytodienoate reductase 2 ame: full=12-oxophytodienoate- -reductase 2� |
| DY305680 | S34125242 | 135 | 261 | 115.4789 | 305.1208 | 1.401751809 | 2 | 33 kda chloroplastic flags: precursor |
| DY305806 | S34125368 | 1185 | 273 | 1013.648 | 319.1493 | -1.667252794 | 2 | probable sarcosine oxidase |
| DY306123 | S34125685 | 1751 | 6110 | 1497.804 | 7142.866 | 2.253654705 | 2 | probable wrky transcription factor 40 ame: full=wrky dna-binding protein 40 |
| EY654511 | S44210427 | 178 | 404 | 152.261 | 472.2943 | 1.633139461 | 2 | 2-oxoisovalerate dehydrogenase subunit mitochondrial ame: full=branched-chain alpha-keto acid dehydrogenase e1 component alpha chain� |
| EY657484 | S44212630 | 8 | 38 | 6.843191 | 44.42372 | 2.698588922 | 2 | ser thr-rich protein t10 in dgcr region |
| EY657971 | S44213005 | 9 | 2 | 7.69859 | 2.33809 | -1.719263592 | 2 | kinesin-like protein kif22 ame: full=chromokinesin kid |
| EY678067 | S44226885 | 69 | 375 | 59.02253 | 438.3919 | 2.892883738 | 2 | asparagine synthetase ame: full=glutamine-dependent asparagine synthetase |
| EY702712 | S44245126 | 617 | 127 | 527.7811 | 148.4687 | -1.829780583 | 2 | microtubule-associated protein map65-1a |
| EY722375 | S44259274 | 837 | 18 | 715.9689 | 21.04281 | -5.088497402 | 2 | s-adenosylmethionine decarboxylase proenzyme� |
| EY723481 | S44260044 | 222 | 740 | 189.8986 | 865.0934 | 2.187627003 | 2 | siroheme synthase includes: ame: full=uroporphyrinogen-iii c-methyltransferase� |
| EY744544 | S44275220 | 89 | 6 | 76.1305 | 7.014271 | -3.440109521 | 2 | populus trichocarpa histidine kinase osmosensor mrna |
| EY746794 | S44276709 | 35 | 4 | 29.93896 | 4.676181 | -2.678621608 | 2 | cyclin-d2-1 ame: full=g1 s-specific cyclin-d2-1� |
| EY750241 | S44278826 | 2748 | 121 | 2350.636 | 141.4545 | -4.054641643 | 2 | brassinosteroid-regulated protein bru1 flags: precursor |
| EY757319 | S44283372 | 130 | 513 | 111.2019 | 599.7202 | 2.431108612 | 2 | regulatory protein npr1 ame: full=nonexpresser of pr genes 1 ame: full=non-inducible immunity protein 1� |
| EY650266 | S44284160 | 341 | 1147 | 291.691 | 1340.895 | 2.200683156 | 2 | probable protein phosphatase 2c 25� |
| EY656741 | S44285847 | 119 | 13 | 101.7925 | 15.19759 | -2.743716636 | 2 | glucan endo- -beta-glucosidase 11 ame: full=(1- |
| EY676847 | S44292121 | 206 | 413 | 176.2122 | 482.8157 | 1.454158853 | 2 | two-component response regulator-like aprr2 ame: full=pseudo-response regulator 2 ame: full=toc2 protein |
| EY727748 | S44306006 | 1073 | 2135 | 917.843 | 2495.911 | 1.443247403 | 2 | ethylene-responsive transcription factor 3 ame: full=ethylene-responsive element-binding factor 3 homolog ame: full=ethylene-responsive element-binding factor 5� |
| EY743302 | S44309994 | 385 | 78 | 329.3286 | 91.18553 | -1.852651008 | 2 | beta-glucosidase 6� |
| EY743728 | S44310420 | 975 | 156 | 834.0139 | 182.3711 | -2.193194781 | 2 | �homolog subfamily b member 6 |
| EY747770 | S44311583 | 154 | 316 | 131.7314 | 369.4183 | 1.487655616 | 2 | transcription factor bim1 ame: full=bes1-interacting myc-like protein 1 ame: full=transcription factor en 126 ame: full=bhlh transcription factor bhlh046 ame: full=basic helix-loop-helix protein 46� |
| DC900125 | S47736423 | 1115 | 259 | 953.7698 | 302.7827 | -1.655358298 | 2 | uncharacterized protein ylba |
| DC900331 | S47736628 | 76 | 7 | 65.01032 | 8.183316 | -2.989911182 | 2 | homeobox-leucine zipper protein athb-13 ame: full=homeodomain transcription factor athb-13 ame: full=hd-zip protein athb-13 |
| CN187848 | S22545642 | 778 | 1541 | 665.5004 | 1801.499 | 1.436686211 | 1 | dof zinc finger protein� |
| CV713259 | S22575531 | 55 | 4 | 47.04694 | 4.676181 | -3.330698305 | 1 | acid phosphatase 1 ame: full=apase-1 flags: precursor |
| CV719908 | S22579316 | 109 | 231 | 93.23848 | 270.0494 | 1.534226126 | 1 | gtp-binding protein yptv3 |
| CV712407 | S22579576 | 52 | 115 | 44.48074 | 134.4402 | 1.595711742 | 1 | nad h-quinone oxidoreductase subunit chloroplastic ame: full=nad h dehydrogenase subunit h ame: full=nadh-plastoquinone oxidoreductase subunit h ame: full=nadh-plastoquinone oxidoreductase 49 kda subunit |
| CV887297 | S22585971 | 70 | 15 | 59.87792 | 17.53568 | -1.771731012 | 1 | patellin-6 |
| CX046632 | S22589541 | 105 | 254 | 89.81689 | 296.9375 | 1.725100578 | 1 | beta- insoluble isoenzyme cwinv3 ame: full=cell wall invertase 3� |
| CX048001 | S22590174 | 5 | 38 | 4.276995 | 44.42372 | 3.376660828 | 1 | nad h-quinone oxidoreductase subunit chloroplastic ame: full=nad h dehydrogenase subunit i� |
| CX047376 | S22593342 | 29 | 4 | 24.80657 | 4.676181 | -2.407319586 | 1 | probable xyloglucan endotransglucosylase hydrolase protein 33� |
| CX052350 | S22596008 | 3 | 9 | 2.566197 | 10.52141 | 2.03562391 | 1 | cysteine proteinase 15a ame: full=turgor-responsive protein 15a flags: precursor |
| CX052367 | S22596020 | 371 | 82 | 317.353 | 95.86171 | -1.727061963 | 1 | two-component response regulator arr3 |
| CX053924 | S22596841 | 20 | 3 | 17.10798 | 3.507136 | -2.286304185 | 1 | kinesin-1 ame: full=kinesin-like protein a |
| CX069962 | S22601546 | 80 | 169 | 68.43191 | 197.5686 | 1.52961275 | 1 | regulatory protein npr1 ame: full=nonexpresser of pr genes 1 ame: full=non-inducible immunity protein 1� |
| CX078571 | S22606132 | 254 | 25 | 217.2713 | 29.22613 | -2.894167088 | 1 | peptidyl-trna hydrolase mitochondrial� |
| CX673824 | S23019466 | 183 | 424 | 156.538 | 495.6752 | 1.662882025 | 1 | chaperone protein dnaj 13� |
| DR404544 | S25679542 | 536 | 3265 | 458.4938 | 3816.933 | 3.057439495 | 1 | protein |
| DY257165 | S34124443 | 4897 | 17930 | 4188.889 | 20960.98 | 2.323066796 | 1 | probable ccr4-associated factor 1 homolog 9 |
| DY305646 | S34125208 | 902 | 1893 | 771.5698 | 2213.003 | 1.520136481 | 1 | secretory carrier-associated membrane protein 3� |
| DY305851 | S34125413 | 10955 | 49842 | 9370.895 | 58267.55 | 2.636433885 | 1 | zinc finger protein 1 ame: full=wzf1 |
| DY305917 | S34125479 | 5372 | 15037 | 4595.203 | 17578.93 | 1.935646966 | 1 | populus trichocarpa ap2 erf domain-containing transcription factor mrna |
| DY306032 | S34125594 | 1048 | 2742 | 896.4581 | 3205.522 | 1.838251263 | 1 | protein tify 10a ame: full=jasmonate zim domain-containing protein 1 |
| EY657719 | S44212753 | 91 | 198 | 77.8413 | 231.4709 | 1.572223389 | 1 | serine threonine-protein kinase srk2f ame: full=ost1-kinase-like 5 ame: full=snf1-related kinase� |
| EY659645 | S44214119 | 381 | 1309 | 325.907 | 1530.28 | 2.231263603 | 1 | endo beta n- |
| EY674382 | S44223858 | 1197 | 2449 | 1023.913 | 2862.992 | 1.483431031 | 1 | photosystem i reaction center subunit chloroplastic ame: full=psi-g flags: precursor |
| EY676061 | S44225327 | 6102 | 13588 | 5219.644 | 15884.99 | 1.605640447 | 1 | probable mitochondrial 2-oxoglutarate malate carrier protein� |
| EY677812 | S44226630 | 2091 | 4719 | 1788.639 | 5516.724 | 1.624949519 | 1 | probable wrky transcription factor 33 ame: full=wrky dna-binding protein 33 |
| EY689158 | S44234666 | 147 | 439 | 125.7436 | 513.2108 | 2.029066194 | 1 | photosystem i p700 chlorophyll a apoprotein a2 ame: full= ame: full=psi-b |
| EY690668 | S44235854 | 16 | 0 | 13.68638 | 0 | -Inf | 1 | uncharacterized amino acid permease yhdg |
| EY694066 | S44238146 | 1723 | 118 | 1473.852 | 137.9473 | -3.417402528 | 1 | chloroplast-targeted copper |
| EY700195 | S44243057 | 22 | 64 | 18.81878 | 74.81889 | 1.99122979 | 1 | f-box lrr-repeat protein 3 |
| EY702054 | S44244580 | 72 | 918 | 61.58872 | 1073.183 | 4.123086751 | 1 | tropinone reductase homolog at1g07440 |
| EY750687 | S44278936 | 434 | 933 | 371.2431 | 1090.719 | 1.554843447 | 1 | e3 ubiquitin-protein ligase rglg2 ame: full=ring domain ligase 2 |
| EY751217 | S44279460 | 101 | 21 | 86.39529 | 24.54995 | -1.815232651 | 1 | acid phosphatase 1 ame: full=apase-1 flags: precursor |
| EY650241 | S44284135 | 1161 | 8289 | 993.1181 | 9690.216 | 3.2864915 | 1 | zinc finger protein constans-like 1 |
| EY658428 | S44286330 | 95 | 364 | 81.2629 | 425.5325 | 2.388600441 | 1 | inositol 2-dehydrogenase ame: full=myo-inositol 2-dehydrogenase� |
| EY661115 | S44287113 | 42 | 110 | 35.92675 | 128.595 | 1.8397037 | 1 | palmitoyl-monogalactosyldiacylglycerol delta-7 chloroplastic ame: full=monogalactosyldiacylglycerol-specific palmitic acid desaturase ame: full=fad5 flags: precursor |
| EY686801 | S44294717 | 35 | 1 | 29.93896 | 1.169045 | -4.678621608 | 1 | cyclin-d1-1 ame: full=g1 s-specific cyclin-d1-1� |
| EY719197 | S44303656 | 32 | 85 | 27.37277 | 99.36884 | 1.860052345 | 1 | probable 6-phosphogluconolactonase 1� |
| EY727780 | S44306038 | 2448 | 280 | 2094.017 | 327.3327 | -2.677443417 | 1 | lipoxygenase chloroplastic� |
| EY745565 | S44310689 | 9 | 0 | 7.69859 | 0 | -Inf | 1 | probable serine threonine-protein kinase tsua ame: full=tsunami |
| EY746577 | S44311160 | 268 | 604 | 229.2469 | 706.1033 | 1.622976958 | 1 | f-box protein skip2 ame: full=skp1-interacting partner 2 |
| BQ623330 | S22531149 | 38 | 100 | 32.50516 | 116.9045 | 1.846590085 | 0 | usda-fp\_00421 ridge pineapple sweet orange entire seedling citrus sinensis cdna clone usda-fp\_00421 5 mrna |
| BQ623495 | S22531314 | 13 | 1 | 11.12019 | 1.169045 | -3.249778309 | 0 | usda-fp\_00586 ridge pineapple sweet orange entire seedling citrus sinensis cdna clone usda-fp\_00586 5 mrna |
| BQ624033 | S22531852 | 55 | 10 | 47.04694 | 11.69045 | -2.00877021 | 0 | usda-fp\_01124 ridge pineapple sweet orange entire seedling citrus sinensis cdna clone usda-fp\_01124 5 mrna |
| BQ624298 | S22532117 | 44 | 0 | 37.63755 | 0 | -Inf | 0 | protein |
| BQ624431 | S22532250 | 654 | 594 | 559.4309 | 694.4128 | 0.311833704 | 0 | protein |
| BQ624477 | S22532296 | 57 | 2 | 48.75774 | 2.33809 | -4.382228605 | 0 | blue copper protein flags: precursor |
| BQ624512 | S22532331 | 3 | 10 | 2.566197 | 11.69045 | 2.187627003 | 0 | probable glutathione s-transferase ame: full=heat shock protein 26a ame: full=g2-4 |
| BQ624820 | S22532639 | 14 | 2 | 11.97558 | 2.33809 | -2.356693513 | 0 | protein gast1 flags: precursor |
| BQ625029 | S22532848 | 117 | 428 | 100.0817 | 500.3513 | 2.321763676 | 0 | uncharacterized protein at1g14870 |
| BQ625107 | S22532926 | 95 | 6 | 81.2629 | 7.014271 | -3.534231699 | 0 | protein |
| BQ625197 | S22533016 | 47 | 7 | 40.20375 | 8.183316 | -2.296572521 | 0 | sambucus nigra clone xp2 expansin complete cds |
| CK665446 | S22533829 | 215 | 37 | 183.9108 | 43.25467 | -2.088078075 | 0 | conserved hypothetical protein [Ricinus communis] |
| CK701562 | S22534644 | 1621 | 2538 | 1386.602 | 2967.037 | 1.097469387 | 0 | usda-fp\_4822 ridge pineapple sweet orange entire seedling citrus sinensis cdna clone rse36a03 5 mrna |
| CK701701 | S22534723 | 258 | 6 | 220.6929 | 7.014271 | -4.975603346 | 0 | usda-fp\_4962 ridge pineapple sweet orange entire seedling citrus sinensis cdna clone rse37e10 5 mrna |
| CK701747 | S22535105 | 17 | 1 | 14.54178 | 1.169045 | -3.636801432 | 0 | usda-fp\_5008 ridge pineapple sweet orange entire seedling citrus sinensis cdna clone rse39e04 5 mrna |
| CK739677 | S22536036 | 21 | 8 | 17.96338 | 9.352362 | -0.941656014 | 0 | predicted protein [Populus trichocarpa] |
| CK739846 | S22536135 | 10 | 1 | 8.553989 | 1.169045 | -2.871266686 | 0 | elongation factor 1-alpha� |
| CK740222 | S22536764 | 4 | 108 | 3.421596 | 126.2569 | 5.205548911 | 0 | protein |
| CK932708 | S22536924 | 63 | 9 | 53.89013 | 10.52141 | -2.356693513 | 0 | endoglucanase 8 ame: full=endo- -beta glucanase 8 ame: full=cellulase 1� |
| CK932724 | S22536939 | 63 | 176 | 53.89013 | 205.752 | 1.932813104 | 0 | ORF493 [Pinus koraiensis] |
| CK932916 | S22537042 | 90 | 21 | 76.9859 | 24.54995 | -1.648874265 | 0 | cgf1004350\_a01 developing fruit juice sac at 38 dafb citrus sinensis cdna clone jsjune0004\_ivf\_a01 5 mrna |
| CK932957 | S22537083 | 182 | 41 | 155.6826 | 47.93085 | -1.699581227 | 0 | �kda heat shock peroxisomal� |
| CK933067 | S22537163 | 195 | 17 | 166.8028 | 19.87377 | -3.069206063 | 0 | cgf1004348\_a07 developing fruit juice sac at 38 dafb citrus sinensis cdna clone jsjune0004\_iif\_a07 5 mrna |
| CK933074 | S22537170 | 12 | 2 | 10.26479 | 2.33809 | -2.134301092 | 0 | crambin precursor=thionin variant thi2ca9 |
| CK933216 | S22537247 | 19 | 3 | 16.25258 | 3.507136 | -2.212303604 | 0 | cgf1004289\_b08 developing fruit juice sac at 38 dafb citrus sinensis cdna clone jsjune0002\_ivf\_b08 5 mrna |
| CK933484 | S22537451 | 82 | 4 | 70.14271 | 4.676181 | -3.906890596 | 0 | xyloglucan endotransglucosylase hydrolase protein 9� |
| CK933521 | S22537482 | 219 | 282 | 187.3324 | 329.6707 | 0.815425702 | 0 | cgf1004284\_e11 developing fruit juice sac at 38 dafb citrus sinensis cdna clone jsjune0003\_iiif\_e11 5 mrna |
| CK933841 | S22537808 | 43 | 0 | 36.78215 | 0 | -Inf | 0 | cgf1004278\_f03 developing fruit juice sac at 38 dafb citrus sinensis cdna clone jsjune0001\_if\_f03 5 mrna |
| CK933171 | S22538027 | 1 | 7 | 0.855399 | 8.183316 | 3.258016331 | 0 | cgf1004289\_h05 developing fruit juice sac at 38 dafb citrus sinensis cdna clone jsjune0002\_ivf\_h05 5 mrna |
| CK933909 | S22538125 | 12 | 228 | 10.26479 | 266.5423 | 4.698588922 | 0 | �gem-like protein 8 |
| CK933934 | S22538150 | 741 | 127 | 633.8506 | 148.4687 | -2.093983637 | 0 | conserved hypothetical protein [Ricinus communis] |
| CK934089 | S22538305 | 10 | 35 | 8.553989 | 40.91658 | 2.258016331 | 0 | medicago truncatula chromosome 7 clone mth2- complete sequence |
| CK934145 | S22538361 | 936 | 190 | 800.6534 | 222.1186 | -1.849847702 | 0 | protein |
| CK934608 | S22538786 | 239 | 565 | 204.4403 | 660.5105 | 1.691901658 | 0 | photosystem ii cp47 chlorophyll apoprotein ame: full=psii 47 kda protein ame: full=protein cp-47 |
| CK934631 | S22538815 | 53 | 12 | 45.33614 | 14.02854 | -1.692296545 | 0 | protein |
| CK934874 | S22539052 | 294 | 274 | 251.4873 | 320.3184 | 0.349021147 | 0 | cgf1004225\_h01 developing fruit peel at 38 dafb citrus sinensis cdna clone p38dab10003\_ivf\_h01 5 mrna |
| CK934971 | S22539149 | 69 | 2 | 59.02253 | 2.33809 | -4.657863048 | 0 | protein |
| CK935156 | S22539338 | 4 | 8 | 3.421596 | 9.352362 | 1.450661409 | 0 | cgf1004177\_c06 developing fruit peel at 38 dafb citrus sinensis cdna clone p38da0001\_iif\_c06 5 mrna |
| CK935175 | S22539353 | 5407 | 10561 | 4625.142 | 12346.29 | 1.416507595 | 0 | wun1\_soltuwound-induced protein 1 |
| CK935574 | S22539458 | 2 | 47 | 1.710798 | 54.94512 | 5.005250261 | 0 | protein |
| CK935341 | S22539631 | 502 | 1366 | 429.4103 | 1596.916 | 1.894859623 | 0 | unknown [Populus trichocarpa] |
| CK935355 | S22539635 | 171 | 40 | 146.2732 | 46.76181 | -1.645263011 | 0 | cgf1004562\_f08 developing fruit 24 dafb citrus sinensis cdna clone t24dab0001\_if\_f08 5 mrna |
| CK935394 | S22539659 | 21 | 138 | 17.96338 | 161.3282 | 3.166868443 | 0 | cgf1004562\_c10 developing fruit 24 dafb citrus sinensis cdna clone t24dab0001\_if\_c10 5 mrna |
| CK935405 | S22539665 | 150 | 28 | 128.3098 | 32.73327 | -1.970802359 | 0 | unknown [Glycine max] |
| CK935463 | S22539693 | 26 | 59 | 22.24037 | 68.97367 | 1.63286474 | 0 | cytokinin-o-glucosyltransferase 1 ame: full=zeatin o-glucosyltransferase 1� |
| CK935746 | S22539858 | 2827 | 321 | 2418.213 | 375.2635 | -2.687965272 | 0 | snakin-2 flags: precursor |
| CK935886 | S22539998 | 134 | 2591 | 114.6235 | 3028.996 | 4.723865519 | 0 | protein |
| CK936202 | S22540314 | 1881 | 347 | 1609.005 | 405.6587 | -1.987830872 | 0 | phosphoenolpyruvate carboxykinase� |
| CK936343 | S22540460 | 711 | 33 | 608.1886 | 38.57849 | -3.978650221 | 0 | subtilisin-like protease ame: full=cucumisin-like serine protease flags: precursor |
| CK936435 | S22540547 | 4 | 10 | 3.421596 | 11.69045 | 1.772589504 | 0 | cgf1004382\_d07 developing fruit 24 dafb citrus sinensis cdna clone t24dab0001\_ivf\_d07 5 mrna |
| CK936468 | S22540575 | 11 | 75 | 9.409388 | 87.67839 | 3.220048481 | 0 | probable mitochondrial chaperone bcs1 ame: full=bcs1-like protein |
| CK936936 | S22540813 | 149 | 115 | 127.4544 | 134.4402 | 0.076982939 | 0 | predicted protein [Populus trichocarpa] |
| CK936515 | S22540833 | 7 | 25 | 5.987792 | 29.22613 | 2.287162677 | 0 | cgf1004521\_e12 developing fruit flavedo at 80 dafb citrus sinensis cdna clone f80dab0001\_ivf\_e12 5 mrna |
| CK936548 | S22540860 | 2 | 11 | 1.710798 | 12.8595 | 2.910093028 | 0 | hat family dimerisation |
| CK936724 | S22540992 | 23 | 77 | 19.67418 | 90.01648 | 2.193885994 | 0 | photosystem ii protein l |
| CK937027 | S22541128 | 39 | 3 | 33.36056 | 3.507136 | -3.249778309 | 0 | predicted protein [Populus trichocarpa] |
| CK937174 | S22541286 | 1478 | 3584 | 1264.28 | 4189.858 | 1.728585777 | 0 | calmodulin-like protein 9� |
| CK937331 | S22541446 | 17 | 84 | 14.54178 | 98.1998 | 2.755515991 | 0 | dna-directed rna polymerase subunit beta ame: full=pep ame: full=plastid-encoded rna polymerase subunit beta� |
| CK937870 | S22541980 | 106 | 97 | 90.67229 | 113.3974 | 0.322653797 | 0 | cgf1004480\_h02 developing fruit albedo at 80 dafb in p x2 vector citrus sinensis cdna clone a80dab0001\_iif\_h02 5 mrna |
| CK938028 | S22542069 | 6 | 15 | 5.132394 | 17.53568 | 1.772589504 | 0 | cgf1004479\_b01 developing fruit albedo at 80 dafb in p x2 vector citrus sinensis cdna clone a80dab0001\_if\_b01 5 mrna |
| CK937430 | S22542279 | 824 | 1709 | 704.8487 | 1997.898 | 1.503097564 | 0 | zinc finger protein constans-like 1 |
| CK938229 | S22542521 | 0 | 57 | 0 | 66.63558 | Inf | 0 | cgf1004476\_e07 developing fruit albedo at 80 dafb in p x2 vector citrus sinensis cdna clone a80dab0002\_iif\_e07 5 mrna |
| CK938890 | S22542633 | 510 | 80 | 436.2535 | 93.52362 | -2.221763933 | 0 | protease inhibitor seed storage lipid transfer protein family protein |
| CK938959 | S22542713 | 7 | 44 | 5.987792 | 51.43799 | 3.102738106 | 0 | 30s ribosomal protein chloroplastic |
| CK939007 | S22542756 | 89 | 12 | 76.1305 | 14.02854 | -2.440109521 | 0 | peptide transporter ptr5 |
| CK938415 | S22542876 | 44 | 7 | 37.63755 | 8.183316 | -2.201415288 | 0 | cgf1004443\_b08 developing fruit albedo at 165 dafb citrus sinensis cdna clone a1650001\_ivf\_b08 5 mrna |
| CK939214 | S22543236 | 12 | 25 | 10.26479 | 29.22613 | 1.509555098 | 0 | ras-related protein raba2a ame: full=ras-related protein rab11c |
| CK939266 | S22543283 | 145 | 21 | 124.0328 | 24.54995 | -2.336930258 | 0 | cgf1004432\_c03 developing fruit albedo at 165 dafb citrus sinensis cdna clone a1650003\_if\_c03 5 mrna |
| CK938403 | S22543304 | 3065 | 6014 | 2621.798 | 7030.638 | 1.423099203 | 0 | cgf1004443\_c08 developing fruit albedo at 165 dafb citrus sinensis cdna clone a1650001\_ivf\_c08 5 mrna |
| CK938405 | S22543309 | 11 | 52 | 9.409388 | 60.79035 | 2.691669509 | 0 | cgf1004443\_c10 developing fruit albedo at 165 dafb citrus sinensis cdna clone a1650001\_ivf\_c10 5 mrna |
| CK938476 | S22543340 | 387 | 988 | 331.0394 | 1155.017 | 1.802838884 | 0 | probable protein phosphatase 2c 73� |
| CK938628 | S22543406 | 984 | 1982 | 841.7125 | 2317.048 | 1.460888151 | 0 | bel1-like homeodomain protein 1� |
| CK938667 | S22543424 | 243 | 1096 | 207.8619 | 1281.274 | 2.623880988 | 0 | protein |
| CK939516 | S22543639 | 1510 | 323 | 1291.652 | 377.6016 | -1.774281071 | 0 | snakin-1 flags: precursor |
| CK939541 | S22543662 | 467 | 2326 | 399.4713 | 2719.199 | 2.767018051 | 0 | ethylene-responsive transcription factor 1a� |
| CK939678 | S22543802 | 106 | 460 | 90.67229 | 537.7608 | 2.568231005 | 0 | hypothetical protein [Vitis vinifera] |
| CK939668 | S22543803 | 7 | 33 | 5.987792 | 38.57849 | 2.687700606 | 0 | cgf1004747\_c07 developing fruit flavedo at 165 dafb citrus sinensis cdna clone f1650002\_iiif\_c07 5 mrna |
| CK939814 | S22543943 | 15 | 3 | 12.83098 | 3.507136 | -1.871266686 | 0 | cgf1004745\_c08 developing fruit flavedo at 165 dafb citrus sinensis cdna clone f1650002\_if\_c08 5 mrna |
| CK939844 | S22543970 | 677 | 29 | 579.1051 | 33.90231 | -4.094369619 | 0 | cgf1004744\_h08 developing fruit flavedo at 165 dafb citrus sinensis cdna clone f1650003\_ivf\_h08 5 mrna |
| CK939969 | S22544098 | 13 | 3 | 11.12019 | 3.507136 | -1.664815808 | 0 | cgf1004743\_c11 developing fruit flavedo at 165 dafb citrus sinensis cdna clone f1650003\_iiif\_c11 5 mrna |
| CK940014 | S22544138 | 23 | 129 | 19.67418 | 150.8068 | 2.938326708 | 0 | cgf1004742\_e04 developing fruit flavedo at 165 dafb citrus sinensis cdna clone f1650003\_iif\_e04 5 mrna |
| CK940099 | S22544223 | 126 | 903 | 107.7803 | 1055.648 | 3.291963663 | 0 | calcium-binding protein cml37 ame: full=calmodulin-like protein 37 |
| CN185801 | S22544640 | 5 | 91 | 4.276995 | 106.3831 | 4.636527954 | 0 | probable lrr receptor-like serine threonine-protein kinase at2g23950 flags: precursor |
| CN185980 | S22544726 | 12 | 30 | 10.26479 | 35.07136 | 1.772589504 | 0 | ucrcs05\_0002c20\_f washington navel orange stored fruit pulp cdna library citrus sinensis cdna clone mrna |
| CN186662 | S22545043 | 21 | 787 | 17.96338 | 920.0386 | 5.678563812 | 0 | indole-3-acetic acid-amido synthetase ame: full=auxin-responsive gh3-like protein 4� |
| CN187647 | S22545540 | 11 | 22 | 9.409388 | 25.71899 | 1.450661409 | 0 | predicted protein [Populus trichocarpa] |
| CN188027 | S22545742 | 124 | 21 | 106.0695 | 24.54995 | -2.111217479 | 0 | predicted protein [Populus trichocarpa] |
| CN188208 | S22545836 | 120 | 37 | 102.6479 | 43.25467 | -1.246775821 | 0 | thaumatin-like protein 1 flags: precursor |
| CN185600 | S22546315 | 116 | 17 | 99.22628 | 19.87377 | -2.319856745 | 0 | ucrcs05\_0001j12\_f washington navel orange stored fruit pulp cdna library citrus sinensis cdna clone mrna |
| CN186015 | S22546485 | 5 | 7 | 4.276995 | 8.183316 | 0.936088236 | 0 | protein |
| CN186149 | S22546539 | 349 | 1522 | 298.5342 | 1779.287 | 2.575330826 | 0 | protein |
| CN188179 | S22547436 | 197 | 589 | 168.5136 | 688.5676 | 2.030733413 | 0 | unknown protein [Oryza sativa Japonica Group] |
| CN188339 | S22547505 | 13555 | 3291 | 11594.93 | 3847.328 | -1.591565773 | 0 | heat shock 70 kda protein |
| CN188518 | S22547586 | 390 | 836 | 333.6056 | 977.3218 | 1.550690227 | 0 | �kda class i heat shock protein ame: full= |
| CN188941 | S22547810 | 131 | 20 | 112.0573 | 23.3809 | -2.260833498 | 0 | predicted protein [Populus trichocarpa] |
| CN190127 | S22548431 | 36 | 143 | 30.79436 | 167.1735 | 2.440607744 | 0 | ucrcs06\_0002p09\_r washington navel orange stored fruit rind cdna library citrus sinensis cdna clone mrna |
| CN191394 | S22549085 | 398 | 5034 | 340.4488 | 5884.974 | 4.11152629 | 0 | protein |
| CN191560 | S22549168 | 316 | 737 | 270.3061 | 861.5863 | 1.67240147 | 0 | vitis vinifera contig whole genome shotgun sequence |
| CN189156 | S22549853 | 32 | 221 | 27.37277 | 258.359 | 3.238563968 | 0 | myb-like protein j |
| CN191678 | S22550905 | 68 | 238 | 58.16713 | 278.2328 | 2.258016331 | 0 | ucrcs06\_0005k01\_f washington navel orange stored fruit rind cdna library citrus sinensis cdna clone mrna |
| CN191705 | S22550915 | 14 | 35 | 11.97558 | 40.91658 | 1.772589504 | 0 | predicted protein [Populus trichocarpa] |
| CN182786 | S22551989 | 13 | 33 | 11.12019 | 38.57849 | 1.79461581 | 0 | flavonol synthase flavanone 3-hydroxylase |
| CN183186 | S22552207 | 4 | 11 | 3.421596 | 12.8595 | 1.910093028 | 0 | predicted protein [Populus trichocarpa] |
| CN184985 | S22553198 | 9 | 18 | 7.69859 | 21.04281 | 1.450661409 | 0 | protein gast1 flags: precursor |
| CN181701 | S22553417 | 14 | 32 | 11.97558 | 37.40945 | 1.643306487 | 0 | peroxidase 60� |
| CN182947 | S22553931 | 105 | 272 | 89.81689 | 317.9803 | 1.823878733 | 0 | �glutamine amidotransferase-like protein yvde homolog |
| CN185028 | S22554792 | 58 | 210 | 49.61314 | 245.4995 | 2.306925932 | 0 | ucrcs04\_0006j12\_r ruby orange developing flower cdna library citrus sinensis cdna clone mrna |
| CB250257 | S22554989 | 3 | 27 | 2.566197 | 31.56422 | 3.62058641 | 0 | polygalacturonase� |
| CB290387 | S22555126 | 195 | 459 | 166.8028 | 536.5917 | 1.685681439 | 0 | probable gibberellin receptor gid1l2 ame: full=gid1-like protein 2 |
| CB290628 | S22555264 | 2 | 10 | 1.710798 | 11.69045 | 2.772589504 | 0 | PREDICTED: hypothetical protein [Vitis vinifera] |
| CB290890 | S22555410 | 286 | 604 | 244.6441 | 706.1033 | 1.529194812 | 0 | hydrolyzing o-glycosyl |
| CB291261 | S22555615 | 5202 | 2582 | 4449.785 | 3018.475 | -0.55991599 | 0 | PREDICTED: hypothetical protein [Vitis vinifera] |
| CB291342 | S22555660 | 9 | 29 | 7.69859 | 33.90231 | 2.138717403 | 0 | aluminum-activated malate transporter-like |
| CB291381 | S22555678 | 14 | 3 | 11.97558 | 3.507136 | -1.771731012 | 0 | cdgsh iron sulfur domain-containing protein 1 ame: full= |
| CB291413 | S22555698 | 6 | 12 | 5.132394 | 14.02854 | 1.450661409 | 0 | ucrcs01\_02de03\_g1 washington navel orange cold acclimated flavedo & albedo cdna library citrus sinensis cdna clone mrna |
| CB291872 | S22555958 | 39 | 92 | 33.36056 | 107.5522 | 1.688821146 | 0 | ucrcs01\_03cd05\_g1 washington navel orange cold acclimated flavedo & albedo cdna library citrus sinensis cdna clone mrna |
| CB291943 | S22555999 | 224 | 473 | 191.6094 | 552.9584 | 1.52900286 | 0 | predicted protein [Populus trichocarpa] |
| CB292276 | S22556183 | 31 | 137 | 26.51737 | 160.1592 | 2.594497182 | 0 | protein |
| CB292402 | S22556252 | 2076 | 335 | 1775.808 | 391.6301 | -2.180912034 | 0 | e3 ubiquitin-protein ligase rnf181 ame: full=ring finger protein 181 |
| CB292505 | S22556310 | 1122 | 3280 | 959.7576 | 3834.468 | 1.998284548 | 0 | f-box family protein |
| CB292519 | S22556318 | 18 | 3 | 15.39718 | 3.507136 | -2.134301092 | 0 | ucrcs01\_04ce12\_g1 washington navel orange cold acclimated flavedo & albedo cdna library citrus sinensis cdna clone mrna |
| CB292559 | S22556340 | 287 | 1178 | 245.4995 | 1377.135 | 2.487878306 | 0 | serine threonine-protein kinase 1 6 |
| CB293231 | S22556716 | 3 | 15 | 2.566197 | 17.53568 | 2.772589504 | 0 | protein |
| CB293368 | S22556792 | 6 | 30 | 5.132394 | 35.07136 | 2.772589504 | 0 | ucrcs01\_06aa05\_g1 washington navel orange cold acclimated flavedo & albedo cdna library citrus sinensis cdna clone mrna |
| CB293734 | S22556991 | 343 | 1033 | 293.4018 | 1207.624 | 2.041221182 | 0 | ucrcs01\_06cd04\_g1 washington navel orange cold acclimated flavedo & albedo cdna library citrus sinensis cdna clone mrna |
| CB293991 | S22557136 | 851 | 1938 | 727.9445 | 2265.61 | 1.637998943 | 0 | upf0497 membrane protein 4 |
| CB290495 | S22557275 | 919 | 2331 | 786.1116 | 2725.044 | 1.793473647 | 0 | protein |
| CB290800 | S22557404 | 105 | 247 | 89.81689 | 288.7542 | 1.684783123 | 0 | �disease resistance protein rga3 ame: full=rga1-blb ame: full=blight resistance protein b149 |
| CB291196 | S22557573 | 13 | 2 | 11.12019 | 2.33809 | -2.249778309 | 0 | protein |
| CB291292 | S22557611 | 67 | 297 | 57.31173 | 347.2064 | 2.598891339 | 0 | circadian clock coupling factor zgt |
| CB293143 | S22558388 | 590 | 1550 | 504.6854 | 1812.02 | 1.844142765 | 0 | protein phosphatase 2c 16� |
| CB293236 | S22558423 | 39 | 337 | 33.36056 | 393.9682 | 3.561863971 | 0 | nac domain-containing protein 71� |
| CB293811 | S22558665 | 2125 | 5064 | 1817.723 | 5920.045 | 1.703475973 | 0 | ucrcs01\_06da01\_b1 washington navel orange cold acclimated flavedo & albedo cdna library citrus sinensis cdna clone mrna |
| CB304415 | S22558772 | 13 | 10 | 11.12019 | 11.69045 | 0.072149786 | 0 | flavedo0001\_ii \_e03 flavedo mature citrus sinensis cdna clone flavedo0001\_ii \_e03 3 mrna |
| CB304426 | S22558777 | 18 | 42 | 15.39718 | 49.0999 | 1.67305383 | 0 | e3 ubiquitin-protein ligase rnf34 ame: full=ring finger protein 34 ame: full=ring finger protein momo |
| CB304607 | S22558946 | 307 | 1079 | 262.6075 | 1261.4 | 2.264045713 | 0 | ethylene-responsive transcription factor erf061 |
| CB304637 | S22558976 | 1 | 11 | 0.855399 | 12.8595 | 3.910093028 | 0 | flavedo0001\_ \_g06 flavedo mature citrus sinensis cdna clone flavedo0001\_ \_g06 3 mrna |
| CB304738 | S22559035 | 32 | 2 | 27.37277 | 2.33809 | -3.549338591 | 0 | flavedo0002\_i \_b08 flavedo mature citrus sinensis cdna clone flavedo0002\_i \_b08 3 mrna |
| CB304782 | S22559079 | 75 | 207 | 64.15492 | 241.9924 | 1.915329676 | 0 | conserved hypothetical protein [Ricinus communis] |
| CB305067 | S22559334 | 11 | 18 | 9.409388 | 21.04281 | 1.161154792 | 0 | hypothetical protein [Vitis vinifera] |
| CB304396 | S22559432 | 2 | 0 | 1.710798 | 0 | -Inf | 0 | flavedo0001\_ii \_c02 flavedo mature citrus sinensis cdna clone flavedo0001\_ii \_c02 3 mrna |
| CB610680 | S22560093 | 6 | 17 | 5.132394 | 19.87377 | 1.95316175 | 0 | albedo0003\_ii \_c11 mature albedo citrus sinensis cdna clone albedo0003\_ii \_c11 5 mrna |
| CB610913 | S22560246 | 125 | 23 | 106.9249 | 26.88804 | -1.99156092 | 0 | albedo0002\_ii \_h06 mature albedo citrus sinensis cdna clone albedo0002\_ii \_h06 5 mrna |
| CB610836 | S22560579 | 2327 | 496 | 1990.513 | 579.8464 | -1.779397776 | 0 | albedo0002\_i \_h08 mature albedo citrus sinensis cdna clone albedo0002\_i \_h08 5 mrna |
| CB611027 | S22560606 | 4223 | 5148 | 3612.35 | 6018.245 | 0.736405215 | 0 | albedo0001\_ \_c05 mature albedo citrus sinensis cdna clone albedo0001\_ \_c05 5 mrna |
| CF417791 | S22561156 | 82 | 5 | 70.14271 | 5.845226 | -3.584962501 | 0 | cytochrome p450 716b1 ame: full=cytochrome p450 cypa1 |
| CF417159 | S22561371 | 20 | 4 | 17.10798 | 4.676181 | -1.871266686 | 0 | conserved hypothetical protein [Ricinus communis] |
| CF417718 | S22561564 | 4 | 14 | 3.421596 | 16.36663 | 2.258016331 | 0 | usda-fp\_115000-648 citrus sinensis: insect-damaged immature leaves stems from field collected valencia orange citrus sinensis cdna clone idfl-008\_d09 5 mrna |
| CF504270 | S22562119 | 159 | 332 | 136.0084 | 388.123 | 1.512817885 | 0 | nac domain-containing protein 90� |
| CF507764 | S22565613 | 476 | 3495 | 407.1699 | 4085.813 | 3.326920386 | 0 | usda-fp\_123000-265 immature ovaries from field-collected valencia sweet orange (citrus sinensis ( ) osbeck) citrus sinensis cdna clone mvf-60\_c08 5 mrna |
| CF508975 | S22566824 | 18 | 184 | 15.39718 | 215.1043 | 3.804298364 | 0 | u-box domain-containing protein 19 ame: full=plant u-box protein 19 |
| CF510057 | S22567906 | 17 | 2 | 14.54178 | 2.33809 | -2.636801432 | 0 | kinesin-4 ame: full=kinesin-like protein d |
| CF832354 | S22568325 | 1087 | 1119 | 929.8186 | 1308.162 | 0.492519505 | 0 | ucrcs02\_01l18\_f ruby orange ovary at anthesis cdna library citrus sinensis cdna clone mrna |
| CF833196 | S22568730 | 15 | 35 | 12.83098 | 40.91658 | 1.67305383 | 0 | ucrcs02\_03d03\_r ruby orange ovary at anthesis cdna library citrus sinensis cdna clone mrna |
| CF833296 | S22568777 | 97 | 8 | 82.9737 | 9.352362 | -3.149251433 | 0 | ucrcs02\_03g01\_r ruby orange ovary at anthesis cdna library citrus sinensis cdna clone mrna |
| CF832725 | S22570124 | 21 | 2 | 17.96338 | 2.33809 | -2.941656014 | 0 | cyclin-d3-1 ame: full=g1 s-specific cyclin-d3-1� |
| CF834184 | S22570834 | 141 | 32 | 120.6112 | 37.40945 | -1.688889943 | 0 | populus trichocarpa mrna |
| CF834198 | S22570840 | 196 | 1093 | 167.6582 | 1277.766 | 2.930029251 | 0 | ring-h2 finger protein atl3b ame: full=ring-h2 finger protein atl6 flags: precursor |
| CF834267 | S22570874 | 9 | 42 | 7.69859 | 49.0999 | 2.67305383 | 0 | lob domain-containing protein 38 ame: full=asymmetric leaves 2-like protein 40� |
| CF834421 | S22570947 | 139 | 15 | 118.9005 | 17.53568 | -2.761389068 | 0 | 3-ketoacyl- synthase 11� |
| CF835215 | S22571322 | 10 | 22 | 8.553989 | 25.71899 | 1.588164933 | 0 | cytochrome p450 71d9 ame: full=p450 cp3 |
| CF836416 | S22571911 | 382 | 2335 | 326.7624 | 2729.721 | 3.062439416 | 0 | ucrcs03\_02o21\_f washington navel orange shoot meristem cdna library citrus sinensis cdna clone mrna |
| CF836738 | S22572065 | 9 | 0 | 7.69859 | 0 | -Inf | 0 | retrovirus-related pol polyprotein from transposon tnt 1-94 includes: ame: full=protease includes: ame: full=reverse transcriptase includes: ame: full=endonuclease |
| CF837090 | S22572241 | 1711 | 301 | 1463.588 | 351.8826 | -2.056342959 | 0 | populus trichocarpa mrna |
| CF837114 | S22572251 | 18 | 2 | 15.39718 | 2.33809 | -2.719263592 | 0 | ucrcs03\_04c02\_f washington navel orange shoot meristem cdna library citrus sinensis cdna clone mrna |
| CF837352 | S22572364 | 261 | 591 | 223.2591 | 690.9057 | 1.629769733 | 0 | zinc finger |
| CF837958 | S22572665 | 1278 | 2551 | 1093.2 | 2982.234 | 1.447836472 | 0 | probable thylakoidal processing peptidase chloroplastic ame: full=signal peptidase i-2 flags: precursor |
| CF838109 | S22572738 | 8 | 16 | 6.843191 | 18.70472 | 1.450661409 | 0 | ucrcs03\_05m03\_r washington navel orange shoot meristem cdna library citrus sinensis cdna clone mrna |
| CF838234 | S22572800 | 1220 | 2711 | 1043.587 | 3169.282 | 1.602605374 | 0 | PREDICTED: hypothetical protein [Vitis vinifera] |
| CF838363 | S22572866 | 19 | 44 | 16.25258 | 51.43799 | 1.662165514 | 0 | ucrcs03\_06f18\_r washington navel orange shoot meristem cdna library citrus sinensis cdna clone mrna |
| CF838764 | S22573058 | 72 | 568 | 61.58872 | 664.0177 | 3.430483527 | 0 | �beta-d-xylosidase ame: full= z152 |
| CF838781 | S22573068 | 32 | 1 | 27.37277 | 1.169045 | -4.549338591 | 0 | ucrcs03\_06j10\_f washington navel orange shoot meristem cdna library citrus sinensis cdna clone mrna |
| CF838806 | S22573079 | 102 | 413 | 87.25069 | 482.8157 | 2.468234038 | 0 | cytochrome p450 94a1 ame: full=p450-dependent fatty acid omega-hydroxylase |
| CF838891 | S22573122 | 10 | 43 | 8.553989 | 50.26894 | 2.554998069 | 0 | pentatricopeptide repeat-containing protein at1g31920 |
| CF836098 | S22573518 | 190 | 20 | 162.5258 | 23.3809 | -2.797266104 | 0 | ice binding |
| CF837348 | S22574131 | 1108 | 2779 | 947.782 | 3248.777 | 1.777269362 | 0 | PREDICTED: hypothetical protein [Vitis vinifera] |
| CF837571 | S22574240 | 136 | 290 | 116.3343 | 339.0231 | 1.543107658 | 0 | u-box domain-containing protein 16 ame: full=plant u-box protein 16 |
| CF838077 | S22574489 | 5 | 17 | 4.276995 | 19.87377 | 2.216196155 | 0 | protein |
| CF838214 | S22574550 | 106 | 2 | 90.67229 | 2.33809 | -5.277259046 | 0 | disease resistance response protein 206 |
| CF838393 | S22574639 | 91 | 450 | 77.8413 | 526.0703 | 2.75664796 | 0 | endochitinase a� |
| CF838525 | S22574705 | 91 | 866 | 77.8413 | 1012.393 | 3.701089984 | 0 | protein |
| CV713002 | S22575386 | 8 | 28 | 6.843191 | 32.73327 | 2.258016331 | 0 | nicotiana tabacum mitochondrial complete genome |
| CV713105 | S22575445 | 10 | 42 | 8.553989 | 49.0999 | 2.521050737 | 0 | ucrcs08\_0002f09\_r parent washington navel orange callus cdna library ucrcs08-1 citrus sinensis cdna clone mrna |
| CV713606 | S22575728 | 18 | 2 | 15.39718 | 2.33809 | -2.719263592 | 0 | cellulose synthase a catalytic subunit 3� |
| CV714151 | S22576036 | 19 | 1 | 16.25258 | 1.169045 | -3.797266104 | 0 | cytokinin biosynthetic isopentenyltransferase |
| CV714710 | S22576355 | 182 | 1355 | 155.6826 | 1584.056 | 3.346943905 | 0 | protein |
| CV714851 | S22576436 | 2 | 1 | 1.710798 | 1.169045 | -0.549338591 | 0 | ucrcs08\_0004o10\_f parent washington navel orange callus cdna library ucrcs08-1 citrus sinensis cdna clone mrna |
| CV715341 | S22576716 | 43 | 165 | 36.78215 | 192.8925 | 2.390718869 | 0 | triacylglycerol lipase like protein |
| CV715674 | S22576905 | 17 | 35 | 14.54178 | 40.91658 | 1.492481585 | 0 | conserved hypothetical protein [Ricinus communis] |
| CV715748 | S22576949 | 1 | 1 | 0.855399 | 1.169045 | 0.450661409 | 0 | ucrcs08\_0006d19\_r parent washington navel orange callus cdna library ucrcs08-1 citrus sinensis cdna clone mrna |
| CV715903 | S22577038 | 35 | 4 | 29.93896 | 4.676181 | -2.678621608 | 0 | wound induced protein |
| CV715912 | S22577041 | 104 | 125 | 88.96149 | 146.1306 | 0.716005976 | 0 | ucrcs08\_0006i04\_r parent washington navel orange callus cdna library ucrcs08-1 citrus sinensis cdna clone mrna |
| CV716488 | S22577370 | 1301 | 297 | 1112.874 | 347.2064 | -1.680424717 | 0 | citrus unshiu gene for glycine-rich complete cds |
| CV716630 | S22577452 | 35 | 8 | 29.93896 | 9.352362 | -1.678621608 | 0 | methionine s-methyltransferase ame: full= et:met s-methyltransferase |
| CV716897 | S22577603 | 172 | 357 | 147.1286 | 417.3491 | 1.504176918 | 0 | ucrcs08\_0008a16\_f parent washington navel orange callus cdna library ucrcs08-1 citrus sinensis cdna clone mrna |
| CV718169 | S22578327 | 9 | 34 | 7.69859 | 39.74754 | 2.368199249 | 0 | probable lrr receptor-like serine threonine-protein kinase at3g47570 flags: precursor |
| CV718209 | S22578347 | 391 | 1530 | 334.461 | 1788.639 | 2.418952549 | 0 | phosphatidylglycerol specific phospholipase c |
| CV718293 | S22578395 | 8 | 22 | 6.843191 | 25.71899 | 1.910093028 | 0 | fragaria vesca americana clone fosmid complete sequence |
| CV718817 | S22578697 | 973 | 3233 | 832.3032 | 3779.523 | 2.183023206 | 0 | anthocyanin 3 -o-beta-glucosyltransferase� |
| CV719693 | S22579191 | 1 | 0 | 0.855399 | 0 | -Inf | 0 | ucrcs08\_0012f04\_f parent washington navel orange callus cdna library ucrcs08-1 citrus sinensis cdna clone mrna |
| CV719989 | S22579361 | 88 | 17 | 75.27511 | 19.87377 | -1.921307368 | 0 | protein |
| CV712647 | S22579680 | 2035 | 1080 | 1740.737 | 1262.569 | -0.463336073 | 0 | nucleosome chromatin assembly factor group |
| CV713065 | S22579858 | 331 | 3069 | 283.137 | 3587.8 | 3.663526933 | 0 | taxadien-5-alpha-ol o-acetyltransferase ame: full=taxa-4 -dien-5alpha-ol-o-acetyltransferase� |
| CV713122 | S22579883 | 57 | 1 | 48.75774 | 1.169045 | -5.382228605 | 0 | glucan endo- -beta-glucosidase ame: full=(1- |
| CV713559 | S22580072 | 17 | 63 | 14.54178 | 73.64985 | 2.340478491 | 0 | hypothetical protein NitaMp116 [Nicotiana tabacum] |
| CV713629 | S22580102 | 3 | 3 | 2.566197 | 3.507136 | 0.450661409 | 0 | ucrcs08\_0003b09\_r parent washington navel orange callus cdna library ucrcs08-1 citrus sinensis cdna clone mrna |
| CV714551 | S22580495 | 42 | 8 | 35.92675 | 9.352362 | -1.941656014 | 0 | glucan endo- -beta-glucosidase 9 ame: full=(1- |
| CV714944 | S22580664 | 266 | 979 | 227.5361 | 1144.495 | 2.330544023 | 0 | abc transporter g family member 39� |
| CV715265 | S22580801 | 11 | 1 | 9.409388 | 1.169045 | -3.00877021 | 0 | seed maturation protein |
| CV716021 | S22581123 | 7 | 13 | 5.987792 | 15.19759 | 1.343746205 | 0 | ucrcs08\_0006l01\_r parent washington navel orange callus cdna library ucrcs08-1 citrus sinensis cdna clone mrna |
| CV716258 | S22581222 | 81 | 215 | 69.28731 | 251.3447 | 1.859004256 | 0 | ucrcs08\_0007a15\_f parent washington navel orange callus cdna library ucrcs08-1 citrus sinensis cdna clone mrna |
| CV716985 | S22581531 | 2 | 8 | 1.710798 | 9.352362 | 2.450661409 | 0 | demeter-like protein 2 |
| CV717112 | S22581586 | 22 | 46 | 18.81878 | 53.77608 | 1.514791746 | 0 | beta vulgaris vulgaris mitochondrial complete genome |
| CV717128 | S22581594 | 262 | 33 | 224.1145 | 38.57849 | -2.538367473 | 0 | peptidase m50 |
| CV718150 | S22582030 | 3 | 26 | 2.566197 | 30.39518 | 3.566138626 | 0 | protein |
| CV718596 | S22582220 | 34 | 3 | 29.08356 | 3.507136 | -3.051838932 | 0 | protein |
| CV718921 | S22582359 | 41 | 89 | 35.07136 | 104.045 | 1.568842835 | 0 | non-symbiotic hemoglobin 1 ame: full=medsa glb1 |
| CV719351 | S22582541 | 516 | 574 | 441.3858 | 671.0319 | 0.60434108 | 0 | citrus maxima clone m29-31b2-1 ctvv resistance partial sequence |
| CV719508 | S22582610 | 24 | 5 | 20.52957 | 5.845226 | -1.812372997 | 0 | conserved hypothetical protein [Ricinus communis] |
| CV719618 | S22582656 | 49 | 2 | 41.91455 | 2.33809 | -4.164048435 | 0 | ucrcs08\_0012d12\_r parent washington navel orange callus cdna library ucrcs08-1 citrus sinensis cdna clone mrna |
| CV719871 | S22582765 | 5 | 20 | 4.276995 | 23.3809 | 2.450661409 | 0 | transcription factor bhlh113 ame: full=transcription factor en 61 ame: full=bhlh transcription factor bhlh113 ame: full=basic helix-loop-helix protein 113� |
| CV884611 | S22583081 | 712 | 1791 | 609.044 | 2093.76 | 1.7814776 | 0 | 4-coumarate-- ligase-like 5 ame: full=4-coumarate-- ligase isoform 9� |
| CV884948 | S22583236 | 171 | 446 | 146.2732 | 521.3942 | 1.833708794 | 0 | ucrcs04\_2\_011e10\_t3 ruby orange developing flower cdna library ucrcs04-ucr citrus sinensis cdna clone mrna |
| CV885133 | S22583323 | 280 | 712 | 239.5117 | 832.3602 | 1.797111823 | 0 | protein |
| CV885563 | S22583523 | 447 | 1440 | 382.3633 | 1683.425 | 2.138383484 | 0 | protein notum homolog flags: precursor |
| CV886253 | S22583865 | 53 | 248 | 45.33614 | 289.9232 | 2.676937265 | 0 | linalool chloroplastic flags: precursor |
| CV886525 | S22583988 | 211 | 621 | 180.4892 | 725.9771 | 2.008011679 | 0 | zinc finger protein constans-like 10 |
| CV886691 | S22584063 | 53 | 7 | 45.33614 | 8.183316 | -2.469904123 | 0 | methyltransferase-like protein 13 |
| CV886834 | S22584129 | 15 | 1 | 12.83098 | 1.169045 | -3.456229187 | 0 | probable rhamnose biosynthetic enzyme 3 |
| CV885023 | S22584821 | 289 | 531 | 247.2103 | 620.763 | 1.328303777 | 0 | protein |
| CV885307 | S22584971 | 4 | 17 | 3.421596 | 19.87377 | 2.53812425 | 0 | vitis vinifera contig whole genome shotgun sequence |
| CV885347 | S22584989 | 384 | 88 | 328.4732 | 102.876 | -1.674869473 | 0 | ucrcs04\_2\_015b08\_t7 ruby orange developing flower cdna library ucrcs04-ucr citrus sinensis cdna clone mrna |
| CV885396 | S22585018 | 355 | 20 | 303.6666 | 23.3809 | -3.69908571 | 0 | protein |
| CV887374 | S22585048 | 0 | 29 | 0 | 33.90231 | Inf | 0 | ucrcs04\_2\_030h07\_t3 ruby orange developing flower cdna library ucrcs04-ucr citrus sinensis cdna clone mrna |
| CV886254 | S22585416 | 295 | 639 | 252.3427 | 747.0199 | 1.565762386 | 0 | predicted protein [Populus trichocarpa] |
| CV998067 | S22586071 | 2461 | 5763 | 2105.137 | 6737.207 | 1.678236771 | 0 | isoflavone 2 -hydroxylase ame: full=cytochrome p450 81e1 ame: full=p450 91a4 ame: full=cyp ge-3 |
| CX043477 | S22586216 | 55 | 335 | 47.04694 | 391.6301 | 3.057318981 | 0 | laccase-8 ame: full=benzenediol:oxygen oxidoreductase 8 ame: full=urishiol oxidase 8 ame: full=diphenol oxidase 8 flags: precursor |
| CX043703 | S22586323 | 207 | 798 | 177.0676 | 932.8981 | 2.397419388 | 0 | acidic endochitinase flags: precursor |
| CX043988 | S22586454 | 56 | 5 | 47.90234 | 5.845226 | -3.034765418 | 0 | meiosis 5 |
| CX044195 | S22586549 | 760 | 1527 | 650.1032 | 1785.132 | 1.457290147 | 0 | predicted protein [Populus trichocarpa] |
| CX044934 | S22586893 | 4 | 13 | 3.421596 | 15.19759 | 2.151101127 | 0 | ring-h2 finger protein atl1k ame: full=ring-h2 finger protein atl10 |
| CX045546 | S22587179 | 1241 | 2784 | 1061.55 | 3254.622 | 1.616317505 | 0 | brassinosteroid lrr receptor kinase ame: full=tbri1 ame: full=altered brassinolide sensitivity 1 ame: full=systemin receptor sr160 flags: precursor |
| CX045547 | S22587180 | 474 | 1214 | 405.4591 | 1419.221 | 1.807470866 | 0 | lrr receptor-like serine threonine-protein kinase rch1 ame: full=protein root clavata-homolog1 1 flags: precursor |
| CX046042 | S22587418 | 2218 | 5457 | 1897.275 | 6379.48 | 1.749510087 | 0 | probable inorganic phosphate transporter 1-3� |
| CX046065 | S22587428 | 194 | 2045 | 165.9474 | 2390.697 | 3.848633695 | 0 | reticuline oxidase-like protein flags: precursor |
| CX043799 | S22588043 | 20 | 65 | 17.10798 | 75.98794 | 2.151101127 | 0 | ethylene-responsive transcription factor 1b� |
| CX044536 | S22588439 | 129 | 31 | 110.3465 | 36.2404 | -1.606369536 | 0 | populus trichocarpa mrna |
| CX044628 | S22588487 | 0 | 30 | 0 | 35.07136 | Inf | 0 | 3 -n-debenzoyl-2 -deoxytaxol n-benzoyltransferase� |
| CX045330 | S22588860 | 7 | 1894 | 5.987792 | 2214.172 | 8.530527102 | 0 | ucrcs07\_22b08\_b parent washington navel orange thrip-challenged flavedo cdna library ucrcs07 citrus sinensis cdna clone ucrcs07-22b08-d16-1- mrna |
| CX045334 | S22588865 | 31 | 128 | 26.51737 | 149.6378 | 2.496465099 | 0 | jasmonate o-methyltransferase ame: full=s-adenosyl-l-methionine:jasmonic acid carboxyl methyltransferase ame: full=floral nectary-specific protein 1 |
| CX045473 | S22588938 | 746 | 1449 | 638.1276 | 1693.946 | 1.408471468 | 0 | protein |
| CX045842 | S22589131 | 410 | 29 | 350.7136 | 33.90231 | -3.370837695 | 0 | unknown protein [Arabidopsis thaliana] |
| CX046356 | S22589397 | 242 | 31 | 207.0065 | 36.2404 | -2.514005518 | 0 | receptor protein kinase clavata1 flags: precursor |
| CX046518 | S22589484 | 178 | 20 | 152.261 | 23.3809 | -2.703143927 | 0 | fatty acyl- reductase 3 ame: full=protein eceriferum 4 |
| CX046529 | S22589490 | 4 | 16 | 3.421596 | 18.70472 | 2.450661409 | 0 | ucrcs07\_8f04\_b parent washington navel orange thrip-challenged flavedo cdna library ucrcs07 citrus sinensis cdna clone ucrcs07-8f04-k8-1- mrna |
| CX046605 | S22589528 | 27 | 309 | 23.09577 | 361.235 | 3.967236935 | 0 | uncharacterized plant-specific domain tigr01615 family expressed |
| CX046670 | S22589560 | 22 | 49 | 18.81878 | 57.28321 | 1.605939634 | 0 | oxygen-evolving enhancer protein 3- chloroplastic� |
| CX046862 | S22589651 | 279 | 746 | 238.6563 | 872.1077 | 1.869571917 | 0 | ribulose bisphosphate carboxylase large chain� |
| CX047081 | S22589751 | 1 | 10 | 0.855399 | 11.69045 | 3.772589504 | 0 | retrotransposon ty1-copia subclass |
| CX047170 | S22589791 | 5 | 15 | 4.276995 | 17.53568 | 2.03562391 | 0 | nicotiana tabacum mitochondrial complete genome |
| CX047235 | S22589821 | 28 | 58 | 23.95117 | 67.80462 | 1.501287482 | 0 | er lumen protein retaining receptor ame: full=hdel receptor ame: full=pgp169-12 |
| CX047303 | S22589854 | 18 | 2 | 15.39718 | 2.33809 | -2.719263592 | 0 | ucrcs09\_13d02\_b ruby orange developing seed cdna library ucrcs09 citrus sinensis cdna clone ucrcs09-13d02-g4-1- mrna |
| CX047329 | S22589866 | 7 | 25 | 5.987792 | 29.22613 | 2.287162677 | 0 | ucrcs09\_13e05\_b ruby orange developing seed cdna library ucrcs09 citrus sinensis cdna clone ucrcs09-13e05-i10-1- mrna |
| CX047459 | S22589926 | 5 | 12 | 4.276995 | 14.02854 | 1.713695815 | 0 | protein hothead ame: full=protein adhesion of calyx edges flags: precursor |
| CX048448 | S22590387 | 6 | 39 | 5.132394 | 45.59276 | 3.151101127 | 0 | salt tolerance protein |
| CX048690 | S22590496 | 44 | 88 | 37.63755 | 102.876 | 1.450661409 | 0 | 60s ribosomal protein mitochondrial |
| CX049094 | S22590683 | 473 | 67 | 404.6037 | 78.32603 | -2.368945774 | 0 | �domain-containing gpi-anchored protein 1 flags: precursor |
| CX049732 | S22590978 | 53 | 110 | 45.33614 | 128.595 | 1.504100668 | 0 | protein |
| CX049939 | S22591074 | 13 | 46 | 11.12019 | 53.77608 | 2.273783647 | 0 | predicted protein [Populus trichocarpa] |
| CX049951 | S22591079 | 4 | 14 | 3.421596 | 16.36663 | 2.258016331 | 0 | ucrcs09\_2e11\_b ruby orange developing seed cdna library ucrcs09 citrus sinensis cdna clone ucrcs09-2e11-i22-1- mrna |
| CX049956 | S22591084 | 5 | 29 | 4.276995 | 33.90231 | 2.986714309 | 0 | vitis vinifera contig whole genome shotgun sequence |
| CX050112 | S22591158 | 1261 | 260 | 1078.658 | 303.9518 | -1.827323338 | 0 | heat shock 70 kda protein |
| CX050437 | S22591308 | 121 | 246 | 103.5033 | 287.5851 | 1.474312677 | 0 | �cytochrome c biosynthesis ccmc-like mitochondrial protein ame: full=abc transporter i family member 3� |
| CX050471 | S22591321 | 2415 | 1547 | 2065.788 | 1808.513 | -0.191888583 | 0 | nad h-quinone oxidoreductase subunit chloroplastic ame: full=nad h dehydrogenase subunit j ame: full=nadh-plastoquinone oxidoreductase subunit j |
| CX050523 | S22591345 | 59 | 115 | 50.46854 | 134.4402 | 1.413508411 | 0 | nad dependent epimerase dehydratase family protein |
| CX051396 | S22591749 | 13 | 26 | 11.12019 | 30.39518 | 1.450661409 | 0 | ac009360\_9this gene is cut |
| CX051758 | S22591917 | 23 | 1 | 19.67418 | 1.169045 | -4.072900547 | 0 | protein |
| CX051823 | S22591947 | 7 | 13 | 5.987792 | 15.19759 | 1.343746205 | 0 | ucrcs09\_42c03\_b ruby orange developing seed cdna library ucrcs09 citrus sinensis cdna clone ucrcs09-42c03-f5-1- mrna |
| CX051996 | S22592029 | 84 | 146 | 71.85351 | 170.6806 | 1.248168545 | 0 | PREDICTED: hypothetical protein [Vitis vinifera] |
| CX052345 | S22592189 | 15 | 43 | 12.83098 | 50.26894 | 1.970035568 | 0 | PREDICTED: hypothetical protein [Vitis vinifera] |
| CX052490 | S22592257 | 827 | 1905 | 707.4149 | 2227.031 | 1.654493172 | 0 | acrs-like protein |
| CX052699 | S22592354 | 87 | 12 | 74.41971 | 14.02854 | -2.407319586 | 0 | probable flavin-containing monooxygenase 1 |
| CX053113 | S22592544 | 109 | 228 | 93.23848 | 266.5423 | 1.515367098 | 0 | cytochrome c oxidase subunit 3 ame: full=cytochrome c oxidase polypeptide iii |
| CX054039 | S22592993 | 4 | 70 | 3.421596 | 81.83316 | 4.579944426 | 0 | dna binding protein |
| CX047037 | S22593159 | 4 | 4 | 3.421596 | 4.676181 | 0.450661409 | 0 | protein |
| CX047321 | S22593311 | 18 | 4 | 15.39718 | 4.676181 | -1.719263592 | 0 | phosphoribosylanthranilate transferase |
| CX047721 | S22593526 | 401 | 3051 | 343.015 | 3566.757 | 3.378269447 | 0 | potassium channel tetramerization domain-containing |
| CX048596 | S22593994 | 8 | 16 | 6.843191 | 18.70472 | 1.450661409 | 0 | polyprotein [Lycopersicon esculentum] |
| CX048969 | S22594194 | 35 | 90 | 29.93896 | 105.2141 | 1.813231488 | 0 | arabidopsis thaliana mitochondrial genome |
| CX049428 | S22594442 | 41 | 272 | 35.07136 | 317.9803 | 3.180572246 | 0 | ucrcs09\_27e07\_b ruby orange developing seed cdna library ucrcs09 citrus sinensis cdna clone ucrcs09-27e07-j14-1- mrna |
| CX049508 | S22594486 | 42 | 4 | 35.92675 | 4.676181 | -2.941656014 | 0 | ucrcs09\_28a02\_b ruby orange developing seed cdna library ucrcs09 citrus sinensis cdna clone ucrcs09-28a02-a3-1- mrna |
| CX049781 | S22594633 | 291 | 59 | 248.9211 | 68.97367 | -1.851570885 | 0 | transcription factor |
| CX049845 | S22594667 | 2 | 14 | 1.710798 | 16.36663 | 3.258016331 | 0 | protein |
| CX050002 | S22594743 | 9 | 29 | 7.69859 | 33.90231 | 2.138717403 | 0 | nicotiana tabacum mitochondrial complete genome |
| CX050195 | S22594852 | 38 | 105 | 32.50516 | 122.7497 | 1.916979413 | 0 | protein |
| CX050713 | S22595131 | 48 | 102 | 41.05915 | 119.2426 | 1.53812425 | 0 | lea2\_cicarlate embryogenesis abundant protein 2 ( -2) |
| CX051193 | S22595387 | 31 | 98 | 26.51737 | 114.5664 | 2.111174943 | 0 | glycogenin-1 |
| CX051392 | S22595492 | 37 | 220 | 31.64976 | 257.1899 | 3.022567757 | 0 | peroxisomal membrane protein 11b ame: full=peroxin-11b� |
| CX051519 | S22595562 | 429 | 72 | 366.9661 | 84.17125 | -2.124247427 | 0 | 3-ketoacyl- synthase 10� |
| CX051582 | S22595596 | 21 | 2 | 17.96338 | 2.33809 | -2.941656014 | 0 | ucrcs09\_40g10\_b ruby orange developing seed cdna library ucrcs09 citrus sinensis cdna clone ucrcs09-40g10-m19-1- mrna |
| CX051691 | S22595656 | 217 | 27 | 185.6216 | 31.56422 | -2.556002321 | 0 | xyloglucan glycosyltransferase 4 ame: full=xyloglucan synthase 4 ame: full=cellulose synthase-like protein c4� |
| CX051790 | S22595708 | 20 | 2 | 17.10798 | 2.33809 | -2.871266686 | 0 | ucrcs09\_6c06\_b ruby orange developing seed cdna library ucrcs09 citrus sinensis cdna clone ucrcs09-6c06-f11-1- mrna |
| CX051949 | S22595794 | 622 | 120 | 532.0581 | 140.2854 | -1.923218766 | 0 | probable lrr receptor-like serine threonine-protein kinase at1g14390 flags: precursor |
| CX052259 | S22595961 | 6 | 20 | 5.132394 | 23.3809 | 2.187627003 | 0 | protein ycf2 |
| CX053181 | S22596455 | 35 | 63 | 29.93896 | 73.64985 | 1.298658316 | 0 | conserved hypothetical protein [Ricinus communis] |
| CX053694 | S22596722 | 30 | 61 | 25.66197 | 71.31176 | 1.474508151 | 0 | carica papaya complete genome |
| CX053848 | S22596803 | 298 | 586 | 254.9089 | 685.0605 | 1.426249743 | 0 | uncharacterized rna-binding protein |
| CX053885 | S22596821 | 15 | 1 | 12.83098 | 1.169045 | -3.456229187 | 0 | auxin transporter-like protein 5 ame: full=aux1-like protein 5 ame: full= 5 |
| CX053938 | S22596849 | 198 | 428 | 169.369 | 500.3513 | 1.562771775 | 0 | uncharacterized mitochondrial protein g00660 ame: full=orf149 |
| CX054032 | S22596897 | 13 | 27 | 11.12019 | 31.56422 | 1.505109193 | 0 | ucrcs09\_9g11\_b ruby orange developing seed cdna library ucrcs09 citrus sinensis cdna clone ucrcs09-9g11-m22-1- mrna |
| CX069388 | S22596922 | 3 | 12 | 2.566197 | 14.02854 | 2.450661409 | 0 | vitis vinifera contig whole genome shotgun sequence |
| CX069789 | S22597106 | 33 | 3 | 28.22816 | 3.507136 | -3.00877021 | 0 | retrovirus-related pol polyprotein from transposon tnt 1-94 includes: ame: full=protease includes: ame: full=reverse transcriptase includes: ame: full=endonuclease |
| CX070091 | S22597247 | 103 | 8 | 88.10609 | 9.352362 | -3.235839118 | 0 | snakin-2 flags: precursor |
| CX071153 | S22597738 | 12 | 1 | 10.26479 | 1.169045 | -3.134301092 | 0 | unnamed protein product [Vitis vinifera] |
| CX071698 | S22597989 | 875 | 98 | 748.4741 | 114.5664 | -2.707767954 | 0 | ucrcs08\_23d06\_g parent washington navel orange callus cdna library ucrcs08-2 citrus sinensis cdna clone ucrcs08-23d06-h11-1- mrna |
| CX072377 | S22598304 | 15 | 29 | 12.83098 | 33.90231 | 1.401751809 | 0 | beta vulgaris vulgaris mitochondrial complete genome |
| CX072481 | S22598352 | 101 | 914 | 86.39529 | 1068.507 | 3.628500281 | 0 | ucrcs08\_28d01\_b parent washington navel orange callus cdna library ucrcs08-2 citrus sinensis cdna clone ucrcs08-28d01-h2-1- mrna |
| CX073325 | S22598744 | 38 | 6 | 32.50516 | 7.014271 | -2.212303604 | 0 | transcription factor tga2 ame: full=hbp-1b homolog� |
| CX073729 | S22598931 | 76 | 18 | 65.01032 | 21.04281 | -1.627341103 | 0 | probable receptor protein kinase tmk1 flags: precursor |
| CX074287 | S22599188 | 89 | 599 | 76.1305 | 700.2581 | 3.201340171 | 0 | ring-h2 finger protein atl1m |
| CX074987 | S22599512 | 164 | 13 | 140.2854 | 15.19759 | -3.206450877 | 0 | chloroplast-targeted copper |
| CX075403 | S22599704 | 209 | 306 | 178.7784 | 357.7278 | 1.00069012 | 0 | ethylene-responsive transcription factor 1 ame: full=ethylene-responsive element-binding factor 1� |
| CX075508 | S22599753 | 2854 | 142 | 2441.309 | 166.0044 | -3.878361091 | 0 | ethylene-responsive transcription factor erf012 |
| CX076196 | S22600070 | 3 | 0 | 2.566197 | 0 | -Inf | 0 | ucrcs08\_49d12\_b parent washington navel orange callus cdna library ucrcs08-2 citrus sinensis cdna clone ucrcs08-49d12-g23-1- mrna |
| CX077188 | S22600532 | 16 | 3 | 13.68638 | 3.507136 | -1.96437609 | 0 | ucrcs08\_55a08\_b parent washington navel orange callus cdna library ucrcs08-2 citrus sinensis cdna clone ucrcs08-55a08-b15-1- mrna |
| CX077201 | S22600538 | 123 | 345 | 105.2141 | 403.3206 | 1.938599455 | 0 | ucrcs08\_55b03\_b parent washington navel orange callus cdna library ucrcs08-2 citrus sinensis cdna clone ucrcs08-55b03-d5-1- mrna |
| CX077288 | S22600576 | 23 | 302 | 19.67418 | 353.0516 | 4.165504192 | 0 | bap2 (bon association protein 2) |
| CX077578 | S22600712 | 36 | 115 | 30.79436 | 134.4402 | 2.126226459 | 0 | hypothetical protein NitaMp078 [Nicotiana tabacum] |
| CX077877 | S22600852 | 151 | 15 | 129.1652 | 17.53568 | -2.880852735 | 0 | u-box domain-containing protein 11 ame: full=plant u-box protein 11 |
| CX077928 | S22600876 | 10 | 35 | 8.553989 | 40.91658 | 2.258016331 | 0 | conserved hypothetical protein [Ricinus communis] |
| CX070380 | S22601769 | 30 | 59 | 25.66197 | 68.97367 | 1.426413863 | 0 | nadh-plastoquinone oxidoreductase subunit 1 |
| CX070848 | S22602021 | 22 | 46 | 18.81878 | 53.77608 | 1.514791746 | 0 | probable glutamate carboxypeptidase 2 ame: full=probable glutamate carboxypeptidase ii |
| CX071017 | S22602114 | 0 | 13 | 0 | 15.19759 | Inf | 0 | protein abscisic acid-insensitive 5 ame: full=dc3 promoter-binding factor 1� |
| CX072640 | S22602985 | 14 | 2 | 11.97558 | 2.33809 | -2.356693513 | 0 | ucrcs08\_29c12\_g parent washington navel orange callus cdna library ucrcs08-2 citrus sinensis cdna clone ucrcs08-29c12-e23-1- mrna |
| CX073187 | S22603277 | 48 | 111 | 41.05915 | 129.764 | 1.660114775 | 0 | guanine nucleotide-binding protein alpha-1 subunit� |
| CX073773 | S22603590 | 267 | 865 | 228.3915 | 1011.224 | 2.1465218 | 0 | probable protein phosphatase 2c 2� |
| CX074526 | S22603998 | 43 | 8 | 36.78215 | 9.352362 | -1.975603346 | 0 | armadillo repeat-containing kinesin-like protein 1 ame: full=protein morphogenesis of root hair 2 |
| CX074538 | S22604004 | 234 | 22 | 200.1633 | 25.71899 | -2.960271692 | 0 | mlo-like protein 11� |
| CX074829 | S22604160 | 80 | 6 | 68.43191 | 7.014271 | -3.286304185 | 0 | afadin- and alpha-actinin-binding |
| CX074986 | S22604245 | 234 | 19 | 200.1633 | 22.21186 | -3.171775797 | 0 | chloroplast-targeted copper |
| CX075279 | S22604401 | 1031 | 1021 | 881.9163 | 1193.595 | 0.436599943 | 0 | cyclin-b1-2 ame: full=g2 mitotic-specific cyclin-b1-2� |
| CX075961 | S22604770 | 34 | 64 | 29.08356 | 74.81889 | 1.363198568 | 0 | ucrcs08\_48a02\_b parent washington navel orange callus cdna library ucrcs08-2 citrus sinensis cdna clone ucrcs08-48a02-b4-1- mrna |
| CX076036 | S22604809 | 42 | 387 | 35.92675 | 452.4205 | 3.654533742 | 0 | dehydration-responsive element-binding protein 1c� |
| CX076101 | S22604844 | 8 | 80 | 6.843191 | 93.52362 | 3.772589504 | 0 | embryogenic cell protein 40� |
| CX076324 | S22604965 | 17 | 4 | 14.54178 | 4.676181 | -1.636801432 | 0 | coleoptile phototropism protein 1 ame: full=non-phototropic hypocotyl 3-like protein� |
| CX076597 | S22605110 | 172 | 35 | 147.1286 | 40.91658 | -1.846320329 | 0 | protein |
| CX076751 | S22605192 | 4 | 18 | 3.421596 | 21.04281 | 2.62058641 | 0 | beta vulgaris vulgaris mitochondrial complete genome |
| CX077352 | S22605517 | 10 | 2 | 8.553989 | 2.33809 | -1.871266686 | 0 | ucrcs08\_56a12\_g parent washington navel orange callus cdna library ucrcs08-2 citrus sinensis cdna clone ucrcs08-56a12-a23-1- mrna |
| CX077854 | S22605785 | 36 | 6 | 30.79436 | 7.014271 | -2.134301092 | 0 | ucrcs08\_5d09\_g parent washington navel orange callus cdna library ucrcs08-2 citrus sinensis cdna clone ucrcs08-5d09-g17-1- mrna |
| CX077884 | S22605796 | 745 | 98 | 637.2722 | 114.5664 | -2.475725362 | 0 | receptor-like protein kinase hsl1 ame: full=protein haesa-like1 flags: precursor |
| AF441124 | S22606170 | 11869 | 2349 | 10152.73 | 2746.087 | -1.886418361 | 0 | (+)-delta-cadinene synthase isozyme c2� |
| AY243478 | S22606176 | 4086 | 11642 | 3495.16 | 13610.02 | 1.961239223 | 0 | allene oxide chloroplastic ame: full=cytochrome p450 74a ame: full=hydroperoxide dehydrase flags: precursor |
| AY242385 | S22606177 | 4264 | 10340 | 3647.421 | 12087.93 | 1.728618251 | 0 | allene oxide chloroplastic ame: full=cytochrome p450 74a ame: full=hydroperoxide dehydrase flags: precursor |
| AY040710 | S22606179 | 549 | 97 | 469.614 | 113.3974 | -2.050088088 | 0 | pectinesterase 3� |
| AY029198 | S22606183 | 326 | 797 | 278.86 | 931.729 | 1.740369169 | 0 | beta-galactosidase ame: full=acid beta-galactosidase� |
| AF321533 | S22606192 | 828 | 11070 | 708.2703 | 12941.33 | 4.191542053 | 0 | 1-aminocyclopropane-1-carboxylate oxidase� |
| U82976 | S22606218 | 19993 | 2690 | 17101.99 | 3144.732 | -2.443155481 | 0 | pectinesterase 1� |
| Y08618 | S22606220 | 4283 | 816 | 3663.674 | 953.9409 | -1.941319211 | 0 | polygalacturonase inhibitor ame: full=polygalacturonase-inhibiting protein� |
| CX675318 | S23016762 | 2 | 28 | 1.710798 | 32.73327 | 4.258016331 | 0 | PREDICTED: hypothetical protein [Vitis vinifera] |
| CX675331 | S23016768 | 58 | 86 | 49.61314 | 100.5379 | 1.018945169 | 0 | ucrcs08\_61e06\_b parent washington navel orange callus cdna library ucrcs08-3 citrus sinensis cdna clone ucrcs08-61e06-j11-1- mrna |
| CX675379 | S23016788 | 102 | 230 | 87.25069 | 268.8804 | 1.623726118 | 0 | hypothetical protein [Vitis vinifera] |
| CX675444 | S23016818 | 18 | 4 | 15.39718 | 4.676181 | -1.719263592 | 0 | structural constituent of ribosome |
| CX675523 | S23016855 | 2721 | 384 | 2327.54 | 448.9134 | -2.374297332 | 0 | protein |
| CX675813 | S23016991 | 47 | 2 | 40.20375 | 2.33809 | -4.103927443 | 0 | btb poz domain-containing protein at5g48800 |
| CX676159 | S23017148 | 2 | 0 | 1.710798 | 0 | -Inf | 0 | transcription factor bhlh96 ame: full=transcription factor en 15 ame: full=bhlh transcription factor bhlh096 ame: full=basic helix-loop-helix protein 96� |
| CX676293 | S23017212 | 11 | 52 | 9.409388 | 60.79035 | 2.691669509 | 0 | ucrcs08\_68e11\_b parent washington navel orange callus cdna library ucrcs08-3 citrus sinensis cdna clone ucrcs08-68e11-i22-1- mrna |
| CX676294 | S23017213 | 2 | 24 | 1.710798 | 28.05708 | 4.03562391 | 0 | ucrcs08\_68e11\_g parent washington navel orange callus cdna library ucrcs08-3 citrus sinensis cdna clone ucrcs08-68e11-i22-1- mrna |
| CX675222 | S23017478 | 8 | 20 | 6.843191 | 23.3809 | 1.772589504 | 0 | conserved hypothetical protein [Ricinus communis] |
| CX675374 | S23017571 | 7 | 27 | 5.987792 | 31.56422 | 2.398193989 | 0 | citrus unshiu 5 flanking region of d-limonene synthase |
| CX675450 | S23017612 | 99 | 17 | 84.68449 | 19.87377 | -2.09123237 | 0 | predicted protein [Populus trichocarpa] |
| CX675554 | S23017668 | 37 | 95 | 31.64976 | 111.0593 | 1.811063652 | 0 | ras-gtpase-activating protein-binding |
| CX675684 | S23017738 | 9 | 0 | 7.69859 | 0 | -Inf | 0 | ethylene-responsive transcription factor wri1 ame: full=protein wrinkled 1 ame: full=protein activator of sporamin::luc 1 |
| CX675713 | S23017754 | 5 | 10 | 4.276995 | 11.69045 | 1.450661409 | 0 | vitis vinifera contig whole genome shotgun sequence |
| CX676104 | S23017965 | 6 | 12 | 5.132394 | 14.02854 | 1.450661409 | 0 | ucrcs08\_67b06\_b parent washington navel orange callus cdna library ucrcs08-3 citrus sinensis cdna clone ucrcs08-67b06-c11-1- mrna |
| CX676116 | S23017971 | 22 | 48 | 18.81878 | 56.11417 | 1.576192291 | 0 | 1-aminocyclopropane-1-carboxylate oxidase 1� |
| CX676285 | S23018062 | 515 | 1681 | 440.5304 | 1965.165 | 2.157336796 | 0 | conserved hypothetical protein [Ricinus communis] |
| CX676339 | S23018092 | 10 | 0 | 8.553989 | 0 | -Inf | 0 | ucrcs08\_68h04\_b parent washington navel orange callus cdna library ucrcs08-3 citrus sinensis cdna clone ucrcs08-68h04-o8-1- mrna |
| CX671705 | S23018431 | 8 | 24 | 6.843191 | 28.05708 | 2.03562391 | 0 | ucrcs10\_12f08\_b madame vinous sweet orange multiple pathogen-infected cdna library ucrcs10 citrus sinensis cdna clone ucrcs10-12f08-l16- mrna |
| CX672039 | S23018591 | 84 | 9 | 71.85351 | 10.52141 | -2.771731012 | 0 | subtilisin-like protease ame: full=cucumisin-like serine protease flags: precursor |
| CX672588 | S23018860 | 11 | 1 | 9.409388 | 1.169045 | -3.00877021 | 0 | protein |
| CX672782 | S23018952 | 9 | 25 | 7.69859 | 29.22613 | 1.924592597 | 0 | ucrcs10\_19c10\_b madame vinous sweet orange multiple pathogen-infected cdna library ucrcs10 citrus sinensis cdna clone ucrcs10-19c10-f19- mrna |
| CX672872 | S23018994 | 101 | 207 | 86.39529 | 241.9924 | 1.485936884 | 0 | ucrcs10\_19h08\_b madame vinous sweet orange multiple pathogen-infected cdna library ucrcs10 citrus sinensis cdna clone ucrcs10-19h08-p15- mrna |
| CX672990 | S23019058 | 88 | 20 | 75.27511 | 23.3809 | -1.686842115 | 0 | transcription factor bhlh74 ame: full=transcription factor en 90 ame: full=bhlh transcription factor bhlh074 ame: full=basic helix-loop-helix protein 74� |
| CX674161 | S23019647 | 9 | 0 | 7.69859 | 0 | -Inf | 0 | cral trio domain containing expressed |
| CX674852 | S23020020 | 212 | 710 | 181.3446 | 830.0221 | 2.194416169 | 0 | 1-aminocyclopropane-1-carboxylate oxidase 1� |
| CX674940 | S23020066 | 209 | 677 | 178.7784 | 791.4436 | 2.146314301 | 0 | cyclic nucleotide-gated ion channel 1� |
| CX674977 | S23020085 | 317 | 47 | 271.1615 | 54.94512 | -2.303088769 | 0 | oligopeptide transporter 1� |
| CX671291 | S23020112 | 480 | 73 | 410.5915 | 85.3403 | -2.266404628 | 0 | receptor-like protein kinase hsl1 ame: full=protein haesa-like1 flags: precursor |
| CX671357 | S23020146 | 186 | 5 | 159.1042 | 5.845226 | -4.766569307 | 0 | l-ascorbate oxidase homolog flags: precursor |
| CX671667 | S23020305 | 42 | 141 | 35.92675 | 164.8354 | 2.197895339 | 0 | basic 7s globulin ame: full=sbg7s� |
| CX672178 | S23020569 | 1027 | 178 | 878.4947 | 208.09 | -2.077825626 | 0 | ucrcs10\_15d12\_b madame vinous sweet orange multiple pathogen-infected cdna library ucrcs10 citrus sinensis cdna clone ucrcs10-15d12-h23- mrna |
| CX672401 | S23020684 | 44 | 93 | 37.63755 | 108.7212 | 1.530388601 | 0 | 2og-fe oxygenase family protein |
| CX672855 | S23020920 | 185 | 35 | 158.2488 | 40.91658 | -1.951437035 | 0 | �ribonuclease h protein at1g65750 |
| CX673163 | S23020996 | 3 | 15 | 2.566197 | 17.53568 | 2.772589504 | 0 | tyrosine-sulfated glycopeptide receptor 1 |
| CX673259 | S23021042 | 712 | 662 | 609.044 | 773.9079 | 0.345615385 | 0 | shoot1 protein |
| CX673271 | S23021048 | 52 | 804 | 44.48074 | 939.9123 | 4.401273382 | 0 | probable lrr receptor-like serine threonine-protein kinase at1g56140 flags: precursor |
| CX673376 | S23021107 | 1154 | 183 | 987.1304 | 213.9353 | -2.206066261 | 0 | ring-h2 finger protein atl2n |
| CX674127 | S23021662 | 35 | 107 | 29.93896 | 125.0878 | 2.062845378 | 0 | protein |
| CX674294 | S23021738 | 27 | 0 | 23.09577 | 0 | -Inf | 0 | serine threonine-protein kinase bri1-like 2 ame: full=brassinosteroid insensitive 1-like protein 2 ame: full=protein vascular highway 1 flags: precursor |
| DN134801 | S23749932 | 193 | 42 | 165.092 | 49.0999 | -1.749478205 | 0 | transcription factor bhlh91 ame: full=transcription factor en 25 ame: full=bhlh transcription factor bhlh091 ame: full=basic helix-loop-helix protein 91� |
| DN134873 | S23749962 | 4 | 3 | 3.421596 | 3.507136 | 0.03562391 | 0 | wca02e12 developing fruit albedo at 80 dafb in vulnus5 vector citrus sinensis cdna clone wca02e12 5 mrna |
| DN134913 | S23749981 | 10 | 26 | 8.553989 | 30.39518 | 1.829173032 | 0 | wca03b10 developing fruit albedo at 80 dafb in vulnus5 vector citrus sinensis cdna clone wca03b10 5 mrna |
| DN135042 | S23750027 | 6 | 12 | 5.132394 | 14.02854 | 1.450661409 | 0 | proteinase inhibitor ame: full=luti |
| DN618152 | S24239321 | 124 | 433 | 106.0695 | 506.1966 | 2.254688313 | 0 | anthranilate n-benzoyltransferase |
| DN620202 | S24240495 | 522 | 89 | 446.5182 | 104.045 | -2.101511157 | 0 | populus trichocarpa mrna |
| DN620718 | S24240789 | 83 | 355 | 70.99811 | 415.011 | 2.547297192 | 0 | serine threonine-protein kinase oxi1 ame: full=protein oxidative signal-inducible 1 |
| DN617866 | S24241571 | 9 | 0 | 7.69859 | 0 | -Inf | 0 | conserved hypothetical protein [Ricinus communis] |
| DN619200 | S24242145 | 24 | 250 | 20.52957 | 292.2613 | 3.831483193 | 0 | cytochrome p450 94a2 ame: full=p450-dependent fatty acid omega-hydroxylase |
| DN619212 | S24242149 | 547 | 96 | 467.9032 | 112.2283 | -2.059773113 | 0 | acyltransferase-like protein chloroplastic flags: precursor |
| DN620794 | S24242827 | 43 | 275 | 36.78215 | 321.4874 | 3.127684463 | 0 | f-box family protein |
| DN620931 | S24242884 | 72 | 9 | 61.58872 | 10.52141 | -2.549338591 | 0 | �phagocytic receptor 1b ame: full= -induced gene c protein flags: precursor |
| DN621118 | S24242967 | 565 | 119 | 483.3004 | 139.1164 | -1.796627885 | 0 | polygalacturonase inhibitor ame: full=polygalacturonase-inhibiting protein� |
| DN621618 | S24243179 | 1793 | 399 | 1533.73 | 466.449 | -1.717253428 | 0 | cytokinin dehydrogenase 5 ame: full=cytokinin oxidase 5� |
| CX300836 | S24634758 | 2 | 12 | 1.710798 | 14.02854 | 3.03562391 | 0 | c08002g06sk ootsw1 citrus sinensis cdna clone mrna |
| CX301048 | S24634819 | 4 | 133 | 3.421596 | 155.483 | 5.505943845 | 0 | u-box domain-containing protein 21 ame: full=plant u-box protein 21 |
| CX301592 | S24634997 | 14 | 2 | 11.97558 | 2.33809 | -2.356693513 | 0 | probable elongation factor 1-gamma 2� |
| CX301759 | S24635040 | 46 | 9 | 39.34835 | 10.52141 | -1.902975546 | 0 | c08013c08sk ootsw1 citrus sinensis cdna clone mrna |
| CX301818 | S24635071 | 19 | 193 | 16.25258 | 225.6257 | 3.795190933 | 0 | elongation factor 1-alpha� |
| CX301947 | S24635325 | 4 | 16 | 3.421596 | 18.70472 | 2.450661409 | 0 | basic 7s globulin ame: full=sbg7s� |
| CX302460 | S24635479 | 10 | 28 | 8.553989 | 32.73327 | 1.936088236 | 0 | c08021a05sk ootsw1 citrus sinensis cdna clone mrna |
| CX302838 | S24635647 | 37 | 10 | 31.64976 | 11.69045 | -1.436863862 | 0 | citrus sinensis dna binding protein (v03-2) complete cds |
| CX303152 | S24636024 | 108 | 111 | 92.38308 | 129.764 | 0.490189773 | 0 | wound-induced protein win1 flags: precursor |
| CX303089 | S24636190 | 35 | 0 | 29.93896 | 0 | -Inf | 0 | c08027d11sk ootsw1 citrus sinensis cdna clone mrna |
| CX303285 | S24636282 | 21 | 0 | 17.96338 | 0 | -Inf | 0 | c08029e10sk ootsw1 citrus sinensis cdna clone mrna |
| CX303326 | S24636299 | 341 | 1858 | 291.691 | 2172.086 | 2.896568266 | 0 | protein tify 10a ame: full=jasmonate zim domain-containing protein 1 |
| DR403529 | S25678527 | 14 | 111 | 11.97558 | 129.764 | 3.437722353 | 0 | arabidopsis thaliana at3g62990 complete cds |
| DR403706 | S25678704 | 84 | 153 | 71.85351 | 178.8639 | 1.315731829 | 0 | csah-pnp1246a12 developing fruit peel at 38 dafb citrus sinensis cdna clone csah-pnp1246a12 5 mrna |
| DR403990 | S25678988 | 4 | 23 | 3.421596 | 26.88804 | 2.974223365 | 0 | csah-pnp1246o19 developing fruit peel at 38 dafb citrus sinensis cdna clone csah-pnp1246o19 5 mrna |
| DR404089 | S25679087 | 61 | 13 | 52.17933 | 15.19759 | -1.77963621 | 0 | csag-pnp1245e01 developing fruit juice sac at 38 dafb citrus sinensis cdna clone csag-pnp1245e01 5 mrna |
| DR404453 | S25679451 | 51 | 229 | 43.62535 | 267.7113 | 2.617439855 | 0 | lrr receptor-like serine threonine-protein kinase gso1 ame: full=protein gassho 1 flags: precursor |
| DR404508 | S25679506 | 407 | 20 | 348.1474 | 23.3809 | -3.89629548 | 0 | protein |
| DR405282 | S25680280 | 13 | 42 | 11.12019 | 49.0999 | 2.142539114 | 0 | csab-pnp1240p15 developing fruit albedo at 165 dafb citrus sinensis cdna clone csab-pnp1240p15 5 mrna |
| DR405613 | S25680611 | 148 | 281 | 126.599 | 328.5017 | 1.375634364 | 0 | cysteine chloroplastic chromoplastic ame: full=o-acetylserine sulfhydrylase ame: full=csase b� |
| DR405840 | S25680838 | 272 | 17 | 232.6685 | 19.87377 | -3.549338591 | 0 | protein |
| DR406100 | S25681098 | 491 | 1207 | 420.0009 | 1411.038 | 1.748292155 | 0 | cysteine proteinase rd21a� |
| DR908284 | S26279161 | 47 | 8 | 40.20375 | 9.352362 | -2.103927443 | 0 | transcription factor bhlh68 ame: full=transcription factor en 60 ame: full=bhlh transcription factor bhlh068 ame: full=basic helix-loop-helix protein 68� |
| DR908518 | S26279395 | 60 | 909 | 51.32394 | 1062.662 | 4.371907298 | 0 | cytochrome p450 76c4 |
| DR909350 | S26280227 | 276 | 42 | 236.0901 | 49.0999 | -2.265545625 | 0 | probable pectinesterase pectinesterase inhibitor 34 includes: ame: full=pectinesterase inhibitor 34 ame: full=pectin methylesterase inhibitor 34 includes: ame: full=pectinesterase 34� |
| DR909433 | S26280310 | 53 | 6 | 45.33614 | 7.014271 | -2.692296545 | 0 | populus trichocarpa mrna |
| DR909527 | S26280404 | 5 | 11 | 4.276995 | 12.8595 | 1.588164933 | 0 | usda-fp\_17655 citrus sinensis phloem citrus sinensis cdna clone vpe-10\_b01 5 mrna |
| DR909768 | S26280645 | 556 | 2746 | 475.6018 | 3210.198 | 2.754836246 | 0 | rotenone-insensitive nadh-ubiquinone mitochondrial |
| DR909987 | S26280864 | 456 | 28 | 390.0619 | 32.73327 | -3.574873683 | 0 | proline-rich glycoprotein |
| DR910154 | S26281031 | 15 | 1 | 12.83098 | 1.169045 | -3.456229187 | 0 | cysteine-rich repeat secretory protein 15 flags: precursor |
| DR910614 | S26281491 | 226 | 20 | 193.3202 | 23.3809 | -3.047589459 | 0 | cytochrome p450 716b2 ame: full=cytochrome p450 cypa2 |
| DR910631 | S26281508 | 39 | 8 | 33.36056 | 9.352362 | -1.83474081 | 0 | plasma membrane associated protein |
| DR910681 | S26281558 | 17 | 118 | 14.54178 | 137.9473 | 3.245841617 | 0 | usda-fp\_18809 citrus sinensis phloem citrus sinensis cdna clone vpe-29\_c08 5 mrna |
| DR910720 | S26281597 | 5 | 16 | 4.276995 | 18.70472 | 2.128733314 | 0 | tgacg-sequence-specific dna-binding protein tga-� |
| DR911102 | S26281979 | 34 | 4 | 29.08356 | 4.676181 | -2.636801432 | 0 | usda-fp\_19230 citrus sinensis phloem citrus sinensis cdna clone vpe-45\_d05 5 mrna |
| DR911129 | S26282006 | 5 | 0 | 4.276995 | 0 | -Inf | 0 | populus trichocarpa mrna |
| DR911173 | S26282050 | 62 | 6 | 53.03473 | 7.014271 | -2.918572401 | 0 | usda-fp\_19301 citrus sinensis phloem citrus sinensis cdna clone vpe-31\_a06 5 mrna |
| DR911175 | S26282052 | 38 | 86 | 32.50516 | 100.5379 | 1.62899865 | 0 | usda-fp\_19303 citrus sinensis phloem citrus sinensis cdna clone vpe-45\_d02 5 mrna |
| DR911190 | S26282067 | 16 | 3 | 13.68638 | 3.507136 | -1.96437609 | 0 | usda-fp\_19318 citrus sinensis phloem citrus sinensis cdna clone vpe-19\_c12 5 mrna |
| DR911368 | S26282245 | 36 | 6 | 30.79436 | 7.014271 | -2.134301092 | 0 | metacaspase-3� |
| DR911514 | S26282391 | 72 | 48 | 61.58872 | 56.11417 | -0.134301092 | 0 | usda-fp\_19642 citrus sinensis phloem citrus sinensis cdna clone vpe-24\_d04 5 mrna |
| DR911519 | S26282396 | 153 | 837 | 130.876 | 978.4908 | 2.902357379 | 0 | usda-fp\_19647 citrus sinensis phloem citrus sinensis cdna clone vpe-42\_h04 5 mrna |
| DR911813 | S26282690 | 7 | 33 | 5.987792 | 38.57849 | 2.687700606 | 0 | usda-fp\_19941 citrus sinensis phloem citrus sinensis cdna clone vpe-44\_b01 5 mrna |
| DR911955 | S26282832 | 13 | 1 | 11.12019 | 1.169045 | -3.249778309 | 0 | usda-fp\_20083 citrus sinensis phloem citrus sinensis cdna clone vpe-12\_b08 5 mrna |
| DR911985 | S26282862 | 351 | 61 | 300.245 | 71.31176 | -2.073928474 | 0 | usda-fp\_20113 citrus sinensis phloem citrus sinensis cdna clone vpe-42\_g09 5 mrna |
| DR912053 | S26282930 | 6 | 24 | 5.132394 | 28.05708 | 2.450661409 | 0 | usda-fp\_20181 citrus sinensis phloem citrus sinensis cdna clone vpe-09\_a04 5 mrna |
| DQ028471 | S32321255 | 75 | 1600 | 64.15492 | 1870.472 | 4.865698908 | 0 | 9-cis-epoxycarotenoid dioxygenase chloroplastic ame: full= 1 flags: precursor |
| DQ001728 | S32891011 | 662 | 1503 | 566.2741 | 1757.075 | 1.633603296 | 0 | cytochrome p450 90a1 |
| DY257187 | S34124465 | 169 | 414 | 144.5624 | 483.9847 | 1.74326893 | 0 | protein |
| DY257191 | S34124469 | 13719 | 2332 | 11735.22 | 2726.213 | -2.105874222 | 0 | caffeoyl- o-methyltransferase ame: full=trans-caffeoyl- 3-o-methyltransferase� |
| DY257218 | S34124496 | 24492 | 5781 | 20950.43 | 6758.25 | -1.632258202 | 0 | protein |
| DY257235 | S34124513 | 1751 | 3591 | 1497.804 | 4198.041 | 1.486867978 | 0 | nac domain-containing protein 68� |
| DY257259 | S34124537 | 818 | 2519 | 699.7163 | 2944.825 | 2.073339783 | 0 | kn0aak1da01fm2 ruit citrus sinensis cdna 5 mrna |
| DY257263 | S34124541 | 2716 | 6012 | 2323.263 | 7028.3 | 1.597022939 | 0 | myb-like protein j |
| DY257267 | S34124545 | 226 | 39 | 193.3202 | 45.59276 | -2.084115335 | 0 | nucleobase-ascorbate transporter 2� |
| DY257306 | S34124584 | 45 | 188 | 38.49295 | 219.7805 | 2.513397164 | 0 | u-box domain-containing protein 19 ame: full=plant u-box protein 19 |
| DY257343 | S34124621 | 4085 | 594 | 3494.305 | 694.4128 | -2.331139833 | 0 | expansin-like a2� |
| DY257345 | S34124623 | 188 | 567 | 160.815 | 662.8486 | 2.043277482 | 0 | upf0301 protein plut\_0637 |
| DY257397 | S34124675 | 677 | 1565 | 579.1051 | 1829.556 | 1.659596327 | 0 | ring-h2 finger protein atl2j |
| DY257400 | S34124678 | 51701 | 9257 | 44224.98 | 10821.85 | -2.030914149 | 0 | citrus unshiu mrna for metallothionein-like complete cds |
| DY257444 | S34124722 | 312 | 619 | 266.8845 | 723.639 | 1.439054789 | 0 | umecyanin� |
| DY257446 | S34124724 | 785 | 121 | 671.4882 | 141.4545 | -2.247024197 | 0 | aquaporin tip2-1 ame: full=tonoplast intrinsic protein 2-1� |
| DY257550 | S34124828 | 34 | 154 | 29.08356 | 180.033 | 2.629985108 | 0 | �receptor protein kinase 1 flags: precursor |
| DY257559 | S34124837 | 6 | 18 | 5.132394 | 21.04281 | 2.03562391 | 0 | protein |
| DY257572 | S34124850 | 10 | 22 | 8.553989 | 25.71899 | 1.588164933 | 0 | glutamyl-trna synthetase ame: full=glutamate--trna ligase� |
| DY257584 | S34124862 | 10 | 21 | 8.553989 | 24.54995 | 1.521050737 | 0 | �mo25-like protein at5g47540 |
| DY257639 | S34124917 | 66 | 5 | 56.45633 | 5.845226 | -3.271804615 | 0 | kn0aak3cf02fm1 ruit citrus sinensis cdna 5 mrna |
| DY257659 | S34124937 | 411 | 41 | 351.569 | 47.93085 | -2.87478117 | 0 | citrus unshiu mrna for metallothionein-like complete cds |
| DY305453 | S34125015 | 15 | 42 | 12.83098 | 49.0999 | 1.936088236 | 0 | citrus x paradisi voucher rjb tubeno 51 psbm-trnd intergenic spacer and trna-asp (trnd-guc) partial sequence chloroplast |
| DY305470 | S34125032 | 1621 | 3929 | 1386.602 | 4593.179 | 1.727939486 | 0 | nac domain-containing protein 48� |
| DY305480 | S34125042 | 75 | 517 | 64.15492 | 604.3964 | 3.235863189 | 0 | probable lrr receptor-like serine threonine-protein kinase at2g16250 flags: precursor |
| DY305488 | S34125050 | 63 | 402 | 53.89013 | 469.9562 | 3.124433177 | 0 | transcription factor bhlh35 ame: full=transcription factor en 41 ame: full=bhlh transcription factor bhlh035 ame: full=basic helix-loop-helix protein 35� |
| DY305490 | S34125052 | 10930 | 26489 | 9349.51 | 30966.84 | 1.727761389 | 0 | indole-3-acetic acid-induced protein arg2 |
| DY305493 | S34125055 | 245 | 504 | 209.5727 | 589.1988 | 1.491303394 | 0 | beta- insoluble isoenzyme cwinv3 ame: full=cell wall invertase 3� |
| DY305505 | S34125067 | 925 | 1947 | 791.244 | 2276.131 | 1.524389022 | 0 | protein |
| DY305530 | S34125092 | 14 | 854 | 11.97558 | 998.3646 | 6.381398747 | 0 | chlorophyllase- chloroplastic ame: full=chlorophyll-chlorophyllido hydrolase 1� |
| DY305550 | S34125112 | 4267 | 11785 | 3649.987 | 13777.2 | 1.916319152 | 0 | polyol transporter 5 ame: full=sugar-proton symporter plt5 ame: full=protein polyol transporter 5� |
| DY305565 | S34125127 | 254 | 33 | 217.2713 | 38.57849 | -2.493629158 | 0 | probable lrr receptor-like serine threonine-protein kinase at2g23950 flags: precursor |
| DY305569 | S34125131 | 257 | 938 | 219.8375 | 1096.564 | 2.318480972 | 0 | protein tify 9 ame: full=protein jasmonate-associated 1 ame: full=jasmonate zim domain-containing protein 10 |
| DY305571 | S34125133 | 491 | 1494 | 420.0009 | 1746.554 | 2.056046627 | 0 | mlo-like protein 6� |
| DY305580 | S34125142 | 2397 | 10063 | 2050.391 | 11764.1 | 2.520420063 | 0 | zinc finger protein 1 ame: full=wzf1 |
| DY305603 | S34125165 | 180 | 352 | 153.9718 | 411.5039 | 1.418239931 | 0 | upf0676 protein |
| DY305607 | S34125169 | 627 | 2194 | 536.3351 | 2564.885 | 2.257687587 | 0 | protein |
| DY305611 | S34125173 | 107 | 456 | 91.52768 | 533.0846 | 2.542084437 | 0 | phytosulfokine receptor 1� |
| DY305613 | S34125175 | 47 | 656 | 40.20375 | 766.8936 | 4.253624562 | 0 | transcription factor bhlh36 ame: full=transcription factor en 6 ame: full=bhlh transcription factor bhlh036 ame: full=basic helix-loop-helix protein 36� |
| DY305677 | S34125239 | 96 | 294 | 82.1183 | 343.6993 | 2.065371253 | 0 | xylem cysteine proteinase 1� |
| DY305681 | S34125243 | 137 | 827 | 117.1897 | 966.8004 | 3.044372845 | 0 | ethylene-responsive transcription factor erf017 |
| DY305690 | S34125252 | 378 | 4085 | 323.3408 | 4775.55 | 3.884539348 | 0 | probable glutathione s-transferase ame: full=auxin-induced protein pgnt35 pcnt111 |
| DY305694 | S34125256 | 330 | 2260 | 282.2816 | 2642.042 | 3.226446252 | 0 | potassium channel tetramerization domain-containing |
| DY305711 | S34125273 | 5 | 12 | 4.276995 | 14.02854 | 1.713695815 | 0 | unnamed protein product [Vitis vinifera] |
| DY305725 | S34125287 | 159 | 2454 | 136.0084 | 2868.837 | 4.398697987 | 0 | tropinone reductase homolog at1g07440 |
| DY305732 | S34125294 | 1421 | 4005 | 1215.522 | 4682.026 | 1.945557097 | 0 | ring-h2 finger protein atl3f ame: full=ring-h2 finger protein atl2 |
| DY305736 | S34125298 | 147 | 873 | 125.7436 | 1020.576 | 3.020826908 | 0 | cytochrome p450 71b35 |
| DY305817 | S34125379 | 528 | 1293 | 451.6506 | 1511.575 | 1.742773849 | 0 | glycosyltransferase 8 domain-containing protein 1 |
| DY305857 | S34125419 | 1697 | 3331 | 1451.612 | 3894.09 | 1.423630198 | 0 | protein |
| DY305861 | S34125423 | 5 | 280 | 4.276995 | 327.3327 | 6.258016331 | 0 | protein |
| DY305873 | S34125435 | 3019 | 6603 | 2582.449 | 7719.205 | 1.579712298 | 0 | ammonium transporter 1 member 1 ame: full= 1 1 |
| DY305885 | S34125447 | 50 | 136 | 42.76995 | 158.9901 | 1.89426806 | 0 | monoglyceride lipase� |
| DY305898 | S34125460 | 273 | 1399 | 233.5239 | 1635.494 | 2.808084515 | 0 | calcium-binding protein cml37 ame: full=calmodulin-like protein 37 |
| DY305904 | S34125466 | 4617 | 255 | 3949.377 | 298.1065 | -3.727725171 | 0 | 21 kda protein ame: full= protein flags: precursor |
| DY305912 | S34125474 | 32 | 87 | 27.37277 | 101.7069 | 1.893604905 | 0 | polyneuridine-aldehyde esterase ame: full=polyneuridine aldehyde esterase flags: precursor |
| DY305919 | S34125481 | 1508 | 61 | 1289.942 | 71.31176 | -4.177021967 | 0 | PREDICTED: hypothetical protein [Vitis vinifera] |
| DY305945 | S34125507 | 29 | 60 | 24.80657 | 70.14271 | 1.499571009 | 0 | nodule inception protein |
| DY306000 | S34125562 | 73 | 141 | 62.44412 | 164.8354 | 1.400388203 | 0 | �adenylate cyclase regulatory protein |
| DY306001 | S34125563 | 428 | 3230 | 366.1107 | 3776.016 | 3.366512872 | 0 | nac domain-containing protein 72� |
| DY306009 | S34125571 | 1519 | 243 | 1299.351 | 284.078 | -2.193432242 | 0 | lob domain-containing protein 40 ame: full=asymmetric leaves 2-like protein 37� |
| DY306014 | S34125576 | 116 | 935 | 99.22628 | 1093.057 | 3.461502969 | 0 | probable lrr receptor-like serine threonine-protein kinase at3g47570 flags: precursor |
| DY306026 | S34125588 | 212 | 32 | 181.3446 | 37.40945 | -2.277259046 | 0 | probable wound-induced protein |
| DY306086 | S34125648 | 50 | 468 | 42.76995 | 547.1132 | 3.677169939 | 0 | receptor serine threonine |
| DY306100 | S34125662 | 1635 | 4131 | 1398.577 | 4829.326 | 1.787861833 | 0 | myb-like protein j |
| DY306136 | S34125698 | 0 | 360 | 0 | 420.8563 | Inf | 0 | jasmonate o-methyltransferase ame: full=s-adenosyl-l-methionine:jasmonic acid carboxyl methyltransferase ame: full=floral nectary-specific protein 1 |
| DY306148 | S34125710 | 22471 | 5345 | 19221.67 | 6248.547 | -1.621141066 | 0 | caffeoyl- o-methyltransferase ame: full=trans-caffeoyl- 3-o-methyltransferase� |
| DY306159 | S34125721 | 1229 | 2893 | 1051.285 | 3382.048 | 1.685742817 | 0 | probable glutathione s-transferase ame: full=heat shock protein 26a ame: full=g2-4 |
| DY306171 | S34125733 | 141 | 305 | 120.6112 | 356.5588 | 1.563775489 | 0 | anthranilate n-benzoyltransferase protein 2 ame: full=anthranilate n-hydroxycinnamoyl benzoyltransferase 2 |
| DY306179 | S34125741 | 2849 | 10609 | 2437.032 | 12402.4 | 2.347422557 | 0 | protein |
| DY306181 | S34125743 | 460 | 4578 | 393.4835 | 5351.889 | 3.765673106 | 0 | protein |
| DY306194 | S34125756 | 283 | 746 | 242.0779 | 872.1077 | 1.849034986 | 0 | purine permease 1� |
| DY306872 | S34125767 | 5 | 94 | 4.276995 | 109.8902 | 4.683322166 | 0 | transcription factor bhlh36 ame: full=transcription factor en 6 ame: full=bhlh transcription factor bhlh036 ame: full=basic helix-loop-helix protein 36� |
| AB276108 | S35152777 | 16 | 190 | 13.68638 | 222.1186 | 4.020517017 | 0 | beta- soluble isoenzyme i ame: full=sucrose hydrolase ame: full=invertase ame: full=saccharase flags: precursor |
| EG358279 | S35174889 | 473 | 5847 | 404.6037 | 6835.407 | 4.078445912 | 0 | blue copper protein flags: precursor |
| EG358322 | S35174932 | 27690 | 5853 | 23686 | 6842.422 | -1.791455461 | 0 | leucoanthocyanidin dioxygenase� |
| EG358327 | S35174937 | 5 | 6 | 4.276995 | 7.014271 | 0.713695815 | 0 | pectinesterase 2� |
| EG358332 | S35174942 | 3 | 13 | 2.566197 | 15.19759 | 2.566138626 | 0 | serine-threonine protein plant- |
| EG358340 | S35174950 | 11 | 33 | 9.409388 | 38.57849 | 2.03562391 | 0 | cytochrome b5 |
| EF010854 | S35394641 | 817 | 2448 | 698.8609 | 2861.823 | 2.033856984 | 0 | calcium-binding allergen bet v 3 ame: full=allergen bet v iii ame: allergen=bet v 3 |
| EF185419 | S36619343 | 976 | 3297 | 834.8693 | 3854.342 | 2.206862243 | 0 | nac domain-containing protein 29� |
| EF596736 | S38754319 | 2737 | 8407 | 2341.227 | 9828.163 | 2.069657047 | 0 | nac domain-containing protein 2� |
| EU200366 | S41488901 | 30 | 305 | 25.66197 | 356.5588 | 3.796436246 | 0 | taxadien-5-alpha-ol o-acetyltransferase ame: full=taxa-4 -dien-5alpha-ol-o-acetyltransferase� |
| EY649677 | S44206839 | 16 | 39 | 13.68638 | 45.59276 | 1.736063628 | 0 | cs00-c1-100-002-c07- sweet orange greenhouse plant citrus sinensis mrna |
| EY649794 | S44206956 | 31 | 0 | 26.51737 | 0 | -Inf | 0 | beta-galactosidase 8� |
| EY649916 | S44207078 | 171 | 382 | 146.2732 | 446.5753 | 1.610237722 | 0 | prolyl endopeptidase� |
| EY650326 | S44207376 | 233 | 754 | 199.3079 | 881.4601 | 2.144895978 | 0 | �uncharacterized protein ycf15 |
| EY650356 | S44207406 | 105 | 24 | 89.81689 | 28.05708 | -1.678621608 | 0 | auxin-binding protein t92� |
| EY650515 | S44207565 | 179 | 717 | 153.1164 | 838.2054 | 2.45267494 | 0 | cytochrome p450 |
| EY650552 | S44207602 | 1114 | 28 | 952.9144 | 32.73327 | -4.863517186 | 0 | subtilisin-like protease ame: full=cucumisin-like serine protease flags: precursor |
| EY650597 | S44207647 | 41 | 158 | 35.07136 | 184.7091 | 2.396890153 | 0 | cs00-c1-100-012-g05- sweet orange greenhouse plant citrus sinensis mrna |
| EY650607 | S44207657 | 218 | 43 | 186.477 | 50.26894 | -1.891258161 | 0 | uncharacterized calcium-binding protein at1g02270 |
| EY650692 | S44207742 | 247 | 39 | 211.2835 | 45.59276 | -2.212303604 | 0 | cytochrome p450 83b1 |
| EY651373 | S44208087 | 897 | 2566 | 767.2928 | 2999.77 | 1.967002689 | 0 | protein |
| EY651594 | S44208308 | 13 | 3 | 11.12019 | 3.507136 | -1.664815808 | 0 | eugenol synthase 1 |
| EY651631 | S44208345 | 119 | 25 | 101.7925 | 29.22613 | -1.800300165 | 0 | monocopper oxidase-like protein sku5 ame: full=skewed roots flags: precursor |
| EY651762 | S44208476 | 441 | 1818 | 377.2309 | 2125.324 | 2.494163048 | 0 | branched-chain-amino-acid aminotransferase chloroplastic� |
| EY652191 | S44208695 | 10 | 2 | 8.553989 | 2.33809 | -1.871266686 | 0 | expansin-b3� |
| EY652211 | S44208715 | 923 | 2466 | 789.5332 | 2882.865 | 1.868431656 | 0 | PREDICTED: hypothetical protein [Vitis vinifera] |
| EY652427 | S44208819 | 12 | 37 | 10.26479 | 43.25467 | 2.075152274 | 0 | protein |
| EY652542 | S44208934 | 109 | 238 | 93.23848 | 278.2328 | 1.577294848 | 0 | calcineurin b-like protein 10 |
| EY652718 | S44209110 | 357 | 750 | 305.3774 | 876.7839 | 1.52162793 | 0 | �disease resistance protein at1g59780 |
| EY652848 | S44209240 | 15 | 2 | 12.83098 | 2.33809 | -2.456229187 | 0 | patatin group d-2 flags: precursor |
| EY652932 | S44209324 | 450 | 98 | 384.9295 | 114.5664 | -1.748409938 | 0 | �homolog subfamily c member 1 ame: full= protein homolog mtj1 flags: precursor |
| EY652975 | S44209367 | 120 | 40 | 102.6479 | 46.76181 | -1.134301092 | 0 | protein iq-domain 1 |
| EY653362 | S44209586 | 87 | 198 | 74.41971 | 231.4709 | 1.637074533 | 0 | transmembrane protein 45b |
| EY653870 | S44209870 | 21 | 51 | 17.96338 | 59.6213 | 1.730769328 | 0 | populus trichocarpa mrna |
| EY653900 | S44209900 | 992 | 94 | 848.5557 | 109.8902 | -2.94894605 | 0 | cs00-c1-100-049-f01- sweet orange greenhouse plant citrus sinensis mrna |
| EY654505 | S44210421 | 1006 | 194 | 860.5313 | 226.7948 | -1.923840339 | 0 | calmodulin-like protein 3 |
| EY654514 | S44210430 | 17 | 41 | 14.54178 | 47.93085 | 1.720750572 | 0 | cs00-c1-100-056-d04- sweet orange greenhouse plant citrus sinensis mrna |
| EY654816 | S44210732 | 1192 | 171 | 1019.636 | 199.9067 | -2.350654597 | 0 | (+)-delta-cadinene synthase isozyme xc1� |
| EY654875 | S44210791 | 84 | 5 | 71.85351 | 5.845226 | -3.619727919 | 0 | probable polygalacturonase� |
| EY654877 | S44210793 | 64 | 213 | 54.74553 | 249.0066 | 2.185371029 | 0 | protein dehydration-induced 19 homolog 4 ame: full= i19-4 |
| EY655271 | S44210977 | 34 | 66 | 29.08356 | 77.15698 | 1.407592687 | 0 | cc-nbs-lrr resistance protein |
| EY655298 | S44211004 | 141 | 723 | 120.6112 | 845.2197 | 2.808961894 | 0 | sulfate transporter ame: full=ast12 ame: full= 1 |
| EY655509 | S44211215 | 165 | 517 | 141.1408 | 604.3964 | 2.098359665 | 0 | nodulin-like protein |
| EY655547 | S44211253 | 8 | 173 | 6.843191 | 202.2448 | 4.885289637 | 0 | ankyrin repeat-containing protein at2g01680 |
| EY655817 | S44211439 | 24 | 102 | 20.52957 | 119.2426 | 2.53812425 | 0 | cs00-c1-100-082-f09- sweet orange greenhouse plant citrus sinensis mrna |
| EY656041 | S44211663 | 138 | 26 | 118.0451 | 30.39518 | -1.95742333 | 0 | subtilisin-like protease ame: full=cucumisin-like serine protease flags: precursor |
| EY656063 | S44211685 | 51 | 5 | 43.62535 | 5.845226 | -2.899835838 | 0 | uncharacterized basic helix-loop-helix protein at1g06150 |
| EY656292 | S44211802 | 3704 | 846 | 3168.398 | 989.0122 | -1.679693121 | 0 | glutamyl-trna amidotransferase subunit a homolog ame: full=glutaminyl-trna synthase-like protein 1 |
| EY656296 | S44211806 | 14 | 37 | 11.97558 | 43.25467 | 1.852759853 | 0 | tocopherol chloroplastic ame: full=vitamin e pathway gene 1 protein ame: full=sucrose export defective 1 flags: precursor |
| EY656328 | S44211838 | 168 | 27 | 143.707 | 31.56422 | -2.186768512 | 0 | dna-damage-inducible protein |
| EY656372 | S44211882 | 2166 | 5503 | 1852.794 | 6433.256 | 1.795846495 | 0 | protein tify 6b ame: full=jasmonate zim domain-containing protein 3 ame: full=protein jasmonate insensitive 3 |
| EY656449 | S44211959 | 67 | 5 | 57.31173 | 5.845226 | -3.293499687 | 0 | myristoyl-acyl carrier protein chloroplastic ame: full=16:0-acyl-carrier protein thioesterase� |
| EY656512 | S44212022 | 151 | 448 | 129.1652 | 523.7322 | 2.019611592 | 0 | poncirus trifoliata citrus tristeza virus resistance gene complete sequence |
| EY656524 | S44212034 | 55 | 3 | 47.04694 | 3.507136 | -3.745735804 | 0 | protein hothead ame: full=protein adhesion of calyx edges flags: precursor |
| EY656785 | S44212141 | 87 | 205 | 74.41971 | 239.6543 | 1.687198013 | 0 | pathogen-related protein |
| EY657045 | S44212289 | 57 | 12 | 48.75774 | 14.02854 | -1.797266104 | 0 | kinesin-like calmodulin-binding protein |
| EY657141 | S44212343 | 155 | 412 | 132.5868 | 481.6466 | 1.861037531 | 0 | predicted protein [Populus trichocarpa] |
| EY657220 | S44212422 | 92 | 10 | 78.6967 | 11.69045 | -2.750972452 | 0 | l-ascorbate oxidase homolog ame: full=pollen-specific protein ntp303 flags: precursor |
| EY657225 | S44212427 | 1788 | 410 | 1529.453 | 479.3085 | -1.673989513 | 0 | probable xyloglucan endotransglucosylase hydrolase protein 23� |
| EY657391 | S44212537 | 694 | 161 | 593.6469 | 188.2163 | -1.657213565 | 0 | zebrafish dna sequence from clone ch211-198o8 in linkage group complete sequence |
| EY657461 | S44212607 | 601 | 77 | 514.0948 | 90.01648 | -2.513773231 | 0 | predicted protein [Populus trichocarpa] |
| EY657482 | S44212628 | 10911 | 1097 | 9333.258 | 1282.443 | -2.863486492 | 0 | probable non-specific lipid-transfer protein akcs9� |
| EY657678 | S44212712 | 844 | 2167 | 721.9567 | 2533.321 | 1.811045658 | 0 | probable inositol transporter 1 |
| EY657722 | S44212756 | 687 | 2431 | 587.6591 | 2841.949 | 2.273829298 | 0 | probable phosphatase phospho1 |
| EY657733 | S44212767 | 805 | 104 | 688.5961 | 121.5807 | -2.501743846 | 0 | probable polygalacturonase non-catalytic subunit jp650 ame: full=aromatic-rich glycoprotein jp650 flags: precursor |
| EY657918 | S44212952 | 146 | 296 | 124.8882 | 346.0374 | 1.470290216 | 0 | predicted protein [Populus trichocarpa] |
| EY658058 | S44213092 | 736 | 1474 | 629.5736 | 1723.173 | 1.452620262 | 0 | serine--glyoxylate aminotransferase ame: full=alanine--glyoxylate aminotransferase� |
| EY658083 | S44213117 | 196 | 422 | 167.6582 | 493.3371 | 1.557050754 | 0 | protein |
| EY658129 | S44213149 | 201 | 8 | 171.9352 | 9.352362 | -4.200390282 | 0 | aquaporin nip2-1 ame: full=nod26-like intrinsic protein 2-1 ame: full= 2 1 ame: full=silicon transporter lsi1 ame: full=low silicon protein 1 |
| EY658336 | S44213244 | 19 | 4 | 16.25258 | 4.676181 | -1.797266104 | 0 | 14 kda proline-rich protein flags: precursor |
| EY658341 | S44213249 | 243 | 50 | 207.8619 | 58.45226 | -1.830294905 | 0 | chalcone-flavanone isomerase family expressed |
| EY658360 | S44213268 | 10223 | 2337 | 8744.743 | 2732.059 | -1.678427578 | 0 | s-adenosylmethionine synthetase� |
| EY658528 | S44213324 | 1272 | 262 | 1088.067 | 306.2898 | -1.828798545 | 0 | probable glycerophosphoryl diester phosphodiesterase 2 flags: precursor |
| EY658548 | S44213344 | 2 | 4 | 1.710798 | 4.676181 | 1.450661409 | 0 | citrus sinensis complete genome |
| EY658585 | S44213381 | 306 | 34 | 261.7521 | 39.74754 | -2.719263592 | 0 | solute carrier family 35 member f1 |
| EY658610 | S44213406 | 52 | 2 | 44.48074 | 2.33809 | -4.249778309 | 0 | gdsl esterase lipase at2g04570 ame: full=extracellular lipase at2g04570 flags: precursor |
| EY658612 | S44213408 | 446 | 1578 | 381.5079 | 1844.753 | 2.273642999 | 0 | aldehyde dehydrogenase family 2 member c4 ame: full=aldh1a ame: full=protein reduced epidermal fluorescence 1 |
| EY658639 | S44213435 | 14 | 33 | 11.97558 | 38.57849 | 1.687700606 | 0 | crr23 ((chlororespiratory reduction 23) |
| EY658729 | S44213525 | 13 | 1 | 11.12019 | 1.169045 | -3.249778309 | 0 | cs00-c1-100-138-f12- sweet orange greenhouse plant citrus sinensis mrna |
| EY658773 | S44213569 | 244 | 491 | 208.7173 | 574.0012 | 1.459503286 | 0 | cbl-interacting serine threonine-protein kinase 17 ame: full=sos2-like protein kinase pks20 ame: full=snf1-related kinase |
| EY658787 | S44213583 | 847 | 136 | 724.5229 | 158.9901 | -2.188093909 | 0 | (+)-delta-cadinene synthase isozyme a� |
| EY658792 | S44213588 | 13 | 45 | 11.12019 | 52.60703 | 2.242074787 | 0 | taxadien-5-alpha-ol o-acetyltransferase ame: full=taxa-4 -dien-5alpha-ol-o-acetyltransferase� |
| EY658811 | S44213607 | 453 | 8 | 387.4957 | 9.352362 | -5.372705831 | 0 | fasciclin-like arabinogalactan protein 2 flags: precursor |
| EY659167 | S44213753 | 326 | 70 | 278.86 | 81.83316 | -1.768783728 | 0 | at3g19540 t31j18\_4 |
| EY659177 | S44213763 | 96 | 243 | 82.1183 | 284.078 | 1.790511412 | 0 | cs00-c1-101-004-c05- sweet orange infected with xylella fastidiosa (stage 1 of 2) citrus sinensis mrna |
| EY659189 | S44213775 | 58 | 15 | 49.61314 | 17.53568 | -1.500428991 | 0 | PREDICTED: hypothetical protein [Vitis vinifera] |
| EY659543 | S44214017 | 228 | 37 | 195.031 | 43.25467 | -2.17277524 | 0 | phosphatase chloroplastic ame: full=protein myo-inositol monophosphatase-like 2 flags: precursor |
| EY659666 | S44214140 | 107 | 12 | 91.52768 | 14.02854 | -2.705843077 | 0 | Pc13g00140 [Penicillium chrysogenum Wisconsin 54-1255] |
| EY659778 | S44214252 | 11 | 4 | 9.409388 | 4.676181 | -1.00877021 | 0 | armadillo beta-catenin repeat family protein |
| EY660216 | S44214578 | 3 | 16 | 2.566197 | 18.70472 | 2.865698908 | 0 | alpha-glucosidase yihq |
| EY660334 | S44214696 | 2001 | 4095 | 1711.653 | 4787.24 | 1.483803694 | 0 | o-succinylhomoserine sulfhydrylase� |
| EY660548 | S44214910 | 148 | 403 | 126.599 | 471.1252 | 1.895844072 | 0 | zinc finger protein 4 |
| EY660689 | S44215051 | 126 | 156 | 107.7803 | 182.3711 | 0.758783704 | 0 | protein |
| EY661071 | S44215209 | 132 | 5 | 112.9127 | 5.845226 | -4.271804615 | 0 | cs00-c1-101-029-b02- sweet orange infected with xylella fastidiosa (stage 1 of 2) citrus sinensis mrna |
| EY661649 | S44215451 | 192 | 437 | 164.2366 | 510.8727 | 1.637188378 | 0 | cytochrome p450 87a3 |
| EY661705 | S44215507 | 31 | 122 | 26.51737 | 142.6235 | 2.427202436 | 0 | cs00-c1-101-036-b01- sweet orange infected with xylella fastidiosa (stage 1 of 2) citrus sinensis mrna |
| EY661918 | S44215608 | 147 | 7 | 125.7436 | 8.183316 | -3.941656014 | 0 | protein |
| EY662029 | S44215719 | 4 | 11 | 3.421596 | 12.8595 | 1.910093028 | 0 | cs00-c1-101-039-f06- sweet orange infected with xylella fastidiosa (stage 1 of 2) citrus sinensis mrna |
| EY662916 | S44216158 | 39 | 79 | 33.36056 | 92.35457 | 1.469039938 | 0 | tir-nbs-lrr resistance protein |
| EY663023 | S44216265 | 63 | 194 | 53.89013 | 226.7948 | 2.073294328 | 0 | �domain-containing protein chloroplastic ame: full= -related thylakoid lumenal protein 1 ame: full=oec23-like protein 3 flags: precursor |
| EY663343 | S44216361 | 445 | 56 | 380.6525 | 65.46653 | -2.539645195 | 0 | protein phloem protein 2-like a9� |
| EY664279 | S44217073 | 100 | 14 | 85.53989 | 16.36663 | -2.385839859 | 0 | af372895\_1 at2g04793 |
| EY664527 | S44217321 | 228 | 42 | 195.031 | 49.0999 | -1.989911182 | 0 | agglutinin ame: full=cca |
| EY664535 | S44217329 | 3 | 90 | 2.566197 | 105.2141 | 5.357552005 | 0 | PREDICTED: hypothetical protein [Vitis vinifera] |
| EY664636 | S44217430 | 1041 | 198 | 890.4703 | 231.4709 | -1.943736324 | 0 | acyltransferase-like protein chloroplastic flags: precursor |
| EY665087 | S44217657 | 42 | 11 | 35.92675 | 12.8595 | -1.482224395 | 0 | rab6-interacting golgin ame: full=n-terminal kinase-like-binding protein 1� |
| EY665091 | S44217661 | 7 | 21 | 5.987792 | 24.54995 | 2.03562391 | 0 | medicago truncatula chromosome 8 clone mth2- complete sequence |
| EY665163 | S44217733 | 8 | 19 | 6.843191 | 22.21186 | 1.698588922 | 0 | elongation factor tu gtp-binding domain-containing protein 1 ame: full=protein fam42a |
| EY665215 | S44217785 | 116 | 15 | 99.22628 | 17.53568 | -2.500428991 | 0 | glycerol-3-phosphate acyltransferase 6� |
| EY665231 | S44217801 | 686 | 1633 | 586.8037 | 1909.051 | 1.701905718 | 0 | �zinc finger protein at1g68190 |
| EY665285 | S44217855 | 10 | 26 | 8.553989 | 30.39518 | 1.829173032 | 0 | cs00-c1-101-086-b08- sweet orange infected with xylella fastidiosa (stage 1 of 2) citrus sinensis mrna |
| EY665311 | S44217881 | 328 | 65 | 280.5708 | 75.98794 | -1.884522783 | 0 | predicted protein [Populus trichocarpa] |
| EY665389 | S44217959 | 223 | 29 | 190.754 | 33.90231 | -2.492257496 | 0 | cs00-c1-101-007-d02- sweet orange infected with xylella fastidiosa (stage 1 of 2) citrus sinensis mrna |
| EY665420 | S44217990 | 60 | 176 | 51.32394 | 205.752 | 2.003202432 | 0 | purple acid phosphatase 2 ame: full=manganese purple acid phosphatase 2 flags: precursor |
| EY666753 | S44219001 | 119 | 349 | 101.7925 | 407.9968 | 2.002926872 | 0 | probable aminotransferase acs10 |
| EY666801 | S44219049 | 87 | 384 | 74.41971 | 448.9134 | 2.592680414 | 0 | protein |
| EY666881 | S44219129 | 90 | 270 | 76.9859 | 315.6422 | 2.03562391 | 0 | at3g04350 t6k12\_3 |
| EY666910 | S44219158 | 708 | 726 | 605.6224 | 848.7268 | 0.486881597 | 0 | thylakoid soluble phosphoprotein |
| EY667138 | S44219274 | 646 | 138 | 552.5877 | 161.3282 | -1.776204489 | 0 | conserved hypothetical protein [Ricinus communis] |
| EY667487 | S44219511 | 194 | 742 | 165.9474 | 867.4315 | 2.386023943 | 0 | abc transporter c family member 10� |
| EY668011 | S44219699 | 108 | 502 | 92.38308 | 586.8607 | 2.667317461 | 0 | reticuline oxidase-like protein flags: precursor |
| EY668288 | S44219864 | 100 | 212 | 85.53989 | 247.8376 | 1.534725674 | 0 | probable s-acyltransferase at3g22180 ame: full=probable palmitoyltransferase at3g22180 ame: full=zinc finger dhhc domain-containing protein at3g22180 |
| EY668407 | S44219983 | 1636 | 338 | 1399.433 | 395.1373 | -1.824416188 | 0 | carbonic chloroplastic ame: full=carbonate dehydratase flags: precursor |
| EY668560 | S44220136 | 87 | 230 | 74.41971 | 268.8804 | 1.853207964 | 0 | cs00-c1-102-043-a04- sweet orange infected with xylella fastidiosa (stage 2 of 2) citrus sinensis mrna |
| EY668853 | S44220429 | 25 | 120 | 21.38497 | 140.2854 | 2.713695815 | 0 | PREDICTED: hypothetical protein [Vitis vinifera] |
| EY669346 | S44220922 | 27 | 59 | 23.09577 | 68.97367 | 1.578416956 | 0 | cs00-c1-102-049-g11- sweet orange infected with xylella fastidiosa (stage 2 of 2) citrus sinensis mrna |
| EY670035 | S44221289 | 19 | 58 | 16.25258 | 67.80462 | 2.060714891 | 0 | probable lrr receptor-like serine threonine-protein kinase at1g53430 flags: precursor |
| EY670039 | S44221293 | 144 | 352 | 123.1774 | 411.5039 | 1.740168026 | 0 | probable aminotransferase acs10 |
| EY670060 | S44221314 | 106 | 805 | 90.67229 | 941.0814 | 3.375585927 | 0 | protein |
| EY670694 | S44221724 | 787 | 1678 | 673.199 | 1961.658 | 1.542968584 | 0 | probable serine threonine-protein kinase wnk5� |
| EY670781 | S44221811 | 16 | 80 | 13.68638 | 93.52362 | 2.772589504 | 0 | protein |
| EY671060 | S44221978 | 62 | 174 | 53.03473 | 203.4139 | 1.939408594 | 0 | protein binding |
| EY672423 | S44222893 | 2425 | 129 | 2074.342 | 150.8068 | -3.781880368 | 0 | heat shock protein 83 |
| EY672670 | S44223028 | 451 | 1186 | 385.7849 | 1386.488 | 1.84556608 | 0 | thioredoxin-like 1 |
| EY673147 | S44223057 | 152 | 298 | 130.0206 | 348.3755 | 1.421902416 | 0 | cs00-c1-102-105-f12- sweet orange infected with xylella fastidiosa (stage 2 of 2) citrus sinensis mrna |
| EY673920 | S44223494 | 261 | 61 | 223.2591 | 71.31176 | -1.64650725 | 0 | cs00-c1-102-004-g03- sweet orange infected with xylella fastidiosa (stage 2 of 2) citrus sinensis mrna |
| EY674006 | S44223580 | 70 | 158 | 59.87792 | 184.7091 | 1.62515914 | 0 | caleosin-related family protein |
| EY674214 | S44223690 | 132 | 383 | 112.9127 | 447.7443 | 1.987467872 | 0 | protein chloroplast import apparatus 2 flags: precursor |
| EY674219 | S44223695 | 54 | 129 | 46.19154 | 150.8068 | 1.707001162 | 0 | cs00-c1-102-022-b11- sweet orange infected with xylella fastidiosa (stage 2 of 2) citrus sinensis mrna |
| EY674234 | S44223710 | 8 | 39 | 6.843191 | 45.59276 | 2.736063628 | 0 | cs00-c1-102-024-a07- sweet orange infected with xylella fastidiosa (stage 2 of 2) citrus sinensis mrna |
| EY674291 | S44223767 | 397 | 804 | 339.5934 | 939.9123 | 1.468717903 | 0 | 7-ethoxycoumarin o-deethylase� |
| EY674342 | S44223818 | 10 | 32 | 8.553989 | 37.40945 | 2.128733314 | 0 | cs00-c1-102-075-b02- sweet orange infected with xylella fastidiosa (stage 2 of 2) citrus sinensis mrna |
| EY674438 | S44223914 | 210 | 705 | 179.6338 | 824.1769 | 2.197895339 | 0 | yela protein gb |
| EY674449 | S44223925 | 1388 | 250 | 1187.294 | 292.2613 | -2.022346159 | 0 | protein |
| EY674949 | S44224215 | 2 | 18 | 1.710798 | 21.04281 | 3.62058641 | 0 | dibenzothiophene desulfurization enzyme c ame: full=dbt sulfur dioxygenase |
| EY675103 | S44224369 | 81 | 11 | 69.28731 | 12.8595 | -2.429756975 | 0 | peroxidase 4 flags: precursor |
| EY675106 | S44224372 | 1235 | 98 | 1056.418 | 114.5664 | -3.204924073 | 0 | probable xyloglucan endotransglucosylase hydrolase protein 6� |
| EY675166 | S44224432 | 39 | 76 | 33.36056 | 88.84743 | 1.413186704 | 0 | cs00-c1-401-007-a07- sweet orange infected with citrus sinensis mrna |
| EY675218 | S44224484 | 87 | 16 | 74.41971 | 18.70472 | -1.992282087 | 0 | protein |
| EY675370 | S44224636 | 130 | 419 | 111.2019 | 489.8299 | 2.13910003 | 0 | cs00-c1-401-009-f08- sweet orange infected with citrus sinensis mrna |
| EY675407 | S44224673 | 2650 | 377 | 2266.807 | 440.73 | -2.362694522 | 0 | heat shock factor protein |
| EY675507 | S44224773 | 11 | 0 | 9.409388 | 0 | -Inf | 0 | citrus x paradisi miraculin-like protein 2 complete cds |
| EY675550 | S44224816 | 37 | 82 | 31.64976 | 95.86171 | 1.598760048 | 0 | transcription factor bhlh128 ame: full=transcription factor en 74 ame: full=bhlh transcription factor bhlh128 ame: full=basic helix-loop-helix protein 128� |
| EY675558 | S44224824 | 332 | 21 | 283.9924 | 24.54995 | -3.5320606 | 0 | probable pectinesterase pectinesterase inhibitor 54 includes: ame: full=pectinesterase inhibitor 54 ame: full=pectin methylesterase inhibitor 54 includes: ame: full=pectinesterase 54� |
| EY675570 | S44224836 | 1 | 7 | 0.855399 | 8.183316 | 3.258016331 | 0 | rna-binding protein 38 ame: full=rna-binding motif protein 38 ame: full=rna-binding region-containing protein 1 ame: full=hsrnaseb ame: full=ssdna-binding protein seb4 ame: full=cll-associated antigen kw-5 |
| EY675698 | S44224964 | 262 | 575 | 224.1145 | 672.201 | 1.584656553 | 0 | cytochrome p450 98a1 |
| EY675709 | S44224975 | 33 | 6 | 28.22816 | 7.014271 | -2.00877021 | 0 | flavoprotein wrba |
| EY675876 | S44225142 | 78 | 1208 | 66.72112 | 1412.207 | 4.403663929 | 0 | probable wrky transcription factor 70 ame: full=wrky dna-binding protein 70 |
| EY675878 | S44225144 | 118 | 141 | 100.9371 | 164.8354 | 0.707569712 | 0 | probable receptor-like protein kinase at5g39020 flags: precursor |
| EY675883 | S44225149 | 2469 | 5696 | 2111.98 | 6658.881 | 1.656683719 | 0 | protein |
| EY675909 | S44225175 | 183 | 42 | 156.538 | 49.0999 | -1.672721006 | 0 | protein |
| EY675953 | S44225219 | 114 | 564 | 97.51548 | 659.3415 | 2.757322747 | 0 | mouse dna sequence from clone rp23-279j20 on chromosome complete sequence |
| EY676026 | S44225292 | 109 | 12 | 93.23848 | 14.02854 | -2.732560415 | 0 | protein |
| EY676054 | S44225320 | 196 | 37 | 167.6582 | 43.25467 | -1.954595069 | 0 | cs00-c1-401-018-b09- sweet orange infected with citrus sinensis mrna |
| EY676079 | S44225345 | 63 | 145 | 53.89013 | 169.5116 | 1.653290576 | 0 | protein |
| EY676106 | S44225372 | 99 | 287 | 84.68449 | 335.516 | 1.986211716 | 0 | predicted protein [Populus trichocarpa] |
| EY676162 | S44225428 | 24 | 127 | 20.52957 | 148.4687 | 2.854383595 | 0 | length cdna complete sequence from clone gsltsil77zb11 of silique of strain col-0 of arabidopsis thaliana (thale cress) |
| EY676272 | S44225538 | 10 | 0 | 8.553989 | 0 | -Inf | 0 | gdsl esterase lipase at5g33370 ame: full=extracellular lipase at5g33370 flags: precursor |
| EY676463 | S44225617 | 22 | 62 | 18.81878 | 72.4808 | 1.945426101 | 0 | protein |
| EY676495 | S44225649 | 2054 | 6725 | 1756.989 | 7861.829 | 2.161759495 | 0 | conserved hypothetical protein [Ricinus communis] |
| EY676504 | S44225658 | 670 | 1852 | 573.1173 | 2165.072 | 1.917512507 | 0 | protein |
| EY676555 | S44225709 | 17 | 41 | 14.54178 | 47.93085 | 1.720750572 | 0 | disease resistance protein rps2 ame: full=resistance to pseudomonas syringae protein 2 |
| EY676565 | S44225719 | 3 | 15 | 2.566197 | 17.53568 | 2.772589504 | 0 | serine carboxypeptidase-like 14 flags: precursor |
| EY676719 | S44225873 | 4 | 509 | 3.421596 | 595.044 | 7.442183255 | 0 | aspartic proteinase nepenthesin-1 ame: full=nepenthesin-i flags: precursor |
| EY676749 | S44225903 | 22 | 4 | 18.81878 | 4.676181 | -2.00877021 | 0 | cs00-c1-401-026-d09- sweet orange infected with citrus sinensis mrna |
| EY676784 | S44225938 | 429 | 55 | 366.9661 | 64.29749 | -2.512812715 | 0 | tsd2 (tumorous shoot development 2) methyltransferase |
| EY677115 | S44226045 | 2361 | 4940 | 2019.597 | 5775.083 | 1.515774409 | 0 | lipoxygenase chloroplastic� |
| EY677289 | S44226219 | 13 | 28 | 11.12019 | 32.73327 | 1.557576613 | 0 | basic 7s globulin ame: full=sbg7s� |
| EY677619 | S44226437 | 167 | 393 | 142.8516 | 459.4348 | 1.685342619 | 0 | uncharacterized acetyltransferase at3g50280 |
| EY677633 | S44226451 | 2200 | 436 | 1881.878 | 509.7037 | -1.884442075 | 0 | fam10 family protein at4g22670 |
| EY677645 | S44226463 | 1198 | 258 | 1024.768 | 301.6137 | -1.764523528 | 0 | ankyrin-like protein |
| EY677716 | S44226534 | 119 | 365 | 101.7925 | 426.7015 | 2.067596299 | 0 | protein |
| EY677736 | S44226554 | 73 | 19 | 62.44412 | 22.21186 | -1.491235636 | 0 | protein tyrosine phosphatase-like protein ptplad1 ame: full=protein-tyrosine phosphatase-like a domain-containing protein 1 |
| EY677792 | S44226610 | 51 | 178 | 43.62535 | 208.09 | 2.253969498 | 0 | cysteine-rich receptor-like protein kinase 5� |
| EY677909 | S44226727 | 88 | 13 | 75.27511 | 15.19759 | -2.308330491 | 0 | taxadien-5-alpha-ol o-acetyltransferase ame: full=taxa-4 -dien-5alpha-ol-o-acetyltransferase� |
| EY677919 | S44226737 | 414 | 97 | 354.1352 | 113.3974 | -1.642912706 | 0 | glycosyltransferase quasimodo1 |
| EY677942 | S44226760 | 160 | 456 | 136.8638 | 533.0846 | 1.961623328 | 0 | aspartic proteinase asp1� |
| EY677956 | S44226774 | 187 | 433 | 159.9596 | 506.1966 | 1.661990164 | 0 | squalene monooxygenase ame: full=squalene epoxidase� |
| EY678053 | S44226871 | 1737 | 396 | 1485.828 | 462.9419 | -1.68236401 | 0 | eugenol o-methyltransferase ame: full= eugenol o-methyltransferase eomt1 ame: full=s-adenosysl-l-methionine: eugenol o-methyltransferase eomt1 |
| EY678059 | S44226877 | 60 | 140 | 51.32394 | 163.6663 | 1.67305383 | 0 | ac007727\_10 ests gb |
| EY678093 | S44226911 | 23 | 53 | 19.67418 | 61.9594 | 1.655019908 | 0 | caffeic acid 3-o-methyltransferase ame: full=s-adenosysl-l-methionine:caffeic acid 3-o-methyltransferase� |
| EY678170 | S44226988 | 501 | 1131 | 428.5549 | 1322.19 | 1.62537783 | 0 | phosphatidylglycerol specific phospholipase c |
| EY678176 | S44226994 | 329 | 72 | 281.4262 | 84.17125 | -1.741357363 | 0 | serine carboxypeptidase-like 27 flags: precursor |
| EY678203 | S44227021 | 2287 | 7097 | 1956.297 | 8296.714 | 2.08441435 | 0 | sigma factor sigb regulation protein rsbq |
| EY678390 | S44227096 | 20 | 0 | 17.10798 | 0 | -Inf | 0 | aphanomyces euteiches cdna |
| EY678462 | S44227168 | 42 | 0 | 35.92675 | 0 | -Inf | 0 | gdsl esterase lipase at5g45910 ame: full=extracellular lipase at5g45910 flags: precursor |
| EY678471 | S44227177 | 738 | 1781 | 631.2844 | 2082.069 | 1.721656204 | 0 | nematode-resistance protein |
| EY678518 | S44227224 | 91 | 13 | 77.8413 | 15.19759 | -2.356693513 | 0 | formin-like protein 1� |
| EY678531 | S44227237 | 49 | 7 | 41.91455 | 8.183316 | -2.356693513 | 0 | glucomannan 4-beta-mannosyltransferase 2 ame: full=glucomannan synthase ame: full=mannan synthase 2 ame: full=cellulose synthase-like protein a2� |
| EY678627 | S44227333 | 16 | 1 | 13.68638 | 1.169045 | -3.549338591 | 0 | uncharacterized protein at5g43822 |
| EY678647 | S44227353 | 56 | 183 | 47.90234 | 213.9353 | 2.159006325 | 0 | cs00-c1-401-050-g06- sweet orange infected with citrus sinensis mrna |
| EY678713 | S44227419 | 42 | 82 | 35.92675 | 95.86171 | 1.415895991 | 0 | l-aminoadipate-semialdehyde dehydrogenase-phosphopantetheinyl transferase ame: full=4 -phosphopantetheinyl transferase ame: full=alpha-aminoadipic semialdehyde dehydrogenase-phosphopantetheinyl transferase� |
| EY678786 | S44227492 | 5 | 56 | 4.276995 | 65.46653 | 3.936088236 | 0 | superoxide dismutase |
| EY678804 | S44227510 | 1861 | 389 | 1591.897 | 454.7586 | -1.807574586 | 0 | probable pectinesterase pectinesterase inhibitor 34 includes: ame: full=pectinesterase inhibitor 34 ame: full=pectin methylesterase inhibitor 34 includes: ame: full=pectinesterase 34� |
| EY679298 | S44227892 | 11 | 1 | 9.409388 | 1.169045 | -3.00877021 | 0 | pmr5 (powdery mildew resistant 5) |
| EY679306 | S44227900 | 3 | 199 | 2.566197 | 232.64 | 6.502323529 | 0 | predicted protein [Populus trichocarpa] |
| EY679334 | S44227928 | 85 | 172 | 72.70891 | 201.0758 | 1.467535228 | 0 | probable adp-ribosylation factor gtpase-activating protein agd5� |
| EY679337 | S44227931 | 2 | 13 | 1.710798 | 15.19759 | 3.151101127 | 0 | cs00-c1-401-059-a12- sweet orange infected with citrus sinensis mrna |
| EY679388 | S44227982 | 1 | 114 | 0.855399 | 133.2712 | 7.283551423 | 0 | aspartic proteinase nepenthesin-1 ame: full=nepenthesin-i flags: precursor |
| EY679457 | S44228051 | 9 | 19 | 7.69859 | 22.21186 | 1.528663921 | 0 | cs00-c1-401-057-d08- sweet orange infected with citrus sinensis mrna |
| EY679466 | S44228060 | 196 | 428 | 167.6582 | 500.3513 | 1.577418551 | 0 | probable wrky transcription factor 2 ame: full=wrky dna-binding protein 2 |
| EY679601 | S44228195 | 8 | 2 | 6.843191 | 2.33809 | -1.549338591 | 0 | cs00-c1-650-002-a04- sweet orange young greenhouse plant citrus sinensis mrna |
| EY679905 | S44228387 | 868 | 98 | 742.4863 | 114.5664 | -2.696179979 | 0 | aquaporin |
| EY680148 | S44228630 | 132 | 16 | 112.9127 | 18.70472 | -2.59373271 | 0 | aquaporin tip2-1 ame: full=tonoplast intrinsic protein 2-1� |
| EY680355 | S44228725 | 1214 | 187 | 1038.454 | 218.6115 | -2.247996837 | 0 | cs00-c1-650-010-b11- sweet orange young greenhouse plant citrus sinensis mrna |
| EY680705 | S44228963 | 248 | 655 | 212.1389 | 765.7246 | 1.851816195 | 0 | receptor-like protein kinase 5 ame: full=protein haesa flags: precursor |
| EY681202 | S44229460 | 21 | 38 | 17.96338 | 44.42372 | 1.3062715 | 0 | protein |
| EY681560 | S44229706 | 574 | 1115 | 490.999 | 1303.485 | 1.408582477 | 0 | f-box family protein |
| EY681849 | S44229771 | 20 | 44 | 17.10798 | 51.43799 | 1.588164933 | 0 | protein |
| EY681898 | S44229820 | 226 | 798 | 193.3202 | 932.8981 | 2.270727383 | 0 | probable beta- -galactosyltransferase 18 |
| EY682146 | S44229956 | 1159 | 239 | 991.4074 | 279.4018 | -1.827136634 | 0 | adiponectin receptor protein 2 ame: full=progestin and adipoq receptor family member ii |
| EY682492 | S44230190 | 43 | 10 | 36.78215 | 11.69045 | -1.653675251 | 0 | �pentatricopeptide repeat-containing protein at1g19290 |
| EY682582 | S44230280 | 873 | 194 | 746.7633 | 226.7948 | -1.719263592 | 0 | cs00-c1-650-034-d09- sweet orange young greenhouse plant citrus sinensis mrna |
| EY682772 | S44230470 | 18 | 84 | 15.39718 | 98.1998 | 2.67305383 | 0 | at3g15630 msj11\_3 |
| EY683741 | S44230991 | 41 | 112 | 35.07136 | 130.9331 | 1.900464326 | 0 | calcineurin b-like protein 10 |
| EY683784 | S44231034 | 57 | 167 | 48.75774 | 195.2305 | 2.001475687 | 0 | cs00-c1-650-040-f04- sweet orange young greenhouse plant citrus sinensis mrna |
| EY683832 | S44231082 | 56 | 123 | 47.90234 | 143.7926 | 1.585820992 | 0 | �zinc metalloprotease slr1821 |
| EY683840 | S44231090 | 74 | 15 | 63.29952 | 17.53568 | -1.851901361 | 0 | omega-6 fatty acid endoplasmic reticulum isozyme 2 |
| EY683842 | S44231092 | 259 | 51 | 221.5483 | 59.6213 | -1.893721537 | 0 | cs00-c1-650-041-c06- sweet orange young greenhouse plant citrus sinensis mrna |
| EY684273 | S44231187 | 17 | 47 | 14.54178 | 54.94512 | 1.917787419 | 0 | protein ruptured pollen grain 1 |
| EY684276 | S44231190 | 28 | 67 | 23.95117 | 78.32603 | 1.709395677 | 0 | yth domain family protein 2 |
| EY684404 | S44231318 | 16 | 2 | 13.68638 | 2.33809 | -2.549338591 | 0 | aspartic proteinase nepenthesin-2 ame: full=nepenthesin-ii flags: precursor |
| EY684470 | S44231384 | 37 | 102 | 31.64976 | 119.2426 | 1.913633385 | 0 | protein |
| EY684523 | S44231437 | 0 | 9 | 0 | 10.52141 | Inf | 0 | cs00-c2-003-001-a12- sweet orange greenhouse plant citrus sinensis mrna |
| EY684552 | S44231466 | 147 | 44 | 125.7436 | 51.43799 | -1.289579317 | 0 | probable inactive receptor kinase at5g58300 flags: precursor |
| EY684567 | S44231481 | 171 | 13 | 146.2732 | 15.19759 | -3.266751388 | 0 | Protein E6, putative [Ricinus communis] |
| EY684834 | S44231748 | 1 | 3 | 0.855399 | 3.507136 | 2.03562391 | 0 | mads box |
| EY684843 | S44231757 | 933 | 21 | 798.0872 | 24.54995 | -5.022754439 | 0 | monothiol glutaredoxin-s9� |
| EY684964 | S44231878 | 37 | 4 | 31.64976 | 4.676181 | -2.758791957 | 0 | 1-acyl-sn-glycerol-3-phosphate acyltransferase� |
| EY685054 | S44231968 | 123 | 241 | 105.2141 | 281.7399 | 1.42103624 | 0 | beta-amylase ame: full= -alpha-d-glucan maltohydrolase |
| EY685065 | S44231979 | 28 | 4 | 23.95117 | 4.676181 | -2.356693513 | 0 | anaeromyxobacter fw109- complete genome |
| EY685097 | S44232011 | 47 | 11 | 40.20375 | 12.8595 | -1.644495824 | 0 | cs00-c2-003-008-g01- sweet orange greenhouse plant citrus sinensis mrna |
| EY685151 | S44232065 | 24 | 72 | 20.52957 | 84.17125 | 2.03562391 | 0 | glutaredoxin-c6� |
| EY685389 | S44232303 | 550 | 77 | 470.4694 | 90.01648 | -2.385839859 | 0 | cytokinin-o-glucosyltransferase 2 ame: full=zeatin o-glucosyltransferase 2� |
| EY685391 | S44232305 | 32 | 5 | 27.37277 | 5.845226 | -2.227410496 | 0 | cs00-c2-003-018-f09- sweet orange greenhouse plant citrus sinensis mrna |
| EY685399 | S44232313 | 26 | 3 | 22.24037 | 3.507136 | -2.664815808 | 0 | cs00-c2-003-018-g06- sweet orange greenhouse plant citrus sinensis mrna |
| EY685417 | S44232331 | 166 | 418 | 141.9962 | 488.6609 | 1.78298111 | 0 | nuclear transcription x-box |
| EY685437 | S44232351 | 3277 | 496 | 2803.142 | 579.8464 | -2.273302238 | 0 | cs00-c2-003-019-c01- sweet orange greenhouse plant citrus sinensis mrna |
| EY685507 | S44232421 | 6 | 20 | 5.132394 | 23.3809 | 2.187627003 | 0 | protein |
| EY685751 | S44232581 | 586 | 115 | 501.2638 | 134.4402 | -1.898605394 | 0 | cs00-c2-003-025-g04- sweet orange greenhouse plant citrus sinensis mrna |
| EY685784 | S44232614 | 18 | 41 | 15.39718 | 47.93085 | 1.638288412 | 0 | protein |
| EY685877 | S44232707 | 51 | 0 | 43.62535 | 0 | -Inf | 0 | gdsl esterase lipase at2g04570 ame: full=extracellular lipase at2g04570 flags: precursor |
| EY685940 | S44232770 | 50 | 7 | 42.76995 | 8.183316 | -2.385839859 | 0 | cs00-c2-003-029-d10- sweet orange greenhouse plant citrus sinensis mrna |
| EY685976 | S44232806 | 365 | 49 | 312.2206 | 57.28321 | -2.446381401 | 0 | cs00-c2-003-029-h06- sweet orange greenhouse plant citrus sinensis mrna |
| EY685993 | S44232823 | 64 | 15 | 54.74553 | 17.53568 | -1.642447995 | 0 | glycosyltransferase 8 domain-containing protein 1 |
| EY686272 | S44232984 | 0 | 2 | 0 | 2.33809 | Inf | 0 | cs00-c2-003-041-h01- sweet orange greenhouse plant citrus sinensis mrna |
| EY686406 | S44233118 | 408 | 88 | 349.0028 | 102.876 | -1.762332314 | 0 | glycosyltransferase quasimodo1 |
| EY686452 | S44233164 | 112 | 15 | 95.80468 | 17.53568 | -2.449802917 | 0 | thaumatin-like protein 1 flags: precursor |
| EY686493 | S44233205 | 13 | 2 | 11.12019 | 2.33809 | -2.249778309 | 0 | cs00-c2-003-054-a11- sweet orange greenhouse plant citrus sinensis mrna |
| EY686549 | S44233261 | 49 | 8 | 41.91455 | 9.352362 | -2.164048435 | 0 | serine-threonine protein plant- |
| EY687063 | S44233327 | 4 | 11 | 3.421596 | 12.8595 | 1.910093028 | 0 | cs00-c2-003-036-g03- sweet orange greenhouse plant citrus sinensis mrna |
| EY687123 | S44233387 | 680 | 122 | 581.6713 | 142.6235 | -2.02799219 | 0 | cs00-c2-003-037-f01- sweet orange greenhouse plant citrus sinensis mrna |
| EY687170 | S44233434 | 623 | 101 | 532.9135 | 118.0736 | -2.174215461 | 0 | anthranilate n-benzoyltransferase |
| EY687309 | S44233573 | 32 | 5 | 27.37277 | 5.845226 | -2.227410496 | 0 | nodulin 21 family protein |
| EY687900 | S44233968 | 95 | 197 | 81.2629 | 230.3019 | 1.50285762 | 0 | cs00-c2-003-050-c06- sweet orange greenhouse plant citrus sinensis mrna |
| EY688092 | S44234048 | 163 | 39 | 139.43 | 45.59276 | -1.612664526 | 0 | cysteine-rich receptor-like protein kinase 24� |
| EY688198 | S44234154 | 1449 | 379 | 1239.473 | 443.0681 | -1.484126432 | 0 | vitis vinifera contig whole genome shotgun sequence |
| EY688205 | S44234161 | 43 | 2 | 36.78215 | 2.33809 | -3.975603346 | 0 | probable lrr receptor-like serine threonine-protein kinase at4g37250 flags: precursor |
| EY689302 | S44234810 | 349 | 73 | 298.5342 | 85.3403 | -1.806597258 | 0 | calreticulin-3 flags: precursor |
| EY689397 | S44234905 | 36 | 6 | 30.79436 | 7.014271 | -2.134301092 | 0 | �kda class i heat shock protein 1 ame: full=heat shock protein ame: full=hsp ame: full=heat shock protein 17 ame: full=low molecular weight heat shock protein |
| EY689419 | S44234927 | 27 | 5 | 23.09577 | 5.845226 | -1.982297998 | 0 | populus trichocarpa mrna |
| EY689618 | S44235028 | 8 | 16 | 6.843191 | 18.70472 | 1.450661409 | 0 | cs00-c2-003-078-a07- sweet orange greenhouse plant citrus sinensis mrna |
| EY689740 | S44235150 | 136 | 260 | 116.3343 | 303.9518 | 1.385566381 | 0 | protein |
| EY689747 | S44235157 | 11 | 1 | 9.409388 | 1.169045 | -3.00877021 | 0 | cs00-c2-003-079-f12- sweet orange greenhouse plant citrus sinensis mrna |
| EY689806 | S44235216 | 142 | 328 | 121.4666 | 383.4468 | 1.658466294 | 0 | poncirus trifoliata citrus tristeza virus resistance gene complete sequence |
| EY689845 | S44235255 | 24 | 2 | 20.52957 | 2.33809 | -3.134301092 | 0 | protein |
| EY689900 | S44235310 | 159 | 34 | 136.0084 | 39.74754 | -1.774758705 | 0 | cs00-c2-003-081-f12- sweet orange greenhouse plant citrus sinensis mrna |
| EY689984 | S44235394 | 245 | 36 | 209.5727 | 42.08563 | -2.316051529 | 0 | vitis vinifera contig whole genome shotgun sequence |
| EY690022 | S44235432 | 115 | 32 | 98.37088 | 37.40945 | -1.394828642 | 0 | o-acetyltransferase family protein |
| EY690744 | S44235930 | 70 | 9 | 59.87792 | 10.52141 | -2.508696606 | 0 | expression vector complete sequence |
| EY690884 | S44236070 | 433 | 86 | 370.3877 | 100.5379 | -1.881297051 | 0 | fasciclin-like arabinogalactan protein 15 flags: precursor |
| EY691064 | S44236250 | 2 | 7 | 1.710798 | 8.183316 | 2.258016331 | 0 | phosphoinositide phospholipase c 6 ame: full=phosphoinositide phospholipase plc6� |
| EY691433 | S44236619 | 17143 | 2924 | 14664.1 | 3418.288 | -2.100942976 | 0 | homo sapiens chromosome 21 segment hs21c100 |
| EY691434 | S44236620 | 44 | 2 | 37.63755 | 2.33809 | -4.00877021 | 0 | citrus sinensis dna binding protein (v03-3) complete cds |
| EY691566 | S44236752 | 8 | 0 | 6.843191 | 0 | -Inf | 0 | peroxidase 52� |
| EY691949 | S44236813 | 1367 | 83 | 1169.33 | 97.03075 | -3.591096687 | 0 | burp domain-containing protein 6� |
| EY692005 | S44236869 | 3044 | 142 | 2603.834 | 166.0044 | -3.971344115 | 0 | cs00-c2-003-086-d08- sweet orange greenhouse plant citrus sinensis mrna |
| EY692040 | S44236904 | 4 | 14 | 3.421596 | 16.36663 | 2.258016331 | 0 | cs00-c2-003-086-h11- sweet orange greenhouse plant citrus sinensis mrna |
| EY692051 | S44236915 | 32 | 1 | 27.37277 | 1.169045 | -4.549338591 | 0 | dna binding protein |
| EY692190 | S44237054 | 390 | 876 | 333.6056 | 1024.084 | 1.618118155 | 0 | cs00-c2-003-098-a02- sweet orange greenhouse plant citrus sinensis mrna |
| EY692201 | S44237065 | 78 | 14 | 66.72112 | 16.36663 | -2.027385888 | 0 | omega-3 fatty acid endoplasmic reticulum |
| EY692352 | S44237216 | 29583 | 3778 | 25305.27 | 4416.653 | -2.518412347 | 0 | snakin-2 flags: precursor |
| EY692375 | S44237239 | 193 | 33 | 165.092 | 38.57849 | -2.097401509 | 0 | citrus hybrid cultivar starch phosphorylase type h complete cds |
| EY693207 | S44237511 | 199 | 380 | 170.2244 | 444.2372 | 1.383892397 | 0 | glutamate receptor� |
| EY693432 | S44237624 | 30 | 0 | 25.66197 | 0 | -Inf | 0 | probable inactive receptor kinase at5g67200 flags: precursor |
| EY693559 | S44237751 | 73 | 15 | 62.44412 | 17.53568 | -1.832272554 | 0 | protein |
| EY693577 | S44237769 | 6 | 770 | 5.132394 | 900.1648 | 7.454413544 | 0 | probable indole-3-acetic acid-amido synthetase ame: full=auxin-responsive gh3-like protein 1� |
| EY693754 | S44237834 | 3751 | 1607 | 3208.601 | 1878.656 | -0.772243925 | 0 | cs00-c3-700-018-a08- sweet orange development stadium (1 of 6) citrus sinensis mrna |
| EY693806 | S44237886 | 19 | 134 | 16.25258 | 156.6521 | 3.268823086 | 0 | protein |
| EY693990 | S44238070 | 11 | 2 | 9.409388 | 2.33809 | -2.00877021 | 0 | cs00-c3-700-020-f11- sweet orange development stadium (1 of 6) citrus sinensis mrna |
| EY694359 | S44238341 | 3 | 17 | 2.566197 | 19.87377 | 2.95316175 | 0 | serine threonine-protein kinase bri1-like 2 ame: full=brassinosteroid insensitive 1-like protein 2 ame: full=protein vascular highway 1 flags: precursor |
| EY694708 | S44238578 | 88 | 15 | 75.27511 | 17.53568 | -2.101879614 | 0 | purple acid phosphatase 15 ame: full=phytase flags: precursor |
| EY694770 | S44238640 | 156 | 315 | 133.4422 | 368.2492 | 1.464467209 | 0 | at4g02010 t10m13\_2 |
| EY695310 | S44238844 | 11 | 2 | 9.409388 | 2.33809 | -2.00877021 | 0 | cs00-c3-700-035-a10- sweet orange development stadium (1 of 6) citrus sinensis mrna |
| EY695365 | S44238899 | 12 | 1 | 10.26479 | 1.169045 | -3.134301092 | 0 | PREDICTED: hypothetical protein [Vitis vinifera] |
| EY695384 | S44238918 | 161 | 364 | 137.7192 | 425.5325 | 1.627539171 | 0 | probable protein phosphatase 2c 49� |
| EY695607 | S44239029 | 366 | 58 | 313.076 | 67.80462 | -2.207057434 | 0 | nucleolar protein gar2-related |
| EY695663 | S44239085 | 320 | 602 | 273.7277 | 703.7652 | 1.362352991 | 0 | glutamate receptor ame: full=ligand-gated ion channel ame: full=ionotropic glutamate receptor glr5 flags: precursor |
| EY695672 | S44239094 | 78 | 8 | 66.72112 | 9.352362 | -2.83474081 | 0 | probable lrr receptor-like serine threonine-protein kinase at2g23950 flags: precursor |
| EY695695 | S44239117 | 42 | 7 | 35.92675 | 8.183316 | -2.134301092 | 0 | probable salt tolerance-like protein at1g75540 |
| EY695697 | S44239119 | 390 | 1330 | 333.6056 | 1554.83 | 2.220541626 | 0 | cs00-c3-700-039-c10- sweet orange development stadium (1 of 6) citrus sinensis mrna |
| EY695699 | S44239121 | 36 | 74 | 30.79436 | 86.50934 | 1.490189773 | 0 | cs00-c3-700-039-c12- sweet orange development stadium (1 of 6) citrus sinensis mrna |
| EY695765 | S44239187 | 114 | 239 | 97.51548 | 279.4018 | 1.518638203 | 0 | receptor-like protein kinase at3g21340 ame: full=leucine-rich repeat receptor-like protein kinase at3g21340 flags: precursor |
| EY695766 | S44239188 | 178 | 23 | 152.261 | 26.88804 | -2.501510066 | 0 | myosin heavy chain |
| EY695997 | S44239419 | 39 | 7 | 33.36056 | 8.183316 | -2.027385888 | 0 | uncharacterized basic helix-loop-helix protein at1g06150 |
| EY696120 | S44239542 | 257 | 56 | 219.8375 | 65.46653 | -1.747608218 | 0 | protein |
| EY696233 | S44239655 | 132 | 14 | 112.9127 | 16.36663 | -2.786377788 | 0 | f-box family protein |
| EY696912 | S44240222 | 15 | 20 | 12.83098 | 23.3809 | 0.865698908 | 0 | protein |
| EY696941 | S44240251 | 30753 | 6537 | 26306.08 | 7642.048 | -1.783365143 | 0 | populus trichocarpa mrna |
| EY696993 | S44240303 | 1531 | 334 | 1309.616 | 390.4611 | -1.745892866 | 0 | cellulose synthase-like protein d3� |
| EY696994 | S44240304 | 699 | 158 | 597.9238 | 184.7091 | -1.694706488 | 0 | protein |
| EY696998 | S44240308 | 23 | 37 | 19.67418 | 43.25467 | 1.136552819 | 0 | cs00-c3-700-054-a09- sweet orange development stadium (1 of 6) citrus sinensis mrna |
| EY697055 | S44240365 | 3 | 19 | 2.566197 | 22.21186 | 3.113626422 | 0 | peroxidase 4 flags: precursor |
| EY697161 | S44240471 | 297 | 30 | 254.0535 | 35.07136 | -2.856767116 | 0 | fiber protein fb34 |
| EY697166 | S44240476 | 21 | 2 | 17.96338 | 2.33809 | -2.941656014 | 0 | myb-related protein pp1 |
| EY697171 | S44240481 | 471 | 51 | 402.8929 | 59.6213 | -2.756496499 | 0 | PREDICTED: hypothetical protein [Vitis vinifera] |
| EY697870 | S44241068 | 508 | 108 | 434.5427 | 126.2569 | -1.783135776 | 0 | ca2+ antiporter cation exchanger |
| EY697902 | S44241100 | 4214 | 515 | 3604.651 | 602.0583 | -2.581884568 | 0 | 3 -n-debenzoyl-2 -deoxytaxol n-benzoyltransferase� |
| EY697936 | S44241134 | 37 | 3 | 31.64976 | 3.507136 | -3.173829456 | 0 | glyoxylate reductase |
| EY697950 | S44241148 | 308 | 673 | 263.4629 | 786.7674 | 1.578337563 | 0 | protein iq-domain 14 |
| EY697963 | S44241161 | 91 | 21 | 77.8413 | 24.54995 | -1.664815808 | 0 | formin-like protein 6� |
| EY697991 | S44241189 | 17 | 1 | 14.54178 | 1.169045 | -3.636801432 | 0 | predicted protein [Populus trichocarpa] |
| EY697996 | S44241194 | 19 | 2 | 16.25258 | 2.33809 | -2.797266104 | 0 | protein |
| EY698022 | S44241220 | 35 | 79 | 29.93896 | 92.35457 | 1.62515914 | 0 | cs00-c3-700-067-a10- sweet orange development stadium (1 of 6) citrus sinensis mrna |
| EY698025 | S44241223 | 34 | 5 | 29.08356 | 5.845226 | -2.314873337 | 0 | probable pectinesterase pectinesterase inhibitor 12 includes: ame: full=pectinesterase inhibitor 12 ame: full=pectin methylesterase inhibitor 12 includes: ame: full=pectinesterase 12� |
| EY698407 | S44241381 | 8 | 29 | 6.843191 | 33.90231 | 2.308642404 | 0 | cs00-c3-700-071-d11- sweet orange development stadium (1 of 6) citrus sinensis mrna |
| EY698534 | S44241508 | 197 | 33 | 168.5136 | 38.57849 | -2.126996291 | 0 | probable xyloglucan glycosyltransferase 8 ame: full=cellulose synthase-like protein c8� |
| EY698899 | S44241873 | 1206 | 270 | 1031.611 | 315.6422 | -1.708537186 | 0 | atp binding |
| EY698931 | S44241905 | 53 | 133 | 45.33614 | 155.483 | 1.77802339 | 0 | conserved hypothetical protein [Ricinus communis] |
| EY698945 | S44241919 | 753 | 2714 | 644.1154 | 3172.789 | 2.30036036 | 0 | ethylene-responsive transcription factor 4 ame: full=ethylene-responsive element-binding factor 4 homolog ame: full=ethylene-responsive element-binding factor 3� |
| EY699143 | S44242117 | 6 | 17 | 5.132394 | 19.87377 | 1.95316175 | 0 | �ac transposase ame: full=orfa |
| EY699595 | S44242457 | 174 | 15 | 148.8394 | 17.53568 | -3.085391491 | 0 | zinc transporter 1 ame: full=zrt irt-like protein 1 flags: precursor |
| EY699625 | S44242487 | 354 | 1390 | 302.8112 | 1624.973 | 2.423925027 | 0 | non-race specific disease resistance 1 |
| EY699678 | S44242540 | 4 | 17 | 3.421596 | 19.87377 | 2.53812425 | 0 | protein |
| EY699737 | S44242599 | 414 | 2017 | 354.1352 | 2357.964 | 2.73516982 | 0 | receptor-like protein kinase feronia ame: full=protein sirene flags: precursor |
| EY699859 | S44242721 | 88 | 2 | 75.27511 | 2.33809 | -5.00877021 | 0 | thymidine kinase |
| EY700022 | S44242884 | 59 | 146 | 50.46854 | 170.6806 | 1.757842919 | 0 | wall-associated receptor kinase-like 2 flags: precursor |
| EY700036 | S44242898 | 8 | 89 | 6.843191 | 104.045 | 3.92639484 | 0 | 2-succinylbenzoate-- ligase ame: full=osb- synthetase ame: full=o-succinylbenzoyl- synthetase |
| EY700264 | S44243126 | 617 | 127 | 527.7811 | 148.4687 | -1.829780583 | 0 | cs00-c3-700-092-e11- sweet orange development stadium (1 of 6) citrus sinensis mrna |
| EY700858 | S44243496 | 345 | 1596 | 295.1126 | 1865.796 | 2.660453794 | 0 | protein |
| EY701155 | S44243681 | 87 | 242 | 74.41971 | 282.9089 | 1.92658115 | 0 | cs00-c3-700-103-g09- sweet orange development stadium (1 of 6) citrus sinensis mrna |
| EY701259 | S44243785 | 104 | 85 | 88.96149 | 99.36884 | 0.159612627 | 0 | cs00-c3-700-109-a10- sweet orange development stadium (1 of 6) citrus sinensis mrna |
| EY701281 | S44243807 | 21 | 1 | 17.96338 | 1.169045 | -3.941656014 | 0 | probable lrr receptor-like serine threonine-protein kinase at5g45780 flags: precursor |
| EY701325 | S44243851 | 627 | 1451 | 536.3351 | 1696.285 | 1.66117158 | 0 | omega-3 fatty acid chloroplastic flags: precursor |
| EY701378 | S44243904 | 131 | 19 | 112.0573 | 22.21186 | -2.334834079 | 0 | uncharacterized membrane protein at1g16860 |
| EY701437 | S44243963 | 83 | 14 | 70.99811 | 16.36663 | -2.1170231 | 0 | cs00-c3-700-106-d04- sweet orange development stadium (1 of 6) citrus sinensis mrna |
| EY701445 | S44243971 | 23 | 5 | 19.67418 | 5.845226 | -1.750972452 | 0 | zinc ribbon 1 |
| EY701476 | S44244002 | 526 | 883 | 449.9398 | 1032.267 | 1.198012047 | 0 | cs00-c3-700-106-g10- sweet orange development stadium (1 of 6) citrus sinensis mrna |
| EY701556 | S44244082 | 32 | 4 | 27.37277 | 4.676181 | -2.549338591 | 0 | arg7\_phaauindole-3-acetic acid-induced protein arg7 |
| EY701685 | S44244211 | 264 | 10 | 225.8253 | 11.69045 | -4.271804615 | 0 | protein |
| EY701744 | S44244270 | 237 | 16 | 202.7295 | 18.70472 | -3.43808184 | 0 | abc transporter g family member 15� |
| EY701822 | S44244348 | 578 | 2060 | 494.4206 | 2408.233 | 2.284164349 | 0 | protein |
| EY701826 | S44244352 | 2230 | 442 | 1907.54 | 516.718 | -1.884264026 | 0 | hydroxyisourate hydrolase� |
| EY701863 | S44244389 | 7 | 16 | 5.987792 | 18.70472 | 1.643306487 | 0 | 4-coumarate-- ligase 3� |
| EY701875 | S44244401 | 30 | 478 | 25.66197 | 558.8036 | 4.444637621 | 0 | probable lrr receptor-like serine threonine-protein kinase at1g56140 flags: precursor |
| EY701880 | S44244406 | 90 | 26 | 76.9859 | 30.39518 | -1.340751969 | 0 | endochitinase flags: precursor |
| EY701986 | S44244512 | 3589 | 722 | 3070.027 | 844.0506 | -1.862849772 | 0 | cytochrome p450 82a4 ame: full=p450 cp9 |
| EY701988 | S44244514 | 228 | 38 | 195.031 | 44.42372 | -2.134301092 | 0 | cs00-c3-700-055-g07- sweet orange development stadium (1 of 6) citrus sinensis mrna |
| EY702045 | S44244571 | 60 | 12 | 51.32394 | 14.02854 | -1.871266686 | 0 | early nodulin-like protein 3 ame: full=phytocyanin-like protein flags: precursor |
| EY702063 | S44244589 | 31601 | 6940 | 27031.46 | 8113.174 | -1.736301236 | 0 | omega-6 fatty acid endoplasmic reticulum ame: full=delta desaturase |
| EY702067 | S44244593 | 941 | 2033 | 804.9304 | 2376.669 | 1.562004996 | 0 | oxygen-evolving enhancer protein chloroplastic� |
| EY702255 | S44244781 | 189 | 643 | 161.6704 | 751.6961 | 2.217093912 | 0 | e3 ubiquitin-protein ligase rglg2 ame: full=ring domain ligase 2 |
| EY702512 | S44244926 | 15 | 36 | 12.83098 | 42.08563 | 1.713695815 | 0 | atp synthase subunit chloroplastic ame: full=f-atpase subunit iv ame: full=atp synthase f0 sector subunit a |
| EY702973 | S44245275 | 221 | 687 | 189.0432 | 803.134 | 2.086925138 | 0 | protein |
| EY703066 | S44245368 | 173 | 377 | 147.984 | 440.73 | 1.574453895 | 0 | metalloendoproteinase 1 ame: full=smep1 flags: precursor |
| EY703099 | S44245401 | 4019 | 3225 | 3437.848 | 3770.171 | 0.133123992 | 0 | medicago truncatula clone mth2- complete sequence |
| EY703128 | S44245430 | 149 | 33 | 127.4544 | 38.57849 | -1.724112992 | 0 | sterol regulatory element-binding protein site 2 |
| EY703257 | S44245559 | 262 | 43 | 224.1145 | 50.26894 | -2.156496838 | 0 | glycosyltransferase 8 domain-containing protein 1 |
| EY703258 | S44245560 | 120 | 28 | 102.6479 | 32.73327 | -1.648874265 | 0 | serine carboxypeptidase-like 26 flags: precursor |
| EY703522 | S44245600 | 99 | 14 | 84.68449 | 16.36663 | -2.371340289 | 0 | zinc finger ccch domain-containing protein 18� |
| EY703774 | S44245852 | 2006 | 7462 | 1715.93 | 8723.415 | 2.345902163 | 0 | ring-h2 finger protein atl5h flags: precursor |
| EY703785 | S44245863 | 88 | 468 | 75.27511 | 547.1132 | 2.86159451 | 0 | receptor-like protein kinase 5 ame: full=protein haesa flags: precursor |
| EY703947 | S44246025 | 30 | 210 | 25.66197 | 245.4995 | 3.258016331 | 0 | receptor-like protein kinase 2 flags: precursor |
| EY704159 | S44246237 | 105 | 159 | 89.81689 | 185.8782 | 1.049298847 | 0 | cs00-c3-701-029-g12- sweet orange development stadium (2 of 6) citrus sinensis mrna |
| EY704766 | S44246732 | 3 | 16 | 2.566197 | 18.70472 | 2.865698908 | 0 | cs00-c3-701-036-c10- sweet orange development stadium (2 of 6) citrus sinensis mrna |
| EY704790 | S44246756 | 623 | 1197 | 532.9135 | 1399.347 | 1.392780493 | 0 | ammonium transporter 1 member 2 ame: full= 1 2 |
| EY704798 | S44246764 | 38 | 146 | 32.50516 | 170.6806 | 2.392558454 | 0 | nudix hydrolase 10� |
| EY704814 | S44246780 | 16 | 2 | 13.68638 | 2.33809 | -2.549338591 | 0 | probable pectinesterase 29� |
| EY704985 | S44246951 | 769 | 100 | 657.8018 | 116.9045 | -2.492322189 | 0 | polygalacturonase-1 non-catalytic subunit beta ame: full= 1 ame: full=polygalacturonase converter� |
| EY704987 | S44246953 | 158 | 379 | 135.153 | 443.0681 | 1.712934699 | 0 | receptor-like protein kinase haiku2 flags: precursor |
| EY705191 | S44247157 | 624 | 1210 | 533.7689 | 1414.545 | 1.406050522 | 0 | protein |
| EY705808 | S44247550 | 609 | 106 | 520.9379 | 123.9188 | -2.071716554 | 0 | ethylene-responsive transcription factor 13� |
| EY706318 | S44248060 | 403 | 782 | 344.7258 | 914.1933 | 1.407050178 | 0 | alpha beta fold family protein |
| EY706402 | S44248144 | 198 | 7 | 169.369 | 8.183316 | -4.371340289 | 0 | major allergen pru ar 1 ame: allergen=pru ar 1 |
| EY706436 | S44248178 | 84 | 426 | 71.85351 | 498.0133 | 2.793053606 | 0 | ethylene-responsive transcription factor 2� |
| EY706443 | S44248185 | 63 | 2 | 53.89013 | 2.33809 | -4.526618514 | 0 | chavicol o-methyltransferase ame: full= eugenol o-methyltransferase cvomt1 ame: full=s-adenosysl-l-methionine: eugenol o-methyltransferase cvomt1 |
| EY706479 | S44248221 | 1879 | 436 | 1607.295 | 509.7037 | -1.656903618 | 0 | probable polygalacturonase� |
| EY706514 | S44248256 | 465 | 3 | 397.7605 | 3.507136 | -6.825462996 | 0 | snakin-1 flags: precursor |
| EY706597 | S44248339 | 1169 | 2393 | 999.9613 | 2797.525 | 1.484206876 | 0 | pentatricopeptide repeat-containing protein at1g18900 |
| EY706709 | S44248451 | 34 | 7 | 29.08356 | 8.183316 | -1.82944651 | 0 | protein |
| EY706810 | S44248552 | 296 | 34 | 253.1981 | 39.74754 | -2.671329115 | 0 | flavanone 7-o-glucoside 2 -o-beta-l-rhamnosyltransferase ame: full= rhamnosyltransferase |
| EY707003 | S44248745 | 18 | 47 | 15.39718 | 54.94512 | 1.835325259 | 0 | protein ruptured pollen grain 1 |
| EY707123 | S44248865 | 51 | 124 | 43.62535 | 144.9616 | 1.732432377 | 0 | at1g03610 f21b7\_13 |
| EY707340 | S44249082 | 1485 | 52 | 1270.267 | 60.79035 | -4.385146089 | 0 | cytochrome p450 76c1 |
| EY707388 | S44249130 | 41 | 4 | 35.07136 | 4.676181 | -2.906890596 | 0 | cs00-c3-701-068-b05- sweet orange development stadium (2 of 6) citrus sinensis mrna |
| EY707544 | S44249286 | 409 | 90 | 349.8582 | 105.2141 | -1.733442528 | 0 | homogentisate phytyltransferase vte2-1 |
| EY708121 | S44249751 | 553 | 56 | 473.0356 | 65.46653 | -2.853119339 | 0 | tubulin beta-2 chain ame: full=beta-2-tubulin |
| EY708222 | S44249852 | 99 | 18 | 84.68449 | 21.04281 | -2.00877021 | 0 | predicted protein [Populus trichocarpa] |
| EY708401 | S44250031 | 214 | 28 | 183.0554 | 32.73327 | -2.483450655 | 0 | dna-damage-inducible protein f |
| EY708789 | S44250209 | 193 | 10 | 165.092 | 11.69045 | -3.819867533 | 0 | cytochrome p450 98a2 |
| EY709707 | S44250804 | 4013 | 11071 | 3432.716 | 12942.5 | 1.914693889 | 0 | �late blight resistance protein homolog r1a-3 |
| EY709806 | S44250903 | 212 | 20 | 181.3446 | 23.3809 | -2.955330951 | 0 | uncharacterized gmc-type oxidoreductase y4nj |
| EY709847 | S44250944 | 909 | 74 | 777.5576 | 86.50934 | -3.16802171 | 0 | myrcene chloroplastic flags: precursor |
| EY709926 | S44251023 | 6 | 12 | 5.132394 | 14.02854 | 1.450661409 | 0 | cs00-c3-701-002-h11- sweet orange development stadium (2 of 6) citrus sinensis mrna |
| EY709989 | S44251086 | 864 | 119 | 739.0647 | 139.1164 | -2.40940833 | 0 | aquaporin pip2-7 ame: full=plasma membrane intrinsic protein 2-7� |
| EY710017 | S44251114 | 992 | 1924 | 848.5557 | 2249.243 | 1.406358182 | 0 | heat stress transcription factor a-4a� |
| EY710037 | S44251134 | 32050 | 1383 | 27415.54 | 1616.79 | -4.083789886 | 0 | germin-like protein subfamily 1 member 8 flags: precursor |
| EY710067 | S44251164 | 148 | 12 | 126.599 | 14.02854 | -3.173829456 | 0 | flavonol sulfotransferase-like ame: full= 47 |
| EY710123 | S44251220 | 6861 | 999 | 5868.892 | 1167.876 | -2.329200874 | 0 | quercetin 3-o-methyltransferase 1� |
| EY710406 | S44251279 | 99 | 1859 | 84.68449 | 2173.255 | 4.681615844 | 0 | probable lrr receptor-like serine threonine-protein kinase at1g56140 flags: precursor |
| EY710466 | S44251339 | 2109 | 388 | 1804.036 | 453.5895 | -1.991769129 | 0 | patatin-05 ame: full=patatin-1-kuras 1 flags: precursor |
| EY710505 | S44251378 | 39 | 98 | 33.36056 | 114.5664 | 1.779969034 | 0 | �upf0481 protein at3g02645 |
| EY710516 | S44251389 | 613 | 72 | 524.3595 | 84.17125 | -2.639156853 | 0 | cytokinin-n-glucosyltransferase 1 ame: full=udp-glucosyl transferase 76c1 |
| EY710574 | S44251447 | 532 | 1144 | 455.0722 | 1337.388 | 1.55525031 | 0 | peroxisomal -2-hydroxy-acid oxidase ame: full=glycolate oxidase� |
| EY710598 | S44251471 | 676 | 111 | 578.2497 | 129.764 | -2.155802161 | 0 | ribulose bisphosphate carboxylase oxygenase activase chloroplastic� |
| EY710632 | S44251505 | 468 | 102 | 400.3267 | 119.2426 | -1.747277969 | 0 | beta-amylase ame: full= -alpha-d-glucan maltohydrolase |
| EY710657 | S44251530 | 11507 | 2051 | 9843.075 | 2397.712 | -2.03745095 | 0 | granule-bound starch synthase chloroplastic amyloplastic ame: full=granule-bound starch synthase i� |
| EY710672 | S44251545 | 84 | 4 | 71.85351 | 4.676181 | -3.941656014 | 0 | f-box family protein |
| EY710689 | S44251562 | 83 | 6 | 70.99811 | 7.014271 | -3.339415522 | 0 | l-ascorbate oxidase homolog ame: full=pollen-specific protein ntp303 flags: precursor |
| EY710697 | S44251570 | 767 | 166 | 656.091 | 194.0615 | -1.757381927 | 0 | homogentisate geranylgeranyl transferase |
| EY710725 | S44251598 | 576 | 48 | 492.7098 | 56.11417 | -3.134301092 | 0 | cs00-c3-701-086-e02- sweet orange development stadium (2 of 6) citrus sinensis mrna |
| EY710905 | S44251666 | 19 | 62 | 16.25258 | 72.4808 | 2.156930206 | 0 | chavicol o-methyltransferase ame: full= eugenol o-methyltransferase cvomt1 ame: full=s-adenosysl-l-methionine: eugenol o-methyltransferase cvomt1 |
| EY710962 | S44251723 | 32305 | 2964 | 27633.66 | 3465.05 | -2.995478713 | 0 | nitrate transporter ame: full=nitrate permease |
| EY711030 | S44251791 | 14 | 38 | 11.97558 | 44.42372 | 1.891234 | 0 | cs00-c3-702-002-h06- sweet orange development stadium (3 of 6) citrus sinensis mrna |
| EY711087 | S44251848 | 30231 | 906 | 25859.56 | 1059.155 | -4.609712433 | 0 | small heat shock chloroplastic flags: precursor |
| EY711963 | S44252290 | 22 | 2 | 18.81878 | 2.33809 | -3.00877021 | 0 | ethylene-responsive transcription factor erf034 |
| EY712018 | S44252345 | 430 | 773 | 367.8215 | 903.6719 | 1.296793163 | 0 | nodule inception protein |
| EY712050 | S44252377 | 0 | 11 | 0 | 12.8595 | Inf | 0 | lrr receptor-like serine threonine-protein kinase efr ame: full=elongation factor tu receptor� |
| EY712132 | S44252459 | 192 | 31 | 164.2366 | 36.2404 | -2.180104781 | 0 | retrotransposable element tf2 155 kda protein type 3 |
| EY712358 | S44252685 | 810 | 192 | 692.8731 | 224.4567 | -1.626154188 | 0 | polyneuridine-aldehyde esterase ame: full=polyneuridine aldehyde esterase flags: precursor |
| EY712436 | S44252763 | 81 | 9 | 69.28731 | 10.52141 | -2.719263592 | 0 | cs00-c3-702-019-c10- sweet orange development stadium (3 of 6) citrus sinensis mrna |
| EY712493 | S44252820 | 262 | 91 | 224.1145 | 106.3831 | -1.074966952 | 0 | single myb histone |
| EY712805 | S44252908 | 34 | 5 | 29.08356 | 5.845226 | -2.314873337 | 0 | cs00-c3-702-023-f07- sweet orange development stadium (3 of 6) citrus sinensis mrna |
| EY712843 | S44252946 | 852 | 1796 | 728.7999 | 2099.605 | 1.526523424 | 0 | nli interacting factor family protein |
| EY712866 | S44252969 | 426 | 849 | 364.3999 | 992.5194 | 1.445572532 | 0 | ring finger and chy zinc finger domain-containing protein 1 ame: full=zinc finger protein 363 ame: full=ch-rich-interacting match with plag1 ame: full=androgen receptor n-terminal-interacting protein ame: full=p53-induced ring-h2 protein� |
| EY713583 | S44253350 | 5759 | 801 | 4926.242 | 936.4052 | -2.395282765 | 0 | glucoamylase ame: full=glucan -alpha-glucosidase ame: full= -alpha-d-glucan glucohydrolase flags: precursor |
| EY713742 | S44253397 | 125 | 15 | 106.9249 | 17.53568 | -2.60823228 | 0 | germin-like protein subfamily 2 member 4 flags: precursor |
| EY713872 | S44253527 | 25 | 49 | 21.38497 | 57.28321 | 1.421515063 | 0 | ribosome biogenesis protein bms1 homolog ame: full=ribosome assembly protein bms1 homolog |
| EY713903 | S44253558 | 91 | 8 | 77.8413 | 9.352362 | -3.057133231 | 0 | anthocyanidin 3-o-glucosyltransferase ame: full=flavonol 3-o-glucosyltransferase ame: full=udp-glucose flavonoid 3-o-glucosyltransferase ame: full=anthocyanin rhamnosyl transferase |
| EY714307 | S44253738 | 3 | 10 | 2.566197 | 11.69045 | 2.187627003 | 0 | cs00-c3-702-040-h06- sweet orange development stadium (3 of 6) citrus sinensis mrna |
| EY714309 | S44253740 | 18 | 0 | 15.39718 | 0 | -Inf | 0 | protein |
| EY714406 | S44253837 | 17 | 87 | 14.54178 | 101.7069 | 2.806142064 | 0 | cs00-c3-702-042-b02- sweet orange development stadium (3 of 6) citrus sinensis mrna |
| EY714543 | S44253974 | 258 | 35 | 220.6929 | 40.91658 | -2.431282829 | 0 | uncharacterized protein at5g01610 |
| EY714614 | S44254045 | 15 | 30 | 12.83098 | 35.07136 | 1.450661409 | 0 | serine threonine protein phosphatase 2a 57 kda regulatory subunit b iota isoform� |
| EY715074 | S44254169 | 4068 | 110 | 3479.763 | 128.595 | -4.758082841 | 0 | endochitinase a� |
| EY715191 | S44254286 | 38 | 65 | 32.50516 | 75.98794 | 1.225101709 | 0 | predicted protein [Populus trichocarpa] |
| EY715444 | S44254315 | 486 | 98 | 415.7239 | 114.5664 | -1.85944125 | 0 | at3g07470 f21o3\_18 |
| EY717749 | S44255626 | 30 | 134 | 25.66197 | 156.6521 | 2.609860004 | 0 | cs00-c3-702-083-b04- sweet orange development stadium (3 of 6) citrus sinensis mrna |
| EY717756 | S44255633 | 110 | 302 | 94.09388 | 353.0516 | 1.907706435 | 0 | ndf6 (ndh dependent flow 6) |
| EY717821 | S44255698 | 33 | 5 | 28.22816 | 5.845226 | -2.271804615 | 0 | wpp domain-associated protein |
| EY718265 | S44255932 | 736 | 108 | 629.5736 | 126.2569 | -2.318013045 | 0 | 6-phospho-beta-galactosidase ame: full=beta-d-phosphogalactoside galactohydrolase� |
| EY718514 | S44256083 | 67 | 10 | 57.31173 | 11.69045 | -2.293499687 | 0 | cs00-c3-702-100-c01- sweet orange development stadium (3 of 6) citrus sinensis mrna |
| EY718617 | S44256186 | 265 | 642 | 226.6807 | 750.527 | 1.727242347 | 0 | cytochrome p450 71a19 |
| EY718638 | S44256207 | 624 | 3308 | 533.7689 | 3867.202 | 2.857002709 | 0 | f-box kelch-repeat protein at2g44130 |
| EY718653 | S44256216 | 645 | 81 | 551.7323 | 94.69266 | -2.542643938 | 0 | farnesylated protein |
| EY718833 | S44256396 | 9815 | 1282 | 8395.74 | 1498.716 | -2.485930597 | 0 | 3 -hydroxy-n-methyl- -coclaurine 4 -o-methyltransferase ame: full=s-adenosyl-l-methionine:3 -hydroxy-n-methylcoclaurine 4 -o-methyltransferase� |
| EY719020 | S44256583 | 577 | 2991 | 493.5652 | 3496.614 | 2.824646095 | 0 | myb-like protein g |
| EY719437 | S44256902 | 23 | 45 | 19.67418 | 52.60703 | 1.418952549 | 0 | abscisic acid 8 -hydroxylase 2� |
| EY719468 | S44256933 | 356 | 58 | 304.522 | 67.80462 | -2.167091027 | 0 | vesicle-associated membrane |
| EY719478 | S44256943 | 20 | 85 | 17.10798 | 99.36884 | 2.53812425 | 0 | zinc finger protein magpie |
| EY719486 | S44256951 | 1074 | 145 | 918.6984 | 169.5116 | -2.438207779 | 0 | cucumisin ame: allergen=cuc m 1 flags: precursor |
| EY719506 | S44256971 | 1208 | 82 | 1033.322 | 95.86171 | -3.430191326 | 0 | fatty acyl- reductase 2 ame: full=fatty acid reductase 2 ame: full=male sterility protein 2 |
| EY719507 | S44256972 | 52762 | 8229 | 45132.56 | 9620.073 | -2.230048815 | 0 | heat shock cognate 70 kda protein 1� |
| EY719828 | S44257293 | 6 | 16 | 5.132394 | 18.70472 | 1.865698908 | 0 | �monooxygenase moxc |
| EY720069 | S44257422 | 94 | 238 | 80.4075 | 278.2328 | 1.790890321 | 0 | f-box kelch-repeat protein at1g26930 |
| EY720079 | S44257432 | 492 | 61 | 420.8563 | 71.31176 | -2.561115759 | 0 | taxadiene 5-alpha hydroxylase |
| EY720455 | S44257696 | 49 | 96 | 41.91455 | 112.2283 | 1.420914066 | 0 | conserved hypothetical protein [Ricinus communis] |
| EY720490 | S44257731 | 597 | 1249 | 510.6732 | 1460.137 | 1.515632049 | 0 | cytochrome p450 76a2 ame: full=cyplxxvia2 ame: full=p-450eg7 |
| EY721129 | S44258364 | 965 | 108 | 825.46 | 126.2569 | -2.708836221 | 0 | myrcene chloroplastic flags: precursor |
| EY721155 | S44258390 | 215 | 38 | 183.9108 | 44.42372 | -2.049603927 | 0 | protein |
| EY721686 | S44258809 | 20 | 40 | 17.10798 | 46.76181 | 1.450661409 | 0 | protein |
| EY722769 | S44259332 | 10 | 68 | 8.553989 | 79.49507 | 3.216196155 | 0 | populus trichocarpa mrna |
| EY722774 | S44259337 | 193 | 406 | 165.092 | 474.6323 | 1.523540289 | 0 | guanosine-3 -bis 3 - |
| EY722802 | S44259365 | 98 | 226 | 83.82909 | 264.2042 | 1.656130527 | 0 | granule-bound starch synthase chloroplastic amyloplastic ame: full=granule-bound starch synthase ii� |
| EY722898 | S44259461 | 50 | 6 | 42.76995 | 7.014271 | -2.60823228 | 0 | pseudomonas fluorescens sbw25 complete genome |
| EY722929 | S44259492 | 5 | 1 | 4.276995 | 1.169045 | -1.871266686 | 0 | 60 kda chaperonin ame: full=protein cpn60 ame: full=groel protein |
| EY722995 | S44259558 | 332 | 53 | 283.9924 | 61.9594 | -2.196457568 | 0 | protein |
| EY723197 | S44259760 | 215 | 23 | 183.9108 | 26.88804 | -2.773969485 | 0 | heavy metal-associated domain containing expressed |
| EY723203 | S44259766 | 280 | 63 | 239.5117 | 73.64985 | -1.701341684 | 0 | interactor of constitutive active rops 3 |
| EY723222 | S44259785 | 271 | 47 | 231.8131 | 54.94512 | -2.076898781 | 0 | protein srg1� |
| EY723374 | S44259937 | 193 | 370 | 165.092 | 432.5467 | 1.389585832 | 0 | af389293\_1 at5g26741 |
| EY723593 | S44260156 | 19 | 37 | 16.25258 | 43.25467 | 1.412187261 | 0 | probable n-acetyltransferase hookless 1 |
| EY724202 | S44260555 | 1698 | 479 | 1452.467 | 559.9726 | -1.375077489 | 0 | 5ng4\_pintaauxin-induced protein 5ng4 |
| EY724756 | S44260997 | 945 | 59 | 808.352 | 68.97367 | -3.550866061 | 0 | cytochrome p450 93a3 ame: full=p450 cp5 |
| EY725089 | S44261218 | 421 | 80 | 360.1229 | 93.52362 | -1.945086919 | 0 | �endonuclease flj39025 homolog |
| EY725606 | S44261617 | 220 | 149 | 188.1878 | 174.1877 | -0.111529784 | 0 | hypothetical protein RCOM\_1431030 [Ricinus communis] |
| EY725739 | S44261652 | 56 | 9 | 47.90234 | 10.52141 | -2.186768512 | 0 | probable lrr receptor-like serine threonine-protein kinase at4g08850 flags: precursor |
| EY726094 | S44262007 | 341 | 720 | 291.691 | 841.7125 | 1.528886576 | 0 | u-box domain-containing protein 29 ame: full=plant u-box protein 29 |
| EY726209 | S44262122 | 75 | 16 | 64.15492 | 18.70472 | -1.778157281 | 0 | unnamed protein product [Vitis vinifera] |
| EY726389 | S44262299 | 3395 | 753 | 2904.079 | 880.291 | -1.722028395 | 0 | upf0717 protein at5g11950 |
| EY726741 | S44262427 | 285 | 827 | 243.7887 | 966.8004 | 1.987586819 | 0 | cytochrome p450 76a1 ame: full=cyplxxvia1 ame: full=p-450eg8 |
| EY727446 | S44262796 | 134 | 22 | 114.6235 | 25.71899 | -2.155996163 | 0 | surface antigen |
| EY727481 | S44262831 | 25 | 56 | 21.38497 | 65.46653 | 1.614160141 | 0 | conserved hypothetical protein [Ricinus communis] |
| EY727509 | S44262859 | 1046 | 214 | 894.7473 | 250.1757 | -1.838538741 | 0 | tsd2 (tumorous shoot development 2) methyltransferase |
| EY727536 | S44262886 | 2487 | 215 | 2127.377 | 251.3447 | -3.081336534 | 0 | flavonoid 3 -hydroxylase 1� |
| EY727543 | S44262893 | 4378 | 175 | 3744.936 | 204.5829 | -4.194183718 | 0 | protein |
| EY727550 | S44262900 | 1449 | 301 | 1239.473 | 351.8826 | -1.816560794 | 0 | 2-oxoisovalerate dehydrogenase subunit mitochondrial ame: full=branched-chain alpha-keto acid dehydrogenase e1 component alpha chain� |
| EY727590 | S44262940 | 1552 | 334 | 1327.579 | 390.4611 | -1.765547141 | 0 | cs00-c3-703-103-b09- sweet orange development stadium (4 of 6) citrus sinensis mrna |
| EY727956 | S44262970 | 170 | 38 | 145.4178 | 44.42372 | -1.710802014 | 0 | cytochrome p450 71b28 |
| EY728036 | S44263050 | 5118 | 567 | 4377.932 | 662.8486 | -2.723498098 | 0 | monooxygenase family protein |
| EY728071 | S44263085 | 495 | 112 | 423.4225 | 130.9331 | -1.693268384 | 0 | protein |
| EY728092 | S44263106 | 119 | 268 | 101.7925 | 313.3041 | 1.621932836 | 0 | msl2 ( -like 2) ion channel |
| EY728096 | S44263110 | 427 | 16 | 365.2553 | 18.70472 | -4.287430851 | 0 | isoflavone-7-o-methyltransferase 9 ame: full=isoflavone-o-methyltransferase 9 ame: full=7 iomt-9 |
| EY728143 | S44263157 | 6 | 19 | 5.132394 | 22.21186 | 2.113626422 | 0 | brassica rapa pekinensis clone complete sequence |
| EY728149 | S44263163 | 39 | 5 | 33.36056 | 5.845226 | -2.512812715 | 0 | cs00-c3-703-108-b05- sweet orange development stadium (4 of 6) citrus sinensis mrna |
| EY728156 | S44263170 | 57 | 145 | 48.75774 | 169.5116 | 1.797680485 | 0 | lysine-specific demethylase 3b ame: full= domain-containing histone demethylation protein 2b ame: full=jumonji domain-containing protein 1b |
| EY728617 | S44263519 | 30 | 74 | 25.66197 | 86.50934 | 1.753224179 | 0 | polyphosphoinositide phosphatase ame: full=phosphatidylinositol -bisphosphate 5-phosphatase ame: full=factor-induced gene 4 protein |
| EY728992 | S44263782 | 62 | 12 | 53.03473 | 14.02854 | -1.918572401 | 0 | at3g08600 f17o14\_7 |
| EY729003 | S44263793 | 19 | 2 | 16.25258 | 2.33809 | -2.797266104 | 0 | cs00-c3-704-011-d02- sweet orange development stadium (5 of 6) citrus sinensis mrna |
| EY729053 | S44263843 | 1435 | 162 | 1227.497 | 189.3853 | -2.69632361 | 0 | hypothetical protein [Vitis vinifera] |
| EY729149 | S44263939 | 1372 | 191 | 1173.607 | 223.2876 | -2.393974529 | 0 | 21 kda protein ame: full= protein flags: precursor |
| EY729348 | S44264138 | 268 | 544 | 229.2469 | 635.9606 | 1.47203506 | 0 | protein kinase chloroplastic flags: precursor |
| EY729359 | S44264149 | 469 | 106 | 401.1821 | 123.9188 | -1.694862249 | 0 | probable e3 ubiquitin-protein ligase rnf217 ame: full=ring finger protein 217 |
| EY729589 | S44264379 | 2038 | 3933 | 1743.303 | 4597.855 | 1.399137544 | 0 | cs00-c3-704-018-g10- sweet orange development stadium (5 of 6) citrus sinensis mrna |
| EY730062 | S44264628 | 1135 | 3613 | 970.8778 | 4223.76 | 2.121166366 | 0 | threonine dehydratase chloroplastic ame: full=threonine deaminase� |
| EY730117 | S44264683 | 18 | 207 | 15.39718 | 241.9924 | 3.974223365 | 0 | f-box and wd40 domain |
| EY731426 | S44265544 | 207 | 430 | 177.0676 | 502.6894 | 1.505367301 | 0 | fyve finger-containing phosphoinositide |
| EY731619 | S44265737 | 12764 | 1697 | 10918.31 | 1983.87 | -2.460360635 | 0 | heat shock protein 101 |
| EY731835 | S44265953 | 17 | 2 | 14.54178 | 2.33809 | -2.636801432 | 0 | ricinus communis beta- mrna |
| EY731853 | S44265971 | 293 | 59 | 250.6319 | 68.97367 | -1.861452396 | 0 | vitis whole genome shotgun contig clone entav 115 |
| EY732565 | S44266571 | 118 | 228 | 100.9371 | 266.5423 | 1.400908374 | 0 | protein |
| EY732591 | S44266597 | 20 | 96 | 17.10798 | 112.2283 | 2.713695815 | 0 | cs00-c3-704-064-b12- sweet orange development stadium (5 of 6) citrus sinensis mrna |
| EY732913 | S44266919 | 678 | 54 | 579.9605 | 63.12844 | -3.199592552 | 0 | �kda heat shock mitochondrial� |
| EY733053 | S44267059 | 130 | 26 | 111.2019 | 30.39518 | -1.871266686 | 0 | lrr receptor-like serine threonine-protein kinase fls2 ame: full=protein flagellin-sensing 2 ame: full=protein flagellin-sensitive 2 flags: precursor |
| EY733167 | S44267173 | 16 | 37 | 13.68638 | 43.25467 | 1.660114775 | 0 | chromatin remodelling complex atpase chain isw- |
| EY733394 | S44267400 | 679 | 92 | 580.8159 | 107.5522 | -2.433044399 | 0 | hydroxyisourate hydrolase� |
| EY734365 | S44268169 | 114 | 86 | 97.51548 | 100.5379 | 0.04403615 | 0 | cs00-c3-704-086-e12- sweet orange development stadium (5 of 6) citrus sinensis mrna |
| EY734601 | S44268293 | 0 | 10 | 0 | 11.69045 | Inf | 0 | cs00-c3-704-104-e01- sweet orange development stadium (5 of 6) citrus sinensis mrna |
| EY734795 | S44268487 | 30328 | 1966 | 25942.54 | 2298.343 | -3.496653726 | 0 | flocculation protein flo11� |
| EY736593 | S44269725 | 4051 | 555 | 3465.221 | 648.8201 | -2.417056999 | 0 | transcription factor bpe ame: full=protein big petal ame: full=transcription factor en 88 ame: full=bhlh transcription factor bhlh031 ame: full=basic helix-loop-helix protein 31� |
| EY736828 | S44269848 | 308 | 813 | 263.4629 | 950.4337 | 1.85098641 | 0 | protein |
| EY737122 | S44269918 | 25 | 4 | 21.38497 | 4.676181 | -2.193194781 | 0 | cs00-c3-705-027-a12- sweet orange development stadium (6 of 6) citrus sinensis mrna |
| EY737138 | S44269934 | 81 | 1 | 69.28731 | 1.169045 | -5.889188594 | 0 | probable 3-beta-hydroxysteroid-delta -isomerase ame: full=cholestenol delta-isomerase ame: full=delta -delta sterol isomerase� |
| EY737358 | S44270148 | 103 | 16 | 88.10609 | 18.70472 | -2.235839118 | 0 | �carrier mitochondrial ame: full=adp atp translocase ame: full=adenine nucleotide translocator� |
| EY737420 | S44270210 | 20 | 42 | 17.10798 | 49.0999 | 1.521050737 | 0 | nac domain-containing protein 4� |
| EY737857 | S44270647 | 387 | 84 | 331.0394 | 98.1998 | -1.753210924 | 0 | cs00-c3-705-036-a06- sweet orange development stadium (6 of 6) citrus sinensis mrna |
| EY738795 | S44271473 | 469 | 76 | 401.1821 | 88.84743 | -2.17485519 | 0 | ent-kaurenoic acid oxidase 2� |
| EY738927 | S44271605 | 3 | 14 | 2.566197 | 16.36663 | 2.67305383 | 0 | �disease resistance rpp13-like protein 3 |
| EY740624 | S44272644 | 1757 | 3622 | 1502.936 | 4234.282 | 1.494333764 | 0 | ethylene-responsive transcription factor erf104 |
| EY740635 | S44272655 | 10 | 29 | 8.553989 | 33.90231 | 1.986714309 | 0 | cs00-c3-705-074-h04- sweet orange development stadium (6 of 6) citrus sinensis mrna |
| EY741041 | S44272949 | 708 | 2395 | 605.6224 | 2799.863 | 2.2088658 | 0 | conserved hypothetical protein [Ricinus communis] |
| EY741259 | S44273167 | 520 | 1006 | 444.8074 | 1176.059 | 1.402708186 | 0 | hypothetical protein [Vitis vinifera] |
| EY741382 | S44273290 | 361 | 1016 | 308.799 | 1187.75 | 1.943491069 | 0 | ent-kaurene oxidase� |
| EY741985 | S44273669 | 17 | 61 | 14.54178 | 71.31176 | 2.293935905 | 0 | photosystem i assembly protein ycf4 |
| EY742526 | S44273986 | 429 | 848 | 366.9661 | 991.3503 | 1.433748026 | 0 | photosystem i reaction center subunit chloroplastic ame: full=photosystem i 20 kda subunit� |
| EY742541 | S44274001 | 2571 | 581 | 2199.231 | 679.2153 | -1.695058132 | 0 | cs00-c3-705-091-c02- sweet orange development stadium (6 of 6) citrus sinensis mrna |
| EY742616 | S44274076 | 241 | 47 | 206.1511 | 54.94512 | -1.907639076 | 0 | lob domain-containing protein 4 ame: full=asymmetric leaves 2-like protein 6� |
| EY742850 | S44274198 | 246 | 508 | 210.4281 | 593.875 | 1.49683159 | 0 | uncharacterized protein c11orf73 homolog |
| EY742851 | S44274199 | 3583 | 551 | 3064.894 | 644.1439 | -2.250382411 | 0 | (+)-delta-cadinene synthase isozyme a� |
| EY742871 | S44274219 | 187 | 20 | 159.9596 | 23.3809 | -2.774304956 | 0 | major allergen pru av 1 ame: full=allergen pru a 1 ame: allergen=pru av 1 |
| EY742919 | S44274267 | 21 | 3 | 17.96338 | 3.507136 | -2.356693513 | 0 | cs00-c3-705-094-g07- sweet orange development stadium (6 of 6) citrus sinensis mrna |
| EY743232 | S44274468 | 1259 | 2457 | 1076.947 | 2872.344 | 1.415280984 | 0 | cs00-c3-705-101-f11- sweet orange development stadium (6 of 6) citrus sinensis mrna |
| EY743868 | S44274544 | 7 | 21 | 5.987792 | 24.54995 | 2.03562391 | 0 | cs00-c3-705-104-a03- sweet orange development stadium (6 of 6) citrus sinensis mrna |
| EY743963 | S44274639 | 490 | 1044 | 419.1455 | 1220.483 | 1.541929467 | 0 | atp-dependent protease la domain-containing protein |
| EY744044 | S44274720 | 2006 | 371 | 1715.93 | 433.7158 | -1.984169105 | 0 | isoflavone-7-o-methyltransferase 9 ame: full=isoflavone-o-methyltransferase 9 ame: full=7 iomt-9 |
| EY744073 | S44274749 | 6508 | 1321 | 5566.936 | 1544.309 | -1.849922375 | 0 | chavicol o-methyltransferase ame: full= eugenol o-methyltransferase cvomt1 ame: full=s-adenosysl-l-methionine: eugenol o-methyltransferase cvomt1 |
| EY744090 | S44274766 | 10 | 22 | 8.553989 | 25.71899 | 1.588164933 | 0 | cs00-c3-705-109-f10- sweet orange development stadium (6 of 6) citrus sinensis mrna |
| EY744415 | S44275091 | 1359 | 88 | 1162.487 | 102.876 | -3.498236713 | 0 | anthocyanin 5-aromatic acyltransferase� |
| EY744477 | S44275153 | 911 | 191 | 779.2684 | 223.2876 | -1.803217007 | 0 | f-box protein skip5 ame: full=skp1-interacting partner 5 |
| EY744529 | S44275205 | 9 | 55 | 7.69859 | 64.29749 | 3.062096121 | 0 | mouse dna sequence from clone rp23-247j12 on chromosome complete sequence |
| EY744610 | S44275286 | 182 | 15 | 155.6826 | 17.53568 | -3.150242636 | 0 | protein |
| EY744692 | S44275368 | 9 | 0 | 7.69859 | 0 | -Inf | 0 | probable xyloglucan endotransglucosylase hydrolase protein 8� |
| EY744925 | S44275503 | 3482 | 11780 | 2978.499 | 13771.35 | 2.20901284 | 0 | early nodulin-like protein 2 ame: full=phytocyanin-like protein flags: precursor |
| EY745106 | S44275684 | 12875 | 1879 | 11013.26 | 2196.636 | -2.325874051 | 0 | probable aquaporin pip1-2 ame: full=plasma membrane intrinsic protein 1-2 ame: full= 1 2 |
| EY745112 | S44275690 | 113 | 364 | 96.66008 | 425.5325 | 2.138277087 | 0 | calcium dependent protein kinase 32 |
| EY745130 | S44275708 | 145 | 34 | 124.0328 | 39.74754 | -1.64178484 | 0 | lustrin a-like |
| EY745147 | S44275725 | 14 | 37 | 11.97558 | 43.25467 | 1.852759853 | 0 | cs00-c5-003-026-d10- sweet orange greenhouse plant citrus sinensis mrna |
| EY745157 | S44275735 | 504 | 35 | 431.1211 | 40.91658 | -3.397335498 | 0 | zinc finger protein constans-like 5 |
| EY745230 | S44275808 | 19 | 3 | 16.25258 | 3.507136 | -2.212303604 | 0 | protein |
| EY745731 | S44276211 | 229 | 55 | 195.8864 | 64.29749 | -1.607182666 | 0 | probable pectate lyase 5 flags: precursor |
| EY745851 | S44276331 | 48 | 10 | 41.05915 | 11.69045 | -1.812372997 | 0 | predicted protein [Populus trichocarpa] |
| EY745902 | S44276377 | 35 | 4 | 29.93896 | 4.676181 | -2.678621608 | 0 | conserved hypothetical protein [Ricinus communis] |
| EY746807 | S44276722 | 138831 | 28803 | 118755.9 | 33672.01 | -1.818377349 | 0 | cs00-c5-003-037-h06- sweet orange greenhouse plant citrus sinensis mrna |
| EY746812 | S44276727 | 1108 | 101 | 947.782 | 118.0736 | -3.004869274 | 0 | pectinesterase pectinesterase inhibitor includes: ame: full=pectinesterase inhibitor ame: full=pectin methylesterase inhibitor includes: ame: full=pectinesterase� |
| EY746830 | S44276745 | 9764 | 876 | 8352.115 | 1024.084 | -3.027808111 | 0 | heat shock 22 kda mitochondrial flags: precursor |
| EY746867 | S44276782 | 917 | 125 | 784.4008 | 146.1306 | -2.42433223 | 0 | solute carrier family 40 member 1 ame: full=ferroportin-1 ame: full=iron-regulated transporter 1 ame: full=metal transporter protein 1� |
| EY746896 | S44276811 | 6114 | 1428 | 5229.909 | 1669.397 | -1.647459164 | 0 | udp-glucuronate 4-epimerase 6 ame: full=udp-glucuronic acid epimerase 6� |
| EY746906 | S44276821 | 17 | 33 | 14.54178 | 38.57849 | 1.407592687 | 0 | iqd33 (iq-domain 33) calmodulin binding |
| EY746939 | S44276854 | 446 | 2487 | 381.5079 | 2907.415 | 2.929952301 | 0 | calcium-dependent protein kinase sk5� |
| EY747045 | S44276960 | 692 | 48 | 591.9361 | 56.11417 | -3.399004318 | 0 | miraculin� |
| EY747295 | S44276986 | 106 | 21 | 90.67229 | 24.54995 | -1.884941623 | 0 | protein |
| EY747349 | S44277040 | 2 | 10 | 1.710798 | 11.69045 | 2.772589504 | 0 | benzoate carboxyl methyltransferase ame: full=s-adenosyl-l-methionine:benzoic acid carboxyl methyltransferase |
| EY747486 | S44277177 | 6 | 29 | 5.132394 | 33.90231 | 2.723679903 | 0 | cs00-c5-003-045-f04- sweet orange greenhouse plant citrus sinensis mrna |
| EY747508 | S44277199 | 2310 | 232 | 1975.972 | 271.2185 | -2.865034732 | 0 | at5g51550 k17n15\_10 |
| EY747562 | S44277253 | 46 | 2 | 39.34835 | 2.33809 | -4.072900547 | 0 | bifunctional enzyme lpxc fabz includes: ame: full=udp-3-o- dehydratase� |
| EY747570 | S44277261 | 6129 | 11782 | 5242.74 | 13773.69 | 1.393522257 | 0 | arginine decarboxylase� |
| EY747587 | S44277278 | 3 | 6 | 2.566197 | 7.014271 | 1.450661409 | 0 | benzoate carboxyl methyltransferase ame: full=s-adenosyl-l-methionine:benzoic acid carboxyl methyltransferase |
| EY747590 | S44277281 | 3555 | 396 | 3040.943 | 462.9419 | -2.715615815 | 0 | expansin-a3� |
| EY747616 | S44277307 | 10 | 0 | 8.553989 | 0 | -Inf | 0 | cs00-c5-003-047-a06- sweet orange greenhouse plant citrus sinensis mrna |
| EY747659 | S44277350 | 125 | 249 | 106.9249 | 291.0923 | 1.444879056 | 0 | probable receptor-like protein kinase at5g59700 flags: precursor |
| EY747662 | S44277353 | 2051 | 285 | 1754.423 | 333.1779 | -2.396632258 | 0 | cell elongation protein diminuto |
| EY747704 | S44277395 | 114 | 17 | 97.51548 | 19.87377 | -2.294765764 | 0 | dna binding protein |
| EY748104 | S44277571 | 11 | 23 | 9.409388 | 26.88804 | 1.514791746 | 0 | cs00-c5-003-053-e07- sweet orange greenhouse plant citrus sinensis mrna |
| EY748618 | S44277749 | 10 | 2 | 8.553989 | 2.33809 | -1.871266686 | 0 | cs00-c5-003-060-f09- sweet orange greenhouse plant citrus sinensis mrna |
| EY748700 | S44277831 | 34 | 277 | 29.08356 | 323.8255 | 3.476940734 | 0 | bahd acyltransferase at5g47980 |
| EY748718 | S44277849 | 153 | 302 | 130.876 | 353.0516 | 1.431678306 | 0 | f-box kelch-repeat protein at1g80440 |
| EY748923 | S44278054 | 195 | 15 | 166.8028 | 17.53568 | -3.249778309 | 0 | cs00-c5-003-064-b03- sweet orange greenhouse plant citrus sinensis mrna |
| EY749305 | S44278114 | 271 | 1375 | 231.8131 | 1607.437 | 2.793728271 | 0 | protein srg1� |
| EY749329 | S44278138 | 136 | 266 | 116.3343 | 310.966 | 1.418481003 | 0 | cs00-c5-003-072-f06- sweet orange greenhouse plant citrus sinensis mrna |
| EY749468 | S44278277 | 176 | 40 | 150.5502 | 46.76181 | -1.686842115 | 0 | vinorine synthase |
| EY749485 | S44278294 | 132 | 13 | 112.9127 | 15.19759 | -2.893292992 | 0 | novel plant |
| EY749875 | S44278460 | 45796 | 1903 | 39173.85 | 2224.693 | -4.138212718 | 0 | cs00-c5-003-080-g09- sweet orange greenhouse plant citrus sinensis mrna |
| EY749940 | S44278525 | 43 | 9 | 36.78215 | 10.52141 | -1.805678344 | 0 | tubulin alpha-2 chain ame: full=alpha-2-tubulin |
| EY750025 | S44278610 | 19 | 1 | 16.25258 | 1.169045 | -3.797266104 | 0 | 14 kda proline-rich protein flags: precursor |
| EY750256 | S44278841 | 36 | 278 | 30.79436 | 324.9946 | 3.39967748 | 0 | monoglyceride lipase� |
| EY750681 | S44278930 | 3 | 33 | 2.566197 | 38.57849 | 3.910093028 | 0 | sigma factor sigb regulation protein rsbq |
| EY750796 | S44279045 | 49 | 8 | 41.91455 | 9.352362 | -2.164048435 | 0 | somatic embryogenesis receptor kinase 2� |
| EY750798 | S44279047 | 625 | 57 | 534.6243 | 66.63558 | -3.004160956 | 0 | tubulin beta-8 chain ame: full=beta-8-tubulin |
| EY750803 | S44279052 | 5136 | 467 | 4393.329 | 545.9441 | -3.008489338 | 0 | l-ascorbate oxidase homolog flags: precursor |
| EY750903 | S44279152 | 200 | 515 | 171.0798 | 602.0583 | 1.815233841 | 0 | udp-glucose 4-epimerase ame: full=udp-galactose 4-epimerase ame: full=galactowaldenase |
| EY750970 | S44279219 | 175 | 13 | 149.6948 | 15.19759 | -3.300109985 | 0 | cs00-c5-003-102-c07- sweet orange greenhouse plant citrus sinensis mrna |
| EY750971 | S44279220 | 50 | 121 | 42.76995 | 141.4545 | 1.725668457 | 0 | beta-amyrin synthase� |
| EY750982 | S44279231 | 272 | 0 | 232.6685 | 0 | -Inf | 0 | gdsl esterase lipase at3g27950 ame: full=extracellular lipase at3g27950 flags: precursor |
| EY751088 | S44279331 | 22 | 43 | 18.81878 | 50.26894 | 1.417494545 | 0 | non-specific lipid-transfer protein� |
| EY751281 | S44279524 | 10 | 20 | 8.553989 | 23.3809 | 1.450661409 | 0 | protein |
| EY751345 | S44279588 | 71 | 224 | 60.73332 | 261.8661 | 2.108269212 | 0 | PREDICTED: hypothetical protein isoform 2 [Vitis vinifera] |
| EY751386 | S44279629 | 19 | 118 | 16.25258 | 137.9473 | 3.085376945 | 0 | homeobox-leucine zipper protein athb-40 ame: full=homeodomain transcription factor athb-40 ame: full=hd-zip protein athb-40 |
| EY751499 | S44279742 | 216 | 526 | 184.7662 | 614.9178 | 1.734692896 | 0 | probable lrr receptor-like serine threonine-protein kinase at1g56130 flags: precursor |
| EY751508 | S44279751 | 5 | 1 | 4.276995 | 1.169045 | -1.871266686 | 0 | mlp-like protein 423 |
| EY751767 | S44279898 | 36 | 176 | 30.79436 | 205.752 | 2.740168026 | 0 | �cysteine-rich receptor-like protein kinase 20� |
| EY751835 | S44279966 | 25 | 5 | 21.38497 | 5.845226 | -1.871266686 | 0 | at3g16570 mgl6\_2 |
| EY751843 | S44279974 | 85 | 6 | 72.70891 | 7.014271 | -3.373767026 | 0 | protein |
| EY751906 | S44280037 | 72 | 203 | 61.58872 | 237.3162 | 1.946072325 | 0 | nad h-quinone oxidoreductase subunit chloroplastic ame: full=nad h dehydrogenase subunit k ame: full=nadh-plastoquinone oxidoreductase subunit k |
| EY752242 | S44280261 | 3 | 14 | 2.566197 | 16.36663 | 2.67305383 | 0 | photoassimilate-responsive protein par-like protein |
| EY752431 | S44280444 | 16 | 2 | 13.68638 | 2.33809 | -2.549338591 | 0 | probable xyloglucan endotransglucosylase hydrolase flags: precursor |
| EY752456 | S44280469 | 106 | 408 | 90.67229 | 476.9704 | 2.395166296 | 0 | galactokinase like protein |
| EY752482 | S44280495 | 143 | 1111 | 122.322 | 1298.809 | 3.408433174 | 0 | geranylgeranyl pyrophosphate synthetase chloroplastic� |
| EY752497 | S44280510 | 96 | 16 | 82.1183 | 18.70472 | -2.134301092 | 0 | 5ng4\_pintaauxin-induced protein 5ng4 |
| EY752777 | S44280706 | 486 | 58 | 415.7239 | 67.80462 | -2.616170099 | 0 | probable inactive purple acid phosphatase 27 flags: precursor |
| EY752900 | S44280829 | 531 | 62 | 454.2168 | 72.4808 | -2.647710331 | 0 | protein |
| EY752992 | S44280921 | 176 | 32 | 150.5502 | 37.40945 | -2.00877021 | 0 | cellulase containing expressed |
| EY753026 | S44280955 | 37 | 122 | 31.64976 | 142.6235 | 2.171945381 | 0 | abc transporter g family member 40� |
| EY753251 | S44281068 | 20 | 122 | 17.10798 | 142.6235 | 3.059470652 | 0 | probable wrky transcription factor 40 ame: full=wrky dna-binding protein 40 |
| EY753520 | S44281337 | 60 | 322 | 51.32394 | 376.4326 | 2.874687692 | 0 | serine threonine-protein kinase bri1-like 2 ame: full=brassinosteroid insensitive 1-like protein 2 ame: full=protein vascular highway 1 flags: precursor |
| EY753636 | S44281453 | 266 | 666 | 227.5361 | 778.5841 | 1.774757341 | 0 | at4g38060 f20d10\_180 |
| EY753771 | S44281588 | 40 | 110 | 34.21596 | 128.595 | 1.910093028 | 0 | protein kinase |
| EY753801 | S44281618 | 254 | 1144 | 217.2713 | 1337.388 | 2.621848059 | 0 | protein |
| EY754123 | S44281716 | 98 | 408 | 83.82909 | 476.9704 | 2.508376907 | 0 | �cysteine-rich receptor-like protein kinase 20� |
| EY754241 | S44281834 | 12 | 32 | 10.26479 | 37.40945 | 1.865698908 | 0 | probable phytol kinase chloroplastic flags: precursor |
| EY754293 | S44281886 | 7 | 41 | 5.987792 | 47.93085 | 3.000858492 | 0 | dynein light chain cytoplasmic ame: full=8 kda dynein light chain |
| EY754300 | S44281893 | 27 | 6 | 23.09577 | 7.014271 | -1.719263592 | 0 | type-b response regulator |
| EY754578 | S44282059 | 124 | 289 | 106.0695 | 337.8541 | 1.671390781 | 0 | �yippee-like protein os10g0369500 |
| EY754908 | S44282193 | 682 | 2622 | 583.3821 | 3065.236 | 2.39348545 | 0 | predicted protein [Populus trichocarpa] |
| EY754929 | S44282214 | 14 | 32 | 11.97558 | 37.40945 | 1.643306487 | 0 | purple acid phosphatase 17 ame: full=acid phosphatase type 5 ame: full=peroxidase flags: precursor |
| EY754945 | S44282230 | 770 | 2353 | 658.6572 | 2750.763 | 2.062232379 | 0 | PREDICTED: hypothetical protein [Vitis vinifera] |
| EY754954 | S44282239 | 18 | 48 | 15.39718 | 56.11417 | 1.865698908 | 0 | cysteine-rich receptor-like protein kinase 22� |
| EY755529 | S44282366 | 311 | 26 | 266.0291 | 30.39518 | -3.129669643 | 0 | predicted protein [Populus trichocarpa] |
| EY755555 | S44282392 | 17 | 24 | 14.54178 | 28.05708 | 0.948161068 | 0 | probable lrr receptor-like serine threonine-protein kinase at3g47570 flags: precursor |
| EY755571 | S44282408 | 173 | 434 | 147.984 | 507.3656 | 1.777584414 | 0 | ring finger protein 43 flags: precursor |
| EY755595 | S44282432 | 26 | 108 | 22.24037 | 126.2569 | 2.505109193 | 0 | cs12-c1-001-030-c09- sweet orange field plant a citrus sinensis mrna |
| EY755717 | S44282442 | 2556 | 6818 | 2186.4 | 7970.55 | 1.866122172 | 0 | protein |
| EY755972 | S44282585 | 22 | 5 | 18.81878 | 5.845226 | -1.686842115 | 0 | vitis vinifera contig whole genome shotgun sequence |
| EY756430 | S44282819 | 2 | 13 | 1.710798 | 15.19759 | 3.151101127 | 0 | cs13-c1-001-008-e05- sweet orange field plant b citrus sinensis mrna |
| EY756541 | S44282930 | 331 | 429 | 283.137 | 501.5204 | 0.82480784 | 0 | populus trichocarpa mrna |
| EY756548 | S44282937 | 10 | 25 | 8.553989 | 29.22613 | 1.772589504 | 0 | generative cell specific-1 |
| EY756570 | S44282959 | 693 | 2449 | 592.7915 | 2862.992 | 2.271926925 | 0 | dna binding |
| EY756842 | S44283119 | 30 | 7 | 25.66197 | 8.183316 | -1.648874265 | 0 | probable inactive receptor kinase at5g58300 flags: precursor |
| EY756883 | S44283160 | 100 | 10 | 85.53989 | 11.69045 | -2.871266686 | 0 | cs13-c1-001-014-g05- sweet orange field plant b citrus sinensis mrna |
| EY757008 | S44283285 | 158 | 387 | 135.153 | 452.4205 | 1.743070417 | 0 | cs13-c1-001-016-c03- sweet orange field plant b citrus sinensis mrna |
| EY757316 | S44283369 | 9 | 22 | 7.69859 | 25.71899 | 1.740168026 | 0 | probable lrr receptor-like serine threonine-protein kinase at2g24230 flags: precursor |
| EY757388 | S44283441 | 71 | 10 | 60.73332 | 11.69045 | -2.377157616 | 0 | arabidopsis halleri mir319a microrna genomic sequence |
| EY757951 | S44283780 | 42 | 84 | 35.92675 | 98.1998 | 1.450661409 | 0 | cs13-c1-001-029-e03- sweet orange field plant b citrus sinensis mrna |
| EY758109 | S44283938 | 954 | 101 | 816.0506 | 118.0736 | -2.788972564 | 0 | monocopper oxidase-like protein sks1 flags: precursor |
| EY758114 | S44283943 | 408 | 2448 | 349.0028 | 2861.823 | 3.03562391 | 0 | zinc finger protein constans-like 2 |
| EY650207 | S44284101 | 125 | 246 | 106.9249 | 287.5851 | 1.42739163 | 0 | iaa-amino acid hydrolase ilr1-like 6 flags: precursor |
| EY650797 | S44284257 | 25 | 4 | 21.38497 | 4.676181 | -2.193194781 | 0 | probable serine threonine-protein kinase at1g54610 |
| EY650814 | S44284274 | 205 | 702 | 175.3568 | 820.6697 | 2.22650853 | 0 | 21 kda protein ame: full= protein flags: precursor |
| EY650832 | S44284292 | 31 | 2 | 26.51737 | 2.33809 | -3.503534901 | 0 | cs00-c1-100-015-c07- sweet orange greenhouse plant citrus sinensis mrna |
| EY650881 | S44284341 | 127 | 33 | 108.6357 | 38.57849 | -1.493629158 | 0 | gdsl esterase lipase at2g42990 ame: full=extracellular lipase at2g42990 flags: precursor |
| EY652027 | S44284689 | 107 | 331 | 91.52768 | 386.954 | 2.079881829 | 0 | probable calcium-binding protein cml44 ame: full=calmodulin-like protein 44 |
| EY652293 | S44284731 | 7089 | 1329 | 6063.923 | 1553.661 | -1.964579616 | 0 | ferric reductase-like transmembrane component |
| EY652344 | S44284782 | 16 | 34 | 13.68638 | 39.74754 | 1.53812425 | 0 | probable protein phosphatase 2c 60� |
| EY653213 | S44284881 | 19 | 40 | 16.25258 | 46.76181 | 1.52466199 | 0 | chromatin modification-related protein meaf6� |
| EY653255 | S44284923 | 412 | 831 | 352.4244 | 971.4766 | 1.462865549 | 0 | atp binding |
| EY653315 | S44284983 | 239 | 526 | 204.4403 | 614.9178 | 1.58871359 | 0 | �phospholipid-transporting atpase 9� |
| EY653480 | S44285036 | 4 | 23 | 3.421596 | 26.88804 | 2.974223365 | 0 | cs00-c1-100-044-h08- sweet orange greenhouse plant citrus sinensis mrna |
| EY653482 | S44285038 | 211 | 406 | 180.4892 | 474.6323 | 1.394898137 | 0 | f-box protein at1g30200 |
| EY653933 | S44285237 | 412 | 800 | 352.4244 | 935.2362 | 1.408017072 | 0 | myb-like protein g |
| EY653967 | S44285271 | 38 | 73 | 32.50516 | 85.3403 | 1.392558454 | 0 | regulator of nonsense |
| EY655127 | S44285423 | 68 | 3 | 58.16713 | 3.507136 | -4.051838932 | 0 | glucan endo- -beta-glucosidase 1 ame: full=(1- |
| EY656636 | S44285742 | 12 | 57 | 10.26479 | 66.63558 | 2.698588922 | 0 | (+)-delta-cadinene synthase isozyme xc14� |
| EY656977 | S44285971 | 4 | 16 | 3.421596 | 18.70472 | 2.450661409 | 0 | cs00-c1-100-060-f12- sweet orange greenhouse plant citrus sinensis mrna |
| EY657105 | S44286001 | 10 | 1 | 8.553989 | 1.169045 | -2.871266686 | 0 | nucleic acid binding |
| EY657499 | S44286087 | 14 | 2 | 11.97558 | 2.33809 | -2.356693513 | 0 | protein |
| EY657532 | S44286120 | 11 | 3 | 9.409388 | 3.507136 | -1.423807709 | 0 | cs00-c1-100-094-a09- sweet orange greenhouse plant citrus sinensis mrna |
| EY658297 | S44286297 | 30 | 0 | 25.66197 | 0 | -Inf | 0 | acyl synthetase |
| EY658484 | S44286386 | 60 | 13 | 51.32394 | 15.19759 | -1.755789468 | 0 | cs00-c1-100-139-h08- sweet orange greenhouse plant citrus sinensis mrna |
| EY658489 | S44286391 | 195 | 38 | 166.8028 | 44.42372 | -1.908741391 | 0 | protein |
| EY658938 | S44286546 | 188 | 44 | 160.815 | 51.43799 | -1.644495824 | 0 | probable lrr receptor-like serine threonine-protein kinase at1g56130 flags: precursor |
| EY659366 | S44286666 | 62 | 8 | 53.03473 | 9.352362 | -2.503534901 | 0 | conserved hypothetical protein [Ricinus communis] |
| EY659391 | S44286691 | 13 | 80 | 11.12019 | 93.52362 | 3.072149786 | 0 | uncharacterized udp-glucosyltransferase at1g05670 |
| EY660034 | S44286788 | 7 | 17 | 5.987792 | 19.87377 | 1.730769328 | 0 | uncharacterized mitochondrial protein g00310 ame: full=orf154 |
| EY660087 | S44286841 | 153 | 11 | 130.876 | 12.8595 | -3.347294815 | 0 | lob domain-containing protein 21 ame: full=asymmetric leaves 2-like protein 12� |
| EY660859 | S44286955 | 10 | 0 | 8.553989 | 0 | -Inf | 0 | auxin-induced protein 6b |
| EY660868 | S44286964 | 1602 | 3467 | 1370.349 | 4053.08 | 1.564475098 | 0 | pathogenesis-related genes transcriptional activator pti5 ame: full=pto-interacting protein 5 |
| EY661135 | S44287133 | 46 | 2 | 39.34835 | 2.33809 | -4.072900547 | 0 | fasciclin-like arabinogalactan protein 11 flags: precursor |
| EY661193 | S44287191 | 19 | 3 | 16.25258 | 3.507136 | -2.212303604 | 0 | cellulose synthase a catalytic subunit 7� |
| EY661436 | S44287210 | 1715 | 929 | 1467.009 | 1086.043 | -0.433796666 | 0 | protein |
| EY661536 | S44287310 | 7 | 19 | 5.987792 | 22.21186 | 1.891234 | 0 | probable lrr receptor-like serine threonine-protein kinase at4g08850 flags: precursor |
| EY662249 | S44287575 | 183 | 29 | 156.538 | 33.90231 | -2.207057434 | 0 | cs00-c1-101-042-a12- sweet orange infected with xylella fastidiosa (stage 1 of 2) citrus sinensis mrna |
| EY662261 | S44287587 | 57 | 3 | 48.75774 | 3.507136 | -3.797266104 | 0 | probable pectinesterase pectinesterase inhibitor 54 includes: ame: full=pectinesterase inhibitor 54 ame: full=pectin methylesterase inhibitor 54 includes: ame: full=pectinesterase 54� |
| EY662278 | S44287604 | 107 | 213 | 91.52768 | 249.0066 | 1.443904043 | 0 | cs00-c1-101-042-d05- sweet orange infected with xylella fastidiosa (stage 1 of 2) citrus sinensis mrna |
| EY662586 | S44287702 | 499 | 1212 | 426.8441 | 1416.883 | 1.730939387 | 0 | neutral invertase |
| EY662635 | S44287751 | 26 | 4 | 22.24037 | 4.676181 | -2.249778309 | 0 | upf0497 membrane protein at4g25830 |
| EY663158 | S44288050 | 10 | 32 | 8.553989 | 37.40945 | 2.128733314 | 0 | protein |
| EY663234 | S44288126 | 3 | 17 | 2.566197 | 19.87377 | 2.95316175 | 0 | cs00-c1-101-053-a03- sweet orange infected with xylella fastidiosa (stage 1 of 2) citrus sinensis mrna |
| EY663264 | S44288156 | 48 | 167 | 41.05915 | 195.2305 | 2.249403201 | 0 | lipoxygenase 1 |
| EY663516 | S44288296 | 374 | 786 | 319.9192 | 918.8695 | 1.522152451 | 0 | probable serine threonine-protein kinase at1g54610 |
| EY663524 | S44288304 | 194 | 413 | 165.9474 | 482.8157 | 1.540746538 | 0 | glutamate receptor ame: full=ligand-gated ion channel flags: precursor |
| EY664186 | S44288420 | 207 | 33 | 177.0676 | 38.57849 | -2.198431429 | 0 | conserved hypothetical protein [Ricinus communis] |
| EY664687 | S44288473 | 49609 | 2314 | 42435.49 | 2705.171 | -3.971479698 | 0 | cs00-c1-101-069-c09- sweet orange infected with xylella fastidiosa (stage 1 of 2) citrus sinensis mrna |
| EY664689 | S44288475 | 28 | 62 | 23.95117 | 72.4808 | 1.597502797 | 0 | s-norcoclaurine synthase flags: precursor |
| EY664724 | S44288510 | 308 | 68 | 263.4629 | 79.49507 | -1.72866229 | 0 | cs00-c1-101-069-g02- sweet orange infected with xylella fastidiosa (stage 1 of 2) citrus sinensis mrna |
| EY664801 | S44288587 | 261 | 39 | 223.2591 | 45.59276 | -2.291842369 | 0 | protein |
| EY664805 | S44288591 | 88 | 1718 | 75.27511 | 2008.42 | 4.737744112 | 0 | probable wrky transcription factor 70 ame: full=wrky dna-binding protein 70 |
| EY664808 | S44288594 | 17 | 56 | 14.54178 | 65.46653 | 2.17055349 | 0 | cs00-c1-101-070-f11- sweet orange infected with xylella fastidiosa (stage 1 of 2) citrus sinensis mrna |
| EY665961 | S44288739 | 1157 | 2193 | 989.6966 | 2563.716 | 1.373178357 | 0 | multidrug resistance |
| EY667216 | S44289098 | 42 | 82 | 35.92675 | 95.86171 | 1.415895991 | 0 | cs00-c1-102-020-f02- sweet orange infected with xylella fastidiosa (stage 2 of 2) citrus sinensis mrna |
| EY667967 | S44289513 | 17 | 3 | 14.54178 | 3.507136 | -2.051838932 | 0 | �ac9 transposase |
| EY668167 | S44289615 | 11 | 2 | 9.409388 | 2.33809 | -2.00877021 | 0 | cs00-c1-102-041-b12- sweet orange infected with xylella fastidiosa (stage 2 of 2) citrus sinensis mrna |
| EY669455 | S44289657 | 1851 | 73 | 1583.343 | 85.3403 | -4.213603212 | 0 | ethylene-responsive transcription factor erf107 |
| EY669560 | S44289762 | 477 | 1128 | 408.0253 | 1318.683 | 1.692367305 | 0 | protein |
| EY672837 | S44291023 | 153 | 1974 | 130.876 | 2307.695 | 4.140179841 | 0 | tyrosine n-monooxygenase ame: full=cytochrome p450tyr ame: full=cytochrome p450 79a1 |
| EY673620 | S44291372 | 36 | 84 | 30.79436 | 98.1998 | 1.67305383 | 0 | zinc finger |
| EY673879 | S44291631 | 261 | 500 | 223.2591 | 584.5226 | 1.388539697 | 0 | protein |
| EY673884 | S44291636 | 41 | 216 | 35.07136 | 252.5138 | 2.847996907 | 0 | primary amine oxidase ame: full=amine oxidase flags: precursor |
| EY674028 | S44291654 | 5664 | 1128 | 4844.979 | 1318.683 | -1.877392789 | 0 | phosphoenolpyruvate carboxykinase� |
| EY674035 | S44291661 | 4 | 69 | 3.421596 | 80.66412 | 4.559185866 | 0 | transcriptional xre family |
| EY674763 | S44291829 | 59 | 0 | 50.46854 | 0 | -Inf | 0 | gdsl esterase lipase at4g28780 ame: full=extracellular lipase at4g28780 flags: precursor |
| EY674818 | S44291870 | 20 | 2 | 17.10798 | 2.33809 | -2.871266686 | 0 | acyl synthetase |
| EY674874 | S44291926 | 75 | 149 | 64.15492 | 174.1877 | 1.441011239 | 0 | cytochrome p450 90d2 ame: full=c6-oxidase |
| EY676418 | S44292028 | 916 | 3452 | 783.5454 | 4035.544 | 2.36467437 | 0 | conserved hypothetical protein [Ricinus communis] |
| EY676803 | S44292077 | 471 | 103 | 402.8929 | 120.4117 | -1.742421313 | 0 | glucan endo- -beta-glucosidase-like protein 3 flags: precursor |
| EY676841 | S44292115 | 31 | 8 | 26.51737 | 9.352362 | -1.503534901 | 0 | protein |
| EY676843 | S44292117 | 903 | 2234 | 772.4252 | 2611.647 | 1.757492702 | 0 | protein |
| EY676846 | S44292120 | 24 | 35 | 20.52957 | 40.91658 | 0.994981925 | 0 | cs00-c1-401-027-g02- sweet orange infected with citrus sinensis mrna |
| EY676956 | S44292230 | 542 | 83 | 463.6262 | 97.03075 | -2.256448201 | 0 | geranylgeranyl pyrophosphate chloroplastic chromoplastic� |
| EY676960 | S44292234 | 260 | 549 | 222.4037 | 641.8058 | 1.528955935 | 0 | probable serine threonine-protein kinase at1g18390 flags: precursor |
| EY677000 | S44292274 | 4885 | 15277 | 4178.624 | 17859.5 | 2.095592206 | 0 | zinc finger ccch domain-containing protein 29� |
| EY677004 | S44292278 | 213 | 2058 | 182.2 | 2405.895 | 3.722979056 | 0 | ethylene-responsive transcription factor 5� |
| EY677013 | S44292287 | 9 | 2 | 7.69859 | 2.33809 | -1.719263592 | 0 | indole-3-glycerol phosphate synthase� |
| EY677554 | S44292394 | 2607 | 281 | 2230.025 | 328.5017 | -2.763087138 | 0 | protein e6 |
| EY678272 | S44292454 | 603 | 22 | 515.8056 | 25.71899 | -4.325921164 | 0 | fasciclin-like arabinogalactan protein 2 flags: precursor |
| EY680588 | S44292866 | 339 | 66 | 289.9802 | 77.15698 | -1.910085935 | 0 | unknown [Glycine max] |
| EY681381 | S44292987 | 12 | 1 | 10.26479 | 1.169045 | -3.134301092 | 0 | receptor protein kinase clavata1 flags: precursor |
| EY681750 | S44293244 | 0 | 10 | 0 | 11.69045 | Inf | 0 | PREDICTED: hypothetical protein [Vitis vinifera] |
| EY681754 | S44293248 | 42 | 1 | 35.92675 | 1.169045 | -4.941656014 | 0 | remorin ame: full=dna-binding protein |
| EY681786 | S44293280 | 14 | 61 | 11.97558 | 71.31176 | 2.574043825 | 0 | PREDICTED: hypothetical protein [Vitis vinifera] |
| EY682244 | S44293416 | 377 | 69 | 322.4854 | 80.66412 | -1.999234847 | 0 | atp binding |
| EY682325 | S44293497 | 554 | 76 | 473.891 | 88.84743 | -2.415153244 | 0 | cs00-c1-650-031-f08- sweet orange young greenhouse plant citrus sinensis mrna |
| EY683932 | S44293998 | 263 | 54 | 224.9699 | 63.12844 | -1.833370078 | 0 | cytochrome p450 86a2 |
| EY684012 | S44294078 | 20 | 115 | 17.10798 | 134.4402 | 2.974223365 | 0 | protein brassinosteroid insensitive 1� |
| EY684017 | S44294083 | 2016 | 274 | 1724.484 | 320.3184 | -2.428586432 | 0 | tsd2 (tumorous shoot development 2) methyltransferase |
| EY684150 | S44294216 | 849 | 77 | 726.2337 | 90.01648 | -3.012172794 | 0 | upf0497 membrane protein 11 |
| EY684198 | S44294264 | 254 | 758 | 217.2713 | 886.1363 | 2.02803076 | 0 | disease resistance protein |
| EY684237 | S44294303 | 1398 | 2898 | 1195.848 | 3387.893 | 1.502354643 | 0 | �transporter |
| EY685678 | S44294372 | 747 | 25 | 638.983 | 29.22613 | -4.450446834 | 0 | mus musculus bac clone rp23-108h19 from complete sequence |
| EY686629 | S44294545 | 17 | 38 | 14.54178 | 44.42372 | 1.611126081 | 0 | cs00-c2-003-055-h07- sweet orange greenhouse plant citrus sinensis mrna |
| EY686851 | S44294767 | 19 | 3 | 16.25258 | 3.507136 | -2.212303604 | 0 | cs00-c2-003-014-h01- sweet orange greenhouse plant citrus sinensis mrna |
| EY686862 | S44294778 | 455 | 1134 | 389.2065 | 1325.697 | 1.768143599 | 0 | cs00-c2-003-030-a03- sweet orange greenhouse plant citrus sinensis mrna |
| EY686876 | S44294792 | 556 | 68 | 475.6018 | 79.49507 | -2.580816822 | 0 | ring finger protein 126 |
| EY686933 | S44294849 | 349 | 238 | 298.5342 | 278.2328 | -0.101604054 | 0 | cs00-c2-003-030-h08- sweet orange greenhouse plant citrus sinensis mrna |
| EY687702 | S44295142 | 4246 | 481 | 3632.024 | 562.3107 | -2.691334163 | 0 | cs00-c2-003-092-e04- sweet orange greenhouse plant citrus sinensis mrna |
| EY687709 | S44295149 | 3187 | 5335 | 2726.156 | 6236.856 | 1.193950661 | 0 | corylus avellana clone kg871 microsatellite sequence |
| EY687966 | S44295182 | 72 | 7 | 61.58872 | 8.183316 | -2.91190867 | 0 | fasciclin-like arabinogalactan protein 10 flags: precursor |
| EY688356 | S44295348 | 201 | 19 | 171.9352 | 22.21186 | -2.952462769 | 0 | 5ng4\_pintaauxin-induced protein 5ng4 |
| EY688459 | S44295451 | 100 | 23 | 85.53989 | 26.88804 | -1.669632825 | 0 | lysine histidine transporter-like 6 |
| EY688477 | S44295469 | 69 | 5 | 59.02253 | 5.845226 | -3.335934953 | 0 | unknown [Glycine max] |
| EY688491 | S44295483 | 17 | 0 | 14.54178 | 0 | -Inf | 0 | patatin-08 ame: full=patatin group d-1 flags: precursor |
| EY690144 | S44295890 | 14 | 1 | 11.97558 | 1.169045 | -3.356693513 | 0 | ubiquitin carboxyl-terminal hydrolase 12 ame: full=ubiquitin thioesterase 12 ame: full=ubiquitin-specific-processing protease 12 ame: full=deubiquitinating enzyme 12� |
| EY690294 | S44295928 | 20 | 2 | 17.10798 | 2.33809 | -2.871266686 | 0 | cs00-c2-003-084-a04- sweet orange greenhouse plant citrus sinensis mrna |
| EY690320 | S44295954 | 16 | 0 | 13.68638 | 0 | -Inf | 0 | cs00-c2-003-084-d03- sweet orange greenhouse plant citrus sinensis mrna |
| EY690363 | S44295997 | 461 | 43 | 394.3389 | 50.26894 | -2.971696777 | 0 | reticuline oxidase-like protein flags: precursor |
| EY691641 | S44296057 | 4640 | 1614 | 3969.051 | 1886.839 | -1.072822818 | 0 | probable non-specific lipid-transfer protein akcs9� |
| EY691688 | S44296104 | 381 | 1282 | 325.907 | 1498.716 | 2.201194768 | 0 | protein |
| EY691718 | S44296134 | 123 | 3 | 105.2141 | 3.507136 | -4.906890596 | 0 | endochitinase 1 flags: precursor |
| EY691729 | S44296145 | 170 | 362 | 145.4178 | 423.1944 | 1.54111636 | 0 | protein kinase g11a |
| EY691800 | S44296216 | 173 | 3 | 147.984 | 3.507136 | -5.399004318 | 0 | dna binding protein |
| EY691847 | S44296263 | 45 | 7 | 38.49295 | 8.183316 | -2.233836765 | 0 | endoglucanase 11 ame: full=endo- -beta glucanase 11 flags: precursor |
| EY691849 | S44296265 | 296 | 55 | 253.1981 | 64.29749 | -1.977432243 | 0 | agglutinin ame: full=cca |
| EY692431 | S44296399 | 13463 | 797 | 11516.24 | 931.729 | -3.627614982 | 0 | cs00-c2-003-071-e03- sweet orange greenhouse plant citrus sinensis mrna |
| EY692924 | S44296668 | 222 | 24 | 189.8986 | 28.05708 | -2.758791957 | 0 | PREDICTED: hypothetical protein [Vitis vinifera] |
| EY693094 | S44296838 | 220 | 589 | 188.1878 | 688.5676 | 1.871425519 | 0 | fructose-6-phosphate 2-kinase fructose- -bisphosphatase |
| EY693117 | S44296861 | 509 | 93 | 435.3981 | 108.7212 | -2.001701626 | 0 | cs00-c3-700-008-a02- sweet orange development stadium (1 of 6) citrus sinensis mrna |
| EY694888 | S44297386 | 172 | 835 | 147.1286 | 976.1527 | 2.730029042 | 0 | 18 kda seed maturation protein |
| EY697211 | S44297931 | 13 | 1 | 11.12019 | 1.169045 | -3.249778309 | 0 | cs00-c3-700-057-e11- sweet orange development stadium (1 of 6) citrus sinensis mrna |
| EY697238 | S44297958 | 72 | 153 | 61.58872 | 178.8639 | 1.53812425 | 0 | 2-hydroxymuconic semialdehyde hydrolase� |
| EY697254 | S44297974 | 101 | 11 | 86.39529 | 12.8595 | -2.748118455 | 0 | gcn5-related n-acetyltransferase brct |
| EY698078 | S44298028 | 139 | 26 | 118.9005 | 30.39518 | -1.967839946 | 0 | tryptophan tyrosine permease |
| EY698173 | S44298123 | 4247 | 1947 | 3632.879 | 2276.131 | -0.674529816 | 0 | ethylene-responsive transcription factor erf003 |
| EY698263 | S44298213 | 12 | 0 | 10.26479 | 0 | -Inf | 0 | myrcene chloroplastic flags: precursor |
| EY699214 | S44298282 | 62 | 29 | 53.03473 | 33.90231 | -0.645553906 | 0 | conserved hypothetical protein [Ricinus communis] |
| EY700386 | S44298460 | 23 | 77 | 19.67418 | 90.01648 | 2.193885994 | 0 | atp synthase subunit chloroplastic ame: full=atpase subunit i ame: full=atp synthase f sector subunit b |
| EY701011 | S44298637 | 453 | 45 | 387.4957 | 52.60703 | -2.880852735 | 0 | subtilisin-like protease ame: full=cucumisin-like serine protease flags: precursor |
| EY702425 | S44298721 | 1273 | 3519 | 1088.923 | 4113.87 | 1.917594504 | 0 | protein |
| EY702934 | S44298894 | 56 | 8 | 47.90234 | 9.352362 | -2.356693513 | 0 | protein |
| EY703299 | S44298923 | 234 | 536 | 200.1633 | 626.6082 | 1.64638588 | 0 | ethylene-responsive transcription factor 3 ame: full=ethylene-responsive element-binding factor 3 homolog ame: full=ethylene-responsive element-binding factor 5� |
| EY703391 | S44299015 | 482 | 100 | 412.3023 | 116.9045 | -1.818371737 | 0 | probable 3-beta-hydroxysteroid-delta -isomerase ame: full=cholestenol delta-isomerase ame: full=delta -delta sterol isomerase� |
| EY703404 | S44299028 | 10 | 70 | 8.553989 | 81.83316 | 3.258016331 | 0 | protein |
| EY705333 | S44299277 | 1212 | 217 | 1036.743 | 253.6828 | -2.030961342 | 0 | transcription factor spatula ame: full=transcription factor en 99 ame: full=bhlh transcription factor bhlh024 ame: full=basic helix-loop-helix protein 24� |
| EY705750 | S44299470 | 480 | 103 | 410.5915 | 120.4117 | -1.769728659 | 0 | cs00-c3-701-047-e04- sweet orange development stadium (2 of 6) citrus sinensis mrna |
| EY707846 | S44299480 | 294 | 553 | 251.4873 | 646.482 | 1.362124734 | 0 | alpha-l-arabinofuranosidase 1� |
| EY707926 | S44299560 | 456 | 1003 | 390.0619 | 1172.552 | 1.587877285 | 0 | probable ubiquitin-conjugating enzyme e2 24 ame: full=ubiquitin-protein ligase 24 ame: full=ubiquitin carrier protein 24 ame: full= 2 |
| EY707941 | S44299575 | 34 | 5 | 29.08356 | 5.845226 | -2.314873337 | 0 | cs00-c3-701-074-c11- sweet orange development stadium (2 of 6) citrus sinensis mrna |
| EY708566 | S44299738 | 1230 | 2947 | 1052.141 | 3445.176 | 1.711250154 | 0 | auxin-induced in root cultures protein 12 flags: precursor |
| EY708591 | S44299763 | 407 | 1875 | 348.1474 | 2191.96 | 2.654451305 | 0 | chaperone protein dnaj chloroplastic� |
| EY708603 | S44299775 | 37 | 4 | 31.64976 | 4.676181 | -2.758791957 | 0 | protein |
| EY708892 | S44299841 | 89 | 7 | 76.1305 | 8.183316 | -3.2177171 | 0 | predicted protein [Populus trichocarpa] |
| EY709546 | S44300061 | 21 | 1 | 17.96338 | 1.169045 | -3.941656014 | 0 | germin-like protein subfamily 1 member 8 flags: precursor |
| EY710217 | S44300172 | 2757 | 445 | 2358.335 | 520.2251 | -2.180560617 | 0 | transcription factor bhlh113 ame: full=transcription factor en 61 ame: full=bhlh transcription factor bhlh113 ame: full=basic helix-loop-helix protein 113� |
| EY710254 | S44300209 | 2025 | 183 | 1732.183 | 213.9353 | -3.017344945 | 0 | cytochrome p450 71d7 |
| EY710274 | S44300229 | 4046 | 51 | 3460.944 | 59.6213 | -5.859193854 | 0 | expansin-a5� |
| EY710287 | S44300242 | 14 | 76 | 11.97558 | 88.84743 | 2.891234 | 0 | 21 kda seed protein flags: precursor |
| EY710311 | S44300266 | 3272 | 460 | 2798.865 | 537.7608 | -2.379805573 | 0 | protein |
| EY710329 | S44300284 | 431 | 1902 | 368.6769 | 2223.524 | 2.592418881 | 0 | wrky transcription factor 6 ame: full=wrky dna-binding protein 6� |
| EY710358 | S44300313 | 3209 | 23011 | 2744.975 | 26900.9 | 3.292789395 | 0 | squidulin ame: full=optic lobe calcium-binding protein ame: full=scabp |
| EY710369 | S44300324 | 1184 | 2309 | 1012.792 | 2699.325 | 1.414260501 | 0 | cs00-c3-701-107-b01- sweet orange development stadium (2 of 6) citrus sinensis mrna |
| EY710382 | S44300337 | 777 | 48 | 664.645 | 56.11417 | -3.566146879 | 0 | miraculin-like protein 2 |
| EY710727 | S44300346 | 4718 | 883 | 4035.772 | 1032.267 | -1.967028667 | 0 | 14 kda proline-rich protein flags: precursor |
| EY710758 | S44300377 | 418 | 60 | 357.5567 | 70.14271 | -2.349807127 | 0 | 3-ketoacyl- synthase 21� |
| EY710797 | S44300416 | 2637 | 583 | 2255.687 | 681.5533 | -1.726668374 | 0 | momilactone a synthase� |
| EY711363 | S44300548 | 4 | 13 | 3.421596 | 15.19759 | 2.151101127 | 0 | cs00-c3-702-006-h03- sweet orange development stadium (3 of 6) citrus sinensis mrna |
| EY711719 | S44300666 | 112 | 245 | 95.80468 | 286.4161 | 1.579944426 | 0 | amino acid binding |
| EY711829 | S44300776 | 3514 | 720 | 3005.872 | 841.7125 | -1.836383971 | 0 | atp-citrate synthase ame: full=atp-citrate (pro-s-)-lyase ame: full=citrate cleavage enzyme |
| EY711870 | S44300817 | 33 | 2 | 28.22816 | 2.33809 | -3.59373271 | 0 | btb poz domain-containing protein at1g30440 |
| EY711896 | S44300843 | 60 | 6 | 51.32394 | 7.014271 | -2.871266686 | 0 | predicted protein [Populus trichocarpa] |
| EY712586 | S44300973 | 27 | 48 | 23.09577 | 56.11417 | 1.280736408 | 0 | 70 kda peptidyl-prolyl isomerase ame: full=peptidyl-prolyl cis-trans isomerase� |
| EY712705 | S44301092 | 151 | 13 | 129.1652 | 15.19759 | -3.087303612 | 0 | PREDICTED: hypothetical protein [Vitis vinifera] |
| EY713160 | S44301211 | 17 | 1 | 14.54178 | 1.169045 | -3.636801432 | 0 | btb poz domain-containing protein at5g48800 |
| EY713356 | S44301407 | 123 | 12 | 105.2141 | 14.02854 | -2.906890596 | 0 | protein |
| EY713677 | S44301504 | 0 | 10 | 0 | 11.69045 | Inf | 0 | cs00-c3-702-033-f07- sweet orange development stadium (3 of 6) citrus sinensis mrna |
| EY713683 | S44301510 | 285 | 35 | 243.7887 | 40.91658 | -2.574873683 | 0 | vinorine synthase |
| EY713717 | S44301544 | 7689 | 1460 | 6577.162 | 1706.806 | -1.946166201 | 0 | (+)-delta-cadinene synthase� |
| EY714117 | S44301608 | 41 | 98 | 35.07136 | 114.5664 | 1.707819249 | 0 | unnamed protein product [Vitis vinifera] |
| EY714913 | S44302068 | 43 | 3 | 36.78215 | 3.507136 | -3.390640845 | 0 | PREDICTED: hypothetical protein [Vitis vinifera] |
| EY714918 | S44302073 | 133 | 9 | 113.7681 | 10.52141 | -3.434696025 | 0 | beta-galactosidase 10� |
| EY714927 | S44302082 | 156 | 309 | 133.4422 | 361.235 | 1.436722218 | 0 | cs00-c3-702-048-d06- sweet orange development stadium (3 of 6) citrus sinensis mrna |
| EY716273 | S44302644 | 483 | 1100 | 413.1577 | 1285.95 | 1.638069839 | 0 | nucleic acid binding |
| EY716408 | S44302779 | 94 | 17 | 80.4075 | 19.87377 | -2.016464601 | 0 | cs00-c3-702-067-b04- sweet orange development stadium (3 of 6) citrus sinensis mrna |
| EY716813 | S44302848 | 451 | 317 | 385.7849 | 370.5873 | -0.057983184 | 0 | cytochrome p450 78a3 |
| EY716845 | S44302880 | 254 | 49 | 217.2713 | 57.28321 | -1.923313434 | 0 | chalcone isomerase-like protein |
| EY717550 | S44303165 | 301 | 750 | 257.4751 | 876.7839 | 1.767788518 | 0 | cs00-c3-702-080-f08- sweet orange development stadium (3 of 6) citrus sinensis mrna |
| EY718133 | S44303510 | 1218 | 222 | 1041.876 | 259.528 | -2.005221143 | 0 | beta-amylase ame: full= -alpha-d-glucan maltohydrolase |
| EY718470 | S44303609 | 879 | 133 | 751.8957 | 155.483 | -2.273775511 | 0 | ent-kaurenoic acid oxidase 2� |
| EY721378 | S44304065 | 862 | 121 | 737.3539 | 141.4545 | -2.382019413 | 0 | sigma factor sigb regulation protein rsbq |
| EY722419 | S44304322 | 23 | 2 | 19.67418 | 2.33809 | -3.072900547 | 0 | f-box protein at3g07870 |
| EY722494 | S44304397 | 548 | 99 | 468.7586 | 115.7355 | -2.018014054 | 0 | (-)-germacrene d synthase |
| EY722541 | S44304444 | 29 | 65 | 24.80657 | 75.98794 | 1.615048227 | 0 | PREDICTED: hypothetical protein [Vitis vinifera] |
| EY724949 | S44305074 | 124 | 358 | 106.0695 | 418.5182 | 1.980280876 | 0 | sugar transport protein 14 ame: full=hexose transporter 14 |
| EY724953 | S44305078 | 189 | 34 | 161.6704 | 39.74754 | -2.024118174 | 0 | �kda class iii heat shock protein ame: full= kda heat shock protein 2� |
| EY725652 | S44305223 | 32 | 86 | 27.37277 | 100.5379 | 1.876926164 | 0 | cs00-c3-703-037-c08- sweet orange development stadium (4 of 6) citrus sinensis mrna |
| EY726529 | S44305445 | 103 | 22 | 88.10609 | 25.71899 | -1.7764075 | 0 | atp binding |
| EY726580 | S44305496 | 869 | 2836 | 743.3417 | 3315.412 | 2.157090859 | 0 | ethylene-responsive transcription factor rap2-3 ame: full=protein related to apetala2 3� |
| EY727621 | S44305879 | 125150 | 24403 | 107053.2 | 28528.21 | -1.907866459 | 0 | cs00-c3-703-103-e10- sweet orange development stadium (4 of 6) citrus sinensis mrna |
| EY727625 | S44305883 | 322 | 10 | 275.4385 | 11.69045 | -4.558327374 | 0 | pollen-specific protein sf3 |
| EY727745 | S44306003 | 93 | 23 | 79.5521 | 26.88804 | -1.564935446 | 0 | zinc finger |
| EY727789 | S44306047 | 473 | 50 | 404.6037 | 58.45226 | -2.791178775 | 0 | cytochrome p450 93a1 |
| EY727793 | S44306051 | 6336 | 1401 | 5419.808 | 1637.832 | -1.726453971 | 0 | �kda class i heat shock protein ame: full=hsp |
| EY727849 | S44306107 | 132 | 245 | 112.9127 | 286.4161 | 1.342905229 | 0 | cs00-c3-703-106-d10- sweet orange development stadium (4 of 6) citrus sinensis mrna |
| EY727895 | S44306153 | 79 | 324 | 67.57651 | 378.7706 | 2.486730664 | 0 | plastidic atp adp-transporter |
| EY728175 | S44306209 | 11651 | 2747 | 9966.253 | 3211.367 | -1.633863562 | 0 | 21 kda protein ame: full= protein flags: precursor |
| EY728204 | S44306238 | 1906 | 323 | 1630.39 | 377.6016 | -2.11028064 | 0 | cellulose synthase a catalytic subunit 2� |
| EY728730 | S44306330 | 707 | 482 | 604.767 | 563.4798 | -0.10201566 | 0 | cs00-c3-704-007-g11- sweet orange development stadium (5 of 6) citrus sinensis mrna |
| EY729980 | S44306572 | 18 | 57 | 15.39718 | 66.63558 | 2.113626422 | 0 | protein |
| EY730799 | S44306845 | 21 | 49 | 17.96338 | 57.28321 | 1.67305383 | 0 | protein binding |
| EY730905 | S44306951 | 61 | 9 | 52.17933 | 10.52141 | -2.310150927 | 0 | tetratricopeptide repeat protein 7b� |
| EY731155 | S44307089 | 38 | 169 | 32.50516 | 197.5686 | 2.603613332 | 0 | u-box domain-containing protein 32 ame: full=plant u-box protein 32 |
| EY734414 | S44307428 | 6887 | 1408 | 5891.132 | 1646.016 | -1.839566934 | 0 | cytokinin-o-glucosyltransferase 2 ame: full=zeatin o-glucosyltransferase 2� |
| EY734418 | S44307432 | 544 | 1330 | 465.337 | 1554.83 | 1.740409098 | 0 | probable wrky transcription factor 65 ame: full=wrky dna-binding protein 65 |
| EY735029 | S44307707 | 21 | 77 | 17.96338 | 90.01648 | 2.325130527 | 0 | cs00-c3-704-075-e05- sweet orange development stadium (5 of 6) citrus sinensis mrna |
| EY735307 | S44307873 | 87 | 1 | 74.41971 | 1.169045 | -5.992282087 | 0 | cs00-c3-704-093-f03- sweet orange development stadium (5 of 6) citrus sinensis mrna |
| EY735395 | S44307961 | 2 | 14 | 1.710798 | 16.36663 | 3.258016331 | 0 | bordetella bronchiseptica strain complete genome segment 11 16 |
| EY736894 | S44308242 | 424 | 826 | 362.6891 | 965.6313 | 1.412738926 | 0 | protein |
| EY740026 | S44308958 | 536 | 65 | 458.4938 | 75.98794 | -2.593059968 | 0 | �homolog subfamily b member 6 ame: full=mrj |
| EY741616 | S44309316 | 65 | 14 | 55.60093 | 16.36663 | -1.764351482 | 0 | acyltransferase-like protein chloroplastic flags: precursor |
| EY742192 | S44309668 | 26 | 5 | 22.24037 | 5.845226 | -1.927850214 | 0 | cs00-c3-705-024-b03- sweet orange development stadium (6 of 6) citrus sinensis mrna |
| EY743321 | S44310013 | 157 | 31 | 134.2976 | 36.2404 | -1.889763029 | 0 | cs00-c3-705-093-a11- sweet orange development stadium (6 of 6) citrus sinensis mrna |
| EY743349 | S44310041 | 896 | 140 | 766.4374 | 163.6663 | -2.227410496 | 0 | ferric reductase-like transmembrane component |
| EY743555 | S44310247 | 1194 | 3011 | 1021.346 | 3519.995 | 1.78510128 | 0 | probable inactive receptor kinase at1g27190 flags: precursor |
| EY743725 | S44310417 | 290 | 46 | 248.0657 | 53.77608 | -2.205685725 | 0 | chavicol o-methyltransferase ame: full= eugenol o-methyltransferase cvomt1 ame: full=s-adenosysl-l-methionine: eugenol o-methyltransferase cvomt1 |
| EY743805 | S44310497 | 6392 | 303 | 5467.71 | 354.2207 | -3.9482163 | 0 | expansin-a10� |
| EY744761 | S44310557 | 1420 | 3042 | 1214.666 | 3556.235 | 1.549790632 | 0 | protein |
| EY744797 | S44310593 | 153 | 28 | 130.876 | 32.73327 | -1.999371512 | 0 | thaumatin-like protein flags: precursor |
| EY744809 | S44310605 | 773 | 2358 | 661.2234 | 2756.609 | 2.059684808 | 0 | floral homeotic protein apetala 2 |
| EY744814 | S44310610 | 130 | 427 | 111.2019 | 499.1823 | 2.166385856 | 0 | phosphatidylserine synthase 2� |
| EY745561 | S44310685 | 82 | 424 | 70.14271 | 495.6752 | 2.821029859 | 0 | heat stress transcription factor c-1� |
| EY745993 | S44310786 | 4426 | 8708 | 3785.996 | 10180.05 | 1.426999365 | 0 | mitogen-activated protein kinase 3� |
| EY746025 | S44310818 | 215 | 32 | 183.9108 | 37.40945 | -2.297531441 | 0 | 4-coumarate-- ligase 1� |
| EY746274 | S44310857 | 9 | 0 | 7.69859 | 0 | -Inf | 0 | cs00-c5-003-030-a04- sweet orange greenhouse plant citrus sinensis mrna |
| EY746283 | S44310866 | 1950 | 3901 | 1668.028 | 4560.445 | 1.451031283 | 0 | �-related lipid transfer protein mitochondrial ame: full=start domain-containing protein 7� |
| EY746318 | S44310901 | 1222 | 64 | 1045.297 | 74.81889 | -3.804367161 | 0 | protein |
| EY746373 | S44310956 | 0 | 26 | 0 | 30.39518 | Inf | 0 | protein |
| EY746433 | S44311016 | 99 | 375 | 84.68449 | 438.3919 | 2.372051574 | 0 | protein tify 5a ame: full=jasmonate zim domain-containing protein 8 |
| EY746569 | S44311152 | 6242 | 973 | 5339.4 | 1137.481 | -2.230835238 | 0 | dehydration-responsive protein rd22 flags: precursor |
| EY746603 | S44311186 | 159 | 307 | 136.0084 | 358.8969 | 1.399873299 | 0 | acyl- chloroplastic ame: full=stearoyl-acp desaturase flags: precursor |
| EY746650 | S44311233 | 1109 | 47 | 948.6374 | 54.94512 | -4.109793389 | 0 | pectinesterase pectinesterase inhibitor ppe8b includes: ame: full=pectinesterase inhibitor ppe8b ame: full=pectin methylesterase inhibitor ppe8b includes: ame: full=pectinesterase ppe8b� |
| EY746671 | S44311254 | 62 | 3 | 53.03473 | 3.507136 | -3.918572401 | 0 | beta-galactosidase 5� |
| EY747177 | S44311424 | 109 | 8 | 93.23848 | 9.352362 | -3.317522916 | 0 | vacuolar protein sorting-associated protein 2 homolog 2� |
| EY747215 | S44311462 | 106 | 23 | 90.67229 | 26.88804 | -1.753697089 | 0 | transcription factor bhlh78 ame: full=transcription factor en 86 ame: full=bhlh transcription factor bhlh078 ame: full=basic helix-loop-helix protein 78� |
| EY747226 | S44311473 | 1467 | 2792 | 1254.87 | 3263.974 | 1.37909148 | 0 | at5g18670 t1a4\_50 |
| EY747267 | S44311514 | 333 | 28 | 284.8478 | 32.73327 | -3.121362036 | 0 | snakin-2 flags: precursor |
| EY747781 | S44311594 | 70 | 14 | 59.87792 | 16.36663 | -1.871266686 | 0 | cs00-c5-003-048-h03- sweet orange greenhouse plant citrus sinensis mrna |
| EY747810 | S44311623 | 6216 | 132 | 5317.16 | 154.314 | -5.10671526 | 0 | acidic endochitinase win6 flags: precursor |
| EY747838 | S44311651 | 136 | 404 | 116.3343 | 472.2943 | 2.021410051 | 0 | at4g37300 c7a10\_60 |
| EY747865 | S44311678 | 147 | 443 | 125.7436 | 517.887 | 2.042151953 | 0 | at1g70780 f5a18\_4 |
| EY748285 | S44311762 | 50 | 11 | 42.76995 | 12.8595 | -1.733763162 | 0 | gdsl esterase lipase exl3 ame: full=family ii extracellular lipase 3� |
| EY748394 | S44311871 | 1 | 11 | 0.855399 | 12.8595 | 3.910093028 | 0 | auxin-induced protein 10a5 |
| EY748412 | S44311889 | 29 | 60 | 24.80657 | 70.14271 | 1.499571009 | 0 | cyclic nucleotide-gated ion channel 1� |
| EY748505 | S44311982 | 210 | 194 | 179.6338 | 226.7948 | 0.336328734 | 0 | superoxide dismutase 4ap |
| EY748586 | S44312063 | 164 | 44 | 140.2854 | 51.43799 | -1.447458977 | 0 | abc transporter c family member 9� |
| EY749118 | S44312245 | 27 | 1 | 23.09577 | 1.169045 | -4.304226093 | 0 | probable xyloglucan endotransglucosylase hydrolase protein b ame: full= 2 flags: precursor |
| EY749642 | S44312433 | 15 | 0 | 12.83098 | 0 | -Inf | 0 | probable lrr receptor-like serine threonine-protein kinase at2g23950 flags: precursor |
| EY749788 | S44312579 | 91 | 16 | 77.8413 | 18.70472 | -2.057133231 | 0 | unknown [Glycine max] |
| EY749818 | S44312609 | 187 | 33 | 159.9596 | 38.57849 | -2.051838932 | 0 | vitis vinifera beta-galactosidase mrna |
| EY749825 | S44312616 | 37 | 75 | 31.64976 | 87.67839 | 1.470026734 | 0 | apocytochrome f flags: precursor |
| EY750341 | S44312684 | 98 | 21 | 83.82909 | 24.54995 | -1.771731012 | 0 | squamosa promoter-binding-like protein 17 |
| EY750381 | S44312724 | 1507 | 240 | 1289.086 | 280.5708 | -2.199911697 | 0 | myb-like protein j |
| EY750476 | S44312819 | 19 | 114 | 16.25258 | 133.2712 | 3.03562391 | 0 | indole-3-acetic acid-amido synthetase ame: full=auxin-responsive gh3-like protein 3� |
| EY750528 | S44312871 | 2 | 14 | 1.710798 | 16.36663 | 3.258016331 | 0 | cs00-c5-003-096-c02- sweet orange greenhouse plant citrus sinensis mrna |
| EY750585 | S44312928 | 60 | 15 | 51.32394 | 17.53568 | -1.549338591 | 0 | phenylalanine ammonia-lyase |
| EY751682 | S44313037 | 20 | 2 | 17.10798 | 2.33809 | -2.871266686 | 0 | gata transcription factor 10� |
| EY752069 | S44313102 | 48 | 620 | 41.05915 | 724.808 | 4.141823314 | 0 | cs00-c5-003-092-e05- sweet orange greenhouse plant citrus sinensis mrna |
| EY752072 | S44313105 | 118 | 11 | 100.9371 | 12.8595 | -2.972550022 | 0 | glycerol-3-phosphate acyltransferase 6� |
| EY752079 | S44313112 | 243 | 1497 | 207.8619 | 1750.061 | 3.073707411 | 0 | transcription factor bhlh148 ame: full=transcription factor en 143 ame: full=bhlh transcription factor bhlh148 ame: full=basic helix-loop-helix protein 148� |
| EY752668 | S44313245 | 303 | 744 | 259.1859 | 869.7696 | 1.746646237 | 0 | iaa-amino acid hydrolase ilr1-like 6 ame: full=protein gr1 flags: precursor |
| EY752669 | S44313246 | 15 | 1 | 12.83098 | 1.169045 | -3.456229187 | 0 | squamosa promoter-binding-like protein 13 |
| EY754004 | S44313587 | 52 | 107 | 44.48074 | 125.0878 | 1.491688677 | 0 | cs12-c1-001-009-d12- sweet orange field plant a citrus sinensis mrna |
| EY754023 | S44313606 | 1508 | 291 | 1289.942 | 340.1922 | -1.922883961 | 0 | conserved hypothetical protein [Ricinus communis] |
| EY754662 | S44313783 | 228 | 395 | 195.031 | 461.7729 | 1.243480238 | 0 | protein twin lov 1 |
| EY754664 | S44313785 | 601 | 870 | 514.0948 | 1017.069 | 0.984311819 | 0 | indole-3-acetate beta-glucosyltransferase ame: full=iaa-glu synthetase ame: full=(uridine 5 -diphosphate-glucose:indol-3-ylacetyl)-beta-d-glucosyl transferase |
| EY754720 | S44313841 | 20 | 75 | 17.10798 | 87.67839 | 2.357552005 | 0 | cs12-c1-001-019-c10- sweet orange field plant a citrus sinensis mrna |
| EY755171 | S44314068 | 16 | 34 | 13.68638 | 39.74754 | 1.53812425 | 0 | probable phosphatase phospho2 |
| EY755611 | S44314396 | 686 | 150 | 586.8037 | 175.3568 | -1.742584667 | 0 | agglutinin ame: full=cca |
| EY756075 | S44314636 | 4 | 9 | 3.421596 | 10.52141 | 1.62058641 | 0 | populus trichocarpa mrna |
| EY756179 | S44314740 | 66 | 7 | 56.45633 | 8.183316 | -2.786377788 | 0 | cs13-c1-001-005-e10- sweet orange field plant b citrus sinensis mrna |
| EY756637 | S44314862 | 13 | 0 | 11.12019 | 0 | -Inf | 0 | phytochromobilin:ferredoxin chloroplastic ame: full=phytochromobilin synthase ame: full=pfb synthase ame: full=p synthase flags: precursor |
| EY756705 | S44314930 | 247 | 1593 | 211.2835 | 1862.289 | 3.139824729 | 0 | uncharacterized n-acetyltransferase p20 |
| EY757160 | S44315049 | 24 | 85 | 20.52957 | 99.36884 | 2.275089844 | 0 | cs13-c1-001-018-b05- sweet orange field plant b citrus sinensis mrna |
| EY757272 | S44315161 | 999 | 2146 | 854.5435 | 2508.771 | 1.553754902 | 0 | pre-mrna-splicing factor sf2 ame: full=sr1 protein |
| EY757666 | S44315233 | 151 | 22 | 129.1652 | 25.71899 | -2.328311712 | 0 | nucleobase-ascorbate transporter 6� |
| EY757863 | S44315318 | 14 | 52 | 11.97558 | 60.79035 | 2.343746205 | 0 | serine carboxypeptidase-like 42 flags: precursor |
| EY757917 | S44315372 | 51 | 4 | 43.62535 | 4.676181 | -3.221763933 | 0 | glucan endo- -beta-glucosidase 1 ame: full=(1- |
| FE659262 | S46102851 | 8 | 19 | 6.843191 | 22.21186 | 1.698588922 | 0 | 347 hong anliu sweet orange ssh library citrus sinensis cdna 5 mrna |
| FE659277 | S46102866 | 4 | 4 | 3.421596 | 4.676181 | 0.450661409 | 0 | probable 1-acyl-sn-glycerol-3-phosphate acyltransferase 5 ame: full=lysophosphatidyl acyltransferase 5 |
| EU861194 | S46915372 | 2819 | 276 | 2411.37 | 322.6565 | -2.901781897 | 0 | glucose-1-phosphate adenylyltransferase large subunit 1 ame: full=alpha-d-glucose-1-phosphate adenyl transferase ame: full=adp-glucose pyrophosphorylase ame: full=agpase s ame: full=adp-glucose synthase |
| DC887765 | S47736242 | 1 | 2 | 0.855399 | 2.33809 | 1.450661409 | 0 | dc887765 eic citrus sinensis cdna clone eic0845 5 mrna |
| DC900219 | S47736517 | 57256 | 2522 | 48976.72 | 2948.332 | -4.054125295 | 0 | peroxidase 42� |
| DC900223 | S47736521 | 189 | 34 | 161.6704 | 39.74754 | -2.024118174 | 0 | xyloglucan endotransglucosylase hydrolase protein 9� |
| FC871130 | S49954784 | 24 | 1 | 20.52957 | 1.169045 | -4.134301092 | 0 | conserved hypothetical protein [Ricinus communis] |
| FC871426 | S49955080 | 459 | 4095 | 392.6281 | 4787.24 | 3.607958802 | 0 | dicyanin blue copper protein precursor |
| FC871575 | S49955229 | 143 | 336 | 122.322 | 392.7992 | 1.683107495 | 0 | conserved hypothetical protein [Ricinus communis] |
| FC921856 | S49955684 | 140 | 538 | 119.7558 | 628.9463 | 2.392840755 | 0 | 6-phosphofructokinase 3 ame: full=phosphofructokinase 3 ame: full=phosphohexokinase 3 |
| FC921914 | S49955742 | 16 | 212 | 13.68638 | 247.8376 | 4.178581864 | 0 | bahd acyltransferase at5g47980 |
| FC922200 | S49956028 | 1778 | 310 | 1520.899 | 362.404 | -2.069253795 | 0 | sjchgc09076 protein |
| FC922215 | S49956043 | 1749 | 7309 | 1496.093 | 8544.551 | 2.513805153 | 0 | af211539\_1avr9 cf-9 rapidly elicited protein 65 |
| FC922338 | S49956166 | 112 | 896 | 95.80468 | 1047.464 | 3.450661409 | 0 | disease resistance response protein 206 |
| FC922537 | S49956365 | 0 | 47 | 0 | 54.94512 | Inf | 0 | lotus japonicus genomic chromosome clone: complete sequence |
| FC922608 | S49956436 | 28 | 606 | 23.95117 | 708.4414 | 4.88648047 | 0 | bap2 (bon association protein 2) |
| FC922707 | S49956535 | 19 | 39 | 16.25258 | 45.59276 | 1.488136114 | 0 | protein |
| FC922972 | S49956800 | 269 | 766 | 230.1023 | 895.4886 | 1.960399628 | 0 | nacl-inducible calcium |
|  |  |  |  |  |  |  |  |  |
