# Supplementary material for: Transcriptome Profiling of Citrus Fruit Response to Huanglongbing Disease
Source: PLoS One. 2012 May 31;7(5):e38039. doi: 10.1371/journal.pone.0038039 (PMC3364978; doi:10.1371/journal.pone.0038039)
Supplement: Table S2 — Differentially expressed genes in asymptomatic fruit in comparison to control (healthy in disease-free location), annotations and number of protein-protein interactions deduced from Arabidopsis knowledgebase. (HTM) [file pone.0038039.s002.htm]

Table�2


# Table�S2

| Table S2. Differentially expressed genes in asymptomatic fruit in comparison to control (healthy in disease-free location), annotations and number of protein-protein interactions deduced from Arabidopsis knowledgebase. Those that also appear in Table S7 are hightlighted in color. | | | | | | | | | | | |
|  |  |  |  |  |  |  |  |  |  |  |  |
| GB id | id2 | count CO | count AS | norm CO | norm AS | log2foldchange | PPI | annotation |  |  |  |
| EY693454 | S44237646 | 4826 | 379 | 4407.676 | 414.9702 | -3.408938678 | 444 | heat shock protein 82 |  |  |  |
| EY696492 | S44297884 | 42 | 6 | 38.35938 | 6.569448 | -2.545735438 | 69 | microtubule-associated protein rp eb family member 3 ame: full=end-binding protein 3� |  |  |  |
| EY696074 | S44239496 | 108 | 233 | 98.63842 | 255.1136 | 1.370918127 | 64 | asc1-like protein 1 ame: full=alternaria stem canker resistance-like protein 1 |  |  |  |
| CN191703 | S22550924 | 23 | 80 | 21.00633 | 87.59265 | 2.059985623 | 57 | branched-chain-amino-acid aminotransferase chloroplastic� |  |  |  |
| CX302207 | S24635232 | 18 | 2 | 16.43974 | 2.189816 | -2.908305517 | 56 | tubulin alpha chain |  |  |  |
| DN620775 | S24240822 | 3 | 19 | 2.739956 | 20.80325 | 2.924584497 | 48 | ucrcs11\_05m20\_f parent washington navel orange scale-infested rind cdna library ucrcs11 citrus sinensis cdna clone mrna |  |  |  |
| EY727672 | S44305930 | 62 | 14 | 56.62576 | 15.32871 | -1.885221904 | 41 | chromatin modification-related protein yng2 ame: full=ing1 homolog 2 |  |  |  |
| EY656304 | S44211814 | 101 | 253 | 92.24519 | 277.0117 | 1.586401576 | 34 | hevamine-a includes: ame: full=chitinase includes: ame: full=lysozyme flags: precursor |  |  |  |
| EY662694 | S44287810 | 12942 | 1183 | 11820.17 | 1295.276 | -3.189919119 | 31 | �kda proline-rich protein |  |  |  |
| EY670319 | S44290185 | 56 | 15 | 51.14585 | 16.42362 | -1.638844842 | 31 | glucan endo- -beta-glucosidase 4 ame: full=(1- |  |  |  |
| EY700239 | S44243101 | 48 | 8 | 43.8393 | 8.759265 | -2.323343017 | 29 | rac-like gtp-binding protein arac7 ame: full=gtpase protein rop9 |  |  |  |
| EY741416 | S44273324 | 663 | 1435 | 605.5303 | 1571.193 | 1.375589446 | 29 | �h aca ribonucleoprotein complex subunit 1-like protein 1 |  |  |  |
| EY754184 | S44281777 | 4 | 15 | 3.653275 | 16.42362 | 2.16851008 | 29 | enhancer of polycomb-like protein |  |  |  |
| EY679658 | S44228252 | 12 | 3 | 10.95982 | 3.284724 | -1.738380516 | 27 | isochorismate chloroplastic flags: precursor |  |  |  |
| EY707908 | S44299542 | 38 | 160 | 34.70611 | 175.1853 | 2.335620066 | 26 | thioredoxin-like 4 |  |  |  |
| CF653559 | S22533293 | 320 | 36 | 292.262 | 39.41669 | -2.890383609 | 24 | pathogenesis-related protein 1� |  |  |  |
| EY701935 | S44244461 | 9 | 0 | 8.219868 | 0 | -Inf | 23 | probable histone |  |  |  |
| EY757748 | S44283689 | 254 | 760 | 231.9829 | 832.1301 | 1.842790406 | 23 | ctp synthase 2 ame: full=utp--ammonia ligase 2 ame: full=ctp synthetase 2 |  |  |  |
| EY658733 | S44213529 | 29158 | 7535 | 26630.55 | 8250.132 | -1.690592865 | 22 | s-adenosylmethionine synthetase 3� |  |  |  |
| EY692586 | S44237338 | 12 | 1 | 10.95982 | 1.094908 | -3.323343017 | 22 | dna replication licensing factor mcm5 ame: full=cdc46 homolog ame: full=p1-cdc46 |  |  |  |
| EY664033 | S44216939 | 20 | 69 | 18.26637 | 75.54866 | 2.048215846 | 21 | protein |  |  |  |
| DC900352 | S47736649 | 1021 | 204 | 932.4984 | 223.3612 | -2.061722325 | 20 | chloroplast-targeted copper |  |  |  |
| EY718305 | S44255972 | 9130 | 1973 | 8338.6 | 2160.254 | -1.94860442 | 19 | heat shock protein 101 |  |  |  |
| EY750410 | S44312753 | 119 | 24 | 108.6849 | 26.27779 | -2.048235779 | 18 | actin-related protein 2 3 complex subunit 2 ame: full=arp2 3 complex 34 kda subunit� |  |  |  |
| EY657469 | S44212615 | 318 | 827 | 290.4353 | 905.489 | 1.640480048 | 17 | probable inositol transporter 2 |  |  |  |
| EY720565 | S44257806 | 847 | 207 | 773.5809 | 226.646 | -1.771111718 | 15 | auxin efflux carrier family protein |  |  |  |
| EY750416 | S44312759 | 652 | 164 | 595.4838 | 179.5649 | -1.729556666 | 15 | 3-ketoacyl- synthase 6� |  |  |  |
| DN619030 | S24242071 | 29 | 130 | 26.48624 | 142.338 | 2.426006302 | 14 | protein |  |  |  |
| CN190487 | S22548612 | 750 | 1952 | 684.989 | 2137.261 | 1.641610036 | 13 | �nuclease harbi1 ame: full=harbinger transposase-derived nuclease |  |  |  |
| EY745239 | S44275817 | 404 | 1791 | 368.9807 | 1960.98 | 2.409957623 | 13 | cbl-interacting serine threonine-protein kinase 14 ame: full=sos2-like protein kinase pks24 ame: full=snf1-related kinase ame: full=serine threonine-protein kinase sr1� |  |  |  |
| EY666662 | S44288880 | 326 | 816 | 297.7419 | 893.445 | 1.585316672 | 13 | two-component response regulator-like aprr1 ame: full=pseudo-response regulator 1 ame: full=timing of cab expression 1 ame: full=abi3-interacting protein 1 |  |  |  |
| EY654993 | S44210909 | 131 | 328 | 119.6447 | 359.1298 | 1.585748487 | 12 | probable pre-mrna-splicing factor atp-dependent rna helicase |  |  |  |
| EY728087 | S44263101 | 352 | 35 | 321.4882 | 38.32178 | -3.068529118 | 12 | peptide transporter ptr2 ame: full=histidine-transporting protein |  |  |  |
| DC900116 | S47736414 | 26566 | 2861 | 24263.22 | 3132.532 | -2.953370133 | 11 | phosphoenolpyruvate carboxykinase� |  |  |  |
| CB610757 | S22560156 | 355 | 1376 | 324.2281 | 1506.593 | 2.216209024 | 10 | homeobox-leucine zipper protein athb-12 ame: full=homeodomain transcription factor athb-12 ame: full=hd-zip protein athb-12 |  |  |  |
| CX074546 | S22599307 | 32 | 2 | 29.2262 | 2.189816 | -3.738380516 | 10 | interactor of constitutive active rops 4 |  |  |  |
| DY257364 | S34124642 | 75 | 212 | 68.4989 | 232.1205 | 1.760721248 | 10 | ammonium transporter 2� |  |  |  |
| CV719482 | S22579072 | 31 | 4 | 28.31288 | 4.379632 | -2.692576826 | 9 | syntaxin-related protein knolle ame: full=syntaxin-111� |  |  |  |
| EY748702 | S44277833 | 20 | 60 | 18.26637 | 65.69448 | 1.846581985 | 9 | 30s ribosomal protein chloroplastic |  |  |  |
| CX050627 | S22591393 | 25 | 72 | 22.83297 | 78.83338 | 1.787688296 | 8 | histidine-containing phosphotransfer protein 5 |  |  |  |
| DY305483 | S34125045 | 2674 | 9228 | 2442.214 | 10103.81 | 2.048638023 | 8 | ferritin- chloroplastic ame: full=s -3 flags: precursor |  |  |  |
| CV712803 | S22579748 | 129 | 351 | 117.8181 | 384.3127 | 1.705719449 | 7 | probable cytochrome b5 isoform 2 |  |  |  |
| EY688187 | S44234143 | 57 | 6 | 52.05917 | 6.569448 | -2.986308029 | 7 | hva22-like protein c� |  |  |  |
| EY724518 | S44260871 | 2327 | 576 | 2125.293 | 630.667 | -1.75271101 | 7 | oligopeptide transporter 3� |  |  |  |
| CV719657 | S22579175 | 5 | 0 | 4.566593 | 0 | -Inf | 6 | general transcription factor iif subunit 2 ame: full=transcription initiation factor iif subunit beta� |  |  |  |
| CX044490 | S22588416 | 107 | 450 | 97.7251 | 492.7086 | 2.333933689 | 6 | sulfate transporter ame: full=ast12 ame: full= 1 |  |  |  |
| DY305635 | S34125197 | 161 | 394 | 147.0443 | 431.3938 | 1.552754425 | 6 | squalene monooxygenase ame: full=squalene epoxidase� |  |  |  |
| EY690487 | S44235673 | 12 | 3 | 10.95982 | 3.284724 | -1.738380516 | 6 | nitrate transporter, putative [Ricinus communis] |  |  |  |
| EY675704 | S44224970 | 389 | 81 | 355.281 | 88.68755 | -2.002156858 | 5 | ring finger and chy zinc finger domain-containing protein 1 ame: full=zinc finger protein 363 ame: full=ch-rich-interacting match with plag1 ame: full=androgen receptor n-terminal-interacting protein ame: full=p53-induced ring-h2 protein� |  |  |  |
| EY746955 | S44276870 | 243 | 7 | 221.9364 | 7.664356 | -4.855838097 | 5 | aquaporin |  |  |  |
| CV712980 | S22579821 | 6 | 13 | 5.479912 | 14.2338 | 1.377096701 | 4 | origin recognition complex subunit 6 |  |  |  |
| CX044129 | S22586518 | 494 | 2771 | 451.1794 | 3033.99 | 2.749443248 | 4 | probable wrky transcription factor 53 ame: full=wrky dna-binding protein 53 |  |  |  |
| CX070827 | S22597584 | 152 | 368 | 138.8244 | 402.9262 | 1.537253927 | 4 | phosphorylated carbohydrates phosphatase tm\_1254 |  |  |  |
| AY098891 | S22606173 | 1443 | 3762 | 1317.919 | 4119.044 | 1.644048033 | 4 | sucrose transport protein suc2 ame: full=sucrose permease 2 ame: full=sucrose-proton symporter 2 ame: full=sucrose transporter 1 |  |  |  |
| EY649645 | S44206807 | 27 | 9 | 24.6596 | 9.854173 | -1.323343017 | 4 | 70 kda peptidyl-prolyl isomerase ame: full=peptidyl-prolyl cis-trans isomerase� |  |  |  |
| EY653808 | S44209808 | 148 | 17 | 135.1712 | 18.61344 | -2.86037104 | 4 | cell division control protein 2 homolog d |  |  |  |
| EY701974 | S44244500 | 515 | 87 | 470.3591 | 95.257 | -2.303865642 | 4 | vacuolar cation proton exchanger 1 ame: full=ca(2+) h(+) exchanger 1 ame: full=ca(2+) h(+) antiporter cax1 ame: full=protein rare cold inducible 4 |  |  |  |
| EY704060 | S44246138 | 3930 | 2456 | 3589.342 | 2689.094 | -0.416599268 | 4 | pleiotropic drug resistance protein 1 ame: full= 1 |  |  |  |
| EY710755 | S44300374 | 38 | 96 | 34.70611 | 105.1112 | 1.598654471 | 4 | uncharacterized aarf domain-containing protein kinase chloroplastic flags: precursor |  |  |  |
| CX069649 | S22597043 | 71 | 22 | 64.84563 | 24.08798 | -1.428696017 | 3 | high mobility group family |  |  |  |
| CX302518 | S24635505 | 191 | 11 | 174.4439 | 12.04399 | -3.856377725 | 3 | ubiquitin |  |  |  |
| DR910057 | S26280934 | 5 | 23 | 4.566593 | 25.18289 | 2.463253345 | 3 | phosphoinositide phospholipase c 6 ame: full=phosphoinositide phospholipase plc6� |  |  |  |
| DY305494 | S34125056 | 421 | 3900 | 384.5072 | 4270.141 | 3.47320147 | 3 | probable calcium-binding protein cml45 ame: full=calmodulin-like protein 45 |  |  |  |
| DY306092 | S34125654 | 764 | 1751 | 697.7755 | 1917.184 | 1.458154024 | 3 | riboflavin biosynthesis protein chloroplastic includes: ame: full= -dihydroxy-2-butanone 4-phosphate synthase� |  |  |  |
| EY652956 | S44209348 | 446 | 113 | 407.3401 | 123.7246 | -1.719101453 | 3 | lys-63-specific deubiquitinase brcc36 ame: full=brca1-a complex subunit brcc36 ame: full=brisc complex subunit brcc36 ame: full=brca1 brca2-containing complex subunit 3 ame: full=brca1 brca2-containing complex subunit 36 |  |  |  |
| EY657959 | S44212993 | 113 | 268 | 103.205 | 293.4354 | 1.507529712 | 3 | protein |  |  |  |
| EY679459 | S44228053 | 24 | 2 | 21.91965 | 2.189816 | -3.323343017 | 3 | protein hothead ame: full=protein adhesion of calyx edges flags: precursor |  |  |  |
| EY688951 | S44234459 | 14 | 4 | 12.78646 | 4.379632 | -1.545735438 | 3 | actin-depolymerizing factor 5� |  |  |  |
| EY703151 | S44245453 | 99 | 37 | 90.41855 | 40.5116 | -1.15828377 | 3 | della protein gai ame: full=gibberellic acid-insensitive mutant protein |  |  |  |
| CB292707 | S22558205 | 1621 | 4387 | 1480.49 | 4803.362 | 1.6979701 | 2 | heat stress transcription factor b-2a� |  |  |  |
| CV714332 | S22576139 | 2 | 24 | 1.826637 | 26.27779 | 3.846581985 | 2 | dctp pyrophosphatase 1 ame: full=deoxycytidine-triphosphatase 1� |  |  |  |
| CX043308 | S22586138 | 13 | 0 | 11.87314 | 0 | -Inf | 2 | probable polyamine oxidase 5� |  |  |  |
| CX052342 | S22596007 | 10 | 2 | 9.133187 | 2.189816 | -2.060308611 | 2 | cyclin-d3-1 ame: full=g1 s-specific cyclin-d3-1� |  |  |  |
| CX071523 | S22602384 | 129 | 22 | 117.8181 | 24.08798 | -2.290176153 | 2 | probable auxin efflux carrier component 1c ame: full= 1c |  |  |  |
| CX673290 | S23019208 | 682 | 181 | 622.8833 | 198.1784 | -1.652162558 | 2 | ino1 gene |  |  |  |
| DN620167 | S24240472 | 52 | 139 | 47.49257 | 152.1922 | 1.680120839 | 2 | arogenate dehydrogenase chloroplastic ame: full= 2 flags: precursor |  |  |  |
| EY657484 | S44212630 | 8 | 29 | 7.306549 | 31.75233 | 2.119600479 | 2 | ser thr-rich protein t10 in dgcr region |  |  |  |
| EY657971 | S44213005 | 9 | 0 | 8.219868 | 0 | -Inf | 2 | kinesin-like protein kif22 ame: full=chromokinesin kid |  |  |  |
| EY676758 | S44225912 | 45 | 110 | 41.09934 | 120.4399 | 1.551126101 | 2 | zinc finger protein constans-like 15 |  |  |  |
| EY678067 | S44226885 | 69 | 171 | 63.01899 | 187.2293 | 1.570947542 | 2 | asparagine synthetase ame: full=glutamine-dependent asparagine synthetase |  |  |  |
| EY722375 | S44259274 | 837 | 176 | 764.4477 | 192.7038 | -1.98803271 | 2 | s-adenosylmethionine decarboxylase proenzyme� |  |  |  |
| EY723481 | S44260044 | 222 | 556 | 202.7567 | 608.7689 | 1.58614469 | 2 | siroheme synthase includes: ame: full=uroporphyrinogen-iii c-methyltransferase� |  |  |  |
| EY750241 | S44278826 | 2748 | 198 | 2509.8 | 216.7918 | -3.533190185 | 2 | brassinosteroid-regulated protein bru1 flags: precursor |  |  |  |
| EY650266 | S44284160 | 341 | 1315 | 311.4417 | 1439.804 | 2.208838639 | 2 | probable protein phosphatase 2c 25� |  |  |  |
| EY727159 | S44305753 | 309 | 916 | 282.2155 | 1002.936 | 1.829360244 | 2 | mitochondrial import inner membrane translocase subunit tim17 |  |  |  |
| EY727648 | S44305906 | 3813 | 888 | 3482.484 | 972.2784 | -1.840675465 | 2 | auxin efflux carrier family protein |  |  |  |
| EY747770 | S44311583 | 154 | 391 | 140.6511 | 428.1091 | 1.605857741 | 2 | transcription factor bim1 ame: full=bes1-interacting myc-like protein 1 ame: full=transcription factor en 126 ame: full=bhlh transcription factor bhlh046 ame: full=basic helix-loop-helix protein 46� |  |  |  |
| DC900331 | S47736628 | 76 | 19 | 69.41222 | 20.80325 | -1.738380516 | 2 | homeobox-leucine zipper protein athb-13 ame: full=homeodomain transcription factor athb-13 ame: full=hd-zip protein athb-13 |  |  |  |
| CN187848 | S22545642 | 778 | 2274 | 710.5619 | 2489.821 | 1.809009678 | 1 | dof zinc finger protein� |  |  |  |
| CN192429 | S22549656 | 141 | 302 | 128.7779 | 330.6622 | 1.360472871 | 1 | sulfate transporter ame: full=ast12 ame: full= 1 |  |  |  |
| CV719908 | S22579316 | 109 | 283 | 99.55174 | 309.859 | 1.638093402 | 1 | gtp-binding protein yptv3 |  |  |  |
| CV887297 | S22585971 | 70 | 4 | 63.93231 | 4.379632 | -3.867663533 | 1 | patellin-6 |  |  |  |
| CX044293 | S22588309 | 56 | 12 | 51.14585 | 13.1389 | -1.960772937 | 1 | adenine phosphoribosyltransferase 2� |  |  |  |
| CX047376 | S22593342 | 29 | 0 | 26.48624 | 0 | -Inf | 1 | probable xyloglucan endotransglucosylase hydrolase protein 33� |  |  |  |
| CX052350 | S22596008 | 3 | 15 | 2.739956 | 16.42362 | 2.583547579 | 1 | cysteine proteinase 15a ame: full=turgor-responsive protein 15a flags: precursor |  |  |  |
| CX053924 | S22596841 | 20 | 4 | 18.26637 | 4.379632 | -2.060308611 | 1 | kinesin-1 ame: full=kinesin-like protein a |  |  |  |
| CX078571 | S22606132 | 254 | 62 | 231.9829 | 67.8843 | -1.772868892 | 1 | peptidyl-trna hydrolase mitochondrial� |  |  |  |
| CX673824 | S23019466 | 183 | 381 | 167.1373 | 417.16 | 1.319566833 | 1 | chaperone protein dnaj 13� |  |  |  |
| DR404544 | S25679542 | 536 | 2134 | 489.5388 | 2336.534 | 2.254874754 | 1 | protein |  |  |  |
| DY257165 | S34124443 | 4897 | 13872 | 4472.522 | 15188.56 | 1.763825187 | 1 | probable ccr4-associated factor 1 homolog 9 |  |  |  |
| DY305851 | S34125413 | 10955 | 27867 | 10005.41 | 30511.8 | 1.608587698 | 1 | zinc finger protein 1 ame: full=wzf1 |  |  |  |
| DY305917 | S34125479 | 5372 | 12941 | 4906.348 | 14169.21 | 1.530037378 | 1 | populus trichocarpa ap2 erf domain-containing transcription factor mrna |  |  |  |
| EY655569 | S44211275 | 65 | 12 | 59.36571 | 13.1389 | -2.175785828 | 1 | 3-ketoacyl- synthase 19� |  |  |  |
| EY663018 | S44216260 | 33 | 7 | 30.13952 | 7.664356 | -1.975419713 | 1 | mterf family protein |  |  |  |
| EY666782 | S44219030 | 182 | 47 | 166.224 | 51.46068 | -1.691586304 | 1 | adenosine 3 -phospho 5 -phosphosulfate transporter 1 ame: full=paps transporter 1 ame: full=solute carrier family 35 member b2 ame: full= mapk-activating protein pm15 ame: full= nf-kappa-b-activating protein 48 |  |  |  |
| EY671382 | S44222188 | 1414 | 375 | 1291.433 | 410.5905 | -1.653200135 | 1 | fructose- - chloroplastic� |  |  |  |
| EY678113 | S44226931 | 208 | 515 | 189.9703 | 563.8777 | 1.569608388 | 1 | dof zinc finger protein� |  |  |  |
| EY690668 | S44235854 | 16 | 0 | 14.6131 | 0 | -Inf | 1 | uncharacterized amino acid permease yhdg |  |  |  |
| EY694066 | S44238146 | 1723 | 411 | 1573.648 | 450.0072 | -1.806092918 | 1 | chloroplast-targeted copper |  |  |  |
| EY702054 | S44244580 | 72 | 194 | 65.75895 | 212.4122 | 1.691607325 | 1 | tropinone reductase homolog at1g07440 |  |  |  |
| EY722059 | S44258958 | 259 | 744 | 236.5495 | 814.6116 | 1.783970008 | 1 | protein |  |  |  |
| EY650241 | S44284135 | 1161 | 5866 | 1060.363 | 6422.731 | 2.598628583 | 1 | zinc finger protein constans-like 1 |  |  |  |
| EY658428 | S44286330 | 95 | 277 | 86.76528 | 303.2895 | 1.805506042 | 1 | inositol 2-dehydrogenase ame: full=myo-inositol 2-dehydrogenase� |  |  |  |
| EY661115 | S44287113 | 42 | 93 | 38.35938 | 101.8265 | 1.408460872 | 1 | palmitoyl-monogalactosyldiacylglycerol delta-7 chloroplastic ame: full=monogalactosyldiacylglycerol-specific palmitic acid desaturase ame: full=fad5 flags: precursor |  |  |  |
| EY686801 | S44294717 | 35 | 8 | 31.96615 | 8.759265 | -1.867663533 | 1 | cyclin-d1-1 ame: full=g1 s-specific cyclin-d1-1� |  |  |  |
| EY748275 | S44311752 | 240 | 532 | 219.1965 | 582.4911 | 1.410011324 | 1 | protease 2 ame: full=protease ii ame: full=oligopeptidase b |  |  |  |
| BQ623132 | S22530951 | 3224 | 4366 | 2944.539 | 4780.369 | 0.699079871 | 0 | PREDICTED: hypothetical protein [Vitis vinifera] |  |  |  |
| BQ623495 | S22531314 | 13 | 4 | 11.87314 | 4.379632 | -1.438820234 | 0 | usda-fp\_00586 ridge pineapple sweet orange entire seedling citrus sinensis cdna clone usda-fp\_00586 5 mrna |  |  |  |
| BQ624431 | S22532250 | 654 | 574 | 597.3104 | 628.4772 | 0.073379585 | 0 | protein |  |  |  |
| BQ624514 | S22532333 | 1604 | 397 | 1464.963 | 434.6785 | -1.752843745 | 0 | gdsl esterase lipase at5g55050 ame: full=extracellular lipase at5g55050 flags: precursor |  |  |  |
| BQ625107 | S22532926 | 95 | 7 | 86.76528 | 7.664356 | -3.500881202 | 0 | protein |  |  |  |
| BQ625197 | S22533016 | 47 | 4 | 42.92598 | 4.379632 | -3.292969368 | 0 | sambucus nigra clone xp2 expansin complete cds |  |  |  |
| CK665407 | S22533806 | 15 | 47 | 13.69978 | 51.46068 | 1.90931774 | 0 | ap2 erf domain-containing transcription factor |  |  |  |
| CK665446 | S22533829 | 215 | 47 | 196.3635 | 51.46068 | -1.931984514 | 0 | conserved hypothetical protein [Ricinus communis] |  |  |  |
| CK701701 | S22534723 | 258 | 21 | 235.6362 | 22.99307 | -3.357290349 | 0 | usda-fp\_4962 ridge pineapple sweet orange entire seedling citrus sinensis cdna clone rse37e10 5 mrna |  |  |  |
| CK701747 | S22535105 | 17 | 3 | 15.52642 | 3.284724 | -2.240880856 | 0 | usda-fp\_5008 ridge pineapple sweet orange entire seedling citrus sinensis cdna clone rse39e04 5 mrna |  |  |  |
| CK739846 | S22536135 | 10 | 1 | 9.133187 | 1.094908 | -3.060308611 | 0 | elongation factor 1-alpha� |  |  |  |
| CK740205 | S22536342 | 28 | 62 | 25.57292 | 67.8843 | 1.408460872 | 0 | aquaporin tip1-3 ame: full=tonoplast intrinsic protein 1-3� |  |  |  |
| CK740222 | S22536764 | 4 | 60 | 3.653275 | 65.69448 | 4.16851008 | 0 | protein |  |  |  |
| CK932708 | S22536924 | 63 | 3 | 57.53908 | 3.284724 | -4.130697939 | 0 | endoglucanase 8 ame: full=endo- -beta glucanase 8 ame: full=cellulase 1� |  |  |  |
| CK932852 | S22537003 | 7 | 29 | 6.393231 | 31.75233 | 2.312245557 | 0 | cgf1004350\_f07 developing fruit juice sac at 38 dafb citrus sinensis cdna clone jsjune0004\_ivf\_f07 5 mrna |  |  |  |
| CK933074 | S22537170 | 12 | 1 | 10.95982 | 1.094908 | -3.323343017 | 0 | crambin precursor=thionin variant thi2ca9 |  |  |  |
| CK933841 | S22537808 | 43 | 7 | 39.2727 | 7.664356 | -2.357290349 | 0 | cgf1004278\_f03 developing fruit juice sac at 38 dafb citrus sinensis cdna clone jsjune0001\_if\_f03 5 mrna |  |  |  |
| CK933102 | S22537998 | 1 | 10 | 0.913319 | 10.94908 | 3.583547579 | 0 | cgf1004347\_f02 developing fruit juice sac at 38 dafb citrus sinensis cdna clone jsjune0004\_if\_f02 5 mrna |  |  |  |
| CK933909 | S22538125 | 12 | 81 | 10.95982 | 88.68755 | 3.016506986 | 0 | �gem-like protein 8 |  |  |  |
| CK933934 | S22538150 | 741 | 153 | 676.7691 | 167.5209 | -2.014322406 | 0 | conserved hypothetical protein [Ricinus communis] |  |  |  |
| CK934089 | S22538305 | 10 | 37 | 9.133187 | 40.5116 | 2.149144755 | 0 | medicago truncatula chromosome 7 clone mth2- complete sequence |  |  |  |
| CK934405 | S22538583 | 20 | 5 | 18.26637 | 5.47454 | -1.738380516 | 0 | protein |  |  |  |
| CK934874 | S22539052 | 294 | 324 | 268.5157 | 354.7502 | 0.401797142 | 0 | cgf1004225\_h01 developing fruit peel at 38 dafb citrus sinensis cdna clone p38dab10003\_ivf\_h01 5 mrna |  |  |  |
| CK934971 | S22539149 | 69 | 8 | 63.01899 | 8.759265 | -2.846904973 | 0 | protein |  |  |  |
| CK935156 | S22539338 | 4 | 9 | 3.653275 | 9.854173 | 1.431544486 | 0 | cgf1004177\_c06 developing fruit peel at 38 dafb citrus sinensis cdna clone p38da0001\_iif\_c06 5 mrna |  |  |  |
| CK935574 | S22539458 | 2 | 17 | 1.826637 | 18.61344 | 3.349082325 | 0 | protein |  |  |  |
| CK935203 | S22539523 | 86 | 209 | 78.54541 | 228.8358 | 1.542713861 | 0 | protein |  |  |  |
| CK935206 | S22539526 | 3 | 2 | 2.739956 | 2.189816 | -0.323343017 | 0 | chloroplast envelope membrane 70 kda heat shock-related protein |  |  |  |
| CK935295 | S22539605 | 163 | 457 | 148.8709 | 500.373 | 1.748941685 | 0 | diphthamide biosynthesis protein 3 |  |  |  |
| CK935341 | S22539631 | 502 | 939 | 458.486 | 1028.119 | 1.165057278 | 0 | unknown [Populus trichocarpa] |  |  |  |
| CK935394 | S22539659 | 21 | 66 | 19.17969 | 72.26393 | 1.913696181 | 0 | cgf1004562\_c10 developing fruit 24 dafb citrus sinensis cdna clone t24dab0001\_if\_c10 5 mrna |  |  |  |
| CK935405 | S22539665 | 150 | 23 | 136.9978 | 25.18289 | -2.44363725 | 0 | unknown [Glycine max] |  |  |  |
| CK935415 | S22539670 | 7 | 23 | 6.393231 | 25.18289 | 1.977826518 | 0 | cgf1004555\_h09 developing fruit 24 dafb citrus sinensis cdna clone t24dab0004\_ivf\_h09 5 mrna |  |  |  |
| CK935639 | S22539754 | 0 | 7 | 0 | 7.664356 | Inf | 0 | leishmania infantum chromosome 3 |  |  |  |
| CK935682 | S22539794 | 27 | 111 | 24.6596 | 121.5348 | 2.301147848 | 0 | cgf1004552\_h09 developing fruit 24 dafb citrus sinensis cdna clone t24dab0004\_if\_h09 5 mrna |  |  |  |
| CK935746 | S22539858 | 2827 | 664 | 2581.952 | 727.019 | -1.828397252 | 0 | snakin-2 flags: precursor |  |  |  |
| CK935886 | S22539998 | 134 | 1517 | 122.3847 | 1660.976 | 3.762535664 | 0 | protein |  |  |  |
| CK936202 | S22540314 | 1881 | 295 | 1717.952 | 322.9979 | -2.411093505 | 0 | phosphoenolpyruvate carboxykinase� |  |  |  |
| CK936343 | S22540460 | 711 | 44 | 649.3696 | 48.17595 | -3.752654647 | 0 | subtilisin-like protease ame: full=cucumisin-like serine protease flags: precursor |  |  |  |
| CK936936 | S22540813 | 149 | 117 | 136.0845 | 128.1042 | -0.087184317 | 0 | predicted protein [Populus trichocarpa] |  |  |  |
| CK937203 | S22541313 | 6 | 12 | 5.479912 | 13.1389 | 1.261619484 | 0 | cgf1004512\_h11 developing fruit flavedo at 80 dafb citrus sinensis cdna clone f80dab0003\_iiif\_h11 5 mrna |  |  |  |
| CK937278 | S22541382 | 878 | 231 | 801.8938 | 252.9238 | -1.664708604 | 0 | inhibitor of trypsin and hageman factor ame: full=cmti-v |  |  |  |
| CK937531 | S22541736 | 39 | 5 | 35.61943 | 5.47454 | -2.70185464 | 0 | cgf1004779\_d06 developing fruit albedo at 80 dafb in p x2 vector citrus sinensis cdna clone a80dab0003\_iiif\_d06 5 mrna |  |  |  |
| CK938060 | S22542085 | 35 | 65 | 31.96615 | 71.16902 | 1.15470428 | 0 | cgf1004478\_g03 developing fruit albedo at 80 dafb in p x2 vector citrus sinensis cdna clone a80dab0002\_ivf\_g03 5 mrna |  |  |  |
| CK937616 | S22542317 | 31 | 84 | 28.31288 | 91.97228 | 1.699740596 | 0 | cgf1004778\_c02 developing fruit albedo at 80 dafb in p x2 vector citrus sinensis cdna clone a80dab0003\_iif\_c02 5 mrna |  |  |  |
| CK938211 | S22542508 | 250 | 595 | 228.3297 | 651.4703 | 1.512581058 | 0 | ethylene-responsive transcription factor erf010 |  |  |  |
| CK938229 | S22542521 | 0 | 20 | 0 | 21.89816 | Inf | 0 | cgf1004476\_e07 developing fruit albedo at 80 dafb in p x2 vector citrus sinensis cdna clone a80dab0002\_iif\_e07 5 mrna |  |  |  |
| CK938307 | S22542548 | 7 | 20 | 6.393231 | 21.89816 | 1.776192657 | 0 | poncirus trifoliata citrus tristeza virus resistance gene complete sequence |  |  |  |
| CK938850 | S22542588 | 64 | 11 | 58.4524 | 12.04399 | -2.278948897 | 0 | cgf1004437\_e06 developing fruit albedo at 165 dafb citrus sinensis cdna clone a1650002\_iif\_e06 5 mrna |  |  |  |
| CK938946 | S22542684 | 53 | 138 | 48.40589 | 151.0973 | 1.642223486 | 0 | cgf1004436\_c07 developing fruit albedo at 165 dafb citrus sinensis cdna clone a1650002\_if\_c07 5 mrna |  |  |  |
| CK939006 | S22542755 | 55 | 148 | 50.23253 | 162.0464 | 1.689713136 | 0 | cgf1004435\_e06 developing fruit albedo at 165 dafb citrus sinensis cdna clone a1650003\_ivf\_e06 5 mrna |  |  |  |
| CK939007 | S22542756 | 89 | 11 | 81.28536 | 12.04399 | -2.754682328 | 0 | peptide transporter ptr5 |  |  |  |
| CK938998 | S22542763 | 9 | 23 | 8.219868 | 25.18289 | 1.615256439 | 0 | cgf1004435\_f06 developing fruit albedo at 165 dafb citrus sinensis cdna clone a1650003\_ivf\_f06 5 mrna |  |  |  |
| CK939180 | S22543193 | 2 | 17 | 1.826637 | 18.61344 | 3.349082325 | 0 | vitis vinifera contig whole genome shotgun sequence |  |  |  |
| CK939266 | S22543283 | 145 | 38 | 132.4312 | 41.60651 | -1.670362092 | 0 | cgf1004432\_c03 developing fruit albedo at 165 dafb citrus sinensis cdna clone a1650003\_if\_c03 5 mrna |  |  |  |
| CK938405 | S22543309 | 11 | 47 | 10.04651 | 51.46068 | 2.356776717 | 0 | cgf1004443\_c10 developing fruit albedo at 165 dafb citrus sinensis cdna clone a1650001\_ivf\_c10 5 mrna |  |  |  |
| CK938667 | S22543424 | 243 | 635 | 221.9364 | 695.2666 | 1.647419762 | 0 | protein |  |  |  |
| CK939541 | S22543662 | 467 | 1255 | 426.5198 | 1374.11 | 1.687812393 | 0 | ethylene-responsive transcription factor 1a� |  |  |  |
| CK939658 | S22543787 | 9 | 23 | 8.219868 | 25.18289 | 1.615256439 | 0 | cgf1004747\_d06 developing fruit flavedo at 165 dafb citrus sinensis cdna clone f1650002\_iiif\_d06 5 mrna |  |  |  |
| CK939678 | S22543802 | 106 | 253 | 96.81178 | 277.0117 | 1.516692604 | 0 | hypothetical protein [Vitis vinifera] |  |  |  |
| CK939668 | S22543803 | 7 | 41 | 6.393231 | 44.89123 | 2.811816567 | 0 | cgf1004747\_c07 developing fruit flavedo at 165 dafb citrus sinensis cdna clone f1650002\_iiif\_c07 5 mrna |  |  |  |
| CK939746 | S22543868 | 1734 | 3868 | 1583.695 | 4235.104 | 1.41910338 | 0 | arabinogalactan peptide 16� |  |  |  |
| CK939814 | S22543943 | 15 | 4 | 13.69978 | 4.379632 | -1.645271112 | 0 | cgf1004745\_c08 developing fruit flavedo at 165 dafb citrus sinensis cdna clone f1650002\_if\_c08 5 mrna |  |  |  |
| CK939844 | S22543970 | 677 | 110 | 618.3168 | 120.4399 | -2.360032826 | 0 | cgf1004744\_h08 developing fruit flavedo at 165 dafb citrus sinensis cdna clone f1650003\_ivf\_h08 5 mrna |  |  |  |
| CK940014 | S22544138 | 23 | 131 | 21.00633 | 143.433 | 2.77148053 | 0 | cgf1004742\_e04 developing fruit flavedo at 165 dafb citrus sinensis cdna clone f1650003\_iif\_e04 5 mrna |  |  |  |
| CK940099 | S22544223 | 126 | 825 | 115.0782 | 903.2992 | 2.97258987 | 0 | calcium-binding protein cml37 ame: full=calmodulin-like protein 37 |  |  |  |
| CN185363 | S22544451 | 2740 | 2613 | 2502.493 | 2860.995 | 0.193150716 | 0 | vitis vinifera contig whole genome shotgun sequence |  |  |  |
| CN185762 | S22544619 | 2063 | 5196 | 1884.176 | 5689.142 | 1.594277094 | 0 | copper transporter 1 |  |  |  |
| CN185801 | S22544640 | 5 | 58 | 4.566593 | 63.50467 | 3.797672384 | 0 | probable lrr receptor-like serine threonine-protein kinase at2g23950 flags: precursor |  |  |  |
| CN186662 | S22545043 | 21 | 247 | 19.17969 | 270.4423 | 3.817669293 | 0 | indole-3-acetic acid-amido synthetase ame: full=auxin-responsive gh3-like protein 4� |  |  |  |
| CN187647 | S22545540 | 11 | 54 | 10.04651 | 59.12504 | 2.557075368 | 0 | predicted protein [Populus trichocarpa] |  |  |  |
| CN186015 | S22546485 | 5 | 27 | 4.566593 | 29.56252 | 2.694578891 | 0 | protein |  |  |  |
| CN186149 | S22546539 | 349 | 2327 | 318.7482 | 2547.851 | 2.998791753 | 0 | protein |  |  |  |
| CN188179 | S22547436 | 197 | 529 | 179.9238 | 579.2064 | 1.686691577 | 0 | unknown protein [Oryza sativa Japonica Group] |  |  |  |
| CN188518 | S22547586 | 390 | 1029 | 356.1943 | 1126.66 | 1.661316437 | 0 | �kda class i heat shock protein ame: full= |  |  |  |
| CN188941 | S22547810 | 131 | 28 | 119.6447 | 30.65743 | -1.964448595 | 0 | predicted protein [Populus trichocarpa] |  |  |  |
| CN190127 | S22548431 | 36 | 314 | 32.87947 | 343.8011 | 3.386315232 | 0 | ucrcs06\_0002p09\_r washington navel orange stored fruit rind cdna library citrus sinensis cdna clone mrna |  |  |  |
| CN191394 | S22549085 | 398 | 1651 | 363.5008 | 1807.693 | 2.314119268 | 0 | protein |  |  |  |
| CN191560 | S22549168 | 316 | 979 | 288.6087 | 1071.915 | 1.893003786 | 0 | vitis vinifera contig whole genome shotgun sequence |  |  |  |
| CN191758 | S22549271 | 13 | 38 | 11.87314 | 41.60651 | 1.809107279 | 0 | lipid binding |  |  |  |
| CN189156 | S22549853 | 32 | 84 | 29.2262 | 91.97228 | 1.653936907 | 0 | myb-like protein j |  |  |  |
| CN190565 | S22550478 | 5 | 24 | 4.566593 | 26.27779 | 2.52465389 | 0 | ucrcs06\_0003k21\_f washington navel orange stored fruit rind cdna library citrus sinensis cdna clone mrna |  |  |  |
| CN191678 | S22550905 | 68 | 286 | 62.10567 | 313.1437 | 2.33402798 | 0 | ucrcs06\_0005k01\_f washington navel orange stored fruit rind cdna library citrus sinensis cdna clone mrna |  |  |  |
| CN182507 | S22551834 | 565 | 1312 | 516.0251 | 1436.519 | 1.477064431 | 0 | ucrcs04\_0002j05\_r ruby orange developing flower cdna library citrus sinensis cdna clone mrna |  |  |  |
| CN181701 | S22553417 | 14 | 58 | 12.78646 | 63.50467 | 2.312245557 | 0 | peroxidase 60� |  |  |  |
| CN185028 | S22554792 | 58 | 280 | 52.97248 | 306.5743 | 2.532921506 | 0 | ucrcs04\_0006j12\_r ruby orange developing flower cdna library citrus sinensis cdna clone mrna |  |  |  |
| CB250257 | S22554989 | 3 | 0 | 2.739956 | 0 | -Inf | 0 | polygalacturonase� |  |  |  |
| CB291261 | S22555615 | 5202 | 5508 | 4751.084 | 6030.754 | 0.344081644 | 0 | PREDICTED: hypothetical protein [Vitis vinifera] |  |  |  |
| CB291342 | S22555660 | 9 | 42 | 8.219868 | 45.98614 | 2.484011905 | 0 | aluminum-activated malate transporter-like |  |  |  |
| CB291413 | S22555698 | 6 | 51 | 5.479912 | 55.84031 | 3.349082325 | 0 | ucrcs01\_02de03\_g1 washington navel orange cold acclimated flavedo & albedo cdna library citrus sinensis cdna clone mrna |  |  |  |
| CB291509 | S22555752 | 38 | 95 | 34.70611 | 104.0163 | 1.583547579 | 0 | ucrcs01\_03ab09\_b1 washington navel orange cold acclimated flavedo & albedo cdna library citrus sinensis cdna clone mrna |  |  |  |
| CB291917 | S22555985 | 159 | 379 | 145.2177 | 414.9702 | 1.514790567 | 0 | scarecrow-like protein 31� |  |  |  |
| CB292168 | S22556123 | 2 | 9 | 1.826637 | 9.854173 | 2.431544486 | 0 | ucrcs01\_04ac10\_g1 washington navel orange cold acclimated flavedo & albedo cdna library citrus sinensis cdna clone mrna |  |  |  |
| CB292276 | S22556183 | 31 | 152 | 28.31288 | 166.426 | 2.555350687 | 0 | protein |  |  |  |
| CB292505 | S22556310 | 1122 | 2627 | 1024.744 | 2876.323 | 1.488963009 | 0 | f-box family protein |  |  |  |
| CB292559 | S22556340 | 287 | 1078 | 262.1225 | 1180.311 | 2.17085402 | 0 | serine threonine-protein kinase 1 6 |  |  |  |
| CB293231 | S22556716 | 3 | 10 | 2.739956 | 10.94908 | 1.998585078 | 0 | protein |  |  |  |
| CB293368 | S22556792 | 6 | 120 | 5.479912 | 131.389 | 4.583547579 | 0 | ucrcs01\_06aa05\_g1 washington navel orange cold acclimated flavedo & albedo cdna library citrus sinensis cdna clone mrna |  |  |  |
| CB293734 | S22556991 | 343 | 1507 | 313.2683 | 1650.026 | 2.39701842 | 0 | ucrcs01\_06cd04\_g1 washington navel orange cold acclimated flavedo & albedo cdna library citrus sinensis cdna clone mrna |  |  |  |
| CB293888 | S22557077 | 3 | 14 | 2.739956 | 15.32871 | 2.484011905 | 0 | ucrcs01\_06de04\_b1 washington navel orange cold acclimated flavedo & albedo cdna library citrus sinensis cdna clone mrna |  |  |  |
| CB290283 | S22557188 | 13 | 9 | 11.87314 | 9.854173 | -0.268895233 | 0 | af320906citrus unshiu metallothionein-like protein complete cds |  |  |  |
| CB290464 | S22557260 | 61893 | 144435 | 56528.03 | 158143 | 1.48419171 | 0 | late embryogenesis abundant protein lea5 |  |  |  |
| CB290495 | S22557275 | 919 | 2564 | 839.3399 | 2807.344 | 1.741878979 | 0 | protein |  |  |  |
| CB291071 | S22557523 | 110 | 235 | 100.4651 | 257.3034 | 1.356776717 | 0 | acyl- chloroplastic ame: full=stearoyl-acp desaturase flags: precursor |  |  |  |
| CB291292 | S22557611 | 67 | 278 | 61.19235 | 304.3844 | 2.314471366 | 0 | circadian clock coupling factor zgt |  |  |  |
| CB291669 | S22557768 | 4 | 20 | 3.653275 | 21.89816 | 2.583547579 | 0 | ucrcs01\_03bb10\_g1 washington navel orange cold acclimated flavedo & albedo cdna library citrus sinensis cdna clone mrna |  |  |  |
| CB292401 | S22558075 | 262 | 863 | 239.2895 | 944.9057 | 1.981413232 | 0 | 40s ribosomal protein s24-1 |  |  |  |
| CB292793 | S22558243 | 413 | 893 | 377.2006 | 977.7529 | 1.374137878 | 0 | predicted protein [Populus trichocarpa] |  |  |  |
| CB293236 | S22558423 | 39 | 183 | 35.61943 | 200.3682 | 2.491917104 | 0 | nac domain-containing protein 71� |  |  |  |
| CB293811 | S22558665 | 2125 | 5019 | 1940.802 | 5495.344 | 1.501556589 | 0 | ucrcs01\_06da01\_b1 washington navel orange cold acclimated flavedo & albedo cdna library citrus sinensis cdna clone mrna |  |  |  |
| CB304390 | S22558759 | 9 | 34 | 8.219868 | 37.22687 | 2.179157324 | 0 | flavedo0001\_ii \_b07 flavedo mature citrus sinensis cdna clone flavedo0001\_ii \_b07 3 mrna |  |  |  |
| CB304569 | S22558908 | 437 | 476 | 399.1203 | 521.1762 | 0.384947778 | 0 | flavedo0001\_i \_g03 flavedo mature citrus sinensis cdna clone flavedo0001\_i \_g03 3 mrna |  |  |  |
| CB304607 | S22558946 | 307 | 885 | 280.3888 | 968.9936 | 1.789058284 | 0 | ethylene-responsive transcription factor erf061 |  |  |  |
| CB304637 | S22558976 | 1 | 11 | 0.913319 | 12.04399 | 3.721051103 | 0 | flavedo0001\_ \_g06 flavedo mature citrus sinensis cdna clone flavedo0001\_ \_g06 3 mrna |  |  |  |
| CB610680 | S22560093 | 6 | 10 | 5.479912 | 10.94908 | 0.998585078 | 0 | albedo0003\_ii \_c11 mature albedo citrus sinensis cdna clone albedo0003\_ii \_c11 5 mrna |  |  |  |
| CB610773 | S22560161 | 4 | 10 | 3.653275 | 10.94908 | 1.583547579 | 0 | albedo0002\_ \_a02 mature albedo citrus sinensis cdna clone albedo0002\_ \_a02 5 mrna |  |  |  |
| CB611027 | S22560606 | 4223 | 5125 | 3856.945 | 5611.404 | 0.540903242 | 0 | albedo0001\_ \_c05 mature albedo citrus sinensis cdna clone albedo0001\_ \_c05 5 mrna |  |  |  |
| CF417791 | S22561156 | 82 | 18 | 74.89213 | 19.70835 | -1.926007519 | 0 | cytochrome p450 716b1 ame: full=cytochrome p450 cypa1 |  |  |  |
| CF417159 | S22561371 | 20 | 5 | 18.26637 | 5.47454 | -1.738380516 | 0 | conserved hypothetical protein [Ricinus communis] |  |  |  |
| CF506091 | S22563940 | 576 | 283 | 526.0716 | 309.859 | -0.763647275 | 0 | c31708e05ef lemenn citrus clementina cdna clone mrna |  |  |  |
| CF507764 | S22565613 | 476 | 2874 | 434.7397 | 3146.766 | 2.855646067 | 0 | usda-fp\_123000-265 immature ovaries from field-collected valencia sweet orange (citrus sinensis ( ) osbeck) citrus sinensis cdna clone mvf-60\_c08 5 mrna |  |  |  |
| CF508975 | S22566824 | 18 | 181 | 16.43974 | 198.1784 | 3.59154037 | 0 | u-box domain-containing protein 19 ame: full=plant u-box protein 19 |  |  |  |
| CF509967 | S22567816 | 319 | 72 | 291.3487 | 78.83338 | -1.885868128 | 0 | sugar carrier protein c |  |  |  |
| CF510057 | S22567906 | 17 | 1 | 15.52642 | 1.094908 | -3.825843357 | 0 | kinesin-4 ame: full=kinesin-like protein d |  |  |  |
| CF832354 | S22568325 | 1087 | 1629 | 992.7774 | 1783.605 | 0.845254148 | 0 | ucrcs02\_01l18\_f ruby orange ovary at anthesis cdna library citrus sinensis cdna clone mrna |  |  |  |
| CF833196 | S22568730 | 15 | 41 | 13.69978 | 44.89123 | 1.712280893 | 0 | ucrcs02\_03d03\_r ruby orange ovary at anthesis cdna library citrus sinensis cdna clone mrna |  |  |  |
| CF833251 | S22568754 | 2 | 10 | 1.826637 | 10.94908 | 2.583547579 | 0 | hypothetical protein [Populus tremula] |  |  |  |
| CF833296 | S22568777 | 97 | 16 | 88.59191 | 17.51853 | -2.338293358 | 0 | ucrcs02\_03g01\_r ruby orange ovary at anthesis cdna library citrus sinensis cdna clone mrna |  |  |  |
| CF833645 | S22568937 | 33 | 1 | 30.13952 | 1.094908 | -4.782774635 | 0 | protein |  |  |  |
| CF832225 | S22569883 | 10 | 2 | 9.133187 | 2.189816 | -2.060308611 | 0 | ucrcs02\_01i08\_r ruby orange ovary at anthesis cdna library citrus sinensis cdna clone mrna |  |  |  |
| CF834184 | S22570834 | 141 | 34 | 128.7779 | 37.22687 | -1.790469027 | 0 | populus trichocarpa mrna |  |  |  |
| CF834198 | S22570840 | 196 | 880 | 179.0105 | 963.5191 | 2.428269353 | 0 | ring-h2 finger protein atl3b ame: full=ring-h2 finger protein atl6 flags: precursor |  |  |  |
| CF834253 | S22570867 | 23 | 60 | 21.00633 | 65.69448 | 1.644948124 | 0 | hat dimerisation domain-containing protein |  |  |  |
| CF834267 | S22570874 | 9 | 30 | 8.219868 | 32.84724 | 1.998585078 | 0 | lob domain-containing protein 38 ame: full=asymmetric leaves 2-like protein 40� |  |  |  |
| CF834421 | S22570947 | 139 | 7 | 126.9513 | 7.664356 | -4.049966667 | 0 | 3-ketoacyl- synthase 11� |  |  |  |
| CF834616 | S22571037 | 19 | 4 | 17.35306 | 4.379632 | -1.986308029 | 0 | protein |  |  |  |
| CF836416 | S22571911 | 382 | 4387 | 348.8877 | 4803.362 | 3.783209647 | 0 | ucrcs03\_02o21\_f washington navel orange shoot meristem cdna library citrus sinensis cdna clone mrna |  |  |  |
| CF836738 | S22572065 | 9 | 2 | 8.219868 | 2.189816 | -1.908305517 | 0 | retrovirus-related pol polyprotein from transposon tnt 1-94 includes: ame: full=protease includes: ame: full=reverse transcriptase includes: ame: full=endonuclease |  |  |  |
| CF837114 | S22572251 | 18 | 11 | 16.43974 | 12.04399 | -0.448873899 | 0 | ucrcs03\_04c02\_f washington navel orange shoot meristem cdna library citrus sinensis cdna clone mrna |  |  |  |
| CF837816 | S22572593 | 312 | 401 | 284.9554 | 439.0581 | 0.623675692 | 0 | umecyanin� |  |  |  |
| CF838781 | S22573068 | 32 | 5 | 29.2262 | 5.47454 | -2.416452421 | 0 | ucrcs03\_06j10\_f washington navel orange shoot meristem cdna library citrus sinensis cdna clone mrna |  |  |  |
| CF835360 | S22573161 | 54 | 213 | 49.31921 | 233.2154 | 2.241441602 | 0 | ucrcs03\_01b20\_r washington navel orange shoot meristem cdna library citrus sinensis cdna clone mrna |  |  |  |
| CF836098 | S22573518 | 190 | 19 | 173.5306 | 20.80325 | -3.060308611 | 0 | ice binding |  |  |  |
| CF837348 | S22574131 | 1108 | 5561 | 1011.957 | 6088.784 | 2.58900594 | 0 | PREDICTED: hypothetical protein [Vitis vinifera] |  |  |  |
| CF837571 | S22574240 | 136 | 355 | 124.2113 | 388.6924 | 1.645831857 | 0 | u-box domain-containing protein 16 ame: full=plant u-box protein 16 |  |  |  |
| CF838077 | S22574489 | 5 | 11 | 4.566593 | 12.04399 | 1.399123008 | 0 | protein |  |  |  |
| CF838214 | S22574550 | 106 | 25 | 96.81178 | 27.3727 | -1.822444781 | 0 | disease resistance response protein 206 |  |  |  |
| CF838525 | S22574705 | 91 | 301 | 83.112 | 329.5673 | 1.987444521 | 0 | protein |  |  |  |
| CV713105 | S22575445 | 10 | 28 | 9.133187 | 30.65743 | 1.747046311 | 0 | ucrcs08\_0002f09\_r parent washington navel orange callus cdna library ucrcs08-1 citrus sinensis cdna clone mrna |  |  |  |
| CV713409 | S22575615 | 13 | 32 | 11.87314 | 35.03706 | 1.561179766 | 0 | AF506028\_16hypothetical protein [Poncirus trifoliata] |  |  |  |
| CV714710 | S22576355 | 182 | 574 | 166.224 | 628.4772 | 1.918731771 | 0 | protein |  |  |  |
| CV715903 | S22577038 | 35 | 4 | 31.96615 | 4.379632 | -2.867663533 | 0 | wound induced protein |  |  |  |
| CV717430 | S22577908 | 188 | 41 | 171.7039 | 44.89123 | -1.935417363 | 0 | cytokinin-o-glucosyltransferase 2 ame: full=zeatin o-glucosyltransferase 2� |  |  |  |
| CV719008 | S22578804 | 15 | 45 | 13.69978 | 49.27086 | 1.846581985 | 0 | ucrcs08\_0011f06\_r parent washington navel orange callus cdna library ucrcs08-1 citrus sinensis cdna clone mrna |  |  |  |
| CV719693 | S22579191 | 1 | 0 | 0.913319 | 0 | -Inf | 0 | ucrcs08\_0012f04\_f parent washington navel orange callus cdna library ucrcs08-1 citrus sinensis cdna clone mrna |  |  |  |
| CV719978 | S22579356 | 36 | 131 | 32.87947 | 143.433 | 2.125117484 | 0 | uncharacterized protein at4g13230 flags: precursor |  |  |  |
| CV719989 | S22579361 | 88 | 19 | 80.37204 | 20.80325 | -1.949884621 | 0 | protein |  |  |  |
| CV720001 | S22579367 | 408 | 107 | 372.634 | 117.1552 | -1.669338871 | 0 | ucrcs08\_0012m10\_r parent washington navel orange callus cdna library ucrcs08-1 citrus sinensis cdna clone mrna |  |  |  |
| CV713065 | S22579858 | 331 | 1155 | 302.3085 | 1264.619 | 2.064609214 | 0 | taxadien-5-alpha-ol o-acetyltransferase ame: full=taxa-4 -dien-5alpha-ol-o-acetyltransferase� |  |  |  |
| CV713122 | S22579883 | 57 | 0 | 52.05917 | 0 | -Inf | 0 | glucan endo- -beta-glucosidase ame: full=(1- |  |  |  |
| CV713559 | S22580072 | 17 | 37 | 15.52642 | 40.5116 | 1.383610008 | 0 | hypothetical protein NitaMp116 [Nicotiana tabacum] |  |  |  |
| CV715444 | S22580876 | 79 | 14 | 72.15218 | 15.32871 | -2.234806342 | 0 | ucrcs08\_0005m06\_f parent washington navel orange callus cdna library ucrcs08-1 citrus sinensis cdna clone mrna |  |  |  |
| CV716021 | S22581123 | 7 | 40 | 6.393231 | 43.79632 | 2.776192657 | 0 | ucrcs08\_0006l01\_r parent washington navel orange callus cdna library ucrcs08-1 citrus sinensis cdna clone mrna |  |  |  |
| CV716258 | S22581222 | 81 | 183 | 73.97881 | 200.3682 | 1.437469319 | 0 | ucrcs08\_0007a15\_f parent washington navel orange callus cdna library ucrcs08-1 citrus sinensis cdna clone mrna |  |  |  |
| CV718150 | S22582030 | 3 | 16 | 2.739956 | 17.51853 | 2.676656983 | 0 | protein |  |  |  |
| CV718401 | S22582137 | 3 | 13 | 2.739956 | 14.2338 | 2.377096701 | 0 | ucrcs08\_0010h03\_f parent washington navel orange callus cdna library ucrcs08-1 citrus sinensis cdna clone mrna |  |  |  |
| CV718780 | S22582298 | 66 | 6 | 60.27903 | 6.569448 | -3.197812135 | 0 | plz12\_luppoprotein pplz12 |  |  |  |
| CV718921 | S22582359 | 41 | 127 | 37.44607 | 139.0533 | 1.892752166 | 0 | non-symbiotic hemoglobin 1 ame: full=medsa glb1 |  |  |  |
| CV719187 | S22582473 | 318 | 678 | 290.4353 | 742.3477 | 1.353877992 | 0 | integrase |  |  |  |
| CV719618 | S22582656 | 49 | 12 | 44.75262 | 13.1389 | -1.768127859 | 0 | ucrcs08\_0012d12\_r parent washington navel orange callus cdna library ucrcs08-1 citrus sinensis cdna clone mrna |  |  |  |
| CV719871 | S22582765 | 5 | 24 | 4.566593 | 26.27779 | 2.52465389 | 0 | transcription factor bhlh113 ame: full=transcription factor en 61 ame: full=bhlh transcription factor bhlh113 ame: full=basic helix-loop-helix protein 113� |  |  |  |
| CV884948 | S22583236 | 171 | 506 | 156.1775 | 554.0235 | 1.826760544 | 0 | ucrcs04\_2\_011e10\_t3 ruby orange developing flower cdna library ucrcs04-ucr citrus sinensis cdna clone mrna |  |  |  |
| CV885148 | S22583331 | 9 | 21 | 8.219868 | 22.99307 | 1.484011905 | 0 | ucrcs04\_2\_012h11\_t7 ruby orange developing flower cdna library ucrcs04-ucr citrus sinensis cdna clone mrna |  |  |  |
| CV886525 | S22583988 | 211 | 1051 | 192.7102 | 1150.748 | 2.578067249 | 0 | zinc finger protein constans-like 10 |  |  |  |
| CV885396 | S22585018 | 355 | 60 | 324.2281 | 65.69448 | -2.303165135 | 0 | protein |  |  |  |
| CV887374 | S22585048 | 0 | 12 | 0 | 13.1389 | Inf | 0 | ucrcs04\_2\_030h07\_t3 ruby orange developing flower cdna library ucrcs04-ucr citrus sinensis cdna clone mrna |  |  |  |
| CV886254 | S22585416 | 295 | 827 | 269.429 | 905.489 | 1.748791859 | 0 | predicted protein [Populus trichocarpa] |  |  |  |
| CV886709 | S22585659 | 5 | 14 | 4.566593 | 15.32871 | 1.747046311 | 0 | serine carboxypeptidase-like 18 flags: precursor |  |  |  |
| CV886949 | S22585789 | 240 | 385 | 219.1965 | 421.5396 | 0.943443524 | 0 | blue copper protein flags: precursor |  |  |  |
| CX044934 | S22586893 | 4 | 13 | 3.653275 | 14.2338 | 1.962059202 | 0 | ring-h2 finger protein atl1k ame: full=ring-h2 finger protein atl10 |  |  |  |
| CX046065 | S22587428 | 194 | 1352 | 177.1838 | 1480.316 | 3.062586078 | 0 | reticuline oxidase-like protein flags: precursor |  |  |  |
| CX046482 | S22587628 | 17 | 4 | 15.52642 | 4.379632 | -1.825843357 | 0 | protein |  |  |  |
| CX044553 | S22588449 | 35 | 50 | 31.96615 | 54.7454 | 0.776192657 | 0 | ucrcs07\_18c09\_b parent washington navel orange thrip-challenged flavedo cdna library ucrcs07 citrus sinensis cdna clone ucrcs07-18c09-f18-1- mrna |  |  |  |
| CX045330 | S22588860 | 7 | 118 | 6.393231 | 129.1992 | 4.336907611 | 0 | ucrcs07\_22b08\_b parent washington navel orange thrip-challenged flavedo cdna library ucrcs07 citrus sinensis cdna clone ucrcs07-22b08-d16-1- mrna |  |  |  |
| CX045334 | S22588865 | 31 | 82 | 28.31288 | 89.78246 | 1.664975178 | 0 | jasmonate o-methyltransferase ame: full=s-adenosyl-l-methionine:jasmonic acid carboxyl methyltransferase ame: full=floral nectary-specific protein 1 |  |  |  |
| CX045473 | S22588938 | 746 | 1646 | 681.3357 | 1802.219 | 1.403336284 | 0 | protein |  |  |  |
| CX045842 | S22589131 | 410 | 36 | 374.4607 | 39.41669 | -3.247935614 | 0 | unknown protein [Arabidopsis thaliana] |  |  |  |
| CX046356 | S22589397 | 242 | 24 | 221.0231 | 26.27779 | -3.072281252 | 0 | receptor protein kinase clavata1 flags: precursor |  |  |  |
| CX046518 | S22589484 | 178 | 42 | 162.5707 | 45.98614 | -1.821796524 | 0 | fatty acyl- reductase 3 ame: full=protein eceriferum 4 |  |  |  |
| CX046529 | S22589490 | 4 | 17 | 3.653275 | 18.61344 | 2.349082325 | 0 | ucrcs07\_8f04\_b parent washington navel orange thrip-challenged flavedo cdna library ucrcs07 citrus sinensis cdna clone ucrcs07-8f04-k8-1- mrna |  |  |  |
| CX046605 | S22589528 | 27 | 125 | 24.6596 | 136.8635 | 2.472516267 | 0 | uncharacterized plant-specific domain tigr01615 family expressed |  |  |  |
| CX046913 | S22589674 | 32 | 85 | 29.2262 | 93.06719 | 1.67101042 | 0 | lea34\_goshilate embryogenesis abundant protein d-34 (lea d-34) |  |  |  |
| CX047329 | S22589866 | 7 | 15 | 6.393231 | 16.42362 | 1.361155158 | 0 | ucrcs09\_13e05\_b ruby orange developing seed cdna library ucrcs09 citrus sinensis cdna clone ucrcs09-13e05-i10-1- mrna |  |  |  |
| CX047858 | S22590108 | 3 | 21 | 2.739956 | 22.99307 | 3.068974406 | 0 | hypothetical protein Cagg\_1305 [Chloroflexus aggregans DSM 9485] |  |  |  |
| CX049732 | S22590978 | 53 | 158 | 48.40589 | 172.9955 | 1.837479778 | 0 | protein |  |  |  |
| CX049939 | S22591074 | 13 | 42 | 11.87314 | 45.98614 | 1.953497189 | 0 | predicted protein [Populus trichocarpa] |  |  |  |
| CX049951 | S22591079 | 4 | 27 | 3.653275 | 29.56252 | 3.016506986 | 0 | ucrcs09\_2e11\_b ruby orange developing seed cdna library ucrcs09 citrus sinensis cdna clone ucrcs09-2e11-i22-1- mrna |  |  |  |
| CX051758 | S22591917 | 23 | 1 | 21.00633 | 1.094908 | -4.261942472 | 0 | protein |  |  |  |
| CX052699 | S22592354 | 87 | 14 | 79.45873 | 15.32871 | -2.37396909 | 0 | probable flavin-containing monooxygenase 1 |  |  |  |
| CX054039 | S22592993 | 4 | 76 | 3.653275 | 83.21301 | 4.509546998 | 0 | dna binding protein |  |  |  |
| CX046840 | S22593052 | 23 | 56 | 21.00633 | 61.31485 | 1.54541245 | 0 | ucrcs09\_10e09\_b ruby orange developing seed cdna library ucrcs09 citrus sinensis cdna clone ucrcs09-10e09-j17-1- mrna |  |  |  |
| CX047037 | S22593159 | 4 | 3 | 3.653275 | 3.284724 | -0.153418015 | 0 | protein |  |  |  |
| CX047721 | S22593526 | 401 | 2479 | 366.2408 | 2714.277 | 2.889703614 | 0 | potassium channel tetramerization domain-containing |  |  |  |
| CX047752 | S22593544 | 3 | 11 | 2.739956 | 12.04399 | 2.136088602 | 0 | ucrcs09\_16c11\_b ruby orange developing seed cdna library ucrcs09 citrus sinensis cdna clone ucrcs09-16c11-e21-1- mrna |  |  |  |
| CX048061 | S22593707 | 9 | 1 | 8.219868 | 1.094908 | -2.908305517 | 0 | ucrcs09\_18e06\_b ruby orange developing seed cdna library ucrcs09 citrus sinensis cdna clone ucrcs09-18e06-j11-1- mrna |  |  |  |
| CX048596 | S22593994 | 8 | 27 | 7.306549 | 29.56252 | 2.016506986 | 0 | polyprotein [Lycopersicon esculentum] |  |  |  |
| CX048727 | S22594065 | 64 | 8 | 58.4524 | 8.759265 | -2.738380516 | 0 | ribosomal protein mitochondrial |  |  |  |
| CX048752 | S22594078 | 6 | 16 | 5.479912 | 17.51853 | 1.676656983 | 0 | hypothetical protein MtrDRAFT\_AC167711g44v2 [Medicago truncatula] |  |  |  |
| CX049428 | S22594442 | 41 | 560 | 37.44607 | 613.1485 | 4.033350496 | 0 | ucrcs09\_27e07\_b ruby orange developing seed cdna library ucrcs09 citrus sinensis cdna clone ucrcs09-27e07-j14-1- mrna |  |  |  |
| CX049594 | S22594531 | 14 | 1 | 12.78646 | 1.094908 | -3.545735438 | 0 | ucrcs09\_28e03\_b ruby orange developing seed cdna library ucrcs09 citrus sinensis cdna clone ucrcs09-28e03-i5-1- mrna |  |  |  |
| CX049781 | S22594633 | 291 | 32 | 265.7757 | 35.03706 | -2.923255859 | 0 | transcription factor |  |  |  |
| CX049911 | S22594703 | 2 | 10 | 1.826637 | 10.94908 | 2.583547579 | 0 | hypoxia induced protein conserved region containing expressed |  |  |  |
| CX050002 | S22594743 | 9 | 22 | 8.219868 | 24.08798 | 1.551126101 | 0 | nicotiana tabacum mitochondrial complete genome |  |  |  |
| CX050195 | S22594852 | 38 | 187 | 34.70611 | 204.7478 | 2.560586431 | 0 | protein |  |  |  |
| CX050314 | S22594917 | 27 | 56 | 24.6596 | 61.31485 | 1.314086904 | 0 | conserved hypothetical protein [Ricinus communis] |  |  |  |
| CX050961 | S22595264 | 6 | 16 | 5.479912 | 17.51853 | 1.676656983 | 0 | stress-inducible membrane pore protein |  |  |  |
| CX051193 | S22595387 | 31 | 69 | 28.31288 | 75.54866 | 1.41594763 | 0 | glycogenin-1 |  |  |  |
| CX051392 | S22595492 | 37 | 93 | 33.79279 | 101.8265 | 1.59132493 | 0 | peroxisomal membrane protein 11b ame: full=peroxin-11b� |  |  |  |
| CX051519 | S22595562 | 429 | 87 | 391.8137 | 95.257 | -2.040270858 | 0 | 3-ketoacyl- synthase 10� |  |  |  |
| CX052602 | S22596147 | 263 | 814 | 240.2028 | 891.2552 | 1.891585479 | 0 | �kda class i heat shock protein ame: full= kda heat shock protein� |  |  |  |
| CX053848 | S22596803 | 298 | 876 | 272.169 | 959.1395 | 1.817238023 | 0 | uncharacterized rna-binding protein |  |  |  |
| CX069489 | S22596967 | 14 | 40 | 12.78646 | 43.79632 | 1.776192657 | 0 | �water chloroplastic flags: precursor |  |  |  |
| CX070091 | S22597247 | 103 | 11 | 94.07182 | 12.04399 | -2.965449424 | 0 | snakin-2 flags: precursor |  |  |  |
| CX071402 | S22597854 | 53 | 158 | 48.40589 | 172.9955 | 1.837479778 | 0 | populus trichocarpa mrna |  |  |  |
| CX071677 | S22597982 | 13 | 3 | 11.87314 | 3.284724 | -1.853857733 | 0 | ucrcs08\_23c05\_b parent washington navel orange callus cdna library ucrcs08-2 citrus sinensis cdna clone ucrcs08-23c05-f9-1- mrna |  |  |  |
| CX072481 | S22598352 | 101 | 810 | 92.24519 | 886.8755 | 3.265186099 | 0 | ucrcs08\_28d01\_b parent washington navel orange callus cdna library ucrcs08-2 citrus sinensis cdna clone ucrcs08-28d01-h2-1- mrna |  |  |  |
| CX073729 | S22598931 | 76 | 21 | 69.41222 | 22.99307 | -1.593990607 | 0 | probable receptor protein kinase tmk1 flags: precursor |  |  |  |
| CX074287 | S22599188 | 89 | 398 | 81.28536 | 435.7734 | 2.422510674 | 0 | ring-h2 finger protein atl1m |  |  |  |
| CX074442 | S22599259 | 28 | 70 | 25.57292 | 76.64356 | 1.583547579 | 0 | protein |  |  |  |
| CX074820 | S22599436 | 88 | 456 | 80.37204 | 499.2781 | 2.63507788 | 0 | citrus ichangensis satellite dna |  |  |  |
| CX075508 | S22599753 | 2854 | 501 | 2606.612 | 548.5489 | -2.248483342 | 0 | ethylene-responsive transcription factor erf012 |  |  |  |
| CX077288 | S22600576 | 23 | 179 | 21.00633 | 195.9885 | 3.221873305 | 0 | bap2 (bon association protein 2) |  |  |  |
| CX077877 | S22600852 | 151 | 26 | 137.9111 | 28.46761 | -2.276345537 | 0 | u-box domain-containing protein 11 ame: full=plant u-box protein 11 |  |  |  |
| CX077928 | S22600876 | 10 | 36 | 9.133187 | 39.41669 | 2.109616391 | 0 | conserved hypothetical protein [Ricinus communis] |  |  |  |
| CX078410 | S22601108 | 9 | 28 | 8.219868 | 30.65743 | 1.899049405 | 0 | ucrcs08\_8h04\_g parent washington navel orange callus cdna library ucrcs08-2 citrus sinensis cdna clone ucrcs08-8h04-p8-1- mrna |  |  |  |
| CX070587 | S22601883 | 3 | 8 | 2.739956 | 8.759265 | 1.676656983 | 0 | ring-h2 finger protein atl4m ame: full=nep1-interacting protein 1 |  |  |  |
| CX070848 | S22602021 | 22 | 84 | 20.09301 | 91.97228 | 2.194505288 | 0 | probable glutamate carboxypeptidase 2 ame: full=probable glutamate carboxypeptidase ii |  |  |  |
| CX071680 | S22602466 | 10 | 1 | 9.133187 | 1.094908 | -3.060308611 | 0 | serine carboxypeptidase 24 ame: full=serine carboxypeptidase ii ame: full=carboxypeptidase d ame: full=bri1 suppressor 1 contains: ame: full=serine carboxypeptidase 24 chain a ame: full=serine carboxypeptidase ii chain a contains: ame: full=serine carboxypeptidase 24 chain b ame: full=serine carboxypeptidase ii chain b flags: precursor |  |  |  |
| CX072640 | S22602985 | 14 | 2 | 12.78646 | 2.189816 | -2.545735438 | 0 | ucrcs08\_29c12\_g parent washington navel orange callus cdna library ucrcs08-2 citrus sinensis cdna clone ucrcs08-29c12-e23-1- mrna |  |  |  |
| CX073187 | S22603277 | 48 | 114 | 43.8393 | 124.8195 | 1.509546998 | 0 | guanine nucleotide-binding protein alpha-1 subunit� |  |  |  |
| CX073773 | S22603590 | 267 | 1067 | 243.8561 | 1168.267 | 2.260268013 | 0 | probable protein phosphatase 2c 2� |  |  |  |
| CX074112 | S22603775 | 26 | 6 | 23.74629 | 6.569448 | -1.853857733 | 0 | receptor protein kinase clavata1 flags: precursor |  |  |  |
| CX074583 | S22604029 | 1 | 10 | 0.913319 | 10.94908 | 3.583547579 | 0 | legumin type b contains: ame: full=legumin type b alpha chain ame: full=legumin type b acidic chain contains: ame: full=legumin type b beta chain ame: full=legumin type b basic chain flags: precursor |  |  |  |
| CX074986 | S22604245 | 234 | 51 | 213.7166 | 55.84031 | -1.936319894 | 0 | chloroplast-targeted copper |  |  |  |
| CX076036 | S22604809 | 42 | 336 | 38.35938 | 367.8891 | 3.261619484 | 0 | dehydration-responsive element-binding protein 1c� |  |  |  |
| CX076101 | S22604844 | 8 | 290 | 7.306549 | 317.5233 | 5.441528574 | 0 | embryogenic cell protein 40� |  |  |  |
| CX076324 | S22604965 | 17 | 3 | 15.52642 | 3.284724 | -2.240880856 | 0 | coleoptile phototropism protein 1 ame: full=non-phototropic hypocotyl 3-like protein� |  |  |  |
| CX076597 | S22605110 | 172 | 32 | 157.0908 | 35.03706 | -2.164645271 | 0 | protein |  |  |  |
| CX077871 | S22605795 | 8 | 18 | 7.306549 | 19.70835 | 1.431544486 | 0 | hydroxyproline-rich glycoprotein family protein |  |  |  |
| CX077884 | S22605796 | 745 | 174 | 680.4224 | 190.514 | -1.836533635 | 0 | receptor-like protein kinase hsl1 ame: full=protein haesa-like1 flags: precursor |  |  |  |
| AF321533 | S22606192 | 828 | 3742 | 756.2279 | 4097.146 | 2.43772637 | 0 | 1-aminocyclopropane-1-carboxylate oxidase� |  |  |  |
| AF000135 | S22606213 | 14 | 4 | 12.78646 | 4.379632 | -1.545735438 | 0 | endoglucanase 1 ame: full=endo- -beta-glucanase 1 ame: full=abscission cellulase 1 flags: precursor |  |  |  |
| U82976 | S22606218 | 19993 | 4131 | 18259.98 | 4523.065 | -2.013312519 | 0 | pectinesterase 1� |  |  |  |
| CX675213 | S23016713 | 9 | 0 | 8.219868 | 0 | -Inf | 0 | ring-h2 finger protein atl1l |  |  |  |
| CX675240 | S23016726 | 11 | 0 | 10.04651 | 0 | -Inf | 0 | ucrcs08\_60g07\_b parent washington navel orange callus cdna library ucrcs08-3 citrus sinensis cdna clone ucrcs08-60g07-m14-1- mrna |  |  |  |
| CX675291 | S23016749 | 9 | 19 | 8.219868 | 20.80325 | 1.339621996 | 0 | 11-beta-hydroxysteroid dehydrogenase-like |  |  |  |
| CX675523 | S23016855 | 2721 | 470 | 2485.14 | 514.6068 | -2.271784811 | 0 | protein |  |  |  |
| CX675813 | S23016991 | 47 | 5 | 42.92598 | 5.47454 | -2.971041273 | 0 | btb poz domain-containing protein at5g48800 |  |  |  |
| CX676293 | S23017212 | 11 | 28 | 10.04651 | 30.65743 | 1.609542787 | 0 | ucrcs08\_68e11\_b parent washington navel orange callus cdna library ucrcs08-3 citrus sinensis cdna clone ucrcs08-68e11-i22-1- mrna |  |  |  |
| CX676294 | S23017213 | 2 | 11 | 1.826637 | 12.04399 | 2.721051103 | 0 | ucrcs08\_68e11\_g parent washington navel orange callus cdna library ucrcs08-3 citrus sinensis cdna clone ucrcs08-68e11-i22-1- mrna |  |  |  |
| CX675374 | S23017571 | 7 | 18 | 6.393231 | 19.70835 | 1.624189563 | 0 | citrus unshiu 5 flanking region of d-limonene synthase |  |  |  |
| CX675554 | S23017668 | 37 | 98 | 33.79279 | 107.301 | 1.666875963 | 0 | ras-gtpase-activating protein-binding |  |  |  |
| CX675713 | S23017754 | 5 | 14 | 4.566593 | 15.32871 | 1.747046311 | 0 | vitis vinifera contig whole genome shotgun sequence |  |  |  |
| CX676092 | S23017959 | 4 | 13 | 3.653275 | 14.2338 | 1.962059202 | 0 | ucrcs08\_67a08\_b parent washington navel orange callus cdna library ucrcs08-3 citrus sinensis cdna clone ucrcs08-67a08-a15-1- mrna |  |  |  |
| CX676116 | S23017971 | 22 | 104 | 20.09301 | 113.8704 | 2.502627584 | 0 | 1-aminocyclopropane-1-carboxylate oxidase 1� |  |  |  |
| CX676339 | S23018092 | 10 | 2 | 9.133187 | 2.189816 | -2.060308611 | 0 | ucrcs08\_68h04\_b parent washington navel orange callus cdna library ucrcs08-3 citrus sinensis cdna clone ucrcs08-68h04-o8-1- mrna |  |  |  |
| CX671518 | S23018337 | 10 | 0 | 9.133187 | 0 | -Inf | 0 | protein |  |  |  |
| CX671600 | S23018380 | 11 | 2 | 10.04651 | 2.189816 | -2.197812135 | 0 | ucrcs10\_12a11\_b madame vinous sweet orange multiple pathogen-infected cdna library ucrcs10 citrus sinensis cdna clone ucrcs10-12a11-b22- mrna |  |  |  |
| CX671705 | S23018431 | 8 | 24 | 7.306549 | 26.27779 | 1.846581985 | 0 | ucrcs10\_12f08\_b madame vinous sweet orange multiple pathogen-infected cdna library ucrcs10 citrus sinensis cdna clone ucrcs10-12f08-l16- mrna |  |  |  |
| CX671835 | S23018494 | 23 | 59 | 21.00633 | 64.59958 | 1.620700577 | 0 | populus trichocarpa mrna |  |  |  |
| CX672039 | S23018591 | 84 | 4 | 76.71877 | 4.379632 | -4.130697939 | 0 | subtilisin-like protease ame: full=cucumisin-like serine protease flags: precursor |  |  |  |
| CX672325 | S23018731 | 174 | 47 | 158.9175 | 51.46068 | -1.62673516 | 0 | zinc transporter 1 ame: full=zrt irt-like protein 1 flags: precursor |  |  |  |
| CX672980 | S23019054 | 11 | 4 | 10.04651 | 4.379632 | -1.197812135 | 0 | ucrcs10\_1f09\_g madame vinous sweet orange multiple pathogen-infected cdna library ucrcs10 citrus sinensis cdna clone ucrcs10-1f09-k17- mrna |  |  |  |
| CX674161 | S23019647 | 9 | 0 | 8.219868 | 0 | -Inf | 0 | cral trio domain containing expressed |  |  |  |
| CX674613 | S23019889 | 61 | 16 | 55.71244 | 17.51853 | -1.669117853 | 0 | protein |  |  |  |
| CX674977 | S23020085 | 317 | 42 | 289.522 | 45.98614 | -2.654402123 | 0 | oligopeptide transporter 1� |  |  |  |
| CX671291 | S23020112 | 480 | 83 | 438.393 | 90.87737 | -2.27023168 | 0 | receptor-like protein kinase hsl1 ame: full=protein haesa-like1 flags: precursor |  |  |  |
| CX671357 | S23020146 | 186 | 15 | 169.8773 | 16.42362 | -3.370648731 | 0 | l-ascorbate oxidase homolog flags: precursor |  |  |  |
| CX671853 | S23020402 | 9 | 29 | 8.219868 | 31.75233 | 1.949675478 | 0 | ucrcs10\_13e07\_g madame vinous sweet orange multiple pathogen-infected cdna library ucrcs10 citrus sinensis cdna clone ucrcs10-13e07-i13- mrna |  |  |  |
| CX673271 | S23021048 | 52 | 134 | 47.49257 | 146.7177 | 1.627268956 | 0 | probable lrr receptor-like serine threonine-protein kinase at1g56140 flags: precursor |  |  |  |
| CX673376 | S23021107 | 1154 | 142 | 1053.97 | 155.4769 | -2.761060905 | 0 | ring-h2 finger protein atl2n |  |  |  |
| CX673883 | S23021551 | 0 | 11 | 0 | 12.04399 | Inf | 0 | thromboxane-a synthase� |  |  |  |
| CX674127 | S23021662 | 35 | 84 | 31.96615 | 91.97228 | 1.52465389 | 0 | protein |  |  |  |
| CX674272 | S23021730 | 111 | 256 | 101.3784 | 280.2965 | 1.467203618 | 0 | ucrcs10\_5d08\_b madame vinous sweet orange multiple pathogen-infected cdna library ucrcs10 citrus sinensis cdna clone ucrcs10-5d08-g15- mrna |  |  |  |
| DN134817 | S23749941 | 2 | 6 | 1.826637 | 6.569448 | 1.846581985 | 0 | methionine s-methyltransferase ame: full= et:met s-methyltransferase |  |  |  |
| DN135042 | S23750027 | 6 | 33 | 5.479912 | 36.13197 | 2.721051103 | 0 | proteinase inhibitor ame: full=luti |  |  |  |
| DN618700 | S24239635 | 20 | 63 | 18.26637 | 68.97921 | 1.916971313 | 0 | glycine max strain williams 82 clone complete sequence |  |  |  |
| DN620718 | S24240789 | 83 | 241 | 75.80545 | 263.8728 | 1.799469389 | 0 | serine threonine-protein kinase oxi1 ame: full=protein oxidative signal-inducible 1 |  |  |  |
| DN621518 | S24241247 | 40 | 91 | 36.53275 | 99.63663 | 1.447486029 | 0 | ucrcs11\_06n05\_f parent washington navel orange scale-infested rind cdna library ucrcs11 citrus sinensis cdna clone mrna |  |  |  |
| DN617952 | S24241609 | 64 | 148 | 58.4524 | 162.0464 | 1.47107285 | 0 | thioredoxin-like chloroplastic flags: precursor |  |  |  |
| DN619200 | S24242145 | 24 | 150 | 21.91965 | 164.2362 | 2.905475674 | 0 | cytochrome p450 94a2 ame: full=p450-dependent fatty acid omega-hydroxylase |  |  |  |
| DN620794 | S24242827 | 43 | 301 | 39.2727 | 329.5673 | 3.068974406 | 0 | f-box family protein |  |  |  |
| CX300836 | S24634758 | 2 | 19 | 1.826637 | 20.80325 | 3.509546998 | 0 | c08002g06sk ootsw1 citrus sinensis cdna clone mrna |  |  |  |
| CX301048 | S24634819 | 4 | 70 | 3.653275 | 76.64356 | 4.390902501 | 0 | u-box domain-containing protein 21 ame: full=plant u-box protein 21 |  |  |  |
| CX301592 | S24634997 | 14 | 0 | 12.78646 | 0 | -Inf | 0 | probable elongation factor 1-gamma 2� |  |  |  |
| CX301818 | S24635071 | 19 | 150 | 17.35306 | 164.2362 | 3.242510661 | 0 | elongation factor 1-alpha� |  |  |  |
| CX302396 | S24635390 | 1 | 10 | 0.913319 | 10.94908 | 3.583547579 | 0 | c08020c09sk ootsw1 citrus sinensis cdna clone mrna |  |  |  |
| CX302838 | S24635647 | 37 | 14 | 33.79279 | 15.32871 | -1.140478959 | 0 | citrus sinensis dna binding protein (v03-2) complete cds |  |  |  |
| CX302890 | S24635897 | 45 | 112 | 41.09934 | 122.6297 | 1.57712131 | 0 | c08025c10sk ootsw1 citrus sinensis cdna clone mrna |  |  |  |
| CX302939 | S24635922 | 759 | 1714 | 693.2089 | 1876.672 | 1.436814803 | 0 | c08025g11sk ootsw1 citrus sinensis cdna clone mrna |  |  |  |
| CX303152 | S24636024 | 108 | 102 | 98.63842 | 111.6806 | 0.179157324 | 0 | wound-induced protein win1 flags: precursor |  |  |  |
| CX303089 | S24636190 | 35 | 3 | 31.96615 | 3.284724 | -3.282701032 | 0 | c08027d11sk ootsw1 citrus sinensis cdna clone mrna |  |  |  |
| CX303285 | S24636282 | 21 | 0 | 19.17969 | 0 | -Inf | 0 | c08029e10sk ootsw1 citrus sinensis cdna clone mrna |  |  |  |
| DR403432 | S25678430 | 0 | 14 | 0 | 15.32871 | Inf | 0 | csac-pnp1241a05 mature albedo citrus sinensis cdna clone csac-pnp1241a05 5 mrna |  |  |  |
| DR403529 | S25678527 | 14 | 46 | 12.78646 | 50.36577 | 1.977826518 | 0 | arabidopsis thaliana at3g62990 complete cds |  |  |  |
| DR403972 | S25678970 | 6 | 10 | 5.479912 | 10.94908 | 0.998585078 | 0 | csah-pnp1246n23 developing fruit peel at 38 dafb citrus sinensis cdna clone csah-pnp1246n23 5 mrna |  |  |  |
| DR403990 | S25678988 | 4 | 16 | 3.653275 | 17.51853 | 2.261619484 | 0 | csah-pnp1246o19 developing fruit peel at 38 dafb citrus sinensis cdna clone csah-pnp1246o19 5 mrna |  |  |  |
| DR404142 | S25679140 | 84 | 226 | 76.71877 | 247.4492 | 1.689481024 | 0 | csag-pnp1245g11 developing fruit juice sac at 38 dafb citrus sinensis cdna clone csag-pnp1245g11 5 mrna |  |  |  |
| DR404508 | S25679506 | 407 | 94 | 371.7207 | 102.9214 | -1.852676649 | 0 | protein |  |  |  |
| DR405064 | S25680062 | 4227 | 4440 | 3860.598 | 4861.392 | 0.332545048 | 0 | csab-pnp1240e07 developing fruit albedo at 165 dafb citrus sinensis cdna clone csab-pnp1240e07 5 mrna |  |  |  |
| DR405282 | S25680280 | 13 | 49 | 11.87314 | 53.6505 | 2.17588961 | 0 | csab-pnp1240p15 developing fruit albedo at 165 dafb citrus sinensis cdna clone csab-pnp1240p15 5 mrna |  |  |  |
| DR405291 | S25680289 | 3 | 25 | 2.739956 | 27.3727 | 3.320513173 | 0 | csad-pnp1242a02 developing fruit flavedo at 165 dafb citrus sinensis cdna clone csad-pnp1242a02 5 mrna |  |  |  |
| DR405437 | S25680435 | 7 | 17 | 6.393231 | 18.61344 | 1.541727403 | 0 | csad-pnp1242h08 developing fruit flavedo at 165 dafb citrus sinensis cdna clone csad-pnp1242h08 5 mrna |  |  |  |
| DR405580 | S25680578 | 0 | 16 | 0 | 17.51853 | Inf | 0 | csad-pnp1242o09 developing fruit flavedo at 165 dafb citrus sinensis cdna clone csad-pnp1242o09 5 mrna |  |  |  |
| DR405840 | S25680838 | 272 | 66 | 248.4227 | 72.26393 | -1.781449238 | 0 | protein |  |  |  |
| DR406097 | S25681095 | 81 | 181 | 73.97881 | 198.1784 | 1.421615368 | 0 | csaj-pnp1248l10 mature juice sac from navel orange in p x2 vector citrus sinensis cdna clone csaj-pnp1248l10 5 mrna |  |  |  |
| DR406100 | S25681098 | 491 | 1410 | 448.4395 | 1543.82 | 1.783519717 | 0 | cysteine proteinase rd21a� |  |  |  |
| DR406125 | S25681123 | 2 | 7 | 1.826637 | 7.664356 | 2.068974406 | 0 | protein |  |  |  |
| DR908518 | S26279395 | 60 | 437 | 54.79912 | 478.4748 | 3.126218358 | 0 | cytochrome p450 76c4 |  |  |  |
| DR909180 | S26280057 | 8 | 19 | 7.306549 | 20.80325 | 1.509546998 | 0 | usda-fp\_17308 citrus sinensis phloem citrus sinensis cdna clone vpe-35\_g11 5 mrna |  |  |  |
| DR909350 | S26280227 | 276 | 45 | 252.076 | 49.27086 | -2.355051876 | 0 | probable pectinesterase pectinesterase inhibitor 34 includes: ame: full=pectinesterase inhibitor 34 ame: full=pectin methylesterase inhibitor 34 includes: ame: full=pectinesterase 34� |  |  |  |
| DR909433 | S26280310 | 53 | 9 | 48.40589 | 9.854173 | -2.296375969 | 0 | populus trichocarpa mrna |  |  |  |
| DR909527 | S26280404 | 5 | 16 | 4.566593 | 17.51853 | 1.939691389 | 0 | usda-fp\_17655 citrus sinensis phloem citrus sinensis cdna clone vpe-10\_b01 5 mrna |  |  |  |
| DR909871 | S26280748 | 127 | 286 | 115.9915 | 313.1437 | 1.432806134 | 0 | uncharacterized protein at3g06530 |  |  |  |
| DR909987 | S26280864 | 456 | 57 | 416.4733 | 62.40976 | -2.738380516 | 0 | proline-rich glycoprotein |  |  |  |
| DR910089 | S26280966 | 13 | 27 | 11.87314 | 29.56252 | 1.316067268 | 0 | usda-fp\_18217 citrus sinensis phloem citrus sinensis cdna clone vpe-21\_f01 5 mrna |  |  |  |
| DR910216 | S26281093 | 8 | 1 | 7.306549 | 1.094908 | -2.738380516 | 0 | auxin transporter-like protein 3 ame: full=aux1-like protein 3 |  |  |  |
| DR910631 | S26281508 | 39 | 1 | 35.61943 | 1.094908 | -5.023782735 | 0 | plasma membrane associated protein |  |  |  |
| DR910681 | S26281558 | 17 | 38 | 15.52642 | 41.60651 | 1.422084156 | 0 | usda-fp\_18809 citrus sinensis phloem citrus sinensis cdna clone vpe-29\_c08 5 mrna |  |  |  |
| DR910720 | S26281597 | 5 | 18 | 4.566593 | 19.70835 | 2.109616391 | 0 | tgacg-sequence-specific dna-binding protein tga-� |  |  |  |
| DR910861 | S26281738 | 5 | 31 | 4.566593 | 33.94215 | 2.8938877 | 0 | usda-fp\_18989 citrus sinensis phloem citrus sinensis cdna clone vpe-14\_f02 5 mrna |  |  |  |
| DR910970 | S26281847 | 7 | 26 | 6.393231 | 28.46761 | 2.15470428 | 0 | usda-fp\_19098 citrus sinensis phloem citrus sinensis cdna clone vpe-36\_e07 5 mrna |  |  |  |
| DR911071 | S26281948 | 81 | 23 | 73.97881 | 25.18289 | -1.554668563 | 0 | usda-fp\_19199 citrus sinensis phloem citrus sinensis cdna clone vpe-20\_f08 5 mrna |  |  |  |
| DR911129 | S26282006 | 5 | 1 | 4.566593 | 1.094908 | -2.060308611 | 0 | populus trichocarpa mrna |  |  |  |
| DR911175 | S26282052 | 38 | 132 | 34.70611 | 144.5279 | 2.05808609 | 0 | usda-fp\_19303 citrus sinensis phloem citrus sinensis cdna clone vpe-45\_d02 5 mrna |  |  |  |
| DR911190 | S26282067 | 16 | 4 | 14.6131 | 4.379632 | -1.738380516 | 0 | usda-fp\_19318 citrus sinensis phloem citrus sinensis cdna clone vpe-19\_c12 5 mrna |  |  |  |
| DR911252 | S26282129 | 7 | 24 | 6.393231 | 26.27779 | 2.039227063 | 0 | usda-fp\_19380 citrus sinensis phloem citrus sinensis cdna clone vpe-49\_a01 5 mrna |  |  |  |
| DR911519 | S26282396 | 153 | 537 | 139.7378 | 587.9656 | 2.073009919 | 0 | usda-fp\_19647 citrus sinensis phloem citrus sinensis cdna clone vpe-42\_h04 5 mrna |  |  |  |
| DR911733 | S26282610 | 58 | 128 | 52.97248 | 140.1482 | 1.403638489 | 0 | usda-fp\_19861 citrus sinensis phloem citrus sinensis cdna clone vpe-43\_f07 5 mrna |  |  |  |
| DR912053 | S26282930 | 6 | 39 | 5.479912 | 42.70141 | 2.962059202 | 0 | usda-fp\_20181 citrus sinensis phloem citrus sinensis cdna clone vpe-09\_a04 5 mrna |  |  |  |
| DR912150 | S26283027 | 222 | 308 | 202.7567 | 337.2317 | 0.733990158 | 0 | usda-fp\_20279 citrus sinensis phloem citrus sinensis cdna clone vpe-10\_e10 5 mrna |  |  |  |
| DT214573 | S26468962 | 44 | 138 | 40.18602 | 151.0973 | 1.910712322 | 0 | aquaporin nip6-1 ame: full=nod26-like intrinsic protein 6-1� |  |  |  |
| DQ028471 | S32321255 | 75 | 517 | 68.4989 | 566.0675 | 3.046821264 | 0 | 9-cis-epoxycarotenoid dioxygenase chloroplastic ame: full= 1 flags: precursor |  |  |  |
| DY257218 | S34124496 | 24492 | 5458 | 22369 | 5976.008 | -1.904246804 | 0 | protein |  |  |  |
| DY257259 | S34124537 | 818 | 2735 | 747.0947 | 2994.574 | 2.002987569 | 0 | kn0aak1da01fm2 ruit citrus sinensis cdna 5 mrna |  |  |  |
| DY257267 | S34124545 | 226 | 38 | 206.41 | 41.60651 | -2.310631965 | 0 | nucleobase-ascorbate transporter 2� |  |  |  |
| DY257306 | S34124584 | 45 | 240 | 41.09934 | 262.7779 | 2.676656983 | 0 | u-box domain-containing protein 19 ame: full=plant u-box protein 19 |  |  |  |
| DY257343 | S34124621 | 4085 | 756 | 3730.907 | 827.7505 | -2.172258455 | 0 | expansin-like a2� |  |  |  |
| DY257446 | S34124724 | 785 | 191 | 716.9552 | 209.1274 | -1.777500532 | 0 | aquaporin tip2-1 ame: full=tonoplast intrinsic protein 2-1� |  |  |  |
| DY257559 | S34124837 | 6 | 24 | 5.479912 | 26.27779 | 2.261619484 | 0 | protein |  |  |  |
| DY257659 | S34124937 | 411 | 105 | 375.374 | 114.9653 | -1.707129582 | 0 | citrus unshiu mrna for metallothionein-like complete cds |  |  |  |
| DY305480 | S34125042 | 75 | 177 | 68.4989 | 193.7987 | 1.500406344 | 0 | probable lrr receptor-like serine threonine-protein kinase at2g16250 flags: precursor |  |  |  |
| DY305488 | S34125050 | 63 | 194 | 57.53908 | 212.4122 | 1.884252403 | 0 | transcription factor bhlh35 ame: full=transcription factor en 41 ame: full=bhlh transcription factor bhlh035 ame: full=basic helix-loop-helix protein 35� |  |  |  |
| DY305490 | S34125052 | 10930 | 24318 | 9982.573 | 26625.97 | 1.415350664 | 0 | indole-3-acetic acid-induced protein arg2 |  |  |  |
| DY305530 | S34125092 | 14 | 61 | 12.78646 | 66.78939 | 2.3850019 | 0 | chlorophyllase- chloroplastic ame: full=chlorophyll-chlorophyllido hydrolase 1� |  |  |  |
| DY305571 | S34125133 | 491 | 1188 | 448.4395 | 1300.751 | 1.536359391 | 0 | mlo-like protein 6� |  |  |  |
| DY305580 | S34125142 | 2397 | 11455 | 2189.225 | 12542.17 | 2.518295129 | 0 | zinc finger protein 1 ame: full=wzf1 |  |  |  |
| DY305607 | S34125169 | 627 | 1870 | 572.6508 | 2047.478 | 1.838120406 | 0 | protein |  |  |  |
| DY305613 | S34125175 | 47 | 186 | 42.92598 | 203.6529 | 2.246189444 | 0 | transcription factor bhlh36 ame: full=transcription factor en 6 ame: full=bhlh transcription factor bhlh036 ame: full=basic helix-loop-helix protein 36� |  |  |  |
| DY305627 | S34125189 | 18 | 2 | 16.43974 | 2.189816 | -2.908305517 | 0 | kn0aam1cg10rm1 slh citrus sinensis cdna 5 mrna |  |  |  |
| DY305655 | S34125217 | 1344 | 120 | 1227.5 | 131.389 | -3.223807343 | 0 | aspartic proteinase nepenthesin-2 ame: full=nepenthesin-ii flags: precursor |  |  |  |
| DY305681 | S34125243 | 137 | 1201 | 125.1247 | 1314.985 | 3.393607837 | 0 | ethylene-responsive transcription factor erf017 |  |  |  |
| DY305690 | S34125252 | 378 | 1021 | 345.2345 | 1117.901 | 1.695144211 | 0 | probable glutathione s-transferase ame: full=auxin-induced protein pgnt35 pcnt111 |  |  |  |
| DY305694 | S34125256 | 330 | 1247 | 301.3952 | 1365.35 | 2.17954302 | 0 | potassium channel tetramerization domain-containing |  |  |  |
| DY305725 | S34125287 | 159 | 812 | 145.2177 | 889.0653 | 2.614072446 | 0 | tropinone reductase homolog at1g07440 |  |  |  |
| DY305732 | S34125294 | 1421 | 3766 | 1297.826 | 4123.424 | 1.667745929 | 0 | ring-h2 finger protein atl3f ame: full=ring-h2 finger protein atl2 |  |  |  |
| DY305734 | S34125296 | 432 | 1070 | 394.5537 | 1171.552 | 1.570127063 | 0 | sucrose transport protein suc1 ame: full=sucrose permease 1 ame: full=sucrose-proton symporter 1 |  |  |  |
| DY305736 | S34125298 | 147 | 486 | 134.2578 | 532.1253 | 1.986759643 | 0 | cytochrome p450 71b35 |  |  |  |
| DY305779 | S34125341 | 149 | 28 | 136.0845 | 30.65743 | -2.150194114 | 0 | alpha galactosidase precursor |  |  |  |
| DY305817 | S34125379 | 528 | 1697 | 482.2323 | 1858.059 | 1.945996214 | 0 | glycosyltransferase 8 domain-containing protein 1 |  |  |  |
| DY305828 | S34125390 | 11 | 0 | 10.04651 | 0 | -Inf | 0 | phosphoglucomutase phosphomannomutase family protein |  |  |  |
| DY305861 | S34125423 | 5 | 39 | 4.566593 | 42.70141 | 3.225093608 | 0 | protein |  |  |  |
| DY305898 | S34125460 | 273 | 1075 | 249.336 | 1177.026 | 2.238983288 | 0 | calcium-binding protein cml37 ame: full=calmodulin-like protein 37 |  |  |  |
| DY305904 | S34125466 | 4617 | 346 | 4216.792 | 378.8382 | -3.476492305 | 0 | 21 kda protein ame: full= protein flags: precursor |  |  |  |
| DY305912 | S34125474 | 32 | 194 | 29.2262 | 212.4122 | 2.861532326 | 0 | polyneuridine-aldehyde esterase ame: full=polyneuridine aldehyde esterase flags: precursor |  |  |  |
| DY305919 | S34125481 | 1508 | 129 | 1377.285 | 141.2431 | -3.285573974 | 0 | PREDICTED: hypothetical protein [Vitis vinifera] |  |  |  |
| DY305927 | S34125489 | 202 | 447 | 184.4904 | 489.4239 | 1.407539023 | 0 | protein brittle- chloroplastic amyloplastic flags: precursor |  |  |  |
| DY305970 | S34125532 | 14 | 35 | 12.78646 | 38.32178 | 1.583547579 | 0 | kn0aam3ad01rm1 slh citrus sinensis cdna 5 mrna |  |  |  |
| DY305997 | S34125559 | 486 | 90 | 443.8729 | 98.54173 | -2.171339923 | 0 | probable metal-nicotianamine transporter ysl5 ame: full=protein yellow stripe like 5� |  |  |  |
| DY306001 | S34125563 | 428 | 4109 | 390.9004 | 4498.977 | 3.524724113 | 0 | nac domain-containing protein 72� |  |  |  |
| DY306009 | S34125571 | 1519 | 149 | 1387.331 | 163.1413 | -3.08811815 | 0 | lob domain-containing protein 40 ame: full=asymmetric leaves 2-like protein 37� |  |  |  |
| DY306027 | S34125589 | 15 | 6 | 13.69978 | 6.569448 | -1.060308611 | 0 | expansin-like b1 ame: full= 1 ame: full=expensin-related 1 ame: full= 1 ame: full= flags: precursor |  |  |  |
| DY306086 | S34125648 | 50 | 129 | 45.66593 | 141.2431 | 1.62899055 | 0 | receptor serine threonine |  |  |  |
| DY306100 | S34125662 | 1635 | 3768 | 1493.276 | 4125.614 | 1.466127813 | 0 | myb-like protein j |  |  |  |
| DY306136 | S34125698 | 0 | 154 | 0 | 168.6158 | Inf | 0 | jasmonate o-methyltransferase ame: full=s-adenosyl-l-methionine:jasmonic acid carboxyl methyltransferase ame: full=floral nectary-specific protein 1 |  |  |  |
| DY306171 | S34125733 | 141 | 325 | 128.7779 | 355.8451 | 1.46636404 | 0 | anthranilate n-benzoyltransferase protein 2 ame: full=anthranilate n-hydroxycinnamoyl benzoyltransferase 2 |  |  |  |
| DY306181 | S34125743 | 460 | 1794 | 420.1266 | 1964.265 | 2.225093608 | 0 | protein |  |  |  |
| DY306194 | S34125756 | 283 | 653 | 258.4692 | 714.975 | 1.467900423 | 0 | purine permease 1� |  |  |  |
| DY306872 | S34125767 | 5 | 36 | 4.566593 | 39.41669 | 3.109616391 | 0 | transcription factor bhlh36 ame: full=transcription factor en 6 ame: full=bhlh transcription factor bhlh036 ame: full=basic helix-loop-helix protein 36� |  |  |  |
| AB276108 | S35152777 | 16 | 71 | 14.6131 | 77.73847 | 2.411366604 | 0 | beta- soluble isoenzyme i ame: full=sucrose hydrolase ame: full=invertase ame: full=saccharase flags: precursor |  |  |  |
| EG358279 | S35174889 | 473 | 2723 | 431.9997 | 2981.435 | 2.786904378 | 0 | blue copper protein flags: precursor |  |  |  |
| EG358327 | S35174937 | 5 | 6 | 4.566593 | 6.569448 | 0.52465389 | 0 | pectinesterase 2� |  |  |  |
| EG358332 | S35174942 | 3 | 22 | 2.739956 | 24.08798 | 3.136088602 | 0 | serine-threonine protein plant- |  |  |  |
| EG358340 | S35174950 | 11 | 31 | 10.04651 | 33.94215 | 1.756384176 | 0 | cytochrome b5 |  |  |  |
| EF185419 | S36619343 | 976 | 2701 | 891.399 | 2957.347 | 1.730160071 | 0 | nac domain-containing protein 29� |  |  |  |
| EU200366 | S41488901 | 30 | 261 | 27.39956 | 285.771 | 3.382634885 | 0 | taxadien-5-alpha-ol o-acetyltransferase ame: full=taxa-4 -dien-5alpha-ol-o-acetyltransferase� |  |  |  |
| EU240878 | S41505081 | 4051 | 990 | 3699.854 | 1083.959 | -1.771158171 | 0 | transcription factor bhlh137 ame: full=transcription factor en 89 ame: full=bhlh transcription factor bhlh137 ame: full=basic helix-loop-helix protein 137� |  |  |  |
| EY649677 | S44206839 | 16 | 38 | 14.6131 | 41.60651 | 1.509546998 | 0 | cs00-c1-100-002-c07- sweet orange greenhouse plant citrus sinensis mrna |  |  |  |
| EY649813 | S44206975 | 4 | 13 | 3.653275 | 14.2338 | 1.962059202 | 0 | at1g65900 f12p19\_7 |  |  |  |
| EY650326 | S44207376 | 233 | 678 | 212.8033 | 742.3477 | 1.802574803 | 0 | �uncharacterized protein ycf15 |  |  |  |
| EY650468 | S44207518 | 71 | 167 | 64.84563 | 182.8496 | 1.495576657 | 0 | cs00-c1-100-011-d03- sweet orange greenhouse plant citrus sinensis mrna |  |  |  |
| EY650515 | S44207565 | 179 | 437 | 163.484 | 478.4748 | 1.549293176 | 0 | cytochrome p450 |  |  |  |
| EY650552 | S44207602 | 1114 | 59 | 1017.437 | 64.59958 | -3.977270984 | 0 | subtilisin-like protease ame: full=cucumisin-like serine protease flags: precursor |  |  |  |
| EY650692 | S44207742 | 247 | 65 | 225.5897 | 71.16902 | -1.664379934 | 0 | cytochrome p450 83b1 |  |  |  |
| EY651373 | S44208087 | 897 | 2968 | 819.2469 | 3249.687 | 1.987930686 | 0 | protein |  |  |  |
| EY651736 | S44208450 | 3 | 14 | 2.739956 | 15.32871 | 2.484011905 | 0 | cs00-c1-100-025-f01- sweet orange greenhouse plant citrus sinensis mrna |  |  |  |
| EY651762 | S44208476 | 441 | 1001 | 402.7735 | 1096.003 | 1.444210897 | 0 | branched-chain-amino-acid aminotransferase chloroplastic� |  |  |  |
| EY652427 | S44208819 | 12 | 28 | 10.95982 | 30.65743 | 1.484011905 | 0 | protein |  |  |  |
| EY652914 | S44209306 | 11 | 31 | 10.04651 | 33.94215 | 1.756384176 | 0 | protein p21 |  |  |  |
| EY652929 | S44209321 | 22 | 5 | 20.09301 | 5.47454 | -1.87588404 | 0 | probable esterase at1g33990 |  |  |  |
| EY652975 | S44209367 | 120 | 47 | 109.5982 | 51.46068 | -1.09068226 | 0 | protein iq-domain 1 |  |  |  |
| EY653765 | S44209765 | 23 | 6 | 21.00633 | 6.569448 | -1.676979971 | 0 | acrosin contains: ame: full=acrosin light chain contains: ame: full=acrosin heavy chain flags: precursor |  |  |  |
| EY653870 | S44209870 | 21 | 62 | 19.17969 | 67.8843 | 1.823498372 | 0 | populus trichocarpa mrna |  |  |  |
| EY654098 | S44210014 | 21 | 51 | 19.17969 | 55.84031 | 1.541727403 | 0 | vitis vinifera contig whole genome shotgun sequence |  |  |  |
| EY654505 | S44210421 | 1006 | 229 | 918.7986 | 250.7339 | -1.873591318 | 0 | calmodulin-like protein 3 |  |  |  |
| EY654618 | S44210534 | 3557 | 707 | 3248.675 | 774.1 | -2.06925937 | 0 | chlorophyll a-b binding protein chloroplastic ame: full=lhcii type ii cab-151� |  |  |  |
| EY654816 | S44210732 | 1192 | 298 | 1088.676 | 326.2826 | -1.738380516 | 0 | (+)-delta-cadinene synthase isozyme xc1� |  |  |  |
| EY654823 | S44210739 | 229 | 59 | 209.15 | 64.59958 | -1.694941255 | 0 | cs00-c1-100-063-h01- sweet orange greenhouse plant citrus sinensis mrna |  |  |  |
| EY654875 | S44210791 | 84 | 20 | 76.71877 | 21.89816 | -1.808769844 | 0 | probable polygalacturonase� |  |  |  |
| EY655352 | S44211058 | 15 | 4 | 13.69978 | 4.379632 | -1.645271112 | 0 | zea mays clone mrna sequence |  |  |  |
| EY655547 | S44211253 | 8 | 23 | 7.306549 | 25.18289 | 1.78518144 | 0 | ankyrin repeat-containing protein at2g01680 |  |  |  |
| EY655795 | S44211417 | 126 | 32 | 115.0782 | 35.03706 | -1.715660439 | 0 | unnamed protein product [Vitis vinifera] |  |  |  |
| EY655817 | S44211439 | 24 | 100 | 21.91965 | 109.4908 | 2.320513173 | 0 | cs00-c1-100-082-f09- sweet orange greenhouse plant citrus sinensis mrna |  |  |  |
| EY656041 | S44211663 | 138 | 36 | 126.038 | 39.41669 | -1.676979971 | 0 | subtilisin-like protease ame: full=cucumisin-like serine protease flags: precursor |  |  |  |
| EY656063 | S44211685 | 51 | 2 | 46.57925 | 2.189816 | -4.410805858 | 0 | uncharacterized basic helix-loop-helix protein at1g06150 |  |  |  |
| EY656296 | S44211806 | 14 | 34 | 12.78646 | 37.22687 | 1.541727403 | 0 | tocopherol chloroplastic ame: full=vitamin e pathway gene 1 protein ame: full=sucrose export defective 1 flags: precursor |  |  |  |
| EY656328 | S44211838 | 168 | 44 | 153.4375 | 48.17595 | -1.67126632 | 0 | dna-damage-inducible protein |  |  |  |
| EY656444 | S44211954 | 17 | 54 | 15.52642 | 59.12504 | 1.929044145 | 0 | serine carboxypeptidase-like 6 flags: precursor |  |  |  |
| EY656449 | S44211959 | 67 | 4 | 61.19235 | 4.379632 | -3.804469706 | 0 | myristoyl-acyl carrier protein chloroplastic ame: full=16:0-acyl-carrier protein thioesterase� |  |  |  |
| EY656512 | S44212022 | 151 | 363 | 137.9111 | 397.4516 | 1.527040483 | 0 | poncirus trifoliata citrus tristeza virus resistance gene complete sequence |  |  |  |
| EY656524 | S44212034 | 55 | 4 | 50.23253 | 4.379632 | -3.519740229 | 0 | protein hothead ame: full=protein adhesion of calyx edges flags: precursor |  |  |  |
| EY656545 | S44212055 | 6 | 13 | 5.479912 | 14.2338 | 1.377096701 | 0 | abc transporter g family member 5� |  |  |  |
| EY657030 | S44212274 | 75 | 16 | 68.4989 | 17.51853 | -1.967199206 | 0 | nucleic acid binding |  |  |  |
| EY657220 | S44212422 | 92 | 5 | 84.02532 | 5.47454 | -3.940014377 | 0 | l-ascorbate oxidase homolog ame: full=pollen-specific protein ntp303 flags: precursor |  |  |  |
| EY657482 | S44212628 | 10911 | 2021 | 9965.22 | 2212.809 | -2.171022621 | 0 | probable non-specific lipid-transfer protein akcs9� |  |  |  |
| EY657896 | S44212930 | 1273 | 2785 | 1162.655 | 3049.319 | 1.391064392 | 0 | ring-h2 zinc finger protein rha2a |  |  |  |
| EY657918 | S44212952 | 146 | 361 | 133.3445 | 395.2618 | 1.567649952 | 0 | predicted protein [Populus trichocarpa] |  |  |  |
| EY658086 | S44213120 | 79 | 15 | 72.15218 | 16.42362 | -2.135270668 | 0 | myb family transcription factor apl� |  |  |  |
| EY658129 | S44213149 | 201 | 22 | 183.5771 | 24.08798 | -2.930000588 | 0 | aquaporin nip2-1 ame: full=nod26-like intrinsic protein 2-1 ame: full= 2 1 ame: full=silicon transporter lsi1 ame: full=low silicon protein 1 |  |  |  |
| EY658336 | S44213244 | 19 | 0 | 17.35306 | 0 | -Inf | 0 | 14 kda proline-rich protein flags: precursor |  |  |  |
| EY658341 | S44213249 | 243 | 59 | 221.9364 | 64.59958 | -1.78054997 | 0 | chalcone-flavanone isomerase family expressed |  |  |  |
| EY658528 | S44213324 | 1272 | 306 | 1161.741 | 335.0419 | -1.793875629 | 0 | probable glycerophosphoryl diester phosphodiesterase 2 flags: precursor |  |  |  |
| EY658548 | S44213344 | 2 | 9 | 1.826637 | 9.854173 | 2.431544486 | 0 | citrus sinensis complete genome |  |  |  |
| EY658585 | S44213381 | 306 | 38 | 279.4755 | 41.60651 | -2.747840845 | 0 | solute carrier family 35 member f1 |  |  |  |
| EY658610 | S44213406 | 52 | 2 | 47.49257 | 2.189816 | -4.438820234 | 0 | gdsl esterase lipase at2g04570 ame: full=extracellular lipase at2g04570 flags: precursor |  |  |  |
| EY658658 | S44213454 | 304 | 70 | 277.6489 | 76.64356 | -1.857025012 | 0 | udp-glucoronosyl udp-glucosyl transferase family protein |  |  |  |
| EY658729 | S44213525 | 13 | 1 | 11.87314 | 1.094908 | -3.438820234 | 0 | cs00-c1-100-138-f12- sweet orange greenhouse plant citrus sinensis mrna |  |  |  |
| EY658787 | S44213583 | 847 | 184 | 773.5809 | 201.4631 | -1.941036719 | 0 | (+)-delta-cadinene synthase isozyme a� |  |  |  |
| EY658792 | S44213588 | 13 | 2 | 11.87314 | 2.189816 | -2.438820234 | 0 | taxadien-5-alpha-ol o-acetyltransferase ame: full=taxa-4 -dien-5alpha-ol-o-acetyltransferase� |  |  |  |
| EY658811 | S44213607 | 453 | 37 | 413.7334 | 40.5116 | -3.35229439 | 0 | fasciclin-like arabinogalactan protein 2 flags: precursor |  |  |  |
| EY659177 | S44213763 | 96 | 264 | 87.67859 | 289.0557 | 1.721051103 | 0 | cs00-c1-101-004-c05- sweet orange infected with xylella fastidiosa (stage 1 of 2) citrus sinensis mrna |  |  |  |
| EY659914 | S44214388 | 4 | 13 | 3.653275 | 14.2338 | 1.962059202 | 0 | transcription factor myb39 ame: full=myb-related protein 39� |  |  |  |
| EY659982 | S44214456 | 110 | 258 | 100.4651 | 282.4863 | 1.491487026 | 0 | brassica oleracea clone bac complete sequence |  |  |  |
| EY660105 | S44214467 | 2 | 11 | 1.826637 | 12.04399 | 2.721051103 | 0 | cs00-c1-101-018-f01- sweet orange infected with xylella fastidiosa (stage 1 of 2) citrus sinensis mrna |  |  |  |
| EY660196 | S44214558 | 37 | 91 | 33.79279 | 99.63663 | 1.559960759 | 0 | probable wrky transcription factor 19 ame: full=wrky dna-binding protein 19 |  |  |  |
| EY660232 | S44214594 | 42 | 9 | 38.35938 | 9.854173 | -1.960772937 | 0 | s-locus-specific glycoprotein s6� |  |  |  |
| EY660243 | S44214605 | 1920 | 406 | 1753.572 | 444.5327 | -1.979935194 | 0 | tubulin alpha-1 chain |  |  |  |
| EY660318 | S44214680 | 2 | 0 | 1.826637 | 0 | -Inf | 0 | cs00-c1-101-020-h10- sweet orange infected with xylella fastidiosa (stage 1 of 2) citrus sinensis mrna |  |  |  |
| EY660325 | S44214687 | 367 | 742 | 335.188 | 812.4218 | 1.277258608 | 0 | populus est from severe drought-stressed opposite wood |  |  |  |
| EY660383 | S44214745 | 332 | 69 | 303.2218 | 75.54866 | -2.00489549 | 0 | probable pectinesterase pectinesterase inhibitor 54 includes: ame: full=pectinesterase inhibitor 54 ame: full=pectin methylesterase inhibitor 54 includes: ame: full=pectinesterase 54� |  |  |  |
| EY660548 | S44214910 | 148 | 321 | 135.1712 | 351.4655 | 1.378595606 | 0 | zinc finger protein 4 |  |  |  |
| EY660567 | S44214929 | 54 | 9 | 49.31921 | 9.854173 | -2.323343017 | 0 | probable lrr receptor-like serine threonine-protein kinase at3g47570 flags: precursor |  |  |  |
| EY661071 | S44215209 | 132 | 12 | 120.5581 | 13.1389 | -3.197812135 | 0 | cs00-c1-101-029-b02- sweet orange infected with xylella fastidiosa (stage 1 of 2) citrus sinensis mrna |  |  |  |
| EY661918 | S44215608 | 147 | 6 | 134.2578 | 6.569448 | -4.35309036 | 0 | protein |  |  |  |
| EY663583 | S44216489 | 41 | 1 | 37.44607 | 1.094908 | -5.095932521 | 0 | cs00-c1-101-056-g04- sweet orange infected with xylella fastidiosa (stage 1 of 2) citrus sinensis mrna |  |  |  |
| EY664267 | S44217061 | 16 | 1 | 14.6131 | 1.094908 | -3.738380516 | 0 | cs00-c1-101-064-g02- sweet orange infected with xylella fastidiosa (stage 1 of 2) citrus sinensis mrna |  |  |  |
| EY664576 | S44217370 | 2798 | 6721 | 2555.466 | 7358.877 | 1.525899425 | 0 | protein |  |  |  |
| EY665087 | S44217657 | 42 | 7 | 38.35938 | 7.664356 | -2.323343017 | 0 | rab6-interacting golgin ame: full=n-terminal kinase-like-binding protein 1� |  |  |  |
| EY665163 | S44217733 | 8 | 26 | 7.306549 | 28.46761 | 1.962059202 | 0 | elongation factor tu gtp-binding domain-containing protein 1 ame: full=protein fam42a |  |  |  |
| EY665215 | S44217785 | 116 | 19 | 105.945 | 20.80325 | -2.348433998 | 0 | glycerol-3-phosphate acyltransferase 6� |  |  |  |
| EY665231 | S44217801 | 686 | 1490 | 626.5366 | 1631.413 | 1.380651333 | 0 | �zinc finger protein at1g68190 |  |  |  |
| EY665285 | S44217855 | 10 | 43 | 9.133187 | 47.08105 | 2.365956144 | 0 | cs00-c1-101-086-b08- sweet orange infected with xylella fastidiosa (stage 1 of 2) citrus sinensis mrna |  |  |  |
| EY665389 | S44217959 | 223 | 54 | 203.6701 | 59.12504 | -1.784392914 | 0 | cs00-c1-101-007-d02- sweet orange infected with xylella fastidiosa (stage 1 of 2) citrus sinensis mrna |  |  |  |
| EY666753 | S44219001 | 119 | 302 | 108.6849 | 330.6622 | 1.60520646 | 0 | probable aminotransferase acs10 |  |  |  |
| EY666801 | S44219049 | 87 | 202 | 79.45873 | 221.1714 | 1.476887471 | 0 | protein |  |  |  |
| EY667487 | S44219511 | 194 | 478 | 177.1838 | 523.3661 | 1.56257345 | 0 | abc transporter c family member 10� |  |  |  |
| EY668011 | S44219699 | 108 | 332 | 98.63842 | 363.5095 | 1.881771413 | 0 | reticuline oxidase-like protein flags: precursor |  |  |  |
| EY668407 | S44219983 | 1636 | 146 | 1494.189 | 159.8566 | -3.22451299 | 0 | carbonic chloroplastic ame: full=carbonate dehydratase flags: precursor |  |  |  |
| EY668560 | S44220136 | 87 | 177 | 79.45873 | 193.7987 | 1.286281538 | 0 | cs00-c1-102-043-a04- sweet orange infected with xylella fastidiosa (stage 2 of 2) citrus sinensis mrna |  |  |  |
| EY668853 | S44220429 | 25 | 121 | 22.83297 | 132.4839 | 2.536626532 | 0 | PREDICTED: hypothetical protein [Vitis vinifera] |  |  |  |
| EY669382 | S44220958 | 18 | 48 | 16.43974 | 52.55559 | 1.676656983 | 0 | cs00-c1-102-074-c04- sweet orange infected with xylella fastidiosa (stage 2 of 2) citrus sinensis mrna |  |  |  |
| EY669763 | S44221017 | 173 | 38 | 158.0041 | 41.60651 | -1.92508123 | 0 | �chloroplastic� |  |  |  |
| EY669973 | S44221227 | 288 | 76 | 263.0358 | 83.21301 | -1.660378004 | 0 | ankyrin repeat-containing |  |  |  |
| EY670039 | S44221293 | 144 | 329 | 131.5179 | 360.2248 | 1.453638256 | 0 | probable aminotransferase acs10 |  |  |  |
| EY670060 | S44221314 | 106 | 691 | 96.81178 | 756.5815 | 2.96624093 | 0 | protein |  |  |  |
| EY670781 | S44221811 | 16 | 50 | 14.6131 | 54.7454 | 1.905475674 | 0 | protein |  |  |  |
| EY671042 | S44221960 | 19 | 47 | 17.35306 | 51.46068 | 1.568280822 | 0 | cs00-c1-102-075-a04- sweet orange infected with xylella fastidiosa (stage 2 of 2) citrus sinensis mrna |  |  |  |
| EY672374 | S44222844 | 4 | 20 | 3.653275 | 21.89816 | 2.583547579 | 0 | cs00-c1-102-109-d06- sweet orange infected with xylella fastidiosa (stage 2 of 2) citrus sinensis mrna |  |  |  |
| EY672423 | S44222893 | 2425 | 226 | 2214.798 | 247.4492 | -3.161970585 | 0 | heat shock protein 83 |  |  |  |
| EY672670 | S44223028 | 451 | 1091 | 411.9067 | 1194.545 | 1.536071247 | 0 | thioredoxin-like 1 |  |  |  |
| EY673147 | S44223057 | 152 | 335 | 138.8244 | 366.7942 | 1.401709256 | 0 | cs00-c1-102-105-f12- sweet orange infected with xylella fastidiosa (stage 2 of 2) citrus sinensis mrna |  |  |  |
| EY673259 | S44223169 | 287 | 634 | 262.1225 | 694.1717 | 1.405051588 | 0 | uncharacterized protein at1g14870 |  |  |  |
| EY673920 | S44223494 | 261 | 56 | 238.3762 | 61.31485 | -1.95893159 | 0 | cs00-c1-102-004-g03- sweet orange infected with xylella fastidiosa (stage 2 of 2) citrus sinensis mrna |  |  |  |
| EY674119 | S44223595 | 24 | 3 | 21.91965 | 3.284724 | -2.738380516 | 0 | probable lrr receptor-like serine threonine-protein kinase at2g23950 flags: precursor |  |  |  |
| EY674214 | S44223690 | 132 | 413 | 120.5581 | 452.197 | 1.907223336 | 0 | protein chloroplast import apparatus 2 flags: precursor |  |  |  |
| EY674234 | S44223710 | 8 | 25 | 7.306549 | 27.3727 | 1.905475674 | 0 | cs00-c1-102-024-a07- sweet orange infected with xylella fastidiosa (stage 2 of 2) citrus sinensis mrna |  |  |  |
| EY674342 | S44223818 | 10 | 32 | 9.133187 | 35.03706 | 1.939691389 | 0 | cs00-c1-102-075-b02- sweet orange infected with xylella fastidiosa (stage 2 of 2) citrus sinensis mrna |  |  |  |
| EY674438 | S44223914 | 210 | 671 | 191.7969 | 734.6833 | 1.937542923 | 0 | yela protein gb |  |  |  |
| EY674909 | S44224175 | 14 | 1 | 12.78646 | 1.094908 | -3.545735438 | 0 | phospho-2-dehydro-3-deoxyheptonate tyr-sensitive ame: full=phospho-2-keto-3-deoxyheptonate aldolase ame: full=3-deoxy-d-arabino-heptulosonate 7-phosphate synthase ame: full=dahp synthetase |  |  |  |
| EY674949 | S44224215 | 2 | 25 | 1.826637 | 27.3727 | 3.905475674 | 0 | dibenzothiophene desulfurization enzyme c ame: full=dbt sulfur dioxygenase |  |  |  |
| EY675106 | S44224372 | 1235 | 146 | 1127.949 | 159.8566 | -2.818851284 | 0 | probable xyloglucan endotransglucosylase hydrolase protein 6� |  |  |  |
| EY675187 | S44224453 | 535 | 142 | 488.6255 | 155.4769 | -1.652028478 | 0 | cs00-c1-401-007-c07- sweet orange infected with citrus sinensis mrna |  |  |  |
| EY675370 | S44224636 | 130 | 628 | 118.7314 | 687.6023 | 2.53387242 | 0 | cs00-c1-401-009-f08- sweet orange infected with citrus sinensis mrna |  |  |  |
| EY675407 | S44224673 | 2650 | 510 | 2420.295 | 558.4031 | -2.115803723 | 0 | heat shock factor protein |  |  |  |
| EY675468 | S44224734 | 14 | 4 | 12.78646 | 4.379632 | -1.545735438 | 0 | cs00-c1-401-010-h12- sweet orange infected with citrus sinensis mrna |  |  |  |
| EY675570 | S44224836 | 1 | 11 | 0.913319 | 12.04399 | 3.721051103 | 0 | rna-binding protein 38 ame: full=rna-binding motif protein 38 ame: full=rna-binding region-containing protein 1 ame: full=hsrnaseb ame: full=ssdna-binding protein seb4 ame: full=cll-associated antigen kw-5 |  |  |  |
| EY675698 | S44224964 | 262 | 618 | 239.2895 | 676.6532 | 1.49965951 | 0 | cytochrome p450 98a1 |  |  |  |
| EY675709 | S44224975 | 33 | 6 | 30.13952 | 6.569448 | -2.197812135 | 0 | flavoprotein wrba |  |  |  |
| EY675861 | S44225127 | 8 | 22 | 7.306549 | 24.08798 | 1.721051103 | 0 | cs00-c1-401-016-a01- sweet orange infected with citrus sinensis mrna |  |  |  |
| EY675876 | S44225142 | 78 | 213 | 71.23886 | 233.2154 | 1.710926885 | 0 | probable wrky transcription factor 70 ame: full=wrky dna-binding protein 70 |  |  |  |
| EY675953 | S44225219 | 114 | 268 | 104.1183 | 293.4354 | 1.49481866 | 0 | mouse dna sequence from clone rp23-279j20 on chromosome complete sequence |  |  |  |
| EY676054 | S44225320 | 196 | 42 | 179.0105 | 45.98614 | -1.960772937 | 0 | cs00-c1-401-018-b09- sweet orange infected with citrus sinensis mrna |  |  |  |
| EY676272 | S44225538 | 10 | 0 | 9.133187 | 0 | -Inf | 0 | gdsl esterase lipase at5g33370 ame: full=extracellular lipase at5g33370 flags: precursor |  |  |  |
| EY676495 | S44225649 | 2054 | 7130 | 1875.957 | 7806.695 | 2.057085379 | 0 | conserved hypothetical protein [Ricinus communis] |  |  |  |
| EY676513 | S44225667 | 10 | 127 | 9.133187 | 139.0533 | 3.928376076 | 0 | af283537\_1lectin-related protein precursor |  |  |  |
| EY676565 | S44225719 | 3 | 10 | 2.739956 | 10.94908 | 1.998585078 | 0 | serine carboxypeptidase-like 14 flags: precursor |  |  |  |
| EY676719 | S44225873 | 4 | 19 | 3.653275 | 20.80325 | 2.509546998 | 0 | aspartic proteinase nepenthesin-1 ame: full=nepenthesin-i flags: precursor |  |  |  |
| EY676784 | S44225938 | 429 | 47 | 391.8137 | 51.46068 | -2.928625502 | 0 | tsd2 (tumorous shoot development 2) methyltransferase |  |  |  |
| EY677139 | S44226069 | 147 | 31 | 134.2578 | 33.94215 | -1.98385655 | 0 | dreg-2 like protein |  |  |  |
| EY677677 | S44226495 | 15 | 2 | 13.69978 | 2.189816 | -2.645271112 | 0 | aquaporin aqpcic |  |  |  |
| EY677736 | S44226554 | 73 | 14 | 66.67226 | 15.32871 | -2.120850153 | 0 | protein tyrosine phosphatase-like protein ptplad1 ame: full=protein-tyrosine phosphatase-like a domain-containing protein 1 |  |  |  |
| EY677751 | S44226569 | 178 | 47 | 162.5707 | 51.46068 | -1.659525095 | 0 | rna polymerase sigma factor rpod |  |  |  |
| EY677834 | S44226652 | 70 | 30 | 63.93231 | 32.84724 | -0.960772937 | 0 | dipterocarpus tempehes microsatellite clone dt29 |  |  |  |
| EY677909 | S44226727 | 88 | 3 | 80.37204 | 3.284724 | -4.612849634 | 0 | taxadien-5-alpha-ol o-acetyltransferase ame: full=taxa-4 -dien-5alpha-ol-o-acetyltransferase� |  |  |  |
| EY677929 | S44226747 | 9 | 32 | 8.219868 | 35.03706 | 2.091694483 | 0 | lectin-4 ame: full=lectin iv ame: full=gs4 |  |  |  |
| EY678176 | S44226994 | 329 | 53 | 300.4818 | 58.03013 | -2.372403835 | 0 | serine carboxypeptidase-like 27 flags: precursor |  |  |  |
| EY678390 | S44227096 | 20 | 4 | 18.26637 | 4.379632 | -2.060308611 | 0 | aphanomyces euteiches cdna |  |  |  |
| EY678462 | S44227168 | 42 | 5 | 38.35938 | 5.47454 | -2.808769844 | 0 | gdsl esterase lipase at5g45910 ame: full=extracellular lipase at5g45910 flags: precursor |  |  |  |
| EY678471 | S44227177 | 738 | 1568 | 674.0292 | 1716.816 | 1.348852322 | 0 | nematode-resistance protein |  |  |  |
| EY678518 | S44227224 | 91 | 7 | 83.112 | 7.664356 | -3.438820234 | 0 | formin-like protein 1� |  |  |  |
| EY678592 | S44227298 | 1829 | 396 | 1670.46 | 433.5836 | -1.945863256 | 0 | universal stress protein |  |  |  |
| EY678647 | S44227353 | 56 | 143 | 51.14585 | 156.5719 | 1.614135899 | 0 | cs00-c1-401-050-g06- sweet orange infected with citrus sinensis mrna |  |  |  |
| EY678725 | S44227431 | 2 | 13 | 1.826637 | 14.2338 | 2.962059202 | 0 | cs00-c1-401-031-f11- sweet orange infected with citrus sinensis mrna |  |  |  |
| EY678786 | S44227492 | 5 | 64 | 4.566593 | 70.07412 | 3.939691389 | 0 | superoxide dismutase |  |  |  |
| EY678804 | S44227510 | 1861 | 447 | 1699.686 | 489.4239 | -1.796111835 | 0 | probable pectinesterase pectinesterase inhibitor 34 includes: ame: full=pectinesterase inhibitor 34 ame: full=pectin methylesterase inhibitor 34 includes: ame: full=pectinesterase 34� |  |  |  |
| EY678922 | S44227628 | 402 | 83 | 367.1541 | 90.87737 | -2.014392776 | 0 | beta-galactosidase 3� |  |  |  |
| EY679230 | S44227824 | 5 | 25 | 4.566593 | 27.3727 | 2.583547579 | 0 | cs00-c1-401-056-h04- sweet orange infected with citrus sinensis mrna |  |  |  |
| EY679298 | S44227892 | 11 | 0 | 10.04651 | 0 | -Inf | 0 | pmr5 (powdery mildew resistant 5) |  |  |  |
| EY679306 | S44227900 | 3 | 26 | 2.739956 | 28.46761 | 3.377096701 | 0 | predicted protein [Populus trichocarpa] |  |  |  |
| EY679310 | S44227904 | 5 | 29 | 4.566593 | 31.75233 | 2.797672384 | 0 | PREDICTED: hypothetical protein [Vitis vinifera] |  |  |  |
| EY679334 | S44227928 | 85 | 189 | 77.63209 | 206.9376 | 1.414470972 | 0 | probable adp-ribosylation factor gtpase-activating protein agd5� |  |  |  |
| EY679373 | S44227967 | 8 | 27 | 7.306549 | 29.56252 | 2.016506986 | 0 | cs00-c1-401-059-e01- sweet orange infected with citrus sinensis mrna |  |  |  |
| EY679388 | S44227982 | 1 | 15 | 0.913319 | 16.42362 | 4.16851008 | 0 | aspartic proteinase nepenthesin-1 ame: full=nepenthesin-i flags: precursor |  |  |  |
| EY679905 | S44228387 | 868 | 68 | 792.7606 | 74.45375 | -3.412468907 | 0 | aquaporin |  |  |  |
| EY681003 | S44229261 | 474 | 120 | 432.9131 | 131.389 | -1.720233169 | 0 | glycine dehydrogenase ame: full=glycine decarboxylase ame: full=glycine cleavage system p-protein |  |  |  |
| EY681849 | S44229771 | 20 | 42 | 18.26637 | 45.98614 | 1.332008812 | 0 | protein |  |  |  |
| EY682772 | S44230470 | 18 | 91 | 16.43974 | 99.63663 | 2.599489123 | 0 | at3g15630 msj11\_3 |  |  |  |
| EY683784 | S44231034 | 57 | 126 | 52.05917 | 137.9584 | 1.406009393 | 0 | cs00-c1-650-040-f04- sweet orange young greenhouse plant citrus sinensis mrna |  |  |  |
| EY683825 | S44231075 | 193 | 50 | 176.2705 | 54.7454 | -1.686981363 | 0 | upf0497 membrane protein 5 |  |  |  |
| EY683832 | S44231082 | 56 | 195 | 51.14585 | 213.5071 | 2.061594876 | 0 | �zinc metalloprotease slr1821 |  |  |  |
| EY683842 | S44231092 | 259 | 57 | 236.5495 | 62.40976 | -1.922298789 | 0 | cs00-c1-650-041-c06- sweet orange young greenhouse plant citrus sinensis mrna |  |  |  |
| EY683894 | S44231144 | 43 | 118 | 39.2727 | 129.1992 | 1.717997779 | 0 | cs00-c1-650-041-h01- sweet orange young greenhouse plant citrus sinensis mrna |  |  |  |
| EY683897 | S44231147 | 15 | 37 | 13.69978 | 40.5116 | 1.564182254 | 0 | PREDICTED: hypothetical protein [Vitis vinifera] |  |  |  |
| EY684273 | S44231187 | 17 | 46 | 15.52642 | 50.36577 | 1.697718599 | 0 | protein ruptured pollen grain 1 |  |  |  |
| EY684404 | S44231318 | 16 | 4 | 14.6131 | 4.379632 | -1.738380516 | 0 | aspartic proteinase nepenthesin-2 ame: full=nepenthesin-ii flags: precursor |  |  |  |
| EY684442 | S44231356 | 117 | 258 | 106.8583 | 282.4863 | 1.40248202 | 0 | calmodulin-binding transcription activator |  |  |  |
| EY684552 | S44231466 | 147 | 44 | 134.2578 | 48.17595 | -1.478621242 | 0 | probable inactive receptor kinase at5g58300 flags: precursor |  |  |  |
| EY684567 | S44231481 | 171 | 18 | 156.1775 | 19.70835 | -2.986308029 | 0 | Protein E6, putative [Ricinus communis] |  |  |  |
| EY684597 | S44231511 | 40 | 145 | 36.53275 | 158.7617 | 2.119600479 | 0 | storage protein |  |  |  |
| EY684760 | S44231674 | 478 | 1254 | 436.5663 | 1373.015 | 1.653074309 | 0 | probable protein phosphatase 2c 8� |  |  |  |
| EY684834 | S44231748 | 1 | 6 | 0.913319 | 6.569448 | 2.846581985 | 0 | mads box |  |  |  |
| EY684843 | S44231757 | 933 | 28 | 852.1263 | 30.65743 | -4.796758865 | 0 | monothiol glutaredoxin-s9� |  |  |  |
| EY685073 | S44231987 | 40 | 11 | 36.53275 | 12.04399 | -1.600876992 | 0 | vacuolar cation proton exchanger 2 ame: full=ca(2+) h(+) exchanger 2 ame: full= 2 |  |  |  |
| EY685091 | S44232005 | 178 | 26 | 162.5707 | 28.46761 | -2.513674229 | 0 | endoglucanase 9 ame: full=endo- -beta glucanase 9 ame: full=cellulase 3� |  |  |  |
| EY685097 | S44232011 | 47 | 8 | 42.92598 | 8.759265 | -2.292969368 | 0 | cs00-c2-003-008-g01- sweet orange greenhouse plant citrus sinensis mrna |  |  |  |
| EY685271 | S44232185 | 290 | 36 | 264.8624 | 39.41669 | -2.748364604 | 0 | vitis vinifera contig whole genome shotgun sequence |  |  |  |
| EY685389 | S44232303 | 550 | 53 | 502.3253 | 58.03013 | -3.11374787 | 0 | cytokinin-o-glucosyltransferase 2 ame: full=zeatin o-glucosyltransferase 2� |  |  |  |
| EY685391 | S44232305 | 32 | 4 | 29.2262 | 4.379632 | -2.738380516 | 0 | cs00-c2-003-018-f09- sweet orange greenhouse plant citrus sinensis mrna |  |  |  |
| EY685417 | S44232331 | 166 | 388 | 151.6109 | 424.8243 | 1.486492895 | 0 | nuclear transcription x-box |  |  |  |
| EY685521 | S44232435 | 61 | 193 | 55.71244 | 211.3173 | 1.923339184 | 0 | cs00-c2-003-020-d07- sweet orange greenhouse plant citrus sinensis mrna |  |  |  |
| EY685784 | S44232614 | 18 | 52 | 16.43974 | 56.93522 | 1.792134201 | 0 | protein |  |  |  |
| EY685877 | S44232707 | 51 | 4 | 46.57925 | 4.379632 | -3.410805858 | 0 | gdsl esterase lipase at2g04570 ame: full=extracellular lipase at2g04570 flags: precursor |  |  |  |
| EY685996 | S44232826 | 668 | 1021 | 610.0969 | 1117.901 | 0.873682342 | 0 | protein |  |  |  |
| EY686415 | S44233127 | 97 | 24 | 88.59191 | 26.27779 | -1.753330857 | 0 | senescence-associated protein 5-like protein |  |  |  |
| EY686452 | S44233164 | 112 | 24 | 102.2917 | 26.27779 | -1.960772937 | 0 | thaumatin-like protein 1 flags: precursor |  |  |  |
| EY686468 | S44233180 | 23 | 0 | 21.00633 | 0 | -Inf | 0 | probable calcium-binding protein cml44 ame: full=calmodulin-like protein 44 |  |  |  |
| EY686549 | S44233261 | 49 | 6 | 44.75262 | 6.569448 | -2.768127859 | 0 | serine-threonine protein plant- |  |  |  |
| EY687063 | S44233327 | 4 | 16 | 3.653275 | 17.51853 | 2.261619484 | 0 | cs00-c2-003-036-g03- sweet orange greenhouse plant citrus sinensis mrna |  |  |  |
| EY687323 | S44233587 | 25 | 65 | 22.83297 | 71.16902 | 1.640131107 | 0 | cs00-c2-003-040-c07- sweet orange greenhouse plant citrus sinensis mrna |  |  |  |
| EY687365 | S44233629 | 1 | 18 | 0.913319 | 19.70835 | 4.431544486 | 0 | acyl- chloroplastic ame: full=stearoyl-acp desaturase flags: precursor |  |  |  |
| EY687900 | S44233968 | 95 | 212 | 86.76528 | 232.1205 | 1.41968433 | 0 | cs00-c2-003-050-c06- sweet orange greenhouse plant citrus sinensis mrna |  |  |  |
| EY688172 | S44234128 | 2 | 13 | 1.826637 | 14.2338 | 2.962059202 | 0 | cs00-c2-003-087-b05- sweet orange greenhouse plant citrus sinensis mrna |  |  |  |
| EY688198 | S44234154 | 1449 | 727 | 1323.399 | 795.9982 | -0.733410842 | 0 | vitis vinifera contig whole genome shotgun sequence |  |  |  |
| EY688205 | S44234161 | 43 | 7 | 39.2727 | 7.664356 | -2.357290349 | 0 | probable lrr receptor-like serine threonine-protein kinase at4g37250 flags: precursor |  |  |  |
| EY688862 | S44234370 | 99 | 11 | 90.41855 | 12.04399 | -2.908305517 | 0 | cs00-c2-003-065-f01- sweet orange greenhouse plant citrus sinensis mrna |  |  |  |
| EY689397 | S44234905 | 36 | 2 | 32.87947 | 2.189816 | -3.908305517 | 0 | �kda class i heat shock protein 1 ame: full=heat shock protein ame: full=hsp ame: full=heat shock protein 17 ame: full=low molecular weight heat shock protein |  |  |  |
| EY689747 | S44235157 | 11 | 1 | 10.04651 | 1.094908 | -3.197812135 | 0 | cs00-c2-003-079-f12- sweet orange greenhouse plant citrus sinensis mrna |  |  |  |
| EY689845 | S44235255 | 24 | 2 | 21.91965 | 2.189816 | -3.323343017 | 0 | protein |  |  |  |
| EY689922 | S44235332 | 15 | 41 | 13.69978 | 44.89123 | 1.712280893 | 0 | cs00-c2-003-082-a08- sweet orange greenhouse plant citrus sinensis mrna |  |  |  |
| EY689944 | S44235354 | 30 | 31 | 27.39956 | 33.94215 | 0.308925199 | 0 | cs00-c2-003-082-c12- sweet orange greenhouse plant citrus sinensis mrna |  |  |  |
| EY690022 | S44235432 | 115 | 29 | 105.0316 | 31.75233 | -1.725889572 | 0 | o-acetyltransferase family protein |  |  |  |
| EY690060 | S44235470 | 216 | 486 | 197.2768 | 532.1253 | 1.431544486 | 0 | cs00-c2-003-083-h07- sweet orange greenhouse plant citrus sinensis mrna |  |  |  |
| EY690201 | S44235499 | 21 | 92 | 19.17969 | 100.7315 | 2.392864017 | 0 | pentatricopeptide repeat-containing protein at2g01860 ame: full=protein embryo defective 975 |  |  |  |
| EY690568 | S44235754 | 106 | 267 | 96.81178 | 292.3405 | 1.594394961 | 0 | probable serine threonine-protein kinase at5g41260 |  |  |  |
| EY690924 | S44236110 | 60 | 147 | 54.79912 | 160.9515 | 1.554401233 | 0 | cs00-c2-003-101-b07- sweet orange greenhouse plant citrus sinensis mrna |  |  |  |
| EY691353 | S44236539 | 638 | 1390 | 582.6973 | 1521.922 | 1.385076038 | 0 | at5g16110 t21h19\_30 |  |  |  |
| EY691406 | S44236592 | 13 | 3 | 11.87314 | 3.284724 | -1.853857733 | 0 | vitis vinifera contig whole genome shotgun sequence |  |  |  |
| EY691470 | S44236656 | 15 | 37 | 13.69978 | 40.5116 | 1.564182254 | 0 | cs00-c2-003-110-a10- sweet orange greenhouse plant citrus sinensis mrna |  |  |  |
| EY691949 | S44236813 | 1367 | 355 | 1248.507 | 388.6924 | -1.683502829 | 0 | burp domain-containing protein 6� |  |  |  |
| EY692014 | S44236878 | 39 | 82 | 35.61943 | 89.78246 | 1.33376927 | 0 | cs00-c2-003-086-e09- sweet orange greenhouse plant citrus sinensis mrna |  |  |  |
| EY692024 | S44236888 | 4 | 9 | 3.653275 | 9.854173 | 1.431544486 | 0 | caenorhabditis elegans yac complete sequence |  |  |  |
| EY692106 | S44236970 | 5 | 14 | 4.566593 | 15.32871 | 1.747046311 | 0 | cs00-c2-003-106-h01- sweet orange greenhouse plant citrus sinensis mrna |  |  |  |
| EY692128 | S44236992 | 31 | 4 | 28.31288 | 4.379632 | -2.692576826 | 0 | probable glucan endo- -beta-glucosidase a6 ame: full=(1- |  |  |  |
| EY692205 | S44237069 | 724 | 193 | 661.2427 | 211.3173 | -1.645769366 | 0 | cs00-c2-003-098-b08- sweet orange greenhouse plant citrus sinensis mrna |  |  |  |
| EY692313 | S44237177 | 29 | 61 | 26.48624 | 66.78939 | 1.334375827 | 0 | PREDICTED: hypothetical protein [Vitis vinifera] |  |  |  |
| EY692353 | S44237217 | 165 | 434 | 150.6976 | 475.1901 | 1.656848502 | 0 | cs00-c2-003-059-e06- sweet orange greenhouse plant citrus sinensis mrna |  |  |  |
| EY692709 | S44237461 | 15 | 2 | 13.69978 | 2.189816 | -2.645271112 | 0 | probable lrr receptor-like serine threonine-protein kinase at1g53420 flags: precursor |  |  |  |
| EY693577 | S44237769 | 6 | 256 | 5.479912 | 280.2965 | 5.676656983 | 0 | probable indole-3-acetic acid-amido synthetase ame: full=auxin-responsive gh3-like protein 1� |  |  |  |
| EY693806 | S44237886 | 19 | 69 | 17.35306 | 75.54866 | 2.122216427 | 0 | protein |  |  |  |
| EY693959 | S44238039 | 1044 | 2328 | 953.5047 | 2548.946 | 1.41858883 | 0 | histone h2ax |  |  |  |
| EY694299 | S44238281 | 88 | 8 | 80.37204 | 8.759265 | -3.197812135 | 0 | conserved hypothetical protein [Ricinus communis] |  |  |  |
| EY694314 | S44238296 | 15 | 3 | 13.69978 | 3.284724 | -2.060308611 | 0 | e3 ubiquitin-protein ligase rnf181 ame: full=ring finger protein 181 |  |  |  |
| EY694545 | S44238415 | 2 | 11 | 1.826637 | 12.04399 | 2.721051103 | 0 | cs00-c3-700-026-f12- sweet orange development stadium (1 of 6) citrus sinensis mrna |  |  |  |
| EY694708 | S44238578 | 88 | 20 | 80.37204 | 21.89816 | -1.87588404 | 0 | purple acid phosphatase 15 ame: full=phytase flags: precursor |  |  |  |
| EY695343 | S44238877 | 36 | 81 | 32.87947 | 88.68755 | 1.431544486 | 0 | expansin-like b1� |  |  |  |
| EY695365 | S44238899 | 12 | 1 | 10.95982 | 1.094908 | -3.323343017 | 0 | PREDICTED: hypothetical protein [Vitis vinifera] |  |  |  |
| EY695384 | S44238918 | 161 | 399 | 147.0443 | 436.8683 | 1.570947542 | 0 | probable protein phosphatase 2c 49� |  |  |  |
| EY695672 | S44239094 | 78 | 12 | 71.23886 | 13.1389 | -2.438820234 | 0 | probable lrr receptor-like serine threonine-protein kinase at2g23950 flags: precursor |  |  |  |
| EY695695 | S44239117 | 42 | 4 | 38.35938 | 4.379632 | -3.130697939 | 0 | probable salt tolerance-like protein at1g75540 |  |  |  |
| EY695697 | S44239119 | 390 | 2229 | 356.1943 | 2440.55 | 2.776470072 | 0 | cs00-c3-700-039-c10- sweet orange development stadium (1 of 6) citrus sinensis mrna |  |  |  |
| EY695766 | S44239188 | 178 | 39 | 162.5707 | 42.70141 | -1.928711728 | 0 | myosin heavy chain |  |  |  |
| EY695840 | S44239262 | 19 | 1 | 17.35306 | 1.094908 | -3.986308029 | 0 | zinc finger ccch domain-containing protein 22� |  |  |  |
| EY695997 | S44239419 | 39 | 2 | 35.61943 | 2.189816 | -4.023782735 | 0 | uncharacterized basic helix-loop-helix protein at1g06150 |  |  |  |
| EY696120 | S44239542 | 257 | 53 | 234.7229 | 58.03013 | -2.016084611 | 0 | protein |  |  |  |
| EY696233 | S44239655 | 132 | 32 | 120.5581 | 35.03706 | -1.782774635 | 0 | f-box family protein |  |  |  |
| EY696242 | S44239664 | 20 | 47 | 18.26637 | 51.46068 | 1.494280241 | 0 | cs00-c3-700-045-d07- sweet orange development stadium (1 of 6) citrus sinensis mrna |  |  |  |
| EY696912 | S44240222 | 15 | 29 | 13.69978 | 31.75233 | 1.212709884 | 0 | protein |  |  |  |
| EY697150 | S44240460 | 34 | 75 | 31.05284 | 82.1181 | 1.402975333 | 0 | protein |  |  |  |
| EY697161 | S44240471 | 297 | 33 | 271.2556 | 36.13197 | -2.908305517 | 0 | fiber protein fb34 |  |  |  |
| EY697658 | S44240856 | 24 | 58 | 21.91965 | 63.50467 | 1.534637978 | 0 | lotus japonicus genomic chromosome clone: complete sequence |  |  |  |
| EY697936 | S44241134 | 37 | 2 | 33.79279 | 2.189816 | -3.947833882 | 0 | glyoxylate reductase |  |  |  |
| EY697991 | S44241189 | 17 | 2 | 15.52642 | 2.189816 | -2.825843357 | 0 | predicted protein [Populus trichocarpa] |  |  |  |
| EY697996 | S44241194 | 19 | 0 | 17.35306 | 0 | -Inf | 0 | protein |  |  |  |
| EY698025 | S44241223 | 34 | 2 | 31.05284 | 2.189816 | -3.825843357 | 0 | probable pectinesterase pectinesterase inhibitor 12 includes: ame: full=pectinesterase inhibitor 12 ame: full=pectin methylesterase inhibitor 12 includes: ame: full=pectinesterase 12� |  |  |  |
| EY698407 | S44241381 | 8 | 23 | 7.306549 | 25.18289 | 1.78518144 | 0 | cs00-c3-700-071-d11- sweet orange development stadium (1 of 6) citrus sinensis mrna |  |  |  |
| EY698443 | S44241417 | 49 | 107 | 44.75262 | 117.1552 | 1.388376626 | 0 | cs00-c3-700-071-h04- sweet orange development stadium (1 of 6) citrus sinensis mrna |  |  |  |
| EY698534 | S44241508 | 197 | 41 | 179.9238 | 44.89123 | -2.002880331 | 0 | probable xyloglucan glycosyltransferase 8 ame: full=cellulose synthase-like protein c8� |  |  |  |
| EY698931 | S44241905 | 53 | 126 | 48.40589 | 137.9584 | 1.510978953 | 0 | conserved hypothetical protein [Ricinus communis] |  |  |  |
| EY698945 | S44241919 | 753 | 3387 | 687.729 | 3708.454 | 2.430905701 | 0 | ethylene-responsive transcription factor 4 ame: full=ethylene-responsive element-binding factor 4 homolog ame: full=ethylene-responsive element-binding factor 3� |  |  |  |
| EY699143 | S44242117 | 6 | 30 | 5.479912 | 32.84724 | 2.583547579 | 0 | �ac transposase ame: full=orfa |  |  |  |
| EY699652 | S44242514 | 60 | 9 | 54.79912 | 9.854173 | -2.47534611 | 0 | heat shock -like |  |  |  |
| EY699859 | S44242721 | 88 | 8 | 80.37204 | 8.759265 | -3.197812135 | 0 | thymidine kinase |  |  |  |
| EY700158 | S44243020 | 32 | 68 | 29.2262 | 74.45375 | 1.349082325 | 0 | cs00-c3-700-091-c07- sweet orange development stadium (1 of 6) citrus sinensis mrna |  |  |  |
| EY700858 | S44243496 | 345 | 1363 | 315.0949 | 1492.36 | 2.243736779 | 0 | protein |  |  |  |
| EY700861 | S44243499 | 5736 | 1537 | 5238.796 | 1682.874 | -1.638308375 | 0 | inositol-3-phosphate synthase ame: full=myo-inositol-1-phosphate synthase� |  |  |  |
| EY701155 | S44243681 | 87 | 188 | 79.45873 | 205.8427 | 1.37326484 | 0 | cs00-c3-700-103-g09- sweet orange development stadium (1 of 6) citrus sinensis mrna |  |  |  |
| EY701256 | S44243782 | 429 | 89 | 391.8137 | 97.44682 | -2.007480922 | 0 | chlorophyll a-b binding protein chloroplastic ame: full=lhci type iii cab-p4 flags: precursor |  |  |  |
| EY701281 | S44243807 | 21 | 3 | 19.17969 | 3.284724 | -2.545735438 | 0 | probable lrr receptor-like serine threonine-protein kinase at5g45780 flags: precursor |  |  |  |
| EY701288 | S44243814 | 3530 | 621 | 3224.015 | 679.9379 | -2.245383526 | 0 | rcc1 domain-containing protein 1 |  |  |  |
| EY701476 | S44244002 | 526 | 713 | 480.4056 | 780.6695 | 0.700458761 | 0 | cs00-c3-700-106-g10- sweet orange development stadium (1 of 6) citrus sinensis mrna |  |  |  |
| EY701685 | S44244211 | 264 | 21 | 241.1161 | 22.99307 | -3.390457213 | 0 | protein |  |  |  |
| EY701744 | S44244270 | 237 | 39 | 216.4565 | 42.70141 | -2.341721546 | 0 | abc transporter g family member 15� |  |  |  |
| EY701801 | S44244327 | 12 | 54 | 10.95982 | 59.12504 | 2.431544486 | 0 | dna binding protein |  |  |  |
| EY701822 | S44244348 | 578 | 2146 | 527.8982 | 2349.673 | 2.154128162 | 0 | protein |  |  |  |
| EY701826 | S44244352 | 2230 | 421 | 2036.701 | 460.9563 | -2.143532088 | 0 | hydroxyisourate hydrolase� |  |  |  |
| EY702045 | S44244571 | 60 | 13 | 54.79912 | 14.2338 | -1.944831393 | 0 | early nodulin-like protein 3 ame: full=phytocyanin-like protein flags: precursor |  |  |  |
| EY702061 | S44244587 | 2 | 7 | 1.826637 | 7.664356 | 2.068974406 | 0 | PREDICTED: hypothetical protein [Vitis vinifera] |  |  |  |
| EY702255 | S44244781 | 189 | 481 | 172.6172 | 526.6508 | 1.609270144 | 0 | e3 ubiquitin-protein ligase rglg2 ame: full=ring domain ligase 2 |  |  |  |
| EY702521 | S44244935 | 106 | 25 | 96.81178 | 27.3727 | -1.822444781 | 0 | epidermis-specific secreted glycoprotein ep1 ame: full=52 54 kda medium protein flags: precursor |  |  |  |
| EY703257 | S44245559 | 262 | 43 | 239.2895 | 47.08105 | -2.345538763 | 0 | glycosyltransferase 8 domain-containing protein 1 |  |  |  |
| EY703258 | S44245560 | 120 | 23 | 109.5982 | 25.18289 | -2.121709155 | 0 | serine carboxypeptidase-like 26 flags: precursor |  |  |  |
| EY703774 | S44245852 | 2006 | 5873 | 1832.117 | 6430.395 | 1.811395516 | 0 | ring-h2 finger protein atl5h flags: precursor |  |  |  |
| EY703785 | S44245863 | 88 | 236 | 80.37204 | 258.3983 | 1.684830915 | 0 | receptor-like protein kinase 5 ame: full=protein haesa flags: precursor |  |  |  |
| EY704766 | S44246732 | 3 | 13 | 2.739956 | 14.2338 | 2.377096701 | 0 | cs00-c3-701-036-c10- sweet orange development stadium (2 of 6) citrus sinensis mrna |  |  |  |
| EY704772 | S44246738 | 33 | 22 | 30.13952 | 24.08798 | -0.323343017 | 0 | cs00-c3-701-036-d04- sweet orange development stadium (2 of 6) citrus sinensis mrna |  |  |  |
| EY704798 | S44246764 | 38 | 115 | 34.70611 | 125.9144 | 1.859182022 | 0 | nudix hydrolase 10� |  |  |  |
| EY704814 | S44246780 | 16 | 2 | 14.6131 | 2.189816 | -2.738380516 | 0 | probable pectinesterase 29� |  |  |  |
| EY705293 | S44247259 | 46 | 9 | 42.01266 | 9.854173 | -2.092017471 | 0 | cs00-c3-701-042-a01- sweet orange development stadium (2 of 6) citrus sinensis mrna |  |  |  |
| EY706135 | S44247877 | 904 | 199 | 825.6401 | 217.8867 | -1.921934858 | 0 | chlorophyll a-b binding protein chloroplastic ame: full=lhcii type iii cab-13 flags: precursor |  |  |  |
| EY706226 | S44247968 | 4 | 10 | 3.653275 | 10.94908 | 1.583547579 | 0 | 2 -cyclic-nucleotide 2 -phosphodiesterase |  |  |  |
| EY706436 | S44248178 | 84 | 181 | 76.71877 | 198.1784 | 1.369147948 | 0 | ethylene-responsive transcription factor 2� |  |  |  |
| EY706443 | S44248185 | 63 | 13 | 57.53908 | 14.2338 | -2.015220721 | 0 | chavicol o-methyltransferase ame: full= eugenol o-methyltransferase cvomt1 ame: full=s-adenosysl-l-methionine: eugenol o-methyltransferase cvomt1 |  |  |  |
| EY706514 | S44248256 | 465 | 72 | 424.6932 | 78.83338 | -2.42954242 | 0 | snakin-1 flags: precursor |  |  |  |
| EY706524 | S44248266 | 11 | 24 | 10.04651 | 26.27779 | 1.387150366 | 0 | protein |  |  |  |
| EY706709 | S44248451 | 34 | 3 | 31.05284 | 3.284724 | -3.240880856 | 0 | protein |  |  |  |
| EY706777 | S44248519 | 77 | 21 | 70.32554 | 22.99307 | -1.612849634 | 0 | morc family cw-type zinc finger protein 3 ame: full=zinc finger cw-type coiled-coil domain protein 3 |  |  |  |
| EY706836 | S44248578 | 59 | 160 | 53.8858 | 175.1853 | 1.70090453 | 0 | 60s ribosomal protein |  |  |  |
| EY707003 | S44248745 | 18 | 41 | 16.43974 | 44.89123 | 1.449246487 | 0 | protein ruptured pollen grain 1 |  |  |  |
| EY707123 | S44248865 | 51 | 188 | 46.57925 | 205.8427 | 2.143782994 | 0 | at1g03610 f21b7\_13 |  |  |  |
| EY707340 | S44249082 | 1485 | 239 | 1356.278 | 261.683 | -2.373760924 | 0 | cytochrome p450 76c1 |  |  |  |
| EY707630 | S44249372 | 8 | 16 | 7.306549 | 17.51853 | 1.261619484 | 0 | cs00-c3-701-070-g12- sweet orange development stadium (2 of 6) citrus sinensis mrna |  |  |  |
| EY707752 | S44249494 | 5759 | 1552 | 5259.802 | 1699.297 | -1.63007028 | 0 | unnamed protein product [Vitis vinifera] |  |  |  |
| EY708121 | S44249751 | 553 | 79 | 505.0652 | 86.49774 | -2.545735438 | 0 | tubulin beta-2 chain ame: full=beta-2-tubulin |  |  |  |
| EY708222 | S44249852 | 99 | 11 | 90.41855 | 12.04399 | -2.908305517 | 0 | predicted protein [Populus trichocarpa] |  |  |  |
| EY708401 | S44250031 | 214 | 35 | 195.4502 | 38.32178 | -2.350564485 | 0 | dna-damage-inducible protein f |  |  |  |
| EY708789 | S44250209 | 193 | 28 | 176.2705 | 30.65743 | -2.523482631 | 0 | cytochrome p450 98a2 |  |  |  |
| EY709707 | S44250804 | 4013 | 10354 | 3665.148 | 11336.68 | 1.629054646 | 0 | �late blight resistance protein homolog r1a-3 |  |  |  |
| EY709806 | S44250903 | 212 | 46 | 193.6236 | 50.36577 | -1.942739014 | 0 | uncharacterized gmc-type oxidoreductase y4nj |  |  |  |
| EY709847 | S44250944 | 909 | 204 | 830.2067 | 223.3612 | -1.894091658 | 0 | myrcene chloroplastic flags: precursor |  |  |  |
| EY709989 | S44251086 | 864 | 138 | 789.1073 | 151.0973 | -2.384743561 | 0 | aquaporin pip2-7 ame: full=plasma membrane intrinsic protein 2-7� |  |  |  |
| EY710037 | S44251134 | 32050 | 4467 | 29271.86 | 4890.954 | -2.581326713 | 0 | germin-like protein subfamily 1 member 8 flags: precursor |  |  |  |
| EY710406 | S44251279 | 99 | 275 | 90.41855 | 301.0997 | 1.735550672 | 0 | probable lrr receptor-like serine threonine-protein kinase at1g56140 flags: precursor |  |  |  |
| EY710516 | S44251389 | 613 | 85 | 559.8644 | 93.06719 | -2.588732843 | 0 | cytokinin-n-glucosyltransferase 1 ame: full=udp-glucosyl transferase 76c1 |  |  |  |
| EY710580 | S44251453 | 12 | 2 | 10.95982 | 2.189816 | -2.323343017 | 0 | PREDICTED: hypothetical protein [Vitis vinifera] |  |  |  |
| EY710672 | S44251545 | 84 | 18 | 76.71877 | 19.70835 | -1.960772937 | 0 | f-box family protein |  |  |  |
| EY710689 | S44251562 | 83 | 22 | 75.80545 | 24.08798 | -1.653988329 | 0 | l-ascorbate oxidase homolog ame: full=pollen-specific protein ntp303 flags: precursor |  |  |  |
| EY710697 | S44251570 | 767 | 171 | 700.5154 | 187.2293 | -1.903610769 | 0 | homogentisate geranylgeranyl transferase |  |  |  |
| EY710725 | S44251598 | 576 | 140 | 526.0716 | 153.2871 | -1.7790225 | 0 | cs00-c3-701-086-e02- sweet orange development stadium (2 of 6) citrus sinensis mrna |  |  |  |
| EY710962 | S44251723 | 32305 | 4919 | 29504.76 | 5385.853 | -2.453701031 | 0 | nitrate transporter ame: full=nitrate permease |  |  |  |
| EY711087 | S44251848 | 30231 | 2281 | 27610.54 | 2497.485 | -3.466670866 | 0 | small heat shock chloroplastic flags: precursor |  |  |  |
| EY711107 | S44251868 | 59 | 10 | 53.8858 | 10.94908 | -2.29909547 | 0 | cs00-c3-702-003-h02- sweet orange development stadium (3 of 6) citrus sinensis mrna |  |  |  |
| EY711614 | S44252263 | 658 | 171 | 600.9637 | 187.2293 | -1.682471775 | 0 | secologanin synthase� |  |  |  |
| EY711963 | S44252290 | 22 | 2 | 20.09301 | 2.189816 | -3.197812135 | 0 | ethylene-responsive transcription factor erf034 |  |  |  |
| EY712132 | S44252459 | 192 | 43 | 175.3572 | 47.08105 | -1.897078262 | 0 | retrotransposable element tf2 155 kda protein type 3 |  |  |  |
| EY712246 | S44252573 | 10 | 18 | 9.133187 | 19.70835 | 1.109616391 | 0 | cs00-c3-702-017-b03- sweet orange development stadium (3 of 6) citrus sinensis mrna |  |  |  |
| EY712436 | S44252763 | 81 | 6 | 73.97881 | 6.569448 | -3.493268018 | 0 | cs00-c3-702-019-c10- sweet orange development stadium (3 of 6) citrus sinensis mrna |  |  |  |
| EY713598 | S44253365 | 62 | 15 | 56.62576 | 16.42362 | -1.785686231 | 0 | poncirus trifoliata citrus tristeza virus resistance gene complete sequence |  |  |  |
| EY713903 | S44253558 | 91 | 5 | 83.112 | 5.47454 | -3.924247061 | 0 | anthocyanidin 3-o-glucosyltransferase ame: full=flavonol 3-o-glucosyltransferase ame: full=udp-glucose flavonoid 3-o-glucosyltransferase ame: full=anthocyanin rhamnosyl transferase |  |  |  |
| EY714309 | S44253740 | 18 | 3 | 16.43974 | 3.284724 | -2.323343017 | 0 | protein |  |  |  |
| EY714406 | S44253837 | 17 | 45 | 15.52642 | 49.27086 | 1.666009739 | 0 | cs00-c3-702-042-b02- sweet orange development stadium (3 of 6) citrus sinensis mrna |  |  |  |
| EY714464 | S44253895 | 17 | 37 | 15.52642 | 40.5116 | 1.383610008 | 0 | cs00-c3-702-042-g04- sweet orange development stadium (3 of 6) citrus sinensis mrna |  |  |  |
| EY714594 | S44254025 | 63 | 9 | 57.53908 | 9.854173 | -2.545735438 | 0 | auxin-induced protein 15a |  |  |  |
| EY715551 | S44254422 | 18 | 56 | 16.43974 | 61.31485 | 1.899049405 | 0 | cs00-c3-702-055-g10- sweet orange development stadium (3 of 6) citrus sinensis mrna |  |  |  |
| EY716101 | S44254748 | 526 | 1517 | 480.4056 | 1660.976 | 1.789705865 | 0 | predicted protein [Populus trichocarpa] |  |  |  |
| EY716580 | S44255003 | 1090 | 136 | 995.5174 | 148.9075 | -2.741030094 | 0 | poncirus trifoliata citrus tristeza virus resistance gene complete sequence |  |  |  |
| EY717821 | S44255698 | 33 | 3 | 30.13952 | 3.284724 | -3.197812135 | 0 | wpp domain-associated protein |  |  |  |
| EY718514 | S44256083 | 67 | 15 | 61.19235 | 16.42362 | -1.897579111 | 0 | cs00-c3-702-100-c01- sweet orange development stadium (3 of 6) citrus sinensis mrna |  |  |  |
| EY718638 | S44256207 | 624 | 2363 | 569.9109 | 2587.268 | 2.182621179 | 0 | f-box kelch-repeat protein at2g44130 |  |  |  |
| EY718653 | S44256216 | 645 | 171 | 589.0906 | 187.2293 | -1.653683351 | 0 | farnesylated protein |  |  |  |
| EY718737 | S44256300 | 70 | 11 | 63.93231 | 12.04399 | -2.408231914 | 0 | dna binding |  |  |  |
| EY718833 | S44256396 | 9815 | 1975 | 8964.223 | 2162.443 | -2.05151613 | 0 | 3 -hydroxy-n-methyl- -coclaurine 4 -o-methyltransferase ame: full=s-adenosyl-l-methionine:3 -hydroxy-n-methylcoclaurine 4 -o-methyltransferase� |  |  |  |
| EY719020 | S44256583 | 577 | 2950 | 526.9849 | 3229.979 | 2.615691215 | 0 | myb-like protein g |  |  |  |
| EY719394 | S44256859 | 1169 | 300 | 1067.67 | 328.4724 | -1.70062104 | 0 | chlorophyll a-b binding protein chloroplastic ame: full=light-harvesting complex ii protein 5 ame: full=lhcb5 ame: full=lhciic flags: precursor |  |  |  |
| EY719455 | S44256920 | 29 | 60 | 26.48624 | 65.69448 | 1.310529085 | 0 | cs00-c3-702-085-e04- sweet orange development stadium (3 of 6) citrus sinensis mrna |  |  |  |
| EY719478 | S44256943 | 20 | 45 | 18.26637 | 49.27086 | 1.431544486 | 0 | zinc finger protein magpie |  |  |  |
| EY719486 | S44256951 | 1074 | 288 | 980.9043 | 315.3335 | -1.637233792 | 0 | cucumisin ame: allergen=cuc m 1 flags: precursor |  |  |  |
| EY719507 | S44256972 | 52762 | 14159 | 48188.52 | 15502.8 | -1.636160392 | 0 | heat shock cognate 70 kda protein 1� |  |  |  |
| EY719773 | S44257238 | 2466 | 565 | 2252.244 | 618.6231 | -1.864230543 | 0 | protein |  |  |  |
| EY719785 | S44257250 | 950 | 1891 | 867.6528 | 2070.471 | 1.254769429 | 0 | cs00-c3-702-106-g10- sweet orange development stadium (3 of 6) citrus sinensis mrna |  |  |  |
| EY721129 | S44258364 | 965 | 202 | 881.3525 | 221.1714 | -1.994554165 | 0 | myrcene chloroplastic flags: precursor |  |  |  |
| EY721482 | S44258605 | 3 | 10 | 2.739956 | 10.94908 | 1.998585078 | 0 | cs00-c3-703-023-e12- sweet orange development stadium (4 of 6) citrus sinensis mrna |  |  |  |
| EY721686 | S44258809 | 20 | 45 | 18.26637 | 49.27086 | 1.431544486 | 0 | protein |  |  |  |
| EY722769 | S44259332 | 10 | 51 | 9.133187 | 55.84031 | 2.612116731 | 0 | populus trichocarpa mrna |  |  |  |
| EY722898 | S44259461 | 50 | 2 | 45.66593 | 2.189816 | -4.382236706 | 0 | pseudomonas fluorescens sbw25 complete genome |  |  |  |
| EY723197 | S44259760 | 215 | 50 | 196.3635 | 54.7454 | -1.842717176 | 0 | heavy metal-associated domain containing expressed |  |  |  |
| EY723222 | S44259785 | 271 | 68 | 247.5094 | 74.45375 | -1.733066716 | 0 | protein srg1� |  |  |  |
| EY723374 | S44259937 | 193 | 553 | 176.2705 | 605.4842 | 1.780298117 | 0 | af389293\_1 at5g26741 |  |  |  |
| EY723927 | S44260392 | 8 | 20 | 7.306549 | 21.89816 | 1.583547579 | 0 | cs00-c3-703-065-c02- sweet orange development stadium (4 of 6) citrus sinensis mrna |  |  |  |
| EY724756 | S44260997 | 945 | 229 | 863.0862 | 250.7339 | -1.783347247 | 0 | cytochrome p450 93a3 ame: full=p450 cp5 |  |  |  |
| EY725739 | S44261652 | 56 | 8 | 51.14585 | 8.759265 | -2.545735438 | 0 | probable lrr receptor-like serine threonine-protein kinase at4g08850 flags: precursor |  |  |  |
| EY726389 | S44262299 | 3395 | 886 | 3100.717 | 970.0885 | -1.676413486 | 0 | upf0717 protein at5g11950 |  |  |  |
| EY726653 | S44262339 | 527 | 93 | 481.3189 | 101.8265 | -2.240880856 | 0 | probable carotenoid cleavage dioxygenase chloroplastic� |  |  |  |
| EY726913 | S44262599 | 182 | 44 | 166.224 | 48.17595 | -1.786743537 | 0 | protein |  |  |  |
| EY727509 | S44262859 | 1046 | 190 | 955.3313 | 208.0325 | -2.199192044 | 0 | tsd2 (tumorous shoot development 2) methyltransferase |  |  |  |
| EY727536 | S44262886 | 2487 | 568 | 2271.424 | 621.9078 | -1.868824189 | 0 | flavonoid 3 -hydroxylase 1� |  |  |  |
| EY727543 | S44262893 | 4378 | 490 | 3998.509 | 536.505 | -2.897798816 | 0 | protein |  |  |  |
| EY727590 | S44262940 | 1552 | 411 | 1417.471 | 450.0072 | -1.655298774 | 0 | cs00-c3-703-103-b09- sweet orange development stadium (4 of 6) citrus sinensis mrna |  |  |  |
| EY728096 | S44263110 | 427 | 75 | 389.9871 | 82.1181 | -2.247654085 | 0 | isoflavone-7-o-methyltransferase 9 ame: full=isoflavone-o-methyltransferase 9 ame: full=7 iomt-9 |  |  |  |
| EY728143 | S44263157 | 6 | 15 | 5.479912 | 16.42362 | 1.583547579 | 0 | brassica rapa pekinensis clone complete sequence |  |  |  |
| EY729003 | S44263793 | 19 | 6 | 17.35306 | 6.569448 | -1.401345529 | 0 | cs00-c3-704-011-d02- sweet orange development stadium (5 of 6) citrus sinensis mrna |  |  |  |
| EY729053 | S44263843 | 1435 | 104 | 1310.612 | 113.8704 | -3.524775819 | 0 | hypothetical protein [Vitis vinifera] |  |  |  |
| EY729113 | S44263903 | 118 | 74 | 107.7716 | 81.0232 | -0.4115702 | 0 | probable receptor-like protein kinase at5g39030 flags: precursor |  |  |  |
| EY729149 | S44263939 | 1372 | 357 | 1253.073 | 390.8822 | -1.680665018 | 0 | 21 kda protein ame: full= protein flags: precursor |  |  |  |
| EY730117 | S44264683 | 18 | 95 | 16.43974 | 104.0163 | 2.661550091 | 0 | f-box and wd40 domain |  |  |  |
| EY730142 | S44264708 | 29 | 108 | 26.48624 | 118.2501 | 2.158525991 | 0 | aphanomyces euteiches cdna |  |  |  |
| EY731619 | S44265737 | 12764 | 2751 | 11657.6 | 3012.092 | -1.952432985 | 0 | heat shock protein 101 |  |  |  |
| EY731835 | S44265953 | 17 | 4 | 15.52642 | 4.379632 | -1.825843357 | 0 | ricinus communis beta- mrna |  |  |  |
| EY732591 | S44266597 | 20 | 51 | 18.26637 | 55.84031 | 1.612116731 | 0 | cs00-c3-704-064-b12- sweet orange development stadium (5 of 6) citrus sinensis mrna |  |  |  |
| EY732751 | S44266757 | 10 | 0 | 9.133187 | 0 | -Inf | 0 | cs00-c3-704-066-e09- sweet orange development stadium (5 of 6) citrus sinensis mrna |  |  |  |
| EY732913 | S44266919 | 678 | 138 | 619.2301 | 151.0973 | -2.034997522 | 0 | �kda heat shock mitochondrial� |  |  |  |
| EY733084 | S44267090 | 9 | 1 | 8.219868 | 1.094908 | -2.908305517 | 0 | cs00-c3-704-071-g02- sweet orange development stadium (5 of 6) citrus sinensis mrna |  |  |  |
| EY733167 | S44267173 | 16 | 40 | 14.6131 | 43.79632 | 1.583547579 | 0 | chromatin remodelling complex atpase chain isw- |  |  |  |
| EY733394 | S44267400 | 679 | 159 | 620.1434 | 174.0904 | -1.832765325 | 0 | hydroxyisourate hydrolase� |  |  |  |
| EY734078 | S44267882 | 22 | 5 | 20.09301 | 5.47454 | -1.87588404 | 0 | cs00-c3-704-081-c08- sweet orange development stadium (5 of 6) citrus sinensis mrna |  |  |  |
| EY734089 | S44267893 | 5 | 31 | 4.566593 | 33.94215 | 2.8938877 | 0 | cs00-c3-704-081-d10- sweet orange development stadium (5 of 6) citrus sinensis mrna |  |  |  |
| EY734302 | S44268106 | 9 | 23 | 8.219868 | 25.18289 | 1.615256439 | 0 | cs00-c3-704-085-f12- sweet orange development stadium (5 of 6) citrus sinensis mrna |  |  |  |
| EY734601 | S44268293 | 0 | 10 | 0 | 10.94908 | Inf | 0 | cs00-c3-704-104-e01- sweet orange development stadium (5 of 6) citrus sinensis mrna |  |  |  |
| EY734701 | S44268393 | 6 | 1 | 5.479912 | 1.094908 | -2.323343017 | 0 | cs00-c3-704-098-c02- sweet orange development stadium (5 of 6) citrus sinensis mrna |  |  |  |
| EY734777 | S44268469 | 4 | 12 | 3.653275 | 13.1389 | 1.846581985 | 0 | cs00-c3-704-087-e05- sweet orange development stadium (5 of 6) citrus sinensis mrna |  |  |  |
| EY735535 | S44268779 | 5 | 11 | 4.566593 | 12.04399 | 1.399123008 | 0 | cs00-c3-704-090-g12- sweet orange development stadium (5 of 6) citrus sinensis mrna |  |  |  |
| EY735636 | S44268880 | 29 | 65 | 26.48624 | 71.16902 | 1.426006302 | 0 | sucrose-phosphate synthase 2 ame: full=udp-glucose-fructose-phosphate glucosyltransferase 2 |  |  |  |
| EY736067 | S44269199 | 2 | 10 | 1.826637 | 10.94908 | 2.583547579 | 0 | cs00-c3-705-009-e12- sweet orange development stadium (6 of 6) citrus sinensis mrna |  |  |  |
| EY737857 | S44270647 | 387 | 99 | 353.4543 | 108.3959 | -1.705213652 | 0 | cs00-c3-705-036-a06- sweet orange development stadium (6 of 6) citrus sinensis mrna |  |  |  |
| EY738795 | S44271473 | 469 | 101 | 428.3465 | 110.5857 | -1.953613146 | 0 | ent-kaurenoic acid oxidase 2� |  |  |  |
| EY740108 | S44272352 | 346 | 88 | 316.0083 | 96.35191 | -1.713577125 | 0 | serine threonine-protein kinase afc2 |  |  |  |
| EY740624 | S44272644 | 1757 | 4044 | 1604.701 | 4427.808 | 1.46428829 | 0 | ethylene-responsive transcription factor erf104 |  |  |  |
| EY741041 | S44272949 | 708 | 1631 | 646.6296 | 1785.795 | 1.465555001 | 0 | conserved hypothetical protein [Ricinus communis] |  |  |  |
| EY741382 | S44273290 | 361 | 709 | 329.708 | 776.2898 | 1.235406274 | 0 | ent-kaurene oxidase� |  |  |  |
| EY741537 | S44273445 | 159 | 43 | 145.2177 | 47.08105 | -1.624998717 | 0 | cs00-c3-705-086-a10- sweet orange development stadium (6 of 6) citrus sinensis mrna |  |  |  |
| EY741985 | S44273669 | 17 | 22 | 15.52642 | 24.08798 | 0.633588261 | 0 | photosystem i assembly protein ycf4 |  |  |  |
| EY742045 | S44273729 | 70 | 16 | 63.93231 | 17.51853 | -1.867663533 | 0 | anthranilate n-benzoyltransferase protein 3 ame: full=anthranilate n-hydroxycinnamoyl benzoyltransferase 3 |  |  |  |
| EY742616 | S44274076 | 241 | 31 | 220.1098 | 33.94215 | -2.697073542 | 0 | lob domain-containing protein 4 ame: full=asymmetric leaves 2-like protein 6� |  |  |  |
| EY742851 | S44274199 | 3583 | 677 | 3272.421 | 741.2528 | -2.142320821 | 0 | (+)-delta-cadinene synthase isozyme a� |  |  |  |
| EY742871 | S44274219 | 187 | 43 | 170.7906 | 47.08105 | -1.859010221 | 0 | major allergen pru av 1 ame: full=allergen pru a 1 ame: allergen=pru av 1 |  |  |  |
| EY743232 | S44274468 | 1259 | 1472 | 1149.868 | 1611.705 | 0.487118872 | 0 | cs00-c3-705-101-f11- sweet orange development stadium (6 of 6) citrus sinensis mrna |  |  |  |
| EY743868 | S44274544 | 7 | 21 | 6.393231 | 22.99307 | 1.846581985 | 0 | cs00-c3-705-104-a03- sweet orange development stadium (6 of 6) citrus sinensis mrna |  |  |  |
| EY743988 | S44274664 | 316 | 54 | 288.6087 | 59.12504 | -2.287273762 | 0 | transcription factor bhlh48 ame: full=transcription factor en 97 ame: full=bhlh transcription factor bhlh048 ame: full=basic helix-loop-helix protein 48� |  |  |  |
| EY744205 | S44274881 | 31 | 5 | 28.31288 | 5.47454 | -2.370648731 | 0 | gdsl esterase lipase at5g45670 ame: full=extracellular lipase at5g45670 flags: precursor |  |  |  |
| EY744415 | S44275091 | 1359 | 135 | 1241.2 | 147.8126 | -3.06989466 | 0 | anthocyanin 5-aromatic acyltransferase� |  |  |  |
| EY744610 | S44275286 | 182 | 31 | 166.224 | 33.94215 | -2.291978846 | 0 | protein |  |  |  |
| EY744652 | S44275328 | 1 | 13 | 0.913319 | 14.2338 | 3.962059202 | 0 | cs00-c5-003-010-a04- sweet orange greenhouse plant citrus sinensis mrna |  |  |  |
| EY744859 | S44275437 | 84 | 208 | 76.71877 | 227.7409 | 1.569741779 | 0 | conserved hypothetical protein [Ricinus communis] |  |  |  |
| EY744911 | S44275489 | 481 | 98 | 439.3063 | 107.301 | -2.033563756 | 0 | subtilisin-like protease ame: full=cucumisin-like serine protease flags: precursor |  |  |  |
| EY744925 | S44275503 | 3482 | 9525 | 3180.176 | 10429 | 1.713422374 | 0 | early nodulin-like protein 2 ame: full=phytocyanin-like protein flags: precursor |  |  |  |
| EY745020 | S44275598 | 5 | 18 | 4.566593 | 19.70835 | 2.109616391 | 0 | dna binding protein |  |  |  |
| EY745106 | S44275684 | 12875 | 3174 | 11758.98 | 3475.238 | -1.758578915 | 0 | probable aquaporin pip1-2 ame: full=plasma membrane intrinsic protein 1-2 ame: full= 1 2 |  |  |  |
| EY745157 | S44275735 | 504 | 128 | 460.3126 | 140.1482 | -1.715660439 | 0 | zinc finger protein constans-like 5 |  |  |  |
| EY745731 | S44276211 | 229 | 58 | 209.15 | 63.50467 | -1.719603309 | 0 | probable pectate lyase 5 flags: precursor |  |  |  |
| EY746754 | S44276669 | 7 | 27 | 6.393231 | 29.56252 | 2.209152064 | 0 | cs00-c5-003-037-c07- sweet orange greenhouse plant citrus sinensis mrna |  |  |  |
| EY746830 | S44276745 | 9764 | 2226 | 8917.644 | 2437.265 | -1.871399218 | 0 | heat shock 22 kda mitochondrial flags: precursor |  |  |  |
| EY746840 | S44276755 | 44 | 6 | 40.18602 | 6.569448 | -2.612849634 | 0 | invertase pectin methylesterase inhibitor family protein |  |  |  |
| EY746867 | S44276782 | 917 | 241 | 837.5132 | 263.8728 | -1.666269103 | 0 | solute carrier family 40 member 1 ame: full=ferroportin-1 ame: full=iron-regulated transporter 1 ame: full=metal transporter protein 1� |  |  |  |
| EY746896 | S44276811 | 6114 | 1469 | 5584.03 | 1608.42 | -1.795662672 | 0 | udp-glucuronate 4-epimerase 6 ame: full=udp-glucuronic acid epimerase 6� |  |  |  |
| EY746898 | S44276813 | 17 | 4 | 15.52642 | 4.379632 | -1.825843357 | 0 | transcription factor myb39 ame: full=myb-related protein 39� |  |  |  |
| EY746939 | S44276854 | 446 | 1720 | 407.3401 | 1883.242 | 2.208912434 | 0 | calcium-dependent protein kinase sk5� |  |  |  |
| EY747486 | S44277177 | 6 | 13 | 5.479912 | 14.2338 | 1.377096701 | 0 | cs00-c5-003-045-f04- sweet orange greenhouse plant citrus sinensis mrna |  |  |  |
| EY747508 | S44277199 | 2310 | 352 | 2109.766 | 385.4076 | -2.452626034 | 0 | at5g51550 k17n15\_10 |  |  |  |
| EY747509 | S44277200 | 6 | 58 | 5.479912 | 63.50467 | 3.534637978 | 0 | defensin-like protein 6 ame: full=plant defensin ame: full=low-molecular-weight cysteine-rich protein 74� |  |  |  |
| EY747562 | S44277253 | 46 | 8 | 42.01266 | 8.759265 | -2.261942472 | 0 | bifunctional enzyme lpxc fabz includes: ame: full=udp-3-o- dehydratase� |  |  |  |
| EY747590 | S44277281 | 3555 | 758 | 3246.848 | 829.9403 | -1.967960322 | 0 | expansin-a3� |  |  |  |
| EY747616 | S44277307 | 10 | 0 | 9.133187 | 0 | -Inf | 0 | cs00-c5-003-047-a06- sweet orange greenhouse plant citrus sinensis mrna |  |  |  |
| EY747662 | S44277353 | 2051 | 424 | 1873.217 | 464.241 | -2.012571838 | 0 | cell elongation protein diminuto |  |  |  |
| EY747704 | S44277395 | 114 | 23 | 104.1183 | 25.18289 | -2.047708574 | 0 | dna binding protein |  |  |  |
| EY748104 | S44277571 | 11 | 31 | 10.04651 | 33.94215 | 1.756384176 | 0 | cs00-c5-003-053-e07- sweet orange greenhouse plant citrus sinensis mrna |  |  |  |
| EY748162 | S44277629 | 115 | 23 | 105.0316 | 25.18289 | -2.060308611 | 0 | protein binding protein |  |  |  |
| EY748232 | S44277699 | 11 | 28 | 10.04651 | 30.65743 | 1.609542787 | 0 | chalcone synthase 5 ame: full=naringenin-chalcone synthase 5 |  |  |  |
| EY748896 | S44278027 | 36 | 91 | 32.87947 | 99.63663 | 1.599489123 | 0 | cs00-c5-003-063-g10- sweet orange greenhouse plant citrus sinensis mrna |  |  |  |
| EY749329 | S44278138 | 136 | 315 | 124.2113 | 344.896 | 1.473364661 | 0 | cs00-c5-003-072-f06- sweet orange greenhouse plant citrus sinensis mrna |  |  |  |
| EY749485 | S44278294 | 132 | 36 | 120.5581 | 39.41669 | -1.612849634 | 0 | novel plant |  |  |  |
| EY749556 | S44278365 | 4 | 18 | 3.653275 | 19.70835 | 2.431544486 | 0 | eh domain-containing protein 3 |  |  |  |
| EY749926 | S44278511 | 230 | 48 | 210.0633 | 52.55559 | -1.998908066 | 0 | auxin transporter protein 1 ame: full=auxin influx carrier protein 1 ame: full=polar auxin transport inhibitor-resistant protein 1 |  |  |  |
| EY749982 | S44278567 | 23354 | 5586 | 21329.64 | 6116.156 | -1.802162709 | 0 | s-adenosylmethionine synthetase 1� |  |  |  |
| EY750025 | S44278610 | 19 | 1 | 17.35306 | 1.094908 | -3.986308029 | 0 | 14 kda proline-rich protein flags: precursor |  |  |  |
| EY750256 | S44278841 | 36 | 106 | 32.87947 | 116.0603 | 1.819614937 | 0 | monoglyceride lipase� |  |  |  |
| EY750681 | S44278930 | 3 | 34 | 2.739956 | 37.22687 | 3.764119825 | 0 | sigma factor sigb regulation protein rsbq |  |  |  |
| EY750796 | S44279045 | 49 | 11 | 44.75262 | 12.04399 | -1.893658741 | 0 | somatic embryogenesis receptor kinase 2� |  |  |  |
| EY750798 | S44279047 | 625 | 93 | 570.8242 | 101.8265 | -2.486934084 | 0 | tubulin beta-8 chain ame: full=beta-8-tubulin |  |  |  |
| EY750803 | S44279052 | 5136 | 895 | 4690.805 | 979.9427 | -2.259066131 | 0 | l-ascorbate oxidase homolog flags: precursor |  |  |  |
| EY750814 | S44279063 | 15 | 1 | 13.69978 | 1.094908 | -3.645271112 | 0 | lrr receptor-like serine threonine-protein kinase rch1 ame: full=protein root clavata-homolog1 1 flags: precursor |  |  |  |
| EY750970 | S44279219 | 175 | 35 | 159.8308 | 38.32178 | -2.060308611 | 0 | cs00-c5-003-102-c07- sweet orange greenhouse plant citrus sinensis mrna |  |  |  |
| EY750982 | S44279231 | 272 | 18 | 248.4227 | 19.70835 | -3.655918356 | 0 | gdsl esterase lipase at3g27950 ame: full=extracellular lipase at3g27950 flags: precursor |  |  |  |
| EY751088 | S44279331 | 22 | 678 | 20.09301 | 742.3477 | 5.207329329 | 0 | non-specific lipid-transfer protein� |  |  |  |
| EY751117 | S44279360 | 3 | 11 | 2.739956 | 12.04399 | 2.136088602 | 0 | cs00-c5-003-103-h09- sweet orange greenhouse plant citrus sinensis mrna |  |  |  |
| EY751241 | S44279484 | 30 | 3 | 27.39956 | 3.284724 | -3.060308611 | 0 | uncharacterized protein at5g12080 |  |  |  |
| EY751345 | S44279588 | 71 | 159 | 64.84563 | 174.0904 | 1.42475532 | 0 | PREDICTED: hypothetical protein isoform 2 [Vitis vinifera] |  |  |  |
| EY751386 | S44279629 | 19 | 73 | 17.35306 | 79.92829 | 2.20351653 | 0 | homeobox-leucine zipper protein athb-40 ame: full=homeodomain transcription factor athb-40 ame: full=hd-zip protein athb-40 |  |  |  |
| EY751508 | S44279751 | 5 | 4 | 4.566593 | 4.379632 | -0.060308611 | 0 | mlp-like protein 423 |  |  |  |
| EY751767 | S44279898 | 36 | 128 | 32.87947 | 140.1482 | 2.091694483 | 0 | �cysteine-rich receptor-like protein kinase 20� |  |  |  |
| EY751774 | S44279905 | 13 | 1 | 11.87314 | 1.094908 | -3.438820234 | 0 | probable fatty acyl- reductase 5 |  |  |  |
| EY751795 | S44279926 | 752 | 1779 | 686.8157 | 1947.841 | 1.503881428 | 0 | cs00-c5-003-086-c02- sweet orange greenhouse plant citrus sinensis mrna |  |  |  |
| EY751835 | S44279966 | 25 | 1 | 22.83297 | 1.094908 | -4.382236706 | 0 | at3g16570 mgl6\_2 |  |  |  |
| EY751931 | S44280062 | 11222 | 2763 | 10249.26 | 3025.231 | -1.760402867 | 0 | subtilisin-like protease ame: full=cucumisin-like serine protease flags: precursor |  |  |  |
| EY752242 | S44280261 | 3 | 14 | 2.739956 | 15.32871 | 2.484011905 | 0 | photoassimilate-responsive protein par-like protein |  |  |  |
| EY752497 | S44280510 | 96 | 26 | 87.67859 | 28.46761 | -1.622903299 | 0 | 5ng4\_pintaauxin-induced protein 5ng4 |  |  |  |
| EY752852 | S44280781 | 456 | 141 | 416.4733 | 154.382 | -1.431719178 | 0 | anthranilate synthase component i- chloroplastic flags: precursor |  |  |  |
| EY752900 | S44280829 | 531 | 111 | 484.9722 | 121.5348 | -1.9965327 | 0 | protein |  |  |  |
| EY752992 | S44280921 | 176 | 23 | 160.7441 | 25.18289 | -2.674250179 | 0 | cellulase containing expressed |  |  |  |
| EY753208 | S44281025 | 168 | 401 | 153.4375 | 439.0581 | 1.516760488 | 0 | cs00-c5-003-083-b09- sweet orange greenhouse plant citrus sinensis mrna |  |  |  |
| EY753680 | S44281497 | 3 | 16 | 2.739956 | 17.51853 | 2.676656983 | 0 | retrovirus-related pol polyprotein from transposon tnt 1-94 includes: ame: full=protease includes: ame: full=reverse transcriptase includes: ame: full=endonuclease |  |  |  |
| EY753771 | S44281588 | 40 | 92 | 36.53275 | 100.7315 | 1.463253345 | 0 | protein kinase |  |  |  |
| EY753801 | S44281618 | 254 | 778 | 231.9829 | 851.8385 | 1.876561142 | 0 | protein |  |  |  |
| EY754241 | S44281834 | 12 | 25 | 10.95982 | 27.3727 | 1.320513173 | 0 | probable phytol kinase chloroplastic flags: precursor |  |  |  |
| EY754293 | S44281886 | 7 | 16 | 6.393231 | 17.51853 | 1.454264562 | 0 | dynein light chain cytoplasmic ame: full=8 kda dynein light chain |  |  |  |
| EY754945 | S44282230 | 770 | 1941 | 703.2554 | 2125.217 | 1.595489251 | 0 | PREDICTED: hypothetical protein [Vitis vinifera] |  |  |  |
| EY755529 | S44282366 | 311 | 62 | 284.0421 | 67.8843 | -2.064954976 | 0 | predicted protein [Populus trichocarpa] |  |  |  |
| EY755555 | S44282392 | 17 | 13 | 15.52642 | 14.2338 | -0.125403639 | 0 | probable lrr receptor-like serine threonine-protein kinase at3g47570 flags: precursor |  |  |  |
| EY755571 | S44282408 | 173 | 414 | 158.0041 | 453.2919 | 1.520478214 | 0 | ring finger protein 43 flags: precursor |  |  |  |
| EY756027 | S44282640 | 11 | 0 | 10.04651 | 0 | -Inf | 0 | cytochrome p450 86a1 ame: full=cyplxxxvi ame: full=p450-dependent fatty acid omega-hydroxylase |  |  |  |
| EY756570 | S44282959 | 693 | 3335 | 632.9298 | 3651.518 | 2.528378988 | 0 | dna binding |  |  |  |
| EY756862 | S44283139 | 44 | 2 | 40.18602 | 2.189816 | -4.197812135 | 0 | hypothetical protein [Vitis vinifera] |  |  |  |
| EY757359 | S44283412 | 100 | 25 | 91.33187 | 27.3727 | -1.738380516 | 0 | peptide transporter ptr1 |  |  |  |
| EY757951 | S44283780 | 42 | 105 | 38.35938 | 114.9653 | 1.583547579 | 0 | cs13-c1-001-029-e03- sweet orange field plant b citrus sinensis mrna |  |  |  |
| EY758109 | S44283938 | 954 | 155 | 871.306 | 169.7108 | -2.360101567 | 0 | monocopper oxidase-like protein sks1 flags: precursor |  |  |  |
| EY758114 | S44283943 | 408 | 1684 | 372.634 | 1843.825 | 2.306870565 | 0 | zinc finger protein constans-like 2 |  |  |  |
| EY650207 | S44284101 | 125 | 378 | 114.1648 | 413.8752 | 1.858077624 | 0 | iaa-amino acid hydrolase ilr1-like 6 flags: precursor |  |  |  |
| EY650724 | S44284184 | 11 | 1 | 10.04651 | 1.094908 | -3.197812135 | 0 | af506028\_20nbs-lrr type disease resistance protein |  |  |  |
| EY652026 | S44284688 | 326 | 37 | 297.7419 | 40.5116 | -2.877655305 | 0 | at3g19540 t31j18\_4 |  |  |  |
| EY652027 | S44284689 | 107 | 239 | 97.7251 | 261.683 | 1.421019306 | 0 | probable calcium-binding protein cml44 ame: full=calmodulin-like protein 44 |  |  |  |
| EY652293 | S44284731 | 7089 | 1809 | 6474.516 | 1980.689 | -1.708770238 | 0 | ferric reductase-like transmembrane component |  |  |  |
| EY653482 | S44285038 | 211 | 465 | 192.7102 | 509.1323 | 1.401607201 | 0 | f-box protein at1g30200 |  |  |  |
| EY655127 | S44285423 | 68 | 6 | 62.10567 | 6.569448 | -3.240880856 | 0 | glucan endo- -beta-glucosidase 1 ame: full=(1- |  |  |  |
| EY655130 | S44285426 | 66 | 16 | 60.27903 | 17.51853 | -1.782774635 | 0 | fasciclin-like arabinogalactan protein 6 flags: precursor |  |  |  |
| EY655144 | S44285440 | 15 | 2 | 13.69978 | 2.189816 | -2.645271112 | 0 | protein ycf2 |  |  |  |
| EY656901 | S44285895 | 14 | 3 | 12.78646 | 3.284724 | -1.960772937 | 0 | calcium-transporting atpase chloroplastic ame: full=ca(2+)-atpase isoform 1 ame: full=plastid envelope atpase 1 flags: precursor |  |  |  |
| EY656912 | S44285906 | 38 | 4 | 34.70611 | 4.379632 | -2.986308029 | 0 | probable lrr receptor-like serine threonine-protein kinase at1g63430 flags: precursor |  |  |  |
| EY656977 | S44285971 | 4 | 12 | 3.653275 | 13.1389 | 1.846581985 | 0 | cs00-c1-100-060-f12- sweet orange greenhouse plant citrus sinensis mrna |  |  |  |
| EY657105 | S44286001 | 10 | 2 | 9.133187 | 2.189816 | -2.060308611 | 0 | nucleic acid binding |  |  |  |
| EY658297 | S44286297 | 30 | 0 | 27.39956 | 0 | -Inf | 0 | acyl synthetase |  |  |  |
| EY658417 | S44286319 | 29 | 4 | 26.48624 | 4.379632 | -2.596361511 | 0 | serine carboxypeptidase-like 45 flags: precursor |  |  |  |
| EY658938 | S44286546 | 188 | 49 | 171.7039 | 53.6505 | -1.678259523 | 0 | probable lrr receptor-like serine threonine-protein kinase at1g56130 flags: precursor |  |  |  |
| EY659009 | S44286617 | 45 | 12 | 41.09934 | 13.1389 | -1.645271112 | 0 | protein |  |  |  |
| EY659366 | S44286666 | 62 | 11 | 56.62576 | 12.04399 | -2.233145208 | 0 | conserved hypothetical protein [Ricinus communis] |  |  |  |
| EY659391 | S44286691 | 13 | 42 | 11.87314 | 45.98614 | 1.953497189 | 0 | uncharacterized udp-glucosyltransferase at1g05670 |  |  |  |
| EY659413 | S44286713 | 143 | 339 | 130.6046 | 371.1738 | 1.50688961 | 0 | yth domain family protein 1 ame: full=dermatomyositis associated with cancer autoantigen 1 homolog� |  |  |  |
| EY660034 | S44286788 | 7 | 19 | 6.393231 | 20.80325 | 1.702192075 | 0 | uncharacterized mitochondrial protein g00310 ame: full=orf154 |  |  |  |
| EY660781 | S44286877 | 137 | 49 | 125.1247 | 53.6505 | -1.221702755 | 0 | cs00-c1-101-025-h08- sweet orange infected with xylella fastidiosa (stage 1 of 2) citrus sinensis mrna |  |  |  |
| EY661135 | S44287133 | 46 | 0 | 42.01266 | 0 | -Inf | 0 | fasciclin-like arabinogalactan protein 11 flags: precursor |  |  |  |
| EY661193 | S44287191 | 19 | 2 | 17.35306 | 2.189816 | -2.986308029 | 0 | cellulose synthase a catalytic subunit 7� |  |  |  |
| EY661436 | S44287210 | 1715 | 922 | 1566.342 | 1009.505 | -0.633750437 | 0 | protein |  |  |  |
| EY662249 | S44287575 | 183 | 29 | 167.1373 | 31.75233 | -2.396099359 | 0 | cs00-c1-101-042-a12- sweet orange infected with xylella fastidiosa (stage 1 of 2) citrus sinensis mrna |  |  |  |
| EY662635 | S44287751 | 26 | 4 | 23.74629 | 4.379632 | -2.438820234 | 0 | upf0497 membrane protein at4g25830 |  |  |  |
| EY663158 | S44288050 | 10 | 30 | 9.133187 | 32.84724 | 1.846581985 | 0 | protein |  |  |  |
| EY664186 | S44288420 | 207 | 48 | 189.057 | 52.55559 | -1.846904973 | 0 | conserved hypothetical protein [Ricinus communis] |  |  |  |
| EY664779 | S44288565 | 10 | 33 | 9.133187 | 36.13197 | 1.984085509 | 0 | cs00-c1-101-070-d02- sweet orange infected with xylella fastidiosa (stage 1 of 2) citrus sinensis mrna |  |  |  |
| EY664801 | S44288587 | 261 | 43 | 238.3762 | 47.08105 | -2.340021758 | 0 | protein |  |  |  |
| EY664805 | S44288591 | 88 | 377 | 80.37204 | 412.7803 | 2.360608579 | 0 | probable wrky transcription factor 70 ame: full=wrky dna-binding protein 70 |  |  |  |
| EY664808 | S44288594 | 17 | 71 | 15.52642 | 77.73847 | 2.323903762 | 0 | cs00-c1-101-070-f11- sweet orange infected with xylella fastidiosa (stage 1 of 2) citrus sinensis mrna |  |  |  |
| EY664847 | S44288633 | 3 | 17 | 2.739956 | 18.61344 | 2.764119825 | 0 | cs00-c1-101-071-b07- sweet orange infected with xylella fastidiosa (stage 1 of 2) citrus sinensis mrna |  |  |  |
| EY669455 | S44289657 | 1851 | 67 | 1690.553 | 73.35884 | -4.526380505 | 0 | ethylene-responsive transcription factor erf107 |  |  |  |
| EY669560 | S44289762 | 477 | 1070 | 435.653 | 1171.552 | 1.427169109 | 0 | protein |  |  |  |
| EY669659 | S44289861 | 1697 | 3811 | 1549.902 | 4172.695 | 1.428802527 | 0 | cbl-interacting serine threonine-protein kinase 12 ame: full=sos2-like protein kinase pks8 ame: full=snf1-related kinase |  |  |  |
| EY670109 | S44289975 | 26 | 53 | 23.74629 | 58.03013 | 1.289100221 | 0 | probable lrr receptor-like serine threonine-protein kinase at1g53420 flags: precursor |  |  |  |
| EY671577 | S44290547 | 131 | 96 | 119.6447 | 105.1112 | -0.186841017 | 0 | pentatricopeptide repeat-containing protein mitochondrial flags: precursor |  |  |  |
| EY672758 | S44290944 | 144 | 346 | 131.5179 | 378.8382 | 1.52632271 | 0 | cs00-c1-102-108-a01- sweet orange infected with xylella fastidiosa (stage 2 of 2) citrus sinensis mrna |  |  |  |
| EY672837 | S44291023 | 153 | 892 | 139.7378 | 976.658 | 2.805131541 | 0 | tyrosine n-monooxygenase ame: full=cytochrome p450tyr ame: full=cytochrome p450 79a1 |  |  |  |
| EY672999 | S44291185 | 436 | 126 | 398.2069 | 137.9584 | -1.529284917 | 0 | caffeic acid 3-o-methyltransferase ame: full=s-adenosysl-l-methionine:caffeic acid 3-o-methyltransferase� |  |  |  |
| EY673657 | S44291409 | 12 | 3 | 10.95982 | 3.284724 | -1.738380516 | 0 | cs00-c1-102-012-f06- sweet orange infected with xylella fastidiosa (stage 2 of 2) citrus sinensis mrna |  |  |  |
| EY674028 | S44291654 | 5664 | 683 | 5173.037 | 747.8222 | -2.790244298 | 0 | phosphoenolpyruvate carboxykinase� |  |  |  |
| EY674035 | S44291661 | 4 | 121 | 3.653275 | 132.4839 | 5.180482721 | 0 | transcriptional xre family |  |  |  |
| EY674080 | S44291706 | 10 | 0 | 9.133187 | 0 | -Inf | 0 | PREDICTED: hypothetical protein [Vitis vinifera] |  |  |  |
| EY674763 | S44291829 | 59 | 1 | 53.8858 | 1.094908 | -5.621023565 | 0 | gdsl esterase lipase at4g28780 ame: full=extracellular lipase at4g28780 flags: precursor |  |  |  |
| EY674818 | S44291870 | 20 | 5 | 18.26637 | 5.47454 | -1.738380516 | 0 | acyl synthetase |  |  |  |
| EY674821 | S44291873 | 57 | 1 | 52.05917 | 1.094908 | -5.57127053 | 0 | briggsae cbr-abt-1 protein |  |  |  |
| EY676418 | S44292028 | 916 | 2584 | 836.5999 | 2829.242 | 1.757806051 | 0 | conserved hypothetical protein [Ricinus communis] |  |  |  |
| EY676841 | S44292115 | 31 | 3 | 28.31288 | 3.284724 | -3.107614326 | 0 | protein |  |  |  |
| EY676843 | S44292117 | 903 | 2370 | 824.7268 | 2594.932 | 1.65370865 | 0 | protein |  |  |  |
| EY676956 | S44292230 | 542 | 65 | 495.0187 | 71.16902 | -2.798161744 | 0 | geranylgeranyl pyrophosphate chloroplastic chromoplastic� |  |  |  |
| EY677000 | S44292274 | 4885 | 11804 | 4461.562 | 12924.29 | 1.534464843 | 0 | zinc finger ccch domain-containing protein 29� |  |  |  |
| EY677004 | S44292278 | 213 | 1366 | 194.5369 | 1495.644 | 2.942651632 | 0 | ethylene-responsive transcription factor 5� |  |  |  |
| EY677013 | S44292287 | 9 | 1 | 8.219868 | 1.094908 | -2.908305517 | 0 | indole-3-glycerol phosphate synthase� |  |  |  |
| EY677460 | S44292300 | 17 | 3 | 15.52642 | 3.284724 | -2.240880856 | 0 | cs00-c1-401-038-a02- sweet orange infected with citrus sinensis mrna |  |  |  |
| EY677554 | S44292394 | 2607 | 597 | 2381.022 | 653.6601 | -1.864968262 | 0 | protein e6 |  |  |  |
| EY678272 | S44292454 | 603 | 60 | 550.7312 | 65.69448 | -3.067504112 | 0 | fasciclin-like arabinogalactan protein 2 flags: precursor |  |  |  |
| EY681751 | S44293245 | 13 | 35 | 11.87314 | 38.32178 | 1.690462783 | 0 | non-specific lipid-transfer protein 3� |  |  |  |
| EY681786 | S44293280 | 14 | 39 | 12.78646 | 42.70141 | 1.739666781 | 0 | PREDICTED: hypothetical protein [Vitis vinifera] |  |  |  |
| EY683346 | S44293860 | 11 | 34 | 10.04651 | 37.22687 | 1.889650707 | 0 | cs00-c1-650-049-a08- sweet orange young greenhouse plant citrus sinensis mrna |  |  |  |
| EY684017 | S44294083 | 2016 | 366 | 1841.25 | 400.7364 | -2.199960601 | 0 | tsd2 (tumorous shoot development 2) methyltransferase |  |  |  |
| EY684150 | S44294216 | 849 | 151 | 775.4076 | 165.3311 | -2.22959652 | 0 | upf0497 membrane protein 11 |  |  |  |
| EY684201 | S44294267 | 411 | 96 | 375.374 | 105.1112 | -1.836412599 | 0 | �domain-containing gpi-anchored protein 2 flags: precursor |  |  |  |
| EY686933 | S44294849 | 349 | 232 | 318.7482 | 254.0187 | -0.327482747 | 0 | cs00-c2-003-030-h08- sweet orange greenhouse plant citrus sinensis mrna |  |  |  |
| EY687709 | S44295149 | 3187 | 4967 | 2910.747 | 5438.408 | 0.901795212 | 0 | corylus avellana clone kg871 microsatellite sequence |  |  |  |
| EY687966 | S44295182 | 72 | 19 | 65.75895 | 20.80325 | -1.660378004 | 0 | fasciclin-like arabinogalactan protein 10 flags: precursor |  |  |  |
| EY688356 | S44295348 | 201 | 26 | 183.5771 | 28.46761 | -2.688992489 | 0 | 5ng4\_pintaauxin-induced protein 5ng4 |  |  |  |
| EY688477 | S44295469 | 69 | 9 | 63.01899 | 9.854173 | -2.676979971 | 0 | unknown [Glycine max] |  |  |  |
| EY688559 | S44295551 | 32 | 79 | 29.2262 | 86.49774 | 1.565400232 | 0 | cs00-c2-003-089-e04- sweet orange greenhouse plant citrus sinensis mrna |  |  |  |
| EY690065 | S44295811 | 58 | 5 | 52.97248 | 5.47454 | -3.274433416 | 0 | cs00-c2-003-083-h12- sweet orange greenhouse plant citrus sinensis mrna |  |  |  |
| EY690095 | S44295841 | 122 | 186 | 111.4249 | 203.6529 | 0.870040958 | 0 | cs00-c2-003-057-d02- sweet orange greenhouse plant citrus sinensis mrna |  |  |  |
| EY690363 | S44295997 | 461 | 61 | 421.0399 | 66.78939 | -2.656266119 | 0 | reticuline oxidase-like protein flags: precursor |  |  |  |
| EY691630 | S44296046 | 95 | 5 | 86.76528 | 5.47454 | -3.986308029 | 0 | ubiquitin carboxyl-terminal hydrolase 12 ame: full=ubiquitin thioesterase 12 ame: full=ubiquitin-specific-processing protease 12 ame: full=deubiquitinating enzyme 12� |  |  |  |
| EY691688 | S44296104 | 381 | 881 | 347.9744 | 964.614 | 1.470970506 | 0 | protein |  |  |  |
| EY691718 | S44296134 | 123 | 13 | 112.3382 | 14.2338 | -2.980455303 | 0 | endochitinase 1 flags: precursor |  |  |  |
| EY691729 | S44296145 | 170 | 513 | 155.2642 | 561.6878 | 1.855043564 | 0 | protein kinase g11a |  |  |  |
| EY691847 | S44296263 | 45 | 10 | 41.09934 | 10.94908 | -1.908305517 | 0 | endoglucanase 11 ame: full=endo- -beta glucanase 11 flags: precursor |  |  |  |
| EY691883 | S44296299 | 31 | 0 | 28.31288 | 0 | -Inf | 0 | (+)-delta-cadinene synthase isozyme xc14� |  |  |  |
| EY693354 | S44296986 | 1721 | 2744 | 1571.821 | 3004.428 | 0.934652868 | 0 | protein |  |  |  |
| EY694888 | S44297386 | 172 | 1944 | 157.0908 | 2128.501 | 3.760167233 | 0 | 18 kda seed maturation protein |  |  |  |
| EY698173 | S44298123 | 4247 | 3005 | 3878.864 | 3290.199 | -0.237459634 | 0 | ethylene-responsive transcription factor erf003 |  |  |  |
| EY698263 | S44298213 | 12 | 2 | 10.95982 | 2.189816 | -2.323343017 | 0 | myrcene chloroplastic flags: precursor |  |  |  |
| EY699267 | S44298335 | 16 | 38 | 14.6131 | 41.60651 | 1.509546998 | 0 | protein |  |  |  |
| EY701011 | S44298637 | 453 | 102 | 413.7334 | 111.6806 | -1.889322414 | 0 | subtilisin-like protease ame: full=cucumisin-like serine protease flags: precursor |  |  |  |
| EY702425 | S44298721 | 1273 | 3520 | 1162.655 | 3854.076 | 1.728962494 | 0 | protein |  |  |  |
| EY702934 | S44298894 | 56 | 14 | 51.14585 | 15.32871 | -1.738380516 | 0 | protein |  |  |  |
| EY703299 | S44298923 | 234 | 515 | 213.7166 | 563.8777 | 1.399683387 | 0 | ethylene-responsive transcription factor 3 ame: full=ethylene-responsive element-binding factor 3 homolog ame: full=ethylene-responsive element-binding factor 5� |  |  |  |
| EY704562 | S44299178 | 39 | 0 | 35.61943 | 0 | -Inf | 0 | protein yippee-like at4g27745 |  |  |  |
| EY705333 | S44299277 | 1212 | 276 | 1106.942 | 302.1946 | -1.873030043 | 0 | transcription factor spatula ame: full=transcription factor en 99 ame: full=bhlh transcription factor bhlh024 ame: full=basic helix-loop-helix protein 24� |  |  |  |
| EY709546 | S44300061 | 21 | 5 | 19.17969 | 5.47454 | -1.808769844 | 0 | germin-like protein subfamily 1 member 8 flags: precursor |  |  |  |
| EY710217 | S44300172 | 2757 | 559 | 2518.02 | 612.0536 | -2.040559595 | 0 | transcription factor bhlh113 ame: full=transcription factor en 61 ame: full=bhlh transcription factor bhlh113 ame: full=basic helix-loop-helix protein 113� |  |  |  |
| EY710254 | S44300209 | 2025 | 422 | 1849.47 | 462.0512 | -2.00098752 | 0 | cytochrome p450 71d7 |  |  |  |
| EY710274 | S44300229 | 4046 | 329 | 3695.287 | 360.2248 | -3.358717347 | 0 | expansin-a5� |  |  |  |
| EY710289 | S44300244 | 2173 | 587 | 1984.642 | 642.711 | -1.626636282 | 0 | bahd acyltransferase at5g47980 |  |  |  |
| EY710321 | S44300276 | 463 | 88 | 422.8666 | 96.35191 | -2.133817281 | 0 | cytochrome p450 82a4 ame: full=p450 cp9 |  |  |  |
| EY710329 | S44300284 | 431 | 943 | 393.6404 | 1032.498 | 1.391189386 | 0 | wrky transcription factor 6 ame: full=wrky dna-binding protein 6� |  |  |  |
| EY710358 | S44300313 | 3209 | 10626 | 2930.84 | 11634.49 | 1.989022407 | 0 | squidulin ame: full=optic lobe calcium-binding protein ame: full=scabp |  |  |  |
| EY710369 | S44300324 | 1184 | 3843 | 1081.369 | 4207.732 | 1.96018338 | 0 | cs00-c3-701-107-b01- sweet orange development stadium (2 of 6) citrus sinensis mrna |  |  |  |
| EY710382 | S44300337 | 777 | 132 | 709.6486 | 144.5279 | -2.295757185 | 0 | miraculin-like protein 2 |  |  |  |
| EY710727 | S44300346 | 4718 | 759 | 4309.038 | 831.0352 | -2.374384144 | 0 | 14 kda proline-rich protein flags: precursor |  |  |  |
| EY710758 | S44300377 | 418 | 97 | 381.7672 | 106.2061 | -1.845826806 | 0 | 3-ketoacyl- synthase 21� |  |  |  |
| EY711870 | S44300817 | 33 | 5 | 30.13952 | 5.47454 | -2.46084654 | 0 | btb poz domain-containing protein at1g30440 |  |  |  |
| EY713160 | S44301211 | 17 | 2 | 15.52642 | 2.189816 | -2.825843357 | 0 | btb poz domain-containing protein at5g48800 |  |  |  |
| EY713165 | S44301216 | 2 | 11 | 1.826637 | 12.04399 | 2.721051103 | 0 | cs00-c3-702-027-g11- sweet orange development stadium (3 of 6) citrus sinensis mrna |  |  |  |
| EY713683 | S44301510 | 285 | 69 | 260.2958 | 75.54866 | -1.784674168 | 0 | vinorine synthase |  |  |  |
| EY714918 | S44302073 | 133 | 11 | 121.4714 | 12.04399 | -3.334231333 | 0 | beta-galactosidase 10� |  |  |  |
| EY716408 | S44302779 | 94 | 23 | 85.85196 | 25.18289 | -1.769407412 | 0 | cs00-c3-702-067-b04- sweet orange development stadium (3 of 6) citrus sinensis mrna |  |  |  |
| EY717550 | S44303165 | 301 | 826 | 274.9089 | 904.3941 | 1.717997779 | 0 | cs00-c3-702-080-f08- sweet orange development stadium (3 of 6) citrus sinensis mrna |  |  |  |
| EY717978 | S44303355 | 9 | 25 | 8.219868 | 27.3727 | 1.735550672 | 0 | cs00-c3-702-086-h11- sweet orange development stadium (3 of 6) citrus sinensis mrna |  |  |  |
| EY726580 | S44305496 | 869 | 2346 | 793.6739 | 2568.654 | 1.694394415 | 0 | ethylene-responsive transcription factor rap2-3 ame: full=protein related to apetala2 3� |  |  |  |
| EY727621 | S44305879 | 125150 | 25970 | 114301.8 | 28434.76 | -2.007120888 | 0 | cs00-c3-703-103-e10- sweet orange development stadium (4 of 6) citrus sinensis mrna |  |  |  |
| EY727625 | S44305883 | 322 | 31 | 294.0886 | 33.94215 | -3.115101084 | 0 | pollen-specific protein sf3 |  |  |  |
| EY727894 | S44306152 | 1576 | 402 | 1439.39 | 440.153 | -1.709380644 | 0 | esterase lipase thioesterase family protein |  |  |  |
| EY727895 | S44306153 | 79 | 216 | 72.15218 | 236.5001 | 1.712726238 | 0 | plastidic atp adp-transporter |  |  |  |
| EY729741 | S44306445 | 13 | 3 | 11.87314 | 3.284724 | -1.853857733 | 0 | cs00-c3-704-020-g10- sweet orange development stadium (5 of 6) citrus sinensis mrna |  |  |  |
| EY730799 | S44306845 | 21 | 47 | 19.17969 | 51.46068 | 1.423890913 | 0 | protein binding |  |  |  |
| EY731098 | S44307032 | 13 | 3 | 11.87314 | 3.284724 | -1.853857733 | 0 | 2-c-methyl-d-erythritol 4-phosphate cytidylyltransferase ame: full=4-diphosphocytidyl-2c-methyl-d-erythritol synthase ame: full=mep cytidylyltransferase� |  |  |  |
| EY731155 | S44307089 | 38 | 230 | 34.70611 | 251.8289 | 2.859182022 | 0 | u-box domain-containing protein 32 ame: full=plant u-box protein 32 |  |  |  |
| EY732215 | S44307141 | 325 | 781 | 296.8286 | 855.1232 | 1.526502314 | 0 | unknown protein [Arabidopsis thaliana] |  |  |  |
| EY735029 | S44307707 | 21 | 58 | 19.17969 | 63.50467 | 1.727283056 | 0 | cs00-c3-704-075-e05- sweet orange development stadium (5 of 6) citrus sinensis mrna |  |  |  |
| EY735290 | S44307856 | 1175 | 275 | 1073.149 | 301.0997 | -1.833537749 | 0 | abc transporter g family member 15� |  |  |  |
| EY735307 | S44307873 | 87 | 9 | 79.45873 | 9.854173 | -3.01139901 | 0 | cs00-c3-704-093-f03- sweet orange development stadium (5 of 6) citrus sinensis mrna |  |  |  |
| EY736996 | S44308344 | 93 | 24 | 84.93864 | 26.27779 | -1.692576826 | 0 | monogalactosyldiacylglycerol synthase chloroplastic� |  |  |  |
| EY741616 | S44309316 | 65 | 10 | 59.36571 | 10.94908 | -2.438820234 | 0 | acyltransferase-like protein chloroplastic flags: precursor |  |  |  |
| EY742619 | S44309759 | 9 | 22 | 8.219868 | 24.08798 | 1.551126101 | 0 | human dna sequence from clone xxyac-68g7 on chromosome 9 contains the 3 end of the secisbp2 gene for secis binding protein the gene for a novel protein and the 3 end of the sema4d gene for sema immunoglobulin domain transmembrane domain and short cytoplasmic complete sequence |  |  |  |
| EY743349 | S44310041 | 896 | 170 | 818.3335 | 186.1344 | -2.136344502 | 0 | ferric reductase-like transmembrane component |  |  |  |
| EY743725 | S44310417 | 290 | 64 | 264.8624 | 70.07412 | -1.918289606 | 0 | chavicol o-methyltransferase ame: full= eugenol o-methyltransferase cvomt1 ame: full=s-adenosysl-l-methionine: eugenol o-methyltransferase cvomt1 |  |  |  |
| EY743805 | S44310497 | 6392 | 1069 | 5837.933 | 1170.457 | -2.318386071 | 0 | expansin-a10� |  |  |  |
| EY744761 | S44310557 | 1420 | 3145 | 1296.913 | 3443.486 | 1.408788571 | 0 | protein |  |  |  |
| EY744773 | S44310569 | 20 | 5 | 18.26637 | 5.47454 | -1.738380516 | 0 | predicted protein [Populus trichocarpa] |  |  |  |
| EY744809 | S44310605 | 773 | 1802 | 705.9953 | 1973.024 | 1.482678176 | 0 | floral homeotic protein apetala 2 |  |  |  |
| EY745561 | S44310685 | 82 | 680 | 74.89213 | 744.5375 | 3.313458416 | 0 | heat stress transcription factor c-1� |  |  |  |
| EY746025 | S44310818 | 215 | 52 | 196.3635 | 56.93522 | -1.786133647 | 0 | 4-coumarate-- ligase 1� |  |  |  |
| EY746274 | S44310857 | 9 | 0 | 8.219868 | 0 | -Inf | 0 | cs00-c5-003-030-a04- sweet orange greenhouse plant citrus sinensis mrna |  |  |  |
| EY746318 | S44310901 | 1222 | 108 | 1116.075 | 118.2501 | -3.238521584 | 0 | protein |  |  |  |
| EY746373 | S44310956 | 0 | 21 | 0 | 22.99307 | Inf | 0 | protein |  |  |  |
| EY746433 | S44311016 | 99 | 278 | 90.41855 | 304.3844 | 1.751203937 | 0 | protein tify 5a ame: full=jasmonate zim domain-containing protein 8 |  |  |  |
| EY746603 | S44311186 | 159 | 551 | 145.2177 | 603.2943 | 2.054645037 | 0 | acyl- chloroplastic ame: full=stearoyl-acp desaturase flags: precursor |  |  |  |
| EY746611 | S44311194 | 69 | 15 | 63.01899 | 16.42362 | -1.940014377 | 0 | 21 kda protein ame: full= protein flags: precursor |  |  |  |
| EY746650 | S44311233 | 1109 | 103 | 1012.87 | 112.7755 | -3.166923639 | 0 | pectinesterase pectinesterase inhibitor ppe8b includes: ame: full=pectinesterase inhibitor ppe8b ame: full=pectin methylesterase inhibitor ppe8b includes: ame: full=pectinesterase ppe8b� |  |  |  |
| EY746671 | S44311254 | 62 | 8 | 56.62576 | 8.759265 | -2.692576826 | 0 | beta-galactosidase 5� |  |  |  |
| EY747117 | S44311364 | 151 | 419 | 137.9111 | 458.7665 | 1.734021178 | 0 | non-specific lipid-transfer protein� |  |  |  |
| EY747267 | S44311514 | 333 | 67 | 304.1351 | 73.35884 | -2.051669693 | 0 | snakin-2 flags: precursor |  |  |  |
| EY747838 | S44311651 | 136 | 518 | 124.2113 | 567.1624 | 2.190964931 | 0 | at4g37300 c7a10\_60 |  |  |  |
| EY747865 | S44311678 | 147 | 320 | 134.2578 | 350.3706 | 1.383875234 | 0 | at1g70780 f5a18\_4 |  |  |  |
| EY747873 | S44311686 | 10 | 1 | 9.133187 | 1.094908 | -3.060308611 | 0 | cs00-c5-003-049-h03- sweet orange greenhouse plant citrus sinensis mrna |  |  |  |
| EY748285 | S44311762 | 50 | 4 | 45.66593 | 4.379632 | -3.382236706 | 0 | gdsl esterase lipase exl3 ame: full=family ii extracellular lipase 3� |  |  |  |
| EY749118 | S44312245 | 27 | 6 | 24.6596 | 6.569448 | -1.908305517 | 0 | probable xyloglucan endotransglucosylase hydrolase protein b ame: full= 2 flags: precursor |  |  |  |
| EY749122 | S44312249 | 16 | 4 | 14.6131 | 4.379632 | -1.738380516 | 0 | early nodulin-like protein 1 ame: full=phytocyanin-like protein flags: precursor |  |  |  |
| EY749788 | S44312579 | 91 | 20 | 83.112 | 21.89816 | -1.924247061 | 0 | unknown [Glycine max] |  |  |  |
| EY749834 | S44312625 | 12 | 2 | 10.95982 | 2.189816 | -2.323343017 | 0 | cs00-c5-003-080-d04- sweet orange greenhouse plant citrus sinensis mrna |  |  |  |
| EY750476 | S44312819 | 19 | 47 | 17.35306 | 51.46068 | 1.568280822 | 0 | indole-3-acetic acid-amido synthetase ame: full=auxin-responsive gh3-like protein 3� |  |  |  |
| EY750529 | S44312872 | 29 | 6 | 26.48624 | 6.569448 | -2.01139901 | 0 | �pectate lyase 19 flags: precursor |  |  |  |
| EY750585 | S44312928 | 60 | 9 | 54.79912 | 9.854173 | -2.47534611 | 0 | phenylalanine ammonia-lyase |  |  |  |
| EY752069 | S44313102 | 48 | 1309 | 43.8393 | 1433.235 | 5.030906365 | 0 | cs00-c5-003-092-e05- sweet orange greenhouse plant citrus sinensis mrna |  |  |  |
| EY752079 | S44313112 | 243 | 939 | 221.9364 | 1028.119 | 2.211788328 | 0 | transcription factor bhlh148 ame: full=transcription factor en 143 ame: full=bhlh transcription factor bhlh148 ame: full=basic helix-loop-helix protein 148� |  |  |  |
| EY752668 | S44313245 | 303 | 1350 | 276.7356 | 1478.126 | 2.417189193 | 0 | iaa-amino acid hydrolase ilr1-like 6 ame: full=protein gr1 flags: precursor |  |  |  |
| EY753079 | S44313320 | 31 | 7 | 28.31288 | 7.664356 | -1.885221904 | 0 | protein |  |  |  |
| EY753091 | S44313332 | 2280 | 5930 | 2082.367 | 6492.805 | 1.640617764 | 0 | cs00-c5-003-050-h09- sweet orange greenhouse plant citrus sinensis mrna |  |  |  |
| EY754662 | S44313783 | 228 | 433 | 208.2367 | 474.0952 | 1.186952685 | 0 | protein twin lov 1 |  |  |  |
| EY754720 | S44313841 | 20 | 55 | 18.26637 | 60.21994 | 1.721051103 | 0 | cs12-c1-001-019-c10- sweet orange field plant a citrus sinensis mrna |  |  |  |
| EY754766 | S44313887 | 277 | 468 | 252.9893 | 512.417 | 1.018242038 | 0 | cs12-c1-001-019-g10- sweet orange field plant a citrus sinensis mrna |  |  |  |
| EY754792 | S44313913 | 36 | 8 | 32.87947 | 8.759265 | -1.908305517 | 0 | wall-associated receptor kinase 5 flags: precursor |  |  |  |
| EY756177 | S44314738 | 55 | 13 | 50.23253 | 14.2338 | -1.819300511 | 0 | probable lrr receptor-like serine threonine-protein kinase at1g12460 flags: precursor |  |  |  |
| EY756692 | S44314917 | 96 | 218 | 87.67859 | 238.69 | 1.444841308 | 0 | histone ame: full=histone |  |  |  |
| EY756705 | S44314930 | 247 | 1007 | 225.5897 | 1102.572 | 2.289100221 | 0 | uncharacterized n-acetyltransferase p20 |  |  |  |
| EY757666 | S44315233 | 151 | 39 | 137.9111 | 42.70141 | -1.691383036 | 0 | nucleobase-ascorbate transporter 6� |  |  |  |
| EY757863 | S44315318 | 14 | 30 | 12.78646 | 32.84724 | 1.361155158 | 0 | serine carboxypeptidase-like 42 flags: precursor |  |  |  |
| FE659262 | S46102851 | 8 | 19 | 7.306549 | 20.80325 | 1.509546998 | 0 | 347 hong anliu sweet orange ssh library citrus sinensis cdna 5 mrna |  |  |  |
| DC887374 | S47736050 | 6 | 17 | 5.479912 | 18.61344 | 1.764119825 | 0 | dc887374 eic citrus sinensis cdna clone eic0284 5 mrna |  |  |  |
| DC899996 | S47736293 | 273 | 39 | 249.336 | 42.70141 | -2.545735438 | 0 | gdsl esterase lipase apg ame: full=extracellular lipase apg flags: precursor |  |  |  |
| DC900189 | S47736487 | 3 | 10 | 2.739956 | 10.94908 | 1.998585078 | 0 | non-specific lipid-transfer protein 2� |  |  |  |
| DC900219 | S47736517 | 57256 | 6383 | 52292.97 | 6988.798 | -2.903500848 | 0 | peroxidase 42� |  |  |  |
| FC871208 | S49954862 | 451 | 272 | 411.9067 | 297.815 | -0.467901298 | 0 | kn0aak3dd01fm1 ruit1 citrus sinensis cdna clone mrna |  |  |  |
| FC871426 | S49955080 | 459 | 2006 | 419.2133 | 2196.386 | 2.389375031 | 0 | dicyanin blue copper protein precursor |  |  |  |
| FC871628 | S49955282 | 11 | 25 | 10.04651 | 27.3727 | 1.446044055 | 0 | kn0aak1bg04fm2 ruit1 citrus sinensis cdna clone mrna |  |  |  |
| FC871875 | S49955529 | 84 | 20 | 76.71877 | 21.89816 | -1.808769844 | 0 | serine-threonine protein plant- |  |  |  |
| FC921914 | S49955742 | 16 | 40 | 14.6131 | 43.79632 | 1.583547579 | 0 | bahd acyltransferase at5g47980 |  |  |  |
| FC922215 | S49956043 | 1749 | 3953 | 1597.394 | 4328.172 | 1.43803715 | 0 | af211539\_1avr9 cf-9 rapidly elicited protein 65 |  |  |  |
| FC922338 | S49956166 | 112 | 294 | 102.2917 | 321.903 | 1.653936907 | 0 | disease resistance response protein 206 |  |  |  |
| FC922373 | S49956201 | 2 | 11 | 1.826637 | 12.04399 | 2.721051103 | 0 | poncirus trifoliata citrus tristeza virus resistance gene complete sequence |  |  |  |
| FC922537 | S49956365 | 0 | 1 | 0 | 1.094908 | Inf | 0 | lotus japonicus genomic chromosome clone: complete sequence |  |  |  |
| FC922608 | S49956436 | 28 | 522 | 25.57292 | 571.542 | 4.482170559 | 0 | bap2 (bon association protein 2) |  |  |  |
| FC922613 | S49956441 | 100 | 16 | 91.33187 | 17.51853 | -2.382236706 | 0 | predicted protein [Populus trichocarpa] |  |  |  |
| FC922972 | S49956800 | 269 | 598 | 245.6827 | 654.755 | 1.414158796 | 0 | nacl-inducible calcium |  |  |  |
|  |  |  |  |  |  |  |  |  |  |  |  |
