# Supplementary material for: Transcriptome Profiling of Citrus Fruit Response to Huanglongbing Disease
Source: PLoS One. 2012 May 31;7(5):e38039. doi: 10.1371/journal.pone.0038039 (PMC3364978; doi:10.1371/journal.pone.0038039)
Supplement: Table S3 — Differentially expressed genes in apparently healthy fruit in comparison to control (healthy in disease-free location), annotations and number of protein-protein interactions deduced from Arabidopsis knowledgebase. (HTM) [file pone.0038039.s003.htm]

Table�S3


# Table�S3

| Table S3. Differentially expressed genes in apparently healthy fruit in comparison to control (healthy in disease-free location), annotations and number of protein-protein interactions deduced from Arabidopsis knowledgebase. Those that also appear in Table S7 are hightlighted in color. | | | | | | | | | | | |
|  |  |  |  |  |  |  |  |  |  |  |  |
| GB id | id2 | count CO | count AH | norm CO | norm AH | log2foldchange | PPI | annotation |  |  |  |
| EY693454 | S44237646 | 4826 | 302 | 3813.29 | 382.2033 | -3.318623923 | 444 | heat shock protein 82 |  |  |  |
| EY696492 | S44297884 | 42 | 4 | 33.18652 | 5.062296 | -2.712733885 | 69 | microtubule-associated protein rp eb family member 3 ame: full=end-binding protein 3� |  |  |  |
| CN184953 | S22553179 | 8549 | 1642 | 6755.038 | 2078.072 | -1.700718009 | 61 | heat shock 70 kda protein |  |  |  |
| CN191703 | S22550924 | 23 | 61 | 18.17357 | 77.20001 | 2.086758919 | 57 | branched-chain-amino-acid aminotransferase chloroplastic� |  |  |  |
| CX302207 | S24635232 | 18 | 2 | 14.2228 | 2.531148 | -2.490341463 | 56 | tubulin alpha chain |  |  |  |
| EY694175 | S44297233 | 43 | 7 | 33.97668 | 8.859017 | -1.939326295 | 54 | g2 mitotic-specific cyclin-1 |  |  |  |
| DC887942 | S47735884 | 104 | 19 | 82.17616 | 24.0459 | -1.772928667 | 49 | fatty acid 2-hydroxylase ame: full=fatty acid alpha-hydroxylase |  |  |  |
| DN620775 | S24240822 | 3 | 15 | 2.370466 | 18.98361 | 3.001511633 | 48 | ucrcs11\_05m20\_f parent washington navel orange scale-infested rind cdna library ucrcs11 citrus sinensis cdna clone mrna |  |  |  |
| DR908750 | S26279627 | 189 | 36 | 149.3394 | 45.56066 | -1.712733885 | 48 | nuclear transport factor 2� |  |  |  |
| CX072408 | S22602858 | 36 | 66 | 28.44559 | 83.52788 | 1.554052656 | 45 | protein breast cancer susceptibility 1 homolog� |  |  |  |
| CX675417 | S23017596 | 11 | 1 | 8.691709 | 1.265574 | -2.779848081 | 43 | triacylglycerol lipase 2 flags: precursor |  |  |  |
| CB304982 | S22559249 | 64 | 106 | 50.56994 | 134.1508 | 1.407503993 | 36 | nucleolar complex protein 3 homolog� |  |  |  |
| EY662694 | S44287810 | 12942 | 897 | 10226.19 | 1135.22 | -3.171225249 | 31 | �kda proline-rich protein |  |  |  |
| CF833058 | S22568663 | 42 | 8 | 33.18652 | 10.12459 | -1.712733885 | 30 | csl zinc finger domain-containing protein |  |  |  |
| FC921949 | S49955777 | 26 | 55 | 20.54404 | 69.60657 | 1.760503533 | 30 | s-adenosylmethionine-dependent methyltransferase |  |  |  |
| EY700239 | S44243101 | 48 | 5 | 37.92746 | 6.32787 | -2.583450868 | 29 | rac-like gtp-binding protein arac7 ame: full=gtpase protein rop9 |  |  |  |
| EY741416 | S44273324 | 663 | 1453 | 523.873 | 1838.879 | 1.811537465 | 29 | �h aca ribonucleoprotein complex subunit 1-like protein 1 |  |  |  |
| EY754184 | S44281777 | 4 | 12 | 3.160621 | 15.18689 | 2.264546039 | 29 | enhancer of polycomb-like protein |  |  |  |
| EY679658 | S44228252 | 12 | 2 | 9.481864 | 2.531148 | -1.905378963 | 27 | isochorismate chloroplastic flags: precursor |  |  |  |
| EY697005 | S44240315 | 12 | 1 | 9.481864 | 1.265574 | -2.905378963 | 27 | rac-like gtp-binding protein rac2 flags: precursor |  |  |  |
| CF653559 | S22533293 | 320 | 27 | 252.8497 | 34.1705 | -2.887457055 | 24 | pathogenesis-related protein 1� |  |  |  |
| EY711150 | S44251911 | 142 | 232 | 112.2021 | 293.6131 | 1.387817414 | 23 | uncharacterized protein mitochondrial flags: precursor |  |  |  |
| EY757748 | S44283689 | 254 | 492 | 200.6995 | 622.6624 | 1.633413357 | 23 | ctp synthase 2 ame: full=utp--ammonia ligase 2 ame: full=ctp synthetase 2 |  |  |  |
| EY692586 | S44237338 | 12 | 2 | 9.481864 | 2.531148 | -1.905378963 | 22 | dna replication licensing factor mcm5 ame: full=cdc46 homolog ame: full=p1-cdc46 |  |  |  |
| EY664033 | S44216939 | 20 | 42 | 15.80311 | 53.1541 | 1.749972866 | 21 | protein |  |  |  |
| DC900352 | S47736649 | 1021 | 188 | 806.7486 | 237.9279 | -1.761594761 | 20 | chloroplast-targeted copper |  |  |  |
| EY718305 | S44255972 | 9130 | 1639 | 7214.118 | 2074.276 | -1.798215468 | 19 | heat shock protein 101 |  |  |  |
| EY657469 | S44212615 | 318 | 594 | 251.2694 | 751.7509 | 1.581019703 | 17 | probable inositol transporter 2 |  |  |  |
| DY257532 | S34124810 | 11 | 0 | 8.691709 | 0 | -Inf | 15 | protein |  |  |  |
| EY720565 | S44257806 | 847 | 168 | 669.2616 | 212.6164 | -1.654317199 | 15 | auxin efflux carrier family protein |  |  |  |
| DN619030 | S24242071 | 29 | 110 | 22.91451 | 139.2131 | 2.602962256 | 14 | protein |  |  |  |
| EY660636 | S44214998 | 36 | 65 | 28.44559 | 82.2623 | 1.53202635 | 14 | nucleolysin tia- |  |  |  |
| EY745239 | S44275817 | 404 | 800 | 319.2228 | 1012.459 | 1.665228245 | 13 | cbl-interacting serine threonine-protein kinase 14 ame: full=sos2-like protein kinase pks24 ame: full=snf1-related kinase ame: full=serine threonine-protein kinase sr1� |  |  |  |
| EY666662 | S44288880 | 326 | 620 | 257.5906 | 784.6558 | 1.606979789 | 13 | two-component response regulator-like aprr1 ame: full=pseudo-response regulator 1 ame: full=timing of cab expression 1 ame: full=abi3-interacting protein 1 |  |  |  |
| EY654993 | S44210909 | 131 | 219 | 103.5104 | 277.1607 | 1.420947596 | 12 | probable pre-mrna-splicing factor atp-dependent rna helicase |  |  |  |
| EY728087 | S44263101 | 352 | 30 | 278.1347 | 37.96722 | -2.872957485 | 12 | peptide transporter ptr2 ame: full=histidine-transporting protein |  |  |  |
| EY684115 | S44294181 | 2688 | 434 | 2123.938 | 549.2591 | -1.951182652 | 12 | peroxisomal -2-hydroxy-acid oxidase ame: full=glycolate oxidase� |  |  |  |
| CB304835 | S22559132 | 41 | 87 | 32.39637 | 110.1049 | 1.764975029 | 11 | dead-box atp-dependent rna helicase 57 |  |  |  |
| DC900116 | S47736414 | 26566 | 2669 | 20991.27 | 3377.817 | -2.635626271 | 11 | phosphoenolpyruvate carboxykinase� |  |  |  |
| CB610757 | S22560156 | 355 | 877 | 280.5051 | 1109.908 | 1.984341356 | 10 | homeobox-leucine zipper protein athb-12 ame: full=homeodomain transcription factor athb-12 ame: full=hd-zip protein athb-12 |  |  |  |
| CX047840 | S22593588 | 14 | 1 | 11.06217 | 1.265574 | -3.127771384 | 10 | serine threonine-protein kinase aurora-1� |  |  |  |
| CX074546 | S22599307 | 32 | 1 | 25.28497 | 1.265574 | -4.320416462 | 10 | interactor of constitutive active rops 4 |  |  |  |
| DY257364 | S34124642 | 75 | 222 | 59.26165 | 280.9574 | 2.245180714 | 10 | ammonium transporter 2� |  |  |  |
| EY658551 | S44213347 | 2087 | 351 | 1649.054 | 444.2164 | -1.892304127 | 10 | serine mitochondrial� |  |  |  |
| EY673229 | S44223139 | 1098 | 194 | 867.5906 | 245.5213 | -1.821165959 | 10 | �homolog subfamily b member 13 ame: full=testis spermatocyte apoptosis-related gene 6 protein ame: full=testis and spermatogenesis cell-related protein 6 ame: full=testis spermatogenesis apoptosis-related gene 6 protein ame: full=testis spermatogenesis apoptosis-related gene 3 protein |  |  |  |
| CV719482 | S22579072 | 31 | 0 | 24.49482 | 0 | -Inf | 9 | syntaxin-related protein knolle ame: full=syntaxin-111� |  |  |  |
| EY748702 | S44277833 | 20 | 48 | 15.80311 | 60.74755 | 1.942617944 | 9 | 30s ribosomal protein chloroplastic |  |  |  |
| CX050627 | S22591393 | 25 | 60 | 19.75388 | 75.93443 | 1.942617944 | 8 | histidine-containing phosphotransfer protein 5 |  |  |  |
| DY305483 | S34125045 | 2674 | 5798 | 2112.875 | 7337.798 | 1.796139406 | 8 | ferritin- chloroplastic ame: full=s -3 flags: precursor |  |  |  |
| CV712803 | S22579748 | 129 | 271 | 101.93 | 342.9705 | 1.750505324 | 7 | probable cytochrome b5 isoform 2 |  |  |  |
| EY651717 | S44208431 | 169 | 25 | 133.5363 | 31.63935 | -2.077439709 | 7 | conserved hypothetical protein [Ricinus communis] |  |  |  |
| EY688187 | S44234143 | 57 | 2 | 45.03885 | 2.531148 | -4.153306476 | 7 | hva22-like protein c� |  |  |  |
| EY724518 | S44260871 | 2327 | 425 | 1838.692 | 537.8689 | -1.773352926 | 7 | oligopeptide transporter 3� |  |  |  |
| CK740089 | S22536707 | 49 | 10 | 38.71761 | 12.65574 | -1.613198211 | 6 | ala-interacting subunit 3� |  |  |  |
| CX044490 | S22588416 | 107 | 328 | 84.54662 | 415.1082 | 2.295668556 | 6 | sulfate transporter ame: full=ast12 ame: full= 1 |  |  |  |
| DY305635 | S34125197 | 161 | 278 | 127.215 | 351.8295 | 1.467607733 | 6 | squalene monooxygenase ame: full=squalene epoxidase� |  |  |  |
| EY683854 | S44231104 | 256 | 34 | 202.2798 | 43.02951 | -2.232953621 | 6 | fructose-bisphosphate cytoplasmic isozyme 1 |  |  |  |
| EY690487 | S44235673 | 12 | 0 | 9.481864 | 0 | -Inf | 6 | nitrate transporter, putative [Ricinus communis] |  |  |  |
| EY702355 | S44244881 | 22 | 4 | 17.38342 | 5.062296 | -1.779848081 | 6 | ubiquitin carrier protein e2 20 |  |  |  |
| EY714976 | S44254071 | 417 | 60 | 329.4948 | 75.93443 | -2.11742944 | 6 | protein |  |  |  |
| EY757042 | S44283319 | 383 | 709 | 302.6295 | 897.2919 | 1.568024773 | 6 | probable ribose-5-phosphate isomerase ame: full=phosphoriboisomerase |  |  |  |
| FC871823 | S49955477 | 14 | 30 | 11.06217 | 37.96722 | 1.779119211 | 6 | predicted protein [Populus trichocarpa] |  |  |  |
| EY675704 | S44224970 | 389 | 39 | 307.3704 | 49.35738 | -2.638640588 | 5 | ring finger and chy zinc finger domain-containing protein 1 ame: full=zinc finger protein 363 ame: full=ch-rich-interacting match with plag1 ame: full=androgen receptor n-terminal-interacting protein ame: full=p53-induced ring-h2 protein� |  |  |  |
| EY680829 | S44229087 | 155 | 28 | 122.4741 | 35.43607 | -1.789185945 | 5 | adipor-like receptor cg5315 |  |  |  |
| EY687316 | S44233580 | 20 | 2 | 15.80311 | 2.531148 | -2.642344557 | 5 | gamma-glutamyltranspeptidase 1 ame: full=gamma-glutamyltransferase 1 contains: ame: full=gamma-glutamyltranspeptidase 1 heavy chain contains: ame: full=gamma-glutamyltranspeptidase 1 light chain flags: precursor |  |  |  |
| EY698519 | S44241493 | 12 | 1 | 9.481864 | 1.265574 | -2.905378963 | 5 | cyclin-a3-2 ame: full=g2 mitotic-specific cyclin-a3-2� |  |  |  |
| EY746955 | S44276870 | 243 | 7 | 192.0078 | 8.859017 | -4.437874044 | 5 | aquaporin |  |  |  |
| EY751310 | S44279553 | 60 | 101 | 47.40932 | 127.823 | 1.430904425 | 5 | amp deaminase� |  |  |  |
| CX044129 | S22586518 | 494 | 1172 | 390.3367 | 1483.253 | 1.925973161 | 4 | probable wrky transcription factor 53 ame: full=wrky dna-binding protein 53 |  |  |  |
| CX053999 | S22592973 | 104 | 186 | 82.17616 | 235.3967 | 1.518302631 | 4 | mitochondrial import inner membrane translocase subunit tim23 |  |  |  |
| CX070827 | S22597584 | 152 | 348 | 120.1036 | 440.4197 | 1.87459952 | 4 | phosphorylated carbohydrates phosphatase tm\_1254 |  |  |  |
| EY653808 | S44209808 | 148 | 15 | 116.943 | 18.98361 | -2.622979232 | 4 | cell division control protein 2 homolog d |  |  |  |
| EY654967 | S44210883 | 38 | 2 | 30.0259 | 2.531148 | -3.568343975 | 4 | cyclin-b1-4 ame: full=g2 mitotic-specific cyclin-b1-4� |  |  |  |
| EY679753 | S44228347 | 1425 | 2422 | 1125.971 | 3065.22 | 1.444820484 | 4 | protein time for coffee |  |  |  |
| EY701434 | S44243960 | 25 | 3 | 19.75388 | 3.796722 | -2.379310151 | 4 | anaphase-promoting complex subunit cdc20 |  |  |  |
| EY704060 | S44246138 | 3930 | 1667 | 3105.311 | 2109.712 | -0.55769167 | 4 | pleiotropic drug resistance protein 1 ame: full= 1 |  |  |  |
| EY719666 | S44257131 | 3821 | 763 | 3019.184 | 965.6329 | -1.644611758 | 4 | splicing factor pwi domain-containing protein rna recognition motif -containing protein |  |  |  |
| EY752385 | S44280398 | 99 | 13 | 78.22538 | 16.45246 | -2.249333364 | 4 | cyclin-u3-1� |  |  |  |
| EY710755 | S44300374 | 38 | 87 | 30.0259 | 110.1049 | 1.87459952 | 4 | uncharacterized aarf domain-containing protein kinase chloroplastic flags: precursor |  |  |  |
| EY747134 | S44311381 | 777 | 156 | 613.9507 | 197.4295 | -1.636785032 | 4 | tubulin alpha-4 chain ame: full=alpha-4-tubulin |  |  |  |
| CV885158 | S22583334 | 7 | 14 | 5.531087 | 17.71803 | 1.679583538 | 3 | dehydrodolichyl diphosphate synthase 6� |  |  |  |
| CX069649 | S22597043 | 71 | 11 | 56.10103 | 13.92131 | -2.010731963 | 3 | high mobility group family |  |  |  |
| CX302518 | S24635505 | 191 | 7 | 150.9197 | 8.859017 | -4.090490368 | 3 | ubiquitin |  |  |  |
| DR910057 | S26280934 | 5 | 16 | 3.950777 | 20.24918 | 2.357655443 | 3 | phosphoinositide phospholipase c 6 ame: full=phosphoinositide phospholipase plc6� |  |  |  |
| DY305494 | S34125056 | 421 | 2880 | 332.6554 | 3644.853 | 3.453760211 | 3 | probable calcium-binding protein cml45 ame: full=calmodulin-like protein 45 |  |  |  |
| DY306092 | S34125654 | 764 | 1286 | 603.6787 | 1627.528 | 1.430829637 | 3 | riboflavin biosynthesis protein chloroplastic includes: ame: full= -dihydroxy-2-butanone 4-phosphate synthase� |  |  |  |
| EY651251 | S44207965 | 214 | 43 | 169.0932 | 54.41968 | -1.635618694 | 3 | vesicle transport protein sft2b ame: full=sft2 domain-containing protein 2 |  |  |  |
| EY658054 | S44213088 | 2245 | 382 | 1773.899 | 483.4492 | -1.875487364 | 3 | glycine dehydrogenase mitochondrial ame: full=glycine decarboxylase ame: full=glycine cleavage system p-protein flags: precursor |  |  |  |
| EY679459 | S44228053 | 24 | 2 | 18.96373 | 2.531148 | -2.905378963 | 3 | protein hothead ame: full=protein adhesion of calyx edges flags: precursor |  |  |  |
| EY681315 | S44229573 | 84 | 145 | 66.37305 | 183.5082 | 1.467175205 | 3 | mitochondrial carrier |  |  |  |
| EY686806 | S44294722 | 165 | 23 | 130.3756 | 29.1082 | -2.16317672 | 3 | hcs2 (holocarboxylase synthetase 2) biotin- |  |  |  |
| EY756670 | S44314895 | 81 | 15 | 64.00258 | 18.98361 | -1.753375869 | 3 | probable leucine-rich repeat receptor-like protein kinase at5g49770 flags: precursor |  |  |  |
| CN191461 | S22549113 | 64 | 113 | 50.56994 | 143.0099 | 1.4997625 | 2 | upf0706 protein at5g01750 |  |  |  |
| CX043308 | S22586138 | 13 | 0 | 10.27202 | 0 | -Inf | 2 | probable polyamine oxidase 5� |  |  |  |
| CX052342 | S22596007 | 10 | 2 | 7.901554 | 2.531148 | -1.642344557 | 2 | cyclin-d3-1 ame: full=g1 s-specific cyclin-d3-1� |  |  |  |
| DN620167 | S24240472 | 52 | 115 | 41.08808 | 145.541 | 1.824633871 | 2 | arogenate dehydrogenase chloroplastic ame: full= 2 flags: precursor |  |  |  |
| EY657484 | S44212630 | 8 | 27 | 6.321243 | 34.1705 | 2.43447104 | 2 | ser thr-rich protein t10 in dgcr region |  |  |  |
| EY657971 | S44213005 | 9 | 1 | 7.111398 | 1.265574 | -2.490341463 | 2 | kinesin-like protein kif22 ame: full=chromokinesin kid |  |  |  |
| EY676758 | S44225912 | 45 | 78 | 35.55699 | 98.71477 | 1.47313266 | 2 | zinc finger protein constans-like 15 |  |  |  |
| EY690546 | S44235732 | 6 | 10 | 4.740932 | 12.65574 | 1.416549132 | 2 | thymidylate synthase-like |  |  |  |
| EY750241 | S44278826 | 2748 | 163 | 2171.347 | 206.2885 | -3.395854597 | 2 | brassinosteroid-regulated protein bru1 flags: precursor |  |  |  |
| EY650266 | S44284160 | 341 | 832 | 269.443 | 1052.957 | 1.966395327 | 2 | probable protein phosphatase 2c 25� |  |  |  |
| EY727159 | S44305753 | 309 | 606 | 244.158 | 766.9378 | 1.651294494 | 2 | mitochondrial import inner membrane translocase subunit tim17 |  |  |  |
| EY727648 | S44305906 | 3813 | 714 | 3012.862 | 903.6198 | -1.737347014 | 2 | auxin efflux carrier family protein |  |  |  |
| EY747770 | S44311583 | 154 | 280 | 121.6839 | 354.3607 | 1.542080014 | 2 | transcription factor bim1 ame: full=bes1-interacting myc-like protein 1 ame: full=transcription factor en 126 ame: full=bhlh transcription factor bhlh046 ame: full=basic helix-loop-helix protein 46� |  |  |  |
| DC900331 | S47736628 | 76 | 10 | 60.05181 | 12.65574 | -2.246415881 | 2 | homeobox-leucine zipper protein athb-13 ame: full=homeodomain transcription factor athb-13 ame: full=hd-zip protein athb-13 |  |  |  |
| CN187848 | S22545642 | 778 | 1556 | 614.7409 | 1969.233 | 1.679583538 | 1 | dof zinc finger protein� |  |  |  |
| CN192429 | S22549656 | 141 | 200 | 111.4119 | 253.1148 | 1.183888375 | 1 | sulfate transporter ame: full=ast12 ame: full= 1 |  |  |  |
| CV887297 | S22585971 | 70 | 4 | 55.31087 | 5.062296 | -3.449699479 | 1 | patellin-6 |  |  |  |
| CX047376 | S22593342 | 29 | 2 | 22.91451 | 2.531148 | -3.178397457 | 1 | probable xyloglucan endotransglucosylase hydrolase protein 33� |  |  |  |
| CX053924 | S22596841 | 20 | 4 | 15.80311 | 5.062296 | -1.642344557 | 1 | kinesin-1 ame: full=kinesin-like protein a |  |  |  |
| CX676031 | S23017090 | 1083 | 220 | 855.7382 | 278.4263 | -1.619874276 | 1 | transcription factor, putative [Ricinus communis] |  |  |  |
| CX673824 | S23019466 | 183 | 320 | 144.5984 | 404.9837 | 1.485811795 | 1 | chaperone protein dnaj 13� |  |  |  |
| DR404544 | S25679542 | 536 | 1074 | 423.5233 | 1359.226 | 1.682272625 | 1 | protein |  |  |  |
| DY305917 | S34125479 | 5372 | 9545 | 4244.715 | 12079.9 | 1.508869431 | 1 | populus trichocarpa ap2 erf domain-containing transcription factor mrna |  |  |  |
| EY655569 | S44211275 | 65 | 1 | 51.3601 | 1.265574 | -5.342784275 | 1 | 3-ketoacyl- synthase 19� |  |  |  |
| EY659067 | S44213653 | 155 | 30 | 122.4741 | 37.96722 | -1.689650272 | 1 | peptide transporter ptr3-a� |  |  |  |
| EY671382 | S44222188 | 1414 | 202 | 1117.28 | 255.6459 | -2.127771384 | 1 | fructose- - chloroplastic� |  |  |  |
| EY678177 | S44226995 | 347 | 41 | 274.1839 | 51.88853 | -2.40165631 | 1 | glutamine chloroplastic ame: full=gs2 ame: full=glutamate--ammonia ligase flags: precursor |  |  |  |
| EY679960 | S44228442 | 247 | 447 | 195.1684 | 565.7115 | 1.535347328 | 1 | f-box lrr-repeat protein 14 ame: full=f-box and leucine-rich repeat protein 14 |  |  |  |
| EY690668 | S44235854 | 16 | 2 | 12.64249 | 2.531148 | -2.320416462 | 1 | uncharacterized amino acid permease yhdg |  |  |  |
| EY694066 | S44238146 | 1723 | 273 | 1361.438 | 345.5017 | -1.978366307 | 1 | chloroplast-targeted copper |  |  |  |
| EY697179 | S44240489 | 128 | 24 | 101.1399 | 30.37377 | -1.735453961 | 1 | PREDICTED: hypothetical protein [Vitis vinifera] |  |  |  |
| EY700195 | S44243057 | 22 | 43 | 17.38342 | 54.41968 | 1.646416674 | 1 | f-box lrr-repeat protein 3 |  |  |  |
| EY705175 | S44247141 | 133 | 10 | 105.0907 | 12.65574 | -3.053770803 | 1 | tetrapyrrole-binding chloroplastic ame: full=genomes uncoupled 4 flags: precursor |  |  |  |
| EY722059 | S44258958 | 259 | 543 | 204.6502 | 687.2066 | 1.747583638 | 1 | protein |  |  |  |
| EY738027 | S44270817 | 326 | 546 | 257.5906 | 691.0034 | 1.423612525 | 1 | abc transporter a family member 1� |  |  |  |
| EY650241 | S44284135 | 1161 | 2754 | 917.3704 | 3485.391 | 1.925744125 | 1 | zinc finger protein constans-like 1 |  |  |  |
| EY658428 | S44286330 | 95 | 199 | 75.06476 | 251.8492 | 1.74635255 | 1 | inositol 2-dehydrogenase ame: full=myo-inositol 2-dehydrogenase� |  |  |  |
| EY661115 | S44287113 | 42 | 71 | 33.18652 | 89.85575 | 1.437013235 | 1 | palmitoyl-monogalactosyldiacylglycerol delta-7 chloroplastic ame: full=monogalactosyldiacylglycerol-specific palmitic acid desaturase ame: full=fad5 flags: precursor |  |  |  |
| EY686801 | S44294717 | 35 | 7 | 27.65544 | 8.859017 | -1.642344557 | 1 | cyclin-d1-1 ame: full=g1 s-specific cyclin-d1-1� |  |  |  |
| EY746577 | S44311160 | 268 | 451 | 211.7616 | 570.7738 | 1.430477971 | 1 | f-box protein skip2 ame: full=skp1-interacting partner 2 |  |  |  |
| EY748275 | S44311752 | 240 | 497 | 189.6373 | 628.9902 | 1.729794984 | 1 | protease 2 ame: full=protease ii ame: full=oligopeptidase b |  |  |  |
| BQ623132 | S22530951 | 3224 | 3543 | 2547.461 | 4483.928 | 0.81570326 | 0 | PREDICTED: hypothetical protein [Vitis vinifera] |  |  |  |
| BQ624033 | S22531852 | 55 | 10 | 43.45854 | 12.65574 | -1.779848081 | 0 | usda-fp\_01124 ridge pineapple sweet orange entire seedling citrus sinensis cdna clone usda-fp\_01124 5 mrna |  |  |  |
| BQ624477 | S22532296 | 57 | 10 | 45.03885 | 12.65574 | -1.831378381 | 0 | blue copper protein flags: precursor |  |  |  |
| BQ624512 | S22532331 | 3 | 14 | 2.370466 | 17.71803 | 2.901975959 | 0 | probable glutathione s-transferase ame: full=heat shock protein 26a ame: full=g2-4 |  |  |  |
| BQ624514 | S22532333 | 1604 | 147 | 1267.409 | 186.0394 | -2.768202544 | 0 | gdsl esterase lipase at5g55050 ame: full=extracellular lipase at5g55050 flags: precursor |  |  |  |
| BQ625107 | S22532926 | 95 | 11 | 75.06476 | 13.92131 | -2.430840452 | 0 | protein |  |  |  |
| BQ625197 | S22533016 | 47 | 8 | 37.1373 | 10.12459 | -1.875005314 | 0 | sambucus nigra clone xp2 expansin complete cds |  |  |  |
| CF653219 | S22533093 | 23 | 0 | 18.17357 | 0 | -Inf | 0 | citrus sinensis complete genome |  |  |  |
| CF653188 | S22533360 | 98 | 33 | 77.43522 | 41.76394 | -0.890732187 | 0 | probable nad h-dependent oxidoreductase 1 |  |  |  |
| CK665205 | S22533689 | 43 | 6 | 33.97668 | 7.593443 | -2.161718716 | 0 | tomato extensin mrna (clone ug-18) |  |  |  |
| CK665498 | S22534213 | 30 | 4 | 23.70466 | 5.062296 | -2.227307058 | 0 | protein |  |  |  |
| CK701701 | S22534723 | 258 | 16 | 203.8601 | 20.24918 | -3.331643717 | 0 | usda-fp\_4962 ridge pineapple sweet orange entire seedling citrus sinensis cdna clone rse37e10 5 mrna |  |  |  |
| CK701747 | S22535105 | 17 | 2 | 13.43264 | 2.531148 | -2.407879303 | 0 | usda-fp\_5008 ridge pineapple sweet orange entire seedling citrus sinensis cdna clone rse39e04 5 mrna |  |  |  |
| CK739846 | S22536135 | 10 | 0 | 7.901554 | 0 | -Inf | 0 | elongation factor 1-alpha� |  |  |  |
| CK740222 | S22536764 | 4 | 63 | 3.160621 | 79.73116 | 4.656863461 | 0 | protein |  |  |  |
| CK933339 | S22536817 | 10 | 2 | 7.901554 | 2.531148 | -1.642344557 | 0 | cgf1004286\_g04 developing fruit juice sac at 38 dafb citrus sinensis cdna clone jsjune0002\_if\_g04 5 mrna |  |  |  |
| CK932708 | S22536924 | 63 | 2 | 49.77979 | 2.531148 | -4.297696386 | 0 | endoglucanase 8 ame: full=endo- -beta glucanase 8 ame: full=cellulase 1� |  |  |  |
| CK933067 | S22537163 | 195 | 33 | 154.0803 | 41.76394 | -1.883352656 | 0 | cgf1004348\_a07 developing fruit juice sac at 38 dafb citrus sinensis cdna clone jsjune0004\_iif\_a07 5 mrna |  |  |  |
| CK933074 | S22537170 | 12 | 0 | 9.481864 | 0 | -Inf | 0 | crambin precursor=thionin variant thi2ca9 |  |  |  |
| CK933109 | S22537191 | 41 | 9 | 32.39637 | 11.39017 | -1.508043465 | 0 | cgf1004347\_d12 developing fruit juice sac at 38 dafb citrus sinensis cdna clone jsjune0004\_if\_d12 5 mrna |  |  |  |
| CK933216 | S22537247 | 19 | 2 | 15.01295 | 2.531148 | -2.568343975 | 0 | cgf1004289\_b08 developing fruit juice sac at 38 dafb citrus sinensis cdna clone jsjune0002\_ivf\_b08 5 mrna |  |  |  |
| CK933484 | S22537451 | 82 | 15 | 64.79274 | 18.98361 | -1.771077871 | 0 | xyloglucan endotransglucosylase hydrolase protein 9� |  |  |  |
| CK933667 | S22537632 | 34 | 18 | 26.86528 | 22.78033 | -0.237954302 | 0 | cgf1004282\_g05 developing fruit juice sac at 38 dafb citrus sinensis cdna clone jsjune0003\_if\_g05 5 mrna |  |  |  |
| CK933841 | S22537808 | 43 | 8 | 33.97668 | 10.12459 | -1.746681217 | 0 | cgf1004278\_f03 developing fruit juice sac at 38 dafb citrus sinensis cdna clone jsjune0001\_if\_f03 5 mrna |  |  |  |
| CK933235 | S22538059 | 133 | 22 | 105.0907 | 27.84263 | -1.916267279 | 0 | b-type cyclin |  |  |  |
| CK933909 | S22538125 | 12 | 32 | 9.481864 | 40.49837 | 2.094621037 | 0 | �gem-like protein 8 |  |  |  |
| CK933934 | S22538150 | 741 | 132 | 585.5051 | 167.0558 | -1.809352075 | 0 | conserved hypothetical protein [Ricinus communis] |  |  |  |
| CK934089 | S22538305 | 10 | 32 | 7.901554 | 40.49837 | 2.357655443 | 0 | medicago truncatula chromosome 7 clone mth2- complete sequence |  |  |  |
| CK934405 | S22538583 | 20 | 0 | 15.80311 | 0 | -Inf | 0 | protein |  |  |  |
| CK934441 | S22538619 | 106 | 15 | 83.75647 | 18.98361 | -2.141446321 | 0 | cgf1004259\_a05 developing fruit peel at 38 dafb citrus sinensis cdna clone p38dab10004\_iif\_a05 5 mrna |  |  |  |
| CK934971 | S22539149 | 69 | 2 | 54.52072 | 2.531148 | -4.428940919 | 0 | protein |  |  |  |
| CK935574 | S22539458 | 2 | 12 | 1.580311 | 15.18689 | 3.264546039 | 0 | protein |  |  |  |
| CK935341 | S22539631 | 502 | 486 | 396.658 | 615.0689 | 0.632852488 | 0 | unknown [Populus trichocarpa] |  |  |  |
| CK935405 | S22539665 | 150 | 22 | 118.5233 | 27.84263 | -2.089803534 | 0 | unknown [Glycine max] |  |  |  |
| CK935886 | S22539998 | 134 | 674 | 105.8808 | 852.9968 | 3.010099129 | 0 | protein |  |  |  |
| CK936202 | S22540314 | 1881 | 306 | 1486.282 | 387.2656 | -1.940312753 | 0 | phosphoenolpyruvate carboxykinase� |  |  |  |
| CK936331 | S22540449 | 10 | 2 | 7.901554 | 2.531148 | -1.642344557 | 0 | transcription factor bhlh135 ame: full=transcription factor en 67 ame: full=bhlh transcription factor bhlh135 ame: full=basic helix-loop-helix protein 135� |  |  |  |
| CK936343 | S22540460 | 711 | 24 | 561.8005 | 30.37377 | -4.209159711 | 0 | subtilisin-like protease ame: full=cucumisin-like serine protease flags: precursor |  |  |  |
| CK936435 | S22540547 | 4 | 27 | 3.160621 | 34.1705 | 3.43447104 | 0 | cgf1004382\_d07 developing fruit 24 dafb citrus sinensis cdna clone t24dab0001\_ivf\_d07 5 mrna |  |  |  |
| CK936444 | S22540564 | 6 | 15 | 4.740932 | 18.98361 | 2.001511633 | 0 | cgf1004382\_c08 developing fruit 24 dafb citrus sinensis cdna clone t24dab0001\_ivf\_c08 5 mrna |  |  |  |
| CK935321 | S22540610 | 33 | 24 | 26.07513 | 30.37377 | 0.220151919 | 0 | dc900926 yjs citrus unshiu cdna clone yjs0893 5 mrna |  |  |  |
| CK937755 | S22541875 | 1 | 7 | 0.790155 | 8.859017 | 3.48693846 | 0 | cgf1004482\_d07 developing fruit albedo at 80 dafb in p x2 vector citrus sinensis cdna clone a80dab0001\_ivf\_d07 5 mrna |  |  |  |
| CK938028 | S22542069 | 6 | 20 | 4.740932 | 25.31148 | 2.416549132 | 0 | cgf1004479\_b01 developing fruit albedo at 80 dafb in p x2 vector citrus sinensis cdna clone a80dab0001\_if\_b01 5 mrna |  |  |  |
| CK938850 | S22542588 | 64 | 7 | 50.56994 | 8.859017 | -2.51306154 | 0 | cgf1004437\_e06 developing fruit albedo at 165 dafb citrus sinensis cdna clone a1650002\_iif\_e06 5 mrna |  |  |  |
| CK938946 | S22542684 | 53 | 84 | 41.87823 | 106.3082 | 1.343980506 | 0 | cgf1004436\_c07 developing fruit albedo at 165 dafb citrus sinensis cdna clone a1650002\_if\_c07 5 mrna |  |  |  |
| CK938959 | S22542713 | 7 | 40 | 5.531087 | 50.62296 | 3.194156711 | 0 | 30s ribosomal protein chloroplastic |  |  |  |
| CK939006 | S22542755 | 55 | 133 | 43.45854 | 168.3213 | 1.95350626 | 0 | cgf1004435\_e06 developing fruit albedo at 165 dafb citrus sinensis cdna clone a1650003\_ivf\_e06 5 mrna |  |  |  |
| CK939007 | S22542756 | 89 | 7 | 70.32383 | 8.859017 | -2.988794971 | 0 | peptide transporter ptr5 |  |  |  |
| CK938998 | S22542763 | 9 | 15 | 7.111398 | 18.98361 | 1.416549132 | 0 | cgf1004435\_f06 developing fruit albedo at 165 dafb citrus sinensis cdna clone a1650003\_ivf\_f06 5 mrna |  |  |  |
| CK939077 | S22542826 | 7 | 1 | 5.531087 | 1.265574 | -2.127771384 | 0 | cgf1004434\_e02 developing fruit albedo at 165 dafb citrus sinensis cdna clone a1650003\_iiif\_e02 5 mrna |  |  |  |
| CK939266 | S22543283 | 145 | 21 | 114.5725 | 26.57705 | -2.108008129 | 0 | cgf1004432\_c03 developing fruit albedo at 165 dafb citrus sinensis cdna clone a1650003\_if\_c03 5 mrna |  |  |  |
| CK938405 | S22543309 | 11 | 22 | 8.691709 | 27.84263 | 1.679583538 | 0 | cgf1004443\_c10 developing fruit albedo at 165 dafb citrus sinensis cdna clone a1650001\_ivf\_c10 5 mrna |  |  |  |
| CK938667 | S22543424 | 243 | 498 | 192.0078 | 630.2558 | 1.714772966 | 0 | protein |  |  |  |
| CK939678 | S22543802 | 106 | 225 | 83.75647 | 284.7541 | 1.765444275 | 0 | hypothetical protein [Vitis vinifera] |  |  |  |
| CK939668 | S22543803 | 7 | 17 | 5.531087 | 21.51476 | 1.959691457 | 0 | cgf1004747\_c07 developing fruit flavedo at 165 dafb citrus sinensis cdna clone f1650002\_iiif\_c07 5 mrna |  |  |  |
| CK939844 | S22543970 | 677 | 69 | 534.9352 | 87.3246 | -2.614904029 | 0 | cgf1004744\_h08 developing fruit flavedo at 165 dafb citrus sinensis cdna clone f1650003\_ivf\_h08 5 mrna |  |  |  |
| CK940014 | S22544138 | 23 | 62 | 18.17357 | 78.46558 | 2.110217892 | 0 | cgf1004742\_e04 developing fruit flavedo at 165 dafb citrus sinensis cdna clone f1650003\_iif\_e04 5 mrna |  |  |  |
| CK940099 | S22544223 | 126 | 353 | 99.55957 | 446.7476 | 2.165827988 | 0 | calcium-binding protein cml37 ame: full=calmodulin-like protein 37 |  |  |  |
| CN185598 | S22544557 | 383 | 76 | 302.6295 | 96.18362 | -1.653689531 | 0 | ethylene-responsive transcription factor 1b� |  |  |  |
| CN185762 | S22544619 | 2063 | 3434 | 1630.09 | 4345.981 | 1.414729756 | 0 | copper transporter 1 |  |  |  |
| CN185801 | S22544640 | 5 | 37 | 3.950777 | 46.82623 | 3.567108809 | 0 | probable lrr receptor-like serine threonine-protein kinase at2g23950 flags: precursor |  |  |  |
| CN185980 | S22544726 | 12 | 38 | 9.481864 | 48.09181 | 2.342548551 | 0 | ucrcs05\_0002c20\_f washington navel orange stored fruit pulp cdna library citrus sinensis cdna clone mrna |  |  |  |
| CN186662 | S22545043 | 21 | 133 | 16.59326 | 168.3213 | 3.342548551 | 0 | indole-3-acetic acid-amido synthetase ame: full=auxin-responsive gh3-like protein 4� |  |  |  |
| CN187647 | S22545540 | 11 | 44 | 8.691709 | 55.68525 | 2.679583538 | 0 | predicted protein [Populus trichocarpa] |  |  |  |
| CN188214 | S22545831 | 38 | 61 | 30.0259 | 77.20001 | 1.362393362 | 0 | udp- |  |  |  |
| CN188208 | S22545836 | 120 | 28 | 94.81864 | 35.43607 | -1.419952136 | 0 | thaumatin-like protein 1 flags: precursor |  |  |  |
| CN185964 | S22546468 | 7 | 16 | 5.531087 | 20.24918 | 1.872228616 | 0 | vitis vinifera contig whole genome shotgun sequence |  |  |  |
| CN186015 | S22546485 | 5 | 28 | 3.950777 | 35.43607 | 3.165010365 | 0 | protein |  |  |  |
| CN186149 | S22546539 | 349 | 1019 | 275.7642 | 1289.62 | 2.225438648 | 0 | protein |  |  |  |
| CN187016 | S22546917 | 38060 | 62548 | 30073.31 | 79159.12 | 1.396271731 | 0 | rhizanthes zippelii 26s ribosomal rna partial sequence |  |  |  |
| CN188086 | S22547394 | 2922 | 551 | 2308.834 | 697.3312 | -1.727248416 | 0 | ucrcs05\_0005h16\_f washington navel orange stored fruit pulp cdna library citrus sinensis cdna clone mrna |  |  |  |
| CN188941 | S22547810 | 131 | 22 | 103.5104 | 27.84263 | -1.894407845 | 0 | predicted protein [Populus trichocarpa] |  |  |  |
| CN190127 | S22548431 | 36 | 139 | 28.44559 | 175.9148 | 2.628599609 | 0 | ucrcs06\_0002p09\_r washington navel orange stored fruit rind cdna library citrus sinensis cdna clone mrna |  |  |  |
| CN191560 | S22549168 | 316 | 702 | 249.6891 | 888.4329 | 1.83113001 | 0 | vitis vinifera contig whole genome shotgun sequence |  |  |  |
| CN191678 | S22550905 | 68 | 117 | 53.73056 | 148.0721 | 1.462485416 | 0 | ucrcs06\_0005k01\_f washington navel orange stored fruit rind cdna library citrus sinensis cdna clone mrna |  |  |  |
| CN182507 | S22551834 | 565 | 990 | 446.4378 | 1252.918 | 1.488761196 | 0 | ucrcs04\_0002j05\_r ruby orange developing flower cdna library citrus sinensis cdna clone mrna |  |  |  |
| CN184985 | S22553198 | 9 | 40 | 7.111398 | 50.62296 | 2.831586631 | 0 | protein gast1 flags: precursor |  |  |  |
| CN185151 | S22553288 | 833 | 56 | 658.1994 | 70.87214 | -3.215234225 | 0 | probable sulfate transporter |  |  |  |
| CN185028 | S22554792 | 58 | 154 | 45.82901 | 194.8984 | 2.088389084 | 0 | ucrcs04\_0006j12\_r ruby orange developing flower cdna library citrus sinensis cdna clone mrna |  |  |  |
| CB290531 | S22555209 | 100 | 193 | 79.01554 | 244.2558 | 1.628184385 | 0 | PREDICTED: hypothetical protein [Vitis vinifera] |  |  |  |
| CB291261 | S22555615 | 5202 | 2667 | 4110.388 | 3375.286 | -0.284265036 | 0 | PREDICTED: hypothetical protein [Vitis vinifera] |  |  |  |
| CB291342 | S22555660 | 9 | 46 | 7.111398 | 58.2164 | 3.033220493 | 0 | aluminum-activated malate transporter-like |  |  |  |
| CB291413 | S22555698 | 6 | 14 | 4.740932 | 17.71803 | 1.901975959 | 0 | ucrcs01\_02de03\_g1 washington navel orange cold acclimated flavedo & albedo cdna library citrus sinensis cdna clone mrna |  |  |  |
| CB292276 | S22556183 | 31 | 85 | 24.49482 | 107.5738 | 2.134778164 | 0 | protein |  |  |  |
| CB292559 | S22556340 | 287 | 669 | 226.7746 | 846.6689 | 1.900539012 | 0 | serine threonine-protein kinase 1 6 |  |  |  |
| CB293368 | S22556792 | 6 | 48 | 4.740932 | 60.74755 | 3.679583538 | 0 | ucrcs01\_06aa05\_g1 washington navel orange cold acclimated flavedo & albedo cdna library citrus sinensis cdna clone mrna |  |  |  |
| CB293734 | S22556991 | 343 | 619 | 271.0233 | 783.3903 | 1.531314371 | 0 | ucrcs01\_06cd04\_g1 washington navel orange cold acclimated flavedo & albedo cdna library citrus sinensis cdna clone mrna |  |  |  |
| CB290495 | S22557275 | 919 | 2140 | 726.1528 | 2708.328 | 1.899057568 | 0 | protein |  |  |  |
| CB291196 | S22557573 | 13 | 2 | 10.27202 | 2.531148 | -2.02085618 | 0 | protein |  |  |  |
| CB291292 | S22557611 | 67 | 216 | 52.94041 | 273.364 | 2.36838185 | 0 | circadian clock coupling factor zgt |  |  |  |
| CB292401 | S22558075 | 262 | 592 | 207.0207 | 749.2198 | 1.855613902 | 0 | 40s ribosomal protein s24-1 |  |  |  |
| CB293236 | S22558423 | 39 | 82 | 30.81606 | 103.7771 | 1.751733324 | 0 | nac domain-containing protein 71� |  |  |  |
| CB293811 | S22558665 | 2125 | 3620 | 1679.08 | 4581.378 | 1.448110394 | 0 | ucrcs01\_06da01\_b1 washington navel orange cold acclimated flavedo & albedo cdna library citrus sinensis cdna clone mrna |  |  |  |
| CB304390 | S22558759 | 9 | 25 | 7.111398 | 31.63935 | 2.153514726 | 0 | flavedo0001\_ii \_b07 flavedo mature citrus sinensis cdna clone flavedo0001\_ii \_b07 3 mrna |  |  |  |
| CB304426 | S22558777 | 18 | 31 | 14.2228 | 39.23279 | 1.463854847 | 0 | e3 ubiquitin-protein ligase rnf34 ame: full=ring finger protein 34 ame: full=ring finger protein momo |  |  |  |
| CB304607 | S22558946 | 307 | 525 | 242.5777 | 664.4263 | 1.453662305 | 0 | ethylene-responsive transcription factor erf061 |  |  |  |
| CB304386 | S22559428 | 71 | 125 | 56.10103 | 158.1967 | 1.495620703 | 0 | flavedo0001\_ii \_a11 flavedo mature citrus sinensis cdna clone flavedo0001\_ii \_a11 3 mrna |  |  |  |
| CB417401 | S22559767 | 10 | 1 | 7.901554 | 1.265574 | -2.642344557 | 0 | PREDICTED: hypothetical protein [Vitis vinifera] |  |  |  |
| CB610680 | S22560093 | 6 | 12 | 4.740932 | 15.18689 | 1.679583538 | 0 | albedo0003\_ii \_c11 mature albedo citrus sinensis cdna clone albedo0003\_ii \_c11 5 mrna |  |  |  |
| CB610814 | S22560187 | 3 | 0 | 2.370466 | 0 | -Inf | 0 | albedo0002\_i \_b12 mature albedo citrus sinensis cdna clone albedo0002\_i \_b12 5 mrna |  |  |  |
| CB611167 | S22560410 | 23 | 1 | 18.17357 | 1.265574 | -3.843978418 | 0 | �kda class i heat shock protein ame: full= kda heat shock protein 1� |  |  |  |
| CB610726 | S22560525 | 272 | 47 | 214.9223 | 59.48197 | -1.853290452 | 0 | albedo0002\_ \_h09 mature albedo citrus sinensis cdna clone albedo0002\_ \_h09 5 mrna |  |  |  |
| CB610787 | S22560553 | 42 | 7 | 33.18652 | 8.859017 | -1.905378963 | 0 | albedo0002\_i \_h05 mature albedo citrus sinensis cdna clone albedo0002\_i \_h05 5 mrna |  |  |  |
| CF417814 | S22561171 | 10 | 1 | 7.901554 | 1.265574 | -2.642344557 | 0 | floral homeotic protein deficiens |  |  |  |
| CF417159 | S22561371 | 20 | 2 | 15.80311 | 2.531148 | -2.642344557 | 0 | conserved hypothetical protein [Ricinus communis] |  |  |  |
| CF507764 | S22565613 | 476 | 2355 | 376.1139 | 2980.427 | 2.986277119 | 0 | usda-fp\_123000-265 immature ovaries from field-collected valencia sweet orange (citrus sinensis ( ) osbeck) citrus sinensis cdna clone mvf-60\_c08 5 mrna |  |  |  |
| CF508975 | S22566824 | 18 | 175 | 14.2228 | 221.4754 | 3.960869648 | 0 | u-box domain-containing protein 19 ame: full=plant u-box protein 19 |  |  |  |
| CF510057 | S22567906 | 17 | 3 | 13.43264 | 3.796722 | -1.822916803 | 0 | kinesin-4 ame: full=kinesin-like protein d |  |  |  |
| CF833296 | S22568777 | 97 | 10 | 76.64507 | 12.65574 | -2.598401209 | 0 | ucrcs02\_03g01\_r ruby orange ovary at anthesis cdna library citrus sinensis cdna clone mrna |  |  |  |
| CF833645 | S22568937 | 33 | 6 | 26.07513 | 7.593443 | -1.779848081 | 0 | protein |  |  |  |
| CF833658 | S22568943 | 288 | 60 | 227.5647 | 75.93443 | -1.583450868 | 0 | ucrcs02\_03p21\_r ruby orange ovary at anthesis cdna library citrus sinensis cdna clone mrna |  |  |  |
| CF834709 | S22569448 | 211 | 54 | 166.7228 | 68.34099 | -1.286628149 | 0 | ucrcs02\_05p23\_r ruby orange ovary at anthesis cdna library citrus sinensis cdna clone mrna |  |  |  |
| CF832225 | S22569883 | 10 | 2 | 7.901554 | 2.531148 | -1.642344557 | 0 | ucrcs02\_01i08\_r ruby orange ovary at anthesis cdna library citrus sinensis cdna clone mrna |  |  |  |
| CF834184 | S22570834 | 141 | 23 | 111.4119 | 29.1082 | -1.936405858 | 0 | populus trichocarpa mrna |  |  |  |
| CF834198 | S22570840 | 196 | 514 | 154.8704 | 650.505 | 2.070498243 | 0 | ring-h2 finger protein atl3b ame: full=ring-h2 finger protein atl6 flags: precursor |  |  |  |
| CF834253 | S22570867 | 23 | 49 | 18.17357 | 62.01312 | 1.770731426 | 0 | hat dimerisation domain-containing protein |  |  |  |
| CF834267 | S22570874 | 9 | 24 | 7.111398 | 30.37377 | 2.094621037 | 0 | lob domain-containing protein 38 ame: full=asymmetric leaves 2-like protein 40� |  |  |  |
| CF834421 | S22570947 | 139 | 10 | 109.8316 | 12.65574 | -3.11742944 | 0 | 3-ketoacyl- synthase 11� |  |  |  |
| CF836416 | S22571911 | 382 | 680 | 301.8393 | 860.5903 | 1.511545646 | 0 | ucrcs03\_02o21\_f washington navel orange shoot meristem cdna library citrus sinensis cdna clone mrna |  |  |  |
| CF836738 | S22572065 | 9 | 1 | 7.111398 | 1.265574 | -2.490341463 | 0 | retrovirus-related pol polyprotein from transposon tnt 1-94 includes: ame: full=protease includes: ame: full=reverse transcriptase includes: ame: full=endonuclease |  |  |  |
| CF837114 | S22572251 | 18 | 4 | 14.2228 | 5.062296 | -1.490341463 | 0 | ucrcs03\_04c02\_f washington navel orange shoot meristem cdna library citrus sinensis cdna clone mrna |  |  |  |
| CF838727 | S22573043 | 16 | 4 | 12.64249 | 5.062296 | -1.320416462 | 0 | citrus sinensis dna binding protein (v03-2) complete cds |  |  |  |
| CF838781 | S22573068 | 32 | 6 | 25.28497 | 7.593443 | -1.735453961 | 0 | ucrcs03\_06j10\_f washington navel orange shoot meristem cdna library citrus sinensis cdna clone mrna |  |  |  |
| CF835360 | S22573161 | 54 | 117 | 42.66839 | 148.0721 | 1.795060755 | 0 | ucrcs03\_01b20\_r washington navel orange shoot meristem cdna library citrus sinensis cdna clone mrna |  |  |  |
| CF836044 | S22573488 | 60 | 12 | 47.40932 | 15.18689 | -1.642344557 | 0 | squamosa promoter-binding |  |  |  |
| CF836098 | S22573518 | 190 | 11 | 150.1295 | 13.92131 | -3.430840452 | 0 | ice binding |  |  |  |
| CF837348 | S22574131 | 1108 | 3798 | 875.4921 | 4806.65 | 2.456865562 | 0 | PREDICTED: hypothetical protein [Vitis vinifera] |  |  |  |
| CF838077 | S22574489 | 5 | 14 | 3.950777 | 17.71803 | 2.165010365 | 0 | protein |  |  |  |
| CF838214 | S22574550 | 106 | 16 | 83.75647 | 20.24918 | -2.048336917 | 0 | disease resistance response protein 206 |  |  |  |
| CF838525 | S22574705 | 91 | 206 | 71.90414 | 260.7082 | 1.858289425 | 0 | protein |  |  |  |
| CV713002 | S22575386 | 8 | 25 | 6.321243 | 31.63935 | 2.323439728 | 0 | nicotiana tabacum mitochondrial complete genome |  |  |  |
| CV713105 | S22575445 | 10 | 18 | 7.901554 | 22.78033 | 1.527580445 | 0 | ucrcs08\_0002f09\_r parent washington navel orange callus cdna library ucrcs08-1 citrus sinensis cdna clone mrna |  |  |  |
| CV713606 | S22575728 | 18 | 1 | 14.2228 | 1.265574 | -3.490341463 | 0 | cellulose synthase a catalytic subunit 3� |  |  |  |
| CV714710 | S22576355 | 182 | 337 | 143.8083 | 426.4984 | 1.568393679 | 0 | protein |  |  |  |
| CV715004 | S22576523 | 14 | 2 | 11.06217 | 2.531148 | -2.127771384 | 0 | athila retroelement orf1 protein |  |  |  |
| CV715748 | S22576949 | 1 | 1 | 0.790155 | 1.265574 | 0.679583538 | 0 | ucrcs08\_0006d19\_r parent washington navel orange callus cdna library ucrcs08-1 citrus sinensis cdna clone mrna |  |  |  |
| CV715903 | S22577038 | 35 | 1 | 27.65544 | 1.265574 | -4.449699479 | 0 | wound induced protein |  |  |  |
| CV715984 | S22577083 | 1 | 0 | 0.790155 | 0 | -Inf | 0 | abc transporter g family member 8� |  |  |  |
| CV716488 | S22577370 | 1301 | 213 | 1027.992 | 269.5672 | -1.931112089 | 0 | citrus unshiu gene for glycine-rich complete cds |  |  |  |
| CV716489 | S22577371 | 361 | 398 | 285.2461 | 503.6984 | 0.820353132 | 0 | ent-kaurene oxidase� |  |  |  |
| CV718227 | S22578359 | 32 | 59 | 25.28497 | 74.66886 | 1.562226587 | 0 | probable cytochrome c biosynthesis protein |  |  |  |
| CV718293 | S22578395 | 8 | 15 | 6.321243 | 18.98361 | 1.586474134 | 0 | fragaria vesca americana clone fosmid complete sequence |  |  |  |
| CV719263 | S22578951 | 29 | 50 | 22.91451 | 63.2787 | 1.465458733 | 0 | PREDICTED: hypothetical protein [Vitis vinifera] |  |  |  |
| CV719693 | S22579191 | 1 | 2 | 0.790155 | 2.531148 | 1.679583538 | 0 | ucrcs08\_0012f04\_f parent washington navel orange callus cdna library ucrcs08-1 citrus sinensis cdna clone mrna |  |  |  |
| CV720001 | S22579367 | 408 | 57 | 322.3834 | 72.13771 | -2.15995179 | 0 | ucrcs08\_0012m10\_r parent washington navel orange callus cdna library ucrcs08-1 citrus sinensis cdna clone mrna |  |  |  |
| CV712673 | S22579690 | 11 | 0 | 8.691709 | 0 | -Inf | 0 | at1g67050 |  |  |  |
| CV713065 | S22579858 | 331 | 753 | 261.5414 | 952.9772 | 1.865402186 | 0 | taxadien-5-alpha-ol o-acetyltransferase ame: full=taxa-4 -dien-5alpha-ol-o-acetyltransferase� |  |  |  |
| CV713122 | S22579883 | 57 | 4 | 45.03885 | 5.062296 | -3.153306476 | 0 | glucan endo- -beta-glucosidase ame: full=(1- |  |  |  |
| CV713541 | S22580062 | 33 | 6 | 26.07513 | 7.593443 | -1.779848081 | 0 | mutator-like transposase |  |  |  |
| CV713559 | S22580072 | 17 | 32 | 13.43264 | 40.49837 | 1.592120697 | 0 | hypothetical protein NitaMp116 [Nicotiana tabacum] |  |  |  |
| CV713629 | S22580102 | 3 | 10 | 2.370466 | 12.65574 | 2.416549132 | 0 | ucrcs08\_0003b09\_r parent washington navel orange callus cdna library ucrcs08-1 citrus sinensis cdna clone mrna |  |  |  |
| CV714282 | S22580378 | 11 | 21 | 8.691709 | 26.57705 | 1.612469342 | 0 | �trna pseudouridine synthase pus10 ame: full=trna pseudouridine 55 synthase� |  |  |  |
| CV714944 | S22580664 | 266 | 497 | 210.1813 | 628.9902 | 1.581403144 | 0 | abc transporter g family member 39� |  |  |  |
| CV715155 | S22580755 | 30 | 121 | 23.70466 | 153.1344 | 2.69155618 | 0 | early nodulin-93� |  |  |  |
| CV715612 | S22580948 | 1 | 5 | 0.790155 | 6.32787 | 3.001511633 | 0 | ucrcs08\_0006a07\_f parent washington navel orange callus cdna library ucrcs08-1 citrus sinensis cdna clone mrna |  |  |  |
| CV717128 | S22581594 | 262 | 36 | 207.0207 | 45.56066 | -2.183914462 | 0 | peptidase m50 |  |  |  |
| CV718150 | S22582030 | 3 | 11 | 2.370466 | 13.92131 | 2.554052656 | 0 | protein |  |  |  |
| CV718748 | S22582282 | 20 | 33 | 15.80311 | 41.76394 | 1.402049562 | 0 | ucrcs08\_0010p03\_f parent washington navel orange callus cdna library ucrcs08-1 citrus sinensis cdna clone mrna |  |  |  |
| CV718780 | S22582298 | 66 | 2 | 52.15025 | 2.531148 | -4.364810581 | 0 | plz12\_luppoprotein pplz12 |  |  |  |
| CV718921 | S22582359 | 41 | 121 | 32.39637 | 153.1344 | 2.240894771 | 0 | non-symbiotic hemoglobin 1 ame: full=medsa glb1 |  |  |  |
| CV719618 | S22582656 | 49 | 3 | 38.71761 | 3.796722 | -3.350163805 | 0 | ucrcs08\_0012d12\_r parent washington navel orange callus cdna library ucrcs08-1 citrus sinensis cdna clone mrna |  |  |  |
| CV719871 | S22582765 | 5 | 11 | 3.950777 | 13.92131 | 1.817087062 | 0 | transcription factor bhlh113 ame: full=transcription factor en 61 ame: full=bhlh transcription factor bhlh113 ame: full=basic helix-loop-helix protein 113� |  |  |  |
| CV886175 | S22583829 | 145 | 17 | 114.5725 | 21.51476 | -2.412862711 | 0 | linalool chloroplastic flags: precursor |  |  |  |
| CV886525 | S22583988 | 211 | 792 | 166.7228 | 1002.335 | 2.587840969 | 0 | zinc finger protein constans-like 10 |  |  |  |
| CV886834 | S22584129 | 15 | 0 | 11.85233 | 0 | -Inf | 0 | probable rhamnose biosynthetic enzyme 3 |  |  |  |
| CV887227 | S22584311 | 33 | 4 | 26.07513 | 5.062296 | -2.364810581 | 0 | hypothetical protein [Vitis vinifera] |  |  |  |
| CV887353 | S22584367 | 15 | 0 | 11.85233 | 0 | -Inf | 0 | protein |  |  |  |
| CV885396 | S22585018 | 355 | 39 | 280.5051 | 49.35738 | -2.506689458 | 0 | protein |  |  |  |
| CV886949 | S22585789 | 240 | 433 | 189.6373 | 547.9935 | 1.530916157 | 0 | blue copper protein flags: precursor |  |  |  |
| CX043988 | S22586454 | 56 | 4 | 44.2487 | 5.062296 | -3.127771384 | 0 | meiosis 5 |  |  |  |
| CX046065 | S22587428 | 194 | 876 | 153.2901 | 1108.643 | 2.854457755 | 0 | reticuline oxidase-like protein flags: precursor |  |  |  |
| CX044433 | S22588384 | 21 | 0 | 16.59326 | 0 | -Inf | 0 | PREDICTED: hypothetical protein [Vitis vinifera] |  |  |  |
| CX045842 | S22589131 | 410 | 34 | 323.9637 | 43.02951 | -2.91243372 | 0 | unknown protein [Arabidopsis thaliana] |  |  |  |
| CX046356 | S22589397 | 242 | 26 | 191.2176 | 32.90492 | -2.538839981 | 0 | receptor protein kinase clavata1 flags: precursor |  |  |  |
| CX046518 | S22589484 | 178 | 35 | 140.6477 | 44.29509 | -1.666866876 | 0 | fatty acyl- reductase 3 ame: full=protein eceriferum 4 |  |  |  |
| CX046529 | S22589490 | 4 | 16 | 3.160621 | 20.24918 | 2.679583538 | 0 | ucrcs07\_8f04\_b parent washington navel orange thrip-challenged flavedo cdna library ucrcs07 citrus sinensis cdna clone ucrcs07-8f04-k8-1- mrna |  |  |  |
| CX046605 | S22589528 | 27 | 85 | 21.33419 | 107.5738 | 2.334086972 | 0 | uncharacterized plant-specific domain tigr01615 family expressed |  |  |  |
| CX046913 | S22589674 | 32 | 56 | 25.28497 | 70.87214 | 1.48693846 | 0 | lea34\_goshilate embryogenesis abundant protein d-34 (lea d-34) |  |  |  |
| CX047170 | S22589791 | 5 | 16 | 3.950777 | 20.24918 | 2.357655443 | 0 | nicotiana tabacum mitochondrial complete genome |  |  |  |
| CX047182 | S22589796 | 11 | 2 | 8.691709 | 2.531148 | -1.779848081 | 0 | serine-threonine protein plant- |  |  |  |
| CX047329 | S22589866 | 7 | 23 | 5.531087 | 29.1082 | 2.395790572 | 0 | ucrcs09\_13e05\_b ruby orange developing seed cdna library ucrcs09 citrus sinensis cdna clone ucrcs09-13e05-i10-1- mrna |  |  |  |
| CX047858 | S22590108 | 3 | 12 | 2.370466 | 15.18689 | 2.679583538 | 0 | hypothetical protein Cagg\_1305 [Chloroflexus aggregans DSM 9485] |  |  |  |
| CX047991 | S22590171 | 23 | 48 | 18.17357 | 60.74755 | 1.740984083 | 0 | ribosomal protein mitochondrial |  |  |  |
| CX048288 | S22590309 | 16 | 2 | 12.64249 | 2.531148 | -2.320416462 | 0 | seed biotin-containing protein sbp65 ame: full=seed biotinylated protein of 65 kda ame: full=bp75 |  |  |  |
| CX048538 | S22590428 | 4 | 10 | 3.160621 | 12.65574 | 2.001511633 | 0 | brassica rapa campestris clone complete sequence |  |  |  |
| CX048690 | S22590496 | 44 | 70 | 34.76684 | 88.59017 | 1.349434936 | 0 | 60s ribosomal protein mitochondrial |  |  |  |
| CX048758 | S22590529 | 273 | 47 | 215.7124 | 59.48197 | -1.858584751 | 0 | gdsl esterase lipase apg ame: full=extracellular lipase apg flags: precursor |  |  |  |
| CX049094 | S22590683 | 473 | 85 | 373.7435 | 107.5738 | -1.796721899 | 0 | �domain-containing gpi-anchored protein 1 flags: precursor |  |  |  |
| CX049732 | S22590978 | 53 | 108 | 41.87823 | 136.682 | 1.706550586 | 0 | protein |  |  |  |
| CX049939 | S22591074 | 13 | 26 | 10.27202 | 32.90492 | 1.679583538 | 0 | predicted protein [Populus trichocarpa] |  |  |  |
| CX049956 | S22591084 | 5 | 12 | 3.950777 | 15.18689 | 1.942617944 | 0 | vitis vinifera contig whole genome shotgun sequence |  |  |  |
| CX051758 | S22591917 | 23 | 3 | 18.17357 | 3.796722 | -2.259015917 | 0 | protein |  |  |  |
| CX052699 | S22592354 | 87 | 4 | 68.74352 | 5.062296 | -3.763359958 | 0 | probable flavin-containing monooxygenase 1 |  |  |  |
| CX046840 | S22593052 | 23 | 39 | 18.17357 | 49.35738 | 1.441423801 | 0 | ucrcs09\_10e09\_b ruby orange developing seed cdna library ucrcs09 citrus sinensis cdna clone ucrcs09-10e09-j17-1- mrna |  |  |  |
| CX046929 | S22593100 | 12 | 0 | 9.481864 | 0 | -Inf | 0 | 30s ribosomal protein chloroplastic |  |  |  |
| CX047166 | S22593228 | 10 | 17 | 7.901554 | 21.51476 | 1.445118284 | 0 | ucrcs09\_12e09\_g ruby orange developing seed cdna library ucrcs09 citrus sinensis cdna clone ucrcs09-12e09-i17-1- mrna |  |  |  |
| CX047321 | S22593311 | 18 | 0 | 14.2228 | 0 | -Inf | 0 | phosphoribosylanthranilate transferase |  |  |  |
| CX047453 | S22593383 | 13 | 2 | 10.27202 | 2.531148 | -2.02085618 | 0 | protein |  |  |  |
| CX047721 | S22593526 | 401 | 1038 | 316.8523 | 1313.666 | 2.05171584 | 0 | potassium channel tetramerization domain-containing |  |  |  |
| CX048061 | S22593707 | 9 | 0 | 7.111398 | 0 | -Inf | 0 | ucrcs09\_18e06\_b ruby orange developing seed cdna library ucrcs09 citrus sinensis cdna clone ucrcs09-18e06-j11-1- mrna |  |  |  |
| CX048087 | S22593721 | 68 | 13 | 53.73056 | 16.45246 | -1.707439585 | 0 | leucine-rich repeat receptor protein kinase exs ame: full=extra sporogenous cells protein ame: full=protein excess microsporocytes 1 flags: precursor |  |  |  |
| CX048531 | S22593959 | 1648 | 1236 | 1302.176 | 1564.249 | 0.264546039 | 0 | protein |  |  |  |
| CX048752 | S22594078 | 6 | 13 | 4.740932 | 16.45246 | 1.795060755 | 0 | hypothetical protein MtrDRAFT\_AC167711g44v2 [Medicago truncatula] |  |  |  |
| CX048969 | S22594194 | 35 | 63 | 27.65544 | 79.73116 | 1.527580445 | 0 | arabidopsis thaliana mitochondrial genome |  |  |  |
| CX049741 | S22594611 | 1088 | 1698 | 859.689 | 2148.945 | 1.32174144 | 0 | ubiquitin domain-containing protein 1 |  |  |  |
| CX049781 | S22594633 | 291 | 40 | 229.9352 | 50.62296 | -2.18336371 | 0 | transcription factor |  |  |  |
| CX049845 | S22594667 | 2 | 15 | 1.580311 | 18.98361 | 3.586474134 | 0 | protein |  |  |  |
| CX049911 | S22594703 | 2 | 16 | 1.580311 | 20.24918 | 3.679583538 | 0 | hypoxia induced protein conserved region containing expressed |  |  |  |
| CX049971 | S22594732 | 2285 | 404 | 1805.505 | 511.2919 | -1.820183429 | 0 | uncharacterized gpi-anchored protein at1g27950 flags: precursor |  |  |  |
| CX050002 | S22594743 | 9 | 17 | 7.111398 | 21.51476 | 1.597121378 | 0 | nicotiana tabacum mitochondrial complete genome |  |  |  |
| CX050195 | S22594852 | 38 | 100 | 30.0259 | 126.5574 | 2.075512214 | 0 | protein |  |  |  |
| CX051341 | S22595470 | 5 | 10 | 3.950777 | 12.65574 | 1.679583538 | 0 | fragaria vesca americana clone fosmid complete sequence |  |  |  |
| CX051519 | S22595562 | 429 | 49 | 338.9766 | 62.01312 | -2.450540455 | 0 | 3-ketoacyl- synthase 10� |  |  |  |
| CX051582 | S22595596 | 21 | 3 | 16.59326 | 3.796722 | -2.127771384 | 0 | ucrcs09\_40g10\_b ruby orange developing seed cdna library ucrcs09 citrus sinensis cdna clone ucrcs09-40g10-m19-1- mrna |  |  |  |
| CX052259 | S22595961 | 6 | 11 | 4.740932 | 13.92131 | 1.554052656 | 0 | protein ycf2 |  |  |  |
| CX053885 | S22596821 | 15 | 2 | 11.85233 | 2.531148 | -2.227307058 | 0 | auxin transporter-like protein 5 ame: full=aux1-like protein 5 ame: full= 5 |  |  |  |
| CX069789 | S22597106 | 33 | 7 | 26.07513 | 8.859017 | -1.557455659 | 0 | retrovirus-related pol polyprotein from transposon tnt 1-94 includes: ame: full=protease includes: ame: full=reverse transcriptase includes: ame: full=endonuclease |  |  |  |
| CX070091 | S22597247 | 103 | 13 | 81.386 | 16.45246 | -2.306477271 | 0 | snakin-2 flags: precursor |  |  |  |
| CX070156 | S22597277 | 10 | 18 | 7.901554 | 22.78033 | 1.527580445 | 0 | predicted protein [Populus trichocarpa] |  |  |  |
| CX071153 | S22597738 | 12 | 1 | 9.481864 | 1.265574 | -2.905378963 | 0 | unnamed protein product [Vitis vinifera] |  |  |  |
| CX071402 | S22597854 | 53 | 10 | 41.87823 | 12.65574 | -1.726408822 | 0 | populus trichocarpa mrna |  |  |  |
| CX071698 | S22597989 | 875 | 88 | 691.3859 | 111.3705 | -2.63412405 | 0 | ucrcs08\_23d06\_g parent washington navel orange callus cdna library ucrcs08-2 citrus sinensis cdna clone ucrcs08-23d06-h11-1- mrna |  |  |  |
| CX072481 | S22598352 | 101 | 441 | 79.80569 | 558.1181 | 2.806006901 | 0 | ucrcs08\_28d01\_b parent washington navel orange callus cdna library ucrcs08-2 citrus sinensis cdna clone ucrcs08-28d01-h2-1- mrna |  |  |  |
| CX073325 | S22598744 | 38 | 7 | 30.0259 | 8.859017 | -1.760989053 | 0 | transcription factor tga2 ame: full=hbp-1b homolog� |  |  |  |
| CX073495 | S22598823 | 22 | 2 | 17.38342 | 2.531148 | -2.779848081 | 0 | ucrcs08\_33d09\_b parent washington navel orange callus cdna library ucrcs08-2 citrus sinensis cdna clone ucrcs08-33d09-g17-1- mrna |  |  |  |
| CX074287 | S22599188 | 89 | 275 | 70.32383 | 348.0328 | 2.307137915 | 0 | ring-h2 finger protein atl1m |  |  |  |
| CX074337 | S22599210 | 69 | 9 | 54.52072 | 11.39017 | -2.259015917 | 0 | probable gibberellin receptor gid1l2 ame: full=gid1-like protein 2 |  |  |  |
| CX074987 | S22599512 | 164 | 23 | 129.5855 | 29.1082 | -2.154406511 | 0 | chloroplast-targeted copper |  |  |  |
| CX075508 | S22599753 | 2854 | 407 | 2255.103 | 515.0886 | -2.130301097 | 0 | ethylene-responsive transcription factor erf012 |  |  |  |
| CX076967 | S22600430 | 174 | 24 | 137.487 | 30.37377 | -2.178397457 | 0 | populus trichocarpa mrna |  |  |  |
| CX077288 | S22600576 | 23 | 42 | 18.17357 | 53.1541 | 1.548339005 | 0 | bap2 (bon association protein 2) |  |  |  |
| CX077578 | S22600712 | 36 | 68 | 28.44559 | 86.05903 | 1.597121378 | 0 | hypothetical protein NitaMp078 [Nicotiana tabacum] |  |  |  |
| CX077877 | S22600852 | 151 | 15 | 119.3135 | 18.98361 | -2.651930606 | 0 | u-box domain-containing protein 11 ame: full=plant u-box protein 11 |  |  |  |
| CX069655 | S22601382 | 10 | 0 | 7.901554 | 0 | -Inf | 0 | peroxidase 72� |  |  |  |
| CX070848 | S22602021 | 22 | 43 | 17.38342 | 54.41968 | 1.646416674 | 0 | probable glutamate carboxypeptidase 2 ame: full=probable glutamate carboxypeptidase ii |  |  |  |
| CX071680 | S22602466 | 10 | 0 | 7.901554 | 0 | -Inf | 0 | serine carboxypeptidase 24 ame: full=serine carboxypeptidase ii ame: full=carboxypeptidase d ame: full=bri1 suppressor 1 contains: ame: full=serine carboxypeptidase 24 chain a ame: full=serine carboxypeptidase ii chain a contains: ame: full=serine carboxypeptidase 24 chain b ame: full=serine carboxypeptidase ii chain b flags: precursor |  |  |  |
| CX072640 | S22602985 | 14 | 2 | 11.06217 | 2.531148 | -2.127771384 | 0 | ucrcs08\_29c12\_g parent washington navel orange callus cdna library ucrcs08-2 citrus sinensis cdna clone ucrcs08-29c12-e23-1- mrna |  |  |  |
| CX073773 | S22603590 | 267 | 489 | 210.9715 | 618.8656 | 1.552578261 | 0 | probable protein phosphatase 2c 2� |  |  |  |
| CX074986 | S22604245 | 234 | 30 | 184.8964 | 37.96722 | -2.283890586 | 0 | chloroplast-targeted copper |  |  |  |
| CX075413 | S22604474 | 10 | 1 | 7.901554 | 1.265574 | -2.642344557 | 0 | ucrcs08\_44e07\_g parent washington navel orange callus cdna library ucrcs08-2 citrus sinensis cdna clone ucrcs08-44e07-j14-1- mrna |  |  |  |
| CX075513 | S22604527 | 43 | 7 | 33.97668 | 8.859017 | -1.939326295 | 0 | ucrcs08\_45c11\_b parent washington navel orange callus cdna library ucrcs08-2 citrus sinensis cdna clone ucrcs08-45c11-e21-1- mrna |  |  |  |
| CX075930 | S22604752 | 64 | 14 | 50.56994 | 17.71803 | -1.51306154 | 0 | dna polymerase beta |  |  |  |
| CX075961 | S22604770 | 34 | 56 | 26.86528 | 70.87214 | 1.399475619 | 0 | ucrcs08\_48a02\_b parent washington navel orange callus cdna library ucrcs08-2 citrus sinensis cdna clone ucrcs08-48a02-b4-1- mrna |  |  |  |
| CX076036 | S22604809 | 42 | 94 | 33.18652 | 118.9639 | 1.841854967 | 0 | dehydration-responsive element-binding protein 1c� |  |  |  |
| CX076101 | S22604844 | 8 | 126 | 6.321243 | 159.4623 | 4.656863461 | 0 | embryogenic cell protein 40� |  |  |  |
| CX078334 | S22606007 | 9 | 7 | 7.111398 | 8.859017 | 0.317013459 | 0 | PREDICTED: hypothetical protein [Vitis vinifera] |  |  |  |
| AF255013 | S22606190 | 62 | 11 | 48.98963 | 13.92131 | -1.815181154 | 0 | trans-cinnamate 4-monooxygenase ame: full=cinnamic acid 4-hydroxylase� |  |  |  |
| AF321533 | S22606192 | 828 | 2929 | 654.2486 | 3706.866 | 2.502289058 | 0 | 1-aminocyclopropane-1-carboxylate oxidase� |  |  |  |
| U82976 | S22606218 | 19993 | 3206 | 15797.58 | 4057.43 | -1.9610651 | 0 | pectinesterase 1� |  |  |  |
| CX675213 | S23016713 | 9 | 1 | 7.111398 | 1.265574 | -2.490341463 | 0 | ring-h2 finger protein atl1l |  |  |  |
| CX675291 | S23016749 | 9 | 15 | 7.111398 | 18.98361 | 1.416549132 | 0 | 11-beta-hydroxysteroid dehydrogenase-like |  |  |  |
| CX675523 | S23016855 | 2721 | 414 | 2150.013 | 523.9476 | -2.036850746 | 0 | protein |  |  |  |
| CX675813 | S23016991 | 47 | 7 | 37.1373 | 8.859017 | -2.067650392 | 0 | btb poz domain-containing protein at5g48800 |  |  |  |
| CX676095 | S23017119 | 15 | 0 | 11.85233 | 0 | -Inf | 0 | probable disease resistance protein at1g61180 |  |  |  |
| CX676293 | S23017212 | 11 | 19 | 8.691709 | 24.0459 | 1.468079433 | 0 | ucrcs08\_68e11\_b parent washington navel orange callus cdna library ucrcs08-3 citrus sinensis cdna clone ucrcs08-68e11-i22-1- mrna |  |  |  |
| CX676294 | S23017213 | 2 | 12 | 1.580311 | 15.18689 | 3.264546039 | 0 | ucrcs08\_68e11\_g parent washington navel orange callus cdna library ucrcs08-3 citrus sinensis cdna clone ucrcs08-68e11-i22-1- mrna |  |  |  |
| CX675554 | S23017668 | 37 | 85 | 29.23575 | 107.5738 | 1.879521108 | 0 | ras-gtpase-activating protein-binding |  |  |  |
| CX675684 | S23017738 | 9 | 0 | 7.111398 | 0 | -Inf | 0 | ethylene-responsive transcription factor wri1 ame: full=protein wrinkled 1 ame: full=protein activator of sporamin::luc 1 |  |  |  |
| CX676116 | S23017971 | 22 | 61 | 17.38342 | 77.20001 | 2.150889257 | 0 | 1-aminocyclopropane-1-carboxylate oxidase 1� |  |  |  |
| CX676339 | S23018092 | 10 | 1 | 7.901554 | 1.265574 | -2.642344557 | 0 | ucrcs08\_68h04\_b parent washington navel orange callus cdna library ucrcs08-3 citrus sinensis cdna clone ucrcs08-68h04-o8-1- mrna |  |  |  |
| CX676530 | S23018178 | 27 | 67 | 21.33419 | 84.79345 | 1.990785226 | 0 | b-cell receptor-associated protein 31-like containing protein |  |  |  |
| CX671518 | S23018337 | 10 | 0 | 7.901554 | 0 | -Inf | 0 | protein |  |  |  |
| CX671835 | S23018494 | 23 | 36 | 18.17357 | 45.56066 | 1.325946583 | 0 | populus trichocarpa mrna |  |  |  |
| CX672039 | S23018591 | 84 | 0 | 66.37305 | 0 | -Inf | 0 | subtilisin-like protease ame: full=cucumisin-like serine protease flags: precursor |  |  |  |
| CX672325 | S23018731 | 174 | 49 | 137.487 | 62.01312 | -1.148650114 | 0 | zinc transporter 1 ame: full=zrt irt-like protein 1 flags: precursor |  |  |  |
| CX672588 | S23018860 | 11 | 0 | 8.691709 | 0 | -Inf | 0 | protein |  |  |  |
| CX674161 | S23019647 | 9 | 0 | 7.111398 | 0 | -Inf | 0 | cral trio domain containing expressed |  |  |  |
| CX674345 | S23019747 | 11 | 2 | 8.691709 | 2.531148 | -1.779848081 | 0 | ucrcs10\_5h05\_b madame vinous sweet orange multiple pathogen-infected cdna library ucrcs10 citrus sinensis cdna clone ucrcs10-5h05-o9- mrna |  |  |  |
| CX674613 | S23019889 | 61 | 1 | 48.19948 | 1.265574 | -5.2511538 | 0 | protein |  |  |  |
| CX674977 | S23020085 | 317 | 49 | 250.4792 | 62.01312 | -2.014045648 | 0 | oligopeptide transporter 1� |  |  |  |
| CX671269 | S23020102 | 940 | 788 | 742.746 | 997.2722 | 0.425118411 | 0 | ucrcs10\_10a07\_b madame vinous sweet orange multiple pathogen-infected cdna library ucrcs10 citrus sinensis cdna clone ucrcs10-10a07-a14- mrna |  |  |  |
| CX671357 | S23020146 | 186 | 10 | 146.9689 | 12.65574 | -3.537647178 | 0 | l-ascorbate oxidase homolog flags: precursor |  |  |  |
| CX671667 | S23020305 | 42 | 68 | 33.18652 | 86.05903 | 1.374728956 | 0 | basic 7s globulin ame: full=sbg7s� |  |  |  |
| CX672332 | S23020647 | 13 | 0 | 10.27202 | 0 | -Inf | 0 | poncirus trifoliata citrus tristeza virus resistance gene complete sequence |  |  |  |
| CX673259 | S23021042 | 712 | 404 | 562.5906 | 511.2919 | -0.13793841 | 0 | shoot1 protein |  |  |  |
| CX673305 | S23021068 | 16 | 2 | 12.64249 | 2.531148 | -2.320416462 | 0 | probable lrr receptor-like serine threonine-protein kinase at1g34110 flags: precursor |  |  |  |
| CX673376 | S23021107 | 1154 | 163 | 911.8393 | 206.2885 | -2.144115816 | 0 | ring-h2 finger protein atl2n |  |  |  |
| CX674961 | S23021431 | 19 | 3 | 15.01295 | 3.796722 | -1.983381475 | 0 | protein |  |  |  |
| CX674127 | S23021662 | 35 | 61 | 27.65544 | 77.20001 | 1.481037859 | 0 | protein |  |  |  |
| DN134801 | S23749932 | 193 | 39 | 152.5 | 49.35738 | -1.62747128 | 0 | transcription factor bhlh91 ame: full=transcription factor en 25 ame: full=bhlh transcription factor bhlh091 ame: full=basic helix-loop-helix protein 91� |  |  |  |
| DN135042 | S23750027 | 6 | 20 | 4.740932 | 25.31148 | 2.416549132 | 0 | proteinase inhibitor ame: full=luti |  |  |  |
| DN618700 | S24239635 | 20 | 40 | 15.80311 | 50.62296 | 1.679583538 | 0 | glycine max strain williams 82 clone complete sequence |  |  |  |
| DN620718 | S24240789 | 83 | 206 | 65.58289 | 260.7082 | 1.991044634 | 0 | serine threonine-protein kinase oxi1 ame: full=protein oxidative signal-inducible 1 |  |  |  |
| DN619200 | S24242145 | 24 | 90 | 18.96373 | 113.9017 | 2.586474134 | 0 | cytochrome p450 94a2 ame: full=p450-dependent fatty acid omega-hydroxylase |  |  |  |
| DN620794 | S24242827 | 43 | 105 | 33.97668 | 132.8853 | 1.967564301 | 0 | f-box family protein |  |  |  |
| DN620931 | S24242884 | 72 | 14 | 56.89119 | 17.71803 | -1.682986541 | 0 | �phagocytic receptor 1b ame: full= -induced gene c protein flags: precursor |  |  |  |
| CX301048 | S24634819 | 4 | 67 | 3.160621 | 84.79345 | 4.745672728 | 0 | u-box domain-containing protein 21 ame: full=plant u-box protein 21 |  |  |  |
| CX301073 | S24634830 | 7 | 16 | 5.531087 | 20.24918 | 1.872228616 | 0 | n-acetyltransferase 13 ame: full=n-acetyltransferase 5� |  |  |  |
| CX301243 | S24634956 | 0 | 1 | 0 | 1.265574 | Inf | 0 | c08007e11sk ootsw1 citrus sinensis cdna clone mrna |  |  |  |
| CX301592 | S24634997 | 14 | 1 | 11.06217 | 1.265574 | -3.127771384 | 0 | probable elongation factor 1-gamma 2� |  |  |  |
| CX301818 | S24635071 | 19 | 134 | 15.01295 | 169.5869 | 3.497745215 | 0 | elongation factor 1-alpha� |  |  |  |
| CX302423 | S24635298 | 19 | 4 | 15.01295 | 5.062296 | -1.568343975 | 0 | ethylene-responsive transcription factor 13� |  |  |  |
| CX302247 | S24635308 | 108 | 22 | 85.33678 | 27.84263 | -1.615872346 | 0 | c08018g01sk ootsw1 citrus sinensis cdna clone mrna |  |  |  |
| CX302564 | S24635511 | 17 | 34 | 13.43264 | 43.02951 | 1.679583538 | 0 | c08003c12sk ootsw1 citrus sinensis cdna clone mrna |  |  |  |
| CX302838 | S24635647 | 37 | 10 | 29.23575 | 12.65574 | -1.207941733 | 0 | citrus sinensis dna binding protein (v03-2) complete cds |  |  |  |
| CX303089 | S24636190 | 35 | 0 | 27.65544 | 0 | -Inf | 0 | c08027d11sk ootsw1 citrus sinensis cdna clone mrna |  |  |  |
| CX303285 | S24636282 | 21 | 1 | 16.59326 | 1.265574 | -3.712733885 | 0 | c08029e10sk ootsw1 citrus sinensis cdna clone mrna |  |  |  |
| DR403849 | S25678847 | 103 | 21 | 81.386 | 26.57705 | -1.614599566 | 0 | phosphoinositide binding |  |  |  |
| DR403990 | S25678988 | 4 | 10 | 3.160621 | 12.65574 | 2.001511633 | 0 | csah-pnp1246o19 developing fruit peel at 38 dafb citrus sinensis cdna clone csah-pnp1246o19 5 mrna |  |  |  |
| DR404142 | S25679140 | 84 | 121 | 66.37305 | 153.1344 | 1.206129352 | 0 | csag-pnp1245g11 developing fruit juice sac at 38 dafb citrus sinensis cdna clone csag-pnp1245g11 5 mrna |  |  |  |
| DR404508 | S25679506 | 407 | 64 | 321.5932 | 80.99673 | -1.989301446 | 0 | protein |  |  |  |
| DR405378 | S25680376 | 0 | 1 | 0 | 1.265574 | Inf | 0 | csad-pnp1242e09 developing fruit flavedo at 165 dafb citrus sinensis cdna clone csad-pnp1242e09 5 mrna |  |  |  |
| DR405437 | S25680435 | 7 | 12 | 5.531087 | 15.18689 | 1.457191117 | 0 | csad-pnp1242h08 developing fruit flavedo at 165 dafb citrus sinensis cdna clone csad-pnp1242h08 5 mrna |  |  |  |
| DR405580 | S25680578 | 0 | 12 | 0 | 15.18689 | Inf | 0 | csad-pnp1242o09 developing fruit flavedo at 165 dafb citrus sinensis cdna clone csad-pnp1242o09 5 mrna |  |  |  |
| DR405840 | S25680838 | 272 | 43 | 214.9223 | 54.41968 | -1.981614549 | 0 | protein |  |  |  |
| DR406100 | S25681098 | 491 | 1077 | 387.9663 | 1363.023 | 1.812806858 | 0 | cysteine proteinase rd21a� |  |  |  |
| DR908518 | S26279395 | 60 | 281 | 47.40932 | 355.6263 | 2.907119263 | 0 | cytochrome p450 76c4 |  |  |  |
| DR909350 | S26280227 | 276 | 15 | 218.0829 | 18.98361 | -3.522050323 | 0 | probable pectinesterase pectinesterase inhibitor 34 includes: ame: full=pectinesterase inhibitor 34 ame: full=pectin methylesterase inhibitor 34 includes: ame: full=pectinesterase 34� |  |  |  |
| DR909527 | S26280404 | 5 | 10 | 3.950777 | 12.65574 | 1.679583538 | 0 | usda-fp\_17655 citrus sinensis phloem citrus sinensis cdna clone vpe-10\_b01 5 mrna |  |  |  |
| DR909873 | S26280750 | 15 | 0 | 11.85233 | 0 | -Inf | 0 | protein |  |  |  |
| DR909987 | S26280864 | 456 | 12 | 360.3108 | 15.18689 | -4.568343975 | 0 | proline-rich glycoprotein |  |  |  |
| DR910310 | S26281187 | 7 | 1 | 5.531087 | 1.265574 | -2.127771384 | 0 | usda-fp\_18438 citrus sinensis phloem citrus sinensis cdna clone vpe-41\_b12 5 mrna |  |  |  |
| DR910337 | S26281214 | 24 | 2 | 18.96373 | 2.531148 | -2.905378963 | 0 | usda-fp\_18465 citrus sinensis phloem citrus sinensis cdna clone vpe-39\_f06 5 mrna |  |  |  |
| DR910631 | S26281508 | 39 | 0 | 30.81606 | 0 | -Inf | 0 | plasma membrane associated protein |  |  |  |
| DR910681 | S26281558 | 17 | 46 | 13.43264 | 58.2164 | 2.115682653 | 0 | usda-fp\_18809 citrus sinensis phloem citrus sinensis cdna clone vpe-29\_c08 5 mrna |  |  |  |
| DR910861 | S26281738 | 5 | 24 | 3.950777 | 30.37377 | 2.942617944 | 0 | usda-fp\_18989 citrus sinensis phloem citrus sinensis cdna clone vpe-14\_f02 5 mrna |  |  |  |
| DR911173 | S26282050 | 62 | 6 | 48.98963 | 7.593443 | -2.689650272 | 0 | usda-fp\_19301 citrus sinensis phloem citrus sinensis cdna clone vpe-31\_a06 5 mrna |  |  |  |
| DR911175 | S26282052 | 38 | 79 | 30.0259 | 99.98034 | 1.735436773 | 0 | usda-fp\_19303 citrus sinensis phloem citrus sinensis cdna clone vpe-45\_d02 5 mrna |  |  |  |
| DR911461 | S26282338 | 95 | 15 | 75.06476 | 18.98361 | -1.983381475 | 0 | usda-fp\_19589 citrus sinensis phloem citrus sinensis cdna clone vpe-35\_d02 5 mrna |  |  |  |
| DR911519 | S26282396 | 153 | 267 | 120.8938 | 337.9082 | 1.482891627 | 0 | usda-fp\_19647 citrus sinensis phloem citrus sinensis cdna clone vpe-42\_h04 5 mrna |  |  |  |
| DR911822 | S26282699 | 16 | 34 | 12.64249 | 43.02951 | 1.767046379 | 0 | usda-fp\_19950 citrus sinensis phloem citrus sinensis cdna clone vpe-14\_h08 5 mrna |  |  |  |
| DR912053 | S26282930 | 6 | 33 | 4.740932 | 41.76394 | 3.139015157 | 0 | usda-fp\_20181 citrus sinensis phloem citrus sinensis cdna clone vpe-09\_a04 5 mrna |  |  |  |
| DQ028471 | S32321255 | 75 | 343 | 59.26165 | 434.0919 | 2.872829614 | 0 | 9-cis-epoxycarotenoid dioxygenase chloroplastic ame: full= 1 flags: precursor |  |  |  |
| DY257155 | S34124433 | 1496 | 287 | 1182.072 | 363.2197 | -1.702403995 | 0 | protein |  |  |  |
| DY257259 | S34124537 | 818 | 1552 | 646.3471 | 1964.171 | 1.603539347 | 0 | kn0aak1da01fm2 ruit citrus sinensis cdna 5 mrna |  |  |  |
| DY257267 | S34124545 | 226 | 25 | 178.5751 | 31.63935 | -2.496739235 | 0 | nucleobase-ascorbate transporter 2� |  |  |  |
| DY257306 | S34124584 | 45 | 164 | 35.55699 | 207.5541 | 2.545282446 | 0 | u-box domain-containing protein 19 ame: full=plant u-box protein 19 |  |  |  |
| DY257343 | S34124621 | 4085 | 494 | 3227.785 | 625.1935 | -2.368169593 | 0 | expansin-like a2� |  |  |  |
| DY257460 | S34124738 | 15 | 2 | 11.85233 | 2.531148 | -2.227307058 | 0 | polyphenol oxidase with tyrosine hydroxylase activity protein |  |  |  |
| DY257659 | S34124937 | 411 | 76 | 324.7538 | 96.18362 | -1.755483532 | 0 | citrus unshiu mrna for metallothionein-like complete cds |  |  |  |
| DY259698 | S34124999 | 46 | 5 | 36.34715 | 6.32787 | -2.522050323 | 0 | predicted protein [Populus trichocarpa] |  |  |  |
| DY305530 | S34125092 | 14 | 35 | 11.06217 | 44.29509 | 2.001511633 | 0 | chlorophyllase- chloroplastic ame: full=chlorophyll-chlorophyllido hydrolase 1� |  |  |  |
| DY305531 | S34125093 | 71509 | 10605 | 56503.22 | 13421.41 | -2.07379667 | 0 | ribulose bisphosphate carboxylase oxygenase activase chloroplastic� |  |  |  |
| DY305580 | S34125142 | 2397 | 5695 | 1894.002 | 7207.443 | 1.928049471 | 0 | zinc finger protein 1 ame: full=wzf1 |  |  |  |
| DY305607 | S34125169 | 627 | 1252 | 495.4274 | 1584.499 | 1.677280752 | 0 | protein |  |  |  |
| DY305613 | S34125175 | 47 | 121 | 37.1373 | 153.1344 | 2.043857924 | 0 | transcription factor bhlh36 ame: full=transcription factor en 6 ame: full=bhlh transcription factor bhlh036 ame: full=basic helix-loop-helix protein 36� |  |  |  |
| DY305655 | S34125217 | 1344 | 104 | 1061.969 | 131.6197 | -3.012294167 | 0 | aspartic proteinase nepenthesin-2 ame: full=nepenthesin-ii flags: precursor |  |  |  |
| DY305681 | S34125243 | 137 | 538 | 108.2513 | 680.8788 | 2.653013818 | 0 | ethylene-responsive transcription factor erf017 |  |  |  |
| DY305694 | S34125256 | 330 | 1044 | 260.7513 | 1321.259 | 2.34116732 | 0 | potassium channel tetramerization domain-containing |  |  |  |
| DY305725 | S34125287 | 159 | 435 | 125.6347 | 550.5247 | 2.131572173 | 0 | tropinone reductase homolog at1g07440 |  |  |  |
| DY305732 | S34125294 | 1421 | 2793 | 1122.811 | 3534.748 | 1.654492557 | 0 | ring-h2 finger protein atl3f ame: full=ring-h2 finger protein atl2 |  |  |  |
| DY305736 | S34125298 | 147 | 268 | 116.1528 | 339.1738 | 1.546000384 | 0 | cytochrome p450 71b35 |  |  |  |
| DY305779 | S34125341 | 149 | 25 | 117.7331 | 31.63935 | -1.895728793 | 0 | alpha galactosidase precursor |  |  |  |
| DY305828 | S34125390 | 11 | 0 | 8.691709 | 0 | -Inf | 0 | phosphoglucomutase phosphomannomutase family protein |  |  |  |
| DY305861 | S34125423 | 5 | 46 | 3.950777 | 58.2164 | 3.881217399 | 0 | protein |  |  |  |
| DY305898 | S34125460 | 273 | 559 | 215.7124 | 707.4558 | 1.71353087 | 0 | calcium-binding protein cml37 ame: full=calmodulin-like protein 37 |  |  |  |
| DY305904 | S34125466 | 4617 | 301 | 3648.147 | 380.9377 | -3.259536802 | 0 | 21 kda protein ame: full= protein flags: precursor |  |  |  |
| DY305910 | S34125472 | 3643 | 666 | 2878.536 | 842.8722 | -1.771949375 | 0 | protein |  |  |  |
| DY305912 | S34125474 | 32 | 86 | 25.28497 | 108.8394 | 2.105848293 | 0 | polyneuridine-aldehyde esterase ame: full=polyneuridine aldehyde esterase flags: precursor |  |  |  |
| DY305919 | S34125481 | 1508 | 109 | 1191.554 | 137.9476 | -3.110652851 | 0 | PREDICTED: hypothetical protein [Vitis vinifera] |  |  |  |
| DY305927 | S34125489 | 202 | 333 | 159.6114 | 421.4361 | 1.400750422 | 0 | protein brittle- chloroplastic amyloplastic flags: precursor |  |  |  |
| DY305970 | S34125532 | 14 | 29 | 11.06217 | 36.70164 | 1.730209611 | 0 | kn0aam3ad01rm1 slh citrus sinensis cdna 5 mrna |  |  |  |
| DY305997 | S34125559 | 486 | 96 | 384.0155 | 121.4951 | -1.660266465 | 0 | probable metal-nicotianamine transporter ysl5 ame: full=protein yellow stripe like 5� |  |  |  |
| DY306001 | S34125563 | 428 | 3126 | 338.1865 | 3956.184 | 3.548218615 | 0 | nac domain-containing protein 72� |  |  |  |
| DY306009 | S34125571 | 1519 | 86 | 1200.246 | 108.8394 | -3.463057862 | 0 | lob domain-containing protein 40 ame: full=asymmetric leaves 2-like protein 37� |  |  |  |
| DY306086 | S34125648 | 50 | 105 | 39.50777 | 132.8853 | 1.749972866 | 0 | receptor serine threonine |  |  |  |
| DY306114 | S34125676 | 1101 | 206 | 869.961 | 260.7082 | -1.738514688 | 0 | homogentisate geranylgeranyl transferase |  |  |  |
| DY306136 | S34125698 | 0 | 130 | 0 | 164.5246 | Inf | 0 | jasmonate o-methyltransferase ame: full=s-adenosyl-l-methionine:jasmonic acid carboxyl methyltransferase ame: full=floral nectary-specific protein 1 |  |  |  |
| DY306181 | S34125743 | 460 | 1017 | 363.4715 | 1287.089 | 1.824197451 | 0 | protein |  |  |  |
| DY306872 | S34125767 | 5 | 22 | 3.950777 | 27.84263 | 2.817087062 | 0 | transcription factor bhlh36 ame: full=transcription factor en 6 ame: full=bhlh transcription factor bhlh036 ame: full=basic helix-loop-helix protein 36� |  |  |  |
| EG358279 | S35174889 | 473 | 1186 | 373.7435 | 1500.971 | 2.005775459 | 0 | blue copper protein flags: precursor |  |  |  |
| EG358327 | S35174937 | 5 | 1 | 3.950777 | 1.265574 | -1.642344557 | 0 | pectinesterase 2� |  |  |  |
| EG358332 | S35174942 | 3 | 12 | 2.370466 | 15.18689 | 2.679583538 | 0 | serine-threonine protein plant- |  |  |  |
| EU200366 | S41488901 | 30 | 243 | 23.70466 | 307.5345 | 3.697505446 | 0 | taxadien-5-alpha-ol o-acetyltransferase ame: full=taxa-4 -dien-5alpha-ol-o-acetyltransferase� |  |  |  |
| EU240878 | S41505081 | 4051 | 838 | 3200.919 | 1060.551 | -1.593672398 | 0 | transcription factor bhlh137 ame: full=transcription factor en 89 ame: full=bhlh transcription factor bhlh137 ame: full=basic helix-loop-helix protein 137� |  |  |  |
| EY649785 | S44206947 | 23 | 6 | 18.17357 | 7.593443 | -1.259015917 | 0 | probable calcium-binding protein cml44 ame: full=calmodulin-like protein 44 |  |  |  |
| EY649805 | S44206967 | 64 | 4 | 50.56994 | 5.062296 | -3.320416462 | 0 | ethylene-responsive transcription factor shine 2 |  |  |  |
| EY650515 | S44207565 | 179 | 401 | 141.4378 | 507.4951 | 1.843226187 | 0 | cytochrome p450 |  |  |  |
| EY650552 | S44207602 | 1114 | 47 | 880.2331 | 59.48197 | -3.887361128 | 0 | subtilisin-like protease ame: full=cucumisin-like serine protease flags: precursor |  |  |  |
| EY650692 | S44207742 | 247 | 39 | 195.1684 | 49.35738 | -1.983381475 | 0 | cytochrome p450 83b1 |  |  |  |
| EY651373 | S44208087 | 897 | 1635 | 708.7693 | 2069.213 | 1.545694283 | 0 | protein |  |  |  |
| EY651631 | S44208345 | 119 | 18 | 94.02849 | 22.78033 | -2.045309224 | 0 | monocopper oxidase-like protein sku5 ame: full=skewed roots flags: precursor |  |  |  |
| EY652156 | S44208660 | 14 | 2 | 11.06217 | 2.531148 | -2.127771384 | 0 | populus trichocarpa clone pop1- complete sequence |  |  |  |
| EY652191 | S44208695 | 10 | 1 | 7.901554 | 1.265574 | -2.642344557 | 0 | expansin-b3� |  |  |  |
| EY652207 | S44208711 | 95 | 12 | 75.06476 | 15.18689 | -2.30530957 | 0 | chlorophyll a-b binding protein chloroplastic ame: full=lhcii type ii cab-151� |  |  |  |
| EY652427 | S44208819 | 12 | 21 | 9.481864 | 26.57705 | 1.48693846 | 0 | protein |  |  |  |
| EY652929 | S44209321 | 22 | 4 | 17.38342 | 5.062296 | -1.779848081 | 0 | probable esterase at1g33990 |  |  |  |
| EY652955 | S44209347 | 456 | 86 | 360.3108 | 108.8394 | -1.727041722 | 0 | anthranilate synthase component i- chloroplastic flags: precursor |  |  |  |
| EY653765 | S44209765 | 23 | 3 | 18.17357 | 3.796722 | -2.259015917 | 0 | acrosin contains: ame: full=acrosin light chain contains: ame: full=acrosin heavy chain flags: precursor |  |  |  |
| EY653870 | S44209870 | 21 | 48 | 16.59326 | 60.74755 | 1.872228616 | 0 | populus trichocarpa mrna |  |  |  |
| EY654505 | S44210421 | 1006 | 133 | 794.8963 | 168.3213 | -2.239548616 | 0 | calmodulin-like protein 3 |  |  |  |
| EY654618 | S44210534 | 3557 | 368 | 2810.583 | 465.7312 | -2.593299765 | 0 | chlorophyll a-b binding protein chloroplastic ame: full=lhcii type ii cab-151� |  |  |  |
| EY654875 | S44210791 | 84 | 15 | 66.37305 | 18.98361 | -1.805843289 | 0 | probable polygalacturonase� |  |  |  |
| EY655352 | S44211058 | 15 | 2 | 11.85233 | 2.531148 | -2.227307058 | 0 | zea mays clone mrna sequence |  |  |  |
| EY655547 | S44211253 | 8 | 14 | 6.321243 | 17.71803 | 1.48693846 | 0 | ankyrin repeat-containing protein at2g01680 |  |  |  |
| EY655817 | S44211439 | 24 | 99 | 18.96373 | 125.2918 | 2.723977657 | 0 | cs00-c1-100-082-f09- sweet orange greenhouse plant citrus sinensis mrna |  |  |  |
| EY656230 | S44211740 | 190 | 350 | 150.1295 | 442.9509 | 1.560939041 | 0 | adenylate kinase 1 |  |  |  |
| EY656241 | S44211751 | 92 | 15 | 72.69429 | 18.98361 | -1.937087822 | 0 | protein |  |  |  |
| EY656328 | S44211838 | 168 | 21 | 132.7461 | 26.57705 | -2.320416462 | 0 | dna-damage-inducible protein |  |  |  |
| EY656449 | S44211959 | 67 | 2 | 52.94041 | 2.531148 | -4.386505653 | 0 | myristoyl-acyl carrier protein chloroplastic ame: full=16:0-acyl-carrier protein thioesterase� |  |  |  |
| EY656524 | S44212034 | 55 | 1 | 43.45854 | 1.265574 | -5.101776176 | 0 | protein hothead ame: full=protein adhesion of calyx edges flags: precursor |  |  |  |
| EY656545 | S44212055 | 6 | 13 | 4.740932 | 16.45246 | 1.795060755 | 0 | abc transporter g family member 5� |  |  |  |
| EY656785 | S44212141 | 87 | 149 | 68.74352 | 188.5705 | 1.455808563 | 0 | pathogen-related protein |  |  |  |
| EY656793 | S44212149 | 41 | 8 | 32.39637 | 10.12459 | -1.677968467 | 0 | cs00-c1-100-124-f07- sweet orange greenhouse plant citrus sinensis mrna |  |  |  |
| EY657220 | S44212422 | 92 | 8 | 72.69429 | 10.12459 | -2.843978418 | 0 | l-ascorbate oxidase homolog ame: full=pollen-specific protein ntp303 flags: precursor |  |  |  |
| EY657482 | S44212628 | 10911 | 2014 | 8621.385 | 2548.866 | -1.758064205 | 0 | probable non-specific lipid-transfer protein akcs9� |  |  |  |
| EY657722 | S44212756 | 687 | 1188 | 542.8367 | 1503.502 | 1.46973637 | 0 | probable phosphatase phospho1 |  |  |  |
| EY657918 | S44212952 | 146 | 232 | 115.3627 | 293.6131 | 1.347739974 | 0 | predicted protein [Populus trichocarpa] |  |  |  |
| EY658086 | S44213120 | 79 | 6 | 62.42227 | 7.593443 | -3.03923471 | 0 | myb family transcription factor apl� |  |  |  |
| EY658129 | S44213149 | 201 | 19 | 158.8212 | 24.0459 | -2.72354064 | 0 | aquaporin nip2-1 ame: full=nod26-like intrinsic protein 2-1 ame: full= 2 1 ame: full=silicon transporter lsi1 ame: full=low silicon protein 1 |  |  |  |
| EY658336 | S44213244 | 19 | 1 | 15.01295 | 1.265574 | -3.568343975 | 0 | 14 kda proline-rich protein flags: precursor |  |  |  |
| EY658341 | S44213249 | 243 | 48 | 192.0078 | 60.74755 | -1.660266465 | 0 | chalcone-flavanone isomerase family expressed |  |  |  |
| EY658528 | S44213324 | 1272 | 260 | 1005.078 | 329.0492 | -1.610931604 | 0 | probable glycerophosphoryl diester phosphodiesterase 2 flags: precursor |  |  |  |
| EY658535 | S44213331 | 158 | 263 | 124.8445 | 332.8459 | 1.414721779 | 0 | PREDICTED: hypothetical protein [Vitis vinifera] |  |  |  |
| EY658548 | S44213344 | 2 | 3 | 1.580311 | 3.796722 | 1.264546039 | 0 | citrus sinensis complete genome |  |  |  |
| EY658585 | S44213381 | 306 | 26 | 241.7875 | 32.90492 | -2.877364587 | 0 | solute carrier family 35 member f1 |  |  |  |
| EY658610 | S44213406 | 52 | 0 | 41.08808 | 0 | -Inf | 0 | gdsl esterase lipase at2g04570 ame: full=extracellular lipase at2g04570 flags: precursor |  |  |  |
| EY658618 | S44213414 | 346 | 57 | 273.3938 | 72.13771 | -1.922154676 | 0 | zinc finger ccch domain-containing protein 12� |  |  |  |
| EY658676 | S44213472 | 1079 | 1996 | 852.5776 | 2526.086 | 1.567000394 | 0 | conserved hypothetical protein [Ricinus communis] |  |  |  |
| EY658729 | S44213525 | 13 | 0 | 10.27202 | 0 | -Inf | 0 | cs00-c1-100-138-f12- sweet orange greenhouse plant citrus sinensis mrna |  |  |  |
| EY658745 | S44213541 | 1397 | 238 | 1103.847 | 301.2066 | -1.873715004 | 0 | glycerate dehydrogenase� |  |  |  |
| EY658787 | S44213583 | 847 | 148 | 669.2616 | 187.3049 | -1.837181256 | 0 | (+)-delta-cadinene synthase isozyme a� |  |  |  |
| EY658792 | S44213588 | 13 | 2 | 10.27202 | 2.531148 | -2.02085618 | 0 | taxadien-5-alpha-ol o-acetyltransferase ame: full=taxa-4 -dien-5alpha-ol-o-acetyltransferase� |  |  |  |
| EY658811 | S44213607 | 453 | 30 | 357.9404 | 37.96722 | -3.236893106 | 0 | fasciclin-like arabinogalactan protein 2 flags: precursor |  |  |  |
| EY659581 | S44214055 | 213 | 38 | 168.3031 | 48.09181 | -1.807198569 | 0 | peroxidase c3 flags: precursor |  |  |  |
| EY659774 | S44214248 | 21 | 2 | 16.59326 | 2.531148 | -2.712733885 | 0 | calcium-dependent protein kinase 2� |  |  |  |
| EY659778 | S44214252 | 11 | 1 | 8.691709 | 1.265574 | -2.779848081 | 0 | armadillo beta-catenin repeat family protein |  |  |  |
| EY659863 | S44214337 | 175 | 23 | 138.2772 | 29.1082 | -2.248065618 | 0 | probable lrr receptor-like serine threonine-protein kinase at3g47570 flags: precursor |  |  |  |
| EY660196 | S44214558 | 37 | 81 | 29.23575 | 102.5115 | 1.809980175 | 0 | probable wrky transcription factor 19 ame: full=wrky dna-binding protein 19 |  |  |  |
| EY660216 | S44214578 | 3 | 13 | 2.370466 | 16.45246 | 2.795060755 | 0 | alpha-glucosidase yihq |  |  |  |
| EY660232 | S44214594 | 42 | 2 | 33.18652 | 2.531148 | -3.712733885 | 0 | s-locus-specific glycoprotein s6� |  |  |  |
| EY660243 | S44214605 | 1920 | 327 | 1517.098 | 413.8427 | -1.874160232 | 0 | tubulin alpha-1 chain |  |  |  |
| EY660318 | S44214680 | 2 | 0 | 1.580311 | 0 | -Inf | 0 | cs00-c1-101-020-h10- sweet orange infected with xylella fastidiosa (stage 1 of 2) citrus sinensis mrna |  |  |  |
| EY660567 | S44214929 | 54 | 3 | 42.66839 | 3.796722 | -3.490341463 | 0 | probable lrr receptor-like serine threonine-protein kinase at3g47570 flags: precursor |  |  |  |
| EY660669 | S44215031 | 14 | 0 | 11.06217 | 0 | -Inf | 0 | predicted protein [Populus trichocarpa] |  |  |  |
| EY661071 | S44215209 | 132 | 21 | 104.3005 | 26.57705 | -1.972493159 | 0 | cs00-c1-101-029-b02- sweet orange infected with xylella fastidiosa (stage 1 of 2) citrus sinensis mrna |  |  |  |
| EY661339 | S44215365 | 152 | 271 | 120.1036 | 342.9705 | 1.513805066 | 0 | protein |  |  |  |
| EY661917 | S44215607 | 82 | 10 | 64.79274 | 12.65574 | -2.356040372 | 0 | ornithine decarboxylase� |  |  |  |
| EY661918 | S44215608 | 147 | 7 | 116.1528 | 8.859017 | -3.712733885 | 0 | protein |  |  |  |
| EY662007 | S44215697 | 10 | 17 | 7.901554 | 21.51476 | 1.445118284 | 0 | pentatricopeptide repeat-containing protein at3g42630 |  |  |  |
| EY663583 | S44216489 | 41 | 3 | 32.39637 | 3.796722 | -3.093005966 | 0 | cs00-c1-101-056-g04- sweet orange infected with xylella fastidiosa (stage 1 of 2) citrus sinensis mrna |  |  |  |
| EY663599 | S44216505 | 18 | 0 | 14.2228 | 0 | -Inf | 0 | cs00-c1-101-056-h08- sweet orange infected with xylella fastidiosa (stage 1 of 2) citrus sinensis mrna |  |  |  |
| EY664384 | S44217178 | 13 | 25 | 10.27202 | 31.63935 | 1.62300001 | 0 | protein |  |  |  |
| EY664535 | S44217329 | 3 | 10 | 2.370466 | 12.65574 | 2.416549132 | 0 | PREDICTED: hypothetical protein [Vitis vinifera] |  |  |  |
| EY665163 | S44217733 | 8 | 22 | 6.321243 | 27.84263 | 2.139015157 | 0 | elongation factor tu gtp-binding domain-containing protein 1 ame: full=protein fam42a |  |  |  |
| EY665215 | S44217785 | 116 | 12 | 91.65802 | 15.18689 | -2.593434956 | 0 | glycerol-3-phosphate acyltransferase 6� |  |  |  |
| EY665311 | S44217881 | 328 | 43 | 259.171 | 54.41968 | -2.251703712 | 0 | predicted protein [Populus trichocarpa] |  |  |  |
| EY665364 | S44217934 | 236 | 389 | 186.4767 | 492.3083 | 1.400566834 | 0 | yth domain-containing |  |  |  |
| EY665690 | S44218260 | 0 | 10 | 0 | 12.65574 | Inf | 0 | cs00-c1-102-092-e05- sweet orange infected with xylella fastidiosa (stage 2 of 2) citrus sinensis mrna |  |  |  |
| EY666753 | S44219001 | 119 | 228 | 94.02849 | 288.5509 | 1.617655789 | 0 | probable aminotransferase acs10 |  |  |  |
| EY667118 | S44219254 | 388 | 63 | 306.5803 | 79.73116 | -1.943049381 | 0 | nad-dependent epimerase dehydratase |  |  |  |
| EY668011 | S44219699 | 108 | 285 | 85.33678 | 360.6886 | 2.079514145 | 0 | reticuline oxidase-like protein flags: precursor |  |  |  |
| EY668407 | S44219983 | 1636 | 89 | 1292.694 | 112.6361 | -3.520640064 | 0 | carbonic chloroplastic ame: full=carbonate dehydratase flags: precursor |  |  |  |
| EY668560 | S44220136 | 87 | 129 | 68.74352 | 163.259 | 1.247867298 | 0 | cs00-c1-102-043-a04- sweet orange infected with xylella fastidiosa (stage 2 of 2) citrus sinensis mrna |  |  |  |
| EY668853 | S44220429 | 25 | 94 | 19.75388 | 118.9639 | 2.5903162 | 0 | PREDICTED: hypothetical protein [Vitis vinifera] |  |  |  |
| EY669763 | S44221017 | 173 | 20 | 136.6969 | 25.31148 | -2.433116595 | 0 | �chloroplastic� |  |  |  |
| EY669903 | S44221157 | 14 | 25 | 11.06217 | 31.63935 | 1.516084806 | 0 | cs00-c1-102-052-h08- sweet orange infected with xylella fastidiosa (stage 2 of 2) citrus sinensis mrna |  |  |  |
| EY670060 | S44221314 | 106 | 496 | 83.75647 | 627.7247 | 2.905859394 | 0 | protein |  |  |  |
| EY670781 | S44221811 | 16 | 46 | 12.64249 | 58.2164 | 2.203145494 | 0 | protein |  |  |  |
| EY671060 | S44221978 | 62 | 126 | 48.98963 | 159.4623 | 1.702667151 | 0 | protein binding |  |  |  |
| EY671339 | S44222145 | 1821 | 265 | 1438.873 | 335.3771 | -2.10108312 | 0 | chlorophyll a-b binding chloroplastic ame: full=lhci type ii cab flags: precursor |  |  |  |
| EY672423 | S44222893 | 2425 | 119 | 1916.127 | 150.6033 | -3.669367731 | 0 | heat shock protein 83 |  |  |  |
| EY672670 | S44223028 | 451 | 988 | 356.3601 | 1250.387 | 1.810967146 | 0 | thioredoxin-like 1 |  |  |  |
| EY673259 | S44223169 | 287 | 504 | 226.7746 | 637.8493 | 1.491956535 | 0 | uncharacterized protein at1g14870 |  |  |  |
| EY673920 | S44223494 | 261 | 13 | 206.2305 | 16.45246 | -3.64788274 | 0 | cs00-c1-102-004-g03- sweet orange infected with xylella fastidiosa (stage 2 of 2) citrus sinensis mrna |  |  |  |
| EY674005 | S44223579 | 335 | 32 | 264.702 | 40.49837 | -2.708433747 | 0 | thylakoid membrane phosphoprotein 14 chloroplastic flags: precursor |  |  |  |
| EY674214 | S44223690 | 132 | 257 | 104.3005 | 325.2525 | 1.640813968 | 0 | protein chloroplast import apparatus 2 flags: precursor |  |  |  |
| EY674234 | S44223710 | 8 | 19 | 6.321243 | 24.0459 | 1.927511051 | 0 | cs00-c1-102-024-a07- sweet orange infected with xylella fastidiosa (stage 2 of 2) citrus sinensis mrna |  |  |  |
| EY674326 | S44223802 | 294 | 54 | 232.3057 | 68.34099 | -1.765201305 | 0 | alcohol dehydrogenase ame: full=aldehyde reductase ame: full=aldo-keto reductase family 1 member a1 |  |  |  |
| EY674342 | S44223818 | 10 | 17 | 7.901554 | 21.51476 | 1.445118284 | 0 | cs00-c1-102-075-b02- sweet orange infected with xylella fastidiosa (stage 2 of 2) citrus sinensis mrna |  |  |  |
| EY674424 | S44223900 | 28 | 2 | 22.12435 | 2.531148 | -3.127771384 | 0 | protein binding protein |  |  |  |
| EY674438 | S44223914 | 210 | 544 | 165.9326 | 688.4722 | 2.052800862 | 0 | yela protein gb |  |  |  |
| EY674449 | S44223925 | 1388 | 272 | 1096.736 | 344.2361 | -1.671745473 | 0 | protein |  |  |  |
| EY674556 | S44224032 | 394 | 61 | 311.3212 | 77.20001 | -2.011730944 | 0 | �phosphate chloroplastic ame: full= orthophosphate dikinase flags: precursor |  |  |  |
| EY674909 | S44224175 | 14 | 0 | 11.06217 | 0 | -Inf | 0 | phospho-2-dehydro-3-deoxyheptonate tyr-sensitive ame: full=phospho-2-keto-3-deoxyheptonate aldolase ame: full=3-deoxy-d-arabino-heptulosonate 7-phosphate synthase ame: full=dahp synthetase |  |  |  |
| EY674949 | S44224215 | 2 | 12 | 1.580311 | 15.18689 | 3.264546039 | 0 | dibenzothiophene desulfurization enzyme c ame: full=dbt sulfur dioxygenase |  |  |  |
| EY675103 | S44224369 | 81 | 16 | 64.00258 | 20.24918 | -1.660266465 | 0 | peroxidase 4 flags: precursor |  |  |  |
| EY675106 | S44224372 | 1235 | 81 | 975.8419 | 102.5115 | -3.250861786 | 0 | probable xyloglucan endotransglucosylase hydrolase protein 6� |  |  |  |
| EY675153 | S44224419 | 97 | 20 | 76.64507 | 25.31148 | -1.598401209 | 0 | rhomboid protease glup ame: full=intramembrane serine protease |  |  |  |
| EY675187 | S44224453 | 535 | 55 | 422.7331 | 69.60657 | -2.60245183 | 0 | cs00-c1-401-007-c07- sweet orange infected with citrus sinensis mrna |  |  |  |
| EY675218 | S44224484 | 87 | 15 | 68.74352 | 18.98361 | -1.856469362 | 0 | protein |  |  |  |
| EY675348 | S44224614 | 25 | 5 | 19.75388 | 6.32787 | -1.642344557 | 0 | protein |  |  |  |
| EY675370 | S44224636 | 130 | 509 | 102.7202 | 644.1771 | 2.648737571 | 0 | cs00-c1-401-009-f08- sweet orange infected with citrus sinensis mrna |  |  |  |
| EY675507 | S44224773 | 11 | 0 | 8.691709 | 0 | -Inf | 0 | citrus x paradisi miraculin-like protein 2 complete cds |  |  |  |
| EY675558 | S44224824 | 332 | 54 | 262.3316 | 68.34099 | -1.940568391 | 0 | probable pectinesterase pectinesterase inhibitor 54 includes: ame: full=pectinesterase inhibitor 54 ame: full=pectin methylesterase inhibitor 54 includes: ame: full=pectinesterase 54� |  |  |  |
| EY675570 | S44224836 | 1 | 5 | 0.790155 | 6.32787 | 3.001511633 | 0 | rna-binding protein 38 ame: full=rna-binding motif protein 38 ame: full=rna-binding region-containing protein 1 ame: full=hsrnaseb ame: full=ssdna-binding protein seb4 ame: full=cll-associated antigen kw-5 |  |  |  |
| EY675709 | S44224975 | 33 | 5 | 26.07513 | 6.32787 | -2.042882487 | 0 | flavoprotein wrba |  |  |  |
| EY675797 | S44225063 | 387 | 56 | 305.7901 | 70.87214 | -2.109251296 | 0 | protein |  |  |  |
| EY675861 | S44225127 | 8 | 24 | 6.321243 | 30.37377 | 2.264546039 | 0 | cs00-c1-401-016-a01- sweet orange infected with citrus sinensis mrna |  |  |  |
| EY675883 | S44225149 | 2469 | 4919 | 1950.894 | 6225.358 | 1.674021757 | 0 | protein |  |  |  |
| EY675948 | S44225214 | 88 | 10 | 69.53367 | 12.65574 | -2.457919986 | 0 | protein |  |  |  |
| EY676054 | S44225320 | 196 | 29 | 154.8704 | 36.70164 | -2.077145311 | 0 | cs00-c1-401-018-b09- sweet orange infected with citrus sinensis mrna |  |  |  |
| EY676162 | S44225428 | 24 | 48 | 18.96373 | 60.74755 | 1.679583538 | 0 | length cdna complete sequence from clone gsltsil77zb11 of silique of strain col-0 of arabidopsis thaliana (thale cress) |  |  |  |
| EY676297 | S44225563 | 191 | 25 | 150.9197 | 31.63935 | -2.2539891 | 0 | 3-ketoacyl- synthase 10� |  |  |  |
| EY676495 | S44225649 | 2054 | 5532 | 1622.979 | 7001.155 | 2.108948513 | 0 | conserved hypothetical protein [Ricinus communis] |  |  |  |
| EY676654 | S44225808 | 794 | 119 | 627.3833 | 150.6033 | -2.058593896 | 0 | alcohol dehydrogenase 1 |  |  |  |
| EY676719 | S44225873 | 4 | 17 | 3.160621 | 21.51476 | 2.767046379 | 0 | aspartic proteinase nepenthesin-1 ame: full=nepenthesin-i flags: precursor |  |  |  |
| EY676749 | S44225903 | 22 | 1 | 17.38342 | 1.265574 | -3.779848081 | 0 | cs00-c1-401-026-d09- sweet orange infected with citrus sinensis mrna |  |  |  |
| EY676784 | S44225938 | 429 | 42 | 338.9766 | 53.1541 | -2.672932877 | 0 | tsd2 (tumorous shoot development 2) methyltransferase |  |  |  |
| EY677189 | S44226119 | 120 | 13 | 94.81864 | 16.45246 | -2.52686734 | 0 | protein |  |  |  |
| EY677677 | S44226495 | 15 | 2 | 11.85233 | 2.531148 | -2.227307058 | 0 | aquaporin aqpcic |  |  |  |
| EY677751 | S44226569 | 178 | 22 | 140.6477 | 27.84263 | -2.336718274 | 0 | rna polymerase sigma factor rpod |  |  |  |
| EY677909 | S44226727 | 88 | 7 | 69.53367 | 8.859017 | -2.972493159 | 0 | taxadien-5-alpha-ol o-acetyltransferase ame: full=taxa-4 -dien-5alpha-ol-o-acetyltransferase� |  |  |  |
| EY678007 | S44226825 | 1568 | 318 | 1238.964 | 402.4525 | -1.622243351 | 0 | chlorophyll a-b binding protein chloroplastic ame: full=lhcii protein ame: full= flags: precursor |  |  |  |
| EY678013 | S44226831 | 421 | 74 | 332.6554 | 93.65247 | -1.828639519 | 0 | photosystem i reaction center subunit chloroplastic ame: full=photosystem i 20 kda subunit� |  |  |  |
| EY678176 | S44226994 | 329 | 35 | 259.9611 | 44.29509 | -2.553077219 | 0 | serine carboxypeptidase-like 27 flags: precursor |  |  |  |
| EY678390 | S44227096 | 20 | 0 | 15.80311 | 0 | -Inf | 0 | aphanomyces euteiches cdna |  |  |  |
| EY678443 | S44227149 | 3567 | 7644 | 2818.484 | 9674.047 | 1.7792001 | 0 | cs00-c1-401-048-c12- sweet orange infected with citrus sinensis mrna |  |  |  |
| EY678462 | S44227168 | 42 | 2 | 33.18652 | 2.531148 | -3.712733885 | 0 | gdsl esterase lipase at5g45910 ame: full=extracellular lipase at5g45910 flags: precursor |  |  |  |
| EY678592 | S44227298 | 1829 | 242 | 1445.194 | 306.2689 | -2.238392585 | 0 | universal stress protein |  |  |  |
| EY678745 | S44227451 | 18 | 4 | 14.2228 | 5.062296 | -1.490341463 | 0 | cs00-c1-401-031-h10- sweet orange infected with citrus sinensis mrna |  |  |  |
| EY678786 | S44227492 | 5 | 54 | 3.950777 | 68.34099 | 4.112542945 | 0 | superoxide dismutase |  |  |  |
| EY678804 | S44227510 | 1861 | 345 | 1470.479 | 436.623 | -1.75182625 | 0 | probable pectinesterase pectinesterase inhibitor 34 includes: ame: full=pectinesterase inhibitor 34 ame: full=pectin methylesterase inhibitor 34 includes: ame: full=pectinesterase 34� |  |  |  |
| EY678922 | S44227628 | 402 | 80 | 317.6425 | 101.2459 | -1.649540058 | 0 | beta-galactosidase 3� |  |  |  |
| EY679298 | S44227892 | 11 | 2 | 8.691709 | 2.531148 | -1.779848081 | 0 | pmr5 (powdery mildew resistant 5) |  |  |  |
| EY679306 | S44227900 | 3 | 23 | 2.370466 | 29.1082 | 3.618182993 | 0 | predicted protein [Populus trichocarpa] |  |  |  |
| EY679310 | S44227904 | 5 | 23 | 3.950777 | 29.1082 | 2.881217399 | 0 | PREDICTED: hypothetical protein [Vitis vinifera] |  |  |  |
| EY679334 | S44227928 | 85 | 146 | 67.1632 | 184.7738 | 1.460017161 | 0 | probable adp-ribosylation factor gtpase-activating protein agd5� |  |  |  |
| EY679373 | S44227967 | 8 | 18 | 6.321243 | 22.78033 | 1.849508539 | 0 | cs00-c1-401-059-e01- sweet orange infected with citrus sinensis mrna |  |  |  |
| EY679377 | S44227971 | 455 | 67 | 359.5207 | 84.79345 | -2.084050007 | 0 | anthranilate n-benzoyltransferase protein 1 ame: full=anthranilate n-hydroxycinnamoyl benzoyltransferase 1 |  |  |  |
| EY679601 | S44228195 | 8 | 1 | 6.321243 | 1.265574 | -2.320416462 | 0 | cs00-c1-650-002-a04- sweet orange young greenhouse plant citrus sinensis mrna |  |  |  |
| EY679747 | S44228341 | 21 | 2 | 16.59326 | 2.531148 | -2.712733885 | 0 | protein |  |  |  |
| EY679905 | S44228387 | 868 | 31 | 685.8548 | 39.23279 | -4.127771384 | 0 | aquaporin |  |  |  |
| EY680103 | S44228585 | 10 | 1 | 7.901554 | 1.265574 | -2.642344557 | 0 | cs00-c1-650-007-e01- sweet orange young greenhouse plant citrus sinensis mrna |  |  |  |
| EY680870 | S44229128 | 509 | 87 | 402.1891 | 110.1049 | -1.868994812 | 0 | probable mannitol dehydrogenase ame: full=nad-dependent mannitol dehydrogenase |  |  |  |
| EY681003 | S44229261 | 474 | 90 | 374.5336 | 113.9017 | -1.717306615 | 0 | glycine dehydrogenase ame: full=glycine decarboxylase ame: full=glycine cleavage system p-protein |  |  |  |
| EY681849 | S44229771 | 20 | 49 | 15.80311 | 62.01312 | 1.972365287 | 0 | protein |  |  |  |
| EY682772 | S44230470 | 18 | 44 | 14.2228 | 55.68525 | 1.969090155 | 0 | at3g15630 msj11\_3 |  |  |  |
| EY683767 | S44231017 | 199 | 40 | 157.2409 | 50.62296 | -1.635112988 | 0 | cs00-c1-650-040-d10- sweet orange young greenhouse plant citrus sinensis mrna |  |  |  |
| EY683832 | S44231082 | 56 | 153 | 44.2487 | 193.6328 | 2.129616459 | 0 | �zinc metalloprotease slr1821 |  |  |  |
| EY683842 | S44231092 | 259 | 32 | 204.6502 | 40.49837 | -2.33722475 | 0 | cs00-c1-650-041-c06- sweet orange young greenhouse plant citrus sinensis mrna |  |  |  |
| EY683894 | S44231144 | 43 | 80 | 33.97668 | 101.2459 | 1.575246878 | 0 | cs00-c1-650-041-h01- sweet orange young greenhouse plant citrus sinensis mrna |  |  |  |
| EY684257 | S44231171 | 41 | 7 | 32.39637 | 8.859017 | -1.870613545 | 0 | gpi mannosyltransferase 2 ame: full=gpi mannosyltransferase ii� |  |  |  |
| EY684273 | S44231187 | 17 | 30 | 13.43264 | 37.96722 | 1.499011292 | 0 | protein ruptured pollen grain 1 |  |  |  |
| EY684276 | S44231190 | 28 | 51 | 22.12435 | 64.54427 | 1.544653958 | 0 | yth domain family protein 2 |  |  |  |
| EY684404 | S44231318 | 16 | 0 | 12.64249 | 0 | -Inf | 0 | aspartic proteinase nepenthesin-2 ame: full=nepenthesin-ii flags: precursor |  |  |  |
| EY684567 | S44231481 | 171 | 11 | 135.1166 | 13.92131 | -3.278837358 | 0 | Protein E6, putative [Ricinus communis] |  |  |  |
| EY684659 | S44231573 | 426 | 59 | 336.6062 | 74.66886 | -2.172483033 | 0 | amino acid permease 6 ame: full=amino acid transporter aap6 |  |  |  |
| EY684834 | S44231748 | 1 | 2 | 0.790155 | 2.531148 | 1.679583538 | 0 | mads box |  |  |  |
| EY684843 | S44231757 | 933 | 48 | 737.2149 | 60.74755 | -3.601187232 | 0 | monothiol glutaredoxin-s9� |  |  |  |
| EY685091 | S44232005 | 178 | 20 | 140.6477 | 25.31148 | -2.474221798 | 0 | endoglucanase 9 ame: full=endo- -beta glucanase 9 ame: full=cellulase 3� |  |  |  |
| EY685097 | S44232011 | 47 | 6 | 37.1373 | 7.593443 | -2.290042813 | 0 | cs00-c2-003-008-g01- sweet orange greenhouse plant citrus sinensis mrna |  |  |  |
| EY685271 | S44232185 | 290 | 28 | 229.1451 | 35.43607 | -2.69297063 | 0 | vitis vinifera contig whole genome shotgun sequence |  |  |  |
| EY685389 | S44232303 | 550 | 37 | 434.5854 | 46.82623 | -3.214250905 | 0 | cytokinin-o-glucosyltransferase 2 ame: full=zeatin o-glucosyltransferase 2� |  |  |  |
| EY685391 | S44232305 | 32 | 0 | 25.28497 | 0 | -Inf | 0 | cs00-c2-003-018-f09- sweet orange greenhouse plant citrus sinensis mrna |  |  |  |
| EY685437 | S44232351 | 3277 | 5633 | 2589.339 | 7128.978 | 1.461111337 | 0 | cs00-c2-003-019-c01- sweet orange greenhouse plant citrus sinensis mrna |  |  |  |
| EY685494 | S44232408 | 121 | 73 | 95.6088 | 92.3869 | -0.04945514 | 0 | predicted protein [Populus trichocarpa] |  |  |  |
| EY685507 | S44232421 | 6 | 15 | 4.740932 | 18.98361 | 2.001511633 | 0 | protein |  |  |  |
| EY685743 | S44232573 | 96 | 5 | 75.85491 | 6.32787 | -3.583450868 | 0 | peptide transporter ptr1 |  |  |  |
| EY685758 | S44232588 | 296 | 56 | 233.886 | 70.87214 | -1.722514906 | 0 | cs00-c2-003-025-h05- sweet orange greenhouse plant citrus sinensis mrna |  |  |  |
| EY685784 | S44232614 | 18 | 33 | 14.2228 | 41.76394 | 1.554052656 | 0 | protein |  |  |  |
| EY685877 | S44232707 | 51 | 0 | 40.29792 | 0 | -Inf | 0 | gdsl esterase lipase at2g04570 ame: full=extracellular lipase at2g04570 flags: precursor |  |  |  |
| EY685940 | S44232770 | 50 | 8 | 39.50777 | 10.12459 | -1.964272652 | 0 | cs00-c2-003-029-d10- sweet orange greenhouse plant citrus sinensis mrna |  |  |  |
| EY686355 | S44233067 | 20 | 4 | 15.80311 | 5.062296 | -1.642344557 | 0 | cs00-c2-003-042-h11- sweet orange greenhouse plant citrus sinensis mrna |  |  |  |
| EY686394 | S44233106 | 10 | 2 | 7.901554 | 2.531148 | -1.642344557 | 0 | aspartic proteinase asp1� |  |  |  |
| EY686415 | S44233127 | 97 | 14 | 76.64507 | 17.71803 | -2.112974382 | 0 | senescence-associated protein 5-like protein |  |  |  |
| EY686452 | S44233164 | 112 | 14 | 88.4974 | 17.71803 | -2.320416462 | 0 | thaumatin-like protein 1 flags: precursor |  |  |  |
| EY686574 | S44233286 | 9 | 1 | 7.111398 | 1.265574 | -2.490341463 | 0 | cs00-c2-003-055-b07- sweet orange greenhouse plant citrus sinensis mrna |  |  |  |
| EY687123 | S44233387 | 680 | 66 | 537.3056 | 83.52788 | -2.685413279 | 0 | cs00-c2-003-037-f01- sweet orange greenhouse plant citrus sinensis mrna |  |  |  |
| EY687567 | S44233719 | 41 | 7 | 32.39637 | 8.859017 | -1.870613545 | 0 | disease resistance protein |  |  |  |
| EY688092 | S44234048 | 163 | 11 | 128.7953 | 13.92131 | -3.209712998 | 0 | cysteine-rich receptor-like protein kinase 24� |  |  |  |
| EY688198 | S44234154 | 1449 | 533 | 1144.935 | 674.5509 | -0.763266619 | 0 | vitis vinifera contig whole genome shotgun sequence |  |  |  |
| EY688205 | S44234161 | 43 | 7 | 33.97668 | 8.859017 | -1.939326295 | 0 | probable lrr receptor-like serine threonine-protein kinase at4g37250 flags: precursor |  |  |  |
| EY688862 | S44234370 | 99 | 25 | 78.22538 | 31.63935 | -1.305916892 | 0 | cs00-c2-003-065-f01- sweet orange greenhouse plant citrus sinensis mrna |  |  |  |
| EY689397 | S44234905 | 36 | 2 | 28.44559 | 2.531148 | -3.490341463 | 0 | �kda class i heat shock protein 1 ame: full=heat shock protein ame: full=hsp ame: full=heat shock protein 17 ame: full=low molecular weight heat shock protein |  |  |  |
| EY689845 | S44235255 | 24 | 4 | 18.96373 | 5.062296 | -1.905378963 | 0 | protein |  |  |  |
| EY689922 | S44235332 | 15 | 27 | 11.85233 | 34.1705 | 1.527580445 | 0 | cs00-c2-003-082-a08- sweet orange greenhouse plant citrus sinensis mrna |  |  |  |
| EY690201 | S44235499 | 21 | 57 | 16.59326 | 72.13771 | 2.120156129 | 0 | pentatricopeptide repeat-containing protein at2g01860 ame: full=protein embryo defective 975 |  |  |  |
| EY690568 | S44235754 | 106 | 183 | 83.75647 | 231.6 | 1.467362922 | 0 | probable serine threonine-protein kinase at5g41260 |  |  |  |
| EY691566 | S44236752 | 8 | 0 | 6.321243 | 0 | -Inf | 0 | peroxidase 52� |  |  |  |
| EY691980 | S44236844 | 60 | 4 | 47.40932 | 5.062296 | -3.227307058 | 0 | ankyrin-1 ame: full=erythrocyte ankyrin ame: full=ankyrin-r |  |  |  |
| EY692128 | S44236992 | 31 | 0 | 24.49482 | 0 | -Inf | 0 | probable glucan endo- -beta-glucosidase a6 ame: full=(1- |  |  |  |
| EY692203 | S44237067 | 25 | 3 | 19.75388 | 3.796722 | -2.379310151 | 0 | cs00-c2-003-098-b06- sweet orange greenhouse plant citrus sinensis mrna |  |  |  |
| EY692205 | S44237069 | 724 | 145 | 572.0725 | 183.5082 | -1.640353259 | 0 | cs00-c2-003-098-b08- sweet orange greenhouse plant citrus sinensis mrna |  |  |  |
| EY692266 | S44237130 | 70 | 122 | 55.31087 | 154.4 | 1.481037859 | 0 | polynucleotide adenylyltransferase family protein |  |  |  |
| EY692313 | S44237177 | 29 | 49 | 22.91451 | 62.01312 | 1.436312387 | 0 | PREDICTED: hypothetical protein [Vitis vinifera] |  |  |  |
| EY692316 | S44237180 | 59 | 12 | 46.61917 | 15.18689 | -1.618097011 | 0 | protein phosphatase 2c 57� |  |  |  |
| EY692353 | S44237217 | 165 | 393 | 130.3756 | 497.3705 | 1.931646826 | 0 | cs00-c2-003-059-e06- sweet orange greenhouse plant citrus sinensis mrna |  |  |  |
| EY692380 | S44237244 | 444 | 71 | 350.829 | 89.85575 | -1.965085209 | 0 | cs00-c2-003-059-h02- sweet orange greenhouse plant citrus sinensis mrna |  |  |  |
| EY692587 | S44237339 | 147 | 27 | 116.1528 | 34.1705 | -1.765201305 | 0 | probable inactive receptor kinase at5g58300 flags: precursor |  |  |  |
| EY693432 | S44237624 | 30 | 4 | 23.70466 | 5.062296 | -2.227307058 | 0 | probable inactive receptor kinase at5g67200 flags: precursor |  |  |  |
| EY693544 | S44237736 | 253 | 51 | 199.9093 | 64.54427 | -1.630984695 | 0 | probable fkbp-type peptidyl-prolyl cis-trans isomerase chloroplastic� |  |  |  |
| EY693577 | S44237769 | 6 | 164 | 4.740932 | 207.5541 | 5.452173042 | 0 | probable indole-3-acetic acid-amido synthetase ame: full=auxin-responsive gh3-like protein 1� |  |  |  |
| EY693806 | S44237886 | 19 | 34 | 15.01295 | 43.02951 | 1.519118866 | 0 | protein |  |  |  |
| EY693990 | S44238070 | 11 | 2 | 8.691709 | 2.531148 | -1.779848081 | 0 | cs00-c3-700-020-f11- sweet orange development stadium (1 of 6) citrus sinensis mrna |  |  |  |
| EY694299 | S44238281 | 88 | 8 | 69.53367 | 10.12459 | -2.779848081 | 0 | conserved hypothetical protein [Ricinus communis] |  |  |  |
| EY694314 | S44238296 | 15 | 2 | 11.85233 | 2.531148 | -2.227307058 | 0 | e3 ubiquitin-protein ligase rnf181 ame: full=ring finger protein 181 |  |  |  |
| EY694708 | S44238578 | 88 | 16 | 69.53367 | 20.24918 | -1.779848081 | 0 | purple acid phosphatase 15 ame: full=phytase flags: precursor |  |  |  |
| EY695310 | S44238844 | 11 | 2 | 8.691709 | 2.531148 | -1.779848081 | 0 | cs00-c3-700-035-a10- sweet orange development stadium (1 of 6) citrus sinensis mrna |  |  |  |
| EY695365 | S44238899 | 12 | 1 | 9.481864 | 1.265574 | -2.905378963 | 0 | PREDICTED: hypothetical protein [Vitis vinifera] |  |  |  |
| EY695384 | S44238918 | 161 | 307 | 127.215 | 388.5312 | 1.610761505 | 0 | probable protein phosphatase 2c 49� |  |  |  |
| EY695587 | S44239009 | 15 | 2 | 11.85233 | 2.531148 | -2.227307058 | 0 | cs00-c3-700-038-b05- sweet orange development stadium (1 of 6) citrus sinensis mrna |  |  |  |
| EY695672 | S44239094 | 78 | 14 | 61.63212 | 17.71803 | -1.798463759 | 0 | probable lrr receptor-like serine threonine-protein kinase at2g23950 flags: precursor |  |  |  |
| EY695695 | S44239117 | 42 | 3 | 33.18652 | 3.796722 | -3.127771384 | 0 | probable salt tolerance-like protein at1g75540 |  |  |  |
| EY695997 | S44239419 | 39 | 7 | 30.81606 | 8.859017 | -1.798463759 | 0 | uncharacterized basic helix-loop-helix protein at1g06150 |  |  |  |
| EY696120 | S44239542 | 257 | 42 | 203.0699 | 53.1541 | -1.933723588 | 0 | protein |  |  |  |
| EY696242 | S44239664 | 20 | 39 | 15.80311 | 49.35738 | 1.643057662 | 0 | cs00-c3-700-045-d07- sweet orange development stadium (1 of 6) citrus sinensis mrna |  |  |  |
| EY696912 | S44240222 | 15 | 23 | 11.85233 | 29.1082 | 1.296254898 | 0 | protein |  |  |  |
| EY697150 | S44240460 | 34 | 37 | 26.86528 | 46.82623 | 0.801574062 | 0 | protein |  |  |  |
| EY697161 | S44240471 | 297 | 52 | 234.6761 | 65.80984 | -1.834295865 | 0 | fiber protein fb34 |  |  |  |
| EY697171 | S44240481 | 471 | 80 | 372.1632 | 101.2459 | -1.878071617 | 0 | PREDICTED: hypothetical protein [Vitis vinifera] |  |  |  |
| EY697936 | S44241134 | 37 | 2 | 29.23575 | 2.531148 | -3.529869828 | 0 | glyoxylate reductase |  |  |  |
| EY697948 | S44241146 | 14 | 2 | 11.06217 | 2.531148 | -2.127771384 | 0 | cs00-c3-700-066-b12- sweet orange development stadium (1 of 6) citrus sinensis mrna |  |  |  |
| EY697996 | S44241194 | 19 | 2 | 15.01295 | 2.531148 | -2.568343975 | 0 | protein |  |  |  |
| EY698025 | S44241223 | 34 | 0 | 26.86528 | 0 | -Inf | 0 | probable pectinesterase pectinesterase inhibitor 12 includes: ame: full=pectinesterase inhibitor 12 ame: full=pectin methylesterase inhibitor 12 includes: ame: full=pectinesterase 12� |  |  |  |
| EY698899 | S44241873 | 1206 | 243 | 952.9274 | 307.5345 | -1.63161815 | 0 | atp binding |  |  |  |
| EY698945 | S44241919 | 753 | 1452 | 594.987 | 1837.613 | 1.626903221 | 0 | ethylene-responsive transcription factor 4 ame: full=ethylene-responsive element-binding factor 4 homolog ame: full=ethylene-responsive element-binding factor 3� |  |  |  |
| EY699143 | S44242117 | 6 | 18 | 4.740932 | 22.78033 | 2.264546039 | 0 | �ac transposase ame: full=orfa |  |  |  |
| EY699314 | S44242176 | 214 | 37 | 169.0932 | 46.82623 | -1.852430083 | 0 | homeobox-leucine zipper protein athb-6 ame: full=homeodomain transcription factor athb-6 ame: full=hd-zip protein athb-6 |  |  |  |
| EY699454 | S44242316 | 191 | 22 | 150.9197 | 27.84263 | -2.438413671 | 0 | hypothetical protein [Vitis vinifera] |  |  |  |
| EY699654 | S44242516 | 11 | 2 | 8.691709 | 2.531148 | -1.779848081 | 0 | �cyclin-d6-1 ame: full=g1 s-specific cyclin-d6-1� |  |  |  |
| EY699859 | S44242721 | 88 | 4 | 69.53367 | 5.062296 | -3.779848081 | 0 | thymidine kinase |  |  |  |
| EY700158 | S44243020 | 32 | 54 | 25.28497 | 68.34099 | 1.43447104 | 0 | cs00-c3-700-091-c07- sweet orange development stadium (1 of 6) citrus sinensis mrna |  |  |  |
| EY700858 | S44243496 | 345 | 816 | 272.6036 | 1032.708 | 1.921556328 | 0 | protein |  |  |  |
| EY701256 | S44243782 | 429 | 50 | 338.9766 | 63.2787 | -2.42139411 | 0 | chlorophyll a-b binding protein chloroplastic ame: full=lhci type iii cab-p4 flags: precursor |  |  |  |
| EY701281 | S44243807 | 21 | 3 | 16.59326 | 3.796722 | -2.127771384 | 0 | probable lrr receptor-like serine threonine-protein kinase at5g45780 flags: precursor |  |  |  |
| EY701288 | S44243814 | 3530 | 446 | 2789.248 | 564.446 | -2.30496903 | 0 | rcc1 domain-containing protein 1 |  |  |  |
| EY701378 | S44243904 | 131 | 21 | 103.5104 | 26.57705 | -1.961522041 | 0 | uncharacterized membrane protein at1g16860 |  |  |  |
| EY701437 | S44243963 | 83 | 9 | 65.58289 | 11.39017 | -2.525530892 | 0 | cs00-c3-700-106-d04- sweet orange development stadium (1 of 6) citrus sinensis mrna |  |  |  |
| EY701476 | S44244002 | 526 | 623 | 415.6217 | 788.4525 | 0.923752902 | 0 | cs00-c3-700-106-g10- sweet orange development stadium (1 of 6) citrus sinensis mrna |  |  |  |
| EY701685 | S44244211 | 264 | 17 | 208.601 | 21.51476 | -3.27734774 | 0 | protein |  |  |  |
| EY701822 | S44244348 | 578 | 1485 | 456.7098 | 1879.377 | 2.040905071 | 0 | protein |  |  |  |
| EY702045 | S44244571 | 60 | 4 | 47.40932 | 5.062296 | -3.227307058 | 0 | early nodulin-like protein 3 ame: full=phytocyanin-like protein flags: precursor |  |  |  |
| EY702521 | S44244935 | 106 | 12 | 83.75647 | 15.18689 | -2.463374416 | 0 | epidermis-specific secreted glycoprotein ep1 ame: full=52 54 kda medium protein flags: precursor |  |  |  |
| EY703257 | S44245559 | 262 | 45 | 207.0207 | 56.95083 | -1.861986367 | 0 | glycosyltransferase 8 domain-containing protein 1 |  |  |  |
| EY703258 | S44245560 | 120 | 13 | 94.81864 | 16.45246 | -2.52686734 | 0 | serine carboxypeptidase-like 26 flags: precursor |  |  |  |
| EY703774 | S44245852 | 2006 | 3398 | 1585.052 | 4300.42 | 1.439947785 | 0 | ring-h2 finger protein atl5h flags: precursor |  |  |  |
| EY704159 | S44246237 | 105 | 150 | 82.96631 | 189.8361 | 1.194156711 | 0 | cs00-c3-701-029-g12- sweet orange development stadium (2 of 6) citrus sinensis mrna |  |  |  |
| EY704314 | S44246392 | 4 | 11 | 3.160621 | 13.92131 | 2.139015157 | 0 | trehalose-phosphate phosphatase |  |  |  |
| EY704798 | S44246764 | 38 | 73 | 30.0259 | 92.3869 | 1.621480583 | 0 | nudix hydrolase 10� |  |  |  |
| EY704814 | S44246780 | 16 | 2 | 12.64249 | 2.531148 | -2.320416462 | 0 | probable pectinesterase 29� |  |  |  |
| EY704864 | S44246830 | 3948 | 702 | 3119.533 | 888.4329 | -1.811995516 | 0 | pre-mrna splicing factor prp38 family protein |  |  |  |
| EY706135 | S44247877 | 904 | 133 | 714.3004 | 168.3213 | -2.085312989 | 0 | chlorophyll a-b binding protein chloroplastic ame: full=lhcii type iii cab-13 flags: precursor |  |  |  |
| EY706436 | S44248178 | 84 | 168 | 66.37305 | 212.6164 | 1.679583538 | 0 | ethylene-responsive transcription factor 2� |  |  |  |
| EY706443 | S44248185 | 63 | 7 | 49.77979 | 8.859017 | -2.490341463 | 0 | chavicol o-methyltransferase ame: full= eugenol o-methyltransferase cvomt1 ame: full=s-adenosysl-l-methionine: eugenol o-methyltransferase cvomt1 |  |  |  |
| EY706479 | S44248221 | 1879 | 291 | 1484.702 | 368.282 | -2.011290471 | 0 | probable polygalacturonase� |  |  |  |
| EY706514 | S44248256 | 465 | 63 | 367.4222 | 79.73116 | -2.204223445 | 0 | snakin-1 flags: precursor |  |  |  |
| EY706709 | S44248451 | 34 | 2 | 26.86528 | 2.531148 | -3.407879303 | 0 | protein |  |  |  |
| EY707122 | S44248864 | 18 | 0 | 14.2228 | 0 | -Inf | 0 | dehydration-responsive family protein |  |  |  |
| EY707123 | S44248865 | 51 | 121 | 40.29792 | 153.1344 | 1.926021433 | 0 | at1g03610 f21b7\_13 |  |  |  |
| EY707388 | S44249130 | 41 | 4 | 32.39637 | 5.062296 | -2.677968467 | 0 | cs00-c3-701-068-b05- sweet orange development stadium (2 of 6) citrus sinensis mrna |  |  |  |
| EY708121 | S44249751 | 553 | 68 | 436.9559 | 86.05903 | -2.344089291 | 0 | tubulin beta-2 chain ame: full=beta-2-tubulin |  |  |  |
| EY708222 | S44249852 | 99 | 9 | 78.22538 | 11.39017 | -2.779848081 | 0 | predicted protein [Populus trichocarpa] |  |  |  |
| EY708401 | S44250031 | 214 | 29 | 169.0932 | 36.70164 | -2.203902453 | 0 | dna-damage-inducible protein f |  |  |  |
| EY708789 | S44250209 | 193 | 32 | 152.5 | 40.49837 | -1.912873499 | 0 | cytochrome p450 98a2 |  |  |  |
| EY709175 | S44250384 | 276 | 127 | 218.0829 | 160.7279 | -0.440256232 | 0 | cs00-c3-701-109-b03- sweet orange development stadium (2 of 6) citrus sinensis mrna |  |  |  |
| EY709631 | S44250728 | 31 | 5 | 24.49482 | 6.32787 | -1.952684678 | 0 | cs00-c3-701-048-d11- sweet orange development stadium (2 of 6) citrus sinensis mrna |  |  |  |
| EY709806 | S44250903 | 212 | 41 | 167.5129 | 51.88853 | -1.690784912 | 0 | uncharacterized gmc-type oxidoreductase y4nj |  |  |  |
| EY709959 | S44251056 | 895 | 177 | 707.189 | 224.0066 | -1.658554784 | 0 | probable polygalacturonase� |  |  |  |
| EY709989 | S44251086 | 864 | 95 | 682.6942 | 120.2295 | -2.505448356 | 0 | aquaporin pip2-7 ame: full=plasma membrane intrinsic protein 2-7� |  |  |  |
| EY710037 | S44251134 | 32050 | 5165 | 25324.48 | 6536.689 | -1.953900565 | 0 | germin-like protein subfamily 1 member 8 flags: precursor |  |  |  |
| EY710412 | S44251285 | 3604 | 629 | 2847.72 | 796.046 | -1.838883551 | 0 | protein |  |  |  |
| EY710505 | S44251378 | 39 | 6 | 30.81606 | 7.593443 | -2.02085618 | 0 | �upf0481 protein at3g02645 |  |  |  |
| EY710516 | S44251389 | 613 | 85 | 484.3652 | 107.5738 | -2.17076879 | 0 | cytokinin-n-glucosyltransferase 1 ame: full=udp-glucosyl transferase 76c1 |  |  |  |
| EY710598 | S44251471 | 676 | 106 | 534.145 | 134.1508 | -1.993375444 | 0 | ribulose bisphosphate carboxylase oxygenase activase chloroplastic� |  |  |  |
| EY710613 | S44251486 | 100 | 19 | 79.01554 | 24.0459 | -1.716345138 | 0 | 1-acyl-sn-glycerol-3-phosphate acyltransferase� |  |  |  |
| EY710672 | S44251545 | 84 | 12 | 66.37305 | 15.18689 | -2.127771384 | 0 | f-box family protein |  |  |  |
| EY710689 | S44251562 | 83 | 5 | 65.58289 | 6.32787 | -3.373527799 | 0 | l-ascorbate oxidase homolog ame: full=pollen-specific protein ntp303 flags: precursor |  |  |  |
| EY710962 | S44251723 | 32305 | 3362 | 25525.97 | 4254.86 | -2.584782307 | 0 | nitrate transporter ame: full=nitrate permease |  |  |  |
| EY711087 | S44251848 | 30231 | 1404 | 23887.19 | 1776.866 | -3.748830323 | 0 | small heat shock chloroplastic flags: precursor |  |  |  |
| EY711961 | S44252288 | 14 | 25 | 11.06217 | 31.63935 | 1.516084806 | 0 | cs00-c3-702-013-g06- sweet orange development stadium (3 of 6) citrus sinensis mrna |  |  |  |
| EY711963 | S44252290 | 22 | 0 | 17.38342 | 0 | -Inf | 0 | ethylene-responsive transcription factor erf034 |  |  |  |
| EY712436 | S44252763 | 81 | 5 | 64.00258 | 6.32787 | -3.33833837 | 0 | cs00-c3-702-019-c10- sweet orange development stadium (3 of 6) citrus sinensis mrna |  |  |  |
| EY712805 | S44252908 | 34 | 5 | 26.86528 | 6.32787 | -2.085951208 | 0 | cs00-c3-702-023-f07- sweet orange development stadium (3 of 6) citrus sinensis mrna |  |  |  |
| EY713583 | S44253350 | 5759 | 888 | 4550.505 | 1123.83 | -2.017603202 | 0 | glucoamylase ame: full=glucan -alpha-glucosidase ame: full= -alpha-d-glucan glucohydrolase flags: precursor |  |  |  |
| EY713903 | S44253558 | 91 | 2 | 71.90414 | 2.531148 | -4.828211102 | 0 | anthocyanidin 3-o-glucosyltransferase ame: full=flavonol 3-o-glucosyltransferase ame: full=udp-glucose flavonoid 3-o-glucosyltransferase ame: full=anthocyanin rhamnosyl transferase |  |  |  |
| EY714406 | S44253837 | 17 | 42 | 13.43264 | 53.1541 | 1.984438119 | 0 | cs00-c3-702-042-b02- sweet orange development stadium (3 of 6) citrus sinensis mrna |  |  |  |
| EY714464 | S44253895 | 17 | 33 | 13.43264 | 41.76394 | 1.636514816 | 0 | cs00-c3-702-042-g04- sweet orange development stadium (3 of 6) citrus sinensis mrna |  |  |  |
| EY714594 | S44254025 | 63 | 10 | 49.77979 | 12.65574 | -1.975768291 | 0 | auxin-induced protein 15a |  |  |  |
| EY716101 | S44254748 | 526 | 1486 | 415.6217 | 1880.643 | 2.177882949 | 0 | predicted protein [Populus trichocarpa] |  |  |  |
| EY716580 | S44255003 | 1090 | 121 | 861.2693 | 153.1344 | -2.491665644 | 0 | poncirus trifoliata citrus tristeza virus resistance gene complete sequence |  |  |  |
| EY717821 | S44255698 | 33 | 5 | 26.07513 | 6.32787 | -2.042882487 | 0 | wpp domain-associated protein |  |  |  |
| EY718514 | S44256083 | 67 | 9 | 52.94041 | 11.39017 | -2.216580651 | 0 | cs00-c3-702-100-c01- sweet orange development stadium (3 of 6) citrus sinensis mrna |  |  |  |
| EY718617 | S44256186 | 265 | 387 | 209.3912 | 489.7771 | 1.225924745 | 0 | cytochrome p450 71a19 |  |  |  |
| EY718638 | S44256207 | 624 | 1235 | 493.0569 | 1562.984 | 1.664476646 | 0 | f-box kelch-repeat protein at2g44130 |  |  |  |
| EY718737 | S44256300 | 70 | 14 | 55.31087 | 17.71803 | -1.642344557 | 0 | dna binding |  |  |  |
| EY719020 | S44256583 | 577 | 2329 | 455.9196 | 2947.522 | 2.692650954 | 0 | myb-like protein g |  |  |  |
| EY719394 | S44256859 | 1169 | 177 | 923.6916 | 224.0066 | -2.043870126 | 0 | chlorophyll a-b binding protein chloroplastic ame: full=light-harvesting complex ii protein 5 ame: full=lhcb5 ame: full=lhciic flags: precursor |  |  |  |
| EY719416 | S44256881 | 56 | 11 | 44.2487 | 13.92131 | -1.668339765 | 0 | epimerase family protein slr1223 |  |  |  |
| EY719455 | S44256920 | 29 | 71 | 22.91451 | 89.85575 | 1.971349662 | 0 | cs00-c3-702-085-e04- sweet orange development stadium (3 of 6) citrus sinensis mrna |  |  |  |
| EY719478 | S44256943 | 20 | 39 | 15.80311 | 49.35738 | 1.643057662 | 0 | zinc finger protein magpie |  |  |  |
| EY719507 | S44256972 | 52762 | 7544 | 41690.18 | 9547.49 | -2.126514133 | 0 | heat shock cognate 70 kda protein 1� |  |  |  |
| EY721084 | S44258319 | 77 | 12 | 60.84196 | 15.18689 | -2.002240502 | 0 | pentatricopeptide repeat-containing protein at2g35130 |  |  |  |
| EY722387 | S44259286 | 32 | 6 | 25.28497 | 7.593443 | -1.735453961 | 0 | uncharacterized mitochondrial protein g00310 ame: full=orf154 |  |  |  |
| EY722769 | S44259332 | 10 | 30 | 7.901554 | 37.96722 | 2.264546039 | 0 | populus trichocarpa mrna |  |  |  |
| EY722898 | S44259461 | 50 | 1 | 39.50777 | 1.265574 | -4.964272652 | 0 | pseudomonas fluorescens sbw25 complete genome |  |  |  |
| EY722929 | S44259492 | 5 | 0 | 3.950777 | 0 | -Inf | 0 | 60 kda chaperonin ame: full=protein cpn60 ame: full=groel protein |  |  |  |
| EY723197 | S44259760 | 215 | 23 | 169.8834 | 29.1082 | -2.545047356 | 0 | heavy metal-associated domain containing expressed |  |  |  |
| EY723199 | S44259762 | 15 | 27 | 11.85233 | 34.1705 | 1.527580445 | 0 | leucine-rich repeat |  |  |  |
| EY723222 | S44259785 | 271 | 59 | 214.1321 | 74.66886 | -1.519922454 | 0 | protein srg1� |  |  |  |
| EY723223 | S44259786 | 6395 | 1091 | 5053.043 | 1380.741 | -1.871709719 | 0 | predicted protein [Populus trichocarpa] |  |  |  |
| EY723237 | S44259800 | 30 | 2 | 23.70466 | 2.531148 | -3.227307058 | 0 | linear gramicidin synthetase subunit d includes: ame: full=atp-dependent tryptophan adenylase� |  |  |  |
| EY723878 | S44260343 | 47 | 10 | 37.1373 | 12.65574 | -1.553077219 | 0 | cs00-c3-703-064-f03- sweet orange development stadium (4 of 6) citrus sinensis mrna |  |  |  |
| EY724015 | S44260480 | 1558 | 302 | 1231.062 | 382.2033 | -1.687491241 | 0 | cs00-c3-703-066-d09- sweet orange development stadium (4 of 6) citrus sinensis mrna |  |  |  |
| EY724078 | S44260543 | 40 | 82 | 31.60621 | 103.7771 | 1.715207448 | 0 | phospholipase a2 homolog 3 flags: precursor |  |  |  |
| EY725089 | S44261218 | 421 | 103 | 332.6554 | 130.3541 | -1.351592358 | 0 | �endonuclease flj39025 homolog |  |  |  |
| EY725739 | S44261652 | 56 | 5 | 44.2487 | 6.32787 | -2.805843289 | 0 | probable lrr receptor-like serine threonine-protein kinase at4g08850 flags: precursor |  |  |  |
| EY726389 | S44262299 | 3395 | 638 | 2682.577 | 807.4362 | -1.732199707 | 0 | upf0717 protein at5g11950 |  |  |  |
| EY727291 | S44262641 | 95 | 14 | 75.06476 | 17.71803 | -2.082917148 | 0 | xyloglucan endotransglucosylase hydrolase protein 9� |  |  |  |
| EY727481 | S44262831 | 25 | 48 | 19.75388 | 60.74755 | 1.620689849 | 0 | conserved hypothetical protein [Ricinus communis] |  |  |  |
| EY727509 | S44262859 | 1046 | 179 | 826.5025 | 226.5377 | -1.867267821 | 0 | tsd2 (tumorous shoot development 2) methyltransferase |  |  |  |
| EY727538 | S44262888 | 13 | 18 | 10.27202 | 22.78033 | 1.149068821 | 0 | cs00-c3-703-102-e10- sweet orange development stadium (4 of 6) citrus sinensis mrna |  |  |  |
| EY727543 | S44262893 | 4378 | 657 | 3459.3 | 831.4821 | -2.056723141 | 0 | protein |  |  |  |
| EY727967 | S44262981 | 45 | 9 | 35.55699 | 11.39017 | -1.642344557 | 0 | probable pectinesterase pectinesterase inhibitor 54 includes: ame: full=pectinesterase inhibitor 54 ame: full=pectin methylesterase inhibitor 54 includes: ame: full=pectinesterase 54� |  |  |  |
| EY728050 | S44263064 | 11 | 2 | 8.691709 | 2.531148 | -1.779848081 | 0 | cs00-c3-703-109-h04- sweet orange development stadium (4 of 6) citrus sinensis mrna |  |  |  |
| EY728053 | S44263067 | 1237 | 219 | 977.4222 | 277.1607 | -1.818259187 | 0 | protein wax2 ame: full=protein yore-yore ame: full=protein faceless pollen 1 |  |  |  |
| EY728071 | S44263085 | 495 | 73 | 391.1269 | 92.3869 | -2.081876618 | 0 | protein |  |  |  |
| EY728149 | S44263163 | 39 | 8 | 30.81606 | 10.12459 | -1.605818681 | 0 | cs00-c3-703-108-b05- sweet orange development stadium (4 of 6) citrus sinensis mrna |  |  |  |
| EY728404 | S44263306 | 48 | 2 | 37.92746 | 2.531148 | -3.905378963 | 0 | gdsl esterase lipase at5g42170 ame: full=extracellular lipase at5g42170 flags: precursor |  |  |  |
| EY728617 | S44263519 | 30 | 53 | 23.70466 | 67.07542 | 1.500613397 | 0 | polyphosphoinositide phosphatase ame: full=phosphatidylinositol -bisphosphate 5-phosphatase ame: full=factor-induced gene 4 protein |  |  |  |
| EY729003 | S44263793 | 19 | 3 | 15.01295 | 3.796722 | -1.983381475 | 0 | cs00-c3-704-011-d02- sweet orange development stadium (5 of 6) citrus sinensis mrna |  |  |  |
| EY729053 | S44263843 | 1435 | 89 | 1133.873 | 112.6361 | -3.331518053 | 0 | hypothetical protein [Vitis vinifera] |  |  |  |
| EY730117 | S44264683 | 18 | 68 | 14.2228 | 86.05903 | 2.597121378 | 0 | f-box and wd40 domain |  |  |  |
| EY731213 | S44265331 | 10 | 18 | 7.901554 | 22.78033 | 1.527580445 | 0 | �ferric transport system permease protein fbpb |  |  |  |
| EY731619 | S44265737 | 12764 | 2375 | 10085.54 | 3005.738 | -1.746497557 | 0 | heat shock protein 101 |  |  |  |
| EY731734 | S44265852 | 17 | 29 | 13.43264 | 36.70164 | 1.450101692 | 0 | zinc finger cchc-type and rna-binding motif-containing protein 1 ame: full=u11 u12 small nuclear ribonucleoprotein 31 kda protein� |  |  |  |
| EY733084 | S44267090 | 9 | 2 | 7.111398 | 2.531148 | -1.490341463 | 0 | cs00-c3-704-071-g02- sweet orange development stadium (5 of 6) citrus sinensis mrna |  |  |  |
| EY733167 | S44267173 | 16 | 30 | 12.64249 | 37.96722 | 1.586474134 | 0 | chromatin remodelling complex atpase chain isw- |  |  |  |
| EY734089 | S44267893 | 5 | 11 | 3.950777 | 13.92131 | 1.817087062 | 0 | cs00-c3-704-081-d10- sweet orange development stadium (5 of 6) citrus sinensis mrna |  |  |  |
| EY734173 | S44267977 | 31 | 6 | 24.49482 | 7.593443 | -1.689650272 | 0 | cs00-c3-704-100-g10- sweet orange development stadium (5 of 6) citrus sinensis mrna |  |  |  |
| EY734701 | S44268393 | 6 | 1 | 4.740932 | 1.265574 | -1.905378963 | 0 | cs00-c3-704-098-c02- sweet orange development stadium (5 of 6) citrus sinensis mrna |  |  |  |
| EY734757 | S44268449 | 18 | 3 | 14.2228 | 3.796722 | -1.905378963 | 0 | cs00-c3-704-087-b05- sweet orange development stadium (5 of 6) citrus sinensis mrna |  |  |  |
| EY734818 | S44268510 | 58 | 52 | 45.82901 | 65.80984 | 0.522042261 | 0 | cs00-c3-704-103-c04- sweet orange development stadium (5 of 6) citrus sinensis mrna |  |  |  |
| EY735096 | S44268564 | 940 | 188 | 742.746 | 237.9279 | -1.642344557 | 0 | uncharacterised protein at1g10890 |  |  |  |
| EY735515 | S44268759 | 5 | 10 | 3.950777 | 12.65574 | 1.679583538 | 0 | cs00-c3-704-090-d10- sweet orange development stadium (5 of 6) citrus sinensis mrna |  |  |  |
| EY737358 | S44270148 | 103 | 19 | 81.386 | 24.0459 | -1.758989476 | 0 | �carrier mitochondrial ame: full=adp atp translocase ame: full=adenine nucleotide translocator� |  |  |  |
| EY741985 | S44273669 | 17 | 35 | 13.43264 | 44.29509 | 1.721403714 | 0 | photosystem i assembly protein ycf4 |  |  |  |
| EY742616 | S44274076 | 241 | 27 | 190.4274 | 34.1705 | -2.478418296 | 0 | lob domain-containing protein 4 ame: full=asymmetric leaves 2-like protein 6� |  |  |  |
| EY742851 | S44274199 | 3583 | 542 | 2831.127 | 685.9411 | -2.045219749 | 0 | (+)-delta-cadinene synthase isozyme a� |  |  |  |
| EY742871 | S44274219 | 187 | 29 | 147.7591 | 36.70164 | -2.009329927 | 0 | major allergen pru av 1 ame: full=allergen pru a 1 ame: allergen=pru av 1 |  |  |  |
| EY742919 | S44274267 | 21 | 4 | 16.59326 | 5.062296 | -1.712733885 | 0 | cs00-c3-705-094-g07- sweet orange development stadium (6 of 6) citrus sinensis mrna |  |  |  |
| EY743232 | S44274468 | 1259 | 1002 | 994.8056 | 1268.105 | 0.350187763 | 0 | cs00-c3-705-101-f11- sweet orange development stadium (6 of 6) citrus sinensis mrna |  |  |  |
| EY743988 | S44274664 | 316 | 42 | 249.6891 | 53.1541 | -2.231879787 | 0 | transcription factor bhlh48 ame: full=transcription factor en 97 ame: full=bhlh transcription factor bhlh048 ame: full=basic helix-loop-helix protein 48� |  |  |  |
| EY744205 | S44274881 | 31 | 2 | 24.49482 | 2.531148 | -3.274612772 | 0 | gdsl esterase lipase at5g45670 ame: full=extracellular lipase at5g45670 flags: precursor |  |  |  |
| EY744415 | S44275091 | 1359 | 154 | 1073.821 | 194.8984 | -2.461959662 | 0 | anthocyanin 5-aromatic acyltransferase� |  |  |  |
| EY744610 | S44275286 | 182 | 15 | 143.8083 | 18.98361 | -2.921320507 | 0 | protein |  |  |  |
| EY745020 | S44275598 | 5 | 27 | 3.950777 | 34.1705 | 3.112542945 | 0 | dna binding protein |  |  |  |
| EY745112 | S44275690 | 113 | 187 | 89.28755 | 236.6623 | 1.406299035 | 0 | calcium dependent protein kinase 32 |  |  |  |
| EY745499 | S44276077 | 27 | 4 | 21.33419 | 5.062296 | -2.075303964 | 0 | PREDICTED: hypothetical protein isoform 2 [Vitis vinifera] |  |  |  |
| EY745902 | S44276377 | 35 | 4 | 27.65544 | 5.062296 | -2.449699479 | 0 | conserved hypothetical protein [Ricinus communis] |  |  |  |
| EY746830 | S44276745 | 9764 | 1467 | 7715.077 | 1856.597 | -2.055019886 | 0 | heat shock 22 kda mitochondrial flags: precursor |  |  |  |
| EY746896 | S44276811 | 6114 | 1100 | 4831.01 | 1392.131 | -1.795029491 | 0 | udp-glucuronate 4-epimerase 6 ame: full=udp-glucuronic acid epimerase 6� |  |  |  |
| EY746898 | S44276813 | 17 | 0 | 13.43264 | 0 | -Inf | 0 | transcription factor myb39 ame: full=myb-related protein 39� |  |  |  |
| EY746939 | S44276854 | 446 | 1523 | 352.4093 | 1927.469 | 2.451383865 | 0 | calcium-dependent protein kinase sk5� |  |  |  |
| EY747045 | S44276960 | 692 | 58 | 546.7875 | 73.40329 | -2.897063695 | 0 | miraculin� |  |  |  |
| EY747051 | S44276966 | 12 | 2 | 9.481864 | 2.531148 | -1.905378963 | 0 | human dna sequence from clone xxyac-68g7 on chromosome 9 contains the 3 end of the secisbp2 gene for secis binding protein the gene for a novel protein and the 3 end of the sema4d gene for sema immunoglobulin domain transmembrane domain and short cytoplasmic complete sequence |  |  |  |
| EY747486 | S44277177 | 6 | 10 | 4.740932 | 12.65574 | 1.416549132 | 0 | cs00-c5-003-045-f04- sweet orange greenhouse plant citrus sinensis mrna |  |  |  |
| EY747508 | S44277199 | 2310 | 445 | 1825.259 | 563.1804 | -1.696432073 | 0 | at5g51550 k17n15\_10 |  |  |  |
| EY747531 | S44277222 | 253 | 39 | 199.9093 | 49.35738 | -2.018007818 | 0 | f-box kelch-repeat protein at5g43190 |  |  |  |
| EY747559 | S44277250 | 7 | 0 | 5.531087 | 0 | -Inf | 0 | inositol oxygenase 1 ame: full=myo-inositol oxygenase 1� |  |  |  |
| EY747562 | S44277253 | 46 | 8 | 36.34715 | 10.12459 | -1.843978418 | 0 | bifunctional enzyme lpxc fabz includes: ame: full=udp-3-o- dehydratase� |  |  |  |
| EY747587 | S44277278 | 3 | 4 | 2.370466 | 5.062296 | 1.094621037 | 0 | benzoate carboxyl methyltransferase ame: full=s-adenosyl-l-methionine:benzoic acid carboxyl methyltransferase |  |  |  |
| EY747704 | S44277395 | 114 | 9 | 90.07771 | 11.39017 | -2.983381475 | 0 | dna binding protein |  |  |  |
| EY748104 | S44277571 | 11 | 18 | 8.691709 | 22.78033 | 1.390076921 | 0 | cs00-c5-003-053-e07- sweet orange greenhouse plant citrus sinensis mrna |  |  |  |
| EY748266 | S44277733 | 43 | 11 | 33.97668 | 13.92131 | -1.287249598 | 0 | volvox carteri nagariensis mrna for pherophorin-dz1 protein |  |  |  |
| EY748923 | S44278054 | 195 | 35 | 154.0803 | 44.29509 | -1.798463759 | 0 | cs00-c5-003-064-b03- sweet orange greenhouse plant citrus sinensis mrna |  |  |  |
| EY749485 | S44278294 | 132 | 24 | 104.3005 | 30.37377 | -1.779848081 | 0 | novel plant |  |  |  |
| EY749614 | S44278423 | 92 | 149 | 72.69429 | 188.5705 | 1.375190102 | 0 | plastid alpha-amylase |  |  |  |
| EY749926 | S44278511 | 230 | 35 | 181.7357 | 44.29509 | -2.036623496 | 0 | auxin transporter protein 1 ame: full=auxin influx carrier protein 1 ame: full=polar auxin transport inhibitor-resistant protein 1 |  |  |  |
| EY749940 | S44278525 | 43 | 0 | 33.97668 | 0 | -Inf | 0 | tubulin alpha-2 chain ame: full=alpha-2-tubulin |  |  |  |
| EY750025 | S44278610 | 19 | 3 | 15.01295 | 3.796722 | -1.983381475 | 0 | 14 kda proline-rich protein flags: precursor |  |  |  |
| EY750256 | S44278841 | 36 | 78 | 28.44559 | 98.71477 | 1.795060755 | 0 | monoglyceride lipase� |  |  |  |
| EY750681 | S44278930 | 3 | 11 | 2.370466 | 13.92131 | 2.554052656 | 0 | sigma factor sigb regulation protein rsbq |  |  |  |
| EY750798 | S44279047 | 625 | 68 | 493.8471 | 86.05903 | -2.520666 | 0 | tubulin beta-8 chain ame: full=beta-8-tubulin |  |  |  |
| EY750803 | S44279052 | 5136 | 684 | 4058.238 | 865.6526 | -2.228993434 | 0 | l-ascorbate oxidase homolog flags: precursor |  |  |  |
| EY750920 | S44279169 | 5 | 0 | 3.950777 | 0 | -Inf | 0 | cs00-c5-003-101-f12- sweet orange greenhouse plant citrus sinensis mrna |  |  |  |
| EY750970 | S44279219 | 175 | 20 | 138.2772 | 25.31148 | -2.449699479 | 0 | cs00-c5-003-102-c07- sweet orange greenhouse plant citrus sinensis mrna |  |  |  |
| EY750982 | S44279231 | 272 | 21 | 214.9223 | 26.57705 | -3.015561881 | 0 | gdsl esterase lipase at3g27950 ame: full=extracellular lipase at3g27950 flags: precursor |  |  |  |
| EY751088 | S44279331 | 22 | 393 | 17.38342 | 497.3705 | 4.838537422 | 0 | non-specific lipid-transfer protein� |  |  |  |
| EY751241 | S44279484 | 30 | 2 | 23.70466 | 2.531148 | -3.227307058 | 0 | uncharacterized protein at5g12080 |  |  |  |
| EY751281 | S44279524 | 10 | 21 | 7.901554 | 26.57705 | 1.749972866 | 0 | protein |  |  |  |
| EY751507 | S44279750 | 10 | 1 | 7.901554 | 1.265574 | -2.642344557 | 0 | chemocyanin ame: full=basic blue protein ame: full=plantacyanin flags: precursor |  |  |  |
| EY751767 | S44279898 | 36 | 94 | 28.44559 | 118.9639 | 2.064247388 | 0 | �cysteine-rich receptor-like protein kinase 20� |  |  |  |
| EY751835 | S44279966 | 25 | 0 | 19.75388 | 0 | -Inf | 0 | at3g16570 mgl6\_2 |  |  |  |
| EY752242 | S44280261 | 3 | 18 | 2.370466 | 22.78033 | 3.264546039 | 0 | photoassimilate-responsive protein par-like protein |  |  |  |
| EY752431 | S44280444 | 16 | 2 | 12.64249 | 2.531148 | -2.320416462 | 0 | probable xyloglucan endotransglucosylase hydrolase flags: precursor |  |  |  |
| EY752497 | S44280510 | 96 | 18 | 75.85491 | 22.78033 | -1.735453961 | 0 | 5ng4\_pintaauxin-induced protein 5ng4 |  |  |  |
| EY752572 | S44280585 | 164 | 320 | 129.5855 | 404.9837 | 1.643959628 | 0 | protein |  |  |  |
| EY752900 | S44280829 | 531 | 114 | 419.5725 | 144.2754 | -1.540094499 | 0 | protein |  |  |  |
| EY752964 | S44280893 | 807 | 162 | 637.6554 | 205.023 | -1.636991322 | 0 | peroxidase 15� |  |  |  |
| EY753208 | S44281025 | 168 | 327 | 132.7461 | 413.8427 | 1.640412941 | 0 | cs00-c5-003-083-b09- sweet orange greenhouse plant citrus sinensis mrna |  |  |  |
| EY753520 | S44281337 | 60 | 13 | 47.40932 | 16.45246 | -1.52686734 | 0 | serine threonine-protein kinase bri1-like 2 ame: full=brassinosteroid insensitive 1-like protein 2 ame: full=protein vascular highway 1 flags: precursor |  |  |  |
| EY753771 | S44281588 | 40 | 76 | 31.60621 | 96.18362 | 1.605582957 | 0 | protein kinase |  |  |  |
| EY753801 | S44281618 | 254 | 526 | 200.6995 | 665.6919 | 1.72981784 | 0 | protein |  |  |  |
| EY754929 | S44282214 | 14 | 33 | 11.06217 | 41.76394 | 1.916622735 | 0 | purple acid phosphatase 17 ame: full=acid phosphatase type 5 ame: full=peroxidase flags: precursor |  |  |  |
| EY755529 | S44282366 | 311 | 24 | 245.7383 | 30.37377 | -3.016224731 | 0 | predicted protein [Populus trichocarpa] |  |  |  |
| EY755986 | S44282599 | 88 | 14 | 69.53367 | 17.71803 | -1.972493159 | 0 | protein |  |  |  |
| EY756027 | S44282640 | 11 | 0 | 8.691709 | 0 | -Inf | 0 | cytochrome p450 86a1 ame: full=cyplxxxvi ame: full=p450-dependent fatty acid omega-hydroxylase |  |  |  |
| EY756377 | S44282766 | 14 | 1 | 11.06217 | 1.265574 | -3.127771384 | 0 | conserved hypothetical protein [Ricinus communis] |  |  |  |
| EY756570 | S44282959 | 693 | 1898 | 547.5777 | 2402.059 | 2.133136273 | 0 | dna binding |  |  |  |
| EY756726 | S44283003 | 59 | 11 | 46.61917 | 13.92131 | -1.743627893 | 0 | calcium-dependent protein kinase sk5� |  |  |  |
| EY756842 | S44283119 | 30 | 2 | 23.70466 | 2.531148 | -3.227307058 | 0 | probable inactive receptor kinase at5g58300 flags: precursor |  |  |  |
| EY756862 | S44283139 | 44 | 3 | 34.76684 | 3.796722 | -3.19488558 | 0 | hypothetical protein [Vitis vinifera] |  |  |  |
| EY757359 | S44283412 | 100 | 18 | 79.01554 | 22.78033 | -1.79434765 | 0 | peptide transporter ptr1 |  |  |  |
| EY757563 | S44283616 | 49 | 6 | 38.71761 | 7.593443 | -2.350163805 | 0 | cs13-c1-001-024-h04- sweet orange field plant b citrus sinensis mrna |  |  |  |
| EY758066 | S44283895 | 230 | 108 | 181.7357 | 136.682 | -0.411019011 | 0 | cs13-c1-001-030-g06- sweet orange field plant b citrus sinensis mrna |  |  |  |
| EY758109 | S44283938 | 954 | 115 | 753.8082 | 145.541 | -2.372771867 | 0 | monocopper oxidase-like protein sks1 flags: precursor |  |  |  |
| EY758114 | S44283943 | 408 | 939 | 322.3834 | 1188.374 | 1.882139544 | 0 | zinc finger protein constans-like 2 |  |  |  |
| EY650207 | S44284101 | 125 | 293 | 98.76942 | 370.8132 | 1.908556108 | 0 | iaa-amino acid hydrolase ilr1-like 6 flags: precursor |  |  |  |
| EY650767 | S44284227 | 17 | 3 | 13.43264 | 3.796722 | -1.822916803 | 0 | cs00-c1-100-014-f01- sweet orange greenhouse plant citrus sinensis mrna |  |  |  |
| EY650832 | S44284292 | 31 | 6 | 24.49482 | 7.593443 | -1.689650272 | 0 | cs00-c1-100-015-c07- sweet orange greenhouse plant citrus sinensis mrna |  |  |  |
| EY650881 | S44284341 | 127 | 29 | 100.3497 | 36.70164 | -1.451120154 | 0 | gdsl esterase lipase at2g42990 ame: full=extracellular lipase at2g42990 flags: precursor |  |  |  |
| EY652026 | S44284688 | 326 | 49 | 257.5906 | 62.01312 | -2.054434772 | 0 | at3g19540 t31j18\_4 |  |  |  |
| EY652293 | S44284731 | 7089 | 1261 | 5601.411 | 1595.889 | -1.811430316 | 0 | ferric reductase-like transmembrane component |  |  |  |
| EY653557 | S44285113 | 714 | 66 | 564.1709 | 83.52788 | -2.755802607 | 0 | dna binding |  |  |  |
| EY653967 | S44285271 | 38 | 63 | 30.0259 | 79.73116 | 1.408935948 | 0 | regulator of nonsense |  |  |  |
| EY655127 | S44285423 | 68 | 7 | 53.73056 | 8.859017 | -2.600524381 | 0 | glucan endo- -beta-glucosidase 1 ame: full=(1- |  |  |  |
| EY655130 | S44285426 | 66 | 13 | 52.15025 | 16.45246 | -1.664370863 | 0 | fasciclin-like arabinogalactan protein 6 flags: precursor |  |  |  |
| EY655665 | S44285569 | 53 | 94 | 41.87823 | 118.9639 | 1.506251935 | 0 | conserved hypothetical protein [Ricinus communis] |  |  |  |
| EY657104 | S44286000 | 2922 | 521 | 2308.834 | 659.364 | -1.808017363 | 0 | glyceraldehyde-3-phosphate dehydrogenase chloroplastic ame: full=nadp-dependent glyceraldehydephosphate dehydrogenase subunit b flags: precursor |  |  |  |
| EY657499 | S44286087 | 14 | 1 | 11.06217 | 1.265574 | -3.127771384 | 0 | protein |  |  |  |
| EY657503 | S44286091 | 52 | 5 | 41.08808 | 6.32787 | -2.698928085 | 0 | chlorophyll a-b binding protein chloroplastic ame: full=lhcii type i cab-37� |  |  |  |
| EY658206 | S44286206 | 803 | 154 | 634.4947 | 194.8984 | -1.702886099 | 0 | caffeic acid 3-o-methyltransferase ame: full=s-adenosysl-l-methionine:caffeic acid 3-o-methyltransferase� |  |  |  |
| EY658297 | S44286297 | 30 | 0 | 23.70466 | 0 | -Inf | 0 | acyl synthetase |  |  |  |
| EY658417 | S44286319 | 29 | 1 | 22.91451 | 1.265574 | -4.178397457 | 0 | serine carboxypeptidase-like 45 flags: precursor |  |  |  |
| EY658938 | S44286546 | 188 | 35 | 148.5492 | 44.29509 | -1.745722297 | 0 | probable lrr receptor-like serine threonine-protein kinase at1g56130 flags: precursor |  |  |  |
| EY658940 | S44286548 | 71 | 10 | 56.10103 | 12.65574 | -2.148235487 | 0 | predicted protein [Populus trichocarpa] |  |  |  |
| EY659366 | S44286666 | 62 | 6 | 48.98963 | 7.593443 | -2.689650272 | 0 | conserved hypothetical protein [Ricinus communis] |  |  |  |
| EY660087 | S44286841 | 153 | 19 | 120.8938 | 24.0459 | -2.329876791 | 0 | lob domain-containing protein 21 ame: full=asymmetric leaves 2-like protein 12� |  |  |  |
| EY660812 | S44286908 | 29 | 3 | 22.91451 | 3.796722 | -2.593434956 | 0 | cs00-c1-101-026-c04- sweet orange infected with xylella fastidiosa (stage 1 of 2) citrus sinensis mrna |  |  |  |
| EY660859 | S44286955 | 10 | 0 | 7.901554 | 0 | -Inf | 0 | auxin-induced protein 6b |  |  |  |
| EY661193 | S44287191 | 19 | 3 | 15.01295 | 3.796722 | -1.983381475 | 0 | cellulose synthase a catalytic subunit 7� |  |  |  |
| EY661436 | S44287210 | 1715 | 637 | 1355.116 | 806.1706 | -0.749259761 | 0 | protein |  |  |  |
| EY661536 | S44287310 | 7 | 16 | 5.531087 | 20.24918 | 1.872228616 | 0 | probable lrr receptor-like serine threonine-protein kinase at4g08850 flags: precursor |  |  |  |
| EY662249 | S44287575 | 183 | 20 | 144.5984 | 25.31148 | -2.514188205 | 0 | cs00-c1-101-042-a12- sweet orange infected with xylella fastidiosa (stage 1 of 2) citrus sinensis mrna |  |  |  |
| EY662261 | S44287587 | 57 | 9 | 45.03885 | 11.39017 | -1.983381475 | 0 | probable pectinesterase pectinesterase inhibitor 54 includes: ame: full=pectinesterase inhibitor 54 ame: full=pectin methylesterase inhibitor 54 includes: ame: full=pectinesterase 54� |  |  |  |
| EY662635 | S44287751 | 26 | 1 | 20.54404 | 1.265574 | -4.02085618 | 0 | upf0497 membrane protein at4g25830 |  |  |  |
| EY662827 | S44287943 | 148 | 26 | 116.943 | 32.90492 | -1.82943011 | 0 | unknown [Zea mays] |  |  |  |
| EY664687 | S44288473 | 49609 | 10005 | 39198.82 | 12662.07 | -1.630297171 | 0 | cs00-c1-101-069-c09- sweet orange infected with xylella fastidiosa (stage 1 of 2) citrus sinensis mrna |  |  |  |
| EY664801 | S44288587 | 261 | 29 | 206.2305 | 36.70164 | -2.490341463 | 0 | protein |  |  |  |
| EY664805 | S44288591 | 88 | 234 | 69.53367 | 296.1443 | 2.090516639 | 0 | probable wrky transcription factor 70 ame: full=wrky dna-binding protein 70 |  |  |  |
| EY664808 | S44288594 | 17 | 34 | 13.43264 | 43.02951 | 1.679583538 | 0 | cs00-c1-101-070-f11- sweet orange infected with xylella fastidiosa (stage 1 of 2) citrus sinensis mrna |  |  |  |
| EY664847 | S44288633 | 3 | 12 | 2.370466 | 15.18689 | 2.679583538 | 0 | cs00-c1-101-071-b07- sweet orange infected with xylella fastidiosa (stage 1 of 2) citrus sinensis mrna |  |  |  |
| EY668167 | S44289615 | 11 | 1 | 8.691709 | 1.265574 | -2.779848081 | 0 | cs00-c1-102-041-b12- sweet orange infected with xylella fastidiosa (stage 2 of 2) citrus sinensis mrna |  |  |  |
| EY669455 | S44289657 | 1851 | 59 | 1462.578 | 74.66886 | -4.291862593 | 0 | ethylene-responsive transcription factor erf107 |  |  |  |
| EY669598 | S44289800 | 91 | 17 | 71.90414 | 21.51476 | -1.740748261 | 0 | bark storage protein a flags: precursor |  |  |  |
| EY670109 | S44289975 | 26 | 40 | 20.54404 | 50.62296 | 1.301071915 | 0 | probable lrr receptor-like serine threonine-protein kinase at1g53420 flags: precursor |  |  |  |
| EY672705 | S44290891 | 20 | 4 | 15.80311 | 5.062296 | -1.642344557 | 0 | cs00-c1-102-103-d01- sweet orange infected with xylella fastidiosa (stage 2 of 2) citrus sinensis mrna |  |  |  |
| EY672777 | S44290963 | 1049 | 1835 | 828.873 | 2322.328 | 1.486348923 | 0 | cs00-c1-102-108-b12- sweet orange infected with xylella fastidiosa (stage 2 of 2) citrus sinensis mrna |  |  |  |
| EY672827 | S44291013 | 109 | 22 | 86.12693 | 27.84263 | -1.629169168 | 0 | probable metal-nicotianamine transporter ysl5 ame: full=protein yellow stripe like 5� |  |  |  |
| EY672837 | S44291023 | 153 | 769 | 120.8938 | 973.2263 | 3.009035483 | 0 | tyrosine n-monooxygenase ame: full=cytochrome p450tyr ame: full=cytochrome p450 79a1 |  |  |  |
| EY672974 | S44291160 | 188 | 38 | 148.5492 | 48.09181 | -1.6270778 | 0 | protein |  |  |  |
| EY673783 | S44291535 | 105 | 7 | 82.96631 | 8.859017 | -3.227307058 | 0 | cytochrome p450 94a2 ame: full=p450-dependent fatty acid omega-hydroxylase |  |  |  |
| EY674028 | S44291654 | 5664 | 725 | 4475.44 | 917.5411 | -2.286184827 | 0 | phosphoenolpyruvate carboxykinase� |  |  |  |
| EY674080 | S44291706 | 10 | 0 | 7.901554 | 0 | -Inf | 0 | PREDICTED: hypothetical protein [Vitis vinifera] |  |  |  |
| EY674763 | S44291829 | 59 | 0 | 46.61917 | 0 | -Inf | 0 | gdsl esterase lipase at4g28780 ame: full=extracellular lipase at4g28780 flags: precursor |  |  |  |
| EY674818 | S44291870 | 20 | 1 | 15.80311 | 1.265574 | -3.642344557 | 0 | acyl synthetase |  |  |  |
| EY674821 | S44291873 | 57 | 2 | 45.03885 | 2.531148 | -4.153306476 | 0 | briggsae cbr-abt-1 protein |  |  |  |
| EY674830 | S44291882 | 58 | 8 | 45.82901 | 10.12459 | -2.178397457 | 0 | unnamed protein product [Vitis vinifera] |  |  |  |
| EY674874 | S44291926 | 75 | 125 | 59.26165 | 158.1967 | 1.416549132 | 0 | cytochrome p450 90d2 ame: full=c6-oxidase |  |  |  |
| EY676377 | S44291987 | 1681 | 319 | 1328.251 | 403.7181 | -1.718107858 | 0 | photosystem ii core complex proteins chloroplastic ame: full=l-arginine-metabolizing enzyme� |  |  |  |
| EY676418 | S44292028 | 916 | 1839 | 723.7823 | 2327.39 | 1.685085514 | 0 | conserved hypothetical protein [Ricinus communis] |  |  |  |
| EY676803 | S44292077 | 471 | 81 | 372.1632 | 102.5115 | -1.860149709 | 0 | glucan endo- -beta-glucosidase-like protein 3 flags: precursor |  |  |  |
| EY676841 | S44292115 | 31 | 3 | 24.49482 | 3.796722 | -2.689650272 | 0 | protein |  |  |  |
| EY676843 | S44292117 | 903 | 1756 | 713.5103 | 2222.348 | 1.63907849 | 0 | protein |  |  |  |
| EY676956 | S44292230 | 542 | 54 | 428.2642 | 68.34099 | -2.647678001 | 0 | geranylgeranyl pyrophosphate chloroplastic chromoplastic� |  |  |  |
| EY677004 | S44292278 | 213 | 858 | 168.3031 | 1085.862 | 2.689707755 | 0 | ethylene-responsive transcription factor 5� |  |  |  |
| EY677554 | S44292394 | 2607 | 534 | 2059.935 | 675.8165 | -1.607895398 | 0 | protein e6 |  |  |  |
| EY678233 | S44292415 | 61 | 12 | 48.19948 | 15.18689 | -1.666191299 | 0 | cs00-c1-401-045-h02- sweet orange infected with citrus sinensis mrna |  |  |  |
| EY678272 | S44292454 | 603 | 37 | 476.4637 | 46.82623 | -3.346977288 | 0 | fasciclin-like arabinogalactan protein 2 flags: precursor |  |  |  |
| EY680282 | S44292784 | 22 | 2 | 17.38342 | 2.531148 | -2.779848081 | 0 | protein |  |  |  |
| EY681381 | S44292987 | 12 | 2 | 9.481864 | 2.531148 | -1.905378963 | 0 | receptor protein kinase clavata1 flags: precursor |  |  |  |
| EY681774 | S44293268 | 1025 | 1977 | 809.9092 | 2502.04 | 1.627272499 | 0 | superoxide dismutase 1 |  |  |  |
| EY681779 | S44293273 | 11 | 2 | 8.691709 | 2.531148 | -1.779848081 | 0 | cs00-c1-650-025-g04- sweet orange young greenhouse plant citrus sinensis mrna |  |  |  |
| EY683932 | S44293998 | 263 | 53 | 207.8109 | 67.07542 | -1.631414997 | 0 | cytochrome p450 86a2 |  |  |  |
| EY684017 | S44294083 | 2016 | 254 | 1592.953 | 321.4558 | -2.309011699 | 0 | tsd2 (tumorous shoot development 2) methyltransferase |  |  |  |
| EY684150 | S44294216 | 849 | 89 | 670.8419 | 112.6361 | -2.574303775 | 0 | upf0497 membrane protein 11 |  |  |  |
| EY684201 | S44294267 | 411 | 76 | 324.7538 | 96.18362 | -1.755483532 | 0 | �domain-containing gpi-anchored protein 2 flags: precursor |  |  |  |
| EY686825 | S44294741 | 63 | 121 | 49.77979 | 153.1344 | 1.621166852 | 0 | phytochrome kinase substrate 1-like |  |  |  |
| EY686833 | S44294749 | 1075 | 731 | 849.417 | 925.1345 | 0.123190189 | 0 | cs00-c2-003-014-f03- sweet orange greenhouse plant citrus sinensis mrna |  |  |  |
| EY687405 | S44294985 | 1 | 0 | 0.790155 | 0 | -Inf | 0 | cs00-c2-003-044-d05- sweet orange greenhouse plant citrus sinensis mrna |  |  |  |
| EY687455 | S44295035 | 491 | 97 | 387.9663 | 122.7607 | -1.660082834 | 0 | cs00-c2-003-046-a09- sweet orange greenhouse plant citrus sinensis mrna |  |  |  |
| EY687709 | S44295149 | 3187 | 3542 | 2518.225 | 4482.663 | 0.831948731 | 0 | corylus avellana clone kg871 microsatellite sequence |  |  |  |
| EY688356 | S44295348 | 201 | 20 | 158.8212 | 25.31148 | -2.649540058 | 0 | 5ng4\_pintaauxin-induced protein 5ng4 |  |  |  |
| EY688477 | S44295469 | 69 | 7 | 54.52072 | 8.859017 | -2.621585997 | 0 | unknown [Glycine max] |  |  |  |
| EY688716 | S44295708 | 18 | 0 | 14.2228 | 0 | -Inf | 0 | cs00-c2-003-091-g03- sweet orange greenhouse plant citrus sinensis mrna |  |  |  |
| EY690065 | S44295811 | 58 | 0 | 45.82901 | 0 | -Inf | 0 | cs00-c2-003-083-h12- sweet orange greenhouse plant citrus sinensis mrna |  |  |  |
| EY690144 | S44295890 | 14 | 1 | 11.06217 | 1.265574 | -3.127771384 | 0 | ubiquitin carboxyl-terminal hydrolase 12 ame: full=ubiquitin thioesterase 12 ame: full=ubiquitin-specific-processing protease 12 ame: full=deubiquitinating enzyme 12� |  |  |  |
| EY690320 | S44295954 | 16 | 2 | 12.64249 | 2.531148 | -2.320416462 | 0 | cs00-c2-003-084-d03- sweet orange greenhouse plant citrus sinensis mrna |  |  |  |
| EY690363 | S44295997 | 461 | 76 | 364.2616 | 96.18362 | -1.921111889 | 0 | reticuline oxidase-like protein flags: precursor |  |  |  |
| EY691630 | S44296046 | 95 | 4 | 75.06476 | 5.062296 | -3.89027207 | 0 | ubiquitin carboxyl-terminal hydrolase 12 ame: full=ubiquitin thioesterase 12 ame: full=ubiquitin-specific-processing protease 12 ame: full=deubiquitinating enzyme 12� |  |  |  |
| EY691677 | S44296093 | 25 | 8 | 19.75388 | 10.12459 | -0.964272652 | 0 | populus est from leave |  |  |  |
| EY691688 | S44296104 | 381 | 701 | 301.0492 | 887.1673 | 1.559206984 | 0 | protein |  |  |  |
| EY691698 | S44296114 | 14 | 2 | 11.06217 | 2.531148 | -2.127771384 | 0 | probable lrr receptor-like serine threonine-protein kinase at4g08850 flags: precursor |  |  |  |
| EY691718 | S44296134 | 123 | 12 | 97.18911 | 15.18689 | -2.677968467 | 0 | endochitinase 1 flags: precursor |  |  |  |
| EY691729 | S44296145 | 170 | 369 | 134.3264 | 466.9968 | 1.797669608 | 0 | protein kinase g11a |  |  |  |
| EY691744 | S44296160 | 13 | 1 | 10.27202 | 1.265574 | -3.02085618 | 0 | ubiquitin-protein ligase, putative [Ricinus communis] |  |  |  |
| EY691847 | S44296263 | 45 | 4 | 35.55699 | 5.062296 | -2.812269558 | 0 | endoglucanase 11 ame: full=endo- -beta glucanase 11 flags: precursor |  |  |  |
| EY691883 | S44296299 | 31 | 0 | 24.49482 | 0 | -Inf | 0 | (+)-delta-cadinene synthase isozyme xc14� |  |  |  |
| EY692908 | S44296652 | 11 | 2 | 8.691709 | 2.531148 | -1.779848081 | 0 | protein |  |  |  |
| EY693354 | S44296986 | 1721 | 2535 | 1359.857 | 3208.23 | 1.238322188 | 0 | protein |  |  |  |
| EY694163 | S44297221 | 20 | 36 | 15.80311 | 45.56066 | 1.527580445 | 0 | serine threonine protein |  |  |  |
| EY694888 | S44297386 | 172 | 373 | 135.9067 | 472.0591 | 1.796350604 | 0 | 18 kda seed maturation protein |  |  |  |
| EY697211 | S44297931 | 13 | 2 | 10.27202 | 2.531148 | -2.02085618 | 0 | cs00-c3-700-057-e11- sweet orange development stadium (1 of 6) citrus sinensis mrna |  |  |  |
| EY698078 | S44298028 | 139 | 27 | 109.8316 | 34.1705 | -1.684470033 | 0 | tryptophan tyrosine permease |  |  |  |
| EY698173 | S44298123 | 4247 | 2325 | 3355.79 | 2942.459 | -0.189629855 | 0 | ethylene-responsive transcription factor erf003 |  |  |  |
| EY698191 | S44298141 | 110 | 19 | 86.91709 | 24.0459 | -1.853848662 | 0 | multidrug and toxin extrusion protein 2� |  |  |  |
| EY699267 | S44298335 | 16 | 30 | 12.64249 | 37.96722 | 1.586474134 | 0 | protein |  |  |  |
| EY700386 | S44298460 | 23 | 47 | 18.17357 | 59.48197 | 1.710610434 | 0 | atp synthase subunit chloroplastic ame: full=atpase subunit i ame: full=atp synthase f sector subunit b |  |  |  |
| EY702425 | S44298721 | 1273 | 2267 | 1005.868 | 2869.056 | 1.51213551 | 0 | protein |  |  |  |
| EY702934 | S44298894 | 56 | 6 | 44.2487 | 7.593443 | -2.542808883 | 0 | protein |  |  |  |
| EY704562 | S44299178 | 39 | 2 | 30.81606 | 2.531148 | -3.605818681 | 0 | protein yippee-like at4g27745 |  |  |  |
| EY705333 | S44299277 | 1212 | 233 | 957.6683 | 294.8787 | -1.699404301 | 0 | transcription factor spatula ame: full=transcription factor en 99 ame: full=bhlh transcription factor bhlh024 ame: full=basic helix-loop-helix protein 24� |  |  |  |
| EY709528 | S44300043 | 37 | 0 | 29.23575 | 0 | -Inf | 0 | isoflavone-7-o-methyltransferase 9 ame: full=isoflavone-o-methyltransferase 9 ame: full=7 iomt-9 |  |  |  |
| EY709546 | S44300061 | 21 | 1 | 16.59326 | 1.265574 | -3.712733885 | 0 | germin-like protein subfamily 1 member 8 flags: precursor |  |  |  |
| EY710217 | S44300172 | 2757 | 495 | 2178.458 | 626.4591 | -1.798015299 | 0 | transcription factor bhlh113 ame: full=transcription factor en 61 ame: full=bhlh transcription factor bhlh113 ame: full=basic helix-loop-helix protein 113� |  |  |  |
| EY710241 | S44300196 | 9 | 16 | 7.111398 | 20.24918 | 1.509658537 | 0 | (+)-delta-cadinene synthase isozyme xc14� |  |  |  |
| EY710274 | S44300229 | 4046 | 236 | 3196.969 | 298.6754 | -3.420054017 | 0 | expansin-a5� |  |  |  |
| EY710321 | S44300276 | 463 | 52 | 365.8419 | 65.80984 | -2.474845127 | 0 | cytochrome p450 82a4 ame: full=p450 cp9 |  |  |  |
| EY710324 | S44300279 | 1 | 10 | 0.790155 | 12.65574 | 4.001511633 | 0 | ubiquitin carboxyl-terminal hydrolase 13 ame: full=ubiquitin thioesterase 13 ame: full=ubiquitin-specific-processing protease 13 ame: full=deubiquitinating enzyme 13� |  |  |  |
| EY710358 | S44300313 | 3209 | 6558 | 2535.609 | 8299.634 | 1.71071565 | 0 | squidulin ame: full=optic lobe calcium-binding protein ame: full=scabp |  |  |  |
| EY710382 | S44300337 | 777 | 57 | 613.9507 | 72.13771 | -3.089297236 | 0 | miraculin-like protein 2 |  |  |  |
| EY710727 | S44300346 | 4718 | 682 | 3727.953 | 863.1214 | -2.110748236 | 0 | 14 kda proline-rich protein flags: precursor |  |  |  |
| EY710758 | S44300377 | 418 | 49 | 330.2849 | 62.01312 | -2.41306575 | 0 | 3-ketoacyl- synthase 21� |  |  |  |
| EY711870 | S44300817 | 33 | 2 | 26.07513 | 2.531148 | -3.364810581 | 0 | btb poz domain-containing protein at1g30440 |  |  |  |
| EY711896 | S44300843 | 60 | 6 | 47.40932 | 7.593443 | -2.642344557 | 0 | predicted protein [Populus trichocarpa] |  |  |  |
| EY714913 | S44302068 | 43 | 6 | 33.97668 | 7.593443 | -2.161718716 | 0 | PREDICTED: hypothetical protein [Vitis vinifera] |  |  |  |
| EY714918 | S44302073 | 133 | 24 | 105.0907 | 30.37377 | -1.790736397 | 0 | beta-galactosidase 10� |  |  |  |
| EY714927 | S44302082 | 156 | 262 | 123.2642 | 331.5804 | 1.427604321 | 0 | cs00-c3-702-048-d06- sweet orange development stadium (3 of 6) citrus sinensis mrna |  |  |  |
| EY715800 | S44302395 | 9 | 1 | 7.111398 | 1.265574 | -2.490341463 | 0 | cs00-c3-702-058-g10- sweet orange development stadium (3 of 6) citrus sinensis mrna |  |  |  |
| EY715871 | S44302466 | 1575 | 2742 | 1244.495 | 3470.204 | 1.47946028 | 0 | glycine-rich rna-binding protein mitochondrial� |  |  |  |
| EY716408 | S44302779 | 94 | 18 | 74.2746 | 22.78033 | -1.705080312 | 0 | cs00-c3-702-067-b04- sweet orange development stadium (3 of 6) citrus sinensis mrna |  |  |  |
| EY716845 | S44302880 | 254 | 35 | 200.6995 | 44.29509 | -2.179818132 | 0 | chalcone isomerase-like protein |  |  |  |
| EY717550 | S44303165 | 301 | 580 | 237.8368 | 734.0329 | 1.625872951 | 0 | cs00-c3-702-080-f08- sweet orange development stadium (3 of 6) citrus sinensis mrna |  |  |  |
| EY721877 | S44304228 | 174 | 125 | 137.487 | 158.1967 | 0.202424327 | 0 | atp synthase subunit chloroplastic ame: full=f-atpase subunit beta ame: full=atp synthase f1 sector subunit beta |  |  |  |
| EY724876 | S44305001 | 11 | 0 | 8.691709 | 0 | -Inf | 0 | protein |  |  |  |
| EY726474 | S44305390 | 17 | 13 | 13.43264 | 16.45246 | 0.292560415 | 0 | probable lrr receptor-like serine threonine-protein kinase at3g47570 flags: precursor |  |  |  |
| EY726580 | S44305496 | 869 | 1580 | 686.645 | 1999.607 | 1.542080014 | 0 | ethylene-responsive transcription factor rap2-3 ame: full=protein related to apetala2 3� |  |  |  |
| EY727166 | S44305760 | 8826 | 1621 | 6973.911 | 2051.495 | -1.765292119 | 0 | fructose-bisphosphate chloroplastic� |  |  |  |
| EY727625 | S44305883 | 322 | 47 | 254.43 | 59.48197 | -2.096744488 | 0 | pollen-specific protein sf3 |  |  |  |
| EY727716 | S44305974 | 1336 | 228 | 1055.648 | 288.5509 | -1.87123074 | 0 | glucosidase ii beta |  |  |  |
| EY727895 | S44306153 | 79 | 221 | 62.42227 | 279.6918 | 2.163705349 | 0 | plastidic atp adp-transporter |  |  |  |
| EY728204 | S44306238 | 1906 | 306 | 1506.036 | 387.2656 | -1.959361023 | 0 | cellulose synthase a catalytic subunit 2� |  |  |  |
| EY730799 | S44306845 | 21 | 38 | 16.59326 | 48.09181 | 1.535193629 | 0 | protein binding |  |  |  |
| EY730905 | S44306951 | 61 | 7 | 48.19948 | 8.859017 | -2.443798878 | 0 | tetratricopeptide repeat protein 7b� |  |  |  |
| EY731098 | S44307032 | 13 | 0 | 10.27202 | 0 | -Inf | 0 | 2-c-methyl-d-erythritol 4-phosphate cytidylyltransferase ame: full=4-diphosphocytidyl-2c-methyl-d-erythritol synthase ame: full=mep cytidylyltransferase� |  |  |  |
| EY731155 | S44307089 | 38 | 116 | 30.0259 | 146.8066 | 2.28963702 | 0 | u-box domain-containing protein 32 ame: full=plant u-box protein 32 |  |  |  |
| EY731164 | S44307098 | 251 | 48 | 198.329 | 60.74755 | -1.706997515 | 0 | �ring-h2 finger protein atl5e |  |  |  |
| EY732196 | S44307122 | 45 | 86 | 35.55699 | 108.8394 | 1.613995196 | 0 | conserved hypothetical protein [Ricinus communis] |  |  |  |
| EY734444 | S44307458 | 30181 | 5363 | 23847.68 | 6787.273 | -1.812944912 | 0 | heat shock cognate 70 kda protein 1� |  |  |  |
| EY735290 | S44307856 | 1175 | 183 | 928.4325 | 231.6 | -2.003161665 | 0 | abc transporter g family member 15� |  |  |  |
| EY735307 | S44307873 | 87 | 13 | 68.74352 | 16.45246 | -2.06292024 | 0 | cs00-c3-704-093-f03- sweet orange development stadium (5 of 6) citrus sinensis mrna |  |  |  |
| EY743074 | S44309878 | 37 | 7 | 29.23575 | 8.859017 | -1.722514906 | 0 | cs00-c3-705-099-h03- sweet orange development stadium (6 of 6) citrus sinensis mrna |  |  |  |
| EY743349 | S44310041 | 896 | 127 | 707.9792 | 160.7279 | -2.139086697 | 0 | ferric reductase-like transmembrane component |  |  |  |
| EY744797 | S44310593 | 153 | 29 | 120.8938 | 36.70164 | -1.71982331 | 0 | thaumatin-like protein flags: precursor |  |  |  |
| EY744814 | S44310610 | 130 | 238 | 102.7202 | 301.2066 | 1.552033488 | 0 | phosphatidylserine synthase 2� |  |  |  |
| EY745561 | S44310685 | 82 | 431 | 64.79274 | 545.4624 | 3.073575592 | 0 | heat stress transcription factor c-1� |  |  |  |
| EY746025 | S44310818 | 215 | 36 | 169.8834 | 45.56066 | -1.89868431 | 0 | 4-coumarate-- ligase 1� |  |  |  |
| EY746274 | S44310857 | 9 | 0 | 7.111398 | 0 | -Inf | 0 | cs00-c5-003-030-a04- sweet orange greenhouse plant citrus sinensis mrna |  |  |  |
| EY746288 | S44310871 | 49 | 24 | 38.71761 | 30.37377 | -0.350163805 | 0 | cs00-c5-003-030-b07- sweet orange greenhouse plant citrus sinensis mrna |  |  |  |
| EY746298 | S44310881 | 19 | 1 | 15.01295 | 1.265574 | -3.568343975 | 0 | maize gl1 homolog |  |  |  |
| EY746318 | S44310901 | 1222 | 155 | 965.5698 | 196.164 | -2.299320627 | 0 | protein |  |  |  |
| EY746373 | S44310956 | 0 | 13 | 0 | 16.45246 | Inf | 0 | protein |  |  |  |
| EY746433 | S44311016 | 99 | 164 | 78.22538 | 207.5541 | 1.407778922 | 0 | protein tify 5a ame: full=jasmonate zim domain-containing protein 8 |  |  |  |
| EY746569 | S44311152 | 6242 | 1142 | 4932.15 | 1445.285 | -1.770862169 | 0 | dehydration-responsive protein rd22 flags: precursor |  |  |  |
| EY746603 | S44311186 | 159 | 400 | 125.6347 | 506.2296 | 2.010556772 | 0 | acyl- chloroplastic ame: full=stearoyl-acp desaturase flags: precursor |  |  |  |
| EY746611 | S44311194 | 69 | 3 | 54.52072 | 3.796722 | -3.843978418 | 0 | 21 kda protein ame: full= protein flags: precursor |  |  |  |
| EY746650 | S44311233 | 1109 | 74 | 876.2823 | 93.65247 | -3.226006747 | 0 | pectinesterase pectinesterase inhibitor ppe8b includes: ame: full=pectinesterase inhibitor ppe8b ame: full=pectin methylesterase inhibitor ppe8b includes: ame: full=pectinesterase ppe8b� |  |  |  |
| EY746671 | S44311254 | 62 | 9 | 48.98963 | 11.39017 | -2.104687771 | 0 | beta-galactosidase 5� |  |  |  |
| EY747114 | S44311361 | 24 | 5 | 18.96373 | 6.32787 | -1.583450868 | 0 | serine carboxypeptidase-like 17 flags: precursor |  |  |  |
| EY747267 | S44311514 | 333 | 33 | 263.1217 | 41.76394 | -2.65540071 | 0 | snakin-2 flags: precursor |  |  |  |
| EY747838 | S44311651 | 136 | 381 | 107.4611 | 482.1837 | 2.165767884 | 0 | at4g37300 c7a10\_60 |  |  |  |
| EY747873 | S44311686 | 10 | 1 | 7.901554 | 1.265574 | -2.642344557 | 0 | cs00-c5-003-049-h03- sweet orange greenhouse plant citrus sinensis mrna |  |  |  |
| EY748285 | S44311762 | 50 | 7 | 39.50777 | 8.859017 | -2.15691773 | 0 | gdsl esterase lipase exl3 ame: full=family ii extracellular lipase 3� |  |  |  |
| EY748583 | S44312060 | 2504 | 327 | 1978.549 | 413.8427 | -2.257288483 | 0 | predicted protein [Populus trichocarpa] |  |  |  |
| EY749118 | S44312245 | 27 | 4 | 21.33419 | 5.062296 | -2.075303964 | 0 | probable xyloglucan endotransglucosylase hydrolase protein b ame: full= 2 flags: precursor |  |  |  |
| EY749122 | S44312249 | 16 | 3 | 12.64249 | 3.796722 | -1.735453961 | 0 | early nodulin-like protein 1 ame: full=phytocyanin-like protein flags: precursor |  |  |  |
| EY749642 | S44312433 | 15 | 2 | 11.85233 | 2.531148 | -2.227307058 | 0 | probable lrr receptor-like serine threonine-protein kinase at2g23950 flags: precursor |  |  |  |
| EY749818 | S44312609 | 187 | 31 | 147.7591 | 39.23279 | -1.913114612 | 0 | vitis vinifera beta-galactosidase mrna |  |  |  |
| EY750401 | S44312744 | 61 | 108 | 48.19948 | 136.682 | 1.503733703 | 0 | conserved hypothetical protein [Ricinus communis] |  |  |  |
| EY750476 | S44312819 | 19 | 52 | 15.01295 | 65.80984 | 2.132095743 | 0 | indole-3-acetic acid-amido synthetase ame: full=auxin-responsive gh3-like protein 3� |  |  |  |
| EY750529 | S44312872 | 29 | 4 | 22.91451 | 5.062296 | -2.178397457 | 0 | �pectate lyase 19 flags: precursor |  |  |  |
| EY750602 | S44312945 | 113 | 14 | 89.28755 | 17.71803 | -2.333240502 | 0 | pan troglodytes bac clone ch251-286m22 from chromosome complete sequence |  |  |  |
| EY751682 | S44313037 | 20 | 1 | 15.80311 | 1.265574 | -3.642344557 | 0 | gata transcription factor 10� |  |  |  |
| EY752069 | S44313102 | 48 | 217 | 37.92746 | 274.6295 | 2.85617227 | 0 | cs00-c5-003-092-e05- sweet orange greenhouse plant citrus sinensis mrna |  |  |  |
| EY752079 | S44313112 | 243 | 495 | 192.0078 | 626.4591 | 1.706055749 | 0 | transcription factor bhlh148 ame: full=transcription factor en 143 ame: full=bhlh transcription factor bhlh148 ame: full=basic helix-loop-helix protein 148� |  |  |  |
| EY752668 | S44313245 | 303 | 1025 | 239.4171 | 1297.213 | 2.437817749 | 0 | iaa-amino acid hydrolase ilr1-like 6 ame: full=protein gr1 flags: precursor |  |  |  |
| EY752669 | S44313246 | 15 | 2 | 11.85233 | 2.531148 | -2.227307058 | 0 | squamosa promoter-binding-like protein 13 |  |  |  |
| EY754662 | S44313783 | 228 | 306 | 180.1554 | 387.2656 | 1.104081366 | 0 | protein twin lov 1 |  |  |  |
| EY754720 | S44313841 | 20 | 41 | 15.80311 | 51.88853 | 1.715207448 | 0 | cs12-c1-001-019-c10- sweet orange field plant a citrus sinensis mrna |  |  |  |
| EY754792 | S44313913 | 36 | 4 | 28.44559 | 5.062296 | -2.490341463 | 0 | wall-associated receptor kinase 5 flags: precursor |  |  |  |
| EY755171 | S44314068 | 16 | 31 | 12.64249 | 39.23279 | 1.633779848 | 0 | probable phosphatase phospho2 |  |  |  |
| EY755825 | S44314498 | 143 | 21 | 112.9922 | 26.57705 | -2.087970376 | 0 | predicted protein [Populus trichocarpa] |  |  |  |
| EY756177 | S44314738 | 55 | 0 | 43.45854 | 0 | -Inf | 0 | probable lrr receptor-like serine threonine-protein kinase at1g12460 flags: precursor |  |  |  |
| EY756692 | S44314917 | 96 | 231 | 75.85491 | 292.3476 | 1.946370079 | 0 | histone ame: full=histone |  |  |  |
| EY756705 | S44314930 | 247 | 690 | 195.1684 | 873.246 | 2.161668858 | 0 | uncharacterized n-acetyltransferase p20 |  |  |  |
| EY756706 | S44314931 | 25 | 1 | 19.75388 | 1.265574 | -3.964272652 | 0 | multidrug resistance |  |  |  |
| EY757656 | S44315223 | 452 | 61 | 357.1502 | 77.20001 | -2.209858087 | 0 | aspartic proteinase nepenthesin-2 |  |  |  |
| EY757917 | S44315372 | 51 | 5 | 40.29792 | 6.32787 | -2.670913709 | 0 | glucan endo- -beta-glucosidase 1 ame: full=(1- |  |  |  |
| FE659288 | S46102877 | 260 | 495 | 205.4404 | 626.4591 | 1.60850044 | 0 | 645 hong anliu sweet orange ssh library citrus sinensis cdna 5 mrna |  |  |  |
| DC887374 | S47736050 | 6 | 9 | 4.740932 | 11.39017 | 1.264546039 | 0 | dc887374 eic citrus sinensis cdna clone eic0284 5 mrna |  |  |  |
| DC887730 | S47736207 | 33 | 4 | 26.07513 | 5.062296 | -2.364810581 | 0 | dc887730 eic citrus sinensis cdna clone eic0799 5 mrna |  |  |  |
| DC900219 | S47736517 | 57256 | 5774 | 45241.13 | 7307.424 | -2.630200334 | 0 | peroxidase 42� |  |  |  |
| FC871208 | S49954862 | 451 | 190 | 356.3601 | 240.459 | -0.567544477 | 0 | kn0aak3dd01fm1 ruit1 citrus sinensis cdna clone mrna |  |  |  |
| FC871426 | S49955080 | 459 | 1067 | 362.6813 | 1350.367 | 1.896577655 | 0 | dicyanin blue copper protein precursor |  |  |  |
| FC871687 | S49955341 | 474 | 552 | 374.5336 | 698.5968 | 0.899364746 | 0 | kn0aak1cb02fm2 ruit1 citrus sinensis cdna clone mrna |  |  |  |
| FC871875 | S49955529 | 84 | 13 | 66.37305 | 16.45246 | -2.012294167 | 0 | serine-threonine protein plant- |  |  |  |
| FC921914 | S49955742 | 16 | 33 | 12.64249 | 41.76394 | 1.723977657 | 0 | bahd acyltransferase at5g47980 |  |  |  |
| FC922338 | S49956166 | 112 | 235 | 88.4974 | 297.4099 | 1.748745562 | 0 | disease resistance response protein 206 |  |  |  |
| FC922537 | S49956365 | 0 | 3 | 0 | 3.796722 | Inf | 0 | lotus japonicus genomic chromosome clone: complete sequence |  |  |  |
| FC922597 | S49956425 | 353 | 66 | 278.9248 | 83.52788 | -1.739546716 | 0 | kn0aam2da04rm1 slh citrus sinensis cdna clone mrna |  |  |  |
| FC922608 | S49956436 | 28 | 220 | 22.12435 | 278.4263 | 3.653588329 | 0 | bap2 (bon association protein 2) |  |  |  |
| FC922613 | S49956441 | 100 | 12 | 79.01554 | 15.18689 | -2.379310151 | 0 | predicted protein [Populus trichocarpa] |  |  |  |
|  |  |  |  |  |  |  |  |  |  |  |  |
