# Supplementary material for: Transcriptome Profiling of Citrus Fruit Response to Huanglongbing Disease
Source: PLoS One. 2012 May 31;7(5):e38039. doi: 10.1371/journal.pone.0038039 (PMC3364978; doi:10.1371/journal.pone.0038039)
Supplement: Table S4 — Differentially expressed genes in apparently healthy fruits in comparison to asymptomatic, annotations and number of protein-protein interactions deduced from Arabidopsis knowledgebase. (HTM) [file pone.0038039.s004.htm]

Table�S4


# Table�S4

| Table S4. Differentially expressed genes in apparently healthy fruits in comparison to asymptomatic, annotations and number of protein-protein interactions deduced from Arabidopsis knowledgebase. | | | | | | | | |
|  |  |  |  |  |  |  |  |  |
| GB id | id2 | count AH | count AS | norm AH | norm AS | log2foldchange | PPI | annotation |
| CX302713 | S24635816 | 4 | 20 | 4.611556326 | 17.34772262 | 1.911420656 | 78 | 40s ribosomal protein s5-1 |
| CX303205 | S24636242 | 0 | 15 | 0 | 13.01079196 | Inf | 44 | heat shock 70 kda protein |
| CB305020 | S22559287 | 48 | 95 | 55.33867592 | 82.40168243 | 0.574385669 | 41 | er lumen protein retaining |
| FC921949 | S49955777 | 55 | 23 | 63.40889949 | 19.94988101 | -1.668305196 | 30 | s-adenosylmethionine-dependent methyltransferase |
| EY697005 | S44240315 | 1 | 2 | 1.152889082 | 1.734772262 | 0.589492561 | 27 | rac-like gtp-binding protein rac2 flags: precursor |
| EY750410 | S44312753 | 70 | 24 | 80.70223571 | 20.81726714 | -1.954827955 | 18 | actin-related protein 2 3 complex subunit 2 ame: full=arp2 3 complex 34 kda subunit� |
| DY257704 | S34124982 | 35 | 130 | 40.35111786 | 112.760197 | 1.482577357 | 16 | acid phosphatase 1 ame: full=apase-1 flags: precursor |
| EY696685 | S44239995 | 12 | 56 | 13.83466898 | 48.57362333 | 1.811884982 | 8 | hmg1 2-like protein |
| EY680829 | S44229087 | 28 | 124 | 32.28089428 | 107.5558802 | 1.736333949 | 5 | adipor-like receptor cg5315 |
| EY687316 | S44233580 | 2 | 10 | 2.305778163 | 8.673861309 | 1.911420656 | 5 | gamma-glutamyltranspeptidase 1 ame: full=gamma-glutamyltransferase 1 contains: ame: full=gamma-glutamyltranspeptidase 1 heavy chain contains: ame: full=gamma-glutamyltranspeptidase 1 light chain flags: precursor |
| EY698519 | S44241493 | 1 | 18 | 1.152889082 | 15.61295036 | 3.759417563 | 5 | cyclin-a3-2 ame: full=g2 mitotic-specific cyclin-a3-2� |
| EY692100 | S44236964 | 7 | 28 | 8.070223571 | 24.28681166 | 1.589492561 | 3 | cholesterol transport protein |
| EY703151 | S44245453 | 38 | 37 | 43.8097851 | 32.09328684 | -0.448981587 | 3 | della protein gai ame: full=gibberellic acid-insensitive mutant protein |
| CV714332 | S22576139 | 6 | 24 | 6.91733449 | 20.81726714 | 1.589492561 | 2 | dctp pyrophosphatase 1 ame: full=deoxycytidine-triphosphatase 1� |
| CX044293 | S22588309 | 37 | 12 | 42.65689602 | 10.40863357 | -2.034998304 | 1 | adenine phosphoribosyltransferase 2� |
| CX052350 | S22596008 | 3 | 15 | 3.458667245 | 13.01079196 | 1.911420656 | 1 | cysteine proteinase 15a ame: full=turgor-responsive protein 15a flags: precursor |
| EY655569 | S44211275 | 1 | 12 | 1.152889082 | 10.40863357 | 3.174455062 | 1 | 3-ketoacyl- synthase 19� |
| EY663018 | S44216260 | 16 | 7 | 18.44622531 | 6.071702916 | -1.603152517 | 1 | mterf family protein |
| EY678177 | S44226995 | 41 | 153 | 47.26845235 | 132.710078 | 1.489328399 | 1 | glutamine chloroplastic ame: full=gs2 ame: full=glutamate--ammonia ligase flags: precursor |
| EY705175 | S44247141 | 10 | 94 | 11.52889082 | 81.5342963 | 2.822153318 | 1 | tetrapyrrole-binding chloroplastic ame: full=genomes uncoupled 4 flags: precursor |
| EY650709 | S44284169 | 3 | 16 | 3.458667245 | 13.87817809 | 2.00453006 | 1 | gossypium hirsutum delta-12 fatty acid desaturase complete cds |
| BQ624512 | S22532331 | 14 | 2 | 16.14044714 | 1.734772262 | -3.217862361 | 0 | probable glutathione s-transferase ame: full=heat shock protein 26a ame: full=g2-4 |
| CF653219 | S22533093 | 0 | 21 | 0 | 18.21510875 | Inf | 0 | citrus sinensis complete genome |
| CK665498 | S22534213 | 4 | 21 | 4.611556326 | 18.21510875 | 1.981809984 | 0 | protein |
| CK739519 | S22535944 | 486 | 939 | 560.3040937 | 814.4755769 | 0.539661405 | 0 | usda-fp\_6357 ridge pineapple sweet orange entire seedling citrus sinensis cdna clone rse62e01 5 mrna |
| CK934734 | S22538912 | 6 | 32 | 6.91733449 | 27.75635619 | 2.00453006 | 0 | protein |
| CK934732 | S22538916 | 84 | 426 | 96.84268285 | 369.5064917 | 1.931884759 | 0 | cgf1004227\_e03 developing fruit peel at 38 dafb citrus sinensis cdna clone p38dab10002\_iif\_e03 5 mrna |
| CK935156 | S22539338 | 2 | 9 | 2.305778163 | 7.806475178 | 1.759417563 | 0 | cgf1004177\_c06 developing fruit peel at 38 dafb citrus sinensis cdna clone p38da0001\_iif\_c06 5 mrna |
| CK935639 | S22539754 | 2 | 7 | 2.305778163 | 6.071702916 | 1.396847483 | 0 | leishmania infantum chromosome 3 |
| CK935682 | S22539794 | 13 | 111 | 14.98755806 | 96.27986052 | 2.683468709 | 0 | cgf1004552\_h09 developing fruit 24 dafb citrus sinensis cdna clone t24dab0004\_if\_h09 5 mrna |
| CK935683 | S22539795 | 2 | 7 | 2.305778163 | 6.071702916 | 1.396847483 | 0 | cgf1004552\_h10 developing fruit 24 dafb citrus sinensis cdna clone t24dab0004\_if\_h10 5 mrna |
| CK936435 | S22540547 | 27 | 7 | 31.1280052 | 6.071702916 | -2.358040019 | 0 | cgf1004382\_d07 developing fruit 24 dafb citrus sinensis cdna clone t24dab0001\_ivf\_d07 5 mrna |
| CK936444 | S22540564 | 15 | 4 | 17.29333622 | 3.469544523 | -2.317398034 | 0 | cgf1004382\_c08 developing fruit 24 dafb citrus sinensis cdna clone t24dab0001\_ivf\_c08 5 mrna |
| CK936555 | S22541558 | 97 | 62 | 111.8302409 | 53.77794011 | -1.056223971 | 0 | cytochrome b6-f complex subunit 4 ame: full=17 kda polypeptide |
| CK937531 | S22541736 | 33 | 5 | 38.04533969 | 4.336930654 | -3.132973463 | 0 | cgf1004779\_d06 developing fruit albedo at 80 dafb in p x2 vector citrus sinensis cdna clone a80dab0003\_iiif\_d06 5 mrna |
| CK938208 | S22542505 | 2 | 0 | 2.305778163 | 0 | -Inf | 0 | cgf1004476\_g01 developing fruit albedo at 80 dafb in p x2 vector citrus sinensis cdna clone a80dab0002\_iif\_g01 5 mrna |
| CK938959 | S22542713 | 40 | 15 | 46.11556326 | 13.01079196 | -1.825544938 | 0 | 30s ribosomal protein chloroplastic |
| CK939658 | S22543787 | 4 | 23 | 4.611556326 | 19.94988101 | 2.113054517 | 0 | cgf1004747\_d06 developing fruit flavedo at 165 dafb citrus sinensis cdna clone f1650002\_iiif\_d06 5 mrna |
| CN185363 | S22544451 | 1869 | 2613 | 2154.749693 | 2266.47996 | 0.072933117 | 0 | vitis vinifera contig whole genome shotgun sequence |
| CN188208 | S22545836 | 28 | 65 | 32.28089428 | 56.38009851 | 0.804505452 | 0 | thaumatin-like protein 1 flags: precursor |
| CN187016 | S22546917 | 62548 | 16699 | 72110.90628 | 14484.481 | -2.315709482 | 0 | rhizanthes zippelii 26s ribosomal rna partial sequence |
| CN188086 | S22547394 | 551 | 2659 | 635.241884 | 2306.379722 | 1.860252114 | 0 | ucrcs05\_0005h16\_f washington navel orange stored fruit pulp cdna library citrus sinensis cdna clone mrna |
| CN189980 | S22548351 | 31752 | 49627 | 36606.53412 | 43045.77152 | 0.233770339 | 0 | ucrcs06\_0002l15\_f washington navel orange stored fruit rind cdna library citrus sinensis cdna clone mrna |
| CN189821 | S22550154 | 193 | 218 | 222.5075927 | 189.0901765 | -0.234780151 | 0 | ethanolamine-phosphate cytidylyltransferase ame: full=phosphorylethanolamine transferase ame: full=ctp:phosphoethanolamine cytidylyltransferase |
| CN182786 | S22551989 | 18 | 8 | 20.75200347 | 6.939089047 | -1.58043244 | 0 | flavonol synthase flavanone 3-hydroxylase |
| CN183270 | S22552255 | 14 | 6 | 16.14044714 | 5.204316785 | -1.63289986 | 0 | ucrcs04\_0003m05\_f ruby orange developing flower cdna library citrus sinensis cdna clone mrna |
| CN184985 | S22553198 | 40 | 16 | 46.11556326 | 13.87817809 | -1.732435534 | 0 | protein gast1 flags: precursor |
| CN185151 | S22553288 | 56 | 275 | 64.56178857 | 238.531186 | 1.885425447 | 0 | probable sulfate transporter |
| CB291413 | S22555698 | 14 | 51 | 16.14044714 | 44.23669267 | 1.454562981 | 0 | ucrcs01\_02de03\_g1 washington navel orange cold acclimated flavedo & albedo cdna library citrus sinensis cdna clone mrna |
| CB292168 | S22556123 | 0 | 9 | 0 | 7.806475178 | Inf | 0 | ucrcs01\_04ac10\_g1 washington navel orange cold acclimated flavedo & albedo cdna library citrus sinensis cdna clone mrna |
| CB292505 | S22556310 | 653 | 2627 | 752.8365703 | 2278.623366 | 1.597753865 | 0 | f-box family protein |
| CB290283 | S22557188 | 3 | 9 | 3.458667245 | 7.806475178 | 1.174455062 | 0 | af320906citrus unshiu metallothionein-like protein complete cds |
| CB291669 | S22557768 | 5 | 20 | 5.764445408 | 17.34772262 | 1.589492561 | 0 | ucrcs01\_03bb10\_g1 washington navel orange cold acclimated flavedo & albedo cdna library citrus sinensis cdna clone mrna |
| CB304476 | S22558815 | 5 | 2 | 5.764445408 | 1.734772262 | -1.732435534 | 0 | flavedo0001\_i \_d01 flavedo mature citrus sinensis cdna clone flavedo0001\_i \_d01 3 mrna |
| CB304943 | S22559228 | 1 | 16 | 1.152889082 | 13.87817809 | 3.589492561 | 0 | flavedo0003\_ii \_e05 flavedo mature citrus sinensis cdna clone flavedo0003\_ii \_e05 3 mrna |
| CB417320 | S22559715 | 6 | 28 | 6.91733449 | 24.28681166 | 1.811884982 | 0 | ucrca01\_01f01\_f bark of madame vinous sweet orange scion on standard sour orange citrus tristeza virus challenged - ucrca01 citrus aurantium cdna clone mrna |
| CB611167 | S22560410 | 1 | 15 | 1.152889082 | 13.01079196 | 3.496383157 | 0 | �kda class i heat shock protein ame: full= kda heat shock protein 1� |
| CF833196 | S22568730 | 10 | 41 | 11.52889082 | 35.56283136 | 1.625116471 | 0 | ucrcs02\_03d03\_r ruby orange ovary at anthesis cdna library citrus sinensis cdna clone mrna |
| CF836416 | S22571911 | 680 | 4387 | 783.9645755 | 3805.222956 | 2.279120616 | 0 | ucrcs03\_02o21\_f washington navel orange shoot meristem cdna library citrus sinensis cdna clone mrna |
| CF838727 | S22573043 | 4 | 23 | 4.611556326 | 19.94988101 | 2.113054517 | 0 | citrus sinensis dna binding protein (v03-2) complete cds |
| CF836044 | S22573488 | 12 | 45 | 13.83466898 | 39.03237589 | 1.496383157 | 0 | squamosa promoter-binding |
| CF836213 | S22573575 | 9545 | 12941 | 11004.32628 | 11224.84392 | 0.028624563 | 0 | ucrcs03\_02j17\_f washington navel orange shoot meristem cdna library citrus sinensis cdna clone mrna |
| CV713409 | S22575615 | 4 | 32 | 4.611556326 | 27.75635619 | 2.589492561 | 0 | AF506028\_16hypothetical protein [Poncirus trifoliata] |
| CV715004 | S22576523 | 2 | 10 | 2.305778163 | 8.673861309 | 1.911420656 | 0 | athila retroelement orf1 protein |
| CV715984 | S22577083 | 0 | 4 | 0 | 3.469544523 | Inf | 0 | abc transporter g family member 8� |
| CV716488 | S22577370 | 213 | 804 | 245.5653744 | 697.3784492 | 1.505834632 | 0 | citrus unshiu gene for glycine-rich complete cds |
| CV718227 | S22578359 | 59 | 20 | 68.02045581 | 17.34772262 | -1.971222393 | 0 | probable cytochrome c biosynthesis protein |
| CV718293 | S22578395 | 15 | 4 | 17.29333622 | 3.469544523 | -2.317398034 | 0 | fragaria vesca americana clone fosmid complete sequence |
| CV715924 | S22581082 | 4 | 22 | 4.611556326 | 19.08249488 | 2.04892418 | 0 | ucrcs08\_0006i12\_r parent washington navel orange callus cdna library ucrcs08-1 citrus sinensis cdna clone mrna |
| CV716021 | S22581123 | 8 | 40 | 9.223112653 | 34.69544523 | 1.911420656 | 0 | ucrcs08\_0006l01\_r parent washington navel orange callus cdna library ucrcs08-1 citrus sinensis cdna clone mrna |
| CV718401 | S22582137 | 2 | 13 | 2.305778163 | 11.2760197 | 2.289932279 | 0 | ucrcs08\_0010h03\_f parent washington navel orange callus cdna library ucrcs08-1 citrus sinensis cdna clone mrna |
| CV718524 | S22582188 | 19 | 10 | 21.90489255 | 8.673861309 | -1.336506857 | 0 | photosystem ii reaction center protein m� |
| CV718748 | S22582282 | 33 | 13 | 38.04533969 | 11.2760197 | -1.75446184 | 0 | ucrcs08\_0010p03\_f parent washington navel orange callus cdna library ucrcs08-1 citrus sinensis cdna clone mrna |
| CV719618 | S22582656 | 3 | 12 | 3.458667245 | 10.40863357 | 1.589492561 | 0 | ucrcs08\_0012d12\_r parent washington navel orange callus cdna library ucrcs08-1 citrus sinensis cdna clone mrna |
| CV885494 | S22583489 | 2 | 2 | 2.305778163 | 1.734772262 | -0.410507439 | 0 | protein |
| CV887374 | S22585048 | 2 | 12 | 2.305778163 | 10.40863357 | 2.174455062 | 0 | ucrcs04\_2\_030h07\_t3 ruby orange developing flower cdna library ucrcs04-ucr citrus sinensis cdna clone mrna |
| CV886709 | S22585659 | 1 | 14 | 1.152889082 | 12.14340583 | 3.396847483 | 0 | serine carboxypeptidase-like 18 flags: precursor |
| CX043988 | S22586454 | 4 | 19 | 4.611556326 | 16.48033649 | 1.837420075 | 0 | meiosis 5 |
| CX047991 | S22590171 | 48 | 11 | 55.33867592 | 9.541247439 | -2.536038321 | 0 | ribosomal protein mitochondrial |
| CX048538 | S22590428 | 10 | 2 | 11.52889082 | 1.734772262 | -2.732435534 | 0 | brassica rapa campestris clone complete sequence |
| CX048690 | S22590496 | 70 | 29 | 80.70223571 | 25.15419779 | -1.681809461 | 0 | 60s ribosomal protein mitochondrial |
| CX048758 | S22590529 | 47 | 39 | 54.18578683 | 33.8280591 | -0.679694072 | 0 | gdsl esterase lipase apg ame: full=extracellular lipase apg flags: precursor |
| CX051996 | S22592029 | 24 | 92 | 27.66933796 | 79.79952404 | 1.528092016 | 0 | PREDICTED: hypothetical protein [Vitis vinifera] |
| CX054039 | S22592993 | 0 | 76 | 0 | 65.92134594 | Inf | 0 | dna binding protein |
| CX046929 | S22593100 | 0 | 17 | 0 | 14.74556422 | Inf | 0 | 30s ribosomal protein chloroplastic |
| CX047037 | S22593159 | 1 | 3 | 1.152889082 | 2.602158393 | 1.174455062 | 0 | protein |
| CX047321 | S22593311 | 0 | 10 | 0 | 8.673861309 | Inf | 0 | phosphoribosylanthranilate transferase |
| CX047752 | S22593544 | 3 | 11 | 3.458667245 | 9.541247439 | 1.463961679 | 0 | ucrcs09\_16c11\_b ruby orange developing seed cdna library ucrcs09 citrus sinensis cdna clone ucrcs09-16c11-e21-1- mrna |
| CX048727 | S22594065 | 23 | 8 | 26.51644888 | 6.939089047 | -1.934069395 | 0 | ribosomal protein mitochondrial |
| CX048969 | S22594194 | 63 | 27 | 72.63201214 | 23.41942553 | -1.63289986 | 0 | arabidopsis thaliana mitochondrial genome |
| CX049428 | S22594442 | 27 | 560 | 31.1280052 | 485.7362333 | 3.963888076 | 0 | ucrcs09\_27e07\_b ruby orange developing seed cdna library ucrcs09 citrus sinensis cdna clone ucrcs09-27e07-j14-1- mrna |
| CX051193 | S22595387 | 16 | 69 | 18.44622531 | 59.84964303 | 1.698017018 | 0 | glycogenin-1 |
| CX051341 | S22595470 | 10 | 1 | 11.52889082 | 0.867386131 | -3.732435534 | 0 | fragaria vesca americana clone fosmid complete sequence |
| CX071402 | S22597854 | 10 | 158 | 11.52889082 | 137.0470087 | 3.571345214 | 0 | populus trichocarpa mrna |
| CX071698 | S22597989 | 88 | 453 | 101.4542392 | 392.9259173 | 1.953428183 | 0 | ucrcs08\_23d06\_g parent washington navel orange callus cdna library ucrcs08-2 citrus sinensis cdna clone ucrcs08-23d06-h11-1- mrna |
| CX073495 | S22598823 | 2 | 11 | 2.305778163 | 9.541247439 | 2.04892418 | 0 | ucrcs08\_33d09\_b parent washington navel orange callus cdna library ucrcs08-2 citrus sinensis cdna clone ucrcs08-33d09-g17-1- mrna |
| CX074820 | S22599436 | 83 | 456 | 95.68979377 | 395.5280757 | 2.047343144 | 0 | citrus ichangensis satellite dna |
| CX077288 | S22600576 | 42 | 179 | 48.42134143 | 155.2621174 | 1.680990916 | 0 | bap2 (bon association protein 2) |
| CX069655 | S22601382 | 0 | 10 | 0 | 8.673861309 | Inf | 0 | peroxidase 72� |
| CX071630 | S22602442 | 25 | 27 | 28.82222704 | 23.41942553 | -0.299476126 | 0 | arabidopsis thaliana mitochondrial genome |
| CX075413 | S22604474 | 1 | 10 | 1.152889082 | 8.673861309 | 2.911420656 | 0 | ucrcs08\_44e07\_g parent washington navel orange callus cdna library ucrcs08-2 citrus sinensis cdna clone ucrcs08-44e07-j14-1- mrna |
| CX075506 | S22604525 | 4 | 17 | 4.611556326 | 14.74556422 | 1.676955402 | 0 | pentatricopeptide repeat-containing protein at5g56310 |
| CX077871 | S22605795 | 2 | 18 | 2.305778163 | 15.61295036 | 2.759417563 | 0 | hydroxyproline-rich glycoprotein family protein |
| AF000135 | S22606213 | 13 | 4 | 14.98755806 | 3.469544523 | -2.110947157 | 0 | endoglucanase 1 ame: full=endo- -beta-glucanase 1 ame: full=abscission cellulase 1 flags: precursor |
| CX673911 | S23019511 | 0 | 10 | 0 | 8.673861309 | Inf | 0 | conserved hypothetical protein [Ricinus communis] |
| CX674613 | S23019889 | 1 | 16 | 1.152889082 | 13.87817809 | 3.589492561 | 0 | protein |
| CX671853 | S23020402 | 7 | 29 | 8.070223571 | 25.15419779 | 1.640118634 | 0 | ucrcs10\_13e07\_g madame vinous sweet orange multiple pathogen-infected cdna library ucrcs10 citrus sinensis cdna clone ucrcs10-13e07-i13- mrna |
| CX672332 | S23020647 | 0 | 12 | 0 | 10.40863357 | Inf | 0 | poncirus trifoliata citrus tristeza virus resistance gene complete sequence |
| CX674961 | S23021431 | 3 | 28 | 3.458667245 | 24.28681166 | 2.811884982 | 0 | protein |
| CX673883 | S23021551 | 0 | 11 | 0 | 9.541247439 | Inf | 0 | thromboxane-a synthase� |
| CX674272 | S23021730 | 37 | 256 | 42.65689602 | 222.0508495 | 2.380039195 | 0 | ucrcs10\_5d08\_b madame vinous sweet orange multiple pathogen-infected cdna library ucrcs10 citrus sinensis cdna clone ucrcs10-5d08-g15- mrna |
| CX300836 | S24634758 | 2 | 19 | 2.305778163 | 16.48033649 | 2.837420075 | 0 | c08002g06sk ootsw1 citrus sinensis cdna clone mrna |
| CX301073 | S24634830 | 16 | 6 | 18.44622531 | 5.204316785 | -1.825544938 | 0 | n-acetyltransferase 13 ame: full=n-acetyltransferase 5� |
| CX302396 | S24635390 | 0 | 10 | 0 | 8.673861309 | Inf | 0 | c08020c09sk ootsw1 citrus sinensis cdna clone mrna |
| CX302610 | S24635769 | 4 | 16 | 4.611556326 | 13.87817809 | 1.589492561 | 0 | basic 7s globulin ame: full=sbg7s� |
| DR403432 | S25678430 | 3 | 14 | 3.458667245 | 12.14340583 | 1.811884982 | 0 | csac-pnp1241a05 mature albedo citrus sinensis cdna clone csac-pnp1241a05 5 mrna |
| DR403913 | S25678911 | 104 | 398 | 119.9004645 | 345.2196801 | 1.525677464 | 0 | potassium transporter 6� |
| DR403972 | S25678970 | 2 | 10 | 2.305778163 | 8.673861309 | 1.911420656 | 0 | csah-pnp1246n23 developing fruit peel at 38 dafb citrus sinensis cdna clone csah-pnp1246n23 5 mrna |
| DR405282 | S25680280 | 15 | 49 | 17.29333622 | 42.50192041 | 1.29731181 | 0 | csab-pnp1240p15 developing fruit albedo at 165 dafb citrus sinensis cdna clone csab-pnp1240p15 5 mrna |
| DR405291 | S25680289 | 2 | 25 | 2.305778163 | 21.68465327 | 3.233348751 | 0 | csad-pnp1242a02 developing fruit flavedo at 165 dafb citrus sinensis cdna clone csad-pnp1242a02 5 mrna |
| DR405609 | S25680607 | 10 | 40 | 11.52889082 | 34.69544523 | 1.589492561 | 0 | csad-pnp1242p19 developing fruit flavedo at 165 dafb citrus sinensis cdna clone csad-pnp1242p19 5 mrna |
| DR406125 | S25681123 | 0 | 7 | 0 | 6.071702916 | Inf | 0 | protein |
| DR909097 | S26279974 | 246 | 460 | 283.6107141 | 398.9976202 | 0.492468107 | 0 | hth dna-binding expressed |
| DR909652 | S26280529 | 10 | 42 | 11.52889082 | 36.4302175 | 1.659881889 | 0 | usda-fp\_17780 citrus sinensis phloem citrus sinensis cdna clone vpe-14\_c04 5 mrna |
| DR909987 | S26280864 | 12 | 57 | 13.83466898 | 49.44100946 | 1.837420075 | 0 | proline-rich glycoprotein |
| DR910089 | S26280966 | 4 | 27 | 4.611556326 | 23.41942553 | 2.344380063 | 0 | usda-fp\_18217 citrus sinensis phloem citrus sinensis cdna clone vpe-21\_f01 5 mrna |
| DR910642 | S26281519 | 3 | 15 | 3.458667245 | 13.01079196 | 1.911420656 | 0 | vitis whole genome shotgun contig clone entav 115 |
| DR911129 | S26282006 | 6 | 1 | 6.91733449 | 0.867386131 | -2.99546994 | 0 | populus trichocarpa mrna |
| DR911173 | S26282050 | 6 | 51 | 6.91733449 | 44.23669267 | 2.676955402 | 0 | usda-fp\_19301 citrus sinensis phloem citrus sinensis cdna clone vpe-31\_a06 5 mrna |
| DR911992 | S26282869 | 158 | 74 | 182.1564749 | 64.18657368 | -1.504834821 | 0 | carica papaya complete genome |
| DT214578 | S26468967 | 4 | 15 | 4.611556326 | 13.01079196 | 1.496383157 | 0 | ucrcs11\_01a07\_f2 parent washington navel orange red scale-infested rind cdna library ucrcs11-2 citrus sinensis cdna clone mrna |
| DY257153 | S34124431 | 602 | 2229 | 694.0392271 | 1933.403686 | 1.478053786 | 0 | homo sapiens chromosome 21 segment hs21c085 |
| DY257186 | S34124464 | 10 | 41 | 11.52889082 | 35.56283136 | 1.625116471 | 0 | kn0aak1bf05fm2 ruit citrus sinensis cdna 5 mrna |
| DY257274 | S34124552 | 23 | 11 | 26.51644888 | 9.541247439 | -1.474637776 | 0 | length cdna complete sequence from clone gsltsil38zb08 of silique of strain col-0 of arabidopsis thaliana (thale cress) |
| DY257298 | S34124576 | 13 | 49 | 14.98755806 | 42.50192041 | 1.503762687 | 0 | arabidopsis thaliana at1g53210 f12m16\_12 complete cds |
| DY257460 | S34124738 | 2 | 16 | 2.305778163 | 13.87817809 | 2.589492561 | 0 | polyphenol oxidase with tyrosine hydroxylase activity protein |
| DY257559 | S34124837 | 5 | 24 | 5.764445408 | 20.81726714 | 1.852526967 | 0 | protein |
| DY305501 | S34125063 | 31 | 13 | 35.73956153 | 11.2760197 | -1.664264031 | 0 | photosystem i assembly protein ycf4 |
| DY305698 | S34125260 | 284 | 132 | 327.4204992 | 114.4949693 | -1.515860439 | 0 | caffeic acid 3-o-methyltransferase 1 ame: full=s-adenosysl-l-methionine:caffeic acid 3-o-methyltransferase 1� |
| DY305720 | S34125282 | 11 | 4 | 12.6817799 | 3.469544523 | -1.869939058 | 0 | citrus sinensis complete genome |
| AB276108 | S35152777 | 18 | 71 | 20.75200347 | 61.58441529 | 1.569314679 | 0 | beta- soluble isoenzyme i ame: full=sucrose hydrolase ame: full=invertase ame: full=saccharase flags: precursor |
| EY649805 | S44206967 | 4 | 32 | 4.611556326 | 27.75635619 | 2.589492561 | 0 | ethylene-responsive transcription factor shine 2 |
| EY650015 | S44207177 | 14 | 59 | 16.14044714 | 51.17578172 | 1.664780688 | 0 | anthocyanidin 3-o-glucosyltransferase 2 ame: full=flavonol 3-o-glucosyltransferase 2 ame: full=udp-glucose flavonoid 3-o-glucosyltransferase 2 |
| EY650389 | S44207439 | 33 | 9 | 38.04533969 | 7.806475178 | -2.284976557 | 0 | phytochromobilin:ferredoxin chloroplastic ame: full=phytochromobilin synthase ame: full=pfb synthase ame: full=p synthase flags: precursor |
| EY650412 | S44207462 | 0 | 10 | 0 | 8.673861309 | Inf | 0 | cs00-c1-100-010-g02- sweet orange greenhouse plant citrus sinensis mrna |
| EY651736 | S44208450 | 1 | 14 | 1.152889082 | 12.14340583 | 3.396847483 | 0 | cs00-c1-100-025-f01- sweet orange greenhouse plant citrus sinensis mrna |
| EY652156 | S44208660 | 2 | 13 | 2.305778163 | 11.2760197 | 2.289932279 | 0 | populus trichocarpa clone pop1- complete sequence |
| EY652581 | S44208973 | 8 | 37 | 9.223112653 | 32.09328684 | 1.798945927 | 0 | conserved hypothetical protein [Ricinus communis] |
| EY652692 | S44209084 | 5 | 21 | 5.764445408 | 18.21510875 | 1.659881889 | 0 | cs00-c1-100-036-b02- sweet orange greenhouse plant citrus sinensis mrna |
| EY653001 | S44209393 | 14 | 77 | 16.14044714 | 66.78873208 | 2.04892418 | 0 | �-like protein chloroplastic ame: full= -related thylakoid lumenal protein 2 ame: full=oec23-like protein 4 flags: precursor |
| EY656063 | S44211685 | 12 | 2 | 13.83466898 | 1.734772262 | -2.99546994 | 0 | uncharacterized basic helix-loop-helix protein at1g06150 |
| EY657167 | S44212369 | 0 | 10 | 0 | 8.673861309 | Inf | 0 | cs00-c1-100-074-h03- sweet orange greenhouse plant citrus sinensis mrna |
| EY657824 | S44212858 | 2 | 4 | 2.305778163 | 3.469544523 | 0.589492561 | 0 | polygalacturonase at1g48100� |
| EY658617 | S44213413 | 0 | 7 | 0 | 6.071702916 | Inf | 0 | protein transparent testa 12 |
| EY660216 | S44214578 | 13 | 4 | 14.98755806 | 3.469544523 | -2.110947157 | 0 | alpha-glucosidase yihq |
| EY662963 | S44216205 | 22 | 8 | 25.36355979 | 6.939089047 | -1.869939058 | 0 | cs00-c1-101-050-a02- sweet orange infected with xylella fastidiosa (stage 1 of 2) citrus sinensis mrna |
| EY663599 | S44216505 | 0 | 6 | 0 | 5.204316785 | Inf | 0 | cs00-c1-101-056-h08- sweet orange infected with xylella fastidiosa (stage 1 of 2) citrus sinensis mrna |
| EY664267 | S44217061 | 8 | 1 | 9.223112653 | 0.867386131 | -3.410507439 | 0 | cs00-c1-101-064-g02- sweet orange infected with xylella fastidiosa (stage 1 of 2) citrus sinensis mrna |
| EY665690 | S44218260 | 10 | 4 | 11.52889082 | 3.469544523 | -1.732435534 | 0 | cs00-c1-102-092-e05- sweet orange infected with xylella fastidiosa (stage 2 of 2) citrus sinensis mrna |
| EY669120 | S44220696 | 0 | 13 | 0 | 11.2760197 | Inf | 0 | cs00-c1-102-072-c01- sweet orange infected with xylella fastidiosa (stage 2 of 2) citrus sinensis mrna |
| EY669903 | S44221157 | 25 | 9 | 28.82222704 | 7.806475178 | -1.884438627 | 0 | cs00-c1-102-052-h08- sweet orange infected with xylella fastidiosa (stage 2 of 2) citrus sinensis mrna |
| EY671014 | S44221932 | 3 | 16 | 3.458667245 | 13.87817809 | 2.00453006 | 0 | protein binding |
| EY672374 | S44222844 | 3 | 20 | 3.458667245 | 17.34772262 | 2.326458155 | 0 | cs00-c1-102-109-d06- sweet orange infected with xylella fastidiosa (stage 2 of 2) citrus sinensis mrna |
| EY673920 | S44223494 | 13 | 56 | 14.98755806 | 48.57362333 | 1.696407765 | 0 | cs00-c1-102-004-g03- sweet orange infected with xylella fastidiosa (stage 2 of 2) citrus sinensis mrna |
| EY674424 | S44223900 | 2 | 15 | 2.305778163 | 13.01079196 | 2.496383157 | 0 | protein binding protein |
| EY675948 | S44225214 | 10 | 52 | 11.52889082 | 45.1040788 | 1.968004184 | 0 | protein |
| EY676513 | S44225667 | 6 | 127 | 6.91733449 | 110.1580386 | 3.993214747 | 0 | af283537\_1lectin-related protein precursor |
| EY676749 | S44225903 | 1 | 10 | 1.152889082 | 8.673861309 | 2.911420656 | 0 | cs00-c1-401-026-d09- sweet orange infected with citrus sinensis mrna |
| EY677097 | S44226027 | 9 | 30 | 10.37600173 | 26.02158393 | 1.326458155 | 0 | protein |
| EY677189 | S44226119 | 13 | 47 | 14.98755806 | 40.76714815 | 1.443641695 | 0 | protein |
| EY677929 | S44226747 | 3 | 32 | 3.458667245 | 27.75635619 | 3.00453006 | 0 | lectin-4 ame: full=lectin iv ame: full=gs4 |
| EY678518 | S44227224 | 20 | 7 | 23.05778163 | 6.071702916 | -1.925080612 | 0 | formin-like protein 1� |
| EY679230 | S44227824 | 4 | 25 | 4.611556326 | 21.68465327 | 2.233348751 | 0 | cs00-c1-401-056-h04- sweet orange infected with citrus sinensis mrna |
| EY679388 | S44227982 | 0 | 15 | 0 | 13.01079196 | Inf | 0 | aspartic proteinase nepenthesin-1 ame: full=nepenthesin-i flags: precursor |
| EY679457 | S44228051 | 4 | 16 | 4.611556326 | 13.87817809 | 1.589492561 | 0 | cs00-c1-401-057-d08- sweet orange infected with citrus sinensis mrna |
| EY679601 | S44228195 | 1 | 8 | 1.152889082 | 6.939089047 | 2.589492561 | 0 | cs00-c1-650-002-a04- sweet orange young greenhouse plant citrus sinensis mrna |
| EY681323 | S44229581 | 2 | 14 | 2.305778163 | 12.14340583 | 2.396847483 | 0 | cs00-c1-650-020-g04- sweet orange young greenhouse plant citrus sinensis mrna |
| EY685494 | S44232408 | 73 | 129 | 84.16090296 | 111.8928109 | 0.410895258 | 0 | predicted protein [Populus trichocarpa] |
| EY685507 | S44232421 | 15 | 6 | 17.29333622 | 5.204316785 | -1.732435534 | 0 | protein |
| EY685743 | S44232573 | 5 | 33 | 5.764445408 | 28.62374232 | 2.311958586 | 0 | peptide transporter ptr1 |
| EY686574 | S44233286 | 1 | 14 | 1.152889082 | 12.14340583 | 3.396847483 | 0 | cs00-c2-003-055-b07- sweet orange greenhouse plant citrus sinensis mrna |
| EY687323 | S44233587 | 17 | 65 | 19.59911439 | 56.38009851 | 1.524397533 | 0 | cs00-c2-003-040-c07- sweet orange greenhouse plant citrus sinensis mrna |
| EY687909 | S44233977 | 5 | 24 | 5.764445408 | 20.81726714 | 1.852526967 | 0 | cs00-c2-003-050-d04- sweet orange greenhouse plant citrus sinensis mrna |
| EY688092 | S44234048 | 11 | 49 | 12.6817799 | 42.50192041 | 1.744770787 | 0 | cysteine-rich receptor-like protein kinase 24� |
| EY688172 | S44234128 | 3 | 13 | 3.458667245 | 11.2760197 | 1.704969779 | 0 | cs00-c2-003-087-b05- sweet orange greenhouse plant citrus sinensis mrna |
| EY691434 | S44236620 | 75 | 25 | 86.46668112 | 21.68465327 | -1.99546994 | 0 | citrus sinensis dna binding protein (v03-3) complete cds |
| EY691980 | S44236844 | 4 | 26 | 4.611556326 | 22.5520394 | 2.289932279 | 0 | ankyrin-1 ame: full=erythrocyte ankyrin ame: full=ankyrin-r |
| EY692203 | S44237067 | 3 | 15 | 3.458667245 | 13.01079196 | 1.911420656 | 0 | cs00-c2-003-098-b06- sweet orange greenhouse plant citrus sinensis mrna |
| EY697656 | S44240854 | 4 | 15 | 4.611556326 | 13.01079196 | 1.496383157 | 0 | uncharacterized protein at4g38062 |
| EY697731 | S44240929 | 8 | 30 | 9.223112653 | 26.02158393 | 1.496383157 | 0 | cs00-c3-700-063-g01- sweet orange development stadium (1 of 6) citrus sinensis mrna |
| EY697948 | S44241146 | 2 | 9 | 2.305778163 | 7.806475178 | 1.759417563 | 0 | cs00-c3-700-066-b12- sweet orange development stadium (1 of 6) citrus sinensis mrna |
| EY699454 | S44242316 | 22 | 94 | 25.36355979 | 81.5342963 | 1.684649794 | 0 | hypothetical protein [Vitis vinifera] |
| EY701801 | S44244327 | 12 | 54 | 13.83466898 | 46.83885107 | 1.759417563 | 0 | dna binding protein |
| EY701880 | S44244406 | 36 | 199 | 41.50400694 | 172.60984 | 2.05619218 | 0 | endochitinase flags: precursor |
| EY702213 | S44244739 | 7 | 30 | 8.070223571 | 26.02158393 | 1.689028235 | 0 | cs00-c3-701-004-c12- sweet orange development stadium (2 of 6) citrus sinensis mrna |
| EY702512 | S44244926 | 11 | 5 | 12.6817799 | 4.336930654 | -1.548010963 | 0 | atp synthase subunit chloroplastic ame: full=f-atpase subunit iv ame: full=atp synthase f0 sector subunit a |
| EY703522 | S44245600 | 25 | 11 | 28.82222704 | 9.541247439 | -1.59493201 | 0 | zinc finger ccch domain-containing protein 18� |
| EY704314 | S44246392 | 11 | 5 | 12.6817799 | 4.336930654 | -1.548010963 | 0 | trehalose-phosphate phosphatase |
| EY704766 | S44246732 | 2 | 13 | 2.305778163 | 11.2760197 | 2.289932279 | 0 | cs00-c3-701-036-c10- sweet orange development stadium (2 of 6) citrus sinensis mrna |
| EY704790 | S44246756 | 132 | 559 | 152.1813588 | 484.8688471 | 1.671802915 | 0 | ammonium transporter 1 member 2 ame: full= 1 2 |
| EY704963 | S44246929 | 10 | 43 | 11.52889082 | 37.29760363 | 1.693829221 | 0 | growth-regulating factor 1 |
| EY705949 | S44247691 | 10 | 42 | 11.52889082 | 36.4302175 | 1.659881889 | 0 | 29 kda ribonucleoprotein chloroplastic ame: full=cp29a flags: precursor |
| EY706226 | S44247968 | 0 | 10 | 0 | 8.673861309 | Inf | 0 | 2 -cyclic-nucleotide 2 -phosphodiesterase |
| EY707122 | S44248864 | 0 | 8 | 0 | 6.939089047 | Inf | 0 | dehydration-responsive family protein |
| EY707742 | S44249484 | 0 | 3 | 0 | 2.602158393 | Inf | 0 | cs00-c3-701-072-b01- sweet orange development stadium (2 of 6) citrus sinensis mrna |
| EY709631 | S44250728 | 5 | 19 | 5.764445408 | 16.48033649 | 1.51549198 | 0 | cs00-c3-701-048-d11- sweet orange development stadium (2 of 6) citrus sinensis mrna |
| EY709864 | S44250961 | 5 | 19 | 5.764445408 | 16.48033649 | 1.51549198 | 0 | anthranilate n-benzoyltransferase protein 1 ame: full=anthranilate n-hydroxycinnamoyl benzoyltransferase 1 |
| EY710505 | S44251378 | 6 | 30 | 6.91733449 | 26.02158393 | 1.911420656 | 0 | �upf0481 protein at3g02645 |
| EY710689 | S44251562 | 5 | 22 | 5.764445408 | 19.08249488 | 1.726996085 | 0 | l-ascorbate oxidase homolog ame: full=pollen-specific protein ntp303 flags: precursor |
| EY711107 | S44251868 | 31 | 10 | 35.73956153 | 8.673861309 | -2.042775654 | 0 | cs00-c3-702-003-h02- sweet orange development stadium (3 of 6) citrus sinensis mrna |
| EY712805 | S44252908 | 5 | 19 | 5.764445408 | 16.48033649 | 1.51549198 | 0 | cs00-c3-702-023-f07- sweet orange development stadium (3 of 6) citrus sinensis mrna |
| EY717756 | S44255633 | 26 | 118 | 29.97511612 | 102.3515634 | 1.771695892 | 0 | ndf6 (ndh dependent flow 6) |
| EY719494 | S44256959 | 2 | 2 | 2.305778163 | 1.734772262 | -0.410507439 | 0 | cs00-c3-702-087-a03- sweet orange development stadium (3 of 6) citrus sinensis mrna |
| EY719738 | S44257203 | 2 | 13 | 2.305778163 | 11.2760197 | 2.289932279 | 0 | cs00-c3-702-106-b10- sweet orange development stadium (3 of 6) citrus sinensis mrna |
| EY719785 | S44257250 | 1072 | 1891 | 1235.897095 | 1640.227173 | 0.408337019 | 0 | cs00-c3-702-106-g10- sweet orange development stadium (3 of 6) citrus sinensis mrna |
| EY723237 | S44259800 | 2 | 21 | 2.305778163 | 18.21510875 | 2.981809984 | 0 | linear gramicidin synthetase subunit d includes: ame: full=atp-dependent tryptophan adenylase� |
| EY727958 | S44262972 | 12 | 51 | 13.83466898 | 44.23669267 | 1.676955402 | 0 | probable esterase at1g33990 |
| EY727967 | S44262981 | 9 | 32 | 10.37600173 | 27.75635619 | 1.41956756 | 0 | probable pectinesterase pectinesterase inhibitor 54 includes: ame: full=pectinesterase inhibitor 54 ame: full=pectin methylesterase inhibitor 54 includes: ame: full=pectinesterase 54� |
| EY728404 | S44263306 | 2 | 19 | 2.305778163 | 16.48033649 | 2.837420075 | 0 | gdsl esterase lipase at5g42170 ame: full=extracellular lipase at5g42170 flags: precursor |
| EY730076 | S44264642 | 8 | 36 | 9.223112653 | 31.22590071 | 1.759417563 | 0 | phosphate import atp-binding protein pstb ame: full=phosphate-transporting atpase ame: full=abc phosphate transporter |
| EY730142 | S44264708 | 17 | 108 | 19.59911439 | 93.67770213 | 2.256917222 | 0 | aphanomyces euteiches cdna |
| EY730330 | S44264896 | 0 | 3 | 0 | 2.602158393 | Inf | 0 | auxin response factor 18 ame: full= 10 |
| EY732042 | S44266160 | 14 | 59 | 16.14044714 | 51.17578172 | 1.664780688 | 0 | sjchgc09076 protein |
| EY732591 | S44266597 | 13 | 51 | 14.98755806 | 44.23669267 | 1.561478185 | 0 | cs00-c3-704-064-b12- sweet orange development stadium (5 of 6) citrus sinensis mrna |
| EY733371 | S44267377 | 6 | 27 | 6.91733449 | 23.41942553 | 1.759417563 | 0 | cs00-c3-704-054-g10- sweet orange development stadium (5 of 6) citrus sinensis mrna |
| EY734757 | S44268449 | 3 | 23 | 3.458667245 | 19.94988101 | 2.528092016 | 0 | cs00-c3-704-087-b05- sweet orange development stadium (5 of 6) citrus sinensis mrna |
| EY734777 | S44268469 | 1 | 12 | 1.152889082 | 10.40863357 | 3.174455062 | 0 | cs00-c3-704-087-e05- sweet orange development stadium (5 of 6) citrus sinensis mrna |
| EY735535 | S44268779 | 0 | 11 | 0 | 9.541247439 | Inf | 0 | cs00-c3-704-090-g12- sweet orange development stadium (5 of 6) citrus sinensis mrna |
| EY736067 | S44269199 | 2 | 10 | 2.305778163 | 8.673861309 | 1.911420656 | 0 | cs00-c3-705-009-e12- sweet orange development stadium (6 of 6) citrus sinensis mrna |
| EY747045 | S44276960 | 58 | 338 | 66.86756673 | 293.1765122 | 2.132391002 | 0 | miraculin� |
| EY747509 | S44277200 | 1 | 58 | 1.152889082 | 50.30839559 | 5.447473556 | 0 | defensin-like protein 6 ame: full=plant defensin ame: full=low-molecular-weight cysteine-rich protein 74� |
| EY748174 | S44277641 | 117 | 53 | 134.8880225 | 45.97146494 | -1.552951704 | 0 | uncharacterized glycosyltransferase at1g55740 |
| EY748232 | S44277699 | 7 | 28 | 8.070223571 | 24.28681166 | 1.589492561 | 0 | chalcone synthase 5 ame: full=naringenin-chalcone synthase 5 |
| EY749464 | S44278273 | 1 | 11 | 1.152889082 | 9.541247439 | 3.04892418 | 0 | cs00-c5-003-070-b11- sweet orange greenhouse plant citrus sinensis mrna |
| EY749556 | S44278365 | 4 | 18 | 4.611556326 | 15.61295036 | 1.759417563 | 0 | eh domain-containing protein 3 |
| EY749940 | S44278525 | 0 | 19 | 0 | 16.48033649 | Inf | 0 | tubulin alpha-2 chain ame: full=alpha-2-tubulin |
| EY750814 | S44279063 | 17 | 1 | 19.59911439 | 0.867386131 | -4.49797028 | 0 | lrr receptor-like serine threonine-protein kinase rch1 ame: full=protein root clavata-homolog1 1 flags: precursor |
| EY750938 | S44279187 | 50 | 19 | 57.64445408 | 16.48033649 | -1.806436115 | 0 | omega-3 fatty acid endoplasmic reticulum ame: full=indole-3-acetic acid-induced protein arg1 |
| EY751117 | S44279360 | 0 | 11 | 0 | 9.541247439 | Inf | 0 | cs00-c5-003-103-h09- sweet orange greenhouse plant citrus sinensis mrna |
| EY753520 | S44281337 | 13 | 47 | 14.98755806 | 40.76714815 | 1.443641695 | 0 | serine threonine-protein kinase bri1-like 2 ame: full=brassinosteroid insensitive 1-like protein 2 ame: full=protein vascular highway 1 flags: precursor |
| EY756541 | S44282930 | 198 | 309 | 228.2720382 | 268.0223144 | 0.231598969 | 0 | populus trichocarpa mrna |
| EY757316 | S44283369 | 4 | 18 | 4.611556326 | 15.61295036 | 1.759417563 | 0 | probable lrr receptor-like serine threonine-protein kinase at2g24230 flags: precursor |
| EY757389 | S44283442 | 1 | 11 | 1.152889082 | 9.541247439 | 3.04892418 | 0 | cs13-c1-001-022-g08- sweet orange field plant b citrus sinensis mrna |
| EY757563 | S44283616 | 6 | 45 | 6.91733449 | 39.03237589 | 2.496383157 | 0 | cs13-c1-001-024-h04- sweet orange field plant b citrus sinensis mrna |
| EY757951 | S44283780 | 20 | 105 | 23.05778163 | 91.07554374 | 1.981809984 | 0 | cs13-c1-001-029-e03- sweet orange field plant b citrus sinensis mrna |
| EY758066 | S44283895 | 108 | 164 | 124.5120208 | 142.2513255 | 0.192157064 | 0 | cs13-c1-001-030-g06- sweet orange field plant b citrus sinensis mrna |
| EY655144 | S44285440 | 21 | 2 | 24.21067071 | 1.734772262 | -3.802824862 | 0 | protein ycf2 |
| EY657503 | S44286091 | 5 | 28 | 5.764445408 | 24.28681166 | 2.074919388 | 0 | chlorophyll a-b binding protein chloroplastic ame: full=lhcii type i cab-37� |
| EY660781 | S44286877 | 70 | 49 | 80.70223571 | 42.50192041 | -0.925080612 | 0 | cs00-c1-101-025-h08- sweet orange infected with xylella fastidiosa (stage 1 of 2) citrus sinensis mrna |
| EY660812 | S44286908 | 3 | 52 | 3.458667245 | 45.1040788 | 3.704969779 | 0 | cs00-c1-101-026-c04- sweet orange infected with xylella fastidiosa (stage 1 of 2) citrus sinensis mrna |
| EY661135 | S44287133 | 12 | 0 | 13.83466898 | 0 | -Inf | 0 | fasciclin-like arabinogalactan protein 11 flags: precursor |
| EY661508 | S44287282 | 39 | 171 | 44.96267418 | 148.3230284 | 1.721942857 | 0 | protein |
| EY663158 | S44288050 | 8 | 30 | 9.223112653 | 26.02158393 | 1.496383157 | 0 | protein |
| EY664186 | S44288420 | 79 | 48 | 91.07823745 | 41.63453428 | -1.129325686 | 0 | conserved hypothetical protein [Ricinus communis] |
| EY672705 | S44290891 | 4 | 20 | 4.611556326 | 17.34772262 | 1.911420656 | 0 | cs00-c1-102-103-d01- sweet orange infected with xylella fastidiosa (stage 2 of 2) citrus sinensis mrna |
| EY673627 | S44291379 | 11 | 25 | 12.6817799 | 21.68465327 | 0.773917132 | 0 | cs00-c1-102-012-c06- sweet orange infected with xylella fastidiosa (stage 2 of 2) citrus sinensis mrna |
| EY674035 | S44291661 | 7 | 121 | 8.070223571 | 104.9537218 | 3.701000876 | 0 | transcriptional xre family |
| EY674830 | S44291882 | 8 | 29 | 9.223112653 | 25.15419779 | 1.447473556 | 0 | unnamed protein product [Vitis vinifera] |
| EY678260 | S44292442 | 4 | 34 | 4.611556326 | 29.49112845 | 2.676955402 | 0 | af283537\_1lectin-related protein precursor |
| EY681751 | S44293245 | 4 | 35 | 4.611556326 | 30.35851458 | 2.718775578 | 0 | non-specific lipid-transfer protein 3� |
| EY688594 | S44295586 | 18 | 6 | 20.75200347 | 5.204316785 | -1.99546994 | 0 | cs00-c2-003-090-a09- sweet orange greenhouse plant citrus sinensis mrna |
| EY688716 | S44295708 | 0 | 8 | 0 | 6.939089047 | Inf | 0 | cs00-c2-003-091-g03- sweet orange greenhouse plant citrus sinensis mrna |
| EY691677 | S44296093 | 8 | 18 | 9.223112653 | 15.61295036 | 0.759417563 | 0 | populus est from leave |
| EY691744 | S44296160 | 1 | 18 | 1.152889082 | 15.61295036 | 3.759417563 | 0 | ubiquitin-protein ligase, putative [Ricinus communis] |
| EY694163 | S44297221 | 36 | 16 | 41.50400694 | 13.87817809 | -1.58043244 | 0 | serine threonine protein |
| EY694888 | S44297386 | 373 | 1944 | 430.0276274 | 1686.198638 | 1.971273244 | 0 | 18 kda seed maturation protein |
| EY697211 | S44297931 | 2 | 15 | 2.305778163 | 13.01079196 | 2.496383157 | 0 | cs00-c3-700-057-e11- sweet orange development stadium (1 of 6) citrus sinensis mrna |
| EY703404 | S44299028 | 5 | 16 | 5.764445408 | 13.87817809 | 1.267564466 | 0 | protein |
| EY709528 | S44300043 | 0 | 12 | 0 | 10.40863357 | Inf | 0 | isoflavone-7-o-methyltransferase 9 ame: full=isoflavone-o-methyltransferase 9 ame: full=7 iomt-9 |
| EY713165 | S44301216 | 3 | 11 | 3.458667245 | 9.541247439 | 1.463961679 | 0 | cs00-c3-702-027-g11- sweet orange development stadium (3 of 6) citrus sinensis mrna |
| EY714913 | S44302068 | 6 | 27 | 6.91733449 | 23.41942553 | 1.759417563 | 0 | PREDICTED: hypothetical protein [Vitis vinifera] |
| EY714918 | S44302073 | 24 | 11 | 27.66933796 | 9.541247439 | -1.536038321 | 0 | beta-galactosidase 10� |
| EY717978 | S44303355 | 7 | 25 | 8.070223571 | 21.68465327 | 1.425993829 | 0 | cs00-c3-702-086-h11- sweet orange development stadium (3 of 6) citrus sinensis mrna |
| EY721877 | S44304228 | 125 | 149 | 144.1111352 | 129.2405335 | -0.157123203 | 0 | atp synthase subunit chloroplastic ame: full=f-atpase subunit beta ame: full=atp synthase f1 sector subunit beta |
| EY729741 | S44306445 | 12 | 3 | 13.83466898 | 2.602158393 | -2.410507439 | 0 | cs00-c3-704-020-g10- sweet orange development stadium (5 of 6) citrus sinensis mrna |
| EY744773 | S44310569 | 12 | 5 | 13.83466898 | 4.336930654 | -1.673541845 | 0 | predicted protein [Populus trichocarpa] |
| EY744797 | S44310593 | 29 | 126 | 33.43378337 | 109.2906525 | 1.708791489 | 0 | thaumatin-like protein flags: precursor |
| EY746288 | S44310871 | 24 | 38 | 27.66933796 | 32.96067297 | 0.252457574 | 0 | cs00-c5-003-030-b07- sweet orange greenhouse plant citrus sinensis mrna |
| EY746298 | S44310881 | 1 | 27 | 1.152889082 | 23.41942553 | 4.344380063 | 0 | maize gl1 homolog |
| EY746611 | S44311194 | 3 | 15 | 3.458667245 | 13.01079196 | 1.911420656 | 0 | 21 kda protein ame: full= protein flags: precursor |
| EY747114 | S44311361 | 5 | 43 | 5.764445408 | 37.29760363 | 2.693829221 | 0 | serine carboxypeptidase-like 17 flags: precursor |
| EY747180 | S44311427 | 3 | 12 | 3.458667245 | 10.40863357 | 1.589492561 | 0 | cs00-c5-003-042-b05- sweet orange greenhouse plant citrus sinensis mrna |
| EY748412 | S44311889 | 26 | 12 | 29.97511612 | 10.40863357 | -1.525984656 | 0 | cyclic nucleotide-gated ion channel 1� |
| EY750585 | S44312928 | 10 | 9 | 11.52889082 | 7.806475178 | -0.562510532 | 0 | phenylalanine ammonia-lyase |
| EY752069 | S44313102 | 217 | 1309 | 250.1769307 | 1135.408445 | 2.182190711 | 0 | cs00-c5-003-092-e05- sweet orange greenhouse plant citrus sinensis mrna |
| EY753091 | S44313332 | 1074 | 5930 | 1238.202874 | 5143.599756 | 2.054530673 | 0 | cs00-c5-003-050-h09- sweet orange greenhouse plant citrus sinensis mrna |
| EY754766 | S44313887 | 174 | 468 | 200.6027002 | 405.9367092 | 1.016913785 | 0 | cs12-c1-001-019-g10- sweet orange field plant a citrus sinensis mrna |
| EY754767 | S44313888 | 9 | 48 | 10.37600173 | 41.63453428 | 2.00453006 | 0 | cs12-c1-001-019-g12- sweet orange field plant a citrus sinensis mrna |
| EY755171 | S44314068 | 31 | 8 | 35.73956153 | 6.939089047 | -2.364703749 | 0 | probable phosphatase phospho2 |
| EY756177 | S44314738 | 0 | 13 | 0 | 11.2760197 | Inf | 0 | probable lrr receptor-like serine threonine-protein kinase at1g12460 flags: precursor |
| EY757129 | S44315018 | 3 | 13 | 3.458667245 | 11.2760197 | 1.704969779 | 0 | cs13-c1-001-017-g03- sweet orange field plant b citrus sinensis mrna |
| EY757863 | S44315318 | 5 | 30 | 5.764445408 | 26.02158393 | 2.174455062 | 0 | serine carboxypeptidase-like 42 flags: precursor |
| DC887730 | S47736207 | 4 | 16 | 4.611556326 | 13.87817809 | 1.589492561 | 0 | dc887730 eic citrus sinensis cdna clone eic0799 5 mrna |
|  |  |  |  |  |  |  |  |  |
