# Supplementary material for: Transcriptome Profiling of Citrus Fruit Response to Huanglongbing Disease
Source: PLoS One. 2012 May 31;7(5):e38039. doi: 10.1371/journal.pone.0038039 (PMC3364978; doi:10.1371/journal.pone.0038039)
Supplement: Table S5 — Differentially expressed genes in symptomatic fruits in comparison to asymptomatic, annotations and number of protein-protein interactions deduced from Arabidopsis knowledgebase. (HTM) [file pone.0038039.s005.htm]

Table�S5


# Table�S5

| Table S5. Differentially expressed genes in symptomatic fruits in comparison to asymptomatic, annotations and number of protein-protein interactions deduced from Arabidopsis knowledgebase. | | | | | | | | |
|  |  |  |  |  |  |  |  |  |
| GB id | id2 | count AS | count SY | norm AS | norm SY | log2foldchange | PPI | annotation |
| EY683846 | S44231096 | 160 | 409 | 150.4830181 | 434.8663443 | 1.530971369 | 54 | serine acetyltransferase mitochondrial� |
| EY747286 | S44276977 | 110 | 34 | 103.457075 | 36.15025845 | -1.516954441 | 53 | probable mannitol dehydrogenase ame: full=nad-dependent mannitol dehydrogenase |
| DR908750 | S26279627 | 68 | 18 | 63.9552827 | 19.13837212 | -1.740595409 | 48 | nuclear transport factor 2� |
| EY662694 | S44287810 | 1183 | 316 | 1112.633815 | 335.984755 | -1.727511179 | 31 | �kda proline-rich protein |
| FC921949 | S49955777 | 23 | 88 | 21.63193386 | 93.56537481 | 2.112812093 | 30 | s-adenosylmethionine-dependent methyltransferase |
| CK933773 | S22537734 | 12 | 3 | 11.28622636 | 3.189728687 | -1.823057569 | 29 | chromatin assembly factor subunit |
| CK934395 | S22538579 | 5 | 17 | 4.702594316 | 18.07512922 | 1.942477177 | 27 | dna-directed rna polymerase subunit beta ame: full=pep ame: full=plastid-encoded rna polymerase subunit beta� |
| EY650454 | S44207504 | 16 | 45 | 15.04830181 | 47.8459303 | 1.668795527 | 25 | ccr4-not transcription complex subunit 3 ame: full=ccr4-associated factor 3 ame: full=leukocyte receptor cluster member 2 |
| CF653559 | S22533293 | 36 | 114 | 33.85867908 | 121.2096901 | 1.839907444 | 24 | pathogenesis-related protein 1� |
| DC900352 | S47736649 | 204 | 52 | 191.8658481 | 55.28863057 | -1.795043193 | 20 | chloroplast-targeted copper |
| EY713241 | S44301292 | 435 | 1211 | 409.1257055 | 1287.587146 | 1.65405399 | 19 | abc transporter c family member 3� |
| EY712020 | S44252347 | 23 | 5 | 21.63193386 | 5.316214478 | -2.02469143 | 14 | 8-oxoguanine dna |
| EY740246 | S44272490 | 686 | 1723 | 645.1959402 | 1831.967509 | 1.505584651 | 14 | peptide transporter ptr2 ame: full=histidine-transporting protein |
| EY756199 | S44314760 | 11 | 3 | 10.3457075 | 3.189728687 | -1.697526687 | 14 | arabidopsis thaliana pola2 (dna polymerase alpha 2) dna binding dna-directed dna polymerase complete cds |
| EY684115 | S44294181 | 1063 | 3151 | 999.7715517 | 3350.278364 | 1.744610588 | 12 | peroxisomal -2-hydroxy-acid oxidase ame: full=glycolate oxidase� |
| CV886236 | S22583855 | 81 | 215 | 76.18202793 | 228.5972225 | 1.585285278 | 10 | developmental protein sepallata 1 ame: full=agamous-like mads-box protein agl2 |
| CX675973 | S23017894 | 23 | 3 | 21.63193386 | 3.189728687 | -2.761657025 | 10 | probable histone ame: full=hta7 |
| DY257364 | S34124642 | 212 | 659 | 199.389999 | 700.6770681 | 1.813156631 | 10 | ammonium transporter 2� |
| EY673229 | S44223139 | 310 | 1149 | 291.5608476 | 1221.666087 | 2.066981108 | 10 | �homolog subfamily b member 13 ame: full=testis spermatocyte apoptosis-related gene 6 protein ame: full=testis and spermatogenesis cell-related protein 6 ame: full=testis spermatogenesis apoptosis-related gene 6 protein ame: full=testis spermatogenesis apoptosis-related gene 3 protein |
| EY684548 | S44231462 | 202 | 80 | 189.9848104 | 85.05943164 | -1.159340957 | 8 | �endonuclease flj39025 |
| DY305557 | S34125119 | 49 | 14 | 46.0854243 | 14.88540054 | -1.630412491 | 7 | serine threonine-protein kinase nek4 ame: full= -related protein kinase 4� |
| DY305942 | S34125504 | 979 | 2711 | 920.7679672 | 2882.45149 | 1.646386779 | 7 | glucan endo- -beta-glucosidase 14 ame: full=(1- |
| EY683854 | S44231104 | 120 | 578 | 112.8622636 | 614.5543936 | 2.444977518 | 6 | fructose-bisphosphate cytoplasmic isozyme 1 |
| EY702355 | S44244881 | 13 | 3 | 12.22674522 | 3.189728687 | -1.938534787 | 6 | ubiquitin carrier protein e2 20 |
| EY677252 | S44226182 | 305 | 94 | 286.8582533 | 99.94483218 | -1.52113415 | 5 | �phosphate dikinase regulatory protein chloroplastic ame: full= pi dikinase regulatory protein 1� |
| DR404045 | S25679043 | 7 | 21 | 6.583632043 | 22.32810081 | 1.761904932 | 4 | para-hydroxybenzoate-- mitochondrial� |
| EY701434 | S44243960 | 10 | 2 | 9.405188633 | 2.126485791 | -2.144985664 | 4 | anaphase-promoting complex subunit cdc20 |
| EY728100 | S44263114 | 100 | 29 | 94.05188633 | 30.83404397 | -1.608932764 | 4 | 125 kda kinesin-related protein |
| CV716509 | S22581329 | 976 | 3211 | 917.9464106 | 3414.072938 | 1.895012043 | 3 | photosystem q protein ame: full=32 kda thylakoid membrane protein ame: full=photosystem ii protein d1 flags: precursor |
| CX051384 | S22591744 | 258 | 667 | 242.6538667 | 709.1830113 | 1.547258127 | 3 | atp synthase subunit chloroplastic ame: full=f-atpase subunit alpha ame: full=atp synthase f1 sector subunit alpha |
| CX053639 | S22596692 | 6 | 24 | 5.64311318 | 25.51782949 | 2.176942431 | 3 | random slug protein 5 ame: full=cral-trio domain-containing protein 5 |
| DR908226 | S26279103 | 702 | 74 | 660.244242 | 78.67997427 | -3.068931424 | 3 | thioredoxin h-type 2� |
| DR910057 | S26280934 | 23 | 6 | 21.63193386 | 6.379457373 | -1.761657025 | 3 | phosphoinositide phospholipase c 6 ame: full=phosphoinositide phospholipase plc6� |
| EY692100 | S44236964 | 28 | 5 | 26.33452817 | 5.316214478 | -2.308484396 | 3 | cholesterol transport protein |
| EY706290 | S44248032 | 358 | 2256 | 336.7057531 | 2398.675972 | 2.832678006 | 3 | probable lrr receptor-like serine threonine-protein kinase at1g56130 flags: precursor |
| EY711989 | S44252316 | 19 | 2 | 17.8698584 | 2.126485791 | -3.070985083 | 3 | lrr receptor-like serine threonine-protein kinase gso1 ame: full=protein gassho 1 flags: precursor |
| EY744046 | S44274722 | 2551 | 6968 | 2399.26362 | 7408.676496 | 1.626624156 | 3 | glyceraldehyde-3-phosphate dehydrogenase chloroplastic ame: full=nadp-dependent glyceraldehydephosphate dehydrogenase subunit a flags: precursor |
| EY692767 | S44296511 | 2 | 11 | 1.881037727 | 11.69567185 | 2.636374049 | 3 | beta-hexosaminidase subunit b2 ame: full=n-acetyl-beta-glucosaminidase subunit b2 ame: full=beta-n-acetylhexosaminidase subunit b2 flags: precursor |
| CV714332 | S22576139 | 24 | 115 | 22.57245272 | 122.272933 | 2.437469981 | 2 | dctp pyrophosphatase 1 ame: full=deoxycytidine-triphosphatase 1� |
| CV715043 | S22580707 | 217 | 428 | 204.0925933 | 455.0679593 | 1.156858185 | 2 | �atp synthase protein ymf19 ame: full=mitochondrial protein ymf19 |
| CV886565 | S22584007 | 478 | 132 | 449.5680167 | 140.3480622 | -1.679530258 | 2 | acyl-protein thioesterase 2� |
| CX071523 | S22602384 | 22 | 4 | 20.69141499 | 4.252971582 | -2.282489188 | 2 | probable auxin efflux carrier component 1c ame: full= 1c |
| CX671540 | S23020242 | 26 | 75 | 24.45349045 | 79.74321716 | 1.705321403 | 2 | primary amine oxidase ame: full=amine oxidase flags: precursor |
| DN620167 | S24240472 | 139 | 489 | 130.732122 | 519.9257759 | 1.991692013 | 2 | arogenate dehydrogenase chloroplastic ame: full= 2 flags: precursor |
| DY305576 | S34125138 | 1439 | 5244 | 1353.406644 | 5575.645744 | 2.042543524 | 2 | 12-oxophytodienoate reductase 2 ame: full=12-oxophytodienoate- -reductase 2� |
| DY305806 | S34125368 | 1387 | 273 | 1304.499663 | 290.2653105 | -2.168052501 | 2 | probable sarcosine oxidase |
| EY722375 | S44259274 | 176 | 18 | 165.5313199 | 19.13837212 | -3.112564186 | 2 | s-adenosylmethionine decarboxylase proenzyme� |
| EY744544 | S44275220 | 82 | 6 | 77.12254679 | 6.379457373 | -3.595647073 | 2 | populus trichocarpa histidine kinase osmosensor mrna |
| EY746794 | S44276709 | 14 | 4 | 13.16726409 | 4.252971582 | -1.630412491 | 2 | cyclin-d2-1 ame: full=g1 s-specific cyclin-d2-1� |
| EY656741 | S44285847 | 52 | 13 | 48.90698089 | 13.82215764 | -1.823057569 | 2 | glucan endo- -beta-glucosidase 11 ame: full=(1- |
| CN192429 | S22549656 | 302 | 723 | 284.0366967 | 768.7246135 | 1.436389528 | 1 | sulfate transporter ame: full=ast12 ame: full= 1 |
| CV713259 | S22575531 | 19 | 4 | 17.8698584 | 4.252971582 | -2.070985083 | 1 | acid phosphatase 1 ame: full=apase-1 flags: precursor |
| CV712407 | S22579576 | 37 | 115 | 34.79919794 | 122.272933 | 1.812979116 | 1 | nad h-quinone oxidoreductase subunit chloroplastic ame: full=nad h dehydrogenase subunit h ame: full=nadh-plastoquinone oxidoreductase subunit h ame: full=nadh-plastoquinone oxidoreductase 49 kda subunit |
| CV887297 | S22585971 | 4 | 15 | 3.762075453 | 15.94864343 | 2.083833026 | 1 | patellin-6 |
| CX044293 | S22588309 | 12 | 32 | 11.28622636 | 34.02377266 | 1.59197993 | 1 | adenine phosphoribosyltransferase 2� |
| CX046632 | S22589541 | 61 | 254 | 57.37165066 | 270.0636955 | 2.23488978 | 1 | beta- insoluble isoenzyme cwinv3 ame: full=cell wall invertase 3� |
| CX048001 | S22590174 | 3 | 38 | 2.82155659 | 40.40323003 | 3.839907444 | 1 | nad h-quinone oxidoreductase subunit chloroplastic ame: full=nad h dehydrogenase subunit i� |
| AJ000081 | S22606215 | 672 | 1412 | 632.0286761 | 1501.298968 | 1.248149381 | 1 | glucan endo- -beta- basic isoform ame: full=(1- |
| EU340032 | S43896462 | 226 | 549 | 212.5572631 | 583.7203496 | 1.457425807 | 1 | phospholipase d gamma 1� |
| EY655569 | S44211275 | 12 | 33 | 11.28622636 | 35.08701555 | 1.636374049 | 1 | 3-ketoacyl- synthase 19� |
| EY657719 | S44212753 | 59 | 198 | 55.49061293 | 210.5220933 | 1.923656002 | 1 | serine threonine-protein kinase srk2f ame: full=ost1-kinase-like 5 ame: full=snf1-related kinase� |
| EY671382 | S44222188 | 375 | 1047 | 352.6945737 | 1113.215312 | 1.658241372 | 1 | fructose- - chloroplastic� |
| EY676236 | S44225502 | 20 | 83 | 18.81037727 | 88.24916033 | 2.230053767 | 1 | cysteine-rich receptor-like protein kinase 8� |
| EY678177 | S44226995 | 153 | 530 | 143.8993861 | 563.5187346 | 1.969403138 | 1 | glutamine chloroplastic ame: full=gs2 ame: full=glutamate--ammonia ligase flags: precursor |
| EY689016 | S44234524 | 164 | 48 | 154.2450936 | 51.03565898 | -1.595647073 | 1 | predicted protein [Populus trichocarpa] |
| EY689864 | S44235274 | 96 | 244 | 90.28981088 | 259.4312665 | 1.522717268 | 1 | protein |
| EY694066 | S44238146 | 411 | 118 | 386.5532528 | 125.4626617 | -1.623409103 | 1 | chloroplast-targeted copper |
| EY702054 | S44244580 | 194 | 918 | 182.4606595 | 976.0569781 | 2.419379932 | 1 | tropinone reductase homolog at1g07440 |
| EY710496 | S44251369 | 14 | 48 | 13.16726409 | 51.03565898 | 1.954550009 | 1 | nudix hydrolase mitochondrial� |
| EY751217 | S44279460 | 130 | 21 | 122.2674522 | 22.32810081 | -2.453107959 | 1 | acid phosphatase 1 ame: full=apase-1 flags: precursor |
| EY710292 | S44300247 | 9443 | 26749 | 8881.319626 | 28440.68421 | 1.679110213 | 1 | ribulose bisphosphate carboxylase small chloroplastic� |
| BQ625029 | S22532848 | 156 | 428 | 146.7209427 | 455.0679593 | 1.633007198 | 0 | uncharacterized protein at1g14870 |
| CK932724 | S22536939 | 56 | 176 | 52.66905634 | 187.1307496 | 1.829019127 | 0 | ORF493 [Pinus koraiensis] |
| CK933067 | S22537163 | 76 | 17 | 71.47943361 | 18.07512922 | -1.983522241 | 0 | cgf1004348\_a07 developing fruit juice sac at 38 dafb citrus sinensis cdna clone jsjune0004\_iif\_a07 5 mrna |
| CK933484 | S22537451 | 26 | 4 | 24.45349045 | 4.252971582 | -2.523497287 | 0 | xyloglucan endotransglucosylase hydrolase protein 9� |
| CK933102 | S22537998 | 10 | 3 | 9.405188633 | 3.189728687 | -1.560023163 | 0 | cgf1004347\_f02 developing fruit juice sac at 38 dafb citrus sinensis cdna clone jsjune0004\_if\_f02 5 mrna |
| CK933909 | S22538125 | 81 | 228 | 76.18202793 | 242.4193802 | 1.669982442 | 0 | �gem-like protein 8 |
| CK934608 | S22538786 | 201 | 565 | 189.0442915 | 600.732236 | 1.667997797 | 0 | photosystem ii cp47 chlorophyll apoprotein ame: full=psii 47 kda protein ame: full=protein cp-47 |
| CK934734 | S22538912 | 32 | 7 | 30.09660363 | 7.442700269 | -2.015702647 | 0 | protein |
| CK934732 | S22538916 | 426 | 68 | 400.6610358 | 72.3005169 | -2.470304348 | 0 | cgf1004227\_e03 developing fruit peel at 38 dafb citrus sinensis cdna clone p38dab10002\_iif\_e03 5 mrna |
| CK935574 | S22539458 | 17 | 47 | 15.98882068 | 49.97241609 | 1.644068441 | 0 | protein |
| CK935206 | S22539526 | 2 | 11 | 1.881037727 | 11.69567185 | 2.636374049 | 0 | chloroplast envelope membrane 70 kda heat shock-related protein |
| CK935355 | S22539635 | 166 | 40 | 156.1261313 | 42.52971582 | -1.876168906 | 0 | cgf1004562\_f08 developing fruit 24 dafb citrus sinensis cdna clone t24dab0001\_if\_f08 5 mrna |
| CK935463 | S22539693 | 20 | 59 | 18.81037727 | 62.73133084 | 1.737657385 | 0 | cytokinin-o-glucosyltransferase 1 ame: full=zeatin o-glucosyltransferase 1� |
| CK935639 | S22539754 | 7 | 1 | 6.583632043 | 1.063242896 | -2.630412491 | 0 | leishmania infantum chromosome 3 |
| CK935682 | S22539794 | 111 | 18 | 104.3975938 | 19.13837212 | -2.447548434 | 0 | cgf1004552\_h09 developing fruit 24 dafb citrus sinensis cdna clone t24dab0004\_if\_h09 5 mrna |
| CK936468 | S22540575 | 20 | 75 | 18.81037727 | 79.74321716 | 2.083833026 | 0 | probable mitochondrial chaperone bcs1 ame: full=bcs1-like protein |
| CK935321 | S22540610 | 22 | 20 | 20.69141499 | 21.26485791 | 0.039438907 | 0 | dc900926 yjs citrus unshiu cdna clone yjs0893 5 mrna |
| CK936515 | S22540833 | 7 | 25 | 6.583632043 | 26.58107239 | 2.013443699 | 0 | cgf1004521\_e12 developing fruit flavedo at 80 dafb citrus sinensis cdna clone f80dab0001\_ivf\_e12 5 mrna |
| CK936548 | S22540860 | 3 | 11 | 2.82155659 | 11.69567185 | 2.051411549 | 0 | hat family dimerisation |
| CK936724 | S22540992 | 31 | 77 | 29.15608476 | 81.86970295 | 1.489532661 | 0 | photosystem ii protein l |
| CK937027 | S22541128 | 31 | 3 | 29.15608476 | 3.189728687 | -3.192291379 | 0 | predicted protein [Populus trichocarpa] |
| CK937174 | S22541286 | 1319 | 3584 | 1240.544381 | 3810.662538 | 1.619068504 | 0 | calmodulin-like protein 9� |
| CK937331 | S22541446 | 28 | 84 | 26.33452817 | 89.31240322 | 1.761904932 | 0 | dna-directed rna polymerase subunit beta ame: full=pep ame: full=plastid-encoded rna polymerase subunit beta� |
| CK936555 | S22541558 | 62 | 170 | 58.31216952 | 180.7512922 | 1.632137057 | 0 | cytochrome b6-f complex subunit 4 ame: full=17 kda polypeptide |
| CK937531 | S22541736 | 5 | 15 | 4.702594316 | 15.94864343 | 1.761904932 | 0 | cgf1004779\_d06 developing fruit albedo at 80 dafb in p x2 vector citrus sinensis cdna clone a80dab0003\_iiif\_d06 5 mrna |
| CK938060 | S22542085 | 65 | 31 | 61.13372611 | 32.96052976 | -0.891229072 | 0 | cgf1004478\_g03 developing fruit albedo at 80 dafb in p x2 vector citrus sinensis cdna clone a80dab0002\_ivf\_g03 5 mrna |
| CK938844 | S22542591 | 85 | 26 | 79.94410338 | 27.64431528 | -1.532008787 | 0 | cgf1004437\_d11 developing fruit albedo at 165 dafb citrus sinensis cdna clone a1650002\_iif\_d11 5 mrna |
| CK938890 | S22542633 | 271 | 80 | 254.880612 | 85.05943164 | -1.583278516 | 0 | protease inhibitor seed storage lipid transfer protein family protein |
| CK938959 | S22542713 | 15 | 44 | 14.10778295 | 46.7826874 | 1.729483454 | 0 | 30s ribosomal protein chloroplastic |
| CK938415 | S22542876 | 57 | 7 | 53.60957521 | 7.442700269 | -2.848592661 | 0 | cgf1004443\_b08 developing fruit albedo at 165 dafb citrus sinensis cdna clone a1650001\_ivf\_b08 5 mrna |
| CK938403 | S22543304 | 1990 | 6014 | 1871.632538 | 6394.342774 | 1.772498868 | 0 | cgf1004443\_c08 developing fruit albedo at 165 dafb citrus sinensis cdna clone a1650001\_ivf\_c08 5 mrna |
| CK939844 | S22543970 | 110 | 29 | 103.457075 | 30.83404397 | -1.746436288 | 0 | cgf1004744\_h08 developing fruit flavedo at 165 dafb citrus sinensis cdna clone f1650003\_ivf\_h08 5 mrna |
| CN185329 | S22544425 | 1658 | 462 | 1559.380275 | 491.2182177 | -1.666536819 | 0 | pathogenesis-related protein r minor form� |
| CN185598 | S22544557 | 219 | 628 | 205.9736311 | 667.7165384 | 1.69677612 | 0 | ethylene-responsive transcription factor 1b� |
| CN186662 | S22545043 | 247 | 787 | 232.3081592 | 836.7721588 | 1.848795025 | 0 | indole-3-acetic acid-amido synthetase ame: full=auxin-responsive gh3-like protein 4� |
| CN188027 | S22545742 | 98 | 21 | 92.1708486 | 22.32810081 | -2.045449991 | 0 | predicted protein [Populus trichocarpa] |
| CN188208 | S22545836 | 65 | 37 | 61.13372611 | 39.33998713 | -0.635972017 | 0 | thaumatin-like protein 1 flags: precursor |
| CN188086 | S22547394 | 2659 | 712 | 2500.839657 | 757.0289416 | -1.7239922 | 0 | ucrcs05\_0005h16\_f washington navel orange stored fruit pulp cdna library citrus sinensis cdna clone mrna |
| CN186421 | S22547782 | 44 | 12 | 41.38282998 | 12.75891475 | -1.697526687 | 0 | predicted protein [Populus trichocarpa] |
| CN191394 | S22549085 | 1651 | 5034 | 1552.796643 | 5352.364736 | 1.785307527 | 0 | protein |
| CN189156 | S22549853 | 84 | 221 | 79.00358452 | 234.9766799 | 1.572527567 | 0 | myb-like protein j |
| CN192158 | S22551120 | 46 | 98 | 43.26386771 | 104.1978038 | 1.268090319 | 0 | solanum lycopersicum clone: htc in fruit |
| CN182786 | S22551989 | 8 | 33 | 7.524150906 | 35.08701555 | 2.22133655 | 0 | flavonol synthase flavanone 3-hydroxylase |
| CN185151 | S22553288 | 275 | 1451 | 258.6426874 | 1542.765441 | 2.576486426 | 0 | probable sulfate transporter |
| CN182947 | S22553931 | 87 | 272 | 81.82514111 | 289.2020676 | 1.821461776 | 0 | �glutamine amidotransferase-like protein yvde homolog |
| CB250354 | S22554978 | 18 | 5 | 16.92933954 | 5.316214478 | -1.671054476 | 0 | est0148 mature fruit abscission zone cdna subtraction library citrus sinensis cdna clone mfaz215 expressed arabidopsis protein mrna |
| CB291312 | S22555642 | 622 | 192 | 585.002733 | 204.1426359 | -1.518865839 | 0 | protein ruptured pollen grain 1 |
| CB291381 | S22555678 | 14 | 3 | 13.16726409 | 3.189728687 | -2.045449991 | 0 | cdgsh iron sulfur domain-containing protein 1 ame: full= |
| CB292519 | S22556318 | 12 | 3 | 11.28622636 | 3.189728687 | -1.823057569 | 0 | ucrcs01\_04ce12\_g1 washington navel orange cold acclimated flavedo & albedo cdna library citrus sinensis cdna clone mrna |
| CB293368 | S22556792 | 120 | 30 | 112.8622636 | 31.89728687 | -1.823057569 | 0 | ucrcs01\_06aa05\_g1 washington navel orange cold acclimated flavedo & albedo cdna library citrus sinensis cdna clone mrna |
| CB291669 | S22557768 | 20 | 3 | 18.81037727 | 3.189728687 | -2.560023163 | 0 | ucrcs01\_03bb10\_g1 washington navel orange cold acclimated flavedo & albedo cdna library citrus sinensis cdna clone mrna |
| CB304738 | S22559035 | 23 | 2 | 21.63193386 | 2.126485791 | -3.346619525 | 0 | flavedo0002\_i \_b08 flavedo mature citrus sinensis cdna clone flavedo0002\_i \_b08 3 mrna |
| CB304782 | S22559079 | 38 | 207 | 35.73971681 | 220.0912794 | 2.622501875 | 0 | conserved hypothetical protein [Ricinus communis] |
| CB610913 | S22560246 | 114 | 23 | 107.2191504 | 24.4545866 | -2.132385627 | 0 | albedo0002\_ii \_h06 mature albedo citrus sinensis cdna clone albedo0002\_ii \_h06 5 mrna |
| CF417791 | S22561156 | 18 | 5 | 16.92933954 | 5.316214478 | -1.671054476 | 0 | cytochrome p450 716b1 ame: full=cytochrome p450 cypa1 |
| CF509967 | S22567816 | 72 | 204 | 67.71735816 | 216.9015507 | 1.679442771 | 0 | sugar carrier protein c |
| CF833645 | S22568937 | 1 | 19 | 0.940518863 | 20.20161501 | 4.424869944 | 0 | protein |
| CF832109 | S22569827 | 111 | 62 | 104.3975938 | 65.92105952 | -0.663277125 | 0 | ucrcs02\_01f08\_r ruby orange ovary at anthesis cdna library citrus sinensis cdna clone mrna |
| CF834253 | S22570867 | 60 | 10 | 56.4311318 | 10.63242896 | -2.40802007 | 0 | hat dimerisation domain-containing protein |
| CF835215 | S22571322 | 6 | 22 | 5.64311318 | 23.3913437 | 2.051411549 | 0 | cytochrome p450 71d9 ame: full=p450 cp3 |
| CF838727 | S22573043 | 23 | 10 | 21.63193386 | 10.63242896 | -1.02469143 | 0 | citrus sinensis dna binding protein (v03-2) complete cds |
| CF838764 | S22573058 | 94 | 568 | 88.40877315 | 603.9219647 | 2.772100699 | 0 | �beta-d-xylosidase ame: full= z152 |
| CF838891 | S22573122 | 18 | 43 | 16.92933954 | 45.71944451 | 1.433282184 | 0 | pentatricopeptide repeat-containing protein at1g31920 |
| CF838214 | S22574550 | 25 | 2 | 23.51297158 | 2.126485791 | -3.466913759 | 0 | disease resistance response protein 206 |
| CF838393 | S22574639 | 158 | 450 | 148.6019804 | 478.459303 | 1.686942874 | 0 | endochitinase a� |
| CF838525 | S22574705 | 301 | 866 | 283.0961779 | 920.7683475 | 1.701545969 | 0 | protein |
| CV714851 | S22576436 | 1 | 1 | 0.940518863 | 1.063242896 | 0.176942431 | 0 | ucrcs08\_0004o10\_f parent washington navel orange callus cdna library ucrcs08-1 citrus sinensis cdna clone mrna |
| CV715341 | S22576716 | 48 | 165 | 45.14490544 | 175.4350778 | 1.958302144 | 0 | triacylglycerol lipase like protein |
| CV715912 | S22577041 | 91 | 125 | 85.58721656 | 132.9053619 | 0.634932075 | 0 | ucrcs08\_0006i04\_r parent washington navel orange callus cdna library ucrcs08-1 citrus sinensis cdna clone mrna |
| CV717430 | S22577908 | 41 | 119 | 38.5612734 | 126.5259046 | 1.71420819 | 0 | cytokinin-o-glucosyltransferase 2 ame: full=zeatin o-glucosyltransferase 2� |
| CV718169 | S22578327 | 7 | 34 | 6.583632043 | 36.15025845 | 2.45705035 | 0 | probable lrr receptor-like serine threonine-protein kinase at3g47570 flags: precursor |
| CV718209 | S22578347 | 370 | 1530 | 347.9919794 | 1626.76163 | 2.224876908 | 0 | phosphatidylglycerol specific phospholipase c |
| CV718293 | S22578395 | 4 | 22 | 3.762075453 | 23.3913437 | 2.636374049 | 0 | fragaria vesca americana clone fosmid complete sequence |
| CV719008 | S22578804 | 45 | 16 | 42.32334885 | 17.01188633 | -1.314910666 | 0 | ucrcs08\_0011f06\_r parent washington navel orange callus cdna library ucrcs08-1 citrus sinensis cdna clone mrna |
| CV719359 | S22579005 | 145 | 410 | 136.3752352 | 435.9295872 | 1.67651344 | 0 | cysteine-rich receptor-like protein kinase 10� |
| CV712647 | S22579680 | 1848 | 1080 | 1738.078859 | 1148.302327 | -0.597991014 | 0 | nucleosome chromatin assembly factor group |
| CV713065 | S22579858 | 1155 | 3069 | 1086.299287 | 3263.092446 | 1.586818225 | 0 | taxadien-5-alpha-ol o-acetyltransferase ame: full=taxa-4 -dien-5alpha-ol-o-acetyltransferase� |
| CV715155 | S22580755 | 61 | 13 | 57.37165066 | 13.82215764 | -2.053355189 | 0 | early nodulin-93� |
| CV717128 | S22581594 | 111 | 33 | 104.3975938 | 35.08701555 | -1.573079316 | 0 | peptidase m50 |
| CV718524 | S22582188 | 10 | 42 | 9.405188633 | 44.65620161 | 2.247331759 | 0 | photosystem ii reaction center protein m� |
| CV718596 | S22582220 | 23 | 3 | 21.63193386 | 3.189728687 | -2.761657025 | 0 | protein |
| CV718780 | S22582298 | 6 | 21 | 5.64311318 | 22.32810081 | 1.984297353 | 0 | plz12\_luppoprotein pplz12 |
| CV719618 | S22582656 | 12 | 2 | 11.28622636 | 2.126485791 | -2.40802007 | 0 | ucrcs08\_0012d12\_r parent washington navel orange callus cdna library ucrcs08-1 citrus sinensis cdna clone mrna |
| CV885563 | S22583523 | 543 | 1440 | 510.7017428 | 1531.06977 | 1.583987139 | 0 | protein notum homolog flags: precursor |
| CV886253 | S22583865 | 77 | 248 | 72.41995247 | 263.6842381 | 1.864352201 | 0 | linalool chloroplastic flags: precursor |
| CV886691 | S22584063 | 36 | 7 | 33.85867908 | 7.442700269 | -2.185627649 | 0 | methyltransferase-like protein 13 |
| CV885307 | S22584971 | 7 | 17 | 6.583632043 | 18.07512922 | 1.45705035 | 0 | vitis vinifera contig whole genome shotgun sequence |
| CV886949 | S22585789 | 385 | 233 | 362.0997624 | 247.7355947 | -0.54758606 | 0 | blue copper protein flags: precursor |
| CX043477 | S22586216 | 39 | 335 | 36.68023567 | 356.18637 | 3.279557497 | 0 | laccase-8 ame: full=benzenediol:oxygen oxidoreductase 8 ame: full=urishiol oxidase 8 ame: full=diphenol oxidase 8 flags: precursor |
| CX043703 | S22586323 | 380 | 798 | 357.3971681 | 848.4678306 | 1.247331759 | 0 | acidic endochitinase flags: precursor |
| CX043988 | S22586454 | 19 | 5 | 17.8698584 | 5.316214478 | -1.749056988 | 0 | meiosis 5 |
| CX045546 | S22587179 | 1083 | 2784 | 1018.581929 | 2960.068221 | 1.539068399 | 0 | brassinosteroid lrr receptor kinase ame: full=tbri1 ame: full=altered brassinolide sensitivity 1 ame: full=systemin receptor sr160 flags: precursor |
| CX043666 | S22587969 | 143 | 557 | 134.4941975 | 592.2262928 | 2.138604611 | 0 | probable glutathione s-transferase ame: full=pathogenesis-related protein 1 |
| CX043799 | S22588043 | 21 | 65 | 19.75089613 | 69.11078821 | 1.806992821 | 0 | ethylene-responsive transcription factor 1b� |
| CX044553 | S22588449 | 50 | 20 | 47.02594316 | 21.26485791 | -1.144985664 | 0 | ucrcs07\_18c09\_b parent washington navel orange thrip-challenged flavedo cdna library ucrcs07 citrus sinensis cdna clone ucrcs07-18c09-f18-1- mrna |
| CX044628 | S22588487 | 4 | 30 | 3.762075453 | 31.89728687 | 3.083833026 | 0 | 3 -n-debenzoyl-2 -deoxytaxol n-benzoyltransferase� |
| CX045330 | S22588860 | 118 | 1894 | 110.9812259 | 2013.782044 | 4.181519997 | 0 | ucrcs07\_22b08\_b parent washington navel orange thrip-challenged flavedo cdna library ucrcs07 citrus sinensis cdna clone ucrcs07-22b08-d16-1- mrna |
| CX046578 | S22589514 | 83 | 213 | 78.06306565 | 226.4707367 | 1.53661262 | 0 | disease resistance protein at4g27190 |
| CX046670 | S22589560 | 20 | 49 | 18.81037727 | 52.09890188 | 1.46972418 | 0 | oxygen-evolving enhancer protein 3- chloroplastic� |
| CX046862 | S22589651 | 182 | 746 | 171.1744331 | 793.1792001 | 2.212179611 | 0 | ribulose bisphosphate carboxylase large chain� |
| CX046913 | S22589674 | 85 | 11 | 79.94410338 | 11.69567185 | -2.773016887 | 0 | lea34\_goshilate embryogenesis abundant protein d-34 (lea d-34) |
| CX047303 | S22589854 | 17 | 2 | 15.98882068 | 2.126485791 | -2.91052041 | 0 | ucrcs09\_13d02\_b ruby orange developing seed cdna library ucrcs09 citrus sinensis cdna clone ucrcs09-13d02-g4-1- mrna |
| CX047339 | S22589869 | 25 | 6 | 23.51297158 | 6.379457373 | -1.881951258 | 0 | ucrcs09\_26b02\_b ruby orange developing seed cdna library ucrcs09 citrus sinensis cdna clone ucrcs09-26b02-d3-1- mrna |
| CX047858 | S22590108 | 21 | 2 | 19.75089613 | 2.126485791 | -3.215374992 | 0 | hypothetical protein Cagg\_1305 [Chloroflexus aggregans DSM 9485] |
| CX047991 | S22590171 | 11 | 29 | 10.3457075 | 30.83404397 | 1.575491807 | 0 | ribosomal protein mitochondrial |
| CX048448 | S22590387 | 8 | 39 | 7.524150906 | 41.46647293 | 2.46234465 | 0 | salt tolerance protein |
| CX048690 | S22590496 | 29 | 88 | 27.27504704 | 93.56537481 | 1.778393054 | 0 | 60s ribosomal protein mitochondrial |
| CX049956 | S22591084 | 7 | 29 | 6.583632043 | 30.83404397 | 2.227568504 | 0 | vitis vinifera contig whole genome shotgun sequence |
| CX050437 | S22591308 | 88 | 246 | 82.76565997 | 261.5577523 | 1.660025318 | 0 | �cytochrome c biosynthesis ccmc-like mitochondrial protein ame: full=abc transporter i family member 3� |
| CX051396 | S22591749 | 5 | 26 | 4.702594316 | 27.64431528 | 2.555454054 | 0 | ac009360\_9this gene is cut |
| CX053113 | S22592544 | 72 | 228 | 67.71735816 | 242.4193802 | 1.839907444 | 0 | cytochrome c oxidase subunit 3 ame: full=cytochrome c oxidase polypeptide iii |
| CX047752 | S22593544 | 11 | 4 | 10.3457075 | 4.252971582 | -1.282489188 | 0 | ucrcs09\_16c11\_b ruby orange developing seed cdna library ucrcs09 citrus sinensis cdna clone ucrcs09-16c11-e21-1- mrna |
| CX048047 | S22593702 | 56 | 135 | 52.66905634 | 143.5377909 | 1.446403106 | 0 | protein ycf2 |
| CX048727 | S22594065 | 8 | 29 | 7.524150906 | 30.83404397 | 2.034923426 | 0 | ribosomal protein mitochondrial |
| CX048969 | S22594194 | 27 | 90 | 25.39400931 | 95.6918606 | 1.913908025 | 0 | arabidopsis thaliana mitochondrial genome |
| CX049508 | S22594486 | 45 | 4 | 42.32334885 | 4.252971582 | -3.314910666 | 0 | ucrcs09\_28a02\_b ruby orange developing seed cdna library ucrcs09 citrus sinensis cdna clone ucrcs09-28a02-a3-1- mrna |
| CX049594 | S22594531 | 1 | 17 | 0.940518863 | 18.07512922 | 4.264405272 | 0 | ucrcs09\_28e03\_b ruby orange developing seed cdna library ucrcs09 citrus sinensis cdna clone ucrcs09-28e03-i5-1- mrna |
| CX051790 | S22595708 | 17 | 2 | 15.98882068 | 2.126485791 | -2.91052041 | 0 | ucrcs09\_6c06\_b ruby orange developing seed cdna library ucrcs09 citrus sinensis cdna clone ucrcs09-6c06-f11-1- mrna |
| CX052259 | S22595961 | 6 | 20 | 5.64311318 | 21.26485791 | 1.913908025 | 0 | protein ycf2 |
| CX069789 | S22597106 | 9 | 3 | 8.46466977 | 3.189728687 | -1.40802007 | 0 | retrovirus-related pol polyprotein from transposon tnt 1-94 includes: ame: full=protease includes: ame: full=reverse transcriptase includes: ame: full=endonuclease |
| CX071698 | S22597989 | 453 | 98 | 426.0550451 | 104.1978038 | -2.031714965 | 0 | ucrcs08\_23d06\_g parent washington navel orange callus cdna library ucrcs08-2 citrus sinensis cdna clone ucrcs08-23d06-h11-1- mrna |
| CX072609 | S22598410 | 8268 | 32247 | 7776.209962 | 34286.39365 | 2.140497087 | 0 | predicted protein [Populus trichocarpa] |
| CX074987 | S22599512 | 55 | 13 | 51.72853748 | 13.82215764 | -1.903977565 | 0 | chloroplast-targeted copper |
| CX075508 | S22599753 | 501 | 142 | 471.1999505 | 150.9804912 | -1.641977243 | 0 | ethylene-responsive transcription factor erf012 |
| CX077188 | S22600532 | 9 | 3 | 8.46466977 | 3.189728687 | -1.40802007 | 0 | ucrcs08\_55a08\_b parent washington navel orange callus cdna library ucrcs08-2 citrus sinensis cdna clone ucrcs08-55a08-b15-1- mrna |
| CX077201 | S22600538 | 136 | 345 | 127.9105654 | 366.818799 | 1.519932141 | 0 | ucrcs08\_55b03\_b parent washington navel orange callus cdna library ucrcs08-2 citrus sinensis cdna clone ucrcs08-55b03-d5-1- mrna |
| CX077578 | S22600712 | 45 | 115 | 42.32334885 | 122.272933 | 1.530579385 | 0 | hypothetical protein NitaMp078 [Nicotiana tabacum] |
| CX071017 | S22602114 | 4 | 13 | 3.762075453 | 13.82215764 | 1.877382149 | 0 | protein abscisic acid-insensitive 5 ame: full=dc3 promoter-binding factor 1� |
| CX071630 | S22602442 | 27 | 47 | 25.39400931 | 49.97241609 | 0.97664378 | 0 | arabidopsis thaliana mitochondrial genome |
| CX074583 | S22604029 | 10 | 2 | 9.405188633 | 2.126485791 | -2.144985664 | 0 | legumin type b contains: ame: full=legumin type b alpha chain ame: full=legumin type b acidic chain contains: ame: full=legumin type b beta chain ame: full=legumin type b basic chain flags: precursor |
| CX074829 | S22604160 | 26 | 6 | 24.45349045 | 6.379457373 | -1.938534787 | 0 | afadin- and alpha-actinin-binding |
| CX075279 | S22604401 | 1170 | 1021 | 1100.40707 | 1085.570996 | -0.019583233 | 0 | cyclin-b1-2 ame: full=g2 mitotic-specific cyclin-b1-2� |
| CX076101 | S22604844 | 290 | 80 | 272.7504704 | 85.05943164 | -1.681038564 | 0 | embryogenic cell protein 40� |
| CX076751 | S22605192 | 5 | 18 | 4.702594316 | 19.13837212 | 2.024939337 | 0 | beta vulgaris vulgaris mitochondrial complete genome |
| CX077469 | S22605580 | 82 | 271 | 77.12254679 | 288.1388247 | 1.901539468 | 0 | cytochrome b6 |
| AY242385 | S22606177 | 3114 | 10340 | 2928.77574 | 10993.93154 | 1.908337767 | 0 | allene oxide chloroplastic ame: full=cytochrome p450 74a ame: full=hydroperoxide dehydrase flags: precursor |
| AF255013 | S22606190 | 18 | 67 | 16.92933954 | 71.237274 | 2.07310662 | 0 | trans-cinnamate 4-monooxygenase ame: full=cinnamic acid 4-hydroxylase� |
| AF321533 | S22606192 | 3742 | 11070 | 3519.421586 | 11770.09885 | 1.741716189 | 0 | 1-aminocyclopropane-1-carboxylate oxidase� |
| AF000135 | S22606213 | 4 | 13 | 3.762075453 | 13.82215764 | 1.877382149 | 0 | endoglucanase 1 ame: full=endo- -beta-glucanase 1 ame: full=abscission cellulase 1 flags: precursor |
| CX675318 | S23016762 | 7 | 28 | 6.583632043 | 29.77080107 | 2.176942431 | 0 | PREDICTED: hypothetical protein [Vitis vinifera] |
| CX676285 | S23018062 | 660 | 1681 | 620.7424498 | 1787.311307 | 1.525724226 | 0 | conserved hypothetical protein [Ricinus communis] |
| CX671600 | S23018380 | 2 | 16 | 1.881037727 | 17.01188633 | 3.176942431 | 0 | ucrcs10\_12a11\_b madame vinous sweet orange multiple pathogen-infected cdna library ucrcs10 citrus sinensis cdna clone ucrcs10-12a11-b22- mrna |
| CX671835 | S23018494 | 59 | 16 | 55.49061293 | 17.01188633 | -1.705700619 | 0 | populus trichocarpa mrna |
| CX672887 | S23019003 | 3455 | 2987 | 3249.492673 | 3175.906529 | -0.033046042 | 0 | ucrcs10\_1a11\_b madame vinous sweet orange multiple pathogen-infected cdna library ucrcs10 citrus sinensis cdna clone ucrcs10-1a11-a21- mrna |
| CX673911 | S23019511 | 10 | 2 | 9.405188633 | 2.126485791 | -2.144985664 | 0 | conserved hypothetical protein [Ricinus communis] |
| CX671538 | S23020240 | 187 | 570 | 175.8770274 | 606.0484504 | 1.78486608 | 0 | serine threonine-protein kinase |
| CX673163 | S23020996 | 2 | 15 | 1.881037727 | 15.94864343 | 3.083833026 | 0 | tyrosine-sulfated glycopeptide receptor 1 |
| CX673271 | S23021048 | 134 | 804 | 126.0295277 | 854.847288 | 2.761904932 | 0 | probable lrr receptor-like serine threonine-protein kinase at1g56140 flags: precursor |
| DN620202 | S24240495 | 644 | 89 | 605.694148 | 94.6286177 | -2.678241016 | 0 | populus trichocarpa mrna |
| DN620501 | S24242702 | 23 | 7 | 21.63193386 | 7.442700269 | -1.539264603 | 0 | protein |
| CX302584 | S24635461 | 105 | 29 | 98.75448065 | 30.83404397 | -1.679322092 | 0 | conserved hypothetical protein [Ricinus communis] |
| CX303326 | S24636299 | 717 | 1858 | 674.352025 | 1975.5053 | 1.550647909 | 0 | protein tify 10a ame: full=jasmonate zim domain-containing protein 1 |
| DR403409 | S25678407 | 3 | 10 | 2.82155659 | 10.63242896 | 1.913908025 | 0 | csaf-pnp1244o18 flavedo mature citrus sinensis cdna clone csaf-pnp1244o18 5 mrna |
| DR403848 | S25678846 | 37 | 9 | 34.79919794 | 9.56918606 | -1.862585933 | 0 | csah-pnp1246h10 developing fruit peel at 38 dafb citrus sinensis cdna clone csah-pnp1246h10 5 mrna |
| DR404453 | S25679451 | 77 | 229 | 72.41995247 | 243.4826231 | 1.749359678 | 0 | lrr receptor-like serine threonine-protein kinase gso1 ame: full=protein gassho 1 flags: precursor |
| DR404508 | S25679506 | 94 | 20 | 88.40877315 | 21.26485791 | -2.055718326 | 0 | protein |
| DR405064 | S25680062 | 4440 | 2567 | 4175.903753 | 2729.344513 | -0.61353395 | 0 | csab-pnp1240e07 developing fruit albedo at 165 dafb citrus sinensis cdna clone csab-pnp1240e07 5 mrna |
| DR405291 | S25680289 | 25 | 7 | 23.51297158 | 7.442700269 | -1.659558837 | 0 | csad-pnp1242a02 developing fruit flavedo at 165 dafb citrus sinensis cdna clone csad-pnp1242a02 5 mrna |
| DR405609 | S25680607 | 40 | 11 | 37.62075453 | 11.69567185 | -1.685554045 | 0 | csad-pnp1242p19 developing fruit flavedo at 165 dafb citrus sinensis cdna clone csad-pnp1242p19 5 mrna |
| DR405840 | S25680838 | 66 | 17 | 62.07424498 | 18.07512922 | -1.779988847 | 0 | protein |
| DR406125 | S25681123 | 7 | 2 | 6.583632043 | 2.126485791 | -1.630412491 | 0 | protein |
| DR908284 | S26279161 | 46 | 8 | 43.26386771 | 8.505943164 | -2.346619525 | 0 | transcription factor bhlh68 ame: full=transcription factor en 60 ame: full=bhlh transcription factor bhlh068 ame: full=basic helix-loop-helix protein 68� |
| DR909231 | S26280108 | 74 | 18 | 69.59839588 | 19.13837212 | -1.862585933 | 0 | usda-fp\_17359 citrus sinensis phloem citrus sinensis cdna clone vpe-33\_c10 5 mrna |
| DR909736 | S26280613 | 288 | 69 | 270.8694326 | 73.36375979 | -1.884458114 | 0 | usda-fp\_17864 citrus sinensis phloem citrus sinensis cdna clone vpe-10\_d05 5 mrna |
| DR909768 | S26280645 | 1020 | 2746 | 959.3292406 | 2919.664991 | 1.605704904 | 0 | rotenone-insensitive nadh-ubiquinone mitochondrial |
| DR910154 | S26281031 | 10 | 1 | 9.405188633 | 1.063242896 | -3.144985664 | 0 | cysteine-rich repeat secretory protein 15 flags: precursor |
| DR910614 | S26281491 | 78 | 20 | 73.36047134 | 21.26485791 | -1.786531693 | 0 | cytochrome p450 716b2 ame: full=cytochrome p450 cypa2 |
| DR910642 | S26281519 | 15 | 4 | 14.10778295 | 4.252971582 | -1.729948165 | 0 | vitis whole genome shotgun contig clone entav 115 |
| DR910681 | S26281558 | 38 | 118 | 35.73971681 | 125.4626617 | 1.811657967 | 0 | usda-fp\_18809 citrus sinensis phloem citrus sinensis cdna clone vpe-29\_c08 5 mrna |
| DR910861 | S26281738 | 31 | 1 | 29.15608476 | 1.063242896 | -4.77725388 | 0 | usda-fp\_18989 citrus sinensis phloem citrus sinensis cdna clone vpe-14\_f02 5 mrna |
| DR911102 | S26281979 | 21 | 4 | 19.75089613 | 4.252971582 | -2.215374992 | 0 | usda-fp\_19230 citrus sinensis phloem citrus sinensis cdna clone vpe-45\_d05 5 mrna |
| DR911173 | S26282050 | 51 | 6 | 47.96646203 | 6.379457373 | -2.91052041 | 0 | usda-fp\_19301 citrus sinensis phloem citrus sinensis cdna clone vpe-31\_a06 5 mrna |
| DR911368 | S26282245 | 24 | 6 | 22.57245272 | 6.379457373 | -1.823057569 | 0 | metacaspase-3� |
| DR911419 | S26282296 | 3 | 6 | 2.82155659 | 6.379457373 | 1.176942431 | 0 | populus trichocarpa mrna |
| DR911940 | S26282817 | 15 | 3 | 14.10778295 | 3.189728687 | -2.144985664 | 0 | usda-fp\_20068 citrus sinensis phloem citrus sinensis cdna clone vpe-19\_g07 5 mrna |
| DQ028471 | S32321255 | 517 | 1600 | 486.2482523 | 1701.188633 | 1.80677815 | 0 | 9-cis-epoxycarotenoid dioxygenase chloroplastic ame: full= 1 flags: precursor |
| DY257186 | S34124464 | 41 | 8 | 38.5612734 | 8.505943164 | -2.180609574 | 0 | kn0aak1bf05fm2 ruit citrus sinensis cdna 5 mrna |
| DY257274 | S34124552 | 11 | 29 | 10.3457075 | 30.83404397 | 1.575491807 | 0 | length cdna complete sequence from clone gsltsil38zb08 of silique of strain col-0 of arabidopsis thaliana (thale cress) |
| DY257639 | S34124917 | 24 | 5 | 22.57245272 | 5.316214478 | -2.086091975 | 0 | kn0aak3cf02fm1 ruit citrus sinensis cdna 5 mrna |
| DY305480 | S34125042 | 177 | 517 | 166.4718388 | 549.696577 | 1.723357351 | 0 | probable lrr receptor-like serine threonine-protein kinase at2g16250 flags: precursor |
| DY305498 | S34125060 | 12 | 4 | 11.28622636 | 4.252971582 | -1.40802007 | 0 | protein |
| DY305501 | S34125063 | 13 | 38 | 12.22674522 | 40.40323003 | 1.724430226 | 0 | photosystem i assembly protein ycf4 |
| DY305530 | S34125092 | 61 | 854 | 57.37165066 | 908.0094328 | 3.984297353 | 0 | chlorophyllase- chloroplastic ame: full=chlorophyll-chlorophyllido hydrolase 1� |
| DY305550 | S34125112 | 3172 | 11785 | 2983.325834 | 12530.31752 | 2.070429513 | 0 | polyol transporter 5 ame: full=sugar-proton symporter plt5 ame: full=protein polyol transporter 5� |
| DY305565 | S34125127 | 140 | 33 | 131.6726409 | 35.08701555 | -1.907946467 | 0 | probable lrr receptor-like serine threonine-protein kinase at2g23950 flags: precursor |
| DY305611 | S34125173 | 52 | 456 | 48.90698089 | 484.8387604 | 3.309392727 | 0 | phytosulfokine receptor 1� |
| DY305613 | S34125175 | 186 | 656 | 174.9365086 | 697.4873395 | 1.995335624 | 0 | transcription factor bhlh36 ame: full=transcription factor en 6 ame: full=bhlh transcription factor bhlh036 ame: full=basic helix-loop-helix protein 36� |
| DY305655 | S34125217 | 120 | 324 | 112.8622636 | 344.4906981 | 1.609901838 | 0 | aspartic proteinase nepenthesin-2 ame: full=nepenthesin-ii flags: precursor |
| DY305677 | S34125239 | 107 | 294 | 100.6355184 | 312.5934113 | 1.635147789 | 0 | xylem cysteine proteinase 1� |
| DY305690 | S34125252 | 1021 | 4085 | 960.2697594 | 4343.347228 | 2.177295643 | 0 | probable glutathione s-transferase ame: full=auxin-induced protein pgnt35 pcnt111 |
| DY305698 | S34125260 | 132 | 504 | 124.14849 | 535.8744193 | 2.109828235 | 0 | caffeic acid 3-o-methyltransferase 1 ame: full=s-adenosysl-l-methionine:caffeic acid 3-o-methyltransferase 1� |
| DY305711 | S34125273 | 2 | 12 | 1.881037727 | 12.75891475 | 2.761904932 | 0 | unnamed protein product [Vitis vinifera] |
| DY305720 | S34125282 | 4 | 13 | 3.762075453 | 13.82215764 | 1.877382149 | 0 | citrus sinensis complete genome |
| DY305725 | S34125287 | 812 | 2454 | 763.701317 | 2609.198066 | 1.772526047 | 0 | tropinone reductase homolog at1g07440 |
| DY305735 | S34125297 | 1257 | 383 | 1182.232211 | 407.222029 | -1.537625922 | 0 | zea mays clone mrna sequence |
| DY305779 | S34125341 | 28 | 87 | 26.33452817 | 92.50213191 | 1.812531005 | 0 | alpha galactosidase precursor |
| DY305861 | S34125423 | 39 | 280 | 36.68023567 | 297.7080107 | 3.020823229 | 0 | protein |
| DY305873 | S34125435 | 3016 | 6603 | 2836.604892 | 7020.592839 | 1.307427648 | 0 | ammonium transporter 1 member 1 ame: full= 1 1 |
| DY305902 | S34125464 | 35 | 93 | 32.91816022 | 98.88158928 | 1.586818225 | 0 | expansin-a4� |
| DY305906 | S34125468 | 4589 | 14993 | 4316.041064 | 15941.20073 | 1.884979805 | 0 | conserved hypothetical protein [Escherichia sp. 3\_2\_53FAA] |
| DY306014 | S34125576 | 138 | 935 | 129.7916031 | 994.1321073 | 2.937240529 | 0 | probable lrr receptor-like serine threonine-protein kinase at3g47570 flags: precursor |
| DY306026 | S34125588 | 141 | 32 | 132.6131597 | 34.02377266 | -1.962608922 | 0 | probable wound-induced protein |
| DY306086 | S34125648 | 129 | 468 | 121.3269334 | 497.5976751 | 2.036079895 | 0 | receptor serine threonine |
| DY306114 | S34125676 | 375 | 950 | 352.6945737 | 1010.080751 | 1.517979349 | 0 | homogentisate geranylgeranyl transferase |
| DY306181 | S34125743 | 1794 | 4578 | 1687.290841 | 4867.525976 | 1.528480003 | 0 | protein |
| DY306872 | S34125767 | 36 | 94 | 33.85867908 | 99.94483218 | 1.561606281 | 0 | transcription factor bhlh36 ame: full=transcription factor en 6 ame: full=bhlh transcription factor bhlh036 ame: full=basic helix-loop-helix protein 36� |
| AB276108 | S35152777 | 71 | 190 | 66.77683929 | 202.0161501 | 1.59705092 | 0 | beta- soluble isoenzyme i ame: full=sucrose hydrolase ame: full=invertase ame: full=saccharase flags: precursor |
| EY649916 | S44207078 | 141 | 382 | 132.6131597 | 406.1587861 | 1.614819906 | 0 | prolyl endopeptidase� |
| EY650389 | S44207439 | 9 | 24 | 8.46466977 | 25.51782949 | 1.59197993 | 0 | phytochromobilin:ferredoxin chloroplastic ame: full=phytochromobilin synthase ame: full=pfb synthase ame: full=p synthase flags: precursor |
| EY650597 | S44207647 | 23 | 158 | 21.63193386 | 167.9923775 | 2.957161223 | 0 | cs00-c1-100-012-g05- sweet orange greenhouse plant citrus sinensis mrna |
| EY652559 | S44208951 | 5 | 51 | 4.702594316 | 54.22538767 | 3.527439678 | 0 | maturase k ame: full=intron maturase |
| EY652797 | S44209189 | 6 | 18 | 5.64311318 | 19.13837212 | 1.761904932 | 0 | gamma-tubulin complex component 5-like |
| EY652848 | S44209240 | 10 | 2 | 9.405188633 | 2.126485791 | -2.144985664 | 0 | patatin group d-2 flags: precursor |
| EY652929 | S44209321 | 5 | 23 | 4.702594316 | 24.4545866 | 2.378576292 | 0 | probable esterase at1g33990 |
| EY653765 | S44209765 | 6 | 16 | 5.64311318 | 17.01188633 | 1.59197993 | 0 | acrosin contains: ame: full=acrosin light chain contains: ame: full=acrosin heavy chain flags: precursor |
| EY653900 | S44209900 | 524 | 94 | 492.8318844 | 99.94483218 | -2.301891719 | 0 | cs00-c1-100-049-f01- sweet orange greenhouse plant citrus sinensis mrna |
| EY654875 | S44210791 | 20 | 5 | 18.81037727 | 5.316214478 | -1.823057569 | 0 | probable polygalacturonase� |
| EY655402 | S44211108 | 14 | 38 | 13.16726409 | 40.40323003 | 1.617515022 | 0 | probable lrr receptor-like serine threonine-protein kinase at3g47570 flags: precursor |
| EY655547 | S44211253 | 23 | 173 | 21.63193386 | 183.9410209 | 3.088008702 | 0 | ankyrin repeat-containing protein at2g01680 |
| EY657461 | S44212607 | 365 | 77 | 343.2893851 | 81.86970295 | -2.068023682 | 0 | predicted protein [Populus trichocarpa] |
| EY657722 | S44212756 | 884 | 2431 | 831.4186752 | 2584.743479 | 1.636374049 | 0 | probable phosphatase phospho1 |
| EY658792 | S44213588 | 2 | 45 | 1.881037727 | 47.8459303 | 4.668795527 | 0 | taxadien-5-alpha-ol o-acetyltransferase ame: full=taxa-4 -dien-5alpha-ol-o-acetyltransferase� |
| EY658811 | S44213607 | 37 | 8 | 34.79919794 | 8.505943164 | -2.032510935 | 0 | fasciclin-like arabinogalactan protein 2 flags: precursor |
| EY659666 | S44214140 | 76 | 12 | 71.47943361 | 12.75891475 | -2.486022582 | 0 | Pc13g00140 [Penicillium chrysogenum Wisconsin 54-1255] |
| EY659774 | S44214248 | 6 | 16 | 5.64311318 | 17.01188633 | 1.59197993 | 0 | calcium-dependent protein kinase 2� |
| EY659863 | S44214337 | 57 | 194 | 53.60957521 | 206.2691217 | 1.943965259 | 0 | probable lrr receptor-like serine threonine-protein kinase at3g47570 flags: precursor |
| EY660105 | S44214467 | 11 | 1 | 10.3457075 | 1.063242896 | -3.282489188 | 0 | cs00-c1-101-018-f01- sweet orange infected with xylella fastidiosa (stage 1 of 2) citrus sinensis mrna |
| EY660216 | S44214578 | 4 | 16 | 3.762075453 | 17.01188633 | 2.176942431 | 0 | alpha-glucosidase yihq |
| EY661649 | S44215451 | 101 | 437 | 94.99240519 | 464.6371453 | 2.290220418 | 0 | cytochrome p450 87a3 |
| EY661705 | S44215507 | 34 | 122 | 31.97764135 | 129.7156333 | 2.020216927 | 0 | cs00-c1-101-036-b01- sweet orange infected with xylella fastidiosa (stage 1 of 2) citrus sinensis mrna |
| EY661917 | S44215607 | 26 | 99 | 24.45349045 | 105.2610467 | 2.105859333 | 0 | ornithine decarboxylase� |
| EY662916 | S44216158 | 29 | 79 | 27.27504704 | 83.99618875 | 1.622742184 | 0 | tir-nbs-lrr resistance protein |
| EY663583 | S44216489 | 1 | 12 | 0.940518863 | 12.75891475 | 3.761904932 | 0 | cs00-c1-101-056-g04- sweet orange infected with xylella fastidiosa (stage 1 of 2) citrus sinensis mrna |
| EY664267 | S44217061 | 1 | 12 | 0.940518863 | 12.75891475 | 3.761904932 | 0 | cs00-c1-101-064-g02- sweet orange infected with xylella fastidiosa (stage 1 of 2) citrus sinensis mrna |
| EY664279 | S44217073 | 56 | 14 | 52.66905634 | 14.88540054 | -1.823057569 | 0 | af372895\_1 at2g04793 |
| EY664535 | S44217329 | 5 | 90 | 4.702594316 | 95.6918606 | 4.346867432 | 0 | PREDICTED: hypothetical protein [Vitis vinifera] |
| EY665831 | S44218401 | 147 | 371 | 138.2562729 | 394.4631142 | 1.512545463 | 0 | lrr receptor-like serine threonine-protein kinase gso2 ame: full=protein gassho 2 ame: full=protein embryo sac development arrest 23 flags: precursor |
| EY666904 | S44219152 | 48 | 11 | 45.14490544 | 11.69567185 | -1.948588451 | 0 | ankyrin repeat-containing |
| EY667138 | S44219274 | 440 | 138 | 413.8282998 | 146.7275196 | -1.495892826 | 0 | conserved hypothetical protein [Ricinus communis] |
| EY671042 | S44221960 | 47 | 14 | 44.20438657 | 14.88540054 | -1.570291499 | 0 | cs00-c1-102-075-a04- sweet orange infected with xylella fastidiosa (stage 2 of 2) citrus sinensis mrna |
| EY671339 | S44222145 | 608 | 1545 | 571.8354689 | 1642.710274 | 1.52240604 | 0 | chlorophyll a-b binding chloroplastic ame: full=lhci type ii cab flags: precursor |
| EY672374 | S44222844 | 20 | 6 | 18.81037727 | 6.379457373 | -1.560023163 | 0 | cs00-c1-102-109-d06- sweet orange infected with xylella fastidiosa (stage 2 of 2) citrus sinensis mrna |
| EY674184 | S44223660 | 2289 | 499 | 2152.847678 | 530.5582049 | -2.020663311 | 0 | citrus sinensis dna binding protein (v03-2) complete cds |
| EY674368 | S44223844 | 19 | 58 | 17.8698584 | 61.66808794 | 1.786995913 | 0 | probable receptor-like protein kinase at2g23200 flags: precursor |
| EY674434 | S44223910 | 635 | 1883 | 597.2294782 | 2002.086372 | 1.745146934 | 0 | cellulose synthase-like protein b6� |
| EY675558 | S44224824 | 69 | 21 | 64.89580157 | 22.32810081 | -1.539264603 | 0 | probable pectinesterase pectinesterase inhibitor 54 includes: ame: full=pectinesterase inhibitor 54 ame: full=pectin methylesterase inhibitor 54 includes: ame: full=pectinesterase 54� |
| EY675862 | S44225128 | 19 | 2 | 17.8698584 | 2.126485791 | -3.070985083 | 0 | lachrymatory-factor synthase flags: precursor |
| EY675876 | S44225142 | 213 | 1208 | 200.3305179 | 1284.397418 | 2.68063755 | 0 | probable wrky transcription factor 70 ame: full=wrky dna-binding protein 70 |
| EY676162 | S44225428 | 44 | 127 | 41.38282998 | 135.0318477 | 1.706195499 | 0 | length cdna complete sequence from clone gsltsil77zb11 of silique of strain col-0 of arabidopsis thaliana (thale cress) |
| EY676513 | S44225667 | 127 | 13 | 119.4458956 | 13.82215764 | -3.111302538 | 0 | af283537\_1lectin-related protein precursor |
| EY676719 | S44225873 | 19 | 509 | 17.8698584 | 541.1906338 | 4.920536763 | 0 | aspartic proteinase nepenthesin-1 ame: full=nepenthesin-i flags: precursor |
| EY677031 | S44225961 | 18 | 3 | 16.92933954 | 3.189728687 | -2.40802007 | 0 | probable pectinesterase pectinesterase inhibitor 17 includes: ame: full=pectinesterase inhibitor 17 ame: full=pectin methylesterase inhibitor 17 includes: ame: full=pectinesterase 17� |
| EY677270 | S44226200 | 90 | 17 | 84.6466977 | 18.07512922 | -2.227447824 | 0 | alpha-xylosidase flags: precursor |
| EY677834 | S44226652 | 30 | 40 | 28.2155659 | 42.52971582 | 0.59197993 | 0 | dipterocarpus tempehes microsatellite clone dt29 |
| EY677909 | S44226727 | 3 | 13 | 2.82155659 | 13.82215764 | 2.292419648 | 0 | taxadien-5-alpha-ol o-acetyltransferase ame: full=taxa-4 -dien-5alpha-ol-o-acetyltransferase� |
| EY677942 | S44226760 | 154 | 456 | 144.8399049 | 484.8387604 | 1.743045904 | 0 | aspartic proteinase asp1� |
| EY678170 | S44226988 | 320 | 1131 | 300.9660363 | 1202.527715 | 1.99839755 | 0 | phosphatidylglycerol specific phospholipase c |
| EY678443 | S44227149 | 7446 | 2196 | 7003.103456 | 2334.881399 | -1.584645131 | 0 | cs00-c1-401-048-c12- sweet orange infected with citrus sinensis mrna |
| EY678531 | S44227237 | 25 | 7 | 23.51297158 | 7.442700269 | -1.659558837 | 0 | glucomannan 4-beta-mannosyltransferase 2 ame: full=glucomannan synthase ame: full=mannan synthase 2 ame: full=cellulose synthase-like protein a2� |
| EY678627 | S44227333 | 13 | 1 | 12.22674522 | 1.063242896 | -3.523497287 | 0 | uncharacterized protein at5g43822 |
| EY679306 | S44227900 | 26 | 199 | 24.45349045 | 211.5853362 | 3.113127333 | 0 | predicted protein [Populus trichocarpa] |
| EY679310 | S44227904 | 29 | 4 | 27.27504704 | 4.252971582 | -2.681038564 | 0 | PREDICTED: hypothetical protein [Vitis vinifera] |
| EY679340 | S44227934 | 203 | 55 | 190.9253292 | 58.47835925 | -1.707033773 | 0 | cs00-c1-401-059-b03- sweet orange infected with citrus sinensis mrna |
| EY679388 | S44227982 | 15 | 114 | 14.10778295 | 121.2096901 | 3.102941849 | 0 | aspartic proteinase nepenthesin-1 ame: full=nepenthesin-i flags: precursor |
| EY679398 | S44227992 | 1147 | 2951 | 1078.775136 | 3137.629785 | 1.54028096 | 0 | ap2 erf and b3 domain-containing transcription repressor tem1 ame: full=rav1-like ethylene-responsive transcription factor tem1 ame: full=protein tempranillo 1 |
| EY679601 | S44228195 | 8 | 2 | 7.524150906 | 2.126485791 | -1.823057569 | 0 | cs00-c1-650-002-a04- sweet orange young greenhouse plant citrus sinensis mrna |
| EY680355 | S44228725 | 732 | 187 | 688.4598079 | 198.8264215 | -1.791862948 | 0 | cs00-c1-650-010-b11- sweet orange young greenhouse plant citrus sinensis mrna |
| EY683840 | S44231090 | 53 | 15 | 49.84749975 | 15.94864343 | -1.644087428 | 0 | omega-6 fatty acid endoplasmic reticulum isozyme 2 |
| EY684493 | S44231407 | 325 | 48 | 305.6686306 | 51.03565898 | -2.582390976 | 0 | populus trichocarpa mrna |
| EY685065 | S44231979 | 16 | 4 | 15.04830181 | 4.252971582 | -1.823057569 | 0 | anaeromyxobacter fw109- complete genome |
| EY685151 | S44232065 | 21 | 72 | 19.75089613 | 76.55348848 | 1.954550009 | 0 | glutaredoxin-c6� |
| EY685437 | S44232351 | 6649 | 496 | 6253.509922 | 527.3684762 | -3.567782921 | 0 | cs00-c2-003-019-c01- sweet orange greenhouse plant citrus sinensis mrna |
| EY685507 | S44232421 | 6 | 20 | 5.64311318 | 21.26485791 | 1.913908025 | 0 | protein |
| EY685521 | S44232435 | 193 | 50 | 181.5201406 | 53.16214478 | -1.771658417 | 0 | cs00-c2-003-020-d07- sweet orange greenhouse plant citrus sinensis mrna |
| EY685976 | S44232806 | 189 | 49 | 177.7580652 | 52.09890188 | -1.770590149 | 0 | cs00-c2-003-029-h06- sweet orange greenhouse plant citrus sinensis mrna |
| EY686493 | S44233205 | 14 | 2 | 13.16726409 | 2.126485791 | -2.630412491 | 0 | cs00-c2-003-054-a11- sweet orange greenhouse plant citrus sinensis mrna |
| EY687058 | S44233322 | 14 | 3 | 13.16726409 | 3.189728687 | -2.045449991 | 0 | adp-ribosylation factor 1 |
| EY687309 | S44233573 | 16 | 5 | 15.04830181 | 5.316214478 | -1.501129474 | 0 | nodulin 21 family protein |
| EY687567 | S44233719 | 13 | 67 | 12.22674522 | 71.237274 | 2.542591903 | 0 | disease resistance protein |
| EY688172 | S44234128 | 13 | 2 | 12.22674522 | 2.126485791 | -2.523497287 | 0 | cs00-c2-003-087-b05- sweet orange greenhouse plant citrus sinensis mrna |
| EY690635 | S44235821 | 59 | 7 | 55.49061293 | 7.442700269 | -2.898345696 | 0 | gdsl esterase lipase at5g14450 ame: full=extracellular lipase at5g14450 flags: precursor |
| EY690744 | S44235930 | 32 | 9 | 30.09660363 | 9.56918606 | -1.653132568 | 0 | expression vector complete sequence |
| EY690770 | S44235956 | 2627 | 796 | 2470.743054 | 846.3413448 | -1.545633434 | 0 | ricinus communis histone h1 mrna |
| EY691064 | S44236250 | 3 | 7 | 2.82155659 | 7.442700269 | 1.399334852 | 0 | phosphoinositide phospholipase c 6 ame: full=phosphoinositide phospholipase plc6� |
| EY691269 | S44236455 | 28 | 9 | 26.33452817 | 9.56918606 | -1.46048749 | 0 | trehalose-6-phosphate phosphatase |
| EY691433 | S44236619 | 11304 | 2924 | 10631.62523 | 3108.922226 | -1.773875724 | 0 | homo sapiens chromosome 21 segment hs21c100 |
| EY691434 | S44236620 | 25 | 2 | 23.51297158 | 2.126485791 | -3.466913759 | 0 | citrus sinensis dna binding protein (v03-3) complete cds |
| EY691949 | S44236813 | 355 | 83 | 333.8841965 | 88.24916033 | -1.919693352 | 0 | burp domain-containing protein 6� |
| EY692005 | S44236869 | 1279 | 142 | 1202.923626 | 150.9804912 | -2.994110999 | 0 | cs00-c2-003-086-d08- sweet orange greenhouse plant citrus sinensis mrna |
| EY692051 | S44236915 | 38 | 1 | 35.73971681 | 1.063242896 | -5.070985083 | 0 | dna binding protein |
| EY692353 | S44237217 | 434 | 108 | 408.1851867 | 114.8302327 | -1.829721299 | 0 | cs00-c2-003-059-e06- sweet orange greenhouse plant citrus sinensis mrna |
| EY692550 | S44237302 | 2 | 1 | 1.881037727 | 1.063242896 | -0.823057569 | 0 | annexin a3 ame: full=annexin-3 ame: full=annexin iii ame: full=lipocortin iii ame: full=placental anticoagulant protein iii� |
| EY693559 | S44237751 | 50 | 15 | 47.02594316 | 15.94864343 | -1.560023163 | 0 | protein |
| EY693577 | S44237769 | 256 | 770 | 240.772829 | 818.6970295 | 1.765657066 | 0 | probable indole-3-acetic acid-amido synthetase ame: full=auxin-responsive gh3-like protein 1� |
| EY693754 | S44237834 | 2612 | 1607 | 2456.635271 | 1708.631333 | -0.523842537 | 0 | cs00-c3-700-018-a08- sweet orange development stadium (1 of 6) citrus sinensis mrna |
| EY694299 | S44238281 | 8 | 34 | 7.524150906 | 36.15025845 | 2.264405272 | 0 | conserved hypothetical protein [Ricinus communis] |
| EY695765 | S44239187 | 88 | 239 | 82.76565997 | 254.115052 | 1.61837762 | 0 | receptor-like protein kinase at3g21340 ame: full=leucine-rich repeat receptor-like protein kinase at3g21340 flags: precursor |
| EY697055 | S44240365 | 1 | 19 | 0.940518863 | 20.20161501 | 4.424869944 | 0 | peroxidase 4 flags: precursor |
| EY697902 | S44241100 | 1718 | 515 | 1615.811407 | 547.5700912 | -1.561143268 | 0 | 3 -n-debenzoyl-2 -deoxytaxol n-benzoyltransferase� |
| EY699454 | S44242316 | 94 | 239 | 88.40877315 | 254.115052 | 1.523220387 | 0 | hypothetical protein [Vitis vinifera] |
| EY699595 | S44242457 | 47 | 15 | 44.20438657 | 15.94864343 | -1.470755825 | 0 | zinc transporter 1 ame: full=zrt irt-like protein 1 flags: precursor |
| EY699652 | S44242514 | 9 | 26 | 8.46466977 | 27.64431528 | 1.707457148 | 0 | heat shock -like |
| EY699678 | S44242540 | 5 | 17 | 4.702594316 | 18.07512922 | 1.942477177 | 0 | protein |
| EY699737 | S44242599 | 612 | 2017 | 575.5975443 | 2144.56092 | 1.897549957 | 0 | receptor-like protein kinase feronia ame: full=protein sirene flags: precursor |
| EY701556 | S44244082 | 21 | 4 | 19.75089613 | 4.252971582 | -2.215374992 | 0 | arg7\_phaauindole-3-acetic acid-induced protein arg7 |
| EY701801 | S44244327 | 54 | 4 | 50.78801862 | 4.252971582 | -3.577945071 | 0 | dna binding protein |
| EY701875 | S44244401 | 54 | 478 | 50.78801862 | 508.2301041 | 3.322921737 | 0 | probable lrr receptor-like serine threonine-protein kinase at1g56140 flags: precursor |
| EY701880 | S44244406 | 199 | 26 | 187.1632538 | 27.64431528 | -2.759242472 | 0 | endochitinase flags: precursor |
| EY701988 | S44244514 | 137 | 38 | 128.8510843 | 40.40323003 | -1.673162139 | 0 | cs00-c3-700-055-g07- sweet orange development stadium (1 of 6) citrus sinensis mrna |
| EY702061 | S44244587 | 7 | 1 | 6.583632043 | 1.063242896 | -2.630412491 | 0 | PREDICTED: hypothetical protein [Vitis vinifera] |
| EY702512 | S44244926 | 5 | 36 | 4.702594316 | 38.27674424 | 3.024939337 | 0 | atp synthase subunit chloroplastic ame: full=f-atpase subunit iv ame: full=atp synthase f0 sector subunit a |
| EY703947 | S44246025 | 29 | 210 | 27.27504704 | 223.2810081 | 3.033206953 | 0 | receptor-like protein kinase 2 flags: precursor |
| EY704737 | S44246703 | 14 | 3 | 13.16726409 | 3.189728687 | -2.045449991 | 0 | cs00-c3-701-036-a05- sweet orange development stadium (2 of 6) citrus sinensis mrna |
| EY706402 | S44248144 | 91 | 7 | 85.58721656 | 7.442700269 | -3.523497287 | 0 | major allergen pru ar 1 ame: allergen=pru ar 1 |
| EY706443 | S44248185 | 13 | 2 | 12.22674522 | 2.126485791 | -2.523497287 | 0 | chavicol o-methyltransferase ame: full= eugenol o-methyltransferase cvomt1 ame: full=s-adenosysl-l-methionine: eugenol o-methyltransferase cvomt1 |
| EY706514 | S44248256 | 72 | 3 | 67.71735816 | 3.189728687 | -4.40802007 | 0 | snakin-1 flags: precursor |
| EY707340 | S44249082 | 239 | 52 | 224.7840083 | 55.28863057 | -2.023484659 | 0 | cytochrome p450 76c1 |
| EY707388 | S44249130 | 14 | 4 | 13.16726409 | 4.252971582 | -1.630412491 | 0 | cs00-c3-701-068-b05- sweet orange development stadium (2 of 6) citrus sinensis mrna |
| EY707630 | S44249372 | 16 | 4 | 15.04830181 | 4.252971582 | -1.823057569 | 0 | cs00-c3-701-070-g12- sweet orange development stadium (2 of 6) citrus sinensis mrna |
| EY708405 | S44250035 | 195 | 521 | 183.4011783 | 553.9495486 | 1.594751679 | 0 | proline-rich protein |
| EY710037 | S44251134 | 4467 | 1383 | 4201.297762 | 1470.464925 | -1.514562667 | 0 | germin-like protein subfamily 1 member 8 flags: precursor |
| EY710063 | S44251160 | 16 | 4 | 15.04830181 | 4.252971582 | -1.823057569 | 0 | cs00-c3-701-111-e02- sweet orange development stadium (2 of 6) citrus sinensis mrna |
| EY710067 | S44251164 | 59 | 12 | 55.49061293 | 12.75891475 | -2.120738118 | 0 | flavonol sulfotransferase-like ame: full= 47 |
| EY710406 | S44251279 | 275 | 1859 | 258.6426874 | 1976.568543 | 2.933965677 | 0 | probable lrr receptor-like serine threonine-protein kinase at1g56140 flags: precursor |
| EY710505 | S44251378 | 30 | 98 | 28.2155659 | 104.1978038 | 1.884761679 | 0 | �upf0481 protein at3g02645 |
| EY710580 | S44251453 | 2 | 10 | 1.881037727 | 10.63242896 | 2.498870526 | 0 | PREDICTED: hypothetical protein [Vitis vinifera] |
| EY710632 | S44251505 | 330 | 102 | 310.3712249 | 108.4507753 | -1.516954441 | 0 | beta-amylase ame: full= -alpha-d-glucan maltohydrolase |
| EY710672 | S44251545 | 18 | 4 | 16.92933954 | 4.252971582 | -1.992982571 | 0 | f-box family protein |
| EY710689 | S44251562 | 22 | 6 | 20.69141499 | 6.379457373 | -1.697526687 | 0 | l-ascorbate oxidase homolog ame: full=pollen-specific protein ntp303 flags: precursor |
| EY710905 | S44251666 | 23 | 62 | 21.63193386 | 65.92105952 | 1.607576785 | 0 | chavicol o-methyltransferase ame: full= eugenol o-methyltransferase cvomt1 ame: full=s-adenosysl-l-methionine: eugenol o-methyltransferase cvomt1 |
| EY711189 | S44251950 | 22 | 61 | 20.69141499 | 64.85781663 | 1.64824815 | 0 | chloroplast envelope membrane protein |
| EY712050 | S44252377 | 2 | 11 | 1.881037727 | 11.69567185 | 2.636374049 | 0 | lrr receptor-like serine threonine-protein kinase efr ame: full=elongation factor tu receptor� |
| EY712246 | S44252573 | 18 | 4 | 16.92933954 | 4.252971582 | -1.992982571 | 0 | cs00-c3-702-017-b03- sweet orange development stadium (3 of 6) citrus sinensis mrna |
| EY712805 | S44252908 | 19 | 5 | 17.8698584 | 5.316214478 | -1.749056988 | 0 | cs00-c3-702-023-f07- sweet orange development stadium (3 of 6) citrus sinensis mrna |
| EY713598 | S44253365 | 15 | 37 | 14.10778295 | 39.33998713 | 1.479505201 | 0 | poncirus trifoliata citrus tristeza virus resistance gene complete sequence |
| EY713742 | S44253397 | 48 | 15 | 45.14490544 | 15.94864343 | -1.501129474 | 0 | germin-like protein subfamily 2 member 4 flags: precursor |
| EY715074 | S44254169 | 1415 | 110 | 1330.834192 | 116.9567185 | -3.508284193 | 0 | endochitinase a� |
| EY717756 | S44255633 | 118 | 302 | 110.9812259 | 321.0993544 | 1.532704121 | 0 | ndf6 (ndh dependent flow 6) |
| EY719351 | S44256816 | 202 | 65 | 189.9848104 | 69.11078821 | -1.458901239 | 0 | cs00-c3-702-089-b05- sweet orange development stadium (3 of 6) citrus sinensis mrna |
| EY719506 | S44256971 | 340 | 82 | 319.7764135 | 87.18591743 | -1.874896501 | 0 | fatty acyl- reductase 2 ame: full=fatty acid reductase 2 ame: full=male sterility protein 2 |
| EY719828 | S44257293 | 6 | 16 | 5.64311318 | 17.01188633 | 1.59197993 | 0 | �monooxygenase moxc |
| EY723927 | S44260392 | 20 | 6 | 18.81037727 | 6.379457373 | -1.560023163 | 0 | cs00-c3-703-065-c02- sweet orange development stadium (4 of 6) citrus sinensis mrna |
| EY724756 | S44260997 | 229 | 59 | 215.3788197 | 62.73133084 | -1.779618308 | 0 | cytochrome p450 93a3 ame: full=p450 cp5 |
| EY725567 | S44261578 | 6721 | 1949 | 6321.22728 | 2072.260403 | -1.608999382 | 0 | cold shock protein-1 |
| EY727446 | S44262796 | 70 | 22 | 65.83632043 | 23.3913437 | -1.492908967 | 0 | surface antigen |
| EY728096 | S44263110 | 75 | 16 | 70.53891475 | 17.01188633 | -2.05187626 | 0 | isoflavone-7-o-methyltransferase 9 ame: full=isoflavone-o-methyltransferase 9 ame: full=7 iomt-9 |
| EY728149 | S44263163 | 18 | 5 | 16.92933954 | 5.316214478 | -1.671054476 | 0 | cs00-c3-703-108-b05- sweet orange development stadium (4 of 6) citrus sinensis mrna |
| EY728992 | S44263782 | 51 | 12 | 47.96646203 | 12.75891475 | -1.91052041 | 0 | at3g08600 f17o14\_7 |
| EY730551 | S44265117 | 163 | 418 | 153.3045747 | 444.4355303 | 1.535573409 | 0 | cs00-c3-704-031-d11- sweet orange development stadium (5 of 6) citrus sinensis mrna |
| EY734089 | S44267893 | 31 | 8 | 29.15608476 | 8.505943164 | -1.77725388 | 0 | cs00-c3-704-081-d10- sweet orange development stadium (5 of 6) citrus sinensis mrna |
| EY734795 | S44268487 | 9627 | 1966 | 9054.375097 | 2090.335533 | -2.114880538 | 0 | flocculation protein flo11� |
| EY735453 | S44268697 | 3097 | 7937 | 2912.78692 | 8438.958862 | 1.534664853 | 0 | cellulose synthase-like protein h2 ame: full= slh2 |
| EY737138 | S44269934 | 23 | 1 | 21.63193386 | 1.063242896 | -4.346619525 | 0 | probable 3-beta-hydroxysteroid-delta -isomerase ame: full=cholestenol delta-isomerase ame: full=delta -delta sterol isomerase� |
| EY738927 | S44271605 | 2 | 14 | 1.881037727 | 14.88540054 | 2.984297353 | 0 | �disease resistance rpp13-like protein 3 |
| EY742919 | S44274267 | 14 | 3 | 13.16726409 | 3.189728687 | -2.045449991 | 0 | cs00-c3-705-094-g07- sweet orange development stadium (6 of 6) citrus sinensis mrna |
| EY743861 | S44274537 | 23 | 59 | 21.63193386 | 62.73133084 | 1.536023524 | 0 | auxin-induced protein |
| EY744044 | S44274720 | 1578 | 371 | 1484.138766 | 394.4631142 | -1.911663683 | 0 | isoflavone-7-o-methyltransferase 9 ame: full=isoflavone-o-methyltransferase 9 ame: full=7 iomt-9 |
| EY744529 | S44275205 | 17 | 55 | 15.98882068 | 58.47835925 | 1.870839303 | 0 | mouse dna sequence from clone rp23-247j12 on chromosome complete sequence |
| EY744652 | S44275328 | 13 | 3 | 12.22674522 | 3.189728687 | -1.938534787 | 0 | cs00-c5-003-010-a04- sweet orange greenhouse plant citrus sinensis mrna |
| EY745157 | S44275735 | 128 | 35 | 120.3864145 | 37.21350134 | -1.693774552 | 0 | zinc finger protein constans-like 5 |
| EY745230 | S44275808 | 17 | 3 | 15.98882068 | 3.189728687 | -2.32555791 | 0 | protein |
| EY745851 | S44276331 | 33 | 10 | 31.03712249 | 10.63242896 | -1.545523594 | 0 | predicted protein [Populus trichocarpa] |
| EY746754 | S44276669 | 27 | 7 | 25.39400931 | 7.442700269 | -1.770590149 | 0 | cs00-c5-003-037-c07- sweet orange greenhouse plant citrus sinensis mrna |
| EY746807 | S44276722 | 104529 | 28803 | 98311.49626 | 30624.58512 | -1.682669831 | 0 | cs00-c5-003-037-h06- sweet orange greenhouse plant citrus sinensis mrna |
| EY746812 | S44276727 | 730 | 101 | 686.5787702 | 107.3875324 | -2.67659874 | 0 | pectinesterase pectinesterase inhibitor includes: ame: full=pectinesterase inhibitor ame: full=pectin methylesterase inhibitor includes: ame: full=pectinesterase� |
| EY746898 | S44276813 | 4 | 12 | 3.762075453 | 12.75891475 | 1.761904932 | 0 | transcription factor myb39 ame: full=myb-related protein 39� |
| EY747045 | S44276960 | 338 | 48 | 317.8953758 | 51.03565898 | -2.638974505 | 0 | miraculin� |
| EY747509 | S44277200 | 58 | 2 | 54.55009407 | 2.126485791 | -4.681038564 | 0 | defensin-like protein 6 ame: full=plant defensin ame: full=low-molecular-weight cysteine-rich protein 74� |
| EY748161 | S44277628 | 29 | 8 | 27.27504704 | 8.505943164 | -1.681038564 | 0 | catalytic, putative [Ricinus communis] |
| EY748700 | S44277831 | 54 | 277 | 50.78801862 | 294.5182821 | 2.535797095 | 0 | bahd acyltransferase at5g47980 |
| EY748923 | S44278054 | 52 | 15 | 48.90698089 | 15.94864343 | -1.616606692 | 0 | cs00-c5-003-064-b03- sweet orange greenhouse plant citrus sinensis mrna |
| EY749305 | S44278114 | 490 | 1375 | 460.854243 | 1461.958981 | 1.665520395 | 0 | protein srg1� |
| EY749464 | S44278273 | 11 | 3 | 10.3457075 | 3.189728687 | -1.697526687 | 0 | cs00-c5-003-070-b11- sweet orange greenhouse plant citrus sinensis mrna |
| EY749875 | S44278460 | 20253 | 1903 | 19048.32854 | 2023.35123 | -3.234845727 | 0 | cs00-c5-003-080-g09- sweet orange greenhouse plant citrus sinensis mrna |
| EY750256 | S44278841 | 106 | 278 | 99.69499951 | 295.581525 | 1.567963049 | 0 | monoglyceride lipase� |
| EY750814 | S44279063 | 1 | 10 | 0.940518863 | 10.63242896 | 3.498870526 | 0 | lrr receptor-like serine threonine-protein kinase rch1 ame: full=protein root clavata-homolog1 1 flags: precursor |
| EY751088 | S44279331 | 678 | 43 | 637.6717893 | 45.71944451 | -3.801934278 | 0 | non-specific lipid-transfer protein� |
| EY751229 | S44279472 | 671 | 1786 | 631.0881573 | 1898.951811 | 1.58928984 | 0 | thaumatin-like protein 1 flags: precursor |
| EY751795 | S44279926 | 1779 | 495 | 1673.183058 | 526.3052333 | -1.668623649 | 0 | cs00-c5-003-086-c02- sweet orange greenhouse plant citrus sinensis mrna |
| EY751843 | S44279974 | 23 | 6 | 21.63193386 | 6.379457373 | -1.761657025 | 0 | protein |
| EY752482 | S44280495 | 244 | 1111 | 229.4866026 | 1181.262857 | 2.363848195 | 0 | geranylgeranyl pyrophosphate synthetase chloroplastic� |
| EY753169 | S44280986 | 15 | 5 | 14.10778295 | 5.316214478 | -1.40802007 | 0 | sni1 transcription repressor |
| EY753251 | S44281068 | 38 | 122 | 35.73971681 | 129.7156333 | 1.859752255 | 0 | probable wrky transcription factor 40 ame: full=wrky dna-binding protein 40 |
| EY753520 | S44281337 | 47 | 322 | 44.20438657 | 342.3642124 | 2.953270457 | 0 | serine threonine-protein kinase bri1-like 2 ame: full=brassinosteroid insensitive 1-like protein 2 ame: full=protein vascular highway 1 flags: precursor |
| EY754123 | S44281716 | 114 | 408 | 107.2191504 | 433.8031014 | 2.016477759 | 0 | �cysteine-rich receptor-like protein kinase 20� |
| EY754293 | S44281886 | 16 | 41 | 15.04830181 | 43.59295872 | 1.534494435 | 0 | dynein light chain cytoplasmic ame: full=8 kda dynein light chain |
| EY754954 | S44282239 | 9 | 48 | 8.46466977 | 51.03565898 | 2.59197993 | 0 | cysteine-rich receptor-like protein kinase 22� |
| EY755001 | S44282286 | 173 | 104 | 162.7097633 | 110.5772611 | -0.557246079 | 0 | chromatin remodelling complex atpase chain isw- |
| EY755595 | S44282432 | 33 | 108 | 31.03712249 | 114.8302327 | 1.887435814 | 0 | cs12-c1-001-030-c09- sweet orange field plant a citrus sinensis mrna |
| EY756862 | S44283139 | 2 | 13 | 1.881037727 | 13.82215764 | 2.877382149 | 0 | hypothetical protein [Vitis vinifera] |
| EY756883 | S44283160 | 28 | 10 | 26.33452817 | 10.63242896 | -1.308484396 | 0 | cs13-c1-001-014-g05- sweet orange field plant b citrus sinensis mrna |
| EY650724 | S44284184 | 1 | 11 | 0.940518863 | 11.69567185 | 3.636374049 | 0 | af506028\_20nbs-lrr type disease resistance protein |
| EY650767 | S44284227 | 10 | 27 | 9.405188633 | 28.70755818 | 1.609901838 | 0 | cs00-c1-100-014-f01- sweet orange greenhouse plant citrus sinensis mrna |
| EY652026 | S44284688 | 37 | 70 | 34.79919794 | 74.42700269 | 1.096772082 | 0 | at3g19540 t31j18\_4 |
| EY653480 | S44285036 | 9 | 23 | 8.46466977 | 24.4545866 | 1.530579385 | 0 | cs00-c1-100-044-h08- sweet orange greenhouse plant citrus sinensis mrna |
| EY655144 | S44285440 | 2 | 20 | 1.881037727 | 21.26485791 | 3.498870526 | 0 | protein ycf2 |
| EY656636 | S44285742 | 11 | 57 | 10.3457075 | 60.60484504 | 2.550400826 | 0 | (+)-delta-cadinene synthase isozyme xc14� |
| EY660781 | S44286877 | 49 | 92 | 46.0854243 | 97.81834639 | 1.085794543 | 0 | cs00-c1-101-025-h08- sweet orange infected with xylella fastidiosa (stage 1 of 2) citrus sinensis mrna |
| EY660964 | S44287060 | 28 | 189 | 26.33452817 | 200.9529073 | 2.931829933 | 0 | protein srg1� |
| EY663234 | S44288126 | 6 | 17 | 5.64311318 | 18.07512922 | 1.679442771 | 0 | cs00-c1-101-053-a03- sweet orange infected with xylella fastidiosa (stage 1 of 2) citrus sinensis mrna |
| EY664665 | S44288451 | 174 | 46 | 163.6502822 | 48.90917319 | -1.742439109 | 0 | upf0497 membrane protein at3g53850 |
| EY664687 | S44288473 | 14335 | 2314 | 13482.33791 | 2460.34406 | -2.454138704 | 0 | cs00-c1-101-069-c09- sweet orange infected with xylella fastidiosa (stage 1 of 2) citrus sinensis mrna |
| EY664724 | S44288510 | 283 | 68 | 266.1668383 | 72.3005169 | -1.880252971 | 0 | cs00-c1-101-069-g02- sweet orange infected with xylella fastidiosa (stage 1 of 2) citrus sinensis mrna |
| EY664805 | S44288591 | 377 | 1718 | 354.5756115 | 1826.651294 | 2.365036039 | 0 | probable wrky transcription factor 70 ame: full=wrky dna-binding protein 70 |
| EY665961 | S44288739 | 972 | 2193 | 914.1843351 | 2331.69167 | 1.350820024 | 0 | multidrug resistance |
| EY667647 | S44289305 | 534 | 143 | 502.237073 | 152.0437341 | -1.723882164 | 0 | thaumatin-like protein 1 ame: full= 44 flags: precursor |
| EY667967 | S44289513 | 13 | 3 | 12.22674522 | 3.189728687 | -1.938534787 | 0 | �ac9 transposase |
| EY672037 | S44290671 | 323 | 963 | 303.7875928 | 1023.902908 | 1.752944064 | 0 | purple acid phosphatase 10 flags: precursor |
| EY673577 | S44291329 | 90 | 23 | 84.6466977 | 24.4545866 | -1.791348709 | 0 | uncharacterized oxidoreductase |
| EY673801 | S44291553 | 131 | 360 | 123.2079711 | 382.7674424 | 1.635372526 | 0 | nac domain ipr003441 |
| EY673884 | S44291636 | 56 | 216 | 52.66905634 | 229.6604654 | 2.124475011 | 0 | primary amine oxidase ame: full=amine oxidase flags: precursor |
| EY674821 | S44291873 | 1 | 12 | 0.940518863 | 12.75891475 | 3.761904932 | 0 | briggsae cbr-abt-1 protein |
| EY676846 | S44292120 | 11 | 35 | 10.3457075 | 37.21350134 | 1.846793829 | 0 | cs00-c1-401-027-g02- sweet orange infected with citrus sinensis mrna |
| EY676885 | S44292159 | 68 | 217 | 63.9552827 | 230.7237083 | 1.851030822 | 0 | serine threonine-protein kinase sapk2 ame: full=osmotic stress abscisic acid-activated protein kinase 2 |
| EY677532 | S44292372 | 4 | 21 | 3.762075453 | 22.32810081 | 2.569259854 | 0 | cs00-c1-401-038-g09- sweet orange infected with citrus sinensis mrna |
| EY681754 | S44293248 | 12 | 1 | 11.28622636 | 1.063242896 | -3.40802007 | 0 | remorin ame: full=dna-binding protein |
| EY681774 | S44293268 | 2106 | 535 | 1980.732726 | 568.8349491 | -1.799952209 | 0 | superoxide dismutase 1 |
| EY682325 | S44293497 | 241 | 76 | 226.6650461 | 80.80646006 | -1.488019392 | 0 | cs00-c1-650-031-f08- sweet orange young greenhouse plant citrus sinensis mrna |
| EY683167 | S44293681 | 140 | 42 | 131.6726409 | 44.65620161 | -1.560023163 | 0 | cs00-c1-650-045-a04- sweet orange young greenhouse plant citrus sinensis mrna |
| EY683346 | S44293860 | 34 | 9 | 31.97764135 | 9.56918606 | -1.740595409 | 0 | cs00-c1-650-049-a08- sweet orange young greenhouse plant citrus sinensis mrna |
| EY683931 | S44293997 | 113 | 27 | 106.2786316 | 28.70755818 | -1.888349029 | 0 | nicotianamine synthase ame: full=s-adenosyl-l-methionine:s-adenosyl-l-methionine:s-adenosyl-methionine 3-amino-3-carboxypropyltransferase ame: full=chloronerva |
| EY684007 | S44294073 | 16 | 75 | 15.04830181 | 79.74321716 | 2.405761121 | 0 | �cysteine-rich receptor-like protein kinase 30� |
| EY684012 | S44294078 | 12 | 115 | 11.28622636 | 122.272933 | 3.437469981 | 0 | protein brassinosteroid insensitive 1� |
| EY684201 | S44294267 | 96 | 272 | 90.28981088 | 289.2020676 | 1.679442771 | 0 | �domain-containing gpi-anchored protein 2 flags: precursor |
| EY685678 | S44294372 | 202 | 25 | 189.9848104 | 26.58107239 | -2.837412862 | 0 | mus musculus bac clone rp23-108h19 from complete sequence |
| EY686837 | S44294753 | 14 | 4 | 13.16726409 | 4.252971582 | -1.630412491 | 0 | udp-glucuronate 4-epimerase 4 ame: full=udp-glucuronic acid epimerase 4� |
| EY686851 | S44294767 | 18 | 3 | 16.92933954 | 3.189728687 | -2.40802007 | 0 | cs00-c2-003-014-h01- sweet orange greenhouse plant citrus sinensis mrna |
| EY686862 | S44294778 | 427 | 1134 | 401.6015546 | 1205.717444 | 1.586055096 | 0 | cs00-c2-003-030-a03- sweet orange greenhouse plant citrus sinensis mrna |
| EY687702 | S44295142 | 3919 | 481 | 3685.893425 | 511.4198327 | -2.849434343 | 0 | cs00-c2-003-092-e04- sweet orange greenhouse plant citrus sinensis mrna |
| EY688459 | S44295451 | 75 | 23 | 70.53891475 | 24.4545866 | -1.528314304 | 0 | lysine histidine transporter-like 6 |
| EY688594 | S44295586 | 6 | 19 | 5.64311318 | 20.20161501 | 1.839907444 | 0 | cs00-c2-003-090-a09- sweet orange greenhouse plant citrus sinensis mrna |
| EY690065 | S44295811 | 5 | 45 | 4.702594316 | 47.8459303 | 3.346867432 | 0 | cs00-c2-003-083-h12- sweet orange greenhouse plant citrus sinensis mrna |
| EY690294 | S44295928 | 14 | 2 | 13.16726409 | 2.126485791 | -2.630412491 | 0 | cs00-c2-003-084-a04- sweet orange greenhouse plant citrus sinensis mrna |
| EY691630 | S44296046 | 5 | 29 | 4.702594316 | 30.83404397 | 2.712995331 | 0 | ubiquitin carboxyl-terminal hydrolase 12 ame: full=ubiquitin thioesterase 12 ame: full=ubiquitin-specific-processing protease 12 ame: full=deubiquitinating enzyme 12� |
| EY691718 | S44296134 | 13 | 3 | 12.22674522 | 3.189728687 | -1.938534787 | 0 | endochitinase 1 flags: precursor |
| EY691800 | S44296216 | 194 | 3 | 182.4606595 | 3.189728687 | -5.838007911 | 0 | dna binding protein |
| EY692431 | S44296399 | 7434 | 797 | 6991.81723 | 847.4045877 | -3.044544628 | 0 | cs00-c2-003-071-e03- sweet orange greenhouse plant citrus sinensis mrna |
| EY693300 | S44296932 | 122 | 33 | 114.7433013 | 35.08701555 | -1.709400787 | 0 | protein |
| EY697211 | S44297931 | 15 | 1 | 14.10778295 | 1.063242896 | -3.729948165 | 0 | cs00-c3-700-057-e11- sweet orange development stadium (1 of 6) citrus sinensis mrna |
| EY697254 | S44297974 | 80 | 11 | 75.24150906 | 11.69567185 | -2.685554045 | 0 | gcn5-related n-acetyltransferase brct |
| EY698173 | S44298123 | 3005 | 1947 | 2826.259184 | 2070.133918 | -0.449169676 | 0 | ethylene-responsive transcription factor erf003 |
| EY703404 | S44299028 | 16 | 70 | 15.04830181 | 74.42700269 | 2.306225448 | 0 | protein |
| EY708591 | S44299763 | 683 | 1875 | 642.3743836 | 1993.580429 | 1.633875543 | 0 | chaperone protein dnaj chloroplastic� |
| EY708603 | S44299775 | 49 | 4 | 46.0854243 | 4.252971582 | -3.437767413 | 0 | protein |
| EY708892 | S44299841 | 25 | 7 | 23.51297158 | 7.442700269 | -1.659558837 | 0 | predicted protein [Populus trichocarpa] |
| EY710274 | S44300229 | 329 | 51 | 309.430706 | 54.22538767 | -2.512576001 | 0 | expansin-a5� |
| EY711363 | S44300548 | 2 | 13 | 1.881037727 | 13.82215764 | 2.877382149 | 0 | cs00-c3-702-006-h03- sweet orange development stadium (3 of 6) citrus sinensis mrna |
| EY712586 | S44300973 | 9 | 48 | 8.46466977 | 51.03565898 | 2.59197993 | 0 | 70 kda peptidyl-prolyl isomerase ame: full=peptidyl-prolyl cis-trans isomerase� |
| EY714913 | S44302068 | 27 | 3 | 25.39400931 | 3.189728687 | -2.992982571 | 0 | PREDICTED: hypothetical protein [Vitis vinifera] |
| EY721825 | S44304176 | 124 | 320 | 116.624339 | 340.2377266 | 1.544674215 | 0 | u-box domain-containing protein 33 ame: full=plant u-box protein 33 includes: ame: full=e3 ubiquitin ligase includes: ame: full=serine threonine-protein kinase |
| EY725657 | S44305228 | 390 | 116 | 366.8023567 | 123.3361759 | -1.572406888 | 0 | calcium-dependent protein |
| EY726474 | S44305390 | 13 | 24 | 12.22674522 | 25.51782949 | 1.061465213 | 0 | probable lrr receptor-like serine threonine-protein kinase at3g47570 flags: precursor |
| EY727849 | S44306107 | 102 | 245 | 95.93292406 | 260.4945094 | 1.441155028 | 0 | cs00-c3-703-106-d10- sweet orange development stadium (4 of 6) citrus sinensis mrna |
| EY729980 | S44306572 | 23 | 57 | 21.63193386 | 60.60484504 | 1.486270489 | 0 | protein |
| EY735211 | S44307777 | 1 | 5 | 0.940518863 | 5.316214478 | 2.498870526 | 0 | cs00-c3-704-110-a08- sweet orange development stadium (5 of 6) citrus sinensis mrna |
| EY735307 | S44307873 | 9 | 1 | 8.46466977 | 1.063242896 | -2.992982571 | 0 | cs00-c3-704-093-f03- sweet orange development stadium (5 of 6) citrus sinensis mrna |
| EY735395 | S44307961 | 1 | 14 | 0.940518863 | 14.88540054 | 3.984297353 | 0 | bordetella bronchiseptica strain complete genome segment 11 16 |
| EY743321 | S44310013 | 117 | 31 | 110.040707 | 32.96052976 | -1.739225978 | 0 | cs00-c3-705-093-a11- sweet orange development stadium (6 of 6) citrus sinensis mrna |
| EY743805 | S44310497 | 1069 | 303 | 1005.414665 | 322.1625973 | -1.641929723 | 0 | expansin-a10� |
| EY744797 | S44310593 | 126 | 28 | 118.5053768 | 29.77080107 | -1.992982571 | 0 | thaumatin-like protein flags: precursor |
| EY746532 | S44311115 | 106 | 360 | 99.69499951 | 382.7674424 | 1.940875073 | 0 | probable calcium-binding protein cml41 ame: full=calmodulin-like protein 41 |
| EY747114 | S44311361 | 43 | 11 | 40.44231112 | 11.69567185 | -1.789890705 | 0 | serine carboxypeptidase-like 17 flags: precursor |
| EY747177 | S44311424 | 48 | 8 | 45.14490544 | 8.505943164 | -2.40802007 | 0 | vacuolar protein sorting-associated protein 2 homolog 2� |
| EY747810 | S44311623 | 2187 | 132 | 2056.914754 | 140.3480622 | -3.873400955 | 0 | acidic endochitinase win6 flags: precursor |
| EY748285 | S44311762 | 4 | 11 | 3.762075453 | 11.69567185 | 1.636374049 | 0 | gdsl esterase lipase exl3 ame: full=family ii extracellular lipase 3� |
| EY748412 | S44311889 | 12 | 60 | 11.28622636 | 63.79457373 | 2.498870526 | 0 | cyclic nucleotide-gated ion channel 1� |
| EY750528 | S44312871 | 5 | 14 | 4.702594316 | 14.88540054 | 1.662369258 | 0 | cs00-c5-003-096-c02- sweet orange greenhouse plant citrus sinensis mrna |
| EY750529 | S44312872 | 6 | 19 | 5.64311318 | 20.20161501 | 1.839907444 | 0 | �pectate lyase 19 flags: precursor |
| EY751674 | S44313029 | 6 | 1 | 5.64311318 | 1.063242896 | -2.40802007 | 0 | stamen-specific protein fil1 flags: precursor |
| EY753911 | S44313494 | 235 | 73 | 221.0219329 | 77.61673137 | -1.509749957 | 0 | cs12-c1-001-008-d02- sweet orange field plant a citrus sinensis mrna |
| EY754023 | S44313606 | 969 | 291 | 911.3627785 | 309.4036826 | -1.558535082 | 0 | conserved hypothetical protein [Ricinus communis] |
| EY754467 | S44313728 | 7 | 17 | 6.583632043 | 18.07512922 | 1.45705035 | 0 | cs12-c1-001-014-h08- sweet orange field plant a citrus sinensis mrna |
| EY755171 | S44314068 | 8 | 34 | 7.524150906 | 36.15025845 | 2.264405272 | 0 | probable phosphatase phospho2 |
| EY755611 | S44314396 | 539 | 150 | 506.9396673 | 159.4864343 | -1.668380341 | 0 | agglutinin ame: full=cca |
| EY755890 | S44314563 | 45 | 14 | 42.32334885 | 14.88540054 | -1.507555743 | 0 | ac007519\_26 ests gb |
| EY756179 | S44314740 | 28 | 7 | 26.33452817 | 7.442700269 | -1.823057569 | 0 | cs13-c1-001-005-e10- sweet orange field plant b citrus sinensis mrna |
| EY757129 | S44315018 | 13 | 3 | 12.22674522 | 3.189728687 | -1.938534787 | 0 | cs13-c1-001-017-g03- sweet orange field plant b citrus sinensis mrna |
| EY757917 | S44315372 | 15 | 4 | 14.10778295 | 4.252971582 | -1.729948165 | 0 | glucan endo- -beta-glucosidase 1 ame: full=(1- |
| FE659277 | S46102866 | 7 | 4 | 6.583632043 | 4.252971582 | -0.630412491 | 0 | probable 1-acyl-sn-glycerol-3-phosphate acyltransferase 5 ame: full=lysophosphatidyl acyltransferase 5 |
| FE659288 | S46102877 | 419 | 103 | 394.0774037 | 109.5140182 | -1.847363476 | 0 | 645 hong anliu sweet orange ssh library citrus sinensis cdna 5 mrna |
| EU861194 | S46915372 | 1459 | 276 | 1372.217022 | 293.4550392 | -2.22529728 | 0 | glucose-1-phosphate adenylyltransferase large subunit 1 ame: full=alpha-d-glucose-1-phosphate adenyl transferase ame: full=adp-glucose pyrophosphorylase ame: full=agpase s ame: full=adp-glucose synthase |
| DC899996 | S47736293 | 39 | 70 | 36.68023567 | 74.42700269 | 1.020823229 | 0 | gdsl esterase lipase apg ame: full=extracellular lipase apg flags: precursor |
| DC900000 | S47736297 | 17 | 47 | 15.98882068 | 49.97241609 | 1.644068441 | 0 | cucumisin ame: allergen=cuc m 1 flags: precursor |
| FC871130 | S49954784 | 12 | 1 | 11.28622636 | 1.063242896 | -3.40802007 | 0 | conserved hypothetical protein [Ricinus communis] |
| FC921914 | S49955742 | 40 | 212 | 37.62075453 | 225.4074938 | 2.582934791 | 0 | bahd acyltransferase at5g47980 |
| FC922338 | S49956166 | 294 | 896 | 276.5125458 | 952.6656344 | 1.784625008 | 0 | disease resistance response protein 206 |
| FC922518 | S49956346 | 14 | 35 | 13.16726409 | 37.21350134 | 1.498870526 | 0 | protein |
| FC922537 | S49956365 | 1 | 47 | 0.940518863 | 49.97241609 | 5.731531283 | 0 | lotus japonicus genomic chromosome clone: complete sequence |
|  |  |  |  |  |  |  |  |  |
