# Supplementary material for: Transcriptome Profiling of Citrus Fruit Response to Huanglongbing Disease
Source: PLoS One. 2012 May 31;7(5):e38039. doi: 10.1371/journal.pone.0038039 (PMC3364978; doi:10.1371/journal.pone.0038039)
Supplement: Table S6 — Differentially expressed genes in symptomatic fruit in comparison to apparently healthy, annotations and number of protein-protein interactions deduced from Arabidopsis knowledgebase. (HTM) [file pone.0038039.s006.htm]

Table�S6


# Table�S6

| Table S6. Differentially expressed genes in symptomatic fruit in comparison to apparently healthy, annotations and number of protein-protein interactions deduced from Arabidopsis knowledgebase. Those that also appear in Table S7 are hightlighted in color. | | | | | | | | | |
|  |  |  |  |  |  |  |  |  |  |
| GB id | id2 | count AH | count SY | norm AH | norm SY | log2foldchange | PPI | annotation |  |
| CX302713 | S24635816 | 4 | 23 | 4.322484351 | 21.28406 | 2.299840474 | 78 | 40s ribosomal protein s5-1 |  |
| EY718661 | S44256224 | 174 | 806 | 188.0280693 | 745.8674 | 1.987971051 | 50 | coatomer subunit beta -1 ame: full=beta -coat protein 1� |  |
| CX303205 | S24636242 | 0 | 14 | 0 | 12.95551 | Inf | 44 | heat shock 70 kda protein |  |
| CX048901 | S22590595 | 3 | 14 | 3.241863264 | 12.95551 | 1.99867094 | 38 | dna-directed rna polymerase subunit beta ame: full=pep ame: full=plastid-encoded rna polymerase subunit beta� |  |
| EY662694 | S44287810 | 897 | 316 | 969.3171158 | 292.4244 | -1.728904909 | 31 | �kda proline-rich protein |  |
| CK933773 | S22537734 | 16 | 3 | 17.28993741 | 2.776181 | -2.638758981 | 29 | chromatin assembly factor subunit |  |
| EY697005 | S44240315 | 1 | 3 | 1.080621088 | 2.776181 | 1.361241019 | 27 | rac-like gtp-binding protein rac2 flags: precursor |  |
| EY707908 | S44299542 | 61 | 271 | 65.91788636 | 250.7817 | 1.927690222 | 26 | thioredoxin-like 4 |  |
| CF653559 | S22533293 | 27 | 114 | 29.17676937 | 105.4949 | 1.85428103 | 24 | pathogenesis-related protein 1� |  |
| EY757748 | S44283689 | 492 | 1568 | 531.6655752 | 1451.017 | 1.448473857 | 23 | ctp synthase 2 ame: full=utp--ammonia ligase 2 ame: full=ctp synthetase 2 |  |
| CX046244 | S22589334 | 743 | 2614 | 802.9014683 | 2418.979 | 1.591103543 | 22 | ribose-phosphate pyrophosphokinase 2 ame: full=phosphoribosyl pyrophosphate synthetase 2 ame: full=prs ii |  |
| DC900352 | S47736649 | 188 | 52 | 203.1567645 | 48.12047 | -2.077870615 | 20 | chloroplast-targeted copper |  |
| EY713241 | S44301292 | 258 | 1211 | 278.8002407 | 1120.652 | 2.007034412 | 19 | abc transporter c family member 3� |  |
| EU586327 | S45783088 | 252 | 993 | 272.3165141 | 918.916 | 1.754648502 | 17 | sugar carrier protein c |  |
| EY657709 | S44212743 | 190 | 837 | 205.3180067 | 774.5546 | 1.915506722 | 15 | glycine cleavage system h mitochondrial flags: precursor |  |
| EY716350 | S44302721 | 135 | 615 | 145.8838469 | 569.1172 | 1.963905521 | 15 | glyceraldehyde-3-phosphate dehydrogenase chloroplastic ame: full=nadp-dependent glyceraldehydephosphate dehydrogenase subunit a flags: precursor |  |
| EY740246 | S44272490 | 499 | 1723 | 539.2299228 | 1594.453 | 1.564089499 | 14 | peptide transporter ptr2 ame: full=histidine-transporting protein |  |
| EY756199 | S44314760 | 20 | 3 | 21.61242176 | 2.776181 | -2.960687076 | 14 | arabidopsis thaliana pola2 (dna polymerase alpha 2) dna binding dna-directed dna polymerase complete cds |  |
| EY684115 | S44294181 | 434 | 3151 | 468.9895521 | 2915.916 | 2.636321325 | 12 | peroxisomal -2-hydroxy-acid oxidase ame: full=glycolate oxidase� |  |
| CV886236 | S22583855 | 59 | 215 | 63.75664418 | 198.9597 | 1.641828318 | 10 | developmental protein sepallata 1 ame: full=agamous-like mads-box protein agl2 |  |
| CV886643 | S22584043 | 280 | 93 | 302.5739046 | 86.06162 | -1.813845688 | 10 | homoserine kinase� |  |
| CX675973 | S23017894 | 10 | 3 | 10.80621088 | 2.776181 | -1.960687076 | 10 | probable histone ame: full=hta7 |  |
| EY652580 | S44208972 | 81 | 284 | 87.53030812 | 262.8118 | 1.586175635 | 10 | gtp cyclohydrolase 1 1 ame: full=gtp cyclohydrolase i 1� |  |
| EY658551 | S44213347 | 351 | 1327 | 379.2980018 | 1227.998 | 1.694903953 | 10 | serine mitochondrial� |  |
| EY673229 | S44223139 | 194 | 1149 | 209.640491 | 1063.277 | 2.342528759 | 10 | �homolog subfamily b member 13 ame: full=testis spermatocyte apoptosis-related gene 6 protein ame: full=testis and spermatogenesis cell-related protein 6 ame: full=testis spermatogenesis apoptosis-related gene 6 protein ame: full=testis spermatogenesis apoptosis-related gene 3 protein |  |
| EH668995 | S36660248 | 19 | 77 | 20.53180067 | 71.25532 | 1.795137545 | 9 | saxifraga stolonifera inverted repeat partial sequence chloroplast |  |
| CF838294 | S22572832 | 6 | 31 | 6.483726527 | 28.68721 | 2.145512328 | 7 | ocs element-binding factor 1� |  |
| CX050469 | S22595000 | 33 | 10 | 35.6604959 | 9.253937 | -1.946187506 | 7 | agamous-like mads-box protein agl11 |  |
| DY305900 | S34125462 | 885 | 3194 | 956.3496627 | 2955.708 | 1.627893471 | 7 | glucan endo- -beta-glucosidase 13 ame: full=(1- |  |
| DY305942 | S34125504 | 638 | 2711 | 689.436254 | 2508.742 | 1.863475302 | 7 | glucan endo- -beta-glucosidase 14 ame: full=(1- |  |
| EY708596 | S44299768 | 32 | 13 | 34.57987481 | 12.03012 | -1.523281764 | 7 | cyclin-u4-1� |  |
| EY683854 | S44231104 | 34 | 578 | 36.74111699 | 534.8776 | 3.863741359 | 6 | fructose-bisphosphate cytoplasmic isozyme 1 |  |
| EY743284 | S44274520 | 1684 | 679 | 1819.765912 | 628.3424 | -1.534130141 | 6 | mitochondrial carrier |  |
| EY677252 | S44226182 | 240 | 94 | 259.3490611 | 86.98701 | -1.576023226 | 5 | �phosphate dikinase regulatory protein chloroplastic ame: full= pi dikinase regulatory protein 1� |  |
| CK933617 | S22537584 | 3 | 10 | 3.241863264 | 9.253937 | 1.513244112 | 4 | dna helicase sim |  |
| EY728100 | S44263114 | 82 | 29 | 88.6109292 | 26.83642 | -1.723292491 | 4 | 125 kda kinesin-related protein |  |
| CX048165 | S22593763 | 4 | 18 | 4.322484351 | 16.65709 | 1.94620352 | 3 | serine threonine-protein kinase ctr1 |  |
| CX053639 | S22596692 | 6 | 24 | 6.483726527 | 22.20945 | 1.776278518 | 3 | random slug protein 5 ame: full=cral-trio domain-containing protein 5 |  |
| DR908226 | S26279103 | 716 | 74 | 773.7246989 | 68.47914 | -3.498083893 | 3 | thioredoxin h-type 2� |  |
| DR910057 | S26280934 | 16 | 6 | 17.28993741 | 5.552362 | -1.638758981 | 3 | phosphoinositide phospholipase c 6 ame: full=phosphoinositide phospholipase plc6� |  |
| EY706290 | S44248032 | 193 | 2256 | 208.55987 | 2087.688 | 3.323372833 | 3 | probable lrr receptor-like serine threonine-protein kinase at1g56130 flags: precursor |  |
| EY711989 | S44252316 | 13 | 2 | 14.04807414 | 1.850787 | -2.9241612 | 3 | lrr receptor-like serine threonine-protein kinase gso1 ame: full=protein gassho 1 flags: precursor |  |
| EY744046 | S44274722 | 1906 | 6968 | 2059.663793 | 6448.144 | 1.646475023 | 3 | glyceraldehyde-3-phosphate dehydrogenase chloroplastic ame: full=nadp-dependent glyceraldehydephosphate dehydrogenase subunit a flags: precursor |  |
| EY686806 | S44294722 | 23 | 116 | 24.85428502 | 107.3457 | 2.110697557 | 3 | hcs2 (holocarboxylase synthetase 2) biotin- |  |
| EY692767 | S44296511 | 2 | 11 | 2.161242176 | 10.17933 | 2.235710137 | 3 | beta-hexosaminidase subunit b2 ame: full=n-acetyl-beta-glucosaminidase subunit b2 ame: full=beta-n-acetylhexosaminidase subunit b2 flags: precursor |  |
| CV714332 | S22576139 | 6 | 115 | 6.483726527 | 106.4203 | 4.036806068 | 2 | dctp pyrophosphatase 1 ame: full=deoxycytidine-triphosphatase 1� |  |
| CV886565 | S22584007 | 494 | 132 | 533.8268174 | 122.152 | -2.127694594 | 2 | acyl-protein thioesterase 2� |  |
| CX071523 | S22602384 | 42 | 4 | 45.38608569 | 3.701575 | -3.616038905 | 2 | probable auxin efflux carrier component 1c ame: full= 1c |  |
| CX671540 | S23020242 | 11 | 75 | 11.88683197 | 69.40453 | 2.54566559 | 2 | primary amine oxidase ame: full=amine oxidase flags: precursor |  |
| DN620167 | S24240472 | 115 | 489 | 124.2714251 | 452.5175 | 1.864479122 | 2 | arogenate dehydrogenase chloroplastic ame: full= 2 flags: precursor |  |
| CX300811 | S24634749 | 0 | 1 | 0 | 0.925394 | Inf | 2 | uncharacterized oxidoreductase yhdf |  |
| DY305576 | S34125138 | 933 | 5244 | 1008.219475 | 4852.765 | 2.266997218 | 2 | 12-oxophytodienoate reductase 2 ame: full=12-oxophytodienoate- -reductase 2� |  |
| DY305806 | S34125368 | 1312 | 273 | 1417.774867 | 252.6325 | -2.488516346 | 2 | probable sarcosine oxidase |  |
| DY306123 | S34125685 | 1666 | 6110 | 1800.314732 | 5654.156 | 1.651062498 | 2 | probable wrky transcription factor 40 ame: full=wrky dna-binding protein 40 |  |
| EY678067 | S44226885 | 73 | 375 | 78.88533941 | 347.0227 | 2.137200745 | 2 | asparagine synthetase ame: full=glutamine-dependent asparagine synthetase |  |
| EY690546 | S44235732 | 10 | 4 | 10.80621088 | 3.701575 | -1.545649577 | 2 | thymidylate synthase-like |  |
| EY709790 | S44250887 | 2910 | 1104 | 3144.607366 | 1021.635 | -1.622000463 | 2 | protein in2-1 homolog b ame: full=glutathione s-transferase gstz5 |  |
| EY722375 | S44259274 | 221 | 18 | 238.8172604 | 16.65709 | -3.84169904 | 2 | s-adenosylmethionine decarboxylase proenzyme� |  |
| EY744544 | S44275220 | 69 | 6 | 74.56285506 | 5.552362 | -3.747283438 | 2 | populus trichocarpa histidine kinase osmosensor mrna |  |
| EY746794 | S44276709 | 22 | 4 | 23.77366393 | 3.701575 | -2.6831531 | 2 | cyclin-d2-1 ame: full=g1 s-specific cyclin-d2-1� |  |
| EY757319 | S44283372 | 118 | 513 | 127.5132884 | 474.727 | 1.896450484 | 2 | regulatory protein npr1 ame: full=nonexpresser of pr genes 1 ame: full=non-inducible immunity protein 1� |  |
| EY658218 | S44286218 | 2544 | 8898 | 2749.100047 | 8234.154 | 1.582660946 | 2 | 5 -adenylylsulfate reductase chloroplastic ame: full=adenosine 5 -phosphosulfate 5 -adenylylsulfate sulfotransferase 1� |  |
| CN184192 | S22554452 | 6 | 11 | 6.483726527 | 10.17933 | 0.650747636 | 1 | atp binding |  |
| CV712407 | S22579576 | 28 | 115 | 30.25739046 | 106.4203 | 1.814413647 | 1 | nad h-quinone oxidoreductase subunit chloroplastic ame: full=nad h dehydrogenase subunit h ame: full=nadh-plastoquinone oxidoreductase subunit h ame: full=nadh-plastoquinone oxidoreductase 49 kda subunit |  |
| CV887297 | S22585971 | 4 | 15 | 4.322484351 | 13.88091 | 1.683169114 | 1 | patellin-6 |  |
| CX046632 | S22589541 | 48 | 254 | 51.86981222 | 235.05 | 2.180000704 | 1 | beta- insoluble isoenzyme cwinv3 ame: full=cell wall invertase 3� |  |
| CX048001 | S22590174 | 9 | 38 | 9.725589791 | 35.16496 | 1.85428103 | 1 | nad h-quinone oxidoreductase subunit chloroplastic ame: full=nad h dehydrogenase subunit i� |  |
| CX051115 | S22595347 | 99 | 554 | 106.9814877 | 512.6681 | 2.260664064 | 1 | f-box protein at1g67340 |  |
| CX052350 | S22596008 | 3 | 9 | 3.241863264 | 8.328544 | 1.361241019 | 1 | cysteine proteinase 15a ame: full=turgor-responsive protein 15a flags: precursor |  |
| CX052367 | S22596020 | 253 | 82 | 273.3971352 | 75.88229 | -1.849163052 | 1 | two-component response regulator arr3 |  |
| CX078571 | S22606132 | 90 | 25 | 97.25589791 | 23.13484 | -2.071718388 | 1 | peptidyl-trna hydrolase mitochondrial� |  |
| AJ000081 | S22606215 | 406 | 1412 | 438.7321617 | 1306.656 | 1.574466974 | 1 | glucan endo- -beta- basic isoform ame: full=(1- |  |
| AB276107 | S35152778 | 2200 | 10076 | 2377.366393 | 9324.267 | 1.971626117 | 1 | acid beta-fructofuranosidase ame: full=acid sucrose hydrolase ame: full=acid invertase� |  |
| EU340032 | S43896462 | 154 | 549 | 166.4156475 | 508.0412 | 1.610154317 | 1 | phospholipase d gamma 1� |  |
| EY655569 | S44211275 | 1 | 33 | 1.080621088 | 30.53799 | 4.820672638 | 1 | 3-ketoacyl- synthase 19� |  |
| EY657719 | S44212753 | 28 | 198 | 30.25739046 | 183.228 | 2.598280216 | 1 | serine threonine-protein kinase srk2f ame: full=ost1-kinase-like 5 ame: full=snf1-related kinase� |  |
| EY659645 | S44214119 | 376 | 1309 | 406.313529 | 1211.34 | 1.575939048 | 1 | endo beta n- |  |
| EY671382 | S44222188 | 202 | 1047 | 218.2854597 | 968.8873 | 2.150112762 | 1 | fructose- - chloroplastic� |  |
| EY674382 | S44223858 | 604 | 2449 | 652.6951371 | 2266.289 | 1.795850837 | 1 | photosystem i reaction center subunit chloroplastic ame: full=psi-g flags: precursor |  |
| EY676236 | S44225502 | 18 | 83 | 19.45117958 | 76.80768 | 1.981392948 | 1 | cysteine-rich receptor-like protein kinase 8� |  |
| EY678177 | S44226995 | 41 | 530 | 44.3054646 | 490.4587 | 3.468575063 | 1 | glutamine chloroplastic ame: full=gs2 ame: full=glutamate--ammonia ligase flags: precursor |  |
| EY689158 | S44234666 | 131 | 439 | 141.5613625 | 406.2479 | 1.520932646 | 1 | photosystem i p700 chlorophyll a apoprotein a2 ame: full= ame: full=psi-b |  |
| EY702054 | S44244580 | 88 | 918 | 95.09465573 | 849.5115 | 3.159197243 | 1 | tropinone reductase homolog at1g07440 |  |
| EY705175 | S44247141 | 10 | 76 | 10.80621088 | 70.32992 | 2.702277937 | 1 | tetrapyrrole-binding chloroplastic ame: full=genomes uncoupled 4 flags: precursor |  |
| EY710496 | S44251369 | 13 | 48 | 14.04807414 | 44.4189 | 1.660801301 | 1 | nudix hydrolase mitochondrial� |  |
| EY710292 | S44300247 | 6069 | 26749 | 6558.289382 | 24753.36 | 1.91623275 | 1 | ribulose bisphosphate carboxylase small chloroplastic� |  |
| EY719197 | S44303656 | 22 | 85 | 23.77366393 | 78.65847 | 1.726237836 | 1 | probable 6-phosphogluconolactonase 1� |  |
| EY727780 | S44306038 | 934 | 280 | 1009.300096 | 259.1102 | -1.961717205 | 1 | lipoxygenase chloroplastic� |  |
| BQ625029 | S22532848 | 125 | 428 | 135.077636 | 396.0685 | 1.55196122 | 0 | uncharacterized protein at1g14870 |  |
| CF653219 | S22533093 | 0 | 31 | 0 | 28.68721 | Inf | 0 | citrus sinensis complete genome |  |
| CK665498 | S22534213 | 4 | 23 | 4.322484351 | 21.28406 | 2.299840474 | 0 | protein |  |
| CK932724 | S22536939 | 47 | 176 | 50.78919113 | 162.8693 | 1.681121285 | 0 | ORF493 [Pinus koraiensis] |  |
| CK933484 | S22537451 | 15 | 4 | 16.20931632 | 3.701575 | -2.130612077 | 0 | xyloglucan endotransglucosylase hydrolase protein 9� |  |
| CK933909 | S22538125 | 32 | 228 | 34.57987481 | 210.9898 | 2.609168532 | 0 | �gem-like protein 8 |  |
| CK934441 | S22538619 | 15 | 56 | 16.20931632 | 51.82205 | 1.676742845 | 0 | cgf1004259\_a05 developing fruit peel at 38 dafb citrus sinensis cdna clone p38dab10004\_iif\_a05 5 mrna |  |
| CK935156 | S22539338 | 2 | 8 | 2.161242176 | 7.40315 | 1.776278518 | 0 | cgf1004177\_c06 developing fruit peel at 38 dafb citrus sinensis cdna clone p38da0001\_iif\_c06 5 mrna |  |
| CK935574 | S22539458 | 12 | 47 | 12.96745305 | 43.49351 | 1.745904869 | 0 | protein |  |
| CK935206 | S22539526 | 3 | 11 | 3.241863264 | 10.17933 | 1.650747636 | 0 | chloroplast envelope membrane 70 kda heat shock-related protein |  |
| CK935341 | S22539631 | 486 | 1366 | 525.1818487 | 1264.088 | 1.267207783 | 0 | unknown [Populus trichocarpa] |  |
| CK935355 | S22539635 | 165 | 40 | 178.3024795 | 37.01575 | -2.268115601 | 0 | cgf1004562\_f08 developing fruit 24 dafb citrus sinensis cdna clone t24dab0001\_if\_f08 5 mrna |  |
| CK935394 | S22539659 | 33 | 138 | 35.6604959 | 127.7043 | 1.840408856 | 0 | cgf1004562\_c10 developing fruit 24 dafb citrus sinensis cdna clone t24dab0001\_if\_c10 5 mrna |  |
| CK935886 | S22539998 | 674 | 2591 | 728.3386132 | 2397.695 | 1.718967037 | 0 | protein |  |
| CK936435 | S22540547 | 27 | 10 | 29.17676937 | 9.253937 | -1.656680889 | 0 | cgf1004382\_d07 developing fruit 24 dafb citrus sinensis cdna clone t24dab0001\_ivf\_d07 5 mrna |  |
| CK936468 | S22540575 | 12 | 75 | 12.96745305 | 69.40453 | 2.420134708 | 0 | probable mitochondrial chaperone bcs1 ame: full=bcs1-like protein |  |
| CK935321 | S22540610 | 24 | 20 | 25.93490611 | 18.50787 | -0.486755888 | 0 | dc900926 yjs citrus unshiu cdna clone yjs0893 5 mrna |  |
| CK936515 | S22540833 | 6 | 25 | 6.483726527 | 23.13484 | 1.835172207 | 0 | cgf1004521\_e12 developing fruit flavedo at 80 dafb citrus sinensis cdna clone f80dab0001\_ivf\_e12 5 mrna |  |
| CK936548 | S22540860 | 2 | 11 | 2.161242176 | 10.17933 | 2.235710137 | 0 | hat family dimerisation |  |
| CK936724 | S22540992 | 16 | 77 | 17.28993741 | 71.25532 | 2.043065059 | 0 | photosystem ii protein l |  |
| CK937027 | S22541128 | 10 | 3 | 10.80621088 | 2.776181 | -1.960687076 | 0 | predicted protein [Populus trichocarpa] |  |
| CK937174 | S22541286 | 518 | 3584 | 559.7617235 | 3316.611 | 2.566825153 | 0 | calmodulin-like protein 9� |  |
| CK937755 | S22541875 | 7 | 2 | 7.564347615 | 1.850787 | -2.031076404 | 0 | cgf1004482\_d07 developing fruit albedo at 80 dafb in p x2 vector citrus sinensis cdna clone a80dab0001\_ivf\_d07 5 mrna |  |
| CK938229 | S22542521 | 3 | 57 | 3.241863264 | 52.74744 | 4.024206032 | 0 | cgf1004476\_e07 developing fruit albedo at 80 dafb in p x2 vector citrus sinensis cdna clone a80dab0002\_iif\_e07 5 mrna |  |
| CK938844 | S22542591 | 68 | 26 | 73.48223397 | 24.06024 | -1.610744605 | 0 | cgf1004437\_d11 developing fruit albedo at 165 dafb citrus sinensis cdna clone a1650002\_iif\_d11 5 mrna |  |
| CK938890 | S22542633 | 316 | 80 | 341.4762638 | 74.0315 | -2.205574135 | 0 | protease inhibitor seed storage lipid transfer protein family protein |  |
| CK939008 | S22542771 | 43 | 11 | 46.46670678 | 10.17933 | -2.190554618 | 0 | cgf1004435\_e09 developing fruit albedo at 165 dafb citrus sinensis cdna clone a1650003\_ivf\_e09 5 mrna |  |
| CK938415 | S22542876 | 29 | 7 | 31.33801155 | 6.477756 | -2.274347555 | 0 | cgf1004443\_b08 developing fruit albedo at 165 dafb citrus sinensis cdna clone a1650001\_ivf\_b08 5 mrna |  |
| CK939516 | S22543639 | 1092 | 323 | 1180.038228 | 298.9022 | -1.981088268 | 0 | snakin-1 flags: precursor |  |
| CK939541 | S22543662 | 701 | 2326 | 757.5153826 | 2152.466 | 1.506643266 | 0 | ethylene-responsive transcription factor 1a� |  |
| CK939658 | S22543787 | 4 | 17 | 4.322484351 | 15.73169 | 1.863741359 | 0 | cgf1004747\_d06 developing fruit flavedo at 165 dafb citrus sinensis cdna clone f1650002\_iiif\_d06 5 mrna |  |
| CK939844 | S22543970 | 69 | 29 | 74.56285506 | 26.83642 | -1.474264943 | 0 | cgf1004744\_h08 developing fruit flavedo at 165 dafb citrus sinensis cdna clone f1650003\_ivf\_h08 5 mrna |  |
| CK940054 | S22544183 | 6 | 30 | 6.483726527 | 27.76181 | 2.098206613 | 0 | ankyrin repeat-containing |  |
| CN185598 | S22544557 | 76 | 628 | 82.12720268 | 581.1473 | 2.822971754 | 0 | ethylene-responsive transcription factor 1b� |  |
| CN186662 | S22545043 | 133 | 787 | 143.7226047 | 728.2849 | 2.341215908 | 0 | indole-3-acetic acid-amido synthetase ame: full=auxin-responsive gh3-like protein 4� |  |
| CN188214 | S22545831 | 61 | 26 | 65.91788636 | 24.06024 | -1.454019101 | 0 | udp- |  |
| CN185600 | S22546315 | 58 | 17 | 62.67602309 | 15.73169 | -1.994239636 | 0 | ucrcs05\_0001j12\_f washington navel orange stored fruit pulp cdna library citrus sinensis cdna clone mrna |  |
| CN185964 | S22546468 | 16 | 7 | 17.28993741 | 6.477756 | -1.41636656 | 0 | vitis vinifera contig whole genome shotgun sequence |  |
| CN186015 | S22546485 | 28 | 7 | 30.25739046 | 6.477756 | -2.223721482 | 0 | protein |  |
| CN188179 | S22547436 | 173 | 589 | 186.9474482 | 545.0569 | 1.543774114 | 0 | unknown protein [Oryza sativa Japonica Group] |  |
| CN186421 | S22547782 | 43 | 12 | 46.46670678 | 11.10472 | -2.065023736 | 0 | predicted protein [Populus trichocarpa] |  |
| CN190352 | S22548549 | 22 | 7 | 23.77366393 | 6.477756 | -1.875798178 | 0 | ucrcs06\_0003f06\_r washington navel orange stored fruit rind cdna library citrus sinensis cdna clone mrna |  |
| CN191394 | S22549085 | 620 | 5034 | 669.9850745 | 4658.432 | 2.797643614 | 0 | protein |  |
| CN189156 | S22549853 | 27 | 221 | 29.17676937 | 204.512 | 2.809293575 | 0 | myb-like protein j |  |
| CN192158 | S22551120 | 23 | 98 | 24.85428502 | 90.68859 | 1.867426406 | 0 | solanum lycopersicum clone: htc in fruit |  |
| CN183186 | S22552207 | 2 | 11 | 2.161242176 | 10.17933 | 2.235710137 | 0 | predicted protein [Populus trichocarpa] |  |
| CN185151 | S22553288 | 56 | 1451 | 60.51478092 | 1342.746 | 4.4717554 | 0 | probable sulfate transporter |  |
| CN185193 | S22553311 | 24 | 41 | 25.93490611 | 37.94114 | 0.548868022 | 0 | ucrcs04\_0006p03\_f ruby orange developing flower cdna library citrus sinensis cdna clone mrna |  |
| CN182947 | S22553931 | 64 | 272 | 69.15974962 | 251.7071 | 1.863741359 | 0 | �glutamine amidotransferase-like protein yvde homolog |  |
| CB250355 | S22554981 | 187 | 798 | 202.0761434 | 738.4642 | 1.869628995 | 0 | acidic endochitinase flags: precursor |  |
| CB292505 | S22556310 | 653 | 3280 | 705.6455704 | 3035.291 | 2.104819436 | 0 | f-box family protein |  |
| CB293991 | S22557136 | 573 | 1938 | 619.1958833 | 1793.413 | 1.534240045 | 0 | upf0497 membrane protein 4 |  |
| CB293236 | S22558423 | 82 | 337 | 88.6109292 | 311.8577 | 1.815331295 | 0 | nac domain-containing protein 71� |  |
| CB304738 | S22559035 | 18 | 2 | 19.45117958 | 1.850787 | -3.393646483 | 0 | flavedo0002\_i \_b08 flavedo mature citrus sinensis cdna clone flavedo0002\_i \_b08 3 mrna |  |
| CB305067 | S22559334 | 6 | 18 | 6.483726527 | 16.65709 | 1.361241019 | 0 | hypothetical protein [Vitis vinifera] |  |
| CB610486 | S22559972 | 18 | 6 | 19.45117958 | 5.552362 | -1.808683983 | 0 | populus trichocarpa mrna |  |
| CB610913 | S22560246 | 102 | 23 | 110.223351 | 21.28406 | -2.372584868 | 0 | albedo0002\_ii \_h06 mature albedo citrus sinensis cdna clone albedo0002\_ii \_h06 5 mrna |  |
| CB611167 | S22560410 | 1 | 10 | 1.080621088 | 9.253937 | 3.098206613 | 0 | �kda class i heat shock protein ame: full= kda heat shock protein 1� |  |
| CB611027 | S22560606 | 2989 | 5148 | 3229.976432 | 4763.927 | 0.560627675 | 0 | albedo0001\_ \_c05 mature albedo citrus sinensis cdna clone albedo0001\_ \_c05 5 mrna |  |
| CF417791 | S22561156 | 31 | 5 | 33.49925372 | 4.626969 | -2.855989697 | 0 | cytochrome p450 716b1 ame: full=cytochrome p450 cypa1 |  |
| CF504270 | S22562119 | 64 | 332 | 69.15974962 | 307.2307 | 2.15131795 | 0 | nac domain-containing protein 90� |  |
| CF509240 | S22567089 | 3 | 10 | 3.241863264 | 9.253937 | 1.513244112 | 0 | uridine cytidine kinase |  |
| CF833196 | S22568730 | 10 | 35 | 10.80621088 | 32.38878 | 1.58363344 | 0 | ucrcs02\_03d03\_r ruby orange ovary at anthesis cdna library citrus sinensis cdna clone mrna |  |
| CF833645 | S22568937 | 6 | 19 | 6.483726527 | 17.58248 | 1.439243531 | 0 | protein |  |
| CF832109 | S22569827 | 114 | 62 | 123.190804 | 57.37441 | -1.102415186 | 0 | ucrcs02\_01f08\_r ruby orange ovary at anthesis cdna library citrus sinensis cdna clone mrna |  |
| CF832725 | S22570124 | 6 | 2 | 6.483726527 | 1.850787 | -1.808683983 | 0 | cyclin-d3-1 ame: full=g1 s-specific cyclin-d3-1� |  |
| CF834253 | S22570867 | 49 | 10 | 52.9504333 | 9.253937 | -2.516503231 | 0 | hat dimerisation domain-containing protein |  |
| CF835215 | S22571322 | 2 | 22 | 2.161242176 | 20.35866 | 3.235710137 | 0 | cytochrome p450 71d9 ame: full=p450 cp3 |  |
| CF836416 | S22571911 | 680 | 2335 | 734.8223397 | 2160.794 | 1.556094417 | 0 | ucrcs03\_02o21\_f washington navel orange shoot meristem cdna library citrus sinensis cdna clone mrna |  |
| CF838109 | S22572738 | 6 | 16 | 6.483726527 | 14.8063 | 1.191316017 | 0 | ucrcs03\_05m03\_r washington navel orange shoot meristem cdna library citrus sinensis cdna clone mrna |  |
| CF838363 | S22572866 | 8 | 44 | 8.644968703 | 40.71732 | 2.235710137 | 0 | ucrcs03\_06f18\_r washington navel orange shoot meristem cdna library citrus sinensis cdna clone mrna |  |
| CF838764 | S22573058 | 28 | 568 | 30.25739046 | 525.6236 | 4.118670716 | 0 | �beta-d-xylosidase ame: full= z152 |  |
| CF838806 | S22573079 | 91 | 413 | 98.33651899 | 382.1876 | 1.958481849 | 0 | cytochrome p450 94a1 ame: full=p450-dependent fatty acid omega-hydroxylase |  |
| CF838891 | S22573122 | 11 | 43 | 11.88683197 | 39.79193 | 1.743111654 | 0 | pentatricopeptide repeat-containing protein at1g31920 |  |
| CF836213 | S22573575 | 9545 | 15037 | 10314.52828 | 13915.15 | 0.431978182 | 0 | ucrcs03\_02j17\_f washington navel orange shoot meristem cdna library citrus sinensis cdna clone mrna |  |
| CF838214 | S22574550 | 16 | 2 | 17.28993741 | 1.850787 | -3.223721482 | 0 | disease resistance response protein 206 |  |
| CF838393 | S22574639 | 65 | 450 | 70.24037071 | 416.4272 | 2.567691896 | 0 | endochitinase a� |  |
| CF838525 | S22574705 | 206 | 866 | 222.6079441 | 801.391 | 1.848001206 | 0 | protein |  |
| CV713409 | S22575615 | 4 | 22 | 4.322484351 | 20.35866 | 2.235710137 | 0 | AF506028\_16hypothetical protein [Poncirus trifoliata] |  |
| CV714710 | S22576355 | 337 | 1355 | 364.1693066 | 1253.909 | 1.783750873 | 0 | protein |  |
| CV715341 | S22576716 | 30 | 165 | 32.41863264 | 152.69 | 2.235710137 | 0 | triacylglycerol lipase like protein |  |
| CV715912 | S22577041 | 79 | 125 | 85.36906594 | 115.6742 | 0.438282055 | 0 | ucrcs08\_0006i04\_r parent washington navel orange callus cdna library ucrcs08-1 citrus sinensis cdna clone mrna |  |
| CV716150 | S22577176 | 2 | 13 | 2.161242176 | 12.03012 | 2.476718236 | 0 | ucrcs08\_0006o05\_r parent washington navel orange callus cdna library ucrcs08-1 citrus sinensis cdna clone mrna |  |
| CV718209 | S22578347 | 268 | 1530 | 289.6064515 | 1415.852 | 2.289505265 | 0 | phosphatidylglycerol specific phospholipase c |  |
| CV719359 | S22579005 | 89 | 410 | 96.17527682 | 379.4114 | 1.980025187 | 0 | cysteine-rich receptor-like protein kinase 10� |  |
| CV719514 | S22579092 | 24 | 146 | 25.93490611 | 135.1075 | 2.381140576 | 0 | retrotransposon gag protein |  |
| CV719572 | S22579126 | 16 | 58 | 17.28993741 | 53.67284 | 1.634259513 | 0 | cysteine-rich receptor-like protein kinase 18� |  |
| CV713065 | S22579858 | 753 | 3069 | 813.7076791 | 2840.033 | 1.803325394 | 0 | taxadien-5-alpha-ol o-acetyltransferase ame: full=taxa-4 -dien-5alpha-ol-o-acetyltransferase� |  |
| CV715155 | S22580755 | 121 | 13 | 130.7551516 | 12.03012 | -3.442145001 | 0 | early nodulin-93� |  |
| CV715612 | S22580948 | 5 | 4 | 5.403105439 | 3.701575 | -0.545649577 | 0 | ucrcs08\_0006a07\_f parent washington navel orange callus cdna library ucrcs08-1 citrus sinensis cdna clone mrna |  |
| CV718596 | S22582220 | 26 | 3 | 28.09614828 | 2.776181 | -3.339198699 | 0 | protein |  |
| CV718780 | S22582298 | 2 | 21 | 2.161242176 | 19.43327 | 3.168595941 | 0 | plz12\_luppoprotein pplz12 |  |
| CV719351 | S22582541 | 249 | 574 | 269.0746509 | 531.176 | 0.981183513 | 0 | citrus maxima clone m29-31b2-1 ctvv resistance partial sequence |  |
| CV885187 | S22583349 | 4 | 19 | 4.322484351 | 17.58248 | 2.024206032 | 0 | ucrcs04\_2\_013b09\_t3 ruby orange developing flower cdna library ucrcs04-ucr citrus sinensis cdna clone mrna |  |
| CV885563 | S22583523 | 322 | 1440 | 347.9599903 | 1332.567 | 1.937214736 | 0 | protein notum homolog flags: precursor |  |
| CV886175 | S22583829 | 17 | 85 | 18.37055849 | 78.65847 | 2.098206613 | 0 | linalool chloroplastic flags: precursor |  |
| CV886253 | S22583865 | 57 | 248 | 61.59540201 | 229.4976 | 1.897584814 | 0 | linalool chloroplastic flags: precursor |  |
| CV885307 | S22584971 | 4 | 17 | 4.322484351 | 15.73169 | 1.863741359 | 0 | vitis vinifera contig whole genome shotgun sequence |  |
| CV887374 | S22585048 | 2 | 29 | 2.161242176 | 26.83642 | 3.634259513 | 0 | ucrcs04\_2\_030h07\_t3 ruby orange developing flower cdna library ucrcs04-ucr citrus sinensis cdna clone mrna |  |
| CV998067 | S22586071 | 1546 | 5763 | 1670.640202 | 5333.044 | 1.674558219 | 0 | isoflavone 2 -hydroxylase ame: full=cytochrome p450 81e1 ame: full=p450 91a4 ame: full=cyp ge-3 |  |
| CX043477 | S22586216 | 33 | 335 | 35.6604959 | 310.0069 | 3.119901684 | 0 | laccase-8 ame: full=benzenediol:oxygen oxidoreductase 8 ame: full=urishiol oxidase 8 ame: full=diphenol oxidase 8 flags: precursor |  |
| CX045546 | S22587179 | 615 | 2784 | 664.581969 | 2576.296 | 1.954779414 | 0 | brassinosteroid lrr receptor kinase ame: full=tbri1 ame: full=altered brassinolide sensitivity 1 ame: full=systemin receptor sr160 flags: precursor |  |
| CX046157 | S22587473 | 40 | 147 | 43.22484351 | 136.0329 | 1.654022768 | 0 | calcium ion binding |  |
| CX046378 | S22587578 | 72 | 347 | 77.80471832 | 321.1116 | 2.045145369 | 0 | uncharacterized plant-specific domain tigr01589 family expressed |  |
| CX043666 | S22587969 | 131 | 557 | 141.5613625 | 515.4443 | 1.864389034 | 0 | probable glutathione s-transferase ame: full=pathogenesis-related protein 1 |  |
| CX044433 | S22588384 | 0 | 2 | 0 | 1.850787 | Inf | 0 | PREDICTED: hypothetical protein [Vitis vinifera] |  |
| CX044628 | S22588487 | 4 | 30 | 4.322484351 | 27.76181 | 2.683169114 | 0 | 3 -n-debenzoyl-2 -deoxytaxol n-benzoyltransferase� |  |
| CX045330 | S22588860 | 29 | 1894 | 31.33801155 | 1752.696 | 5.805518139 | 0 | ucrcs07\_22b08\_b parent washington navel orange thrip-challenged flavedo cdna library ucrcs07 citrus sinensis cdna clone ucrcs07-22b08-d16-1- mrna |  |
| CX045334 | S22588865 | 30 | 128 | 32.41863264 | 118.4504 | 1.869387923 | 0 | jasmonate o-methyltransferase ame: full=s-adenosyl-l-methionine:jasmonic acid carboxyl methyltransferase ame: full=floral nectary-specific protein 1 |  |
| CX046087 | S22589258 | 243 | 305 | 262.5909243 | 282.2451 | 0.104131447 | 0 | alcohol acyl transferase |  |
| CX046605 | S22589528 | 85 | 309 | 91.85279247 | 285.9467 | 1.63835061 | 0 | uncharacterized plant-specific domain tigr01615 family expressed |  |
| CX046670 | S22589560 | 16 | 49 | 17.28993741 | 45.34429 | 1.390988362 | 0 | oxygen-evolving enhancer protein 3- chloroplastic� |  |
| CX046913 | S22589674 | 56 | 11 | 60.51478092 | 10.17933 | -2.571644785 | 0 | lea34\_goshilate embryogenesis abundant protein d-34 (lea d-34) |  |
| CX047235 | S22589821 | 7 | 58 | 7.564347615 | 53.67284 | 2.826904591 | 0 | er lumen protein retaining receptor ame: full=hdel receptor ame: full=pgp169-12 |  |
| CX047303 | S22589854 | 12 | 2 | 12.96745305 | 1.850787 | -2.808683983 | 0 | ucrcs09\_13d02\_b ruby orange developing seed cdna library ucrcs09 citrus sinensis cdna clone ucrcs09-13d02-g4-1- mrna |  |
| CX047858 | S22590108 | 12 | 2 | 12.96745305 | 1.850787 | -2.808683983 | 0 | hypothetical protein Cagg\_1305 [Chloroflexus aggregans DSM 9485] |  |
| CX048288 | S22590309 | 2 | 9 | 2.161242176 | 8.328544 | 1.94620352 | 0 | seed biotin-containing protein sbp65 ame: full=seed biotinylated protein of 65 kda ame: full=bp75 |  |
| CX048448 | S22590387 | 4 | 39 | 4.322484351 | 36.09036 | 3.061680737 | 0 | salt tolerance protein |  |
| CX049214 | S22590740 | 28 | 11 | 30.25739046 | 10.17933 | -1.571644785 | 0 | conserved hypothetical protein [Ricinus communis] |  |
| CX051823 | S22591947 | 2 | 13 | 2.161242176 | 12.03012 | 2.476718236 | 0 | ucrcs09\_42c03\_b ruby orange developing seed cdna library ucrcs09 citrus sinensis cdna clone ucrcs09-42c03-f5-1- mrna |  |
| CX052345 | S22592189 | 7 | 43 | 7.564347615 | 39.79193 | 2.395188351 | 0 | PREDICTED: hypothetical protein [Vitis vinifera] |  |
| CX054039 | S22592993 | 0 | 70 | 0 | 64.77756 | Inf | 0 | dna binding protein |  |
| CX046929 | S22593100 | 0 | 18 | 0 | 16.65709 | Inf | 0 | 30s ribosomal protein chloroplastic |  |
| CX047037 | S22593159 | 1 | 4 | 1.080621088 | 3.701575 | 1.776278518 | 0 | protein |  |
| CX048087 | S22593721 | 13 | 46 | 14.04807414 | 42.56811 | 1.599400756 | 0 | leucine-rich repeat receptor protein kinase exs ame: full=extra sporogenous cells protein ame: full=protein excess microsporocytes 1 flags: precursor |  |
| CX048272 | S22593823 | 27 | 9 | 29.17676937 | 8.328544 | -1.808683983 | 0 | nicotiana tabacum mitochondrial complete genome |  |
| CX049428 | S22594442 | 27 | 272 | 29.17676937 | 251.7071 | 3.108853857 | 0 | ucrcs09\_27e07\_b ruby orange developing seed cdna library ucrcs09 citrus sinensis cdna clone ucrcs09-27e07-j14-1- mrna |  |
| CX049508 | S22594486 | 48 | 4 | 51.86981222 | 3.701575 | -3.808683983 | 0 | ucrcs09\_28a02\_b ruby orange developing seed cdna library ucrcs09 citrus sinensis cdna clone ucrcs09-28a02-a3-1- mrna |  |
| CX049594 | S22594531 | 5 | 17 | 5.403105439 | 15.73169 | 1.541813265 | 0 | ucrcs09\_28e03\_b ruby orange developing seed cdna library ucrcs09 citrus sinensis cdna clone ucrcs09-28e03-i5-1- mrna |  |
| CX049911 | S22594703 | 16 | 0 | 17.28993741 | 0 | -Inf | 0 | hypoxia induced protein conserved region containing expressed |  |
| CX051193 | S22595387 | 16 | 98 | 17.28993741 | 90.68859 | 2.390988362 | 0 | glycogenin-1 |  |
| CX051392 | S22595492 | 39 | 220 | 42.14422243 | 203.5866 | 2.272236013 | 0 | peroxisomal membrane protein 11b ame: full=peroxin-11b� |  |
| CX069388 | S22596922 | 3 | 12 | 3.241863264 | 11.10472 | 1.776278518 | 0 | vitis vinifera contig whole genome shotgun sequence |  |
| CX070778 | S22597563 | 44 | 17 | 47.54732786 | 15.73169 | -1.595690259 | 0 | ucrcs08\_18h07\_b parent washington navel orange callus cdna library ucrcs08-2 citrus sinensis cdna clone ucrcs08-18h07-o14-1- mrna |  |
| CX071402 | S22597854 | 10 | 70 | 10.80621088 | 64.77756 | 2.58363344 | 0 | populus trichocarpa mrna |  |
| CX073115 | S22598646 | 186 | 172 | 200.9955223 | 159.1677 | -0.336615538 | 0 | ribosome-recycling factor� |  |
| CX073495 | S22598823 | 2 | 23 | 2.161242176 | 21.28406 | 3.299840474 | 0 | ucrcs08\_33d09\_b parent washington navel orange callus cdna library ucrcs08-2 citrus sinensis cdna clone ucrcs08-33d09-g17-1- mrna |  |
| CX074337 | S22599210 | 9 | 60 | 9.725589791 | 55.52362 | 2.513244112 | 0 | probable gibberellin receptor gid1l2 ame: full=gid1-like protein 2 |  |
| CX075508 | S22599753 | 407 | 142 | 439.8127828 | 131.4059 | -1.742859347 | 0 | ethylene-responsive transcription factor erf012 |  |
| CX077288 | S22600576 | 42 | 302 | 45.38608569 | 279.4689 | 2.622365835 | 0 | bap2 (bon association protein 2) |  |
| CX078107 | S22600959 | 8 | 30 | 8.644968703 | 27.76181 | 1.683169114 | 0 | gag-pol precursor |  |
| CX070380 | S22601769 | 19 | 59 | 20.53180067 | 54.59823 | 1.410994054 | 0 | nadh-plastoquinone oxidoreductase subunit 1 |  |
| CX071017 | S22602114 | 1 | 13 | 1.080621088 | 12.03012 | 3.476718236 | 0 | protein abscisic acid-insensitive 5 ame: full=dc3 promoter-binding factor 1� |  |
| CX072399 | S22602857 | 13 | 4 | 14.04807414 | 3.701575 | -1.9241612 | 0 | luvunga tt 364 ribosomal protein s16 intron chloroplast |  |
| CX074538 | S22604004 | 82 | 22 | 88.6109292 | 20.35866 | -2.121841868 | 0 | mlo-like protein 11� |  |
| CX074829 | S22604160 | 26 | 6 | 28.09614828 | 5.552362 | -2.339198699 | 0 | afadin- and alpha-actinin-binding |  |
| CX075279 | S22604401 | 1020 | 1021 | 1102.23351 | 944.827 | -0.222307768 | 0 | cyclin-b1-2 ame: full=g2 mitotic-specific cyclin-b1-2� |  |
| CX075413 | S22604474 | 1 | 18 | 1.080621088 | 16.65709 | 3.94620352 | 0 | ucrcs08\_44e07\_g parent washington navel orange callus cdna library ucrcs08-2 citrus sinensis cdna clone ucrcs08-44e07-j14-1- mrna |  |
| CX076036 | S22604809 | 94 | 387 | 101.5783823 | 358.1274 | 1.817879423 | 0 | dehydration-responsive element-binding protein 1c� |  |
| CX077854 | S22605785 | 21 | 6 | 22.69304284 | 5.552362 | -2.031076404 | 0 | ucrcs08\_5d09\_g parent washington navel orange callus cdna library ucrcs08-2 citrus sinensis cdna clone ucrcs08-5d09-g17-1- mrna |  |
| CX078533 | S22606113 | 227 | 850 | 245.3009869 | 786.5847 | 1.681049062 | 0 | retrovirus-related pol polyprotein from transposon 412 includes: ame: full=protease includes: ame: full=reverse transcriptase includes: ame: full=endonuclease |  |
| AY242385 | S22606177 | 1961 | 10340 | 2119.097953 | 9568.571 | 2.174853263 | 0 | allene oxide chloroplastic ame: full=cytochrome p450 74a ame: full=hydroperoxide dehydrase flags: precursor |  |
| AY029198 | S22606183 | 209 | 797 | 225.8498074 | 737.5388 | 1.7073553 | 0 | beta-galactosidase ame: full=acid beta-galactosidase� |  |
| AF255013 | S22606190 | 11 | 67 | 11.88683197 | 62.00138 | 2.38293609 | 0 | trans-cinnamate 4-monooxygenase ame: full=cinnamic acid 4-hydroxylase� |  |
| AF321533 | S22606192 | 2929 | 11070 | 3165.139166 | 10244.11 | 1.694453642 | 0 | 1-aminocyclopropane-1-carboxylate oxidase� |  |
| CX675005 | S23016617 | 12 | 45 | 12.96745305 | 41.64272 | 1.683169114 | 0 | ucrcs08\_59a10\_b parent washington navel orange callus cdna library ucrcs08-3 citrus sinensis cdna clone ucrcs08-59a10-a19-1- mrna |  |
| CX675318 | S23016762 | 0 | 28 | 0 | 25.91102 | Inf | 0 | PREDICTED: hypothetical protein [Vitis vinifera] |  |
| CX675379 | S23016788 | 44 | 230 | 47.54732786 | 212.8406 | 2.16233695 | 0 | hypothetical protein [Vitis vinifera] |  |
| CX675444 | S23016818 | 12 | 4 | 12.96745305 | 3.701575 | -1.808683983 | 0 | structural constituent of ribosome |  |
| CX676159 | S23017148 | 1 | 0 | 1.080621088 | 0 | -Inf | 0 | transcription factor bhlh96 ame: full=transcription factor en 15 ame: full=bhlh transcription factor bhlh096 ame: full=basic helix-loop-helix protein 96� |  |
| CX675374 | S23017571 | 9 | 27 | 9.725589791 | 24.98563 | 1.361241019 | 0 | citrus unshiu 5 flanking region of d-limonene synthase |  |
| CX671301 | S23018234 | 38 | 173 | 41.06360134 | 160.0931 | 1.962979232 | 0 | probable lrr receptor-like serine threonine-protein kinase at4g08850 flags: precursor |  |
| CX674613 | S23019889 | 1 | 30 | 1.080621088 | 27.76181 | 4.683169114 | 0 | protein |  |
| CX674852 | S23020020 | 150 | 710 | 162.0931632 | 657.0296 | 2.019135042 | 0 | 1-aminocyclopropane-1-carboxylate oxidase 1� |  |
| CX671538 | S23020240 | 156 | 570 | 168.5768897 | 527.4744 | 1.645694408 | 0 | serine threonine-protein kinase |  |
| CX672401 | S23020684 | 22 | 93 | 23.77366393 | 86.06162 | 1.856005711 | 0 | 2og-fe oxygenase family protein |  |
| CX673163 | S23020996 | 2 | 15 | 2.161242176 | 13.88091 | 2.683169114 | 0 | tyrosine-sulfated glycopeptide receptor 1 |  |
| CX673271 | S23021048 | 57 | 804 | 61.59540201 | 744.0166 | 3.594440195 | 0 | probable lrr receptor-like serine threonine-protein kinase at1g56140 flags: precursor |  |
| CX674272 | S23021730 | 37 | 127 | 39.98298025 | 117.525 | 1.555509839 | 0 | ucrcs10\_5d08\_b madame vinous sweet orange multiple pathogen-infected cdna library ucrcs10 citrus sinensis cdna clone ucrcs10-5d08-g15- mrna |  |
| CX674294 | S23021738 | 9 | 0 | 9.725589791 | 0 | -Inf | 0 | serine threonine-protein kinase bri1-like 2 ame: full=brassinosteroid insensitive 1-like protein 2 ame: full=protein vascular highway 1 flags: precursor |  |
| CX674658 | S23021906 | 7 | 13 | 7.564347615 | 12.03012 | 0.669363314 | 0 | ucrcs10\_7h06\_b madame vinous sweet orange multiple pathogen-infected cdna library ucrcs10 citrus sinensis cdna clone ucrcs10-7h06-p11- mrna |  |
| DN134777 | S23749922 | 30 | 9 | 32.41863264 | 8.328544 | -1.960687076 | 0 | harpin-induced protein |  |
| DN617353 | S24238864 | 503 | 200 | 543.5524072 | 185.0787 | -1.554279882 | 0 | ucrcs11\_01b13\_f parent washington navel orange scale-infested rind cdna library ucrcs11 citrus sinensis cdna clone mrna |  |
| DN618152 | S24239321 | 162 | 433 | 175.0606162 | 400.6955 | 1.19465173 | 0 | anthranilate n-benzoyltransferase |  |
| DN620202 | S24240495 | 477 | 89 | 515.4562589 | 82.36004 | -2.645833507 | 0 | populus trichocarpa mrna |  |
| DN618681 | S24241922 | 62 | 22 | 66.99850745 | 20.35866 | -1.718486174 | 0 | chloroplast protein import component |  |
| CX300836 | S24634758 | 2 | 12 | 2.161242176 | 11.10472 | 2.361241019 | 0 | c08002g06sk ootsw1 citrus sinensis cdna clone mrna |  |
| CX301073 | S24634830 | 16 | 5 | 17.28993741 | 4.626969 | -1.901793387 | 0 | n-acetyltransferase 13 ame: full=n-acetyltransferase 5� |  |
| CX302584 | S24635461 | 75 | 29 | 81.04658159 | 26.83642 | -1.594559177 | 0 | conserved hypothetical protein [Ricinus communis] |  |
| CX302874 | S24635669 | 5 | 18 | 5.403105439 | 16.65709 | 1.624275425 | 0 | c08025b06sk ootsw1 citrus sinensis cdna clone mrna |  |
| CX302610 | S24635769 | 4 | 12 | 4.322484351 | 11.10472 | 1.361241019 | 0 | basic 7s globulin ame: full=sbg7s� |  |
| CX303214 | S24636058 | 0 | 3 | 0 | 2.776181 | Inf | 0 | c08028g09sk ootsw1 citrus sinensis cdna clone mrna |  |
| CX303326 | S24636299 | 426 | 1858 | 460.3445834 | 1719.382 | 1.901103684 | 0 | protein tify 10a ame: full=jasmonate zim domain-containing protein 1 |  |
| CX303356 | S24636313 | 8 | 24 | 8.644968703 | 22.20945 | 1.361241019 | 0 | ubiquitin |  |
| DR403529 | S25678527 | 20 | 111 | 21.61242176 | 102.7187 | 2.24876629 | 0 | arabidopsis thaliana at3g62990 complete cds |  |
| DR403848 | S25678846 | 41 | 9 | 44.3054646 | 8.328544 | -2.411348485 | 0 | csah-pnp1246h10 developing fruit peel at 38 dafb citrus sinensis cdna clone csah-pnp1246h10 5 mrna |  |
| DR404453 | S25679451 | 32 | 229 | 34.57987481 | 211.9152 | 2.615482306 | 0 | lrr receptor-like serine threonine-protein kinase gso1 ame: full=protein gassho 1 flags: precursor |  |
| DR404508 | S25679506 | 64 | 20 | 69.15974962 | 18.50787 | -1.901793387 | 0 | protein |  |
| DR405064 | S25680062 | 3712 | 2567 | 4011.265478 | 2375.486 | -0.755834896 | 0 | csab-pnp1240e07 developing fruit albedo at 165 dafb citrus sinensis cdna clone csab-pnp1240e07 5 mrna |  |
| DR405378 | S25680376 | 1 | 2 | 1.080621088 | 1.850787 | 0.776278518 | 0 | csad-pnp1242e09 developing fruit flavedo at 165 dafb citrus sinensis cdna clone csad-pnp1242e09 5 mrna |  |
| DR405410 | S25680408 | 1 | 4 | 1.080621088 | 3.701575 | 1.776278518 | 0 | csad-pnp1242f24 developing fruit flavedo at 165 dafb citrus sinensis cdna clone csad-pnp1242f24 5 mrna |  |
| DR405840 | S25680838 | 43 | 17 | 46.46670678 | 15.73169 | -1.562523395 | 0 | protein |  |
| DR908284 | S26279161 | 41 | 8 | 44.3054646 | 7.40315 | -2.581273486 | 0 | transcription factor bhlh68 ame: full=transcription factor en 60 ame: full=bhlh transcription factor bhlh068 ame: full=basic helix-loop-helix protein 68� |  |
| DR909652 | S26280529 | 10 | 33 | 10.80621088 | 30.53799 | 1.498744543 | 0 | usda-fp\_17780 citrus sinensis phloem citrus sinensis cdna clone vpe-14\_c04 5 mrna |  |
| DR909768 | S26280645 | 507 | 2746 | 547.8748915 | 2541.131 | 2.213552491 | 0 | rotenone-insensitive nadh-ubiquinone mitochondrial |  |
| DR910089 | S26280966 | 4 | 17 | 4.322484351 | 15.73169 | 1.863741359 | 0 | usda-fp\_18217 citrus sinensis phloem citrus sinensis cdna clone vpe-21\_f01 5 mrna |  |
| DR910337 | S26281214 | 2 | 18 | 2.161242176 | 16.65709 | 2.94620352 | 0 | usda-fp\_18465 citrus sinensis phloem citrus sinensis cdna clone vpe-39\_f06 5 mrna |  |
| DR910614 | S26281491 | 75 | 20 | 81.04658159 | 18.50787 | -2.130612077 | 0 | cytochrome p450 716b2 ame: full=cytochrome p450 cypa2 |  |
| DR910861 | S26281738 | 24 | 1 | 25.93490611 | 0.925394 | -4.808683983 | 0 | usda-fp\_18989 citrus sinensis phloem citrus sinensis cdna clone vpe-14\_f02 5 mrna |  |
| DR911102 | S26281979 | 16 | 4 | 17.28993741 | 3.701575 | -2.223721482 | 0 | usda-fp\_19230 citrus sinensis phloem citrus sinensis cdna clone vpe-45\_d05 5 mrna |  |
| DR911129 | S26282006 | 6 | 0 | 6.483726527 | 0 | -Inf | 0 | populus trichocarpa mrna |  |
| DR911419 | S26282296 | 1 | 6 | 1.080621088 | 5.552362 | 2.361241019 | 0 | populus trichocarpa mrna |  |
| DR911742 | S26282619 | 5 | 28 | 5.403105439 | 25.91102 | 2.261705345 | 0 | usda-fp\_19870 citrus sinensis phloem citrus sinensis cdna clone vpe-10\_g10 5 mrna |  |
| DR911822 | S26282699 | 34 | 11 | 36.74111699 | 10.17933 | -1.851752704 | 0 | usda-fp\_19950 citrus sinensis phloem citrus sinensis cdna clone vpe-14\_h08 5 mrna |  |
| DR911940 | S26282817 | 21 | 3 | 22.69304284 | 2.776181 | -3.031076404 | 0 | usda-fp\_20068 citrus sinensis phloem citrus sinensis cdna clone vpe-19\_g07 5 mrna |  |
| DR911951 | S26282828 | 30 | 11 | 32.41863264 | 10.17933 | -1.671180459 | 0 | usda-fp\_20079 citrus sinensis phloem citrus sinensis cdna clone vpe-17\_d07 5 mrna |  |
| DR912040 | S26282917 | 2 | 5 | 2.161242176 | 4.626969 | 1.098206613 | 0 | usda-fp\_20168 citrus sinensis phloem citrus sinensis cdna clone vpe-30\_e08 5 mrna |  |
| DT214571 | S26468960 | 71 | 163 | 76.72409724 | 150.8392 | 0.975259553 | 0 | upf0016 membrane protein sll0615 |  |
| DT214578 | S26468967 | 4 | 12 | 4.322484351 | 11.10472 | 1.361241019 | 0 | ucrcs11\_01a07\_f2 parent washington navel orange red scale-infested rind cdna library ucrcs11-2 citrus sinensis cdna clone mrna |  |
| DQ028471 | S32321255 | 343 | 1600 | 370.6530331 | 1480.63 | 1.998069942 | 0 | 9-cis-epoxycarotenoid dioxygenase chloroplastic ame: full= 1 flags: precursor |  |
| DY257153 | S34124431 | 602 | 1330 | 650.5338949 | 1230.774 | 0.919869372 | 0 | homo sapiens chromosome 21 segment hs21c085 |  |
| DY257345 | S34124623 | 120 | 567 | 129.6745305 | 524.6983 | 2.016592848 | 0 | upf0301 protein plut\_0637 |  |
| DY257400 | S34124678 | 23678 | 9257 | 25586.94612 | 8566.37 | -1.578652081 | 0 | citrus unshiu mrna for metallothionein-like complete cds |  |
| DY257442 | S34124720 | 5006 | 1968 | 5409.589166 | 1821.175 | -1.570649552 | 0 | dna binding protein |  |
| DY257479 | S34124757 | 130 | 462 | 140.4807414 | 427.5319 | 1.605659747 | 0 | kn0aak2da08fm1 ruit citrus sinensis cdna 5 mrna |  |
| DY257559 | S34124837 | 5 | 18 | 5.403105439 | 16.65709 | 1.624275425 | 0 | protein |  |
| DY257584 | S34124862 | 7 | 21 | 7.564347615 | 19.43327 | 1.361241019 | 0 | �mo25-like protein at5g47540 |  |
| DY257639 | S34124917 | 20 | 5 | 21.61242176 | 4.626969 | -2.223721482 | 0 | kn0aak3cf02fm1 ruit citrus sinensis cdna 5 mrna |  |
| DY259698 | S34124999 | 5 | 19 | 5.403105439 | 17.58248 | 1.702277937 | 0 | predicted protein [Populus trichocarpa] |  |
| DY305480 | S34125042 | 93 | 517 | 100.4977612 | 478.4286 | 2.251140177 | 0 | probable lrr receptor-like serine threonine-protein kinase at2g16250 flags: precursor |  |
| DY305488 | S34125050 | 77 | 402 | 83.20782376 | 372.0083 | 2.160543669 | 0 | transcription factor bhlh35 ame: full=transcription factor en 41 ame: full=bhlh transcription factor bhlh035 ame: full=basic helix-loop-helix protein 35� |  |
| DY305530 | S34125092 | 35 | 854 | 37.82173807 | 790.2863 | 4.385087761 | 0 | chlorophyllase- chloroplastic ame: full=chlorophyll-chlorophyllido hydrolase 1� |  |
| DY305531 | S34125093 | 10605 | 37688 | 11459.98664 | 34876.24 | 1.605639134 | 0 | ribulose bisphosphate carboxylase oxygenase activase chloroplastic� |  |
| DY305550 | S34125112 | 2569 | 11785 | 2776.115575 | 10905.77 | 1.973951481 | 0 | polyol transporter 5 ame: full=sugar-proton symporter plt5 ame: full=protein polyol transporter 5� |  |
| DY305565 | S34125127 | 155 | 33 | 167.4962686 | 30.53799 | -2.455451768 | 0 | probable lrr receptor-like serine threonine-protein kinase at2g23950 flags: precursor |  |
| DY305569 | S34125131 | 214 | 938 | 231.2529128 | 868.0193 | 1.908255644 | 0 | protein tify 9 ame: full=protein jasmonate-associated 1 ame: full=jasmonate zim domain-containing protein 10 |  |
| DY305611 | S34125173 | 27 | 456 | 29.17676937 | 421.9795 | 3.85428103 | 0 | phytosulfokine receptor 1� |  |
| DY305613 | S34125175 | 121 | 656 | 130.7551516 | 607.0583 | 2.214967286 | 0 | transcription factor bhlh36 ame: full=transcription factor en 6 ame: full=bhlh transcription factor bhlh036 ame: full=basic helix-loop-helix protein 36� |  |
| DY305677 | S34125239 | 49 | 294 | 52.9504333 | 272.0658 | 2.361241019 | 0 | xylem cysteine proteinase 1� |  |
| DY305690 | S34125252 | 562 | 4085 | 607.3090514 | 3780.233 | 2.637972561 | 0 | probable glutathione s-transferase ame: full=auxin-induced protein pgnt35 pcnt111 |  |
| DY305725 | S34125287 | 435 | 2454 | 470.0701732 | 2270.916 | 2.272326461 | 0 | tropinone reductase homolog at1g07440 |  |
| DY305735 | S34125297 | 937 | 383 | 1012.541959 | 354.4258 | -1.514426138 | 0 | zea mays clone mrna sequence |  |
| DY305779 | S34125341 | 25 | 87 | 27.0155272 | 80.50926 | 1.575365824 | 0 | alpha galactosidase precursor |  |
| DY305861 | S34125423 | 46 | 280 | 49.70857004 | 259.1102 | 2.381999579 | 0 | protein |  |
| DY305890 | S34125452 | 47 | 185 | 50.78919113 | 171.1978 | 1.753071127 | 0 | iaa-amino acid hydrolase ilr1-like 1 flags: precursor |  |
| DY305902 | S34125464 | 27 | 93 | 29.17676937 | 86.06162 | 1.560549827 | 0 | expansin-a4� |  |
| DY305999 | S34125561 | 10 | 36 | 10.80621088 | 33.31417 | 1.624275425 | 0 | cytochrome b6-f complex subunit 5 ame: full=cytochrome b6-f complex subunit v ame: full=cytochrome b6-f complex subunit petg |  |
| DY306014 | S34125576 | 129 | 935 | 139.4001203 | 865.2432 | 2.633873818 | 0 | probable lrr receptor-like serine threonine-protein kinase at3g47570 flags: precursor |  |
| DY306026 | S34125588 | 87 | 32 | 94.01403464 | 29.6126 | -1.666664978 | 0 | probable wound-induced protein |  |
| DY306086 | S34125648 | 105 | 468 | 113.4652142 | 433.0843 | 1.93239772 | 0 | receptor serine threonine |  |
| DY306114 | S34125676 | 206 | 950 | 222.6079441 | 879.1241 | 1.981561694 | 0 | homogentisate geranylgeranyl transferase |  |
| DY306148 | S34125710 | 13465 | 5345 | 14550.56295 | 4946.23 | -1.556673859 | 0 | caffeoyl- o-methyltransferase ame: full=trans-caffeoyl- 3-o-methyltransferase� |  |
| DY306179 | S34125741 | 2725 | 10609 | 2944.692464 | 9817.502 | 1.737239058 | 0 | protein |  |
| DY306181 | S34125743 | 1017 | 4578 | 1098.991646 | 4236.453 | 1.946676302 | 0 | protein |  |
| DY306872 | S34125767 | 22 | 94 | 23.77366393 | 86.98701 | 1.871435751 | 0 | transcription factor bhlh36 ame: full=transcription factor en 6 ame: full=bhlh transcription factor bhlh036 ame: full=basic helix-loop-helix protein 36� |  |
| AB276108 | S35152777 | 18 | 190 | 19.45117958 | 175.8248 | 3.176209125 | 0 | beta- soluble isoenzyme i ame: full=sucrose hydrolase ame: full=invertase ame: full=saccharase flags: precursor |  |
| EG358279 | S35174889 | 1186 | 5847 | 1281.61661 | 5410.777 | 2.0778711 | 0 | blue copper protein flags: precursor |  |
| EG358327 | S35174937 | 1 | 6 | 1.080621088 | 5.552362 | 2.361241019 | 0 | pectinesterase 2� |  |
| EF596736 | S38754319 | 2303 | 8407 | 2488.670365 | 7779.785 | 1.64435518 | 0 | nac domain-containing protein 2� |  |
| EY649755 | S44206917 | 51 | 183 | 55.11167548 | 169.3471 | 1.619553015 | 0 | cytochrome p450 82a3 ame: full=p450 cp6 |  |
| EY649794 | S44206956 | 10 | 0 | 10.80621088 | 0 | -Inf | 0 | beta-galactosidase 8� |  |
| EY649805 | S44206967 | 4 | 24 | 4.322484351 | 22.20945 | 2.361241019 | 0 | ethylene-responsive transcription factor shine 2 |  |
| EY649916 | S44207078 | 111 | 382 | 119.9489408 | 353.5004 | 1.55929148 | 0 | prolyl endopeptidase� |  |
| EY650015 | S44207177 | 14 | 70 | 15.12869523 | 64.77756 | 2.098206613 | 0 | anthocyanidin 3-o-glucosyltransferase 2 ame: full=flavonol 3-o-glucosyltransferase 2 ame: full=udp-glucose flavonoid 3-o-glucosyltransferase 2 |  |
| EY650412 | S44207462 | 0 | 11 | 0 | 10.17933 | Inf | 0 | cs00-c1-100-010-g02- sweet orange greenhouse plant citrus sinensis mrna |  |
| EY650597 | S44207647 | 19 | 158 | 20.53180067 | 146.2122 | 2.832131753 | 0 | cs00-c1-100-012-g05- sweet orange greenhouse plant citrus sinensis mrna |  |
| EY651594 | S44208308 | 5 | 3 | 5.403105439 | 2.776181 | -0.960687076 | 0 | eugenol synthase 1 |  |
| EY652156 | S44208660 | 2 | 10 | 2.161242176 | 9.253937 | 2.098206613 | 0 | populus trichocarpa clone pop1- complete sequence |  |
| EY652207 | S44208711 | 12 | 64 | 12.96745305 | 59.2252 | 2.191316017 | 0 | chlorophyll a-b binding protein chloroplastic ame: full=lhcii type ii cab-151� |  |
| EY652929 | S44209321 | 4 | 23 | 4.322484351 | 21.28406 | 2.299840474 | 0 | probable esterase at1g33990 |  |
| EY653001 | S44209393 | 14 | 77 | 15.12869523 | 71.25532 | 2.235710137 | 0 | �-like protein chloroplastic ame: full= -related thylakoid lumenal protein 2 ame: full=oec23-like protein 4 flags: precursor |  |
| EY653103 | S44209481 | 79 | 269 | 85.36906594 | 248.9309 | 1.543960133 | 0 | cytokinin-o-glucosyltransferase 1 ame: full=zeatin o-glucosyltransferase 1� |  |
| EY653765 | S44209765 | 3 | 16 | 3.241863264 | 14.8063 | 2.191316017 | 0 | acrosin contains: ame: full=acrosin light chain contains: ame: full=acrosin heavy chain flags: precursor |  |
| EY653900 | S44209900 | 410 | 94 | 443.054646 | 86.98701 | -2.34861273 | 0 | cs00-c1-100-049-f01- sweet orange greenhouse plant citrus sinensis mrna |  |
| EY654618 | S44210534 | 368 | 1268 | 397.6685603 | 1173.399 | 1.561055592 | 0 | chlorophyll a-b binding protein chloroplastic ame: full=lhcii type ii cab-151� |  |
| EY654875 | S44210791 | 15 | 5 | 16.20931632 | 4.626969 | -1.808683983 | 0 | probable polygalacturonase� |  |
| EY655298 | S44211004 | 200 | 723 | 216.1242176 | 669.0597 | 1.630274165 | 0 | sulfate transporter ame: full=ast12 ame: full= 1 |  |
| EY655509 | S44211215 | 102 | 517 | 110.223351 | 478.4286 | 2.117873647 | 0 | nodulin-like protein |  |
| EY655547 | S44211253 | 14 | 173 | 15.12869523 | 160.0931 | 3.403551824 | 0 | ankyrin repeat-containing protein at2g01680 |  |
| EY656241 | S44211751 | 15 | 50 | 16.20931632 | 46.26969 | 1.513244112 | 0 | protein |  |
| EY657445 | S44212591 | 192 | 732 | 207.4792489 | 677.3882 | 1.707015856 | 0 | photosystem i reaction center subunit chloroplastic ame: full=photosystem i subunit x ame: full=psi-k flags: precursor |  |
| EY657461 | S44212607 | 358 | 77 | 386.8623494 | 71.25532 | -2.440750718 | 0 | predicted protein [Populus trichocarpa] |  |
| EY657656 | S44212690 | 623 | 2073 | 673.2269377 | 1918.341 | 1.510694566 | 0 | cs00-c1-100-126-e03- sweet orange greenhouse plant citrus sinensis mrna |  |
| EY657678 | S44212712 | 538 | 2167 | 581.3741453 | 2005.328 | 1.786299594 | 0 | probable inositol transporter 1 |  |
| EY657733 | S44212767 | 264 | 104 | 285.2839672 | 96.24095 | -1.567675883 | 0 | probable polygalacturonase non-catalytic subunit jp650 ame: full=aromatic-rich glycoprotein jp650 flags: precursor |  |
| EY658058 | S44213092 | 335 | 1474 | 362.0080644 | 1364.03 | 1.913782042 | 0 | serine--glyoxylate aminotransferase ame: full=alanine--glyoxylate aminotransferase� |  |
| EY658086 | S44213120 | 6 | 25 | 6.483726527 | 23.13484 | 1.835172207 | 0 | myb family transcription factor apl� |  |
| EY658114 | S44213134 | 37 | 139 | 39.98298025 | 128.6297 | 1.685766225 | 0 | germin-like protein subfamily 3 member 1� |  |
| EY658132 | S44213152 | 1240 | 4309 | 1339.970149 | 3987.522 | 1.573291496 | 0 | ferredoxin-dependent glutamate chloroplastic ame: full=fd-gogat flags: precursor |  |
| EY658612 | S44213408 | 469 | 1578 | 506.8112902 | 1460.271 | 1.526715896 | 0 | aldehyde dehydrogenase family 2 member c4 ame: full=aldh1a ame: full=protein reduced epidermal fluorescence 1 |  |
| EY658727 | S44213523 | 61 | 25 | 65.91788636 | 23.13484 | -1.51060263 | 0 | PREDICTED: hypothetical protein [Vitis vinifera] |  |
| EY658745 | S44213541 | 238 | 1143 | 257.1878189 | 1057.725 | 2.040070443 | 0 | glycerate dehydrogenase� |  |
| EY658792 | S44213588 | 2 | 45 | 2.161242176 | 41.64272 | 4.268131615 | 0 | taxadien-5-alpha-ol o-acetyltransferase ame: full=taxa-4 -dien-5alpha-ol-o-acetyltransferase� |  |
| EY658811 | S44213607 | 30 | 8 | 32.41863264 | 7.40315 | -2.130612077 | 0 | fasciclin-like arabinogalactan protein 2 flags: precursor |  |
| EY659774 | S44214248 | 2 | 16 | 2.161242176 | 14.8063 | 2.776278518 | 0 | calcium-dependent protein kinase 2� |  |
| EY659863 | S44214337 | 23 | 194 | 24.85428502 | 179.5264 | 2.852629404 | 0 | probable lrr receptor-like serine threonine-protein kinase at3g47570 flags: precursor |  |
| EY660567 | S44214929 | 3 | 21 | 3.241863264 | 19.43327 | 2.58363344 | 0 | probable lrr receptor-like serine threonine-protein kinase at3g47570 flags: precursor |  |
| EY660669 | S44215031 | 0 | 8 | 0 | 7.40315 | Inf | 0 | predicted protein [Populus trichocarpa] |  |
| EY661071 | S44215209 | 21 | 5 | 22.69304284 | 4.626969 | -2.29411081 | 0 | cs00-c1-101-029-b02- sweet orange infected with xylella fastidiosa (stage 1 of 2) citrus sinensis mrna |  |
| EY661649 | S44215451 | 48 | 437 | 51.86981222 | 404.3971 | 2.962805487 | 0 | cytochrome p450 87a3 |  |
| EY661705 | S44215507 | 17 | 122 | 18.37055849 | 112.898 | 2.619553015 | 0 | cs00-c1-101-036-b01- sweet orange infected with xylella fastidiosa (stage 1 of 2) citrus sinensis mrna |  |
| EY661917 | S44215607 | 10 | 99 | 10.80621088 | 91.61398 | 3.083707043 | 0 | ornithine decarboxylase� |  |
| EY662029 | S44215719 | 0 | 11 | 0 | 10.17933 | Inf | 0 | cs00-c1-101-039-f06- sweet orange infected with xylella fastidiosa (stage 1 of 2) citrus sinensis mrna |  |
| EY662896 | S44216138 | 47 | 171 | 50.78919113 | 158.2423 | 1.639542181 | 0 | gcn5-related n-acetyltransferase family-like protein |  |
| EY662916 | S44216158 | 12 | 79 | 12.96745305 | 73.10611 | 2.495096766 | 0 | tir-nbs-lrr resistance protein |  |
| EY662963 | S44216205 | 22 | 8 | 23.77366393 | 7.40315 | -1.6831531 | 0 | cs00-c1-101-050-a02- sweet orange infected with xylella fastidiosa (stage 1 of 2) citrus sinensis mrna |  |
| EY663343 | S44216361 | 147 | 56 | 158.8512999 | 51.82205 | -1.616038905 | 0 | protein phloem protein 2-like a9� |  |
| EY663583 | S44216489 | 3 | 12 | 3.241863264 | 11.10472 | 1.776278518 | 0 | cs00-c1-101-056-g04- sweet orange infected with xylella fastidiosa (stage 1 of 2) citrus sinensis mrna |  |
| EY663599 | S44216505 | 0 | 1 | 0 | 0.925394 | Inf | 0 | cs00-c1-101-056-h08- sweet orange infected with xylella fastidiosa (stage 1 of 2) citrus sinensis mrna |  |
| EY664279 | S44217073 | 61 | 14 | 65.91788636 | 12.95551 | -2.347103897 | 0 | af372895\_1 at2g04793 |  |
| EY664535 | S44217329 | 10 | 90 | 10.80621088 | 83.28544 | 2.94620352 | 0 | PREDICTED: hypothetical protein [Vitis vinifera] |  |
| EY665690 | S44218260 | 10 | 3 | 10.80621088 | 2.776181 | -1.960687076 | 0 | cs00-c1-102-092-e05- sweet orange infected with xylella fastidiosa (stage 2 of 2) citrus sinensis mrna |  |
| EY665831 | S44218401 | 70 | 371 | 75.64347615 | 343.3211 | 2.182270878 | 0 | lrr receptor-like serine threonine-protein kinase gso2 ame: full=protein gassho 2 ame: full=protein embryo sac development arrest 23 flags: precursor |  |
| EY666801 | S44219049 | 52 | 384 | 56.19229657 | 355.3512 | 2.660801301 | 0 | protein |  |
| EY667118 | S44219254 | 63 | 227 | 68.07912853 | 210.0644 | 1.625547082 | 0 | nad-dependent epimerase dehydratase |  |
| EY668371 | S44219947 | 350 | 1305 | 378.2173807 | 1207.639 | 1.674901498 | 0 | ankyrin repeat-containing protein at5g02620 |  |
| EY668407 | S44219983 | 89 | 338 | 96.17527682 | 312.7831 | 1.701424524 | 0 | carbonic chloroplastic ame: full=carbonate dehydratase flags: precursor |  |
| EY671014 | S44221932 | 3 | 10 | 3.241863264 | 9.253937 | 1.513244112 | 0 | protein binding |  |
| EY671339 | S44222145 | 265 | 1545 | 286.3645883 | 1429.733 | 2.319821092 | 0 | chlorophyll a-b binding chloroplastic ame: full=lhci type ii cab flags: precursor |  |
| EY673905 | S44223479 | 12 | 76 | 12.96745305 | 70.32992 | 2.439243531 | 0 | probable cinnamyl alcohol dehydrogenase 6� |  |
| EY673920 | S44223494 | 13 | 61 | 14.04807414 | 56.44902 | 2.006576138 | 0 | cs00-c1-102-004-g03- sweet orange infected with xylella fastidiosa (stage 2 of 2) citrus sinensis mrna |  |
| EY674005 | S44223579 | 32 | 213 | 34.57987481 | 197.1089 | 2.510988138 | 0 | thylakoid membrane phosphoprotein 14 chloroplastic flags: precursor |  |
| EY674167 | S44223643 | 21 | 76 | 22.69304284 | 70.32992 | 1.631888609 | 0 | oxygen-evolving enhancer protein 3- chloroplastic� |  |
| EY674184 | S44223660 | 1728 | 499 | 1867.31324 | 461.7715 | -2.015712979 | 0 | citrus sinensis dna binding protein (v03-2) complete cds |  |
| EY674326 | S44223802 | 54 | 271 | 58.35353874 | 250.7817 | 2.103540057 | 0 | alcohol dehydrogenase ame: full=aldehyde reductase ame: full=aldo-keto reductase family 1 member a1 |  |
| EY674368 | S44223844 | 11 | 58 | 11.88683197 | 53.67284 | 2.174827895 | 0 | probable receptor-like protein kinase at2g23200 flags: precursor |  |
| EY674424 | S44223900 | 2 | 10 | 2.161242176 | 9.253937 | 2.098206613 | 0 | protein binding protein |  |
| EY674434 | S44223910 | 570 | 1883 | 615.9540201 | 1742.516 | 1.500277694 | 0 | cellulose synthase-like protein b6� |  |
| EY674556 | S44224032 | 61 | 216 | 65.91788636 | 199.885 | 1.600428683 | 0 | �phosphate chloroplastic ame: full= orthophosphate dikinase flags: precursor |  |
| EY675153 | S44224419 | 20 | 70 | 21.61242176 | 64.77756 | 1.58363344 | 0 | rhomboid protease glup ame: full=intramembrane serine protease |  |
| EY675187 | S44224453 | 55 | 225 | 59.43415983 | 208.2136 | 1.808699996 | 0 | cs00-c1-401-007-c07- sweet orange infected with citrus sinensis mrna |  |
| EY675348 | S44224614 | 5 | 17 | 5.403105439 | 15.73169 | 1.541813265 | 0 | protein |  |
| EY675558 | S44224824 | 54 | 21 | 58.35353874 | 19.43327 | -1.586291561 | 0 | probable pectinesterase pectinesterase inhibitor 54 includes: ame: full=pectinesterase inhibitor 54 ame: full=pectin methylesterase inhibitor 54 includes: ame: full=pectinesterase 54� |  |
| EY675797 | S44225063 | 56 | 211 | 60.51478092 | 195.2581 | 1.690022785 | 0 | protein |  |
| EY675861 | S44225127 | 24 | 9 | 25.93490611 | 8.328544 | -1.638758981 | 0 | cs00-c1-401-016-a01- sweet orange infected with citrus sinensis mrna |  |
| EY675862 | S44225128 | 11 | 2 | 11.88683197 | 1.850787 | -2.6831531 | 0 | lachrymatory-factor synthase flags: precursor |  |
| EY675876 | S44225142 | 70 | 1208 | 75.64347615 | 1117.876 | 3.885400241 | 0 | probable wrky transcription factor 70 ame: full=wrky dna-binding protein 70 |  |
| EY675878 | S44225144 | 46 | 141 | 49.70857004 | 130.4805 | 1.392267915 | 0 | probable receptor-like protein kinase at5g39020 flags: precursor |  |
| EY675948 | S44225214 | 10 | 67 | 10.80621088 | 62.00138 | 2.520439614 | 0 | protein |  |
| EY675957 | S44225223 | 5 | 26 | 5.403105439 | 24.06024 | 2.154790141 | 0 | cs00-c1-401-017-a07- sweet orange infected with citrus sinensis mrna |  |
| EY675992 | S44225258 | 284 | 1007 | 306.8963889 | 931.8715 | 1.602379367 | 0 | uncharacterized protein chloroplastic flags: precursor |  |
| EY676106 | S44225372 | 72 | 287 | 77.80471832 | 265.588 | 1.771260443 | 0 | predicted protein [Populus trichocarpa] |  |
| EY676463 | S44225617 | 12 | 62 | 12.96745305 | 57.37441 | 2.145512328 | 0 | protein |  |
| EY676719 | S44225873 | 17 | 509 | 18.37055849 | 471.0254 | 4.680337523 | 0 | aspartic proteinase nepenthesin-1 ame: full=nepenthesin-i flags: precursor |  |
| EY676790 | S44225944 | 223 | 772 | 240.9785026 | 714.404 | 1.567835656 | 0 | chlorophyll a-b binding protein chloroplastic ame: full=lhci type i cab-6a ame: full=light-harvesting complex i 26 kda protein flags: precursor |  |
| EY677097 | S44226027 | 9 | 24 | 9.725589791 | 22.20945 | 1.191316017 | 0 | protein |  |
| EY677189 | S44226119 | 13 | 40 | 14.04807414 | 37.01575 | 1.397766895 | 0 | protein |  |
| EY677270 | S44226200 | 95 | 17 | 102.6590033 | 15.73169 | -2.706114249 | 0 | alpha-xylosidase flags: precursor |  |
| EY677715 | S44226533 | 5 | 17 | 5.403105439 | 15.73169 | 1.541813265 | 0 | ptac14 (plastid transcriptionally active14) |  |
| EY677716 | S44226534 | 88 | 365 | 95.09465573 | 337.7687 | 1.828599553 | 0 | protein |  |
| EY677792 | S44226610 | 39 | 178 | 42.14422243 | 164.7201 | 1.96660973 | 0 | cysteine-rich receptor-like protein kinase 5� |  |
| EY677942 | S44226760 | 133 | 456 | 143.7226047 | 421.9795 | 1.553886097 | 0 | aspartic proteinase asp1� |  |
| EY678013 | S44226831 | 74 | 372 | 79.9659605 | 344.2465 | 2.105983964 | 0 | photosystem i reaction center subunit chloroplastic ame: full=photosystem i 20 kda subunit� |  |
| EY678059 | S44226877 | 34 | 140 | 36.74111699 | 129.5551 | 1.818098694 | 0 | ac007727\_10 ests gb |  |
| EY678170 | S44226988 | 343 | 1131 | 370.6530331 | 1046.62 | 1.497596966 | 0 | phosphatidylglycerol specific phospholipase c |  |
| EY678203 | S44227021 | 1969 | 7097 | 2127.742922 | 6567.519 | 1.626024713 | 0 | sigma factor sigb regulation protein rsbq |  |
| EY678443 | S44227149 | 7644 | 2196 | 8260.267595 | 2032.165 | -2.023171206 | 0 | cs00-c1-401-048-c12- sweet orange infected with citrus sinensis mrna |  |
| EY678745 | S44227451 | 4 | 13 | 4.322484351 | 12.03012 | 1.476718236 | 0 | cs00-c1-401-031-h10- sweet orange infected with citrus sinensis mrna |  |
| EY678794 | S44227500 | 125 | 438 | 135.077636 | 405.3225 | 1.585281293 | 0 | at3g61870 f21f14\_40 |  |
| EY679306 | S44227900 | 23 | 199 | 24.85428502 | 184.1534 | 2.889341183 | 0 | predicted protein [Populus trichocarpa] |  |
| EY679310 | S44227904 | 23 | 4 | 24.85428502 | 3.701575 | -2.747283438 | 0 | PREDICTED: hypothetical protein [Vitis vinifera] |  |
| EY679335 | S44227929 | 5 | 30 | 5.403105439 | 27.76181 | 2.361241019 | 0 | cycloartenol synthase� |  |
| EY679340 | S44227934 | 140 | 55 | 151.2869523 | 50.89666 | -1.571644785 | 0 | cs00-c1-401-059-b03- sweet orange infected with citrus sinensis mrna |  |
| EY679388 | S44227982 | 0 | 114 | 0 | 105.4949 | Inf | 0 | aspartic proteinase nepenthesin-1 ame: full=nepenthesin-i flags: precursor |  |
| EY679398 | S44227992 | 799 | 2951 | 863.4162492 | 2730.837 | 1.661215031 | 0 | ap2 erf and b3 domain-containing transcription repressor tem1 ame: full=rav1-like ethylene-responsive transcription factor tem1 ame: full=protein tempranillo 1 |  |
| EY679457 | S44228051 | 4 | 19 | 4.322484351 | 17.58248 | 2.024206032 | 0 | cs00-c1-401-057-d08- sweet orange infected with citrus sinensis mrna |  |
| EY679747 | S44228341 | 2 | 19 | 2.161242176 | 17.58248 | 3.024206032 | 0 | protein |  |
| EY680355 | S44228725 | 716 | 187 | 773.7246989 | 173.0486 | -2.160642799 | 0 | cs00-c1-650-010-b11- sweet orange young greenhouse plant citrus sinensis mrna |  |
| EY680705 | S44228963 | 137 | 655 | 148.045089 | 606.1329 | 2.033597532 | 0 | receptor-like protein kinase 5 ame: full=protein haesa flags: precursor |  |
| EY682492 | S44230190 | 33 | 10 | 35.6604959 | 9.253937 | -1.946187506 | 0 | �pentatricopeptide repeat-containing protein at1g19290 |  |
| EY683747 | S44230997 | 39 | 141 | 42.14422243 | 130.4805 | 1.630427652 | 0 | 2-succinylbenzoate-- ligase ame: full=osb- synthetase ame: full=o-succinylbenzoyl- synthetase |  |
| EY683784 | S44231034 | 45 | 167 | 48.62794895 | 154.5408 | 1.668129714 | 0 | cs00-c1-650-040-f04- sweet orange young greenhouse plant citrus sinensis mrna |  |
| EY683840 | S44231090 | 44 | 15 | 47.54732786 | 13.88091 | -1.776262505 | 0 | omega-6 fatty acid endoplasmic reticulum isozyme 2 |  |
| EY684493 | S44231407 | 149 | 48 | 161.0125421 | 44.4189 | -1.857927502 | 0 | populus trichocarpa mrna |  |
| EY684495 | S44231409 | 28 | 9 | 30.25739046 | 8.328544 | -1.861151402 | 0 | protein |  |
| EY684659 | S44231573 | 59 | 209 | 63.75664418 | 193.4073 | 1.600994601 | 0 | amino acid permease 6 ame: full=amino acid transporter aap6 |  |
| EY685054 | S44231968 | 43 | 241 | 46.46670678 | 223.0199 | 2.2629031 | 0 | beta-amylase ame: full= -alpha-d-glucan maltohydrolase |  |
| EY685437 | S44232351 | 5633 | 496 | 6087.138588 | 458.9953 | -3.729212928 | 0 | cs00-c2-003-019-c01- sweet orange greenhouse plant citrus sinensis mrna |  |
| EY685494 | S44232408 | 73 | 59 | 78.88533941 | 54.59823 | -0.530902991 | 0 | predicted protein [Populus trichocarpa] |  |
| EY685743 | S44232573 | 5 | 28 | 5.403105439 | 25.91102 | 2.261705345 | 0 | peptide transporter ptr1 |  |
| EY686493 | S44233205 | 14 | 2 | 15.12869523 | 1.850787 | -3.031076404 | 0 | cs00-c2-003-054-a11- sweet orange greenhouse plant citrus sinensis mrna |  |
| EY687058 | S44233322 | 16 | 3 | 17.28993741 | 2.776181 | -2.638758981 | 0 | adp-ribosylation factor 1 |  |
| EY687139 | S44233403 | 156 | 54 | 168.5768897 | 49.97126 | -1.754236199 | 0 | lrr receptor-like serine threonine-protein kinase fei 2 flags: precursor |  |
| EY687309 | S44233573 | 18 | 5 | 19.45117958 | 4.626969 | -2.071718388 | 0 | nodulin 21 family protein |  |
| EY687567 | S44233719 | 7 | 67 | 7.564347615 | 62.00138 | 3.035012787 | 0 | disease resistance protein |  |
| EY687909 | S44233977 | 5 | 16 | 5.403105439 | 14.8063 | 1.454350423 | 0 | cs00-c2-003-050-d04- sweet orange greenhouse plant citrus sinensis mrna |  |
| EY688092 | S44234048 | 11 | 39 | 11.88683197 | 36.09036 | 1.602249118 | 0 | cysteine-rich receptor-like protein kinase 24� |  |
| EY689121 | S44234629 | 4 | 21 | 4.322484351 | 19.43327 | 2.168595941 | 0 | protein elc-like ame: full=vacuolar protein sorting-associated protein 23 homolog 2 ame: full=escrt-i complex subunit vps23 homolog 2 |  |
| EY689317 | S44234825 | 0 | 2 | 0 | 1.850787 | Inf | 0 | copia protein ame: full=gag-int-pol protein contains: ame: full=copia vlp protein contains: ame: full=copia protease |  |
| EY690635 | S44235821 | 48 | 7 | 51.86981222 | 6.477756 | -3.00132906 | 0 | gdsl esterase lipase at5g14450 ame: full=extracellular lipase at5g14450 flags: precursor |  |
| EY690744 | S44235930 | 22 | 9 | 23.77366393 | 8.328544 | -1.513228099 | 0 | expression vector complete sequence |  |
| EY691064 | S44236250 | 0 | 7 | 0 | 6.477756 | Inf | 0 | phosphoinositide phospholipase c 6 ame: full=phosphoinositide phospholipase plc6� |  |
| EY691433 | S44236619 | 8772 | 2924 | 9479.208183 | 2705.851 | -1.808683983 | 0 | homo sapiens chromosome 21 segment hs21c100 |  |
| EY691434 | S44236620 | 75 | 2 | 81.04658159 | 1.850787 | -5.452540172 | 0 | citrus sinensis dna binding protein (v03-3) complete cds |  |
| EY691949 | S44236813 | 495 | 83 | 534.9074385 | 76.80768 | -2.799966765 | 0 | burp domain-containing protein 6� |  |
| EY691980 | S44236844 | 4 | 16 | 4.322484351 | 14.8063 | 1.776278518 | 0 | ankyrin-1 ame: full=erythrocyte ankyrin ame: full=ankyrin-r |  |
| EY692005 | S44236869 | 1850 | 142 | 1999.149013 | 131.4059 | -3.927283918 | 0 | cs00-c2-003-086-d08- sweet orange greenhouse plant citrus sinensis mrna |  |
| EY692036 | S44236900 | 42 | 14 | 45.38608569 | 12.95551 | -1.808683983 | 0 | cs00-c2-003-086-h02- sweet orange greenhouse plant citrus sinensis mrna |  |
| EY692040 | S44236904 | 3 | 14 | 3.241863264 | 12.95551 | 1.99867094 | 0 | cs00-c2-003-086-h11- sweet orange greenhouse plant citrus sinensis mrna |  |
| EY692051 | S44236915 | 21 | 1 | 22.69304284 | 0.925394 | -4.616038905 | 0 | dna binding protein |  |
| EY692201 | S44237065 | 56 | 14 | 60.51478092 | 12.95551 | -2.223721482 | 0 | omega-3 fatty acid endoplasmic reticulum |  |
| EY692352 | S44237216 | 9910 | 3778 | 10708.95498 | 3496.138 | -1.614983837 | 0 | snakin-2 flags: precursor |  |
| EY692353 | S44237217 | 393 | 108 | 424.6840875 | 99.94252 | -2.087219482 | 0 | cs00-c2-003-059-e06- sweet orange greenhouse plant citrus sinensis mrna |  |
| EY693577 | S44237769 | 164 | 770 | 177.2218584 | 712.5532 | 2.007441149 | 0 | probable indole-3-acetic acid-amido synthetase ame: full=auxin-responsive gh3-like protein 1� |  |
| EY693754 | S44237834 | 1573 | 1607 | 1699.816971 | 1487.108 | -0.192870224 | 0 | cs00-c3-700-018-a08- sweet orange development stadium (1 of 6) citrus sinensis mrna |  |
| EY693806 | S44237886 | 34 | 134 | 36.74111699 | 124.0028 | 1.754904867 | 0 | protein |  |
| EY694299 | S44238281 | 8 | 34 | 8.644968703 | 31.46339 | 1.863741359 | 0 | conserved hypothetical protein [Ricinus communis] |  |
| EY695587 | S44239009 | 2 | 9 | 2.161242176 | 8.328544 | 1.94620352 | 0 | cs00-c3-700-038-b05- sweet orange development stadium (1 of 6) citrus sinensis mrna |  |
| EY695983 | S44239405 | 0 | 9 | 0 | 8.328544 | Inf | 0 | protein |  |
| EY696849 | S44240159 | 398 | 1016 | 430.087193 | 940.2 | 1.128338584 | 0 | ent-kaurene oxidase� |  |
| EY696998 | S44240308 | 5 | 37 | 5.403105439 | 34.23957 | 2.663803789 | 0 | cs00-c3-700-054-a09- sweet orange development stadium (1 of 6) citrus sinensis mrna |  |
| EY697055 | S44240365 | 2 | 19 | 2.161242176 | 17.58248 | 3.024206032 | 0 | peroxidase 4 flags: precursor |  |
| EY697656 | S44240854 | 4 | 6 | 4.322484351 | 5.552362 | 0.361241019 | 0 | uncharacterized protein at4g38062 |  |
| EY697902 | S44241100 | 2116 | 515 | 2286.594222 | 476.5778 | -2.262416772 | 0 | 3 -n-debenzoyl-2 -deoxytaxol n-benzoyltransferase� |  |
| EY698344 | S44241318 | 22 | 80 | 23.77366393 | 74.0315 | 1.638774994 | 0 | at5g10110-like protein |  |
| EY699454 | S44242316 | 22 | 239 | 23.77366393 | 221.1691 | 3.217713708 | 0 | hypothetical protein [Vitis vinifera] |  |
| EY699595 | S44242457 | 49 | 15 | 52.9504333 | 13.88091 | -1.93154073 | 0 | zinc transporter 1 ame: full=zrt irt-like protein 1 flags: precursor |  |
| EY699678 | S44242540 | 7 | 17 | 7.564347615 | 15.73169 | 1.056386437 | 0 | protein |  |
| EY699737 | S44242599 | 406 | 2017 | 438.7321617 | 1866.519 | 2.08893797 | 0 | receptor-like protein kinase feronia ame: full=protein sirene flags: precursor |  |
| EY701445 | S44243971 | 14 | 5 | 15.12869523 | 4.626969 | -1.709148309 | 0 | zinc ribbon 1 |  |
| EY701556 | S44244082 | 17 | 4 | 18.37055849 | 3.701575 | -2.311184323 | 0 | arg7\_phaauindole-3-acetic acid-induced protein arg7 |  |
| EY701702 | S44244228 | 273 | 111 | 295.009557 | 102.7187 | -1.522062756 | 0 | protein |  |
| EY701744 | S44244270 | 76 | 16 | 82.12720268 | 14.8063 | -2.471648995 | 0 | abc transporter g family member 15� |  |
| EY701875 | S44244401 | 47 | 478 | 50.78919113 | 442.3382 | 3.122556474 | 0 | probable lrr receptor-like serine threonine-protein kinase at1g56140 flags: precursor |  |
| EY701988 | S44244514 | 122 | 38 | 131.8357727 | 35.16496 | -1.906531306 | 0 | cs00-c3-700-055-g07- sweet orange development stadium (1 of 6) citrus sinensis mrna |  |
| EY702067 | S44244593 | 515 | 2033 | 556.5198602 | 1881.325 | 1.757244396 | 0 | oxygen-evolving enhancer protein chloroplastic� |  |
| EY702521 | S44244935 | 12 | 42 | 12.96745305 | 38.86654 | 1.58363344 | 0 | epidermis-specific secreted glycoprotein ep1 ame: full=52 54 kda medium protein flags: precursor |  |
| EY703041 | S44245343 | 583 | 2133 | 630.0020942 | 1973.865 | 1.647594695 | 0 | protein |  |
| EY703785 | S44245863 | 141 | 468 | 152.3675734 | 433.0843 | 1.507091885 | 0 | receptor-like protein kinase 5 ame: full=protein haesa flags: precursor |  |
| EY703947 | S44246025 | 26 | 210 | 28.09614828 | 194.3327 | 2.790084318 | 0 | receptor-like protein kinase 2 flags: precursor |  |
| EY704298 | S44246376 | 84 | 351 | 90.77217138 | 324.8132 | 1.839288316 | 0 | cs00-c3-701-031-d01- sweet orange development stadium (2 of 6) citrus sinensis mrna |  |
| EY704314 | S44246392 | 11 | 4 | 11.88683197 | 3.701575 | -1.6831531 | 0 | trehalose-phosphate phosphatase |  |
| EY704766 | S44246732 | 2 | 16 | 2.161242176 | 14.8063 | 2.776278518 | 0 | cs00-c3-701-036-c10- sweet orange development stadium (2 of 6) citrus sinensis mrna |  |
| EY704790 | S44246756 | 132 | 1197 | 142.6419836 | 1107.696 | 2.957091836 | 0 | ammonium transporter 1 member 2 ame: full= 1 2 |  |
| EY704963 | S44246929 | 10 | 38 | 10.80621088 | 35.16496 | 1.702277937 | 0 | growth-regulating factor 1 |  |
| EY705949 | S44247691 | 10 | 36 | 10.80621088 | 33.31417 | 1.624275425 | 0 | 29 kda ribonucleoprotein chloroplastic ame: full=cp29a flags: precursor |  |
| EY706085 | S44247827 | 152 | 563 | 164.2544054 | 520.9967 | 1.665342117 | 0 | pgr5-like protein chloroplastic flags: precursor |  |
| EY706402 | S44248144 | 81 | 7 | 87.53030812 | 6.477756 | -3.756216563 | 0 | major allergen pru ar 1 ame: allergen=pru ar 1 |  |
| EY706514 | S44248256 | 63 | 3 | 68.07912853 | 2.776181 | -4.616038905 | 0 | snakin-1 flags: precursor |  |
| EY707340 | S44249082 | 366 | 52 | 395.5073181 | 48.12047 | -3.038981602 | 0 | cytochrome p450 76c1 |  |
| EY708405 | S44250035 | 135 | 521 | 145.8838469 | 482.1301 | 1.724602483 | 0 | proline-rich protein |  |
| EY708789 | S44250209 | 32 | 10 | 34.57987481 | 9.253937 | -1.901793387 | 0 | cytochrome p450 98a2 |  |
| EY709847 | S44250944 | 259 | 74 | 279.8808618 | 68.47914 | -2.031076404 | 0 | myrcene chloroplastic flags: precursor |  |
| EY710037 | S44251134 | 5165 | 1383 | 5581.407919 | 1279.82 | -2.124688674 | 0 | germin-like protein subfamily 1 member 8 flags: precursor |  |
| EY710063 | S44251160 | 11 | 4 | 11.88683197 | 3.701575 | -1.6831531 | 0 | cs00-c3-701-111-e02- sweet orange development stadium (2 of 6) citrus sinensis mrna |  |
| EY710067 | S44251164 | 55 | 12 | 59.43415983 | 11.10472 | -2.420118695 | 0 | flavonol sulfotransferase-like ame: full= 47 |  |
| EY710123 | S44251220 | 2830 | 999 | 3058.157679 | 924.4684 | -1.725966952 | 0 | quercetin 3-o-methyltransferase 1� |  |
| EY710406 | S44251279 | 114 | 1859 | 123.190804 | 1720.307 | 3.803699559 | 0 | probable lrr receptor-like serine threonine-protein kinase at1g56140 flags: precursor |  |
| EY710417 | S44251290 | 638 | 2279 | 689.436254 | 2108.972 | 1.613051114 | 0 | photosystem i reaction center subunit chloroplastic� |  |
| EY710505 | S44251378 | 6 | 98 | 6.483726527 | 90.68859 | 3.806025862 | 0 | �upf0481 protein at3g02645 |  |
| EY710672 | S44251545 | 12 | 4 | 12.96745305 | 3.701575 | -1.808683983 | 0 | f-box family protein |  |
| EY710725 | S44251598 | 133 | 48 | 143.7226047 | 44.4189 | -1.694041417 | 0 | cs00-c3-701-086-e02- sweet orange development stadium (2 of 6) citrus sinensis mrna |  |
| EY711030 | S44251791 | 6 | 38 | 6.483726527 | 35.16496 | 2.439243531 | 0 | cs00-c3-702-002-h06- sweet orange development stadium (3 of 6) citrus sinensis mrna |  |
| EY711172 | S44251933 | 28 | 10 | 30.25739046 | 9.253937 | -1.709148309 | 0 | zinc finger ccch domain-containing protein 44� |  |
| EY711407 | S44252056 | 10 | 62 | 10.80621088 | 57.37441 | 2.408546734 | 0 | ests gb |  |
| EY713742 | S44253397 | 61 | 15 | 65.91788636 | 13.88091 | -2.247568224 | 0 | germin-like protein subfamily 2 member 4 flags: precursor |  |
| EY714307 | S44253738 | 2 | 10 | 2.161242176 | 9.253937 | 2.098206613 | 0 | cs00-c3-702-040-h06- sweet orange development stadium (3 of 6) citrus sinensis mrna |  |
| EY715074 | S44254169 | 1002 | 110 | 1082.78233 | 101.7933 | -3.411028561 | 0 | endochitinase a� |  |
| EY717749 | S44255626 | 28 | 134 | 30.25739046 | 124.0028 | 2.035012787 | 0 | cs00-c3-702-083-b04- sweet orange development stadium (3 of 6) citrus sinensis mrna |  |
| EY717756 | S44255633 | 26 | 302 | 28.09614828 | 279.4689 | 3.314243539 | 0 | ndf6 (ndh dependent flow 6) |  |
| EY718542 | S44256111 | 154 | 648 | 166.4156475 | 599.6551 | 1.84934198 | 0 | nad h-quinone oxidoreductase subunit chloroplastic ame: full=nad h dehydrogenase i subunit m� |  |
| EY718696 | S44256259 | 83 | 305 | 89.69155029 | 282.2451 | 1.653904519 | 0 | patatin-3-kuras 1 flags: precursor |  |
| EY718717 | S44256280 | 75 | 265 | 81.04658159 | 245.2293 | 1.597308377 | 0 | vitis vinifera contig whole genome shotgun sequence |  |
| EY719351 | S44256816 | 177 | 65 | 191.2699325 | 60.15059 | -1.668959219 | 0 | cs00-c3-702-089-b05- sweet orange development stadium (3 of 6) citrus sinensis mrna |  |
| EY719506 | S44256971 | 255 | 82 | 275.5583774 | 75.88229 | -1.860522914 | 0 | fatty acyl- reductase 2 ame: full=fatty acid reductase 2 ame: full=male sterility protein 2 |  |
| EY719738 | S44257203 | 2 | 17 | 2.161242176 | 15.73169 | 2.863741359 | 0 | cs00-c3-702-106-b10- sweet orange development stadium (3 of 6) citrus sinensis mrna |  |
| EY719828 | S44257293 | 3 | 16 | 3.241863264 | 14.8063 | 2.191316017 | 0 | �monooxygenase moxc |  |
| EY720079 | S44257432 | 288 | 61 | 311.2188733 | 56.44902 | -2.462909146 | 0 | taxadiene 5-alpha hydroxylase |  |
| EY721084 | S44258319 | 12 | 65 | 12.96745305 | 60.15059 | 2.21368383 | 0 | pentatricopeptide repeat-containing protein at2g35130 |  |
| EY721132 | S44258367 | 1505 | 6603 | 1626.334737 | 6110.375 | 1.909636677 | 0 | ammonium transporter 1 member 1� |  |
| EY723237 | S44259800 | 2 | 12 | 2.161242176 | 11.10472 | 2.361241019 | 0 | linear gramicidin synthetase subunit d includes: ame: full=atp-dependent tryptophan adenylase� |  |
| EY723878 | S44260343 | 10 | 37 | 10.80621088 | 34.23957 | 1.663803789 | 0 | cs00-c3-703-064-f03- sweet orange development stadium (4 of 6) citrus sinensis mrna |  |
| EY724756 | S44260997 | 231 | 59 | 249.6234713 | 54.59823 | -2.192827474 | 0 | cytochrome p450 93a3 ame: full=p450 cp5 |  |
| EY726741 | S44262427 | 245 | 827 | 264.7521665 | 765.3006 | 1.531384098 | 0 | cytochrome p450 76a1 ame: full=cyplxxvia1 ame: full=p-450eg8 |  |
| EY727291 | S44262641 | 14 | 63 | 15.12869523 | 58.29981 | 1.94620352 | 0 | xyloglucan endotransglucosylase hydrolase protein 9� |  |
| EY727445 | S44262795 | 2 | 2 | 2.161242176 | 1.850787 | -0.223721482 | 0 | cs00-c3-703-101-d09- sweet orange development stadium (4 of 6) citrus sinensis mrna |  |
| EY727536 | S44262886 | 563 | 215 | 608.3896725 | 198.9597 | -1.612519744 | 0 | flavonoid 3 -hydroxylase 1� |  |
| EY727543 | S44262893 | 657 | 175 | 709.9680547 | 161.9439 | -2.13225993 | 0 | protein |  |
| EY728025 | S44263039 | 12 | 51 | 12.96745305 | 47.19508 | 1.863741359 | 0 | pentatricopeptide repeat-containing protein at2g02980 |  |
| EY728036 | S44263050 | 1990 | 567 | 2150.435965 | 524.6983 | -2.035069272 | 0 | monooxygenase family protein |  |
| EY728050 | S44263064 | 2 | 11 | 2.161242176 | 10.17933 | 2.235710137 | 0 | cs00-c3-703-109-h04- sweet orange development stadium (4 of 6) citrus sinensis mrna |  |
| EY728066 | S44263080 | 51 | 188 | 55.11167548 | 173.974 | 1.658442028 | 0 | peroxidase 12� |  |
| EY728096 | S44263110 | 112 | 16 | 121.0295618 | 14.8063 | -3.031076404 | 0 | isoflavone-7-o-methyltransferase 9 ame: full=isoflavone-o-methyltransferase 9 ame: full=7 iomt-9 |  |
| EY728143 | S44263157 | 5 | 19 | 5.403105439 | 17.58248 | 1.702277937 | 0 | brassica rapa pekinensis clone complete sequence |  |
| EY728404 | S44263306 | 2 | 34 | 2.161242176 | 31.46339 | 3.863741359 | 0 | gdsl esterase lipase at5g42170 ame: full=extracellular lipase at5g42170 flags: precursor |  |
| EY728992 | S44263782 | 34 | 12 | 36.74111699 | 11.10472 | -1.726221822 | 0 | at3g08600 f17o14\_7 |  |
| EY730551 | S44265117 | 119 | 418 | 128.5939095 | 386.8146 | 1.588819887 | 0 | cs00-c3-704-031-d11- sweet orange development stadium (5 of 6) citrus sinensis mrna |  |
| EY732591 | S44266597 | 13 | 96 | 14.04807414 | 88.8378 | 2.660801301 | 0 | cs00-c3-704-064-b12- sweet orange development stadium (5 of 6) citrus sinensis mrna |  |
| EY732913 | S44266919 | 161 | 54 | 173.9799951 | 49.97126 | -1.799750858 | 0 | �kda heat shock mitochondrial� |  |
| EY733371 | S44267377 | 6 | 37 | 6.483726527 | 34.23957 | 2.400769383 | 0 | cs00-c3-704-054-g10- sweet orange development stadium (5 of 6) citrus sinensis mrna |  |
| EY734795 | S44268487 | 6115 | 1966 | 6607.997952 | 1819.324 | -1.860810659 | 0 | flocculation protein flo11� |  |
| EY735453 | S44268697 | 1843 | 7937 | 1991.584665 | 7344.85 | 1.882816253 | 0 | cellulose synthase-like protein h2 ame: full= slh2 |  |
| EY735515 | S44268759 | 10 | 3 | 10.80621088 | 2.776181 | -1.960687076 | 0 | cs00-c3-704-090-d10- sweet orange development stadium (5 of 6) citrus sinensis mrna |  |
| EY737122 | S44269918 | 18 | 4 | 19.45117958 | 3.701575 | -2.393646483 | 0 | cs00-c3-705-027-a12- sweet orange development stadium (6 of 6) citrus sinensis mrna |  |
| EY737138 | S44269934 | 24 | 1 | 25.93490611 | 0.925394 | -4.808683983 | 0 | probable 3-beta-hydroxysteroid-delta -isomerase ame: full=cholestenol delta-isomerase ame: full=delta -delta sterol isomerase� |  |
| EY738927 | S44271605 | 2 | 14 | 2.161242176 | 12.95551 | 2.58363344 | 0 | �disease resistance rpp13-like protein 3 |  |
| EY739702 | S44272044 | 77 | 28 | 83.20782376 | 25.91102 | -1.6831531 | 0 | nhl repeat-containing protein |  |
| EY744044 | S44274720 | 2150 | 371 | 2323.335339 | 343.3211 | -2.75856705 | 0 | isoflavone-7-o-methyltransferase 9 ame: full=isoflavone-o-methyltransferase 9 ame: full=7 iomt-9 |  |
| EY744205 | S44274881 | 2 | 9 | 2.161242176 | 8.328544 | 1.94620352 | 0 | gdsl esterase lipase at5g45670 ame: full=extracellular lipase at5g45670 flags: precursor |  |
| EY744519 | S44275195 | 2 | 12 | 2.161242176 | 11.10472 | 2.361241019 | 0 | trehalose-6-phosphate phosphatase |  |
| EY744529 | S44275205 | 12 | 55 | 12.96745305 | 50.89666 | 1.972675731 | 0 | mouse dna sequence from clone rp23-247j12 on chromosome complete sequence |  |
| EY745020 | S44275598 | 27 | 7 | 29.17676937 | 6.477756 | -2.171254062 | 0 | dna binding protein |  |
| EY745157 | S44275735 | 131 | 35 | 141.5613625 | 32.38878 | -2.127861466 | 0 | zinc finger protein constans-like 5 |  |
| EY745262 | S44275840 | 5 | 21 | 5.403105439 | 19.43327 | 1.846667846 | 0 | cs00-c5-003-027-f07- sweet orange greenhouse plant citrus sinensis mrna |  |
| EY745851 | S44276331 | 34 | 10 | 36.74111699 | 9.253937 | -1.989256228 | 0 | predicted protein [Populus trichocarpa] |  |
| EY746812 | S44276727 | 523 | 101 | 565.1648289 | 93.46477 | -2.596177135 | 0 | pectinesterase pectinesterase inhibitor includes: ame: full=pectinesterase inhibitor ame: full=pectin methylesterase inhibitor includes: ame: full=pectinesterase� |  |
| EY746898 | S44276813 | 0 | 12 | 0 | 11.10472 | Inf | 0 | transcription factor myb39 ame: full=myb-related protein 39� |  |
| EY747349 | S44277040 | 2 | 10 | 2.161242176 | 9.253937 | 2.098206613 | 0 | benzoate carboxyl methyltransferase ame: full=s-adenosyl-l-methionine:benzoic acid carboxyl methyltransferase |  |
| EY748174 | S44277641 | 117 | 36 | 126.4326673 | 33.31417 | -1.9241612 | 0 | uncharacterized glycosyltransferase at1g55740 |  |
| EY748266 | S44277733 | 11 | 33 | 11.88683197 | 30.53799 | 1.361241019 | 0 | volvox carteri nagariensis mrna for pherophorin-dz1 protein |  |
| EY748700 | S44277831 | 24 | 277 | 25.93490611 | 256.3341 | 3.305058184 | 0 | bahd acyltransferase at5g47980 |  |
| EY749305 | S44278114 | 266 | 1375 | 287.4452094 | 1272.416 | 2.146211986 | 0 | protein srg1� |  |
| EY749468 | S44278277 | 103 | 40 | 111.303972 | 37.01575 | -1.588293914 | 0 | vinorine synthase |  |
| EY749875 | S44278460 | 18912 | 1903 | 20436.70601 | 1761.024 | -3.536675956 | 0 | cs00-c5-003-080-g09- sweet orange greenhouse plant citrus sinensis mrna |  |
| EY750256 | S44278841 | 78 | 278 | 84.28844485 | 257.2595 | 1.609817372 | 0 | monoglyceride lipase� |  |
| EY750802 | S44279051 | 2543 | 1000 | 2748.019426 | 925.3937 | -1.570252944 | 0 | glutathione s-transferase mitochondrial ame: full=glutathione-dependent dehydroascorbate reductase 1� |  |
| EY750938 | S44279187 | 50 | 13 | 54.03105439 | 12.03012 | -2.167137953 | 0 | omega-3 fatty acid endoplasmic reticulum ame: full=indole-3-acetic acid-induced protein arg1 |  |
| EY750982 | S44279231 | 21 | 0 | 22.69304284 | 0 | -Inf | 0 | gdsl esterase lipase at3g27950 ame: full=extracellular lipase at3g27950 flags: precursor |  |
| EY751036 | S44279279 | 17 | 5 | 18.37055849 | 4.626969 | -1.989256228 | 0 | omega-3 fatty acid chloroplastic flags: precursor |  |
| EY751088 | S44279331 | 393 | 43 | 424.6840875 | 39.79193 | -3.415842229 | 0 | non-specific lipid-transfer protein� |  |
| EY751241 | S44279484 | 2 | 11 | 2.161242176 | 10.17933 | 2.235710137 | 0 | uncharacterized protein at5g12080 |  |
| EY751386 | S44279629 | 29 | 118 | 31.33801155 | 109.1965 | 1.800940572 | 0 | homeobox-leucine zipper protein athb-40 ame: full=homeodomain transcription factor athb-40 ame: full=hd-zip protein athb-40 |  |
| EY751751 | S44279882 | 88 | 453 | 95.09465573 | 419.2034 | 2.14021414 | 0 | protein srg1� |  |
| EY751843 | S44279974 | 32 | 6 | 34.57987481 | 5.552362 | -2.638758981 | 0 | protein |  |
| EY752456 | S44280469 | 95 | 408 | 102.6590033 | 377.5606 | 1.878848252 | 0 | galactokinase like protein |  |
| EY752482 | S44280495 | 120 | 1111 | 129.6745305 | 1028.112 | 2.987031024 | 0 | geranylgeranyl pyrophosphate synthetase chloroplastic� |  |
| EY752777 | S44280706 | 149 | 58 | 161.0125421 | 53.67284 | -1.584909007 | 0 | probable inactive purple acid phosphatase 27 flags: precursor |  |
| EY752884 | S44280813 | 31 | 108 | 33.49925372 | 99.94252 | 1.57696971 | 0 | protein phosphatase 2c 57� |  |
| EY753026 | S44280955 | 20 | 122 | 21.61242176 | 112.898 | 2.385087761 | 0 | abc transporter g family member 40� |  |
| EY753169 | S44280986 | 21 | 5 | 22.69304284 | 4.626969 | -2.29411081 | 0 | sni1 transcription repressor |  |
| EY753251 | S44281068 | 25 | 122 | 27.0155272 | 112.898 | 2.063159666 | 0 | probable wrky transcription factor 40 ame: full=wrky dna-binding protein 40 |  |
| EY753520 | S44281337 | 13 | 322 | 14.04807414 | 297.9768 | 4.406755678 | 0 | serine threonine-protein kinase bri1-like 2 ame: full=brassinosteroid insensitive 1-like protein 2 ame: full=protein vascular highway 1 flags: precursor |  |
| EY753573 | S44281390 | 403 | 1434 | 435.4902984 | 1327.015 | 1.607471798 | 0 | photosystem ii 22 kda chloroplastic ame: full=cp22 flags: precursor |  |
| EY754123 | S44281716 | 76 | 408 | 82.12720268 | 377.5606 | 2.200776347 | 0 | �cysteine-rich receptor-like protein kinase 20� |  |
| EY754293 | S44281886 | 8 | 41 | 8.644968703 | 37.94114 | 2.133830523 | 0 | dynein light chain cytoplasmic ame: full=8 kda dynein light chain |  |
| EY754954 | S44282239 | 4 | 48 | 4.322484351 | 44.4189 | 3.361241019 | 0 | cysteine-rich receptor-like protein kinase 22� |  |
| EY755595 | S44282432 | 20 | 108 | 21.61242176 | 99.94252 | 2.209237925 | 0 | cs12-c1-001-030-c09- sweet orange field plant a citrus sinensis mrna |  |
| EY756541 | S44282930 | 198 | 429 | 213.9629754 | 396.9939 | 0.891755736 | 0 | populus trichocarpa mrna |  |
| EY756862 | S44283139 | 3 | 13 | 3.241863264 | 12.03012 | 1.891755736 | 0 | hypothetical protein [Vitis vinifera] |  |
| EY756883 | S44283160 | 24 | 10 | 25.93490611 | 9.253937 | -1.486755888 | 0 | cs13-c1-001-014-g05- sweet orange field plant b citrus sinensis mrna |  |
| EY757009 | S44283286 | 101 | 247 | 109.1427299 | 228.5723 | 1.066434267 | 0 | �serine threonine-protein kinase receptor ame: full=s-receptor kinase� |  |
| EY757316 | S44283369 | 4 | 22 | 4.322484351 | 20.35866 | 2.235710137 | 0 | probable lrr receptor-like serine threonine-protein kinase at2g24230 flags: precursor |  |
| EY757389 | S44283442 | 1 | 9 | 1.080621088 | 8.328544 | 2.94620352 | 0 | cs13-c1-001-022-g08- sweet orange field plant b citrus sinensis mrna |  |
| EY757563 | S44283616 | 6 | 26 | 6.483726527 | 24.06024 | 1.891755736 | 0 | cs13-c1-001-024-h04- sweet orange field plant b citrus sinensis mrna |  |
| EY757951 | S44283780 | 20 | 84 | 21.61242176 | 77.73307 | 1.846667846 | 0 | cs13-c1-001-029-e03- sweet orange field plant b citrus sinensis mrna |  |
| EY758066 | S44283895 | 108 | 145 | 116.7070775 | 134.1821 | 0.201300106 | 0 | cs13-c1-001-030-g06- sweet orange field plant b citrus sinensis mrna |  |
| EY650223 | S44284117 | 78 | 268 | 84.28844485 | 248.0055 | 1.55696549 | 0 | vacuolar amino acid transporter 1 |  |
| EY650767 | S44284227 | 3 | 27 | 3.241863264 | 24.98563 | 2.94620352 | 0 | cs00-c1-100-014-f01- sweet orange greenhouse plant citrus sinensis mrna |  |
| EY652344 | S44284782 | 6 | 34 | 6.483726527 | 31.46339 | 2.278778859 | 0 | probable protein phosphatase 2c 60� |  |
| EY653213 | S44284881 | 11 | 40 | 11.88683197 | 37.01575 | 1.638774994 | 0 | chromatin modification-related protein meaf6� |  |
| EY653480 | S44285036 | 5 | 23 | 5.403105439 | 21.28406 | 1.977912379 | 0 | cs00-c1-100-044-h08- sweet orange greenhouse plant citrus sinensis mrna |  |
| EY653557 | S44285113 | 66 | 470 | 71.3209918 | 434.9351 | 2.608401345 | 0 | dna binding |  |
| EY656636 | S44285742 | 8 | 57 | 8.644968703 | 52.74744 | 2.609168532 | 0 | (+)-delta-cadinene synthase isozyme xc14� |  |
| EY656977 | S44285971 | 4 | 16 | 4.322484351 | 14.8063 | 1.776278518 | 0 | cs00-c1-100-060-f12- sweet orange greenhouse plant citrus sinensis mrna |  |
| EY657503 | S44286091 | 5 | 34 | 5.403105439 | 31.46339 | 2.541813265 | 0 | chlorophyll a-b binding protein chloroplastic ame: full=lhcii type i cab-37� |  |
| EY658481 | S44286383 | 9 | 33 | 9.725589791 | 30.53799 | 1.650747636 | 0 | unknown [Lycopersicon esculentum] |  |
| EY658914 | S44286522 | 22 | 114 | 23.77366393 | 105.4949 | 2.149736914 | 0 | 16kda membrane protein |  |
| EY659391 | S44286691 | 13 | 80 | 14.04807414 | 74.0315 | 2.397766895 | 0 | uncharacterized udp-glucosyltransferase at1g05670 |  |
| EY660812 | S44286908 | 3 | 29 | 3.241863264 | 26.83642 | 3.049297013 | 0 | cs00-c1-101-026-c04- sweet orange infected with xylella fastidiosa (stage 1 of 2) citrus sinensis mrna |  |
| EY660964 | S44287060 | 24 | 189 | 25.93490611 | 174.8994 | 2.753558442 | 0 | protein srg1� |  |
| EY661135 | S44287133 | 12 | 2 | 12.96745305 | 1.850787 | -2.808683983 | 0 | fasciclin-like arabinogalactan protein 11 flags: precursor |  |
| EY661170 | S44287168 | 1031 | 3933 | 1114.120342 | 3639.574 | 1.707864372 | 0 | calreticulin-3 flags: precursor |  |
| EY661453 | S44287227 | 10005 | 2314 | 10811.61398 | 2141.361 | -2.335981879 | 0 | cell wall protein dan4 ame: full=delayed anaerobic protein 4 flags: precursor |  |
| EY663158 | S44288050 | 8 | 32 | 8.644968703 | 29.6126 | 1.776278518 | 0 | protein |  |
| EY663234 | S44288126 | 3 | 17 | 3.241863264 | 15.73169 | 2.278778859 | 0 | cs00-c1-101-053-a03- sweet orange infected with xylella fastidiosa (stage 1 of 2) citrus sinensis mrna |  |
| EY664186 | S44288420 | 79 | 33 | 85.36906594 | 30.53799 | -1.483108111 | 0 | conserved hypothetical protein [Ricinus communis] |  |
| EY664689 | S44288475 | 8 | 62 | 8.644968703 | 57.37441 | 2.730474829 | 0 | s-norcoclaurine synthase flags: precursor |  |
| EY664805 | S44288591 | 234 | 1718 | 252.8653346 | 1589.826 | 2.65242812 | 0 | probable wrky transcription factor 70 ame: full=wrky dna-binding protein 70 |  |
| EY667068 | S44289062 | 90 | 315 | 97.25589791 | 291.499 | 1.58363344 | 0 | probable -dihydroxy-2-naphthoate octaprenyltransferase� |  |
| EY672705 | S44290891 | 4 | 15 | 4.322484351 | 13.88091 | 1.683169114 | 0 | cs00-c1-102-103-d01- sweet orange infected with xylella fastidiosa (stage 2 of 2) citrus sinensis mrna |  |
| EY673627 | S44291379 | 11 | 15 | 11.88683197 | 13.88091 | 0.223737495 | 0 | cs00-c1-102-012-c06- sweet orange infected with xylella fastidiosa (stage 2 of 2) citrus sinensis mrna |  |
| EY673801 | S44291553 | 83 | 360 | 89.69155029 | 333.1417 | 1.893092183 | 0 | nac domain ipr003441 |  |
| EY673884 | S44291636 | 21 | 216 | 22.69304284 | 199.885 | 3.138848598 | 0 | primary amine oxidase ame: full=amine oxidase flags: precursor |  |
| EY674035 | S44291661 | 7 | 69 | 7.564347615 | 63.85217 | 3.077448053 | 0 | transcriptional xre family |  |
| EY674830 | S44291882 | 8 | 45 | 8.644968703 | 41.64272 | 2.268131615 | 0 | unnamed protein product [Vitis vinifera] |  |
| EY676377 | S44291987 | 319 | 1478 | 344.718127 | 1367.732 | 1.988296459 | 0 | photosystem ii core complex proteins chloroplastic ame: full=l-arginine-metabolizing enzyme� |  |
| EY676846 | S44292120 | 7 | 35 | 7.564347615 | 32.38878 | 2.098206613 | 0 | cs00-c1-401-027-g02- sweet orange infected with citrus sinensis mrna |  |
| EY676885 | S44292159 | 56 | 217 | 60.51478092 | 200.8104 | 1.730474829 | 0 | serine threonine-protein kinase sapk2 ame: full=osmotic stress abscisic acid-activated protein kinase 2 |  |
| EY676894 | S44292168 | 9 | 36 | 9.725589791 | 33.31417 | 1.776278518 | 0 | transcription factor ilr3 ame: full=transcription factor en 133 ame: full=protein iaa-leucine resistant 3 ame: full=bhlh transcription factor bhlh105 ame: full=basic helix-loop-helix protein 105� |  |
| EY678260 | S44292442 | 4 | 26 | 4.322484351 | 24.06024 | 2.476718236 | 0 | af283537\_1lectin-related protein precursor |  |
| EY681750 | S44293244 | 0 | 10 | 0 | 9.253937 | Inf | 0 | PREDICTED: hypothetical protein [Vitis vinifera] |  |
| EY681754 | S44293248 | 11 | 1 | 11.88683197 | 0.925394 | -3.6831531 | 0 | remorin ame: full=dna-binding protein |  |
| EY681774 | S44293268 | 1977 | 535 | 2136.387891 | 495.0857 | -2.109423556 | 0 | superoxide dismutase 1 |  |
| EY682325 | S44293497 | 385 | 76 | 416.0391188 | 70.32992 | -2.564508604 | 0 | cs00-c1-650-031-f08- sweet orange young greenhouse plant citrus sinensis mrna |  |
| EY683964 | S44294030 | 57 | 348 | 61.59540201 | 322.037 | 2.386332 | 0 | thioredoxin m- chloroplastic� |  |
| EY684007 | S44294073 | 18 | 75 | 19.45117958 | 69.40453 | 1.835172207 | 0 | �cysteine-rich receptor-like protein kinase 30� |  |
| EY684012 | S44294078 | 8 | 115 | 8.644968703 | 106.4203 | 3.621768569 | 0 | protein brassinosteroid insensitive 1� |  |
| EY684201 | S44294267 | 76 | 272 | 82.12720268 | 251.7071 | 1.615813846 | 0 | �domain-containing gpi-anchored protein 2 flags: precursor |  |
| EY685678 | S44294372 | 191 | 25 | 206.3986278 | 23.13484 | -3.15729412 | 0 | mus musculus bac clone rp23-108h19 from complete sequence |  |
| EY686825 | S44294741 | 121 | 44 | 130.7551516 | 40.71732 | -1.6831531 | 0 | phytochrome kinase substrate 1-like |  |
| EY686851 | S44294767 | 16 | 3 | 17.28993741 | 2.776181 | -2.638758981 | 0 | cs00-c2-003-014-h01- sweet orange greenhouse plant citrus sinensis mrna |  |
| EY686862 | S44294778 | 225 | 1134 | 243.1397448 | 1049.397 | 2.109702252 | 0 | cs00-c2-003-030-a03- sweet orange greenhouse plant citrus sinensis mrna |  |
| EY686876 | S44294792 | 168 | 68 | 181.5443428 | 62.92677 | -1.528576063 | 0 | ring finger protein 126 |  |
| EY687702 | S44295142 | 3307 | 481 | 3573.613937 | 445.1144 | -3.005135728 | 0 | cs00-c2-003-092-e04- sweet orange greenhouse plant citrus sinensis mrna |  |
| EY687966 | S44295182 | 15 | 7 | 16.20931632 | 6.477756 | -1.323257155 | 0 | fasciclin-like arabinogalactan protein 10 flags: precursor |  |
| EY688491 | S44295483 | 10 | 0 | 10.80621088 | 0 | -Inf | 0 | patatin-08 ame: full=patatin group d-1 flags: precursor |  |
| EY690065 | S44295811 | 0 | 45 | 0 | 41.64272 | Inf | 0 | cs00-c2-003-083-h12- sweet orange greenhouse plant citrus sinensis mrna |  |
| EY690294 | S44295928 | 14 | 2 | 15.12869523 | 1.850787 | -3.031076404 | 0 | cs00-c2-003-084-a04- sweet orange greenhouse plant citrus sinensis mrna |  |
| EY691630 | S44296046 | 4 | 29 | 4.322484351 | 26.83642 | 2.634259513 | 0 | ubiquitin carboxyl-terminal hydrolase 12 ame: full=ubiquitin thioesterase 12 ame: full=ubiquitin-specific-processing protease 12 ame: full=deubiquitinating enzyme 12� |  |
| EY691718 | S44296134 | 12 | 3 | 12.96745305 | 2.776181 | -2.223721482 | 0 | endochitinase 1 flags: precursor |  |
| EY691800 | S44296216 | 257 | 3 | 277.7196196 | 2.776181 | -6.64438353 | 0 | dna binding protein |  |
| EY691883 | S44296299 | 0 | 10 | 0 | 9.253937 | Inf | 0 | (+)-delta-cadinene synthase isozyme xc14� |  |
| EY692431 | S44296399 | 6219 | 797 | 6720.382545 | 737.5388 | -3.18775247 | 0 | cs00-c2-003-071-e03- sweet orange greenhouse plant citrus sinensis mrna |  |
| EY692924 | S44296668 | 94 | 24 | 101.5783823 | 22.20945 | -2.193347833 | 0 | PREDICTED: hypothetical protein [Vitis vinifera] |  |
| EY694948 | S44297446 | 110 | 43 | 118.8683197 | 39.79193 | -1.578816441 | 0 | alpha-glucan water dikinase 2 flags: precursor |  |
| EY694974 | S44297472 | 76 | 29 | 82.12720268 | 26.83642 | -1.613668 | 0 | amp-activated protein gamma regulatory |  |
| EY697254 | S44297974 | 59 | 11 | 63.75664418 | 10.17933 | -2.646932913 | 0 | gcn5-related n-acetyltransferase brct |  |
| EY698191 | S44298141 | 19 | 72 | 20.53180067 | 66.62835 | 1.698276006 | 0 | multidrug and toxin extrusion protein 2� |  |
| EY701011 | S44298637 | 129 | 45 | 139.4001203 | 41.64272 | -1.743095641 | 0 | subtilisin-like protease ame: full=cucumisin-like serine protease flags: precursor |  |
| EY703404 | S44299028 | 5 | 70 | 5.403105439 | 64.77756 | 3.58363344 | 0 | protein |  |
| EY704562 | S44299178 | 2 | 19 | 2.161242176 | 17.58248 | 3.024206032 | 0 | protein yippee-like at4g27745 |  |
| EY708591 | S44299763 | 217 | 1875 | 234.4947761 | 1735.113 | 2.887402166 | 0 | chaperone protein dnaj chloroplastic� |  |
| EY708603 | S44299775 | 44 | 4 | 47.54732786 | 3.701575 | -3.6831531 | 0 | protein |  |
| EY708892 | S44299841 | 20 | 7 | 21.61242176 | 6.477756 | -1.738294655 | 0 | predicted protein [Populus trichocarpa] |  |
| EY709528 | S44300043 | 0 | 17 | 0 | 15.73169 | Inf | 0 | isoflavone-7-o-methyltransferase 9 ame: full=isoflavone-o-methyltransferase 9 ame: full=7 iomt-9 |  |
| EY710274 | S44300229 | 236 | 51 | 255.0265767 | 47.19508 | -2.433939189 | 0 | expansin-a5� |  |
| EY710311 | S44300266 | 1183 | 460 | 1278.374747 | 425.6811 | -1.586465789 | 0 | protein |  |
| EY710321 | S44300276 | 52 | 173 | 56.19229657 | 160.0931 | 1.510467028 | 0 | cytochrome p450 82a4 ame: full=p450 cp9 |  |
| EY710324 | S44300279 | 10 | 2 | 10.80621088 | 1.850787 | -2.545649577 | 0 | ubiquitin carboxyl-terminal hydrolase 13 ame: full=ubiquitin thioesterase 13 ame: full=ubiquitin-specific-processing protease 13 ame: full=deubiquitinating enzyme 13� |  |
| EY710329 | S44300284 | 445 | 1902 | 480.8763841 | 1760.099 | 1.871918523 | 0 | wrky transcription factor 6 ame: full=wrky dna-binding protein 6� |  |
| EY710358 | S44300313 | 6558 | 23011 | 7086.713094 | 21294.24 | 1.587274392 | 0 | squidulin ame: full=optic lobe calcium-binding protein ame: full=scabp |  |
| EY711363 | S44300548 | 2 | 13 | 2.161242176 | 12.03012 | 2.476718236 | 0 | cs00-c3-702-006-h03- sweet orange development stadium (3 of 6) citrus sinensis mrna |  |
| EY712586 | S44300973 | 10 | 48 | 10.80621088 | 44.4189 | 2.039312924 | 0 | 70 kda peptidyl-prolyl isomerase ame: full=peptidyl-prolyl cis-trans isomerase� |  |
| EY713683 | S44301510 | 106 | 35 | 114.5458353 | 32.38878 | -1.822358919 | 0 | vinorine synthase |  |
| EY713717 | S44301544 | 3669 | 1460 | 3964.798771 | 1351.075 | -1.553140017 | 0 | (+)-delta-cadinene synthase� |  |
| EY715375 | S44302306 | 83 | 292 | 89.69155029 | 270.215 | 1.591063646 | 0 | transposon protein |  |
| EY716813 | S44302848 | 190 | 317 | 205.3180067 | 293.3498 | 0.51476194 | 0 | cytochrome p450 78a3 |  |
| EY717225 | S44303050 | 10 | 42 | 10.80621088 | 38.86654 | 1.846667846 | 0 | nadph--cytochrome p450 reductase� |  |
| EY719891 | S44303790 | 127 | 304 | 137.2388782 | 281.3197 | 1.035521345 | 0 | conserved hypothetical protein [Ricinus communis] |  |
| EY721394 | S44304081 | 33 | 136 | 35.6604959 | 125.8535 | 1.81934724 | 0 | nudix hydrolase mitochondrial� |  |
| EY721877 | S44304228 | 125 | 280 | 135.077636 | 259.1102 | 0.93977725 | 0 | atp synthase subunit chloroplastic ame: full=f-atpase subunit beta ame: full=atp synthase f1 sector subunit beta |  |
| EY722494 | S44304397 | 336 | 99 | 363.0886855 | 91.61398 | -1.986682285 | 0 | (-)-germacrene d synthase |  |
| EY725647 | S44305218 | 30 | 131 | 32.41863264 | 121.2266 | 1.902810924 | 0 | leucoanthocyanidin dioxygenase� |  |
| EY725657 | S44305228 | 380 | 116 | 410.6360134 | 107.3457 | -1.935596095 | 0 | calcium-dependent protein |  |
| EY726474 | S44305390 | 13 | 24 | 14.04807414 | 22.20945 | 0.660801301 | 0 | probable lrr receptor-like serine threonine-protein kinase at3g47570 flags: precursor |  |
| EY727166 | S44305760 | 1621 | 7548 | 1751.686783 | 6984.872 | 1.99548885 | 0 | fructose-bisphosphate chloroplastic� |  |
| EY727625 | S44305883 | 47 | 10 | 50.78919113 | 9.253937 | -2.456382239 | 0 | pollen-specific protein sf3 |  |
| EY727745 | S44306003 | 52 | 23 | 56.19229657 | 21.28406 | -1.400599244 | 0 | zinc finger |  |
| EY727751 | S44306009 | 91 | 372 | 98.33651899 | 344.2465 | 1.807642689 | 0 | electron transporter thiol-disulfide exchange intermediate |  |
| EY727789 | S44306047 | 128 | 50 | 138.3194992 | 46.26969 | -1.579865292 | 0 | cytochrome p450 93a1 |  |
| EY728730 | S44306330 | 384 | 482 | 414.9584977 | 446.0398 | 0.104205354 | 0 | cs00-c3-704-007-g11- sweet orange development stadium (5 of 6) citrus sinensis mrna |  |
| EY729741 | S44306445 | 12 | 3 | 12.96745305 | 2.776181 | -2.223721482 | 0 | cs00-c3-704-020-g10- sweet orange development stadium (5 of 6) citrus sinensis mrna |  |
| EY734418 | S44307432 | 343 | 1330 | 370.6530331 | 1230.774 | 1.731424282 | 0 | probable wrky transcription factor 65 ame: full=wrky dna-binding protein 65 |  |
| EY735307 | S44307873 | 13 | 1 | 14.04807414 | 0.925394 | -3.9241612 | 0 | cs00-c3-704-093-f03- sweet orange development stadium (5 of 6) citrus sinensis mrna |  |
| EY735395 | S44307961 | 3 | 14 | 3.241863264 | 12.95551 | 1.99867094 | 0 | bordetella bronchiseptica strain complete genome segment 11 16 |  |
| EY740026 | S44308958 | 205 | 65 | 221.527323 | 60.15059 | -1.880833768 | 0 | �homolog subfamily b member 6 ame: full=mrj |  |
| EY742168 | S44309644 | 3 | 10 | 3.241863264 | 9.253937 | 1.513244112 | 0 | pheophorbide a chloroplastic� |  |
| EY742192 | S44309668 | 25 | 5 | 27.0155272 | 4.626969 | -2.545649577 | 0 | cs00-c3-705-024-b03- sweet orange development stadium (6 of 6) citrus sinensis mrna |  |
| EY743305 | S44309997 | 624 | 2619 | 674.3075588 | 2423.606 | 1.845676644 | 0 | cytochrome b6-f complex iron-sulfur chloroplastic ame: full=rieske iron-sulfur protein� |  |
| EY743805 | S44310497 | 1460 | 303 | 1577.706788 | 280.3943 | -2.492300152 | 0 | expansin-a10� |  |
| EY746288 | S44310871 | 24 | 32 | 25.93490611 | 29.6126 | 0.191316017 | 0 | cs00-c5-003-030-b07- sweet orange greenhouse plant citrus sinensis mrna |  |
| EY746298 | S44310881 | 1 | 10 | 1.080621088 | 9.253937 | 3.098206613 | 0 | maize gl1 homolog |  |
| EY746611 | S44311194 | 3 | 17 | 3.241863264 | 15.73169 | 2.278778859 | 0 | 21 kda protein ame: full= protein flags: precursor |  |
| EY747177 | S44311424 | 28 | 8 | 30.25739046 | 7.40315 | -2.031076404 | 0 | vacuolar protein sorting-associated protein 2 homolog 2� |  |
| EY747781 | S44311594 | 51 | 14 | 55.11167548 | 12.95551 | -2.088791902 | 0 | cs00-c5-003-048-h03- sweet orange greenhouse plant citrus sinensis mrna |  |
| EY747810 | S44311623 | 1599 | 132 | 1727.913119 | 122.152 | -3.822281586 | 0 | acidic endochitinase win6 flags: precursor |  |
| EY748394 | S44311871 | 0 | 11 | 0 | 10.17933 | Inf | 0 | auxin-induced protein 10a5 |  |
| EY748586 | S44312063 | 125 | 44 | 135.077636 | 40.71732 | -1.730074148 | 0 | abc transporter c family member 9� |  |
| EY748961 | S44312088 | 15 | 5 | 16.20931632 | 4.626969 | -1.808683983 | 0 | cs00-c5-003-064-e08- sweet orange greenhouse plant citrus sinensis mrna |  |
| EY750528 | S44312871 | 1 | 14 | 1.080621088 | 12.95551 | 3.58363344 | 0 | cs00-c5-003-096-c02- sweet orange greenhouse plant citrus sinensis mrna |  |
| EY750529 | S44312872 | 4 | 19 | 4.322484351 | 17.58248 | 2.024206032 | 0 | �pectate lyase 19 flags: precursor |  |
| EY750585 | S44312928 | 10 | 15 | 10.80621088 | 13.88091 | 0.361241019 | 0 | phenylalanine ammonia-lyase |  |
| EY751674 | S44313029 | 0 | 1 | 0 | 0.925394 | Inf | 0 | stamen-specific protein fil1 flags: precursor |  |
| EY752652 | S44313229 | 327 | 122 | 353.3630957 | 112.898 | -1.64613097 | 0 | pleiotropic drug resistance protein 2 ame: full= 2 |  |
| EY753878 | S44313461 | 12 | 48 | 12.96745305 | 44.4189 | 1.776278518 | 0 | probable lrr receptor-like serine threonine-protein kinase at3g47570 flags: precursor |  |
| EY754023 | S44313606 | 856 | 291 | 925.0116512 | 269.2896 | -1.780313125 | 0 | conserved hypothetical protein [Ricinus communis] |  |
| EY754792 | S44313913 | 4 | 20 | 4.322484351 | 18.50787 | 2.098206613 | 0 | wall-associated receptor kinase 5 flags: precursor |  |
| EY755110 | S44314007 | 9 | 57 | 9.725589791 | 52.74744 | 2.439243531 | 0 | nad h-dependent 6 -deoxychalcone synthase |  |
| EY756075 | S44314636 | 2 | 9 | 2.161242176 | 8.328544 | 1.94620352 | 0 | populus trichocarpa mrna |  |
| EY756177 | S44314738 | 0 | 21 | 0 | 19.43327 | Inf | 0 | probable lrr receptor-like serine threonine-protein kinase at1g12460 flags: precursor |  |
| EY756179 | S44314740 | 27 | 7 | 29.17676937 | 6.477756 | -2.171254062 | 0 | cs13-c1-001-005-e10- sweet orange field plant b citrus sinensis mrna |  |
| EY756665 | S44314890 | 12 | 41 | 12.96745305 | 37.94114 | 1.548868022 | 0 | cs13-c1-001-011-c07- sweet orange field plant b citrus sinensis mrna |  |
| EY757157 | S44315046 | 18 | 62 | 19.45117958 | 57.37441 | 1.560549827 | 0 | carotenoid (9 )-cleavage dioxygenase 1 ame: full= 1 ame: full=neoxanthin cleavage enzyme nc1� |  |
| EY757863 | S44315318 | 5 | 52 | 5.403105439 | 48.12047 | 3.154790141 | 0 | serine carboxypeptidase-like 42 flags: precursor |  |
| FE659083 | S46102672 | 4 | 1 | 4.322484351 | 0.925394 | -2.223721482 | 0 | c22 hong anliu sweet orange ssh library citrus sinensis cdna 5 mrna |  |
| FE659277 | S46102866 | 4 | 4 | 4.322484351 | 3.701575 | -0.223721482 | 0 | probable 1-acyl-sn-glycerol-3-phosphate acyltransferase 5 ame: full=lysophosphatidyl acyltransferase 5 |  |
| FE659288 | S46102877 | 495 | 103 | 534.9074385 | 95.31556 | -2.48850567 | 0 | 645 hong anliu sweet orange ssh library citrus sinensis cdna 5 mrna |  |
| EU861194 | S46915372 | 1801 | 276 | 1946.198579 | 255.4087 | -2.929779491 | 0 | glucose-1-phosphate adenylyltransferase large subunit 1 ame: full=alpha-d-glucose-1-phosphate adenyl transferase ame: full=adp-glucose pyrophosphorylase ame: full=agpase s ame: full=adp-glucose synthase |  |
| DC900129 | S47736427 | 2124 | 872 | 2295.239191 | 806.9433 | -1.508105208 | 0 | aldose 1-epimerase ame: full=galactose mutarotase |  |
| FC871426 | S49955080 | 1067 | 4095 | 1153.022701 | 3789.487 | 1.716581794 | 0 | dicyanin blue copper protein precursor |  |
| FC871875 | S49955529 | 13 | 48 | 14.04807414 | 44.4189 | 1.660801301 | 0 | serine-threonine protein plant- |  |
| FC921914 | S49955742 | 33 | 212 | 35.6604959 | 196.1835 | 2.459804853 | 0 | bahd acyltransferase at5g47980 |  |
| FC922338 | S49956166 | 235 | 896 | 253.9459556 | 829.1528 | 1.707116494 | 0 | disease resistance response protein 206 |  |
| FC922518 | S49956346 | 10 | 35 | 10.80621088 | 32.38878 | 1.58363344 | 0 | protein |  |
| FC922537 | S49956365 | 3 | 47 | 3.241863264 | 43.49351 | 3.745904869 | 0 | lotus japonicus genomic chromosome clone: complete sequence |  |
| FC922591 | S49956419 | 2 | 11 | 2.161242176 | 10.17933 | 2.235710137 | 0 | kn0aam1ac08rm1 slh citrus sinensis cdna clone mrna |  |
|  |  |  |  |  |  |  |  |  |  |
