# Supplementary material for: Transcriptome Profiling of Citrus Fruit Response to Huanglongbing Disease
Source: PLoS One. 2012 May 31;7(5):e38039. doi: 10.1371/journal.pone.0038039 (PMC3364978; doi:10.1371/journal.pone.0038039)
Supplement: Table S7 — 21 genes making the strongest contribution to each of four classes. Five genes with count and log2foldchange values also appear in two or more of Tables S1, S2, S3 and S6 as indicated. (HTM) [file pone.0038039.s007.htm]

| Table S7. 21 genes making the strongest contribution to each of four classes. Five genes highlighted in color also appear in Tables S1, S2, S3 and S6. | | | | | | | | | | | | |  |  |  |  |  |  |  |  |  |  |  |  |  |  |  |  |  |  |  |  |  |  |  |  |  |  |  |
|  | | | | | | | | | | | | | | | | | | | | | | | | | | | | | | | | | | | | | | | |
| GB id | id2 | Class | count CO | count SY | count AH | count AS | log2foldchange: Tables S1, S2, S3, S6 | | | | top hit At | description | annotation, blast2go |  | | | | | | | | | | | | | | | | | | | | | | | | | |
|  | |  |  | | | | CO-SY (S1) | CO-AS (S2) | CO-AH (S3) | AH-SY (S6) |  | | | | | | | | | | | | | | | | | | | | | | | | | | | | |
| EY752612 | S44280625 | MF-CO |  |  |  |  |  |  |  |  | AT5G23950 | stress.abiotic.cold' c2 domain-containing protein | c2 domain-containing protein | |  | | | | | | | | | | | | | | | | | | | | | | | | |
| EY752517 | S44280530 | MF-SY |  |  |  |  |  |  |  |  | AT3G16080 | 'protein.synthesis.ribosomal protein.eukaryotic.60S subunit.L37' | rl373\_arath ame: full=60s ribosomal protein l37-3 | | | |  |  |  |  |  |  |  |  |  |  |  |  |  |  |  |  |  |  |  |  |  |  |  |
| EY747804 | S44311617 | MF-CO |  |  |  |  |  |  |  |  | AT5G59320 | lipid metabolism.lipid transfer proteins etc' non-specific lipid-transfer protein | nltp1\_prudu ame: full=non-specific lipid-transfer protein 1 short=ltp 1 flags: precursor | | | | | | |  |  |  |  |  |  |  |  |  |  |  |  |  |  |  |  |  |  |  |  |
| EY747746 | S44311559 | MF-AH, MF-AS |  |  |  |  |  |  |  |  | AT1G69230 | nitrilase-associated protein | nitrilase-associated protein | |  |  |  |  |  |  |  |  |  |  |  |  |  |  |  |  |  |  |  |  |  |  |  |  |  |
| EY736504 | S44269636 | MF-CO |  |  |  |  |  |  |  |  | AT1G24260 | 'RNA.regulation of transcription.MADS box transcription factor family' | agl9\_pethy ame: full=agamous-like mads-box protein agl9 homolog ame: full=floral homeotic protein fbp2 ame: full=floral-binding protein 2 | | | | | | | | | | | |  |  |  |  |  |  |  |  |  |  |  |  |  |  |  |
| EY735479 | S44268723 | MF-CO |  |  |  |  |  |  |  |  | AT5G65260 | RNA.processing' polyadenylate-binding protein | pabp2\_mouse ame: full=polyadenylate-binding protein 2 short=poly -binding protein 2 ame: full=poly -binding protein ii short=pabii ame: full=polyadenylate-binding nuclear protein 1 ame: full=nuclear poly -binding protein 1 | | | | | | | | | | | | | | | | | | |  |  |  |  |  |  |  |  |
| EY733990 | S44307340 | MF-CO |  |  |  |  |  |  |  |  | AT1G24260 | 'RNA.regulation of transcription.MADS box transcription factor family' | agl9\_pethy ame: full=agamous-like mads-box protein agl9 homolog ame: full=floral homeotic protein fbp2 ame: full=floral-binding protein 2 | | | | | | | | | | | |  |  |  |  |  |  |  |  |  |  |  |  |  |  |  |
| EY727621 | S44305879 | MF-CO | 125150 | 24403 |  | 25970 | -1.9078665 | -2.0071209 |  |  | no AGI | sweet orange development stadium (4 of 6) citrus sinensis mrna | cs00-c3-703-103-e10- sweet orange development stadium (4 of 6) citrus sinensis mrna | | | | | | |  |  |  |  |  |  |  |  |  |  |  |  |  |  |  |  |  |  |  |  |
| EY722369 | S44259268 | MF-AH, MF-AS |  |  |  |  |  |  |  |  | AT2G28840 | cell.organisation' ankyrin repeat domain-containing protein | anr28\_human ame: full=serine threonine-protein phosphatase 6 regulatory ankyrin repeat subunit a short=serine threonine-protein phosphatase 6 regulatory subunit ars-a short=pp6-ars-a ame: full=ankyrin repeat domain-containing protein 28 ame: full=phosphatase interactor targeting protein hnrnp k short=pitk | | | | | | | | | | | | | | | | | | | | | | | | | | |
| EY719507 | S44256972 | MF-CO | 52762 | 8229 | 7544 | 14159 | -2.2300488 | -1.6361604 | -2.1265141 |  | AT5G02500 | protein.folding'; 'stress.abiotic.heat' heat shock cognate 70 kda protein | hsp71\_arath ame: full=heat shock cognate 70 kda protein 1 short= | | | | |  |  |  |  |  |  |  |  |  |  |  |  |  |  |  |  |  |  |  |  |  |  |
| EY717256 | S44303081 | MF-AH, MF-AS |  |  |  |  |  |  |  |  | AT3G46230 | stress.abiotic.heat' hsp17\_arath | hsp17\_arath ame: full= kda class i heat shock protein ame: full= kda heat shock protein 1 short= | | | | | | | |  |  |  |  |  |  |  |  |  |  |  |  |  |  |  |  |  |  |  |
| EY710530 | S44251403 | MF-AH, MF-AS |  |  |  |  |  |  |  |  | AT4G09800 | 'protein.synthesis.ribosomal protein.eukaryotic.40S subunit.S18' | rs18\_arath ame: full=40s ribosomal protein s18 | | | |  |  |  |  |  |  |  |  |  |  |  |  |  |  |  |  |  |  |  |  |  |  |  |
| EY710358 | S44300313 | MF-SY | 3209 | 23011 | 6558 | 10626 | 3.2927894 | 1.98902241 | 1.71071565 | 1.5872744 | AT5G49480 | signalling.calcium' squidulin; optic lobe calcium-binding protein | cabo\_lolpe ame: full=squidulin ame: full=optic lobe calcium-binding protein ame: full=scabp | | | | | | | |  |  |  |  |  |  |  |  |  |  |  |  |  |  |  |  |  |  |  |
| EY701956 | S44244482 | MF-CO |  |  |  |  |  |  |  |  | AT3G51030 | 'redox.thioredoxin' | trxh\_ricco ame: full=thioredoxin h-type short=trx-h | | | |  |  |  |  |  |  |  |  |  |  |  |  |  |  |  |  |  |  |  |  |  |  |  |
| EY679621 | S44228215 | MF-SY |  |  |  |  |  |  |  |  | AT4G05320 | 'protein.degradation.ubiquitin.ubiquitin' | ubiq\_arath ame: full=ubiquitin | |  |  |  |  |  |  |  |  |  |  |  |  |  |  |  |  |  |  |  |  |  |  |  |  |  |
| EY675276 | S44224542 | MF-SY |  |  |  |  |  |  |  |  | AT5G01410 | Co-factor and vitamine metabolism' probable pyridoxal biosynthesis protein pdx1 | pdx1\_hevbr ame: full=probable pyridoxal biosynthesis protein pdx1 ame: full=ethylene-inducible protein hever | | | | | | | | |  |  |  |  |  |  |  |  |  |  |  |  |  |  |  |  |  |  |
| EY668836 | S44220412 | MF-SY |  |  |  |  |  |  |  |  | AT5G06570 | probable gibberellin receptor gid1 | gi1l3\_arath ame: full=probable gibberellin receptor gid1l3 ame: full=gid1-like protein 3 | | | | | | |  |  |  |  |  |  |  |  |  |  |  |  |  |  |  |  |  |  |  |  |
| DY305870 | S34125432 | MF-SY |  |  |  |  |  |  |  |  | AT3G62290 | protein.targeting.secretory pathway.unspecified' adp-ribosylation factor 2 | arf2\_orysj ame: full=adp-ribosylation factor 2 | | |  |  |  |  |  |  |  |  |  |  |  |  |  |  |  |  |  |  |  |  |  |  |  |  |
| DY305851 | S34125413 | MF-SY | 10955 | 49842 |  | 27867 | 2.63643388 | 1.6085877 |  |  | AT1G27730 | 'RNA.regulation of transcription.C2H2 zinc finger family' | zfp1\_wheat ame: full=zinc finger protein 1 ame: full=wzf1 | | | | |  |  |  |  |  |  |  |  |  |  |  |  |  |  |  |  |  |  |  |  |  |  |
| DY257296 | S34124574 | MF-AH, MF-AS |  |  |  |  |  |  |  |  | AT4G15910 | stress.abiotic.drought/salt' late embryogenesis abundant protein lea5 | lea5\_citsi ame: full=late embryogenesis abundant protein lea5 | | | | |  |  |  |  |  |  |  |  |  |  |  |  |  |  |  |  |  |  |  |  |  |  |
| DC900219 | S47736517 | MF-CO | 57256 | 2522 | 5774 | 6383 | -4.0541253 | -2.9035008 | -2.6302003 |  | AT4G21960 | misc.peroxidases' peroxidase 42 | per42\_arath ame: full=peroxidase 42 short=atperox p42 ame: full=prxr1 ame: full=atp1a atp1b flags: precursor | | | | | | | | |  | | | | | | | | | | | | | | | | |  |
|  |  |  |  |  |  |  |  |  |  |  |  |  |  |  |  |  |  |  |  |  |  |  |  |  |  |  |  |  |  |  |  |  |  |  |  |  |  |  |  |
