# Supplementary material for: Transcriptome Profiling of Citrus Fruit Response to Huanglongbing Disease
Source: PLoS One. 2012 May 31;7(5):e38039. doi: 10.1371/journal.pone.0038039 (PMC3364978; doi:10.1371/journal.pone.0038039)
Supplement: Table S8 — GO categories of transcripts found in significantly higher levels in pairwise comparisons (indicated as “up” and “down” groups), using Fisher's Exact Test. (HTM) [file pone.0038039.s008.htm]

Table�S7


# Table�S8

| Table S8. GO categories of transcripts found in significantly higher levels in pairwise comparisons (indicated as "up" and "down" groups), using Fisher's Exact Test. | | | | | | | | | |  |
|  |  |  |  |  |  |  |  |  |  |  |
| go | name | up group | down group | FDR | FWER | p-Value | #Test | #Ref | #notAnnotTest | #notAnnotRef |
| GO:0000160 | two-component signal transduction system (phosphorelay) | SY | all | 0.0233618 | 0.145893 | 6.42E-04 | 6 | 27 | 247 | 7375 |
|  |  |  |  |  |  |  |  |  |  |  |
| GO:0003824 | catalytic activity | SY | AH, AS | 0.0170217 | 0.0965339 | 4.12E-04 | 113 | 3400 | 82 | 4060 |
|  |  |  |  |  |  |  |  |  |  |  |
| GO:0004497 | monooxygenase activity | CO | all | 0.0282206 | 0.0876387 | 3.69E-04 | 9 | 81 | 181 | 7384 |
| GO:0004497 | monooxygenase activity | SY | all | 0.00915455 | 0.0491041 | 1.70E-04 | 11 | 79 | 242 | 7323 |
|  |  |  |  |  |  |  |  |  |  |  |
| GO:0004553 | hydrolase activity, hydrolyzing O-glycosyl compounds | SY | AH | 0.034247 | 0.0581726 | 2.69E-04 | 8 | 75 | 148 | 7424 |
| GO:0004553 | hydrolase activity, hydrolyzing O-glycosyl compounds | SY | all | 0.0178463 | 0.105546 | 3.78E-04 | 10 | 73 | 243 | 7329 |
| GO:0004553 | hydrolase activity, hydrolyzing O-glycosyl compounds | SY | AH, AS | 0.0114043 | 0.0581165 | 2.42E-04 | 9 | 74 | 186 | 7386 |
|  |  |  |  |  |  |  |  |  |  |  |
| GO:0004564 | beta-fructofuranosidase activity | SY | AH, AS | 0.0481388 | 0.359737 | 0.00122082 | 3 | 6 | 192 | 7454 |
|  |  |  |  |  |  |  |  |  |  |  |
| GO:0005261 | cation channel activity | AH | CO | 0.017777 | 0.00443438 | 2.25E-05 | 3 | 5 | 55 | 7592 |
|  |  |  |  |  |  |  |  |  |  |  |
| GO:0005576 | extracellular region | CO | AS | 0.00525259 | 0.0112605 | 4.10E-05 | 9 | 96 | 113 | 7437 |
| GO:0005576 | extracellular region | CO | SY | 7.4E-05 | 1.86E-05 | 1.08E-07 | 11 | 94 | 90 | 7460 |
| GO:0005576 | extracellular region | CO | all | 2.4E-05 | 5.96E-06 | 3.78E-08 | 15 | 90 | 175 | 7375 |
| GO:0005576 | extracellular region | SY | all | 0.00915455 | 0.047459 | 1.66E-04 | 12 | 93 | 241 | 7309 |
| GO:0005576 | extracellular region | SY | AH, AS | 0.0046399 | 0.0195265 | 6.81E-05 | 11 | 94 | 184 | 7366 |
|  |  |  |  |  |  |  |  |  |  |  |
| GO:0005618 | cell wall | CO | AS | 0.00525259 | 0.0143409 | 6.43E-05 | 7 | 57 | 115 | 7476 |
| GO:0005618 | cell wall | CO | SY | 0.00223599 | 0.00390535 | 1.89E-05 | 7 | 57 | 94 | 7497 |
| GO:0005618 | cell wall | CO | all | 5.6E-04 | 7.01E-04 | 3.31E-06 | 10 | 54 | 180 | 7411 |
|  |  |  |  |  |  |  |  |  |  |  |
| GO:0005773 | vacuole | SY | all | 0.0116157 | 0.0646095 | 2.52E-04 | 10 | 69 | 243 | 7333 |
| GO:0005773 | vacuole | SY | AH, AS | 0.0432684 | 0.30022 | 8.52E-04 | 8 | 71 | 187 | 7389 |
|  |  |  |  |  |  |  |  |  |  |  |
| GO:0005975 | carbohydrate metabolic process | SY | AH, AS | 0.0174522 | 0.109405 | 4.89E-04 | 23 | 403 | 172 | 7057 |
|  |  |  |  |  |  |  |  |  |  |  |
| GO:0005996 | monosaccharide metabolic process | SY | AH | 0.0285061 | 0.0350056 | 1.55E-04 | 13 | 181 | 143 | 7318 |
| GO:0005996 | monosaccharide metabolic process | SY | AH, AS | 0.0481388 | 0.367047 | 0.00131807 | 13 | 181 | 182 | 7279 |
|  |  |  |  |  |  |  |  |  |  |  |
| GO:0006012 | galactose metabolic process | SY | AH | 0.0285061 | 0.0305941 | 1.41E-04 | 6 | 34 | 150 | 7465 |
| GO:0006012 | galactose metabolic process | SY | AH, AS | 0.0174522 | 0.108489 | 4.72E-04 | 6 | 34 | 189 | 7426 |
|  |  |  |  |  |  |  |  |  |  |  |
| GO:0006091 | generation of precursor metabolites and energy | SY | AH, AS | 0.0107968 | 0.0525537 | 1.99E-04 | 16 | 210 | 179 | 7250 |
|  |  |  |  |  |  |  |  |  |  |  |
| GO:0006869 | lipid transport | AS | SY | 0.0378876 | 0.0187656 | 6.71E-05 | 4 | 31 | 45 | 7575 |
|  |  |  |  |  |  |  |  |  |  |  |
| GO:0006952 | defense response | AS | SY | 0.00714435 | 0.00178449 | 9.09E-06 | 6 | 72 | 43 | 7534 |
| GO:0006952 | defense response | SY | all | 0.00379346 | 0.0141248 | 4.52E-05 | 11 | 67 | 242 | 7335 |
|  |  |  |  |  |  |  |  |  |  |  |
| GO:0008519 | ammonium transmembrane transporter activity | SY | all | 0.00316158 | 0.0110046 | 3.57E-05 | 3 | 0 | 250 | 7402 |
| GO:0008519 | ammonium transmembrane transporter activity | SY | AH, AS | 0.00172609 | 0.00516489 | 1.63E-05 | 3 | 0 | 192 | 7460 |
|  |  |  |  |  |  |  |  |  |  |  |
| GO:0009055 | electron carrier activity | SY | AH, AS | 0.0425664 | 0.281012 | 8.01E-04 | 12 | 149 | 183 | 7311 |
|  |  |  |  |  |  |  |  |  |  |  |
| GO:0009507 | chloroplast | SY | AS | 0.0101399 | 0.0196201 | 8.26E-05 | 13 | 231 | 104 | 7307 |
| GO:0009507 | chloroplast | SY | all | 1.6E-04 | 7.84E-05 | 4.07E-07 | 25 | 219 | 228 | 7183 |
| GO:0009507 | chloroplast | SY | AH, AS | 1.5E-05 | 7.37E-06 | 4.85E-08 | 23 | 221 | 172 | 7239 |
|  |  |  |  |  |  |  |  |  |  |  |
| GO:0009521 | photosystem | SY | all | 0.00619276 | 0.0252589 | 1.00E-04 | 6 | 18 | 247 | 7384 |
| GO:0009521 | photosystem | SY | AH, AS | 0.00197466 | 0.00639711 | 2.32E-05 | 6 | 18 | 189 | 7442 |
|  |  |  |  |  |  |  |  |  |  |  |
| GO:0009522 | photosystem I | SY | AH | 0.0285061 | 0.0123794 | 4.90E-05 | 4 | 7 | 152 | 7492 |
| GO:0009522 | photosystem I | SY | all | 0.0134934 | 0.0777712 | 3.20E-04 | 4 | 7 | 249 | 7395 |
| GO:0009522 | photosystem I | SY | AH, AS | 0.00663395 | 0.0294118 | 1.17E-04 | 4 | 7 | 191 | 7453 |
|  |  |  |  |  |  |  |  |  |  |  |
| GO:0009536 | plastid | SY | AH, AS | 0.00663426 | 0.0310216 | 1.34E-04 | 36 | 716 | 159 | 6744 |
|  |  |  |  |  |  |  |  |  |  |  |
| GO:0009573 | chloroplast ribulose bisphosphate carboxylase complex | SY | AS | 0.00107855 | 0.00107797 | 3.48E-06 | 3 | 0 | 114 | 7538 |
| GO:0009573 | chloroplast ribulose bisphosphate carboxylase complex | SY | all | 0.00316158 | 0.0110046 | 3.57E-05 | 3 | 0 | 250 | 7402 |
| GO:0009573 | chloroplast ribulose bisphosphate carboxylase complex | SY | AH, AS | 0.00172609 | 0.00516489 | 1.63E-05 | 3 | 0 | 192 | 7460 |
|  |  |  |  |  |  |  |  |  |  |  |
| GO:0009579 | thylakoid | SY | AS | 1.0E-04 | 2.49E-05 | 1.47E-07 | 8 | 33 | 109 | 7505 |
| GO:0009579 | thylakoid | SY | all | 4.6E-05 | 1.15E-05 | 5.43E-08 | 11 | 30 | 242 | 7372 |
| GO:0009579 | thylakoid | SY | AH, AS | 2.3E-06 | 5.64E-07 | 3.58E-09 | 11 | 30 | 184 | 7430 |
|  |  |  |  |  |  |  |  |  |  |  |
| GO:0009628 | response to abiotic stimulus | SY | all | 0.0188075 | 0.115075 | 4.51E-04 | 15 | 154 | 238 | 7248 |
|  |  |  |  |  |  |  |  |  |  |  |
| GO:0009723 | response to ethylene stimulus | SY | all | 0.00316158 | 0.00675764 | 3.22E-05 | 6 | 14 | 247 | 7388 |
|  |  |  |  |  |  |  |  |  |  |  |
| GO:0009767 | photosynthetic electron transport chain | SY | AS | 0.00773089 | 0.00961709 | 3.40E-05 | 3 | 2 | 114 | 7536 |
| GO:0009767 | photosynthetic electron transport chain | SY | all | 0.00153947 | 0.00153828 | 5.68E-06 | 4 | 1 | 249 | 7401 |
| GO:0009767 | photosynthetic electron transport chain | SY | AH, AS | 6.7E-04 | 4.99E-04 | 2.00E-06 | 4 | 1 | 191 | 7459 |
|  |  |  |  |  |  |  |  |  |  |  |
| GO:0009835 | ripening | SY | AS | 0.0460299 | 0.148802 | 5.25E-04 | 3 | 8 | 114 | 7530 |
|  |  |  |  |  |  |  |  |  |  |  |
| GO:0009873 | ethylene mediated signaling pathway | SY | all | 0.00201718 | 0.00352384 | 1.63E-05 | 6 | 12 | 247 | 7390 |
|  |  |  |  |  |  |  |  |  |  |  |
| GO:0015101 | organic cation transmembrane transporter activity | SY | all | 0.00316158 | 0.0110046 | 3.57E-05 | 3 | 0 | 250 | 7402 |
| GO:0015101 | organic cation transmembrane transporter activity | SY | AH, AS | 0.00172609 | 0.00516489 | 1.63E-05 | 3 | 0 | 192 | 7460 |
|  |  |  |  |  |  |  |  |  |  |  |
| GO:0015695 | organic cation transport | SY | all | 0.00915455 | 0.0444234 | 1.39E-04 | 3 | 1 | 250 | 7401 |
| GO:0015695 | organic cation transport | SY | AH, AS | 0.0046399 | 0.0184148 | 6.39E-05 | 3 | 1 | 192 | 7459 |
|  |  |  |  |  |  |  |  |  |  |  |
| GO:0015696 | ammonium transport | SY | all | 0.00316158 | 0.0110046 | 3.57E-05 | 3 | 0 | 250 | 7402 |
| GO:0015696 | ammonium transport | SY | AH, AS | 0.00172609 | 0.00516489 | 1.63E-05 | 3 | 0 | 192 | 7460 |
|  |  |  |  |  |  |  |  |  |  |  |
| GO:0016021 | integral to membrane | SY | AH, AS | 0.0174522 | 0.111131 | 5.10E-04 | 20 | 327 | 175 | 7133 |
|  |  |  |  |  |  |  |  |  |  |  |
| GO:0016023 | cytoplasmic membrane-bounded vesicle | CO | AS | 4.3E-04 | 2.89E-04 | 1.68E-06 | 20 | 356 | 102 | 7177 |
| GO:0016023 | cytoplasmic membrane-bounded vesicle | CO | SY | 3.6E-04 | 2.68E-04 | 1.61E-06 | 18 | 358 | 83 | 7196 |
| GO:0016023 | cytoplasmic membrane-bounded vesicle | CO | all | 7.3E-04 | 0.00124179 | 5.64E-06 | 25 | 351 | 165 | 7114 |
|  |  |  |  |  |  |  |  |  |  |  |
| GO:0016052 | carbohydrate catabolic process | SY | AH, AS | 0.0457088 | 0.321963 | 0.00107866 | 11 | 133 | 184 | 7327 |
|  |  |  |  |  |  |  |  |  |  |  |
| GO:0016491 | oxidoreductase activity | SY | AH | 0.034247 | 0.0573286 | 2.63E-04 | 28 | 658 | 128 | 6841 |
| GO:0016491 | oxidoreductase activity | SY | all | 0.00140026 | 0.00104964 | 5.01E-06 | 45 | 641 | 208 | 6761 |
| GO:0016491 | oxidoreductase activity | SY | AH, AS | 0.00113886 | 0.00170683 | 7.76E-06 | 37 | 649 | 158 | 6811 |
|  |  |  |  |  |  |  |  |  |  |  |
| GO:0016709 | oxidoreductase activity, acting on paired donors, with incorporation or reduction of molecular oxygen, NADH or NADPH as one donor, and incorporation of one atom of oxygen | CO | AS | 0.0466538 | 0.130612 | 4.30E-04 | 4 | 19 | 118 | 7514 |
|  |  |  |  |  |  |  |  |  |  |  |
| GO:0016798 | hydrolase activity, acting on glycosyl bonds | SY | AH | 0.0285061 | 0.0228219 | 8.17E-05 | 10 | 101 | 146 | 7398 |
| GO:0016798 | hydrolase activity, acting on glycosyl bonds | SY | AS | 0.0409051 | 0.106395 | 2.79E-04 | 8 | 103 | 109 | 7435 |
| GO:0016798 | hydrolase activity, acting on glycosyl bonds | CO | all | 0.0290696 | 0.0967415 | 4.10E-04 | 10 | 101 | 180 | 7364 |
| GO:0016798 | hydrolase activity, acting on glycosyl bonds | SY | all | 0.00201718 | 0.00330104 | 1.55E-05 | 14 | 97 | 239 | 7305 |
| GO:0016798 | hydrolase activity, acting on glycosyl bonds | SY | AH, AS | 9.1E-04 | 9.08E-04 | 4.29E-06 | 13 | 98 | 182 | 7362 |
|  |  |  |  |  |  |  |  |  |  |  |
| GO:0016838 | carbon-oxygen lyase activity, acting on phosphates | CO | AS | 0.00392697 | 0.00684865 | 3.16E-05 | 5 | 19 | 117 | 7514 |
| GO:0016838 | carbon-oxygen lyase activity, acting on phosphates | CO | all | 0.00228007 | 0.00564065 | 2.00E-05 | 6 | 18 | 184 | 7447 |
|  |  |  |  |  |  |  |  |  |  |  |
| GO:0016984 | ribulose-bisphosphate carboxylase activity | SY | AS | 0.00107855 | 0.00107797 | 3.48E-06 | 3 | 0 | 114 | 7538 |
| GO:0016984 | ribulose-bisphosphate carboxylase activity | SY | all | 0.00316158 | 0.0110046 | 3.57E-05 | 3 | 0 | 250 | 7402 |
| GO:0016984 | ribulose-bisphosphate carboxylase activity | SY | AH, AS | 0.00172609 | 0.00516489 | 1.63E-05 | 3 | 0 | 192 | 7460 |
|  |  |  |  |  |  |  |  |  |  |  |
| GO:0019253 | reductive pentose-phosphate cycle | SY | AS | 0.0101399 | 0.019558 | 8.19E-05 | 4 | 12 | 113 | 7526 |
| GO:0019253 | reductive pentose-phosphate cycle | SY | all | 0.00645123 | 0.0286134 | 1.23E-04 | 5 | 11 | 248 | 7391 |
| GO:0019253 | reductive pentose-phosphate cycle | SY | AH, AS | 0.00257243 | 0.0089631 | 3.54E-05 | 5 | 11 | 190 | 7449 |
|  |  |  |  |  |  |  |  |  |  |  |
| GO:0019318 | hexose metabolic process | SY | AH | 0.0285061 | 0.0149637 | 6.82E-05 | 13 | 166 | 143 | 7333 |
| GO:0019318 | hexose metabolic process | SY | AH, AS | 0.0228763 | 0.147977 | 6.24E-04 | 13 | 166 | 182 | 7294 |
|  |  |  |  |  |  |  |  |  |  |  |
| GO:0019685 | photosynthesis, dark reaction | SY | AS | 0.0101399 | 0.0225566 | 1.06E-04 | 4 | 13 | 113 | 7525 |
| GO:0019685 | photosynthesis, dark reaction | SY | all | 0.00915455 | 0.0488764 | 1.69E-04 | 5 | 12 | 248 | 7390 |
| GO:0019685 | photosynthesis, dark reaction | SY | AH, AS | 0.00321595 | 0.0119874 | 4.91E-05 | 5 | 12 | 190 | 7448 |
|  |  |  |  |  |  |  |  |  |  |  |
| GO:0022900 | electron transport chain | SY | AH, AS | 0.0432684 | 0.299187 | 8.35E-04 | 9 | 89 | 186 | 7371 |
|  |  |  |  |  |  |  |  |  |  |  |
| GO:0022904 | respiratory electron transport chain | SY | AS | 0.0442506 | 0.129086 | 4.15E-04 | 5 | 37 | 112 | 7501 |
|  |  |  |  |  |  |  |  |  |  |  |
| GO:0030312 | external encapsulating structure | CO | AS | 0.00525259 | 0.0143409 | 6.43E-05 | 7 | 57 | 115 | 7476 |
| GO:0030312 | external encapsulating structure | CO | SY | 0.00223599 | 0.00390535 | 1.89E-05 | 7 | 57 | 94 | 7497 |
| GO:0030312 | external encapsulating structure | CO | all | 5.6E-04 | 7.01E-04 | 3.31E-06 | 10 | 54 | 180 | 7411 |
|  |  |  |  |  |  |  |  |  |  |  |
| GO:0031224 | intrinsic to membrane | SY | AS | 0.0442506 | 0.133955 | 4.53E-04 | 15 | 353 | 102 | 7185 |
| GO:0031224 | intrinsic to membrane | SY | AH, AS | 0.0170217 | 0.0970908 | 4.21E-04 | 21 | 347 | 174 | 7113 |
|  |  |  |  |  |  |  |  |  |  |  |
| GO:0031410 | cytoplasmic vesicle | CO | AS | 0.00112908 | 0.00129275 | 4.35E-06 | 20 | 380 | 102 | 7153 |
| GO:0031410 | cytoplasmic vesicle | CO | SY | 8.0E-04 | 9.27E-04 | 3.90E-06 | 18 | 382 | 83 | 7172 |
| GO:0031410 | cytoplasmic vesicle | CO | all | 0.00228007 | 0.00516582 | 1.66E-05 | 25 | 375 | 165 | 7090 |
|  |  |  |  |  |  |  |  |  |  |  |
| GO:0031982 | vesicle | CO | AS | 0.00112908 | 0.00141036 | 5.25E-06 | 20 | 385 | 102 | 7148 |
| GO:0031982 | vesicle | CO | SY | 8.0E-04 | 1.00E-03 | 4.64E-06 | 18 | 387 | 83 | 7167 |
| GO:0031982 | vesicle | CO | all | 0.00228007 | 0.00568398 | 2.05E-05 | 25 | 380 | 165 | 7085 |
|  |  |  |  |  |  |  |  |  |  |  |
| GO:0031988 | membrane-bounded vesicle | CO | AS | 4.3E-04 | 3.20E-04 | 1.75E-06 | 20 | 357 | 102 | 7176 |
| GO:0031988 | membrane-bounded vesicle | CO | SY | 3.6E-04 | 2.73E-04 | 1.68E-06 | 18 | 359 | 83 | 7195 |
| GO:0031988 | membrane-bounded vesicle | CO | all | 7.3E-04 | 0.00127181 | 5.92E-06 | 25 | 352 | 165 | 7113 |
|  |  |  |  |  |  |  |  |  |  |  |
| GO:0032787 | monocarboxylic acid metabolic process | SY | AH, AS | 0.0481388 | 0.365712 | 0.00129674 | 16 | 252 | 179 | 7208 |
|  |  |  |  |  |  |  |  |  |  |  |
| GO:0034357 | photosynthetic membrane | SY | AH, AS | 0.0470499 | 0.33749 | 0.0011722 | 5 | 27 | 190 | 7433 |
|  |  |  |  |  |  |  |  |  |  |  |
| GO:0042545 | cell wall modification | CO | AS | 0.00525259 | 0.0134083 | 5.43E-05 | 4 | 10 | 118 | 7523 |
| GO:0042545 | cell wall modification | CO | all | 0.0279016 | 0.0802989 | 3.03E-04 | 4 | 10 | 186 | 7455 |
|  |  |  |  |  |  |  |  |  |  |  |
| GO:0043094 | cellular metabolic compound salvage | SY | AH, AS | 0.0495878 | 0.383395 | 0.00143607 | 4 | 16 | 191 | 7444 |
|  |  |  |  |  |  |  |  |  |  |  |
| GO:0043623 | cellular protein complex assembly | CO | AH | 0.0418191 | 0.0142818 | 7.43E-05 | 6 | 48 | 96 | 7505 |
|  |  |  |  |  |  |  |  |  |  |  |
| GO:0044425 | membrane part | SY | AH, AS | 0.0157291 | 0.0828756 | 3.15E-04 | 30 | 578 | 165 | 6882 |
|  |  |  |  |  |  |  |  |  |  |  |
| GO:0045156 | electron transporter, transferring electrons within the cyclic electron transport pathway of photosynthesis activity | SY | AS | 0.040067 | 0.0953161 | 2.32E-04 | 2 | 0 | 115 | 7538 |
| GO:0045156 | electron transporter, transferring electrons within the cyclic electron transport pathway of photosynthesis activity | SY | AH, AS | 0.0423635 | 0.264463 | 6.46E-04 | 2 | 0 | 193 | 7460 |
|  |  |  |  |  |  |  |  |  |  |  |
| GO:0048046 | apoplast | CO | SY | 0.0100528 | 0.019905 | 9.50E-05 | 4 | 15 | 97 | 7539 |
| GO:0048046 | apoplast | CO | all | 0.00797056 | 0.0216807 | 7.83E-05 | 5 | 14 | 185 | 7451 |
|  |  |  |  |  |  |  |  |  |  |  |
| GO:0048492 | ribulose bisphosphate carboxylase complex | SY | AS | 0.00107855 | 0.00107797 | 3.48E-06 | 3 | 0 | 114 | 7538 |
| GO:0048492 | ribulose bisphosphate carboxylase complex | SY | all | 0.00316158 | 0.0110046 | 3.57E-05 | 3 | 0 | 250 | 7402 |
| GO:0048492 | ribulose bisphosphate carboxylase complex | SY | AH, AS | 0.00172609 | 0.00516489 | 1.63E-05 | 3 | 0 | 192 | 7460 |
|  |  |  |  |  |  |  |  |  |  |  |
| GO:0051258 | protein polymerization | CO | AH | 0.0418191 | 0.0206926 | 9.88E-05 | 4 | 15 | 98 | 7538 |
|  |  |  |  |  |  |  |  |  |  |  |
| GO:0055085 | transmembrane transport | SY | AH, AS | 0.0425664 | 0.278365 | 7.75E-04 | 9 | 88 | 186 | 7372 |
|  |  |  |  |  |  |  |  |  |  |  |
| GO:0055114 | oxidation reduction | SY | AS | 0.0101399 | 0.0212915 | 1.01E-04 | 17 | 380 | 100 | 7158 |
| GO:0055114 | oxidation reduction | SY | all | 0.00619276 | 0.0259761 | 1.10E-04 | 28 | 369 | 225 | 7033 |
| GO:0055114 | oxidation reduction | SY | AH, AS | 0.00113886 | 0.00167378 | 7.69E-06 | 26 | 371 | 169 | 7089 |
|  |  |  |  |  |  |  |  |  |  |  |
| GO:0071369 | cellular response to ethylene stimulus | SY | all | 0.00201718 | 0.00352384 | 1.63E-05 | 6 | 12 | 247 | 7390 |
|  |  |  |  |  |  |  |  |  |  |  |
| GO:0071554 | cell wall organization or biogenesis | CO | AS | 0.00128678 | 0.0019283 | 8.15E-06 | 7 | 40 | 115 | 7493 |
| GO:0071554 | cell wall organization or biogenesis | CO | all | 4.3E-04 | 3.22E-04 | 1.78E-06 | 9 | 38 | 181 | 7427 |
|  |  |  |  |  |  |  |  |  |  |  |
| GO:0071555 | cell wall organization | CO | AS | 4.3E-04 | 1.21E-04 | 6.66E-07 | 7 | 26 | 115 | 7507 |
| GO:0071555 | cell wall organization | CO | all | 3.9E-04 | 1.96E-04 | 1.01E-06 | 8 | 25 | 182 | 7440 |
|  |  |  |  |  |  |  |  |  |  |  |
